# Transcriptomic response to prolonged ethanol production in the cyanobacterium *Synechocystis* sp. PCC6803

**DOI:** 10.1186/1754-6834-7-21

**Published:** 2014-02-06

**Authors:** Dennis Dienst, Jens Georg, Thomas Abts, Lew Jakorew, Ekaterina Kuchmina, Thomas Börner, Annegret Wilde, Ulf Dühring, Heike Enke, Wolfgang R Hess

**Affiliations:** 1 Algenol Biofuels Germany GmbH, Magnusstraße 1, Berlin D-12489, Germany; 2 Faculty of Biology, Inst. Biology III, University of Freiburg, Schänzlestr 1, Freiburg D-79104, Germany; 3 Institute of Biology, Humboldt-University Berlin, Chausseestr 117, Berlin D-10115, Germany; 4 Current address: Department of Plant and Environmental Sciences, University of Copenhagen, Thorvaldsensvej 40, Frederiksberg C DK-1871, Denmark

**Keywords:** Biofuel, Cyanobacteria, Ethanol production, *Synechocystis*, Metabolic engineering, Synthetic biology, Transcription

## Abstract

**Background:**

The production of biofuels in photosynthetic microalgae and cyanobacteria is a promising alternative to the generation of fuels from fossil resources. To be economically competitive, producer strains need to be established that synthesize the targeted product at high yield and over a long time. Engineering cyanobacteria into forced fuel producers should considerably interfere with overall cell homeostasis, which in turn might counteract productivity and sustainability of the process. Therefore, in-depth characterization of the cellular response upon long-term production is of high interest for the targeted improvement of a desired strain.

**Results:**

The transcriptome-wide response to continuous ethanol production was examined in *Synechocystis* sp. PCC6803 using high resolution microarrays. In two independent experiments, ethanol production rates of 0.0338% (v/v) ethanol d^-1^ and 0.0303% (v/v) ethanol d^-1^ were obtained over 18 consecutive days, measuring two sets of biological triplicates in fully automated photobioreactors. Ethanol production caused a significant (~40%) delay in biomass accumulation, the development of a bleaching phenotype and a down-regulation of light harvesting capacity. However, microarray analyses performed at day 4, 7, 11 and 18 of the experiment revealed only three mRNAs with a strongly modified accumulation level throughout the course of the experiment. In addition to the overexpressed *adhA* (*slr1192*) gene, this was an approximately 4 fold reduction in *cpcB* (*sll1577*) and 3 to 6 fold increase in *rps8* (*sll1809*) mRNA levels. Much weaker modifications of expression level or modifications restricted to day 18 of the experiment were observed for genes involved in carbon assimilation (Ribulose bisphosphate carboxylase and Glutamate decarboxylase). Molecular analysis of the reduced *cpcB* levels revealed a post-transcriptional processing of the *cpcBA* operon mRNA leaving a truncated mRNA cpcA* likely not competent for translation. Moreover, western blots and zinc-enhanced bilin fluorescence blots confirmed a severe reduction in the amounts of both phycocyanin subunits, explaining the cause of the bleaching phenotype.

**Conclusions:**

Changes in gene expression upon induction of long-term ethanol production in *Synechocystis* sp. PCC6803 are highly specific. In particular, we did not observe a comprehensive stress response as might have been expected.

## Background

Cyanobacteria are considered to be important and promising resources for the production of biofuels, such as hydrogen [1], ethanol [2], isobutyraldehyde and isobutanol [3], ethylene [4], volatile isoprene hydrocarbons [5] and alkanes [6]. Several commercial companies have begun working toward the metabolic remodeling of genetically modified cyanobacteria [7]. To achieve economically feasible production rates, the following two goals need to be addressed: (i) the yield of the intended product is to be maximized, and (ii) the producer strains should be of considerable robustness to tolerate the product, which is frequently alien to their metabolism.

Indeed, genetic instability and the onset of severe stress responses have been reported. Thus far, two unicellular model strains of cyanobacteria have mainly been used in these studies, *Synechococcus* sp. PCC7942 and *Synechocystis* sp. PCC6803 (from now on *Synechocystis* 6803). A depressed growth rate and a yellow-green phenotype interpreted as severe metabolic stress was reported for an ethylene-producing strain of *Synechococcus* sp. PCC7942 [4]. A substantial and unspecific general stress response was found upon the external application of ethanol both at proteome [8], as well as transcriptome level in *Synechocystis* 6803 [9].

To be meaningful for the optimization of biofuel production from cyanobacteria, the actual response to the internal production of a metabolite should be analyzed. Here we focused on an engineered strain of *Synechocystis* 6803, which synthesizes ethanol from pyruvate by the sequential activity of overexpressed pyruvate decarboxylase (PDC) from *Zymomonas mobilis* and alcohol dehydrogenase (ADH) from *Synechocystis* 6803. Employing high-resolution microarrays we identified a remarkably focused remodeling of the transcriptome in the course of 18 days of continuous ethanol production. The response included a discoordinated operon expression between the phycocyanin *cpcB* and *cpcA* genes, fully consistent with the observed bleaching phenotype.

## Results

### Characterization of *Synechocystis* 6803 upon long-term ethanol production

Engineering cyanobacteria to produce ethanol from pyruvate is accomplished by coupled overexpression of the cytosolic enzymes PDC and ADH. The synthesized ethanol further accumulates in the growth medium, most likely as a result of diffusion from the interior of the cells [2].

**Figure 1:**
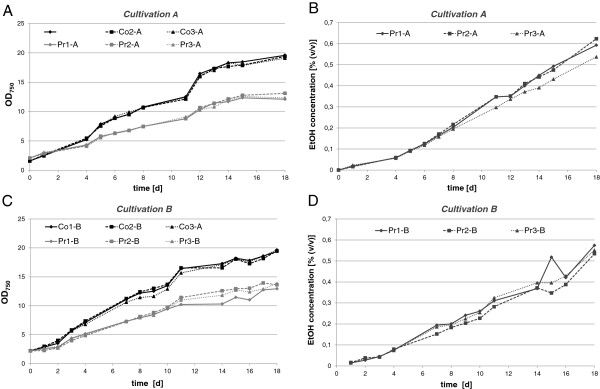
**Growth and ethanol production of an ethanologenic *Synechocystis *6803 strain (Pr) compared to the empty vector control strain (Co) over a period of 18 days in two independent cultivation experiments, A and B, and in three biological replicates each. (A)** Growth curves of triplicate cultures during cultivation A. At time points 4 d, 7 d, 11 d and 18 d, samples were taken for RNA preparation and transcriptome analysis. **(B)** Accumulation of ethanol in the producer strain (Pr) during cultivation A in three biological replicates. **(C)** Growth curves of triplicate cultures during cultivation B. At time points 14 d and 18 d, samples were taken for northern and immunoblot analysis. **(D)** Accumulation of ethanol in the Pr during cultivation B in three biological replicates. Growth during cultivation A: Co, 1.08 ± 0.017 optical density (OD) units d^-1^; Pr, 0.65 ± 0.021 OD units d^-1^. Growth during cultivation B: Co, 1.06 ± 0.010 OD units d^-1^; Pr, 0.66 ± 0.085 OD units d^-1^. Production during cultivation A: Pr, 0.0338 ± 0.002% ethanol (EtOH) d^-1^. Production during cultivation B: Pr, 0.0303 ± 0.002% EtOH d^-1^.

Appreciable intra- or extracellular ethanol concentrations, however, appear to be a rather unlikely stress parameter in the course of cyanobacterial evolution. Therefore, examination of long-term ethanol-related stress responses requires particular care with respect to experimental design and data validation. That is to minimize the chance of detecting non-ethanol effects resulting from imbalances in, for example, nutrient availability or physical parameters (pH, temperature, oxygen) that may arise upon long-term cultivation between producer and wild type. Therefore, and for accuracy of data validation and interpretation, two identical cultivation experiments were performed, comprising the ethanol producer strain #309 (Pr) and the empty vector control strain #621 (Co), each cultivated in triplicate in photobioreactors (PBRs). Temperature, pH and oxygen saturation of the cultures were monitored computationally using Crison MultiMeter units; CO_2_ supply was automatically controlled in dependence of the pH of the medium, which was thereby kept constant within the range between 7.25 and 7.35. As cultivation was performed in 12 h/12 h day/night cycles, samples were consistently taken at the same time point in the middle of the photoperiod, to exclude phase-dependent effects. RNA from cultivation A was used for microarray hybridization, whereas cultivation B was conducted as the validation run for northern blot hybridization, and where appropriate, protein analysis. Figure 1 provides an overview of the general growth parameters, demonstrating highly aligning growth dynamics between both cultivations. The measured increase of 1.08 ± 0.017 optical density at 750 nm (OD) units d^-1^ (cultivation A)/1.06 ± 0.010 OD units d^-1^ (cultivation B) for the control and 0.65 ± 0.021 OD units d^-1^ (cultivation A)/0.66 ± 0.085 OD units d^-1^ (cultivation B) for the producer strain indicates a significant (approximately 40%) defect in biomass accumulation in the producer strain.

The OD in the controls continued to increase during the whole course of the experiment at a steady pace (Figure 1A,C). An increase in OD was also observed for the ethanol producer strain but at a slower pace, and growth started to level off after approximately 2 weeks.

The production rates were quite similar in both Pr cultivations, with rates of 0.0338 ± 0.002% (v/v) EtOH d^-1^ (266.7 mg L^-1^ d^-1^) in cultivation A and 0.0303 ± 0.002% (v/v) EtOH d^-1^ (239.1 mg L^-1^ d^-1^) in cultivation B. These productivities were comparable to recently published data on a similar *Synechocystis* 6803 system (212 mg L^-1^ d^-1^; [10]) and several orders of magnitude higher than demonstrated for the pioneering *Synechococcus* PCC 7942 system (4.3 μg L^-1^ d^-1^; [2]).

**Figure 2:**
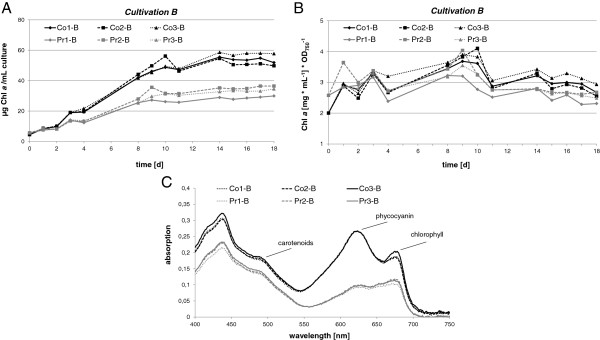
**Pigment content of ethanol producer (Pr) compared to isogenic empty vector control strain (Co) from cultivation B in three biological replicates. (A)** Chlorophyll content; **(B)** ratio between chlorophyll and optical density (OD)_750_ for each triplicate; **(C)** representative whole-cell absorption spectra at day 18 of the experiment.

Over 18 days of cultivation, an increase in ethanol concentration from 0% (v/v) to about 0.6% (v/v) was observed in the producer strains (Figure 1B,D), corresponding to a total yield of 4.7 g/L. Despite a visible and significant bleaching phenotype of strain Pr, the chlorophyll *a* (Chl *a*) content was nearly identical between the triplicates of ethanol producer and control strain in the early stages of cultivation (Figure 2A). However, between both strains differences in Chl *a* content rose strongly with increasing cultivation time, which is likely linked to the impaired growth of strain Pr, as indicated by the Chl/OD ratio depicted in Figure 2B and suggested downregulation of light-harvesting capacity.

These characteristics were clearly linked to the development of a bleaching phenotype.

### Microarray analysis of ethanol producer strains

Samples were taken for RNA preparation and subsequent transcriptome analysis at day 4, 7, 11 and 18 of the experiment. Because we used a full transcriptome microarray designed on the basis of previous dRNAseq analysis [11] and the prediction of non-coding RNAs (ncRNAs) [12,13], we were able to measure the differential expression not only of mRNAs, but also of putative antisense RNAs (asRNAs) and ncRNAs in cultures continuously producing ethanol. The complete microarray dataset is accessible [GEO: GSE49552]. The transcripts exhibiting the strongest fold changes in their accumulation are summarized in Table 1. Given the strong phenotype and the fact that 8,887 separate features (mRNAs, UTRs, asRNAs, ncRNAs, internal sense transcripts) can be detected by this microarray, a surprisingly narrow reorganization of the transcriptome was detected (Table 1).

**Table 1:** **List of transcripts exhibiting the strongest fold changes in transcript accumulation in response to ethanol production (expressed as log**_**2 **_**difference producer-control strain)**

			**(log**_**2**_**) fold change**		
**Annotation**	**Gene function**	**Day 4**	**Day 7**	**Day 11**	**Day 18**
slr1295-int1 *futA1*	Iron transport system (ferric ions)	−1.10	−1.08	−0.34	−3.13
slr1295-int3 *futA1*	Iron transport system (ferric ions)	−1.06	−1.16	−0.38	−3.09
sll1198-as1 *trmD* (3′ extension of *futA1* transcript)	tRNA (guanine-N1)-methyltransferase	−1.00	−1.01	−0.26	−2.98
sll0477-as2	Biopolymer transport ExbB-like protein	−0.43	−0.86	0.67	−2.77
sll1577 *cpcB*	Phycocyanin beta subunit	−1.97	−2.54	−2.16	−2.15
slr1192 adhA*	Alcohol dehydrogenase	1.95	1.85	1.49	1.82
NC-117	ncRNA ncl1390	0.10	0.25	0.83	1.93
slr1418-int2 *pyrD*	Dihydroorotate dehydrogenase	1.05	0.34	−0.56	2.03
NC-184	ncRNA ncl1740	0.22	0.12	−0.25	2.06
sll1809 *rps8*	Ribosomal protein S8	1.46	1.81	1.44	2.59
slr0254-as2	Hypothetical protein	0.45	0.31	−0.22	2.72
slr1897-5′UTR srrA**	Periplasmic sugar-binding protein of ABC transporter	2.28	1.90	2.58	3.29

The list is ordered according to fold change at day 18, with transcripts repressed in the upper part and those induced during ethanol production in the lower part. For comparison, values from all four time points are shown but only three mRNAs (*cpcB, adhA* and *rps8*) exhibited strong fold changes throughout the course of the experiment. int, gene internal transcript; as, detected transcript is in antisense orientation to mentioned gene. *Overexpressed in producer strain; **part of *petJ* promoter fragment used for overexpression of *adhA*.

**Figure 3:**
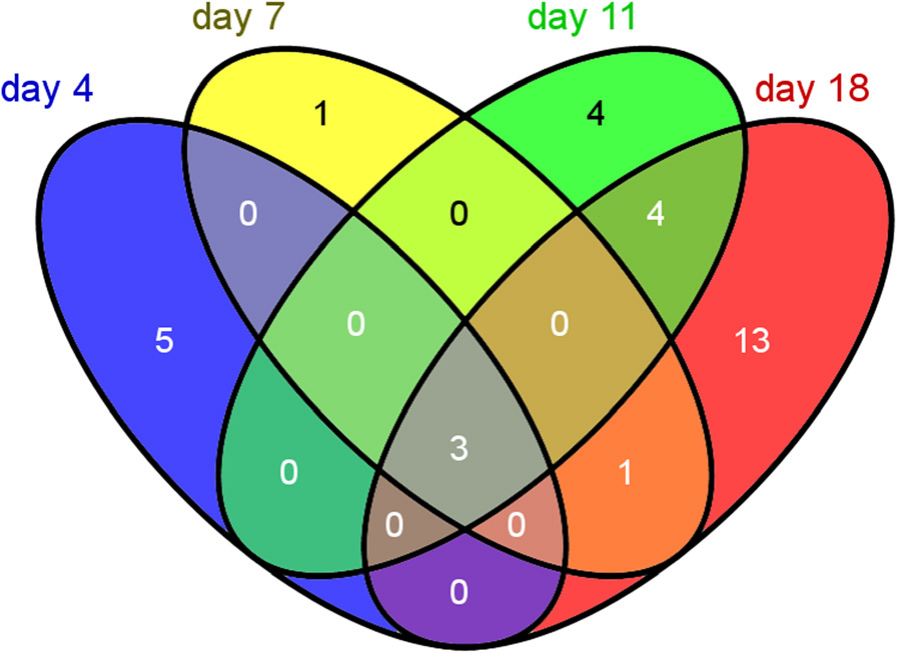
**Overlaps between significantly regulated (log**_**2 **_**fold change >0.7 between control and producer strain) mRNAs between the four time points.** The numbers of mRNAs are indicated. Only the transcripts of *adhA, rps8* and *cpcB* (center) showed significantly altered levels at all time points. Further details are listed in Table 1. The complete set of microarray data is presented in Additional file 1.

When applying a log_2_ fold-change limit of 0.9 across all time points, a total of 31 mRNAs showed differential accumulation between the producer and control strain. Among them, 17 were negatively affected by ethanol production, whereas 14 mRNAs were positively regulated. A considerable subset of regulated genes appears to be related to the ribosomes and photosynthesis. The Venn diagram in Figure 3 indicates only very few overlaps of regulated mRNAs between the four examined time points, whereas the most numerous and the strongest differences were observed at the late cultivation phase, particularly at day 18. However, three mRNAs (*adhA*, *rps8* and *cpcB*) showed significantly altered levels at all time points, further exhibiting the strongest changes among all mRNAs. Furthermore, the transcript encoding a further ribosomal subunit, *rpl28*, exhibited significantly enhanced levels in the producer compared to the wild type (WT) in the progressed (day 11; log_2_ fold change (FC) + 1.14) to late (day 18; log_2_ FC + 1.19) cultivation phase. Moreover, the mRNA levels for photosystem I subunit PsaC were significantly decreased at day 11 and day 18 in the producer (log_2_ FC −0.92 and −1.15, respectively).

**Figure 4:**
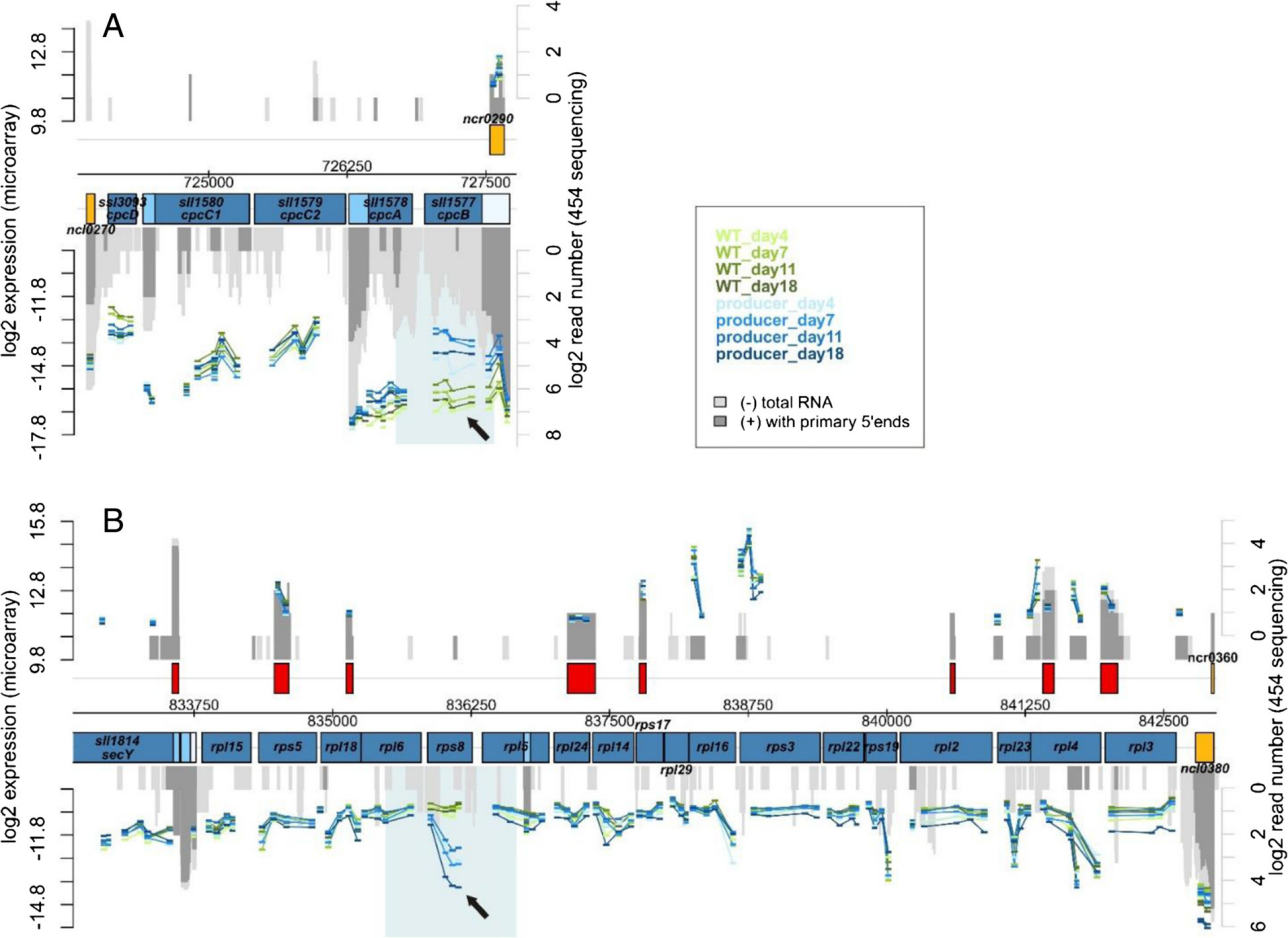
**Detail of gene expression analysis (microarrays) in *Synechocystis *6803 producer and isogenic control strains (wild type) 4 days (time point t1), 7 days (t2), 11 days (t3) and 18 days (t4) after start of the experiment. (A)** The genome section containing the phycocyanin operon *cpcBAC2C1D* is shown. The location of annotated genes is indicated by the blue boxes. The numbers of RNAseq reads from previous transcriptome analyses under standard conditions [11] are plotted for comparison (dark grey, primary reads; light grey, secondary reads). The normalized log_2_ expression values obtained by microarray analyses (normalized expression of biological duplicates A1 and A2 in two technical duplicates each) are plotted for each probe as bars in blue (producer A1 and A2, with increasing colour intensity from t_o_ to t_4_) or green (control incubation, with increasing colour intensity from t_o_ to t_4_). The scale for the microarray data is given at the left y-axis. Under ethanol biosynthesis conditions, *cpcB*– related transcripts decrease in abundance (black arrow). **(B)** Genome section containing the ribosomal protein operon (genes *rpl3-4-23-2-rps19-rpl22-rps3-rpl16-29-rps17-rpl14-24-5-rps8-rpl6-18-rps5-rpl15*). Under ethanol biosynthesis conditions, *rps8*–related transcripts increase as indicated by the arrow. WT, wild type.

At day 18, when the strongest differences were observed between producer and control strains, only ten features exhibited an at least four-fold change in transcript accumulation (Table 1). Among the most strongly induced genes compared to WT were *rps8* (*sll1809*), encoding the ribosomal protein S8 with a log_2_ FC of +2.59 at day 18 (Figure 4), compared to a value of +1.82 for the *adhA* (*slr1192*) gene, which encodes the ADH [14] that was used here for overexpression. Among the most strongly repressed genes compared to the WT were *cpcB* (*sll1577*), encoding the phycocyanin beta subunit of the phycobilisome with a log_2_ FC of −2.15 (Figure 4) and *futA2* (*idiA*, *slr0513*), encoding the iron transport system substrate-binding protein with a log_2_ FC of −1.61. Interestingly, there were several disparately regulated ncRNAs, for instance *ncl1740* (NC-184) and *ncl1390* (NC-117) with a log_2_ FC of +2.06 and +1.92, respectively. Among the ncRNAs accumulating at day 18 to a higher level in the control was *ncl1600* (NC-181) with a log_2_ FC of −1.43. The exact function of none of these ncRNAs is known, but we noticed previously that *ncl1600* (NC-181) is the top most-induced ncRNA under iron limitation [15]. Therefore, transcripts such as NC-181 and *futA2* rather indicate a beginning iron limitation in the control rather than a specific response in the producer strains. Another set of genes repressed in the producer strain encode subunits of photosystem I, *psaC* and *psaK1* with a log_2_ FC up to −1.15 at day 18.

An overview on the most strongly responding transcripts has been compiled in Table 1. The complete set of microarray data is visualized in Additional file 1 and can be downloaded from the database [GEO: GSE49552].

### Ethanol production induces discoordinated expression of the *cpcBA* operon

**Figure 5:**
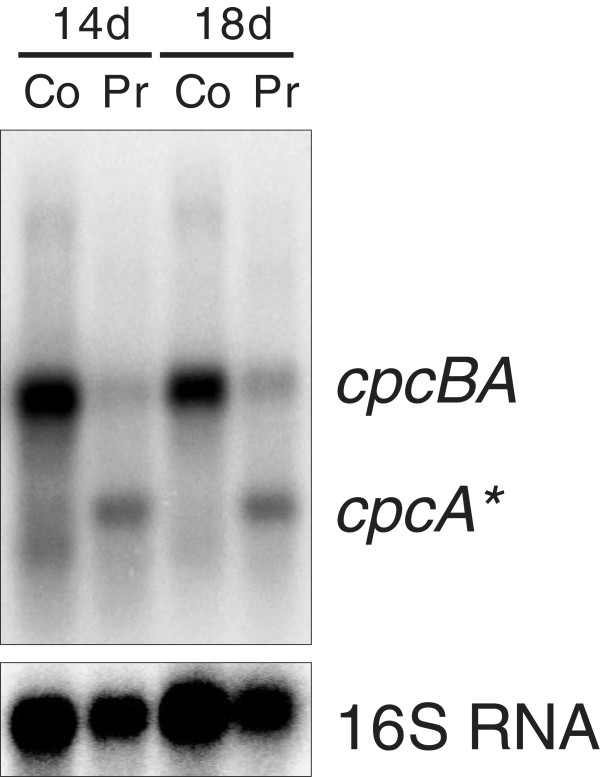
Differential accumulation of the transcripts *cpcBA *and cpcA* in the ethanol producer (Pr) and non-producer control strains (Co) of *Synechocystis *6803, after 14 or 18 days of cultivation in Crison photobioreactors. For loading control, blots were hybridized with a probe against the 16S rRNA.

The expression of phycobiliprotein genes in cyanobacteria is well investigated. Transcription of the *cpcBAC2C1D* operon in *Synechocystis* 6803 starts from a single major transcriptional start site (TSS) at position −253 with regard to the start codon of *cpcB*[11]. However, the mRNA accumulation level of *cpcBA*, the first two genes in the operon, was clearly higher in the wild type than for *cpcC2C1D*, suggesting possible regulation by imperfect termination of transcription 3′ to *cpcA* (Figure 4A). Under ethanol-producing conditions specific downregulation of *cpcB* but not *cpcA* occurred as revealed by the microarray results shown in Figure 4. Also the remaining operon appeared to be transcribed in a rather unchanged way. As transcription of this operon occurs from a TSS-mapped upstream of *cpcB*, it appeared puzzling how the mRNA for this gene could decrease, whereas the mRNA levels of the genes located downstream of *cpcB* in this operon remained stable. The search for an additional TSS upstream of *cpcA*, possibly linked to a cryptic promoter activated under ethanol-producing conditions was not successful. However, the analysis of transcript accumulation by northern blot analysis using a *cpcA*-specific probe not only verified the microarray data but also demonstrated the accumulation of a monocistronic *cpcA*-specific mRNA (cpcA*) not observed before (Figure 5).

**Figure 6:**
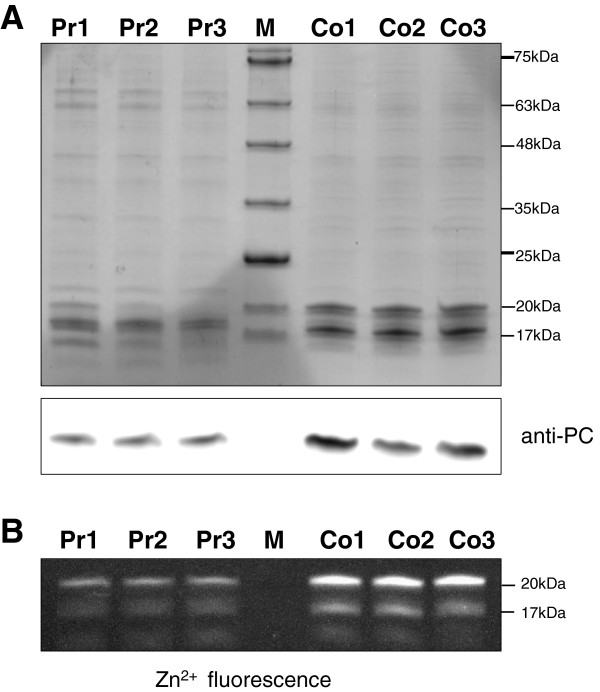
**Reduced accumulation of α phycocyanin in the producer (Pr1-3) in comparison to the control strain (Co1-3). (A)** Soluble protein extract (3 μg) from three replicate cultures for each strain were separated using a 16% Tricine-SDS polyacrylamide gel containing urea (upper panel) and subjected to western blot analysis (lower panel) using a phycocyanin-specific antibody [16]. **(B)** Zinc fluorescence of covalently attached bilins visualized on a UV transilluminator in a 16% Tricine-SDS gel. Molecular masses inferred from protein marker VI, (M; AppliChem, Darmstadt, Germany) are shown on the right. The samples were prepared at day 14 of the experiment.

In order to investigate the possibility of differential transcription of cpcA* from a hitherto undescribed alternative promoter, comparative 5′RACE analysis was conducted, using total RNA from producer and control strains as separate templates. This analysis yielded a 5′ end located 40 nt downstream of the *cpcA* start codon. As cpcA* is lacking the start codon it cannot become translated to give rise to the phycocyanin α subunit, but is very likely non-functional as an mRNA. As the signal leading to this result was not enhanced by treatment of the RNA with tobacco acid pyrophosphatase (TAP) (not shown), it could not result from activation of an unknown TSS but from the cleavage of a longer transcript. We propose therefore that the differential accumulation of *cpcB* and *cpcA* transcripts resulted from a post-transcriptional processing event leading to the strong reduction in *cpcB* mRNA level and the accumulation of a 5′ truncated *cpcA* mRNA, called here cpcA*. Western blot analysis as well as zinc-enhanced bilin fluorescence further demonstrated distinctly lower accumulation of the phycocyanin subunits alpha and beta in the EtOH-producing strains compared to the control (Figure 6). We conclude that the observed specific processing of the *cpcBA* mRNA led to the strongly reduced levels of alpha and beta phycocyanin subunits and was likely causative of the reduction in the light harvesting apparatus observed on spectroscopy (Figure 2).

### Ethanol production induces the transcription of a small part of a ribosomal gene cluster

**Figure 7:**
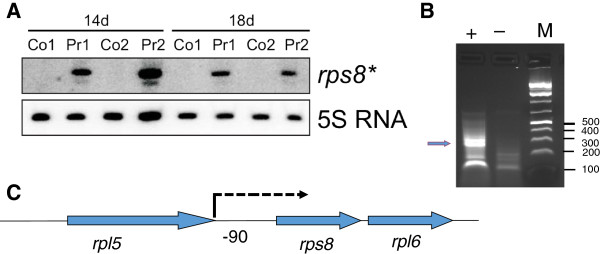
Accumulation of rps8*, an internal transcript within the *rps8/spc *operon covering part of the *rps8 *gene and the *rpl5-rps8 *intergenic spacer. (A) Accumulation of rps8* in the ethanol producer (Pr1 and Pr2) but not in the control strain (Co1 and Co2) after 14 and 18 days of cultivation in Crison photobioreactors. Total RNA was isolated from duplicate cultures, blotted and hybridized with a strand-specific RNA probe antisense to the *rps8* sequence. For loading control, blots were hybridized with a probe against the 5S rRNA. **(B)** 5′RACE mapping of rps8* 5′ ends revealed an initiation of transcription 91 to 96 nt upstream of the *rps8* start codon; plus sign (+) indicates treatment of RNA samples with tobacco acid pyrophosphatase; minus sign (−) indicates mock treatment. The bands excised for cloning and sequence analysis is indicated by the arrow. **(C)** Location of rps8* (arrow) within the *rps8/spc* operon.

One of the most prominent differences detected in the microarray analysis was the accumulation of a specific transcript within a large operon of around 9 kb encoding 18 different ribosomal proteins (genes *sll1799-sll1813*, *ssl3432*, *ssl3436* and *ssl3437*; *rpl3-4-23-2-rps19-rpl22-rps3-rpl16-29-rps17-rpl14-24-5-rps8-rpl6-18-rps5-rpl15*). This operon is followed by the *secY* gene, similar to the situation in *Escherichia coli*, where it is part of the *spc* operon of ribosomal proteins [17]. The major TSS of this operon in *Synechocystis* 6803 is located 337 nt upstream of the *rpl3* gene [11], whereas the *secY* gene is transcribed from a separate TSS. Here, we detected with three out of four microarray probes covering the *rps8* gene a significantly higher transcript accumulation in the producer strain than in the control (Figure 4B; log_2_ fold changes between 1.44 and 2.59, Table 1). Northern blot analysis using a single-stranded RNA probe against the *rps8* mRNA detected a transcript of about 350 nt. Its 5′ end was located by 5′RACE to a small window 91 to 96 nt upstream of the *rps8* start codon, close to the stop codon of the preceding gene *rpl5* (Figure 7). The *rps8* gene itself is 402 bp long, suggesting that the specific transcript that accumulates in the producer strain, called here rps8*, does not cover the open reading frame (ORF) over its entire length. Indeed, in the microarray analysis the 3′ end of *rps8* mRNA seems not to accumulate to a higher level during ethanol production (Figure 4B). Thus, rps8* is not competent for the synthesis of a full-length Rps8 protein.

## Discussion

The generation of microbial producer strains for the sustainable and economically feasible production of biofuels through photosynthetic processes is considered a challenging topic of research. Here we used a full transcriptome microarray developed on the basis of previous RNAseq and dRNAseq analyses [11] for the model cyanobacterium *Synechocystis* 6803. In contrast to previous studies in which a massive stress response was reported upon the external application of ethanol [8,9] we used a producer strain in which the ethanol was produced by an intracellular metabolic process. Our results demonstrate the host response on the internal ethanol synthesis to be unexpectedly narrow. In contrast to a comprehensive stress response, we identified mainly minor changes in transcript levels.

We detected a post-transcriptional regulatory component, involving a previously unknown RNA processing event in the *cpcBA* operon, leading to the generation of a truncated version of the *cpcA* transcript (cpcA*) by cleavage of the longer transcript at a specific position. According to its sequence, cpcA* is most likely not coding for a protein as it is 5′-truncated with regard to the *cpcA* reading frame and is interrupted by multiple stop codons in the other two reading frames. Discoordinated operon expression is frequently linked to the activity of regulatory small RNAs [18,19]. Recently, the successful metabolic engineering of *E. coli* was reported using synthetic small regulatory RNAs [20]. However, a native process that induces specific processing of the *cpcBA* operon mRNA and is leading to a translational nonfunctional cpcA* transcript is currently not known, nor is the possible function of cpcA*.

The second truncated mRNA that appeared specific for the ethanologenic conditions was rps8*. The protein Rps8 plays a major role in assembly of the 30S ribosomal subunit through interaction with 16S rRNA [21] as well as in the autoregulatory control of ribosomal protein expression from the *spc* operon in *E. coli*[22-24]. Although this operon is much longer in *Synechocystis* 6803 (effectively constituting a fusion of the S10 and *spc* operons known from enterobacteria), it is tempting to speculate that it plays a regulatory role as well. If so, the preceding intergenic spacer appears conspicuous with its length of 91 nt being by far the longest spacer in this 18-gene operon and constituting the 5′ UTR of *rps8*. One could speculate that this long 5′ UTR is the autoregulatory target of Rps8 in *Synechocystis* 6803 and that rps8* serves as competitive binding partner for surplus Rps8 subunits, in this way bypassing the default mechanism for autoregulatory control and allowing further Rps8 production.

In addition to the strongly responding mRNAs for *rps8* and *cpcB*, analyzed here in more detail, in total 240 transcripts were identified that showed mainly minor, but significant expression changes at some point during the experiment. Among these transcripts are many newly discovered transcripts not coding for protein. Some of these transcripts might be regulated by promoters that become induced or repressed at different stages of the production process. Therefore, this dataset can be used in conjunction with our previous genome-wide mapping of TSS [11] to construct expression cassettes that become active or repressed during different stages of the ethanol producing process.

## Conclusions

High ethanol production rates were obtained in engineered strains of *Synechocystis* 6803 over 18 consecutive days in fully automated PBRs. The physiological effects of high ethanol production include a delay in biomass accumulation, downregulation of light-harvesting capacity and the development of a bleaching phenotype. Microarray-based RNA profiling revealed a highly specific stress response, involving differential accumulation levels of only 31 mRNAs and a small number of non-coding RNAs. The molecular basis for the observed physiological effects of ethanol overproduction consists of a specific RNA processing event in the major light-harvesting operon encoding the phycocyanin subunits α and β. Thus, the molecular responses of engineered cyanobacteria upon sustained ethanol production are specific and appear well manageable for desired long-term cultivation.

## Materials and methods

### Culture media and growth conditions

The ethanologenic Pr strain #309 of *Synechocystis* 6803 and the isogenic wild-type Co strain #621 were cultivated in triplicate for 19 days in optimized PBRs containing 0.5 L BG11 medium [25] supplemented with 2 mM TES, 35 g/L instant-ocean seawater salts (Aquarium Systems Inc., France) and 10 μg/mL gentamycin. The lid was fitted with ports for incoming pH-, dissolved oxygen- and temperature-sensors as well as sampling ports and connections to in- and out-gas. Dissolved oxygen, pH and temperature were monitored by three-channel MultiMeter 44 devices (Crison Instruments, S. A., Barcelona, Spain). Cells were grown under day-night cycle conditions with a 12-h photoperiod. The light intensity was successively adapted to the increasing cell density (approximately 100 μmol photons m^-2^ s^-1^ per OD_750_ unit) and reached a maximum value of 1,000 μmol photons m^-2^ s^-1^. The culture temperature was controlled in a day-night cycle with 35°C daytime and 25°C night-time temperature. During the 12-h photoperiod, the liquid phase was discontinuously aerated with CO_2_-enriched air (10% CO_2_), pH-dependent and computer-controlled. At a culture pH above 7.35, the aeration started and incoming air flow ceased at a pH below 7.25. There was no aeration of the culture at night. Cells were constantly mixed by stirring with a magnetic stir bar (7 cm length) at 250 rpm. Samples from discrete stages of cultivation were subsequently subjected to microarray (transcriptome) analysis. Furthermore, growth, ethanol accumulation and pigment profiles were monitored over the cultivation period.

Induction of ethanol synthesis from the *petJ* promoter in #309 was triggered by centrifugation and resuspension of pre-cultures in copper-free medium. Thereby, pre-cultures of OD_750_ = 7 to 8 were diluted to a final OD_750_ of 2 (equivalent to about 10 mg chlorophyll * L^-1^) and subsequently divided into triplicates. In order to maintain maximal ethanol production, nutrient limitations were counter-steered by proportionate supplementation of a 100× nutrient concentrate when the nitrate concentration was below 50% of the BG11 concentration (determined with Quantofix Nitrate/Nitrite, Macherey-Nagel, Düren, Germany).

### Ethanol producer strain and quantification of ethanol accumulation

For generation of the ethanologenic strain #309, initially the dicistronic *pdc-adhII* cassette was *Eco*RI/*Bam*HI cut from plasmid pCB4-LR(TF)pa [2] and fused at its 5′ end (via *Eco*RI) to the promoter P*petJ* from *Synechocystis* 6803. The *Z. mobilis adhII* gene was replaced by the AdhA-encoding ORF *slr1192* from *Synechocystis* 6803 (*synADH*) via *Sac*I/*Pst*I. In the final construct [see Additional file 2], the ethanologenic cassette is integrated via *Sal*I/ *Pst*I into the self-replicating plasmid pVZ325, which is a derivative of pVZ321 [26] with an additional spectinomycin/streptomycin (Sp/Sm) resistance cassette (from pRL277 [27]), introduced into the *Xba*I site (resulting in pVZ321B) and a gentamycin (Gm) resistance cassette (from pVZ322 [26]), replacing the original kanamycin resistance cassette via *Cla*I/ *Xho*I. Plasmid pVZ325 was used for generating the empty-vector-control strain #621.

Primers used for cloning were:

synADH-fw: 5′-ATGAGCTCTCTGGATAAAACTAATAAAC -3′

synADH-rev: 5′- ATCTGCAGATCGAATGTCAAGCTTTCC -3′

PpetJ-fw: 5′- GTCGACGGGAATTGCTCTGGCAAC -3′

PpetJ-rev: 5′- GAATTCATTAGTTCTCCTTTCAAGG -3′

Gm-fw: 5′- ATCGATGCTCGAATTGACATAAGC -3′

Gm-rev: 5′- ATCGATGCTCGAATTGACATAAGC -3′

Quantification of ethanol in the liquid phase was accomplished by head-space gas chromatography (GC) using a Shimadzu GC-20104 gas chromatograph, with a medium-bore capillary column (FS-CS-624, length 30 m; I.D. 0.32 mm; film 1.8 μm; Chromatographie Service GmbH, Germany) and a flame ionization detector (FID). For analysis, 0.5 mL of culture were transferred into 20-mL GC vials for headspace autosampling (Shimadzu PAL LHS2-SHIM/AOC-5000) with screwed silicone-septum caps. For generation of a calibration curve, 0.5 mL calibrator solutions of 0.0125, 0.025, 0.059, 0.5, 1.0, 2.0, 3.0, 4.0, 5.0 and 10.0 mg*mL^-1^ ethanol were measured.

### Absorption spectra and determination of the chlorophyll content

Absorption spectra of whole cells were recorded using an UV-2450 PC UV–vis spectrophotometer (Shimadzu Deutschland GmbH, Duisburg, Germany). Chlorophyll contents were measured by spectrophotometry after extraction in 90% methanol [28].

### RNA preparation and northern blot hybridization

Samples from discrete stages of cultivation (as labelled in Figure 1) in PBRs were immediately quenched on ice and spun down at 0°C. RNA isolation and northern blot hybridization were performed essentially as described previously [29]. For analysis of the approximately 300-nt *rps8* transcript, total RNA was separated by electrophoresis using urea-polyacrylamide gels (8% acrylamide-bisacrylamide, 19:1; 8.3 M urea; 1× TBE (Tris-Borate-EDTA buffer) and transferred to nylon^+^ membranes using the Trans-Blot SD Semi-Dry Electrophoretic Transfer Cell (Biorad, Munich, Germany). The RNA probe for detection of *rps8*-specific transcripts was prepared using *in vitro* transcription with the MAXIscript kit (Invitrogen, Darmstadt, Germany) from a T7 promoter containing PCR fragment, which was amplified with the primer pair rps8-S-for 5′-ATGGCTTCAACAGACACAATTTC-3′ and T7-rps8-S-rev 5′-TAATACGACTCACTATAGGGACCAAATGTAACAAAGGAT-3′. The respective probe for the detection of *cpcA* transcripts was generated using the primers cpcA-fw: 5′- CAAACCCAAGGCAACAACTT −3′ and cpcA-T7: 5′- TAATACGACTCACTATAGGGGCCGTGGTTAGCTTTGATGT - 3′.

### Analyses by 5′RACE

The analyses of RNA primary and secondary 5′ ends followed previously established protocols [30] with the following modifications. For determination of TSS and RNA 5′ ends, 0.65 μg (for cpcA*) and 2.00 μg (for rps8*) of total RNA were subjected to Turbo DNase (Life Technologies, Darmstadt, Germany) digestion, followed by tobacco acid pyrophosphatase (TAP) treatment (Epicentre) and 5′-RNA linker addition using T4 RNA ligase (Epicentre, Madison, Wisconsin, U.S.). Two different oligonucleotides were used as 5′-RNA linkers, li1 in the case of cpcA* and adapterB in the case of rps8*. Synthesis of cDNA was performed with Superscript III reverse transcriptase (Life Technologies) using primers cpcA_R1 or rps8-R1, respectively. For the PCR amplification the RNA-linker-specific primers, Anchor-P1a’ (for cpcA*) or antiadapterB-fw (for rps8*) as well as primer cpcA_R2 or rps8-R1 were used. For the rps8* amplification, a second PCR with nested primers rps8-R2 and antiadapterBII-fw was performed. All reactions were carried out in accordance with the manufacturers’ recommendations.

The following oligonucleotides were used:

li1: 5′- GAUAUGCGCGAAUUCCUGUAGAACGAACACUAGAAGAAA −3′

adapterB: 5′ GUGAUCCAACCGACGCGACAAGCUAAUGCAAGANNN-3′

cpcA_R1: 5′- ATTGTCGGTCAGAGCTTTAG −3′,

cpcA_R2: 5′- TGCAAACCAGCATTAGCTTG −3′,

rps8-R1: 5′- ACCAAATGTAACAAAGGATTTCGCC

rps8-R2: 5′- CCTTCGCCGGTTTCAGAGT

Anchor-P1a′: 5′-CGAATTCCTGTAGAACGAACACTAGAAG-3′

antiadapterB-fw: 5′- TGATCCAACCGACGCGAC

antiadapterBII-fw: 5′- ACCGACGCGACAAGCTAATGC

### Gene expression microarray

The microarray design, hybridization procedure and data analysis have been described previously [11,12]. The microarray data are available in the database [GEO: GSE49552].

### SDS PAGE and immunoblot analyses

Soluble extracts of *Synechocystis* 6803 were prepared as described [31]. Proteins were separated by Tricine SDS-PAGE [32] using gels containing 6 M urea and transferred by electrophoresis onto nitrocellulose membranes. Blot membranes were incubated with specific primary antibodies and then with a secondary antibody (goat anti-rabbit IgG-peroxidase conjugate) (Sigma). Immunolabelled bands were visualized using the Immobilon western membrane chemiluminescence system (Millipore, Bedford, MA, USA). For detection of Zn^2+^ -induced fluorescence a 16% Tricine SDS-PAGE without urea containing 1 mM zinc acetate was used.

## Abbreviations

ADH: alcohol dehydrogenase; AsRNA: antisense RNA; Chl a: chlorophyll *a*; Co: control strain; FC: fold change; GC: gas chromatography; Gm: gentamycin; ncRNA: non-coding RNA; OD: optical density; ORF: open reading frame; PBR: photobioreactor; PDC: pyruvate decarboxylase; PR: producer strain; TAP: Tobacco acid pyrophosphatase; TSS: transcriptional start site; UTR: untranslated region; WT: wild type.

## Competing interests

The authors declare that they have no competing interests.

## Authors’ contributions

DD, JG, TA, LJ, EK and UD carried out the experimental analyses. DD, JG, AW and WRH participated in the microarray data analysis and drafted the manuscript. DD, JG, TB, AW, UD, HE and WRH conceived of the study, and participated in its design and coordination. All authors read and approved the final manuscript.

## Supplementary Material

Additional file 1 **Genome-wide overview on the log**_**2**_**-normalized expression values (left scale) from the microarray analysis of the producer strain versus control as indicated by the coloured lines.** Both strands are shown with the location of annotated genes (blue boxes), 5′-UTRs (light grey), internal sense RNAs (light blue), asRNAs (red) and intergenic ncRNA genes (yellow). The normalized log_2_ expression values obtained by microarray analyses (normalized expression of biological duplicates A1 and A2 in two technical duplicates each) are plotted for each probe as bars in blue (producer A1 and A2, with increasing colour intensity from t_o_ to t_4_) or green (control incubation, with increasing colour intensity from t_o_ to t_4_). The scale for the microarray data is given at the left y-axis. For comparison, the numbers of RNAseq reads from previous transcriptome analyses under standard conditions [11] are plotted (dark grey, primary reads; light grey, secondary reads). Click here for file

Additional file 2: Figure S1 Ethanologenic plasmid pVZ325-PpetJ-PDC-synADH of producer strain #309. Non-ethanologenic empty-vector-control plasmid pVZ325 of control strain #621. Click here for file
