# Supplementary figures and images for: Transcriptomic response to prolonged ethanol production in the cyanobacterium Synechocystis sp. PCC6803
Source: Biotechnol Biofuels. 2014 Feb 6;7:21. doi: 10.1186/1754-6834-7-21 (PMC3925133; doi:10.1186/1754-6834-7-21)

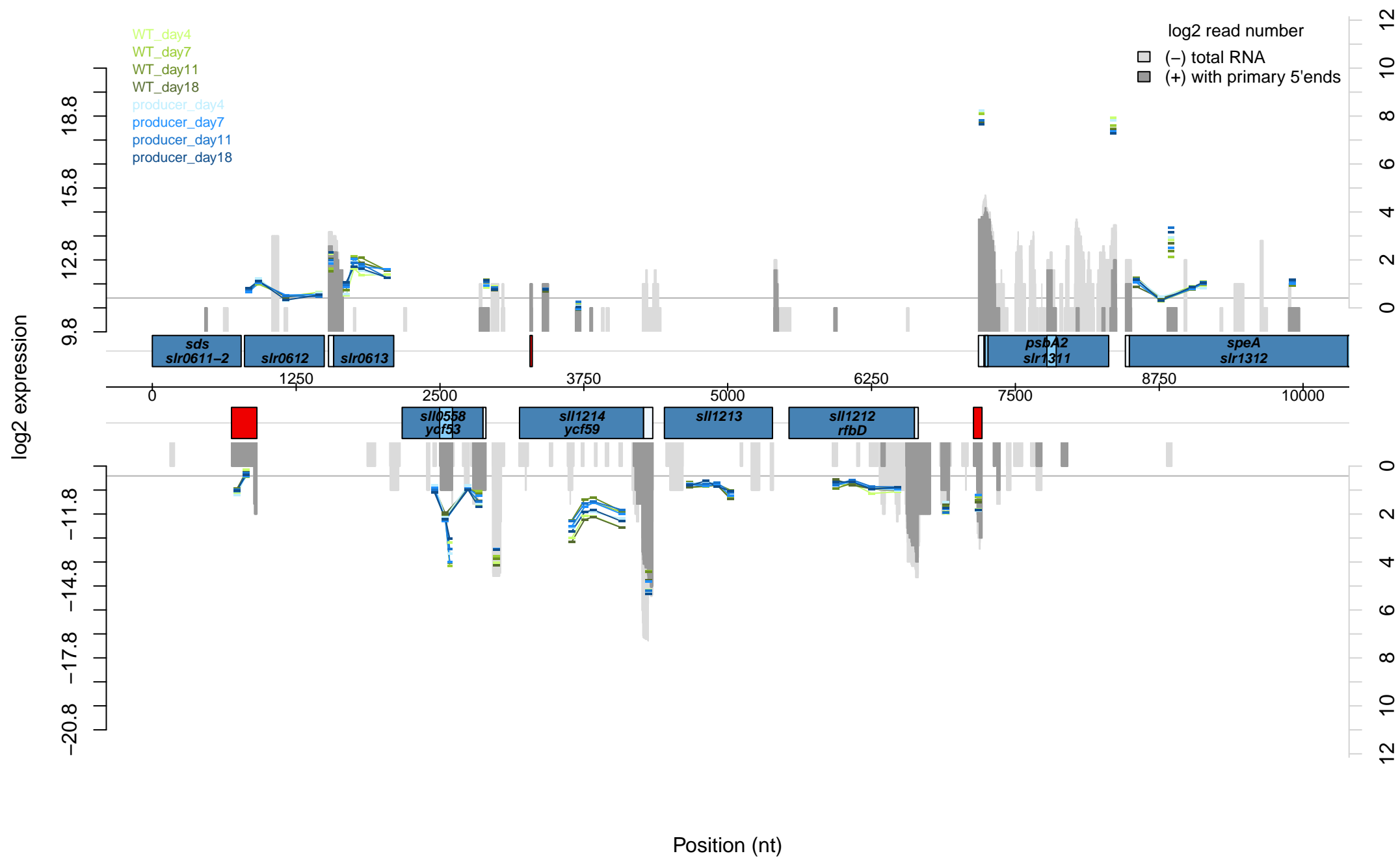

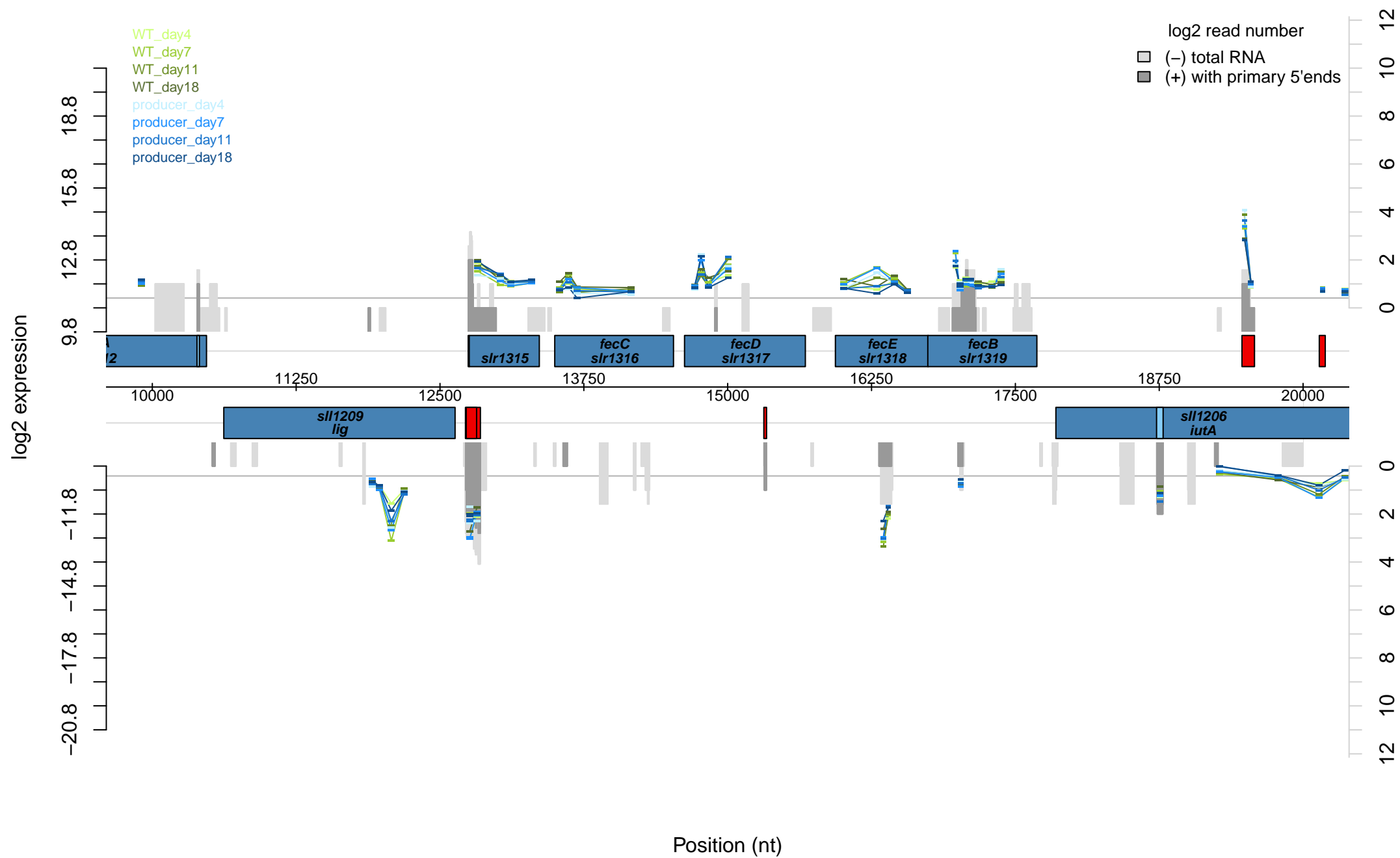

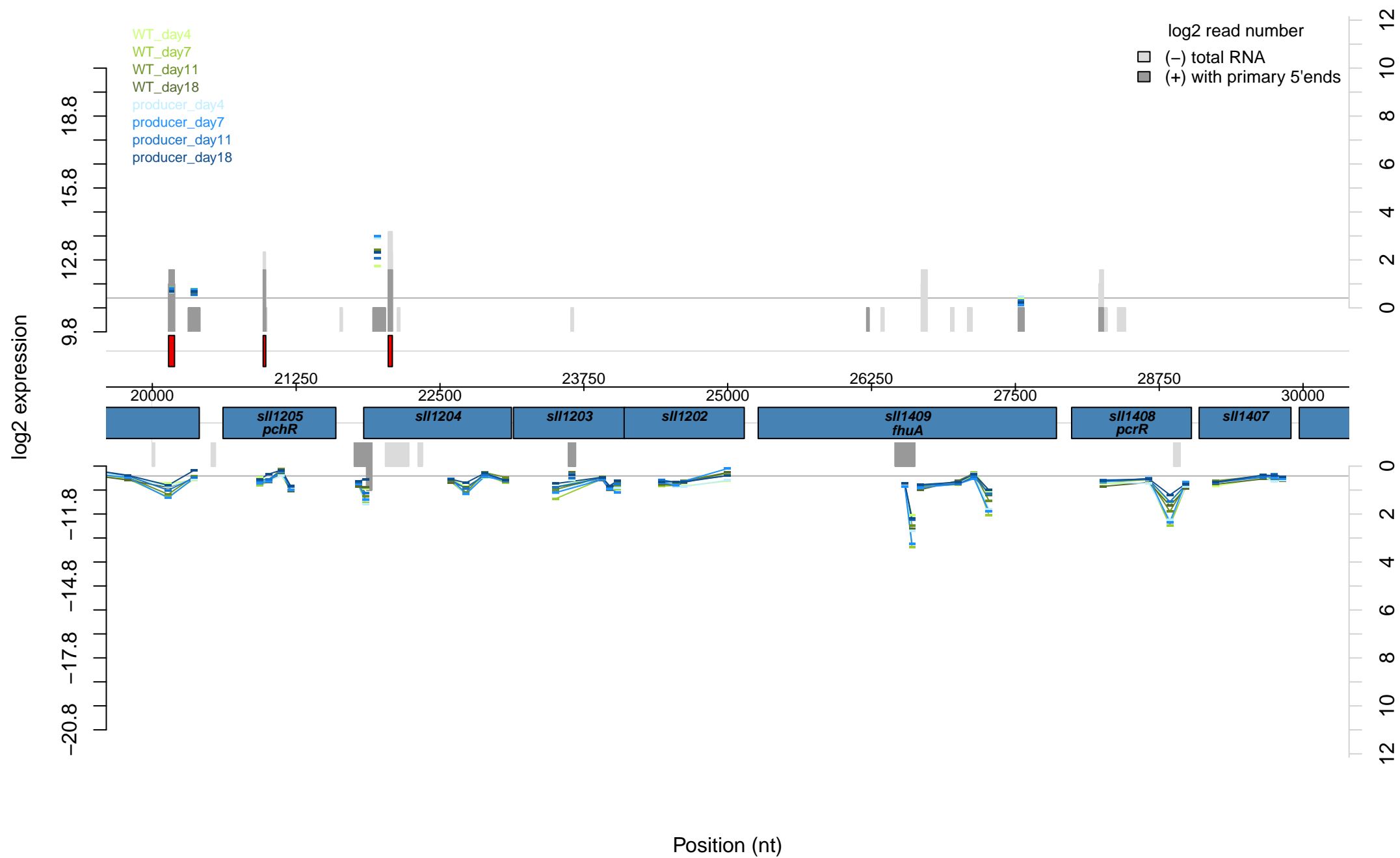

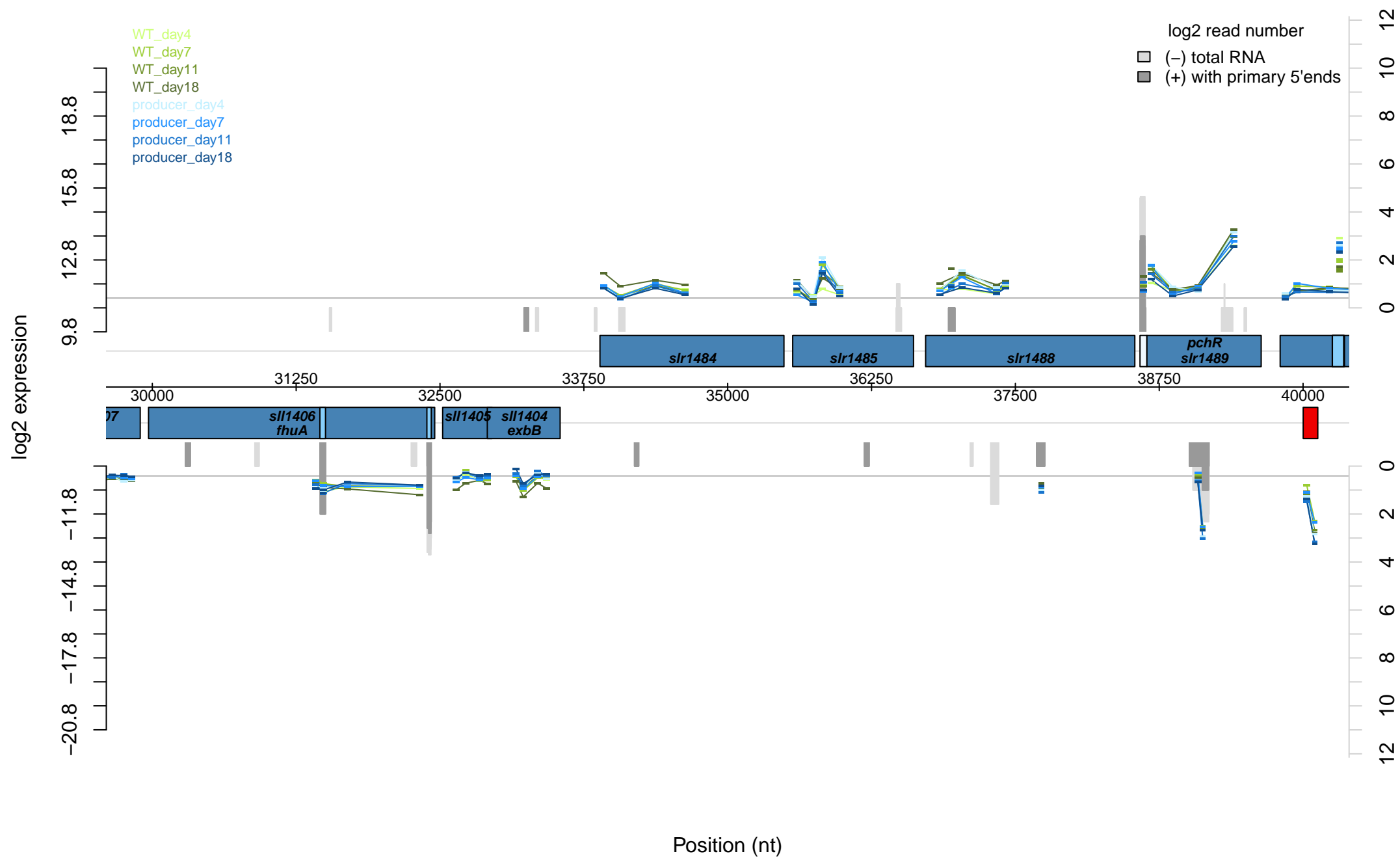

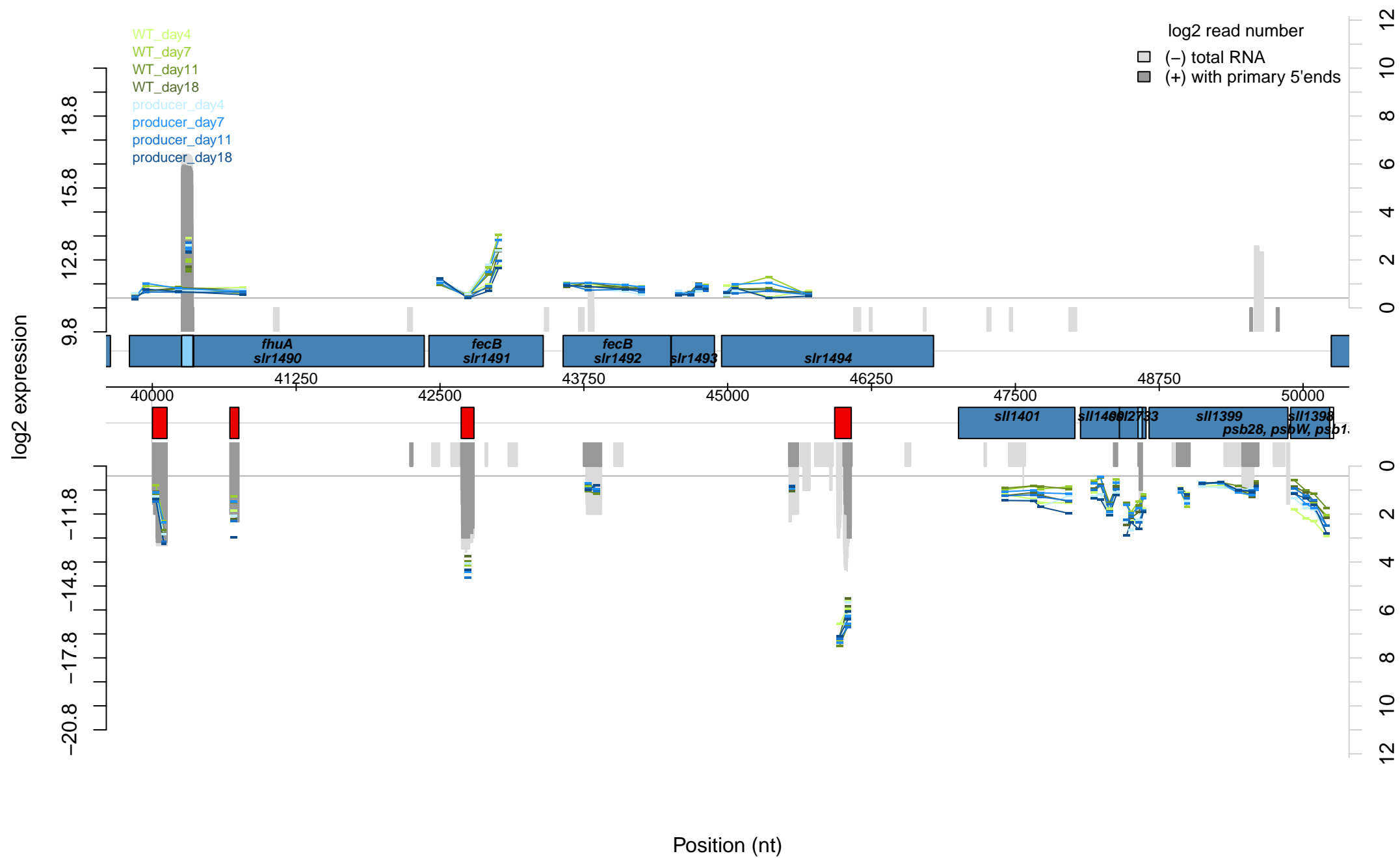

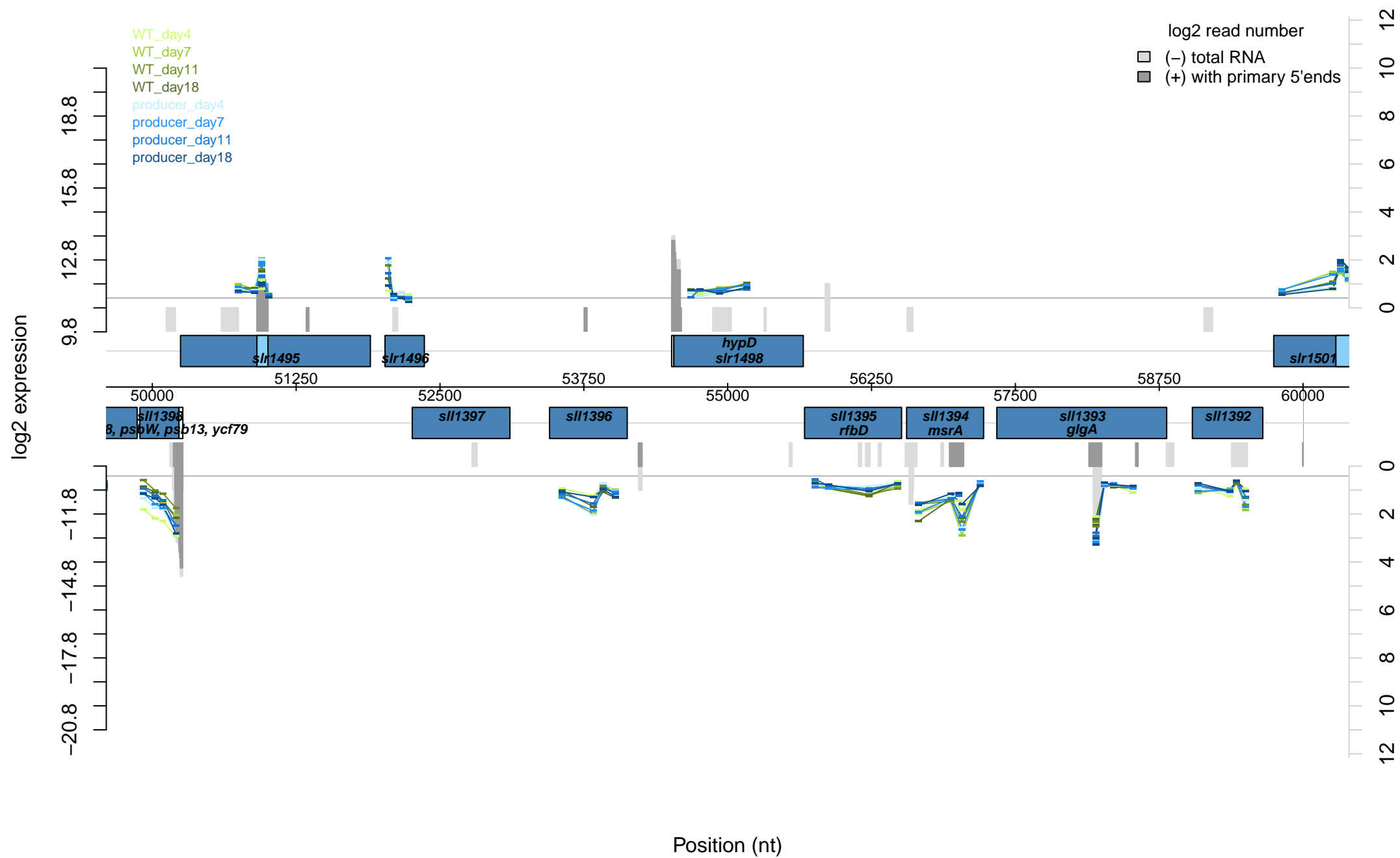

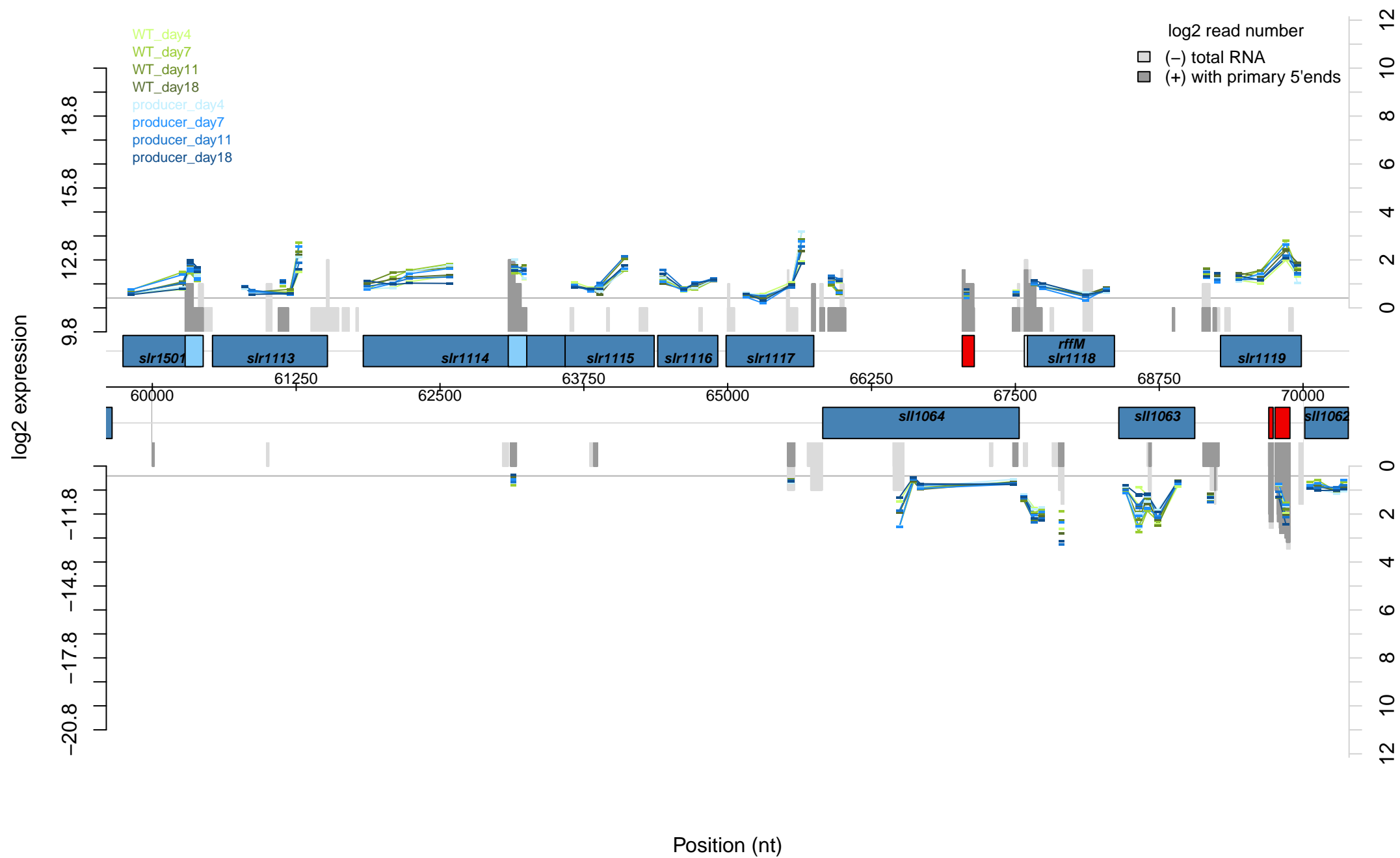

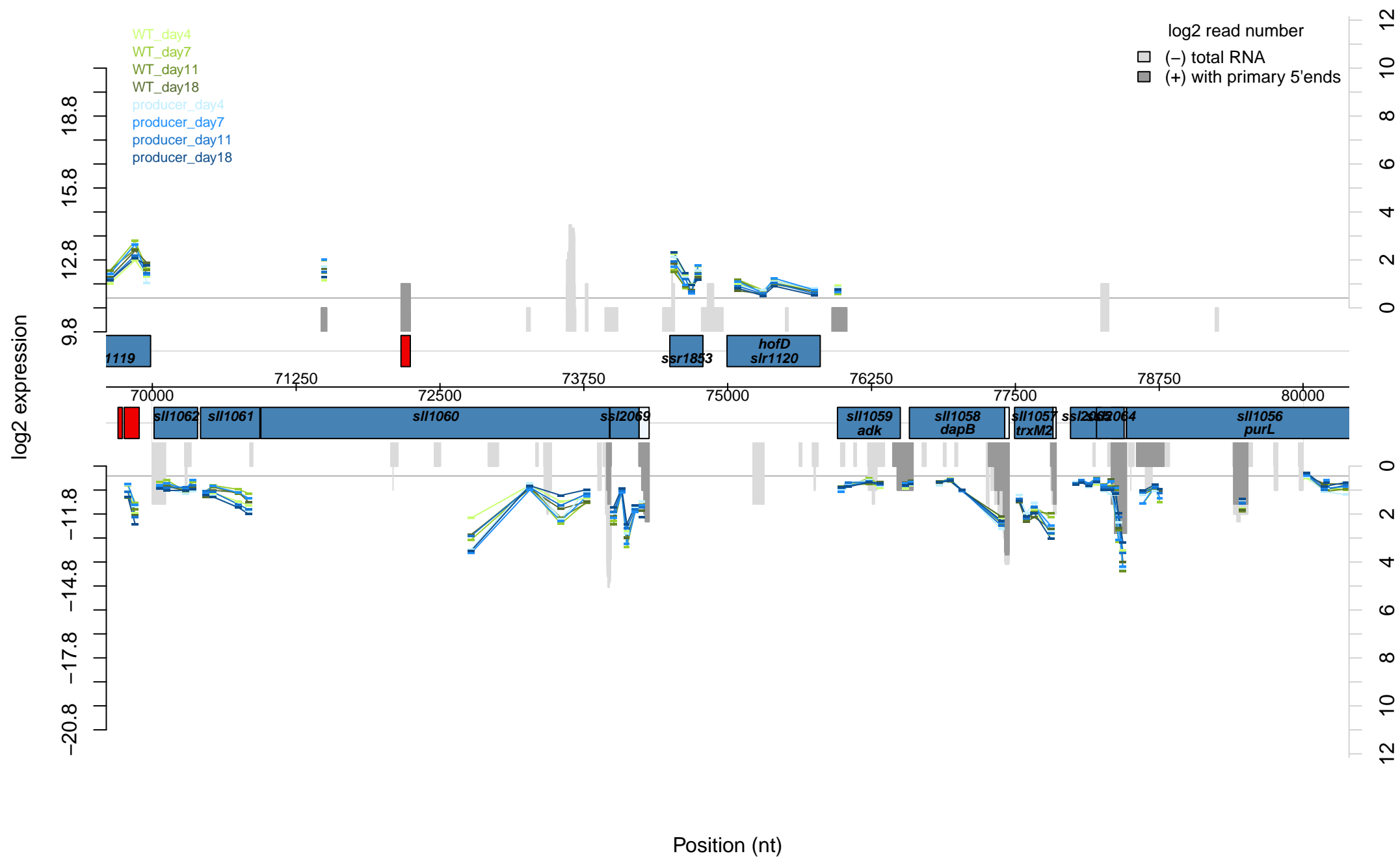

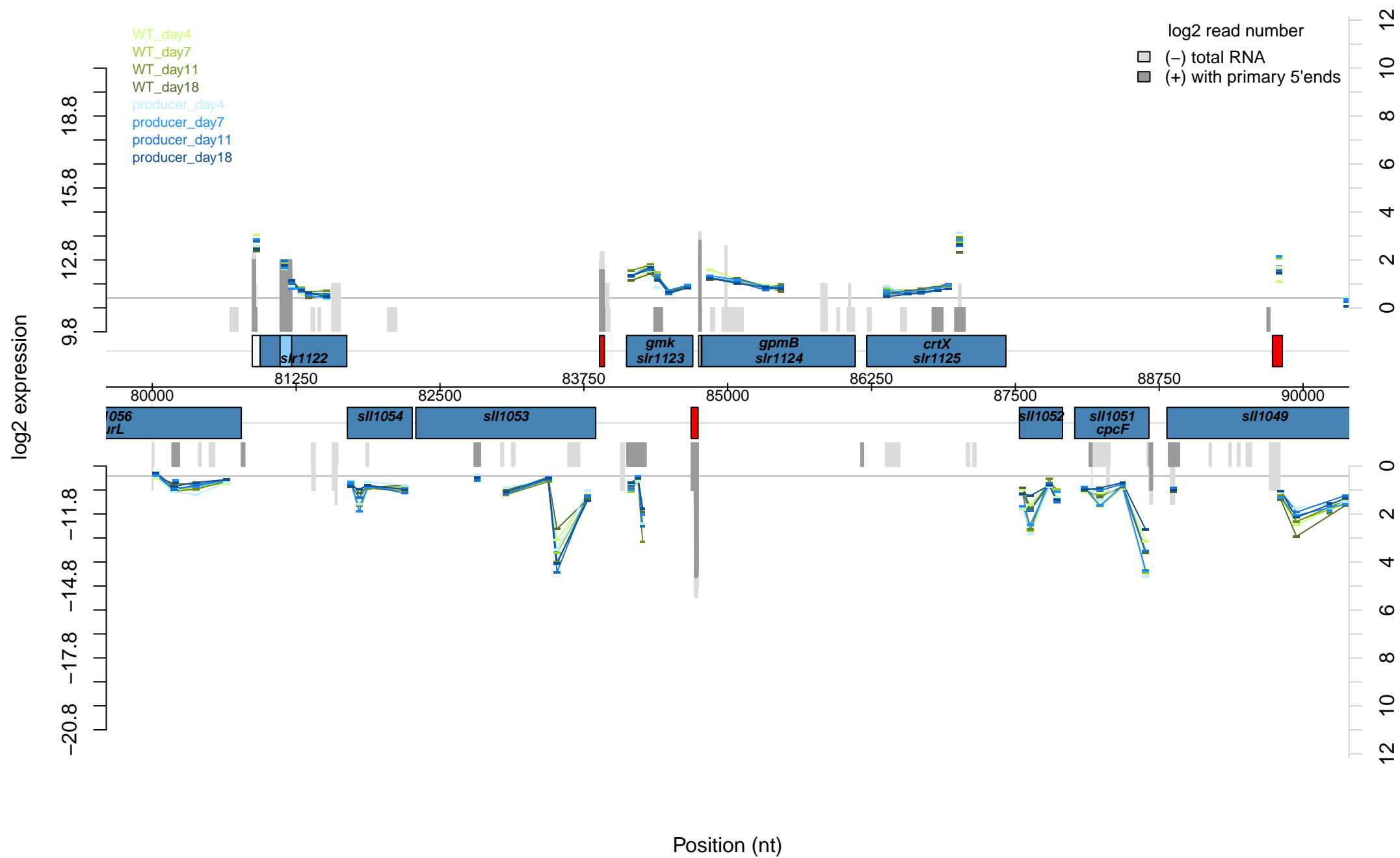

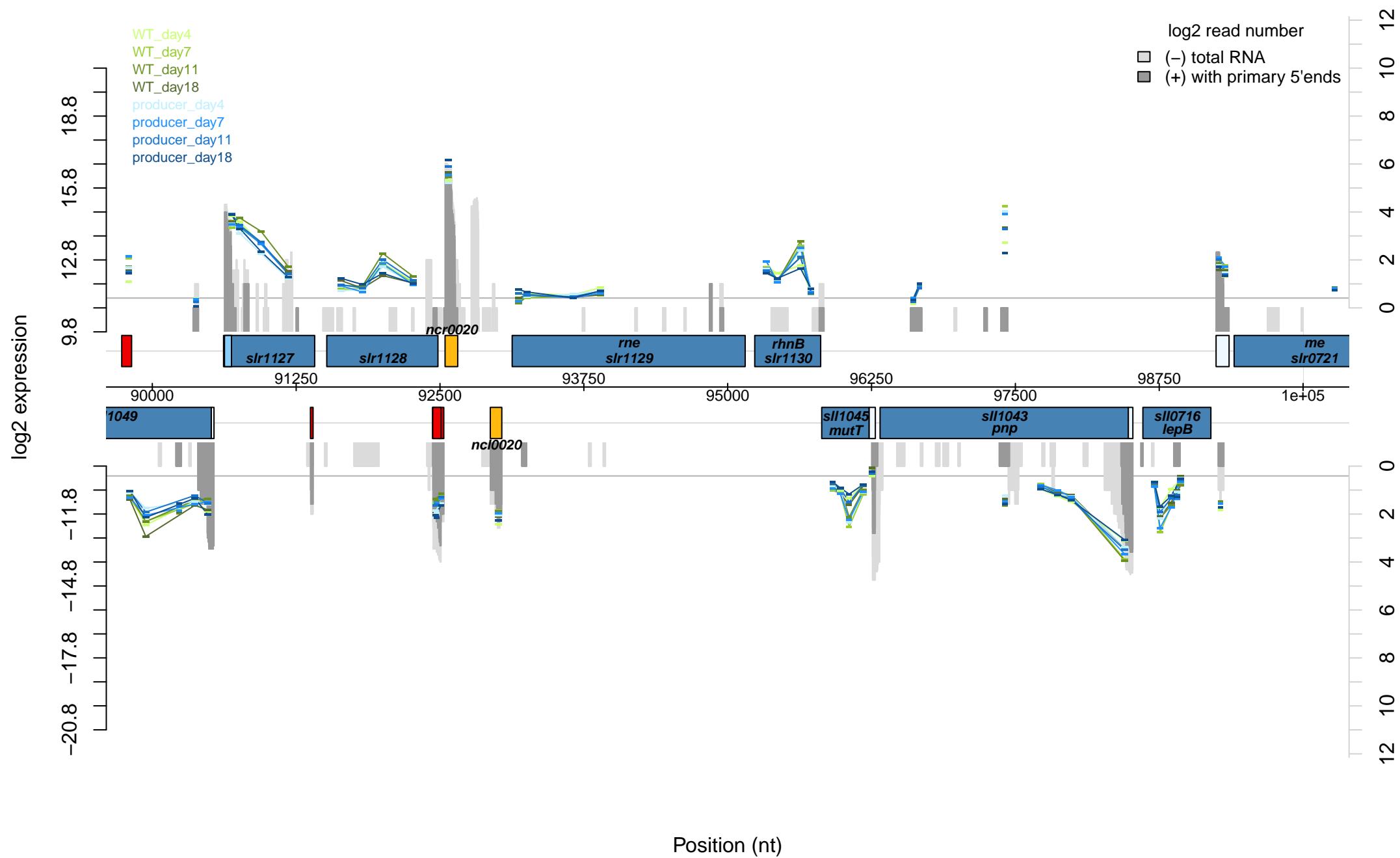

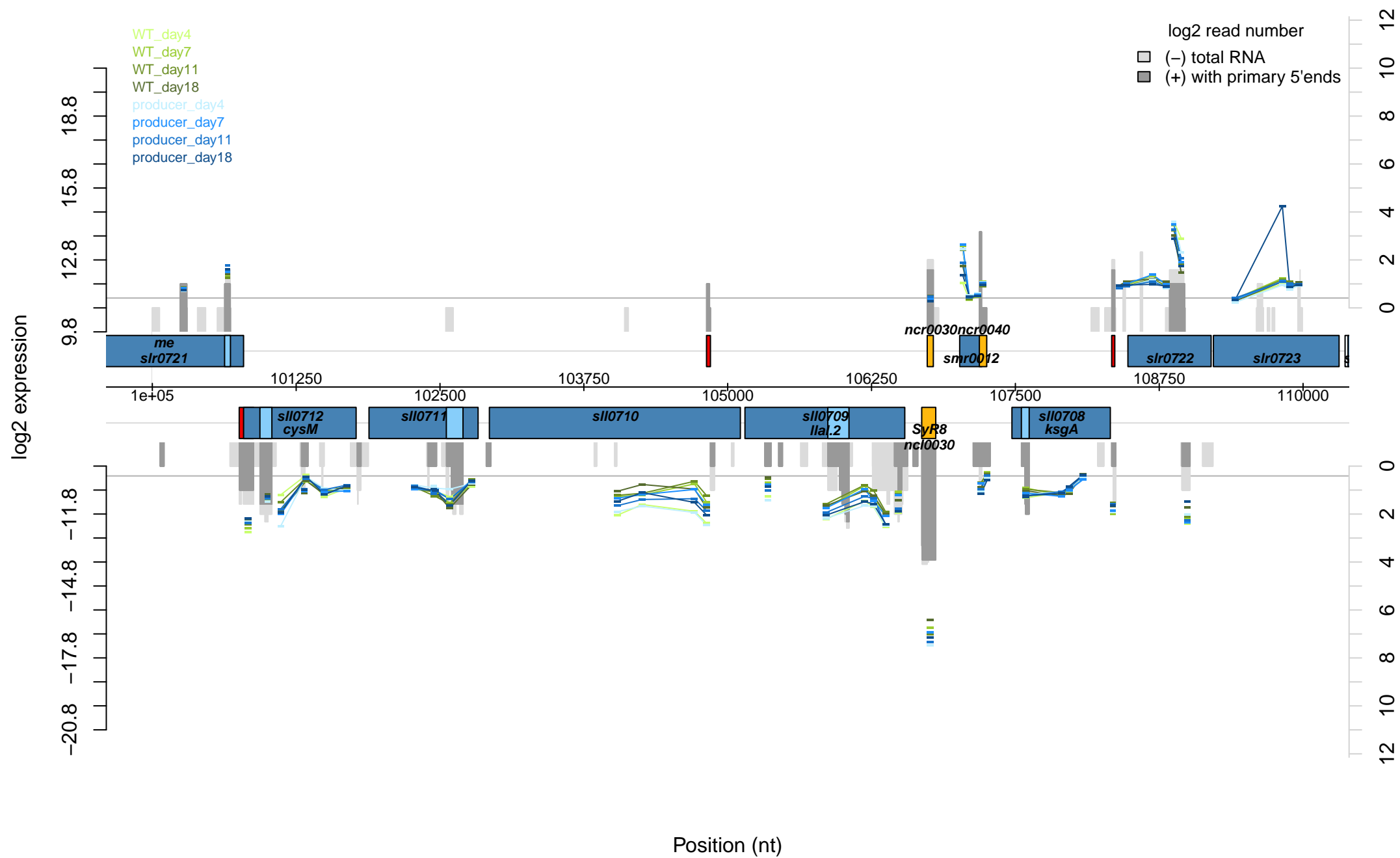

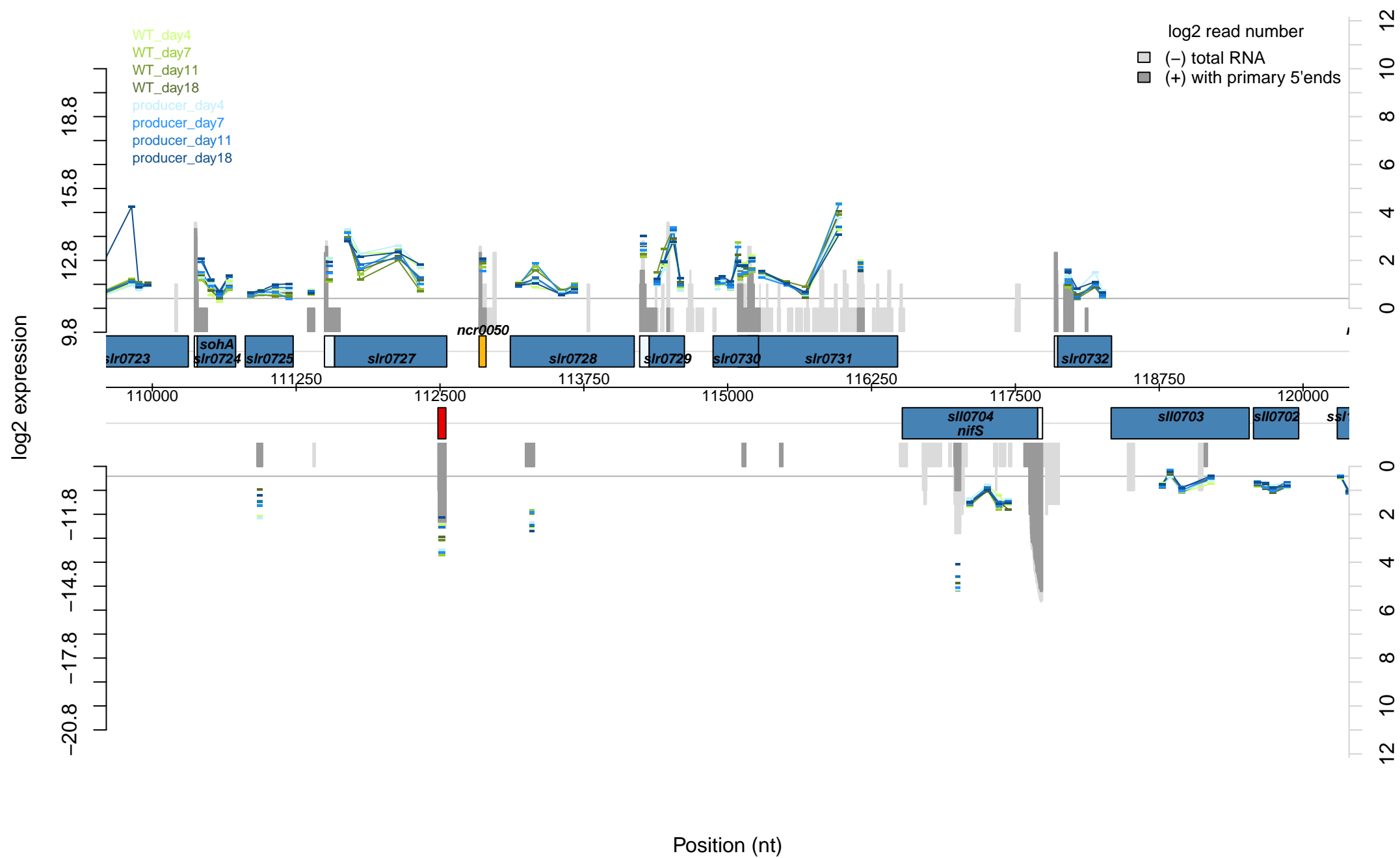

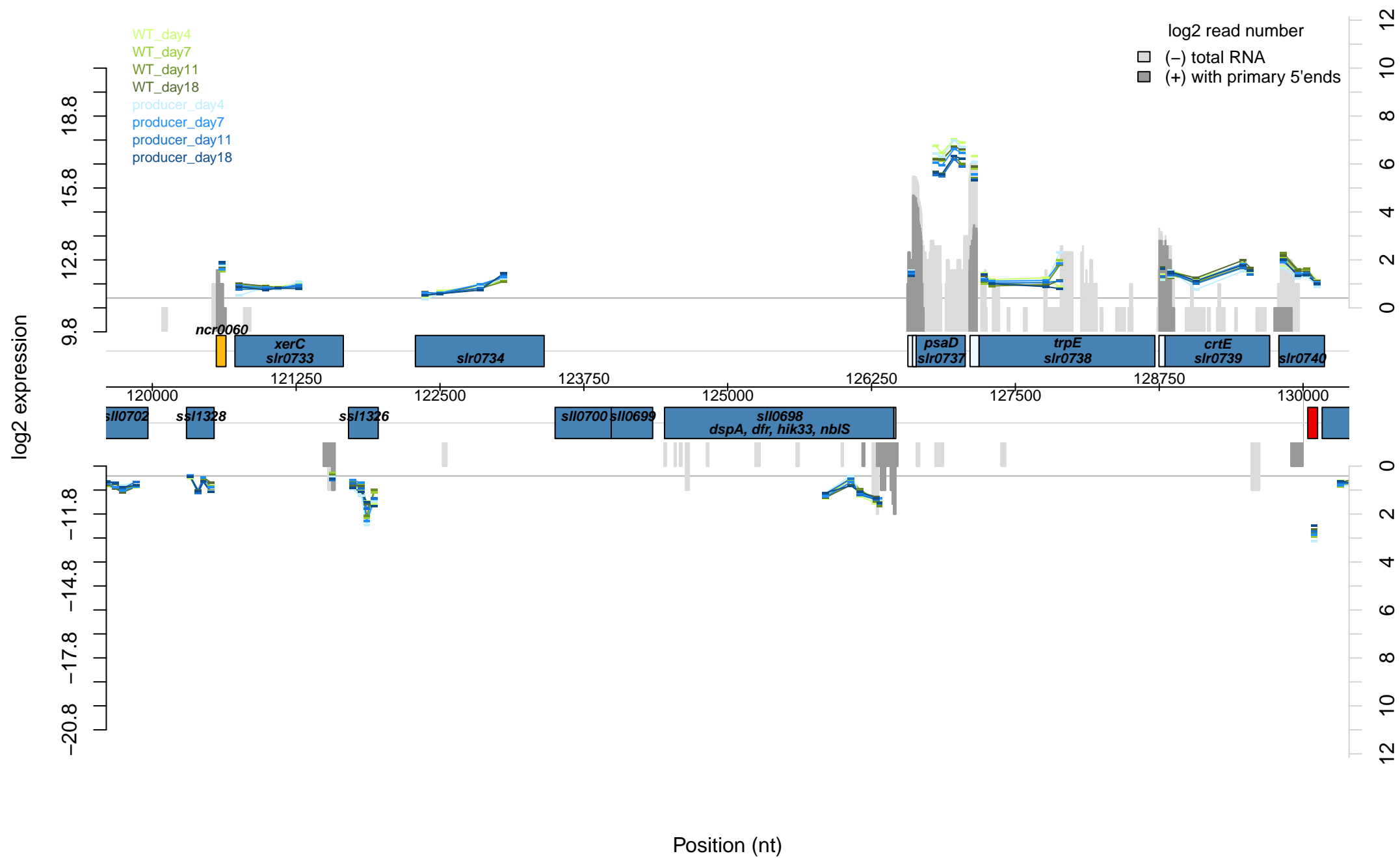

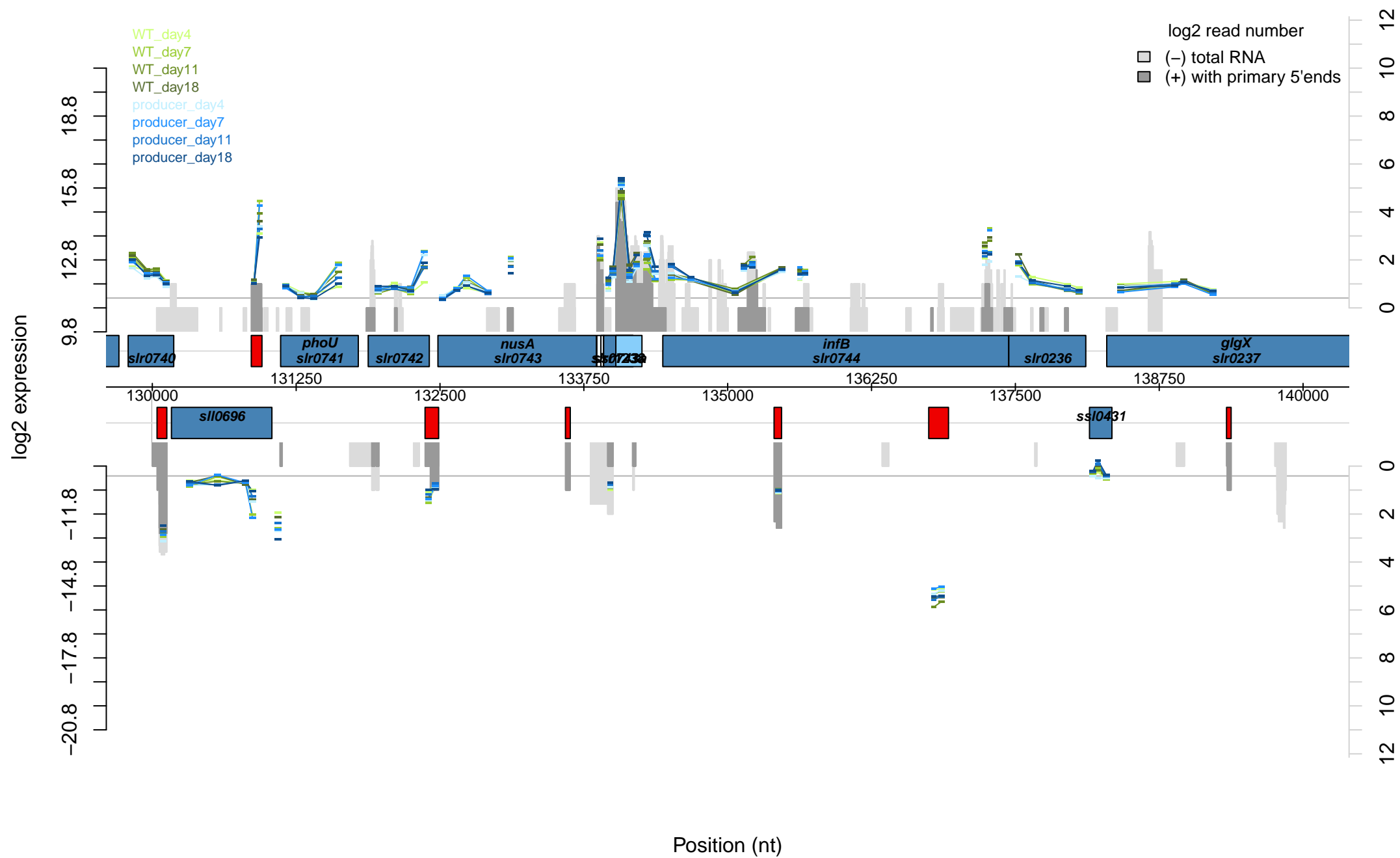

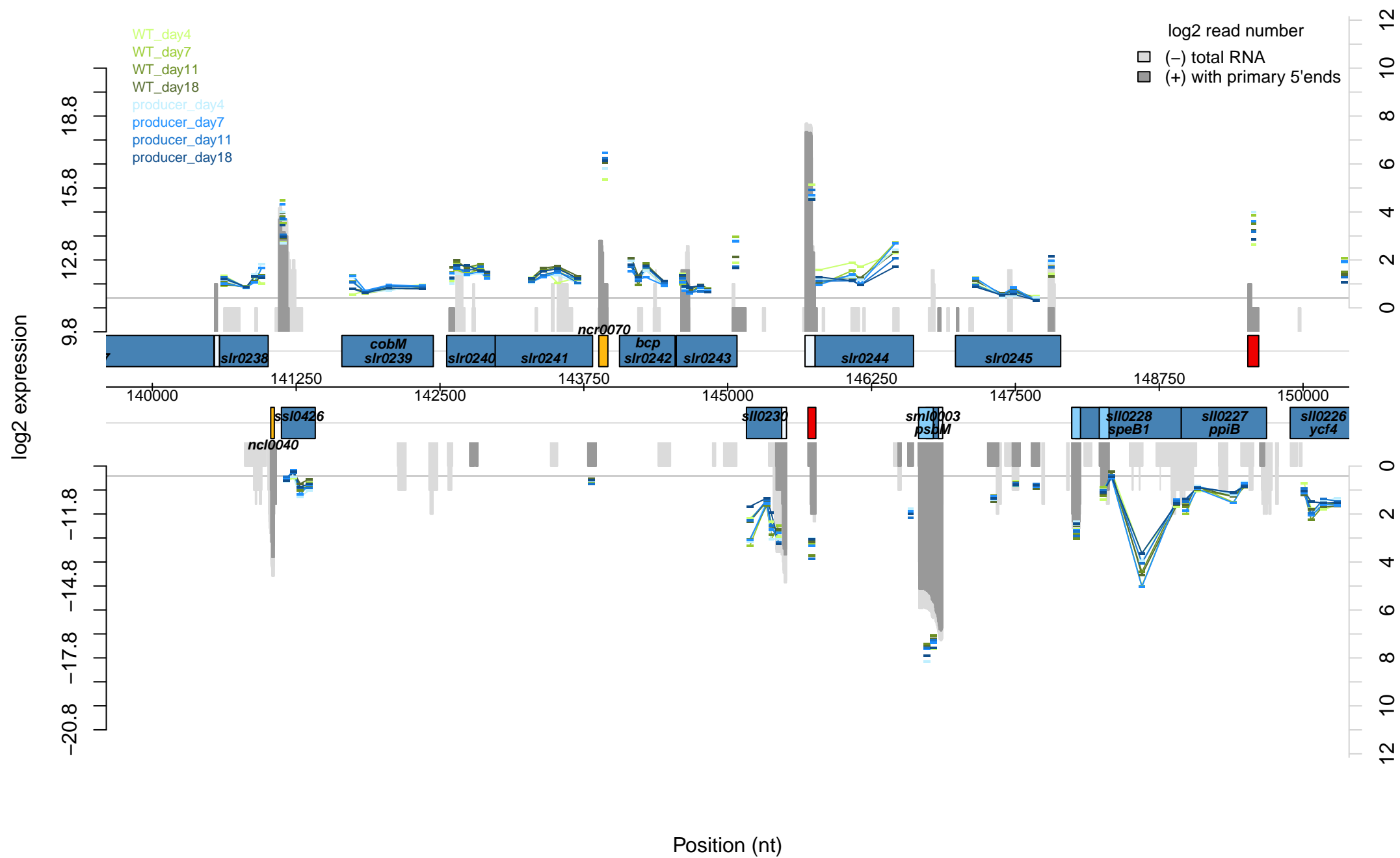

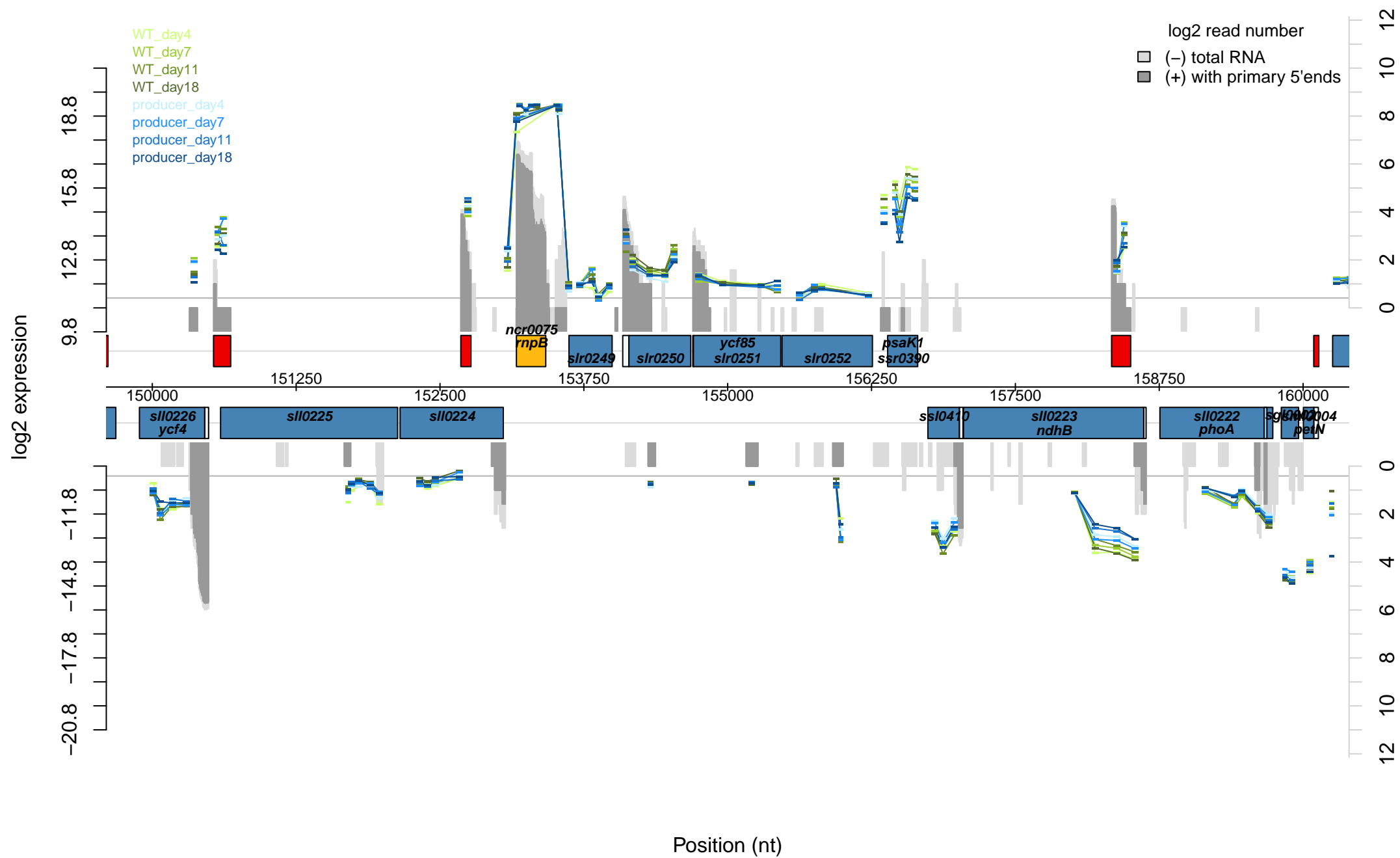

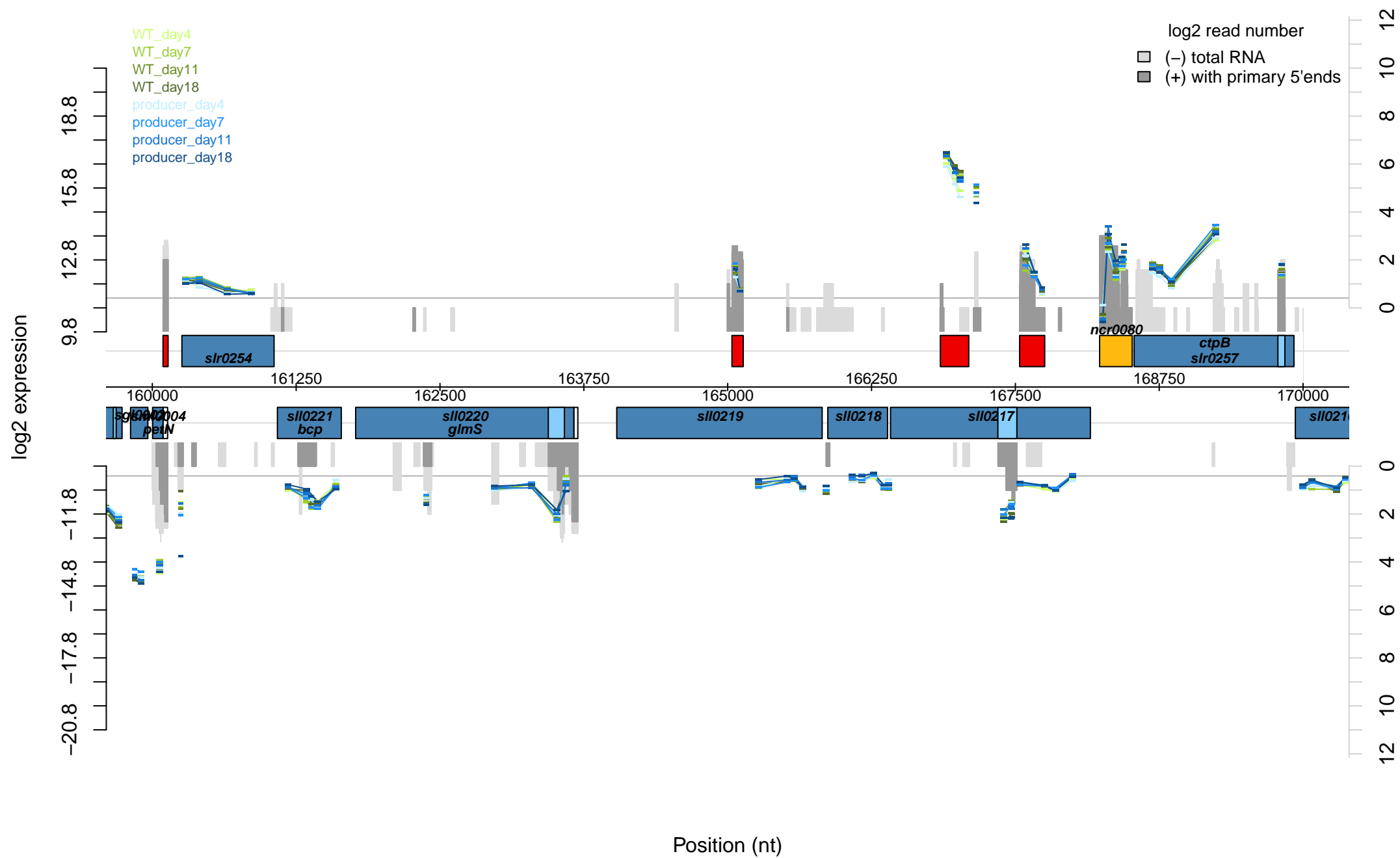

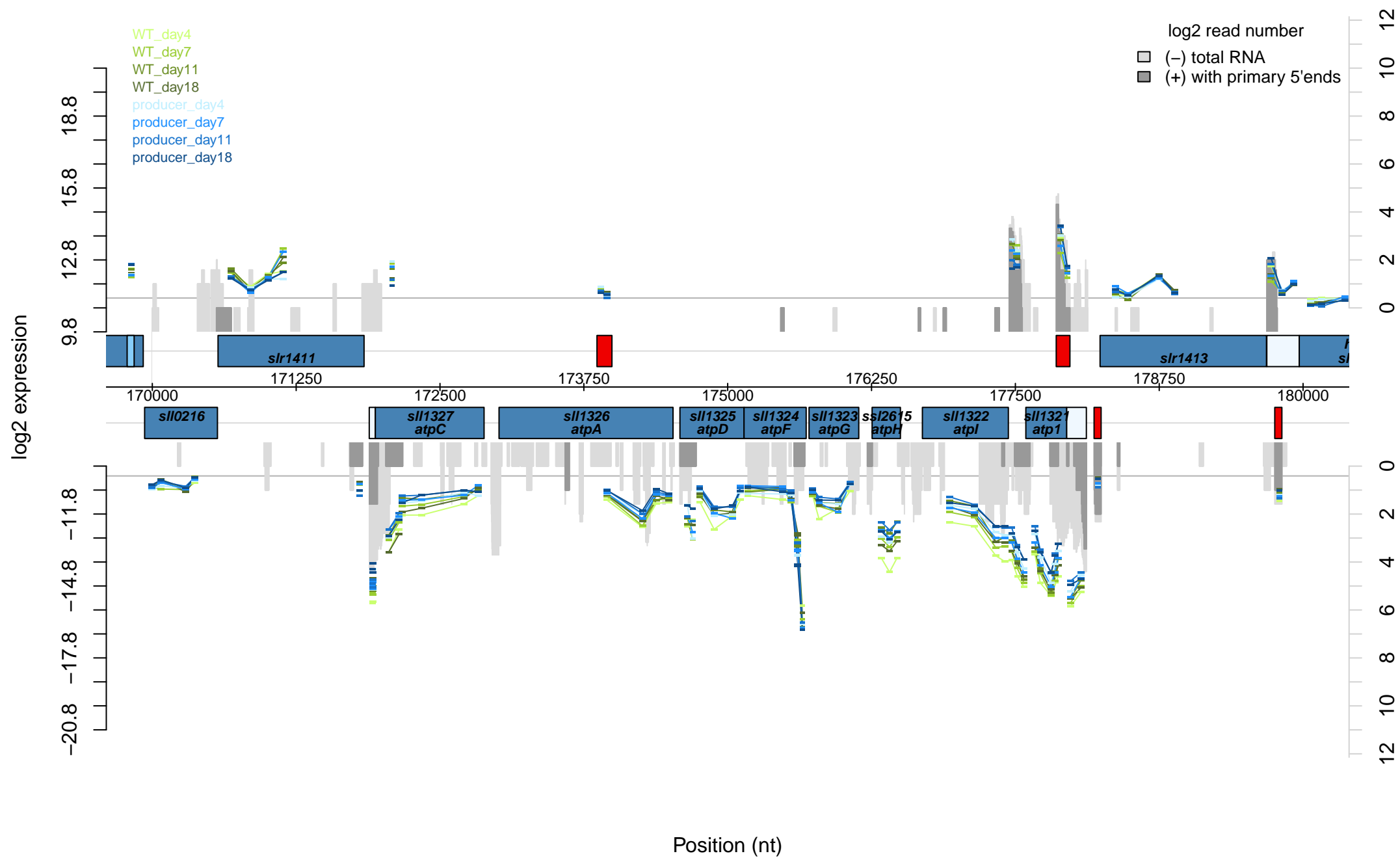

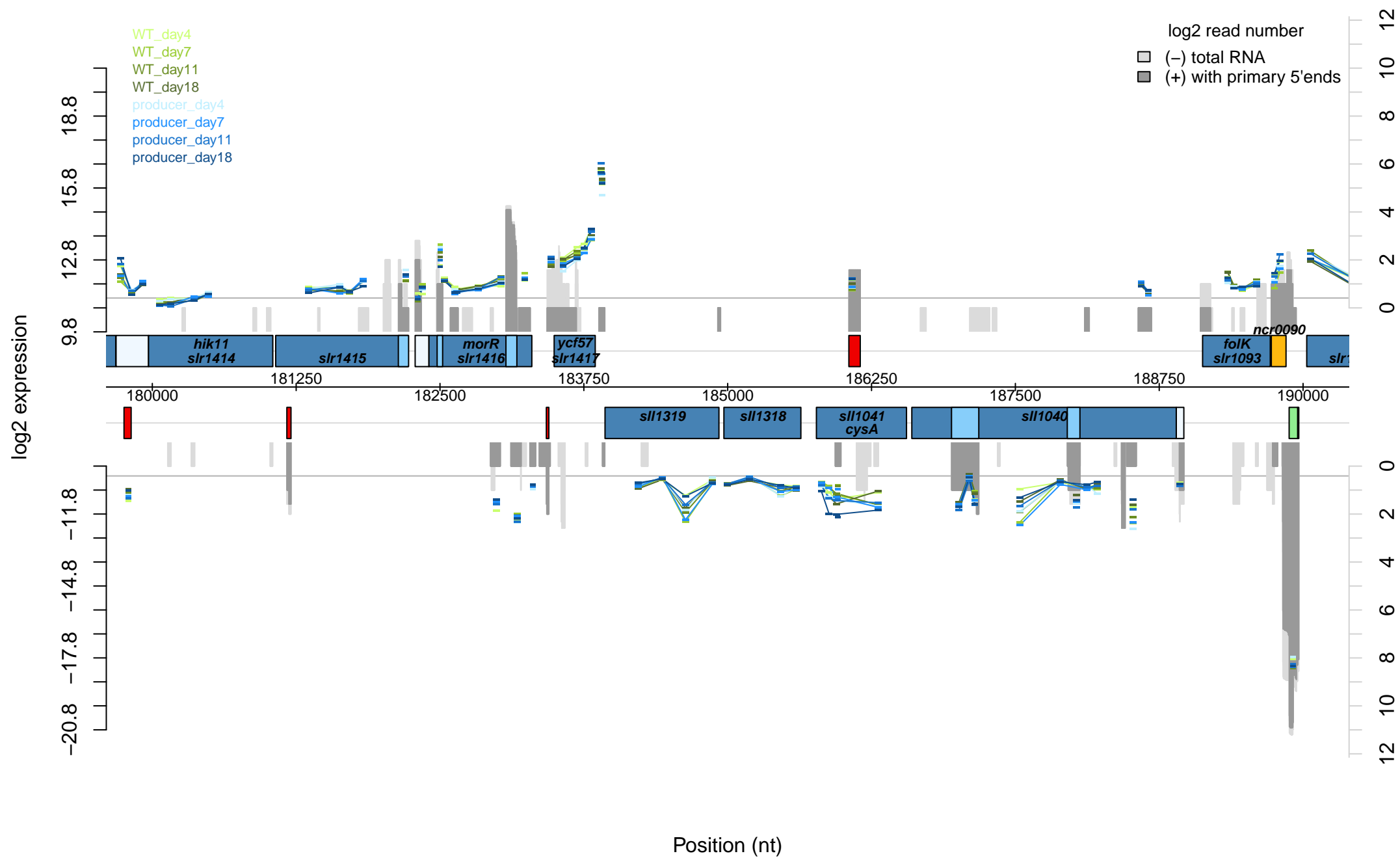

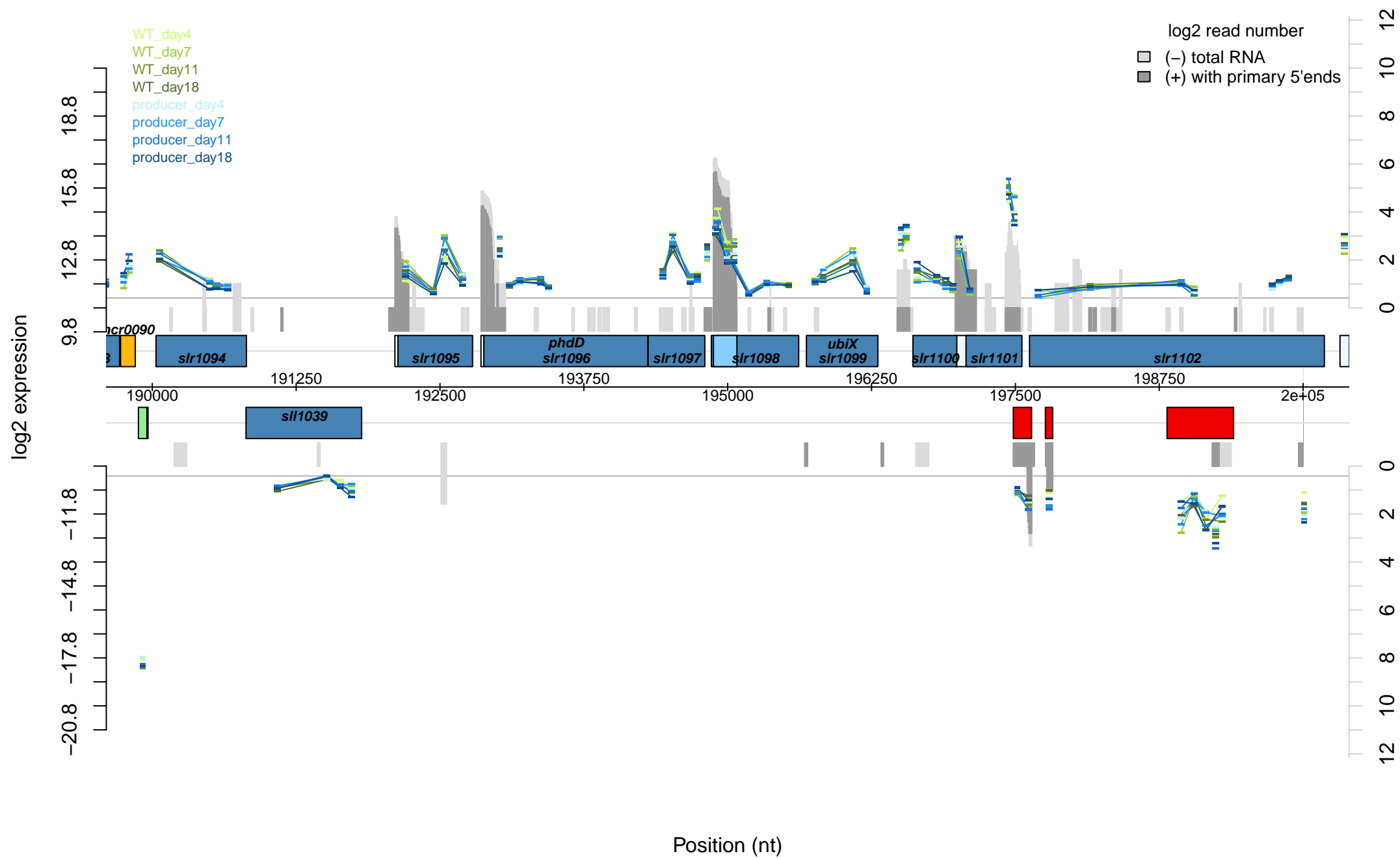

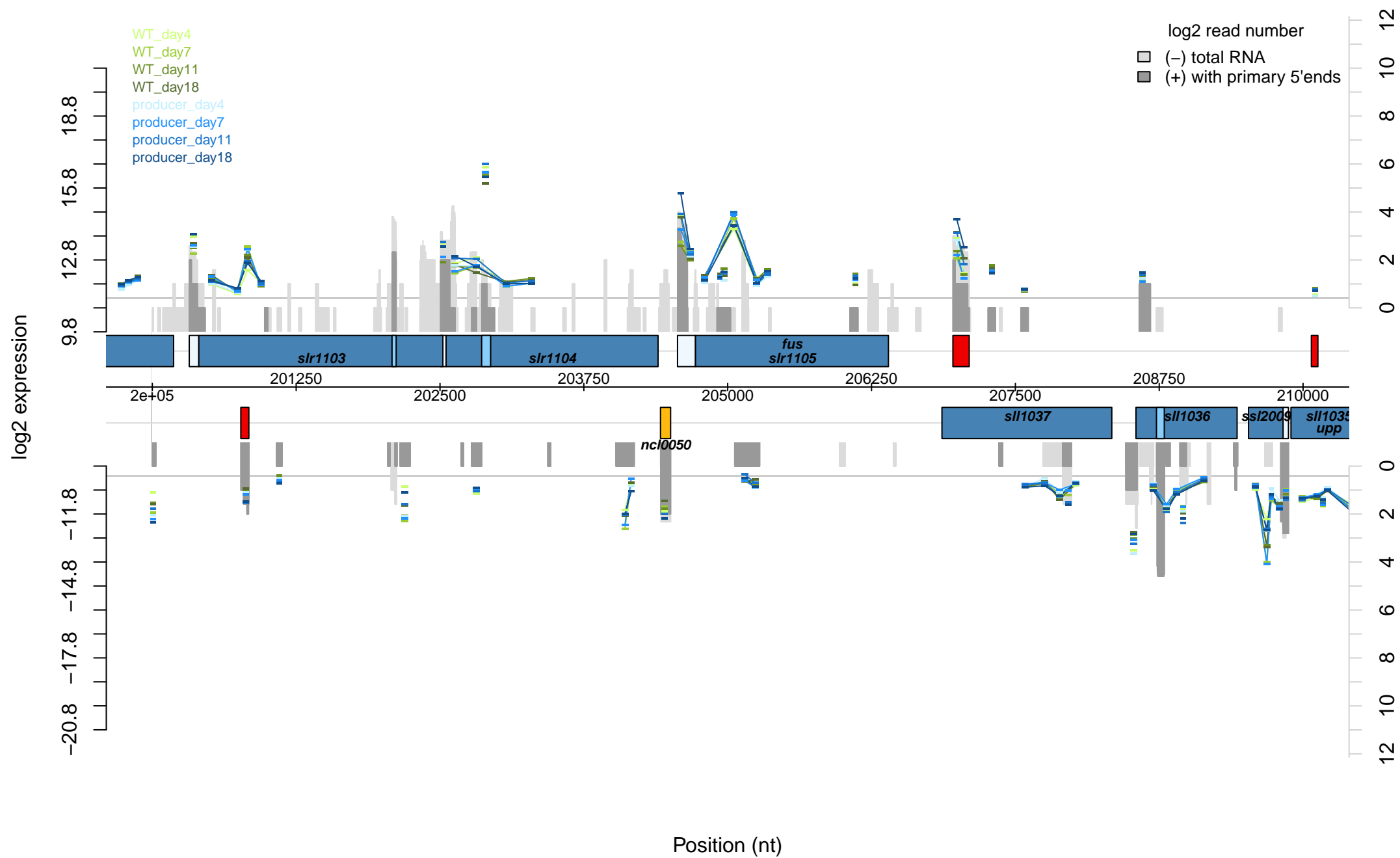

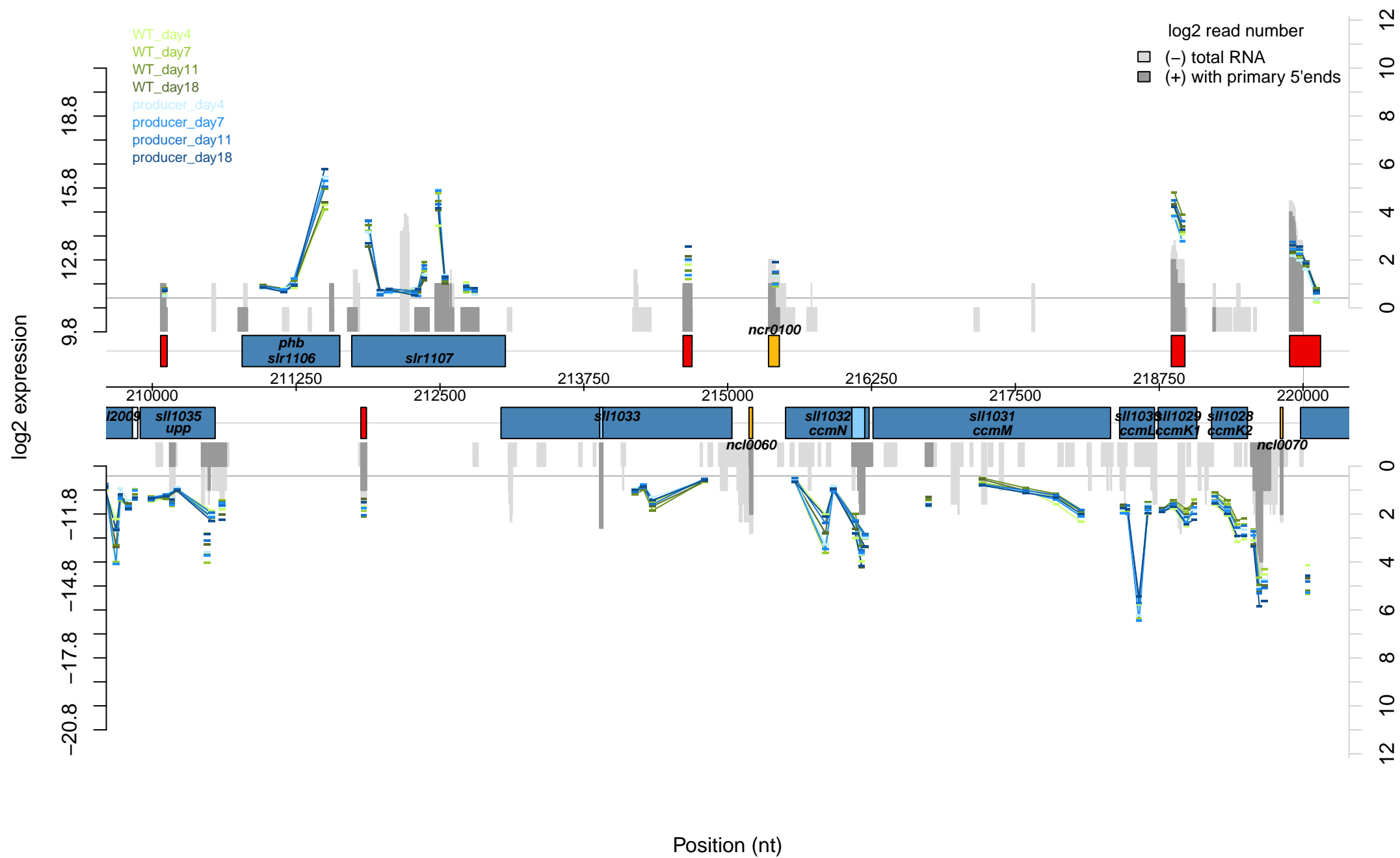

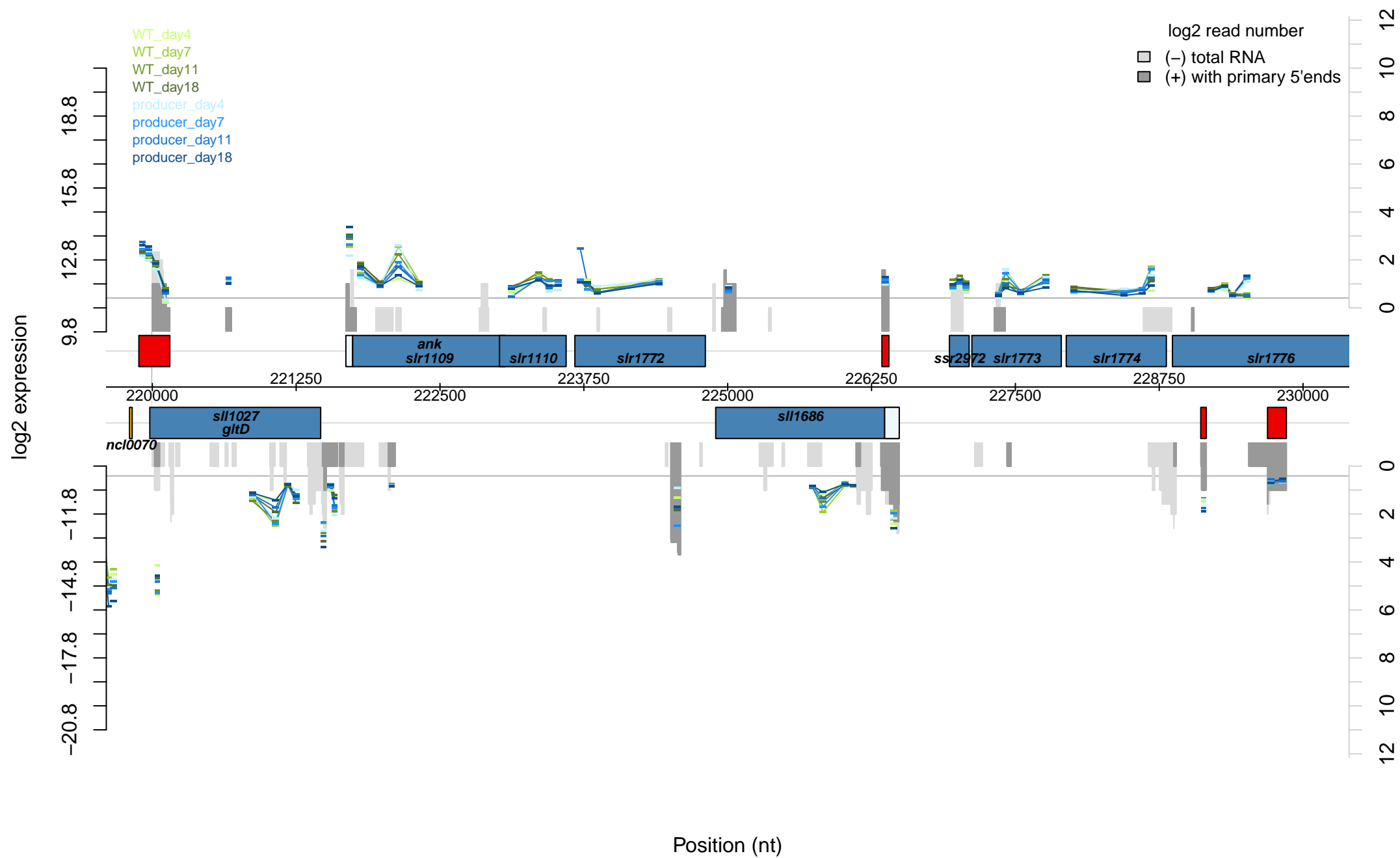

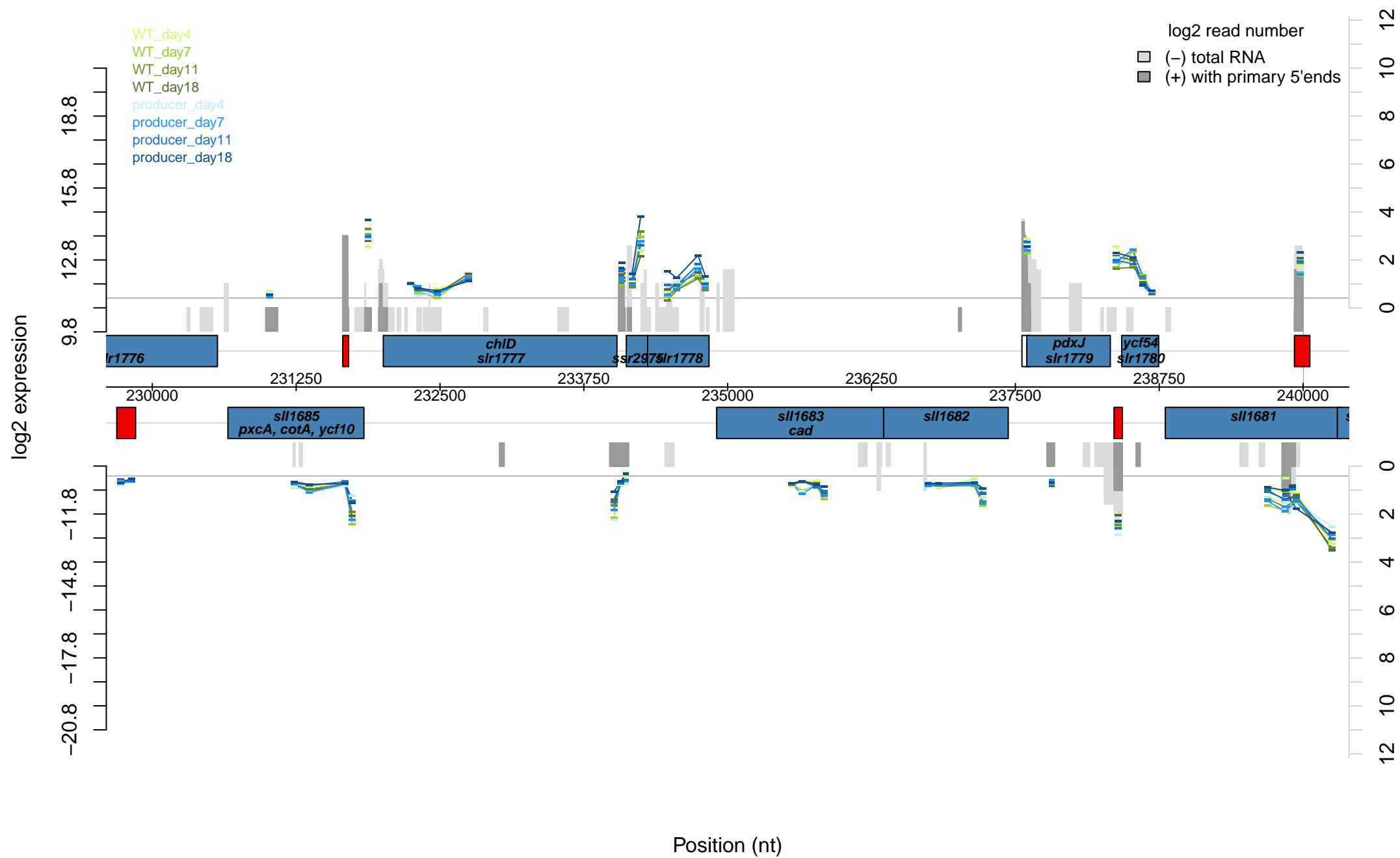

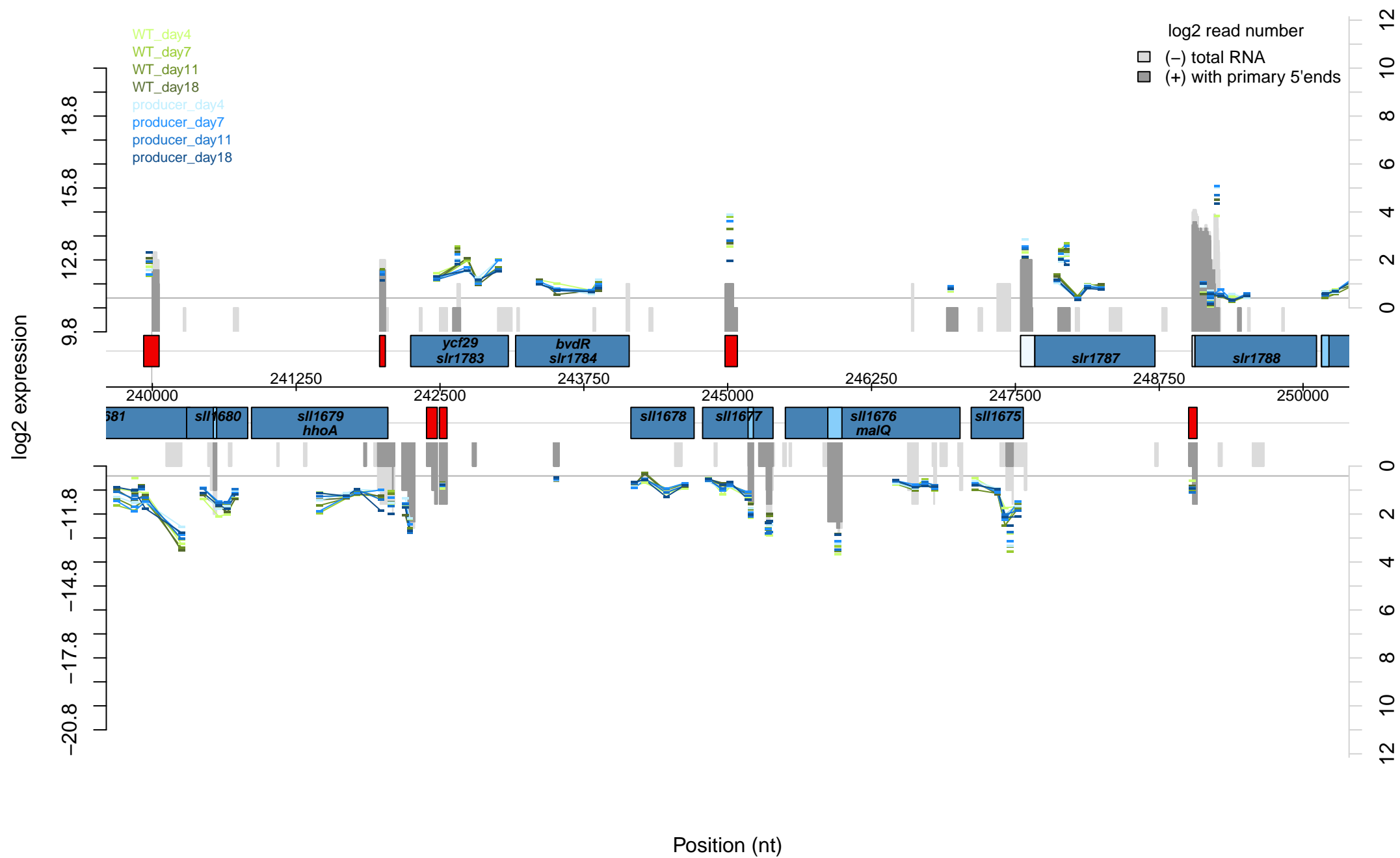

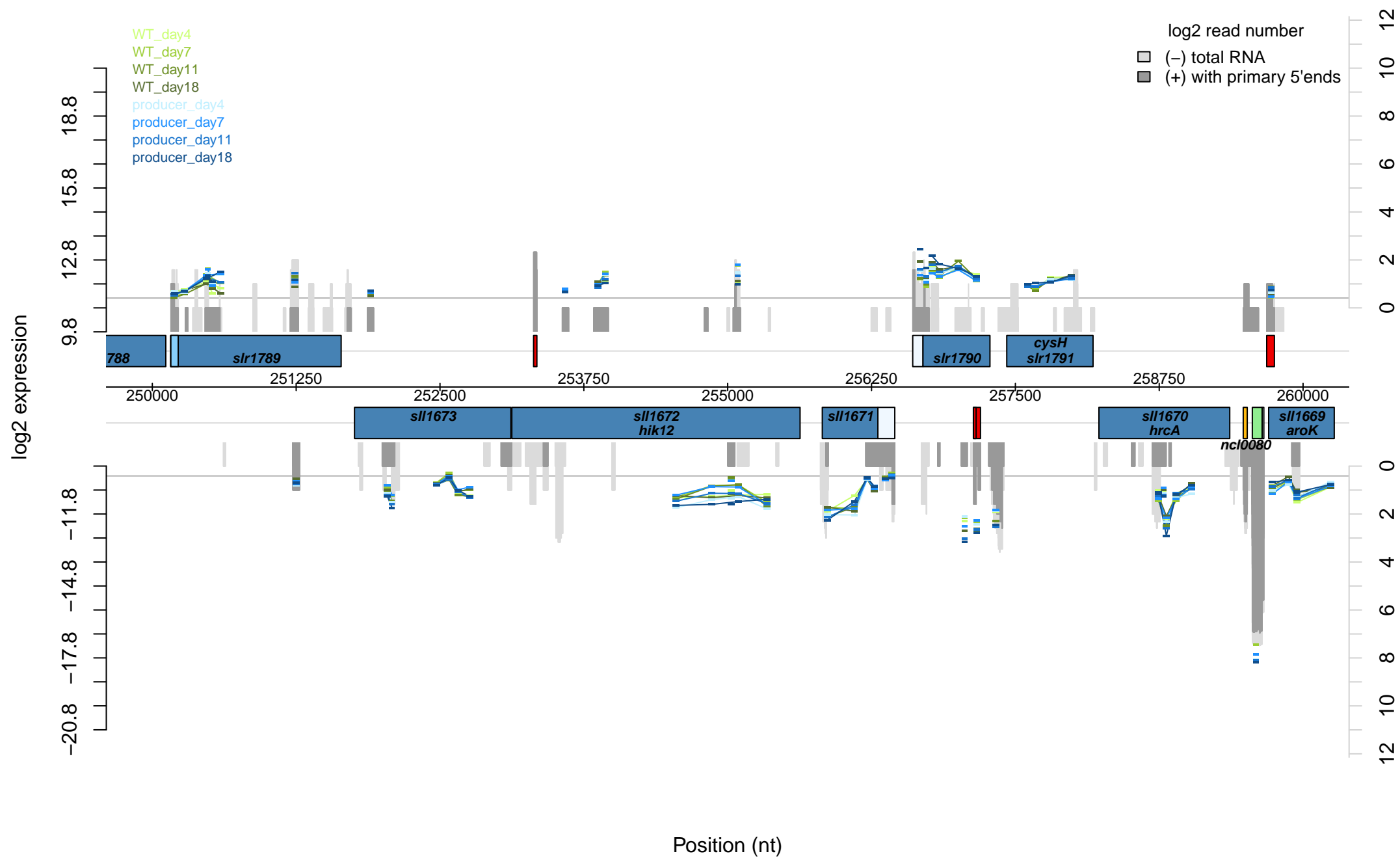

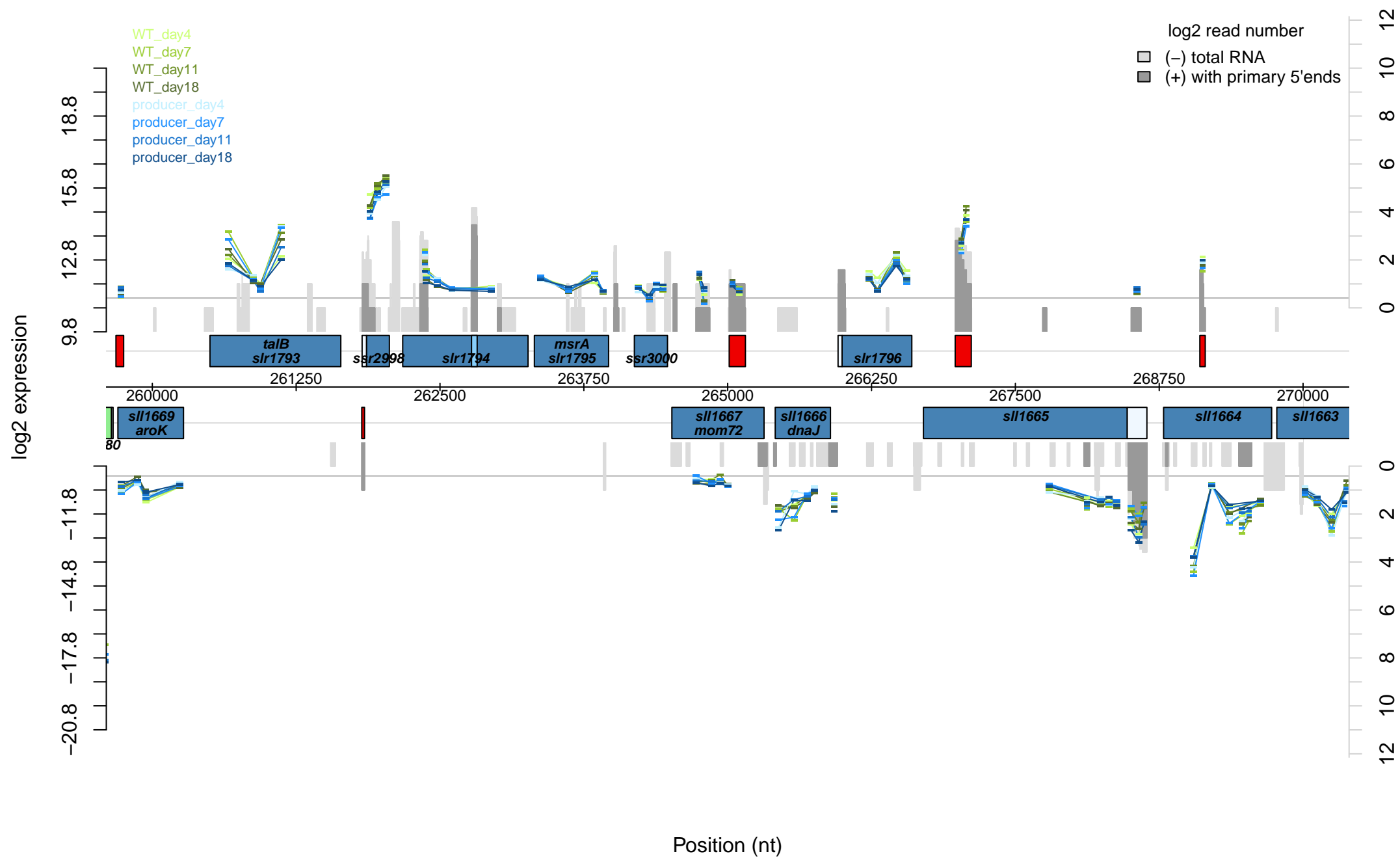

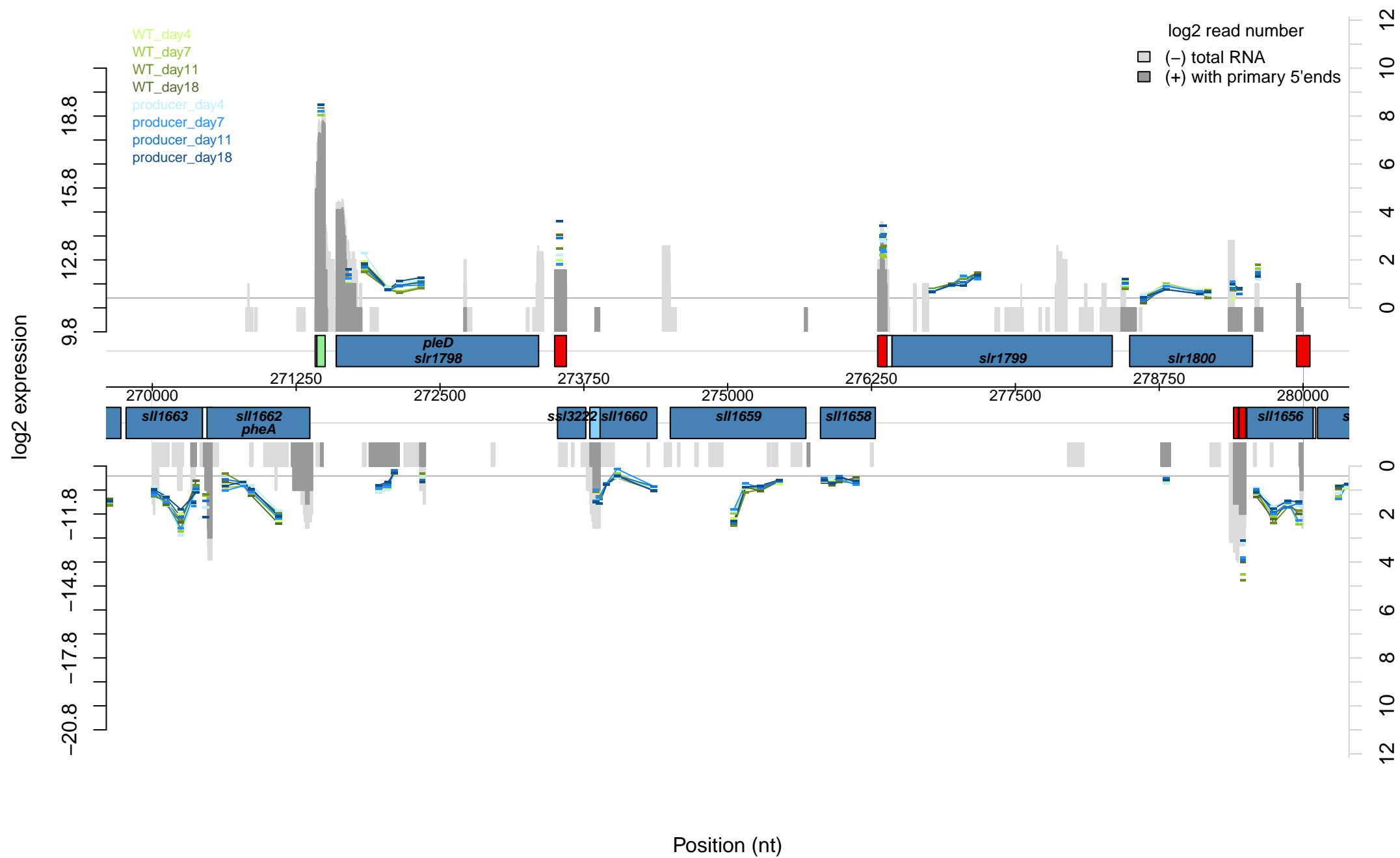

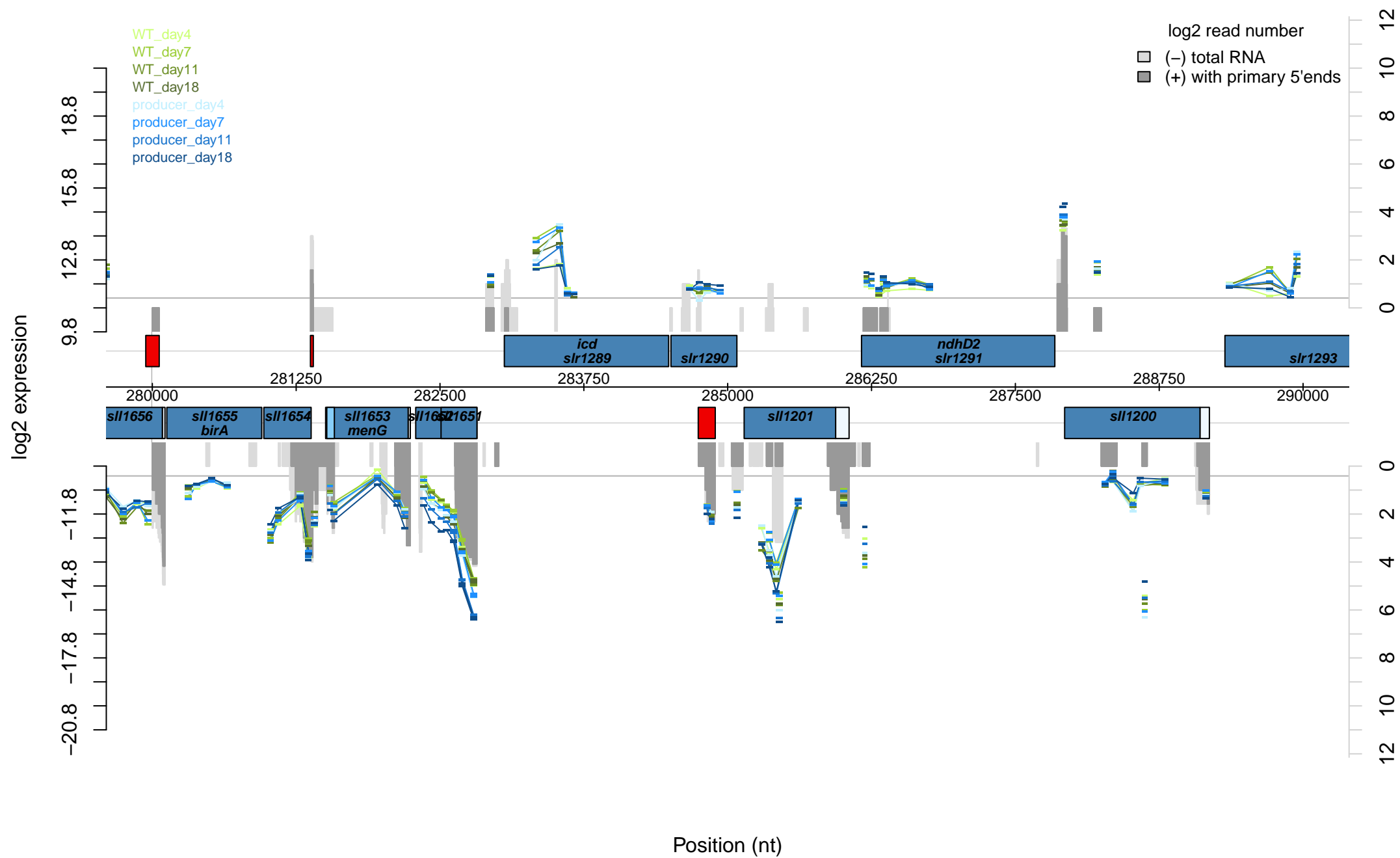

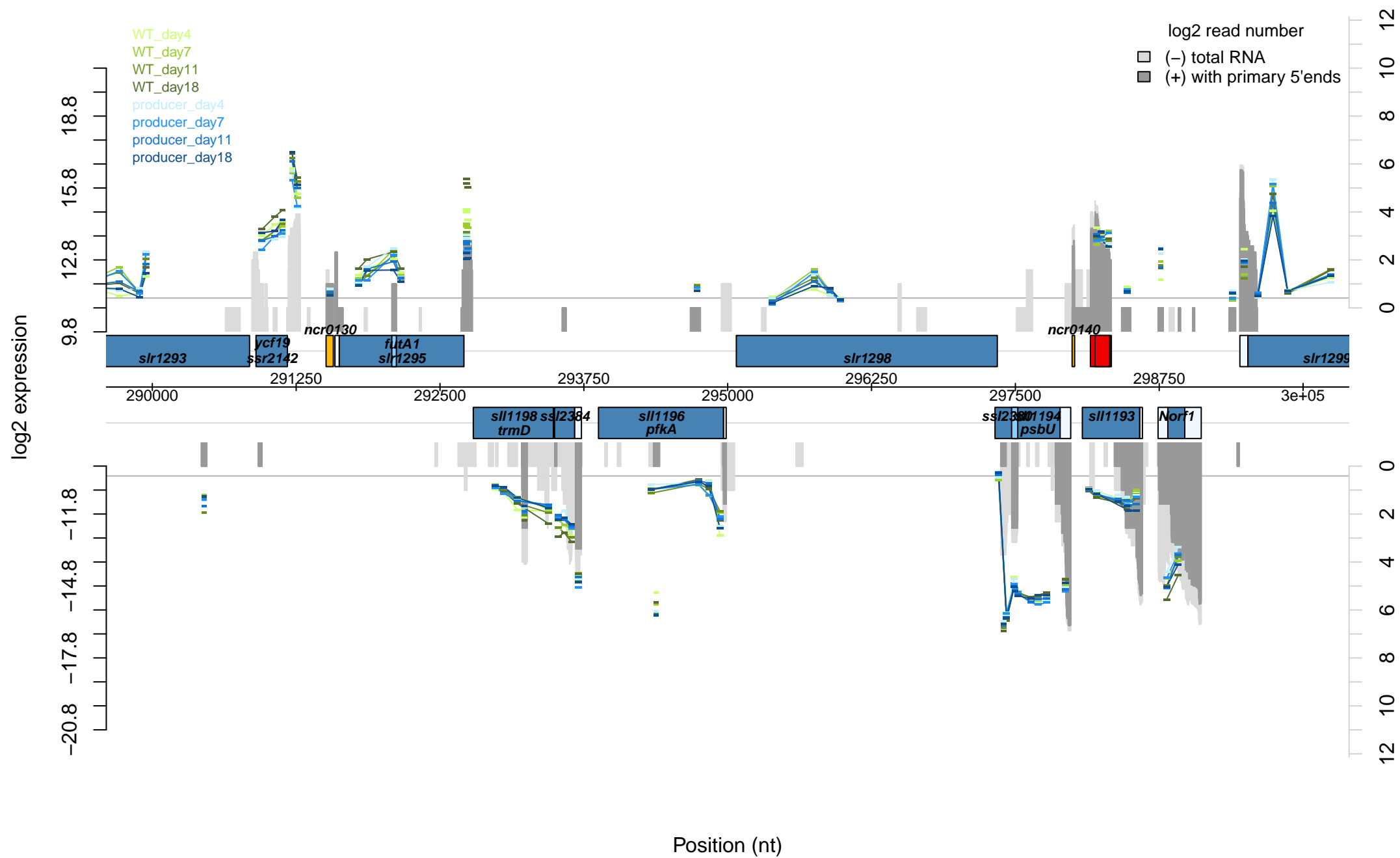

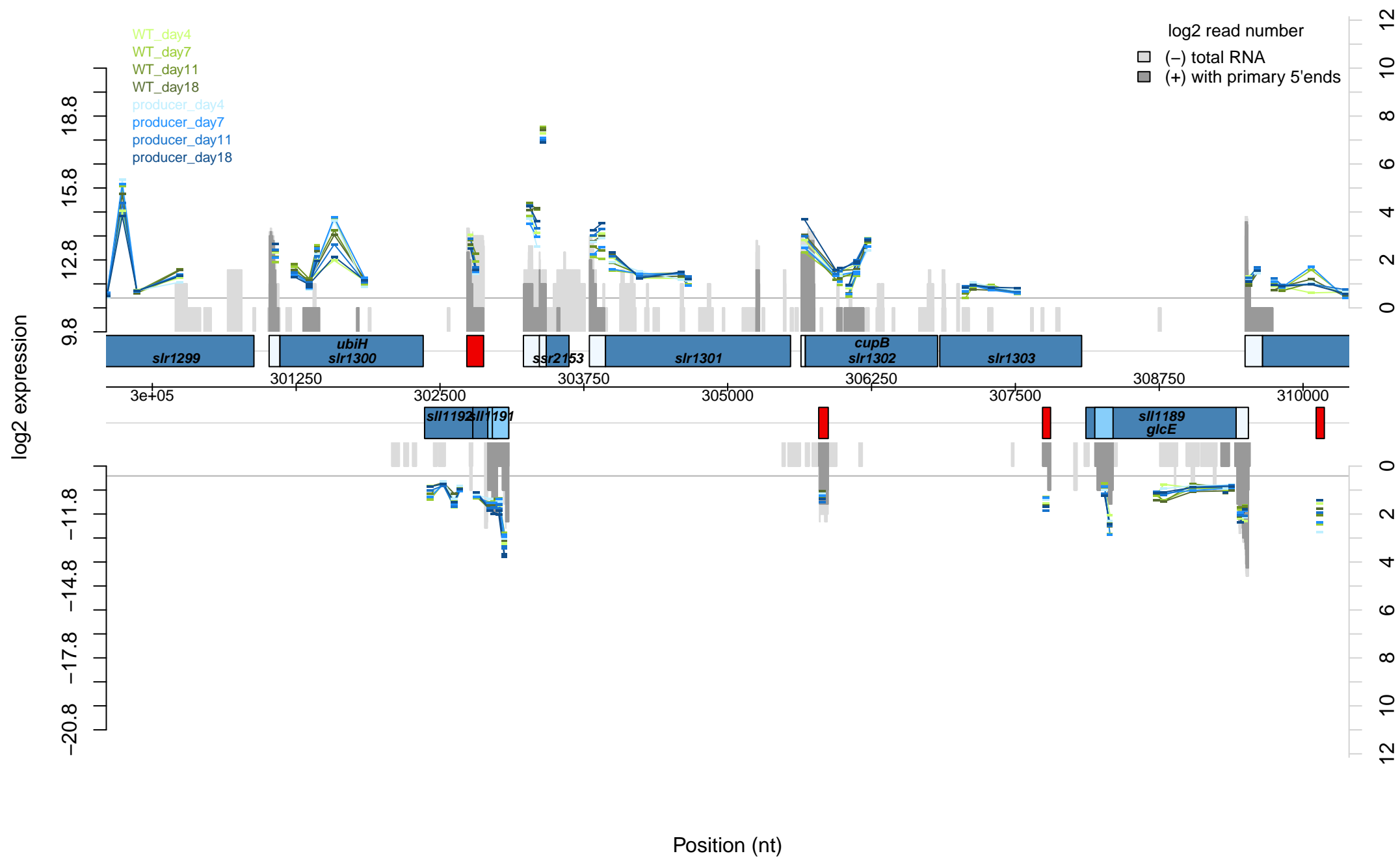

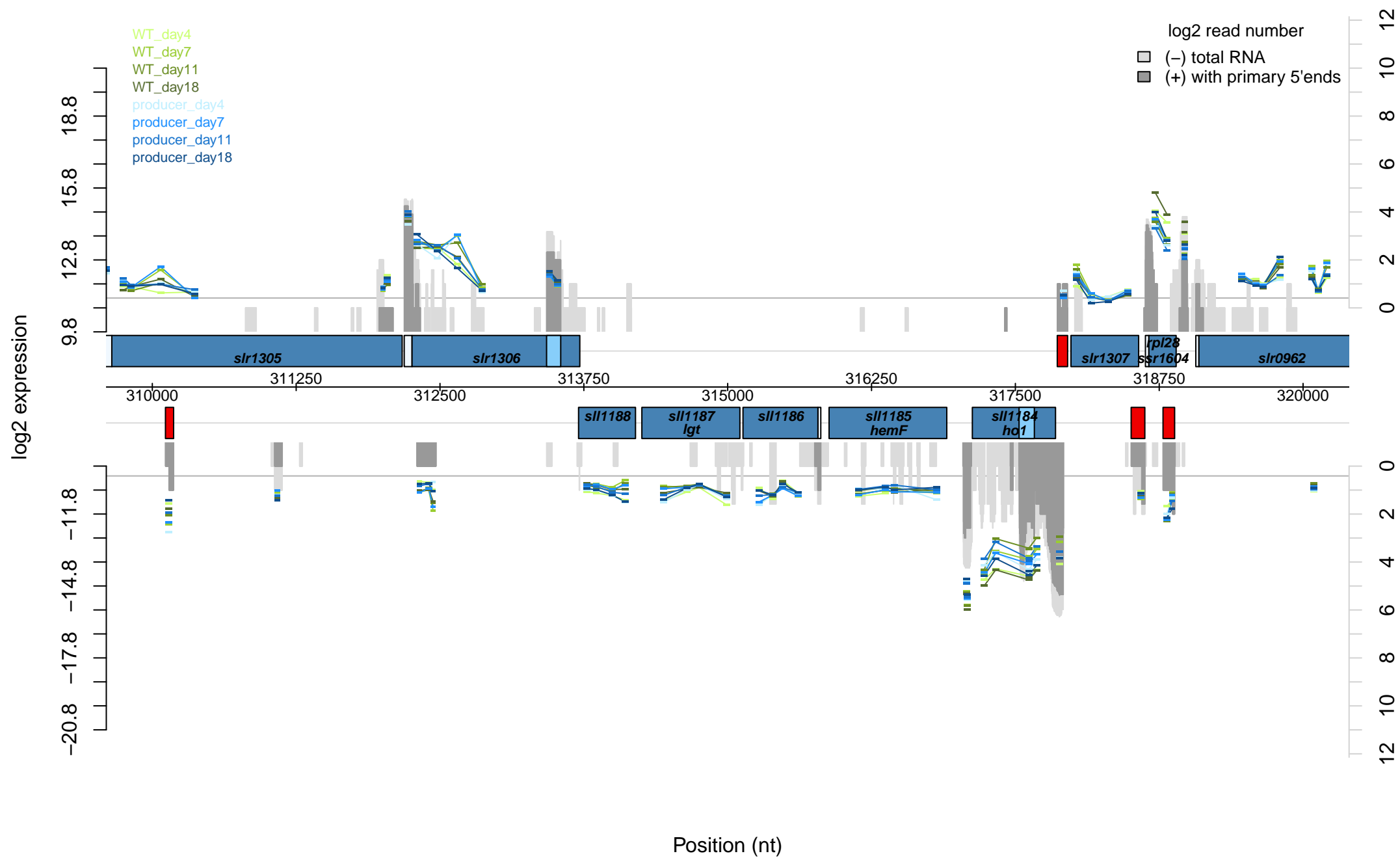

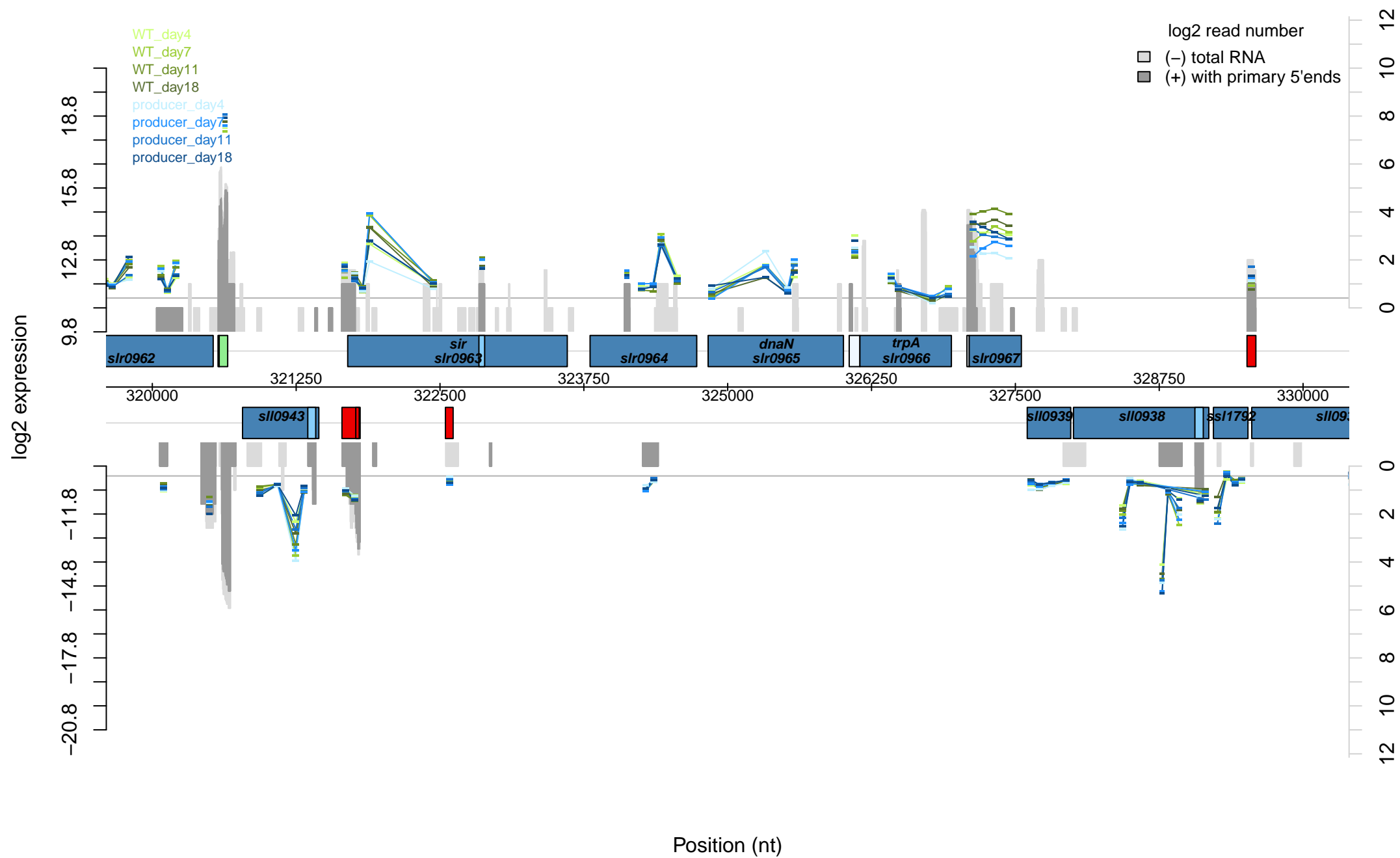

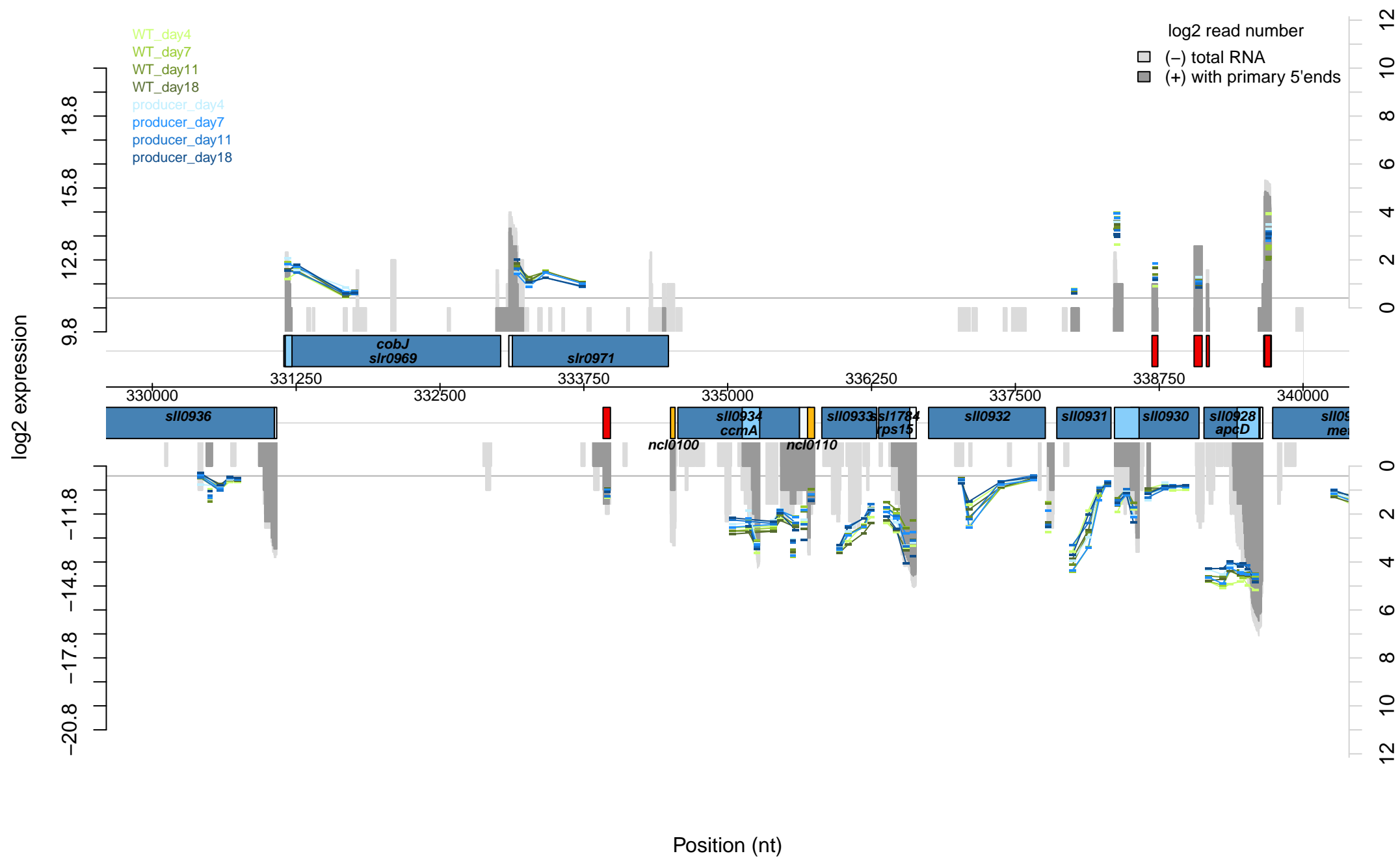

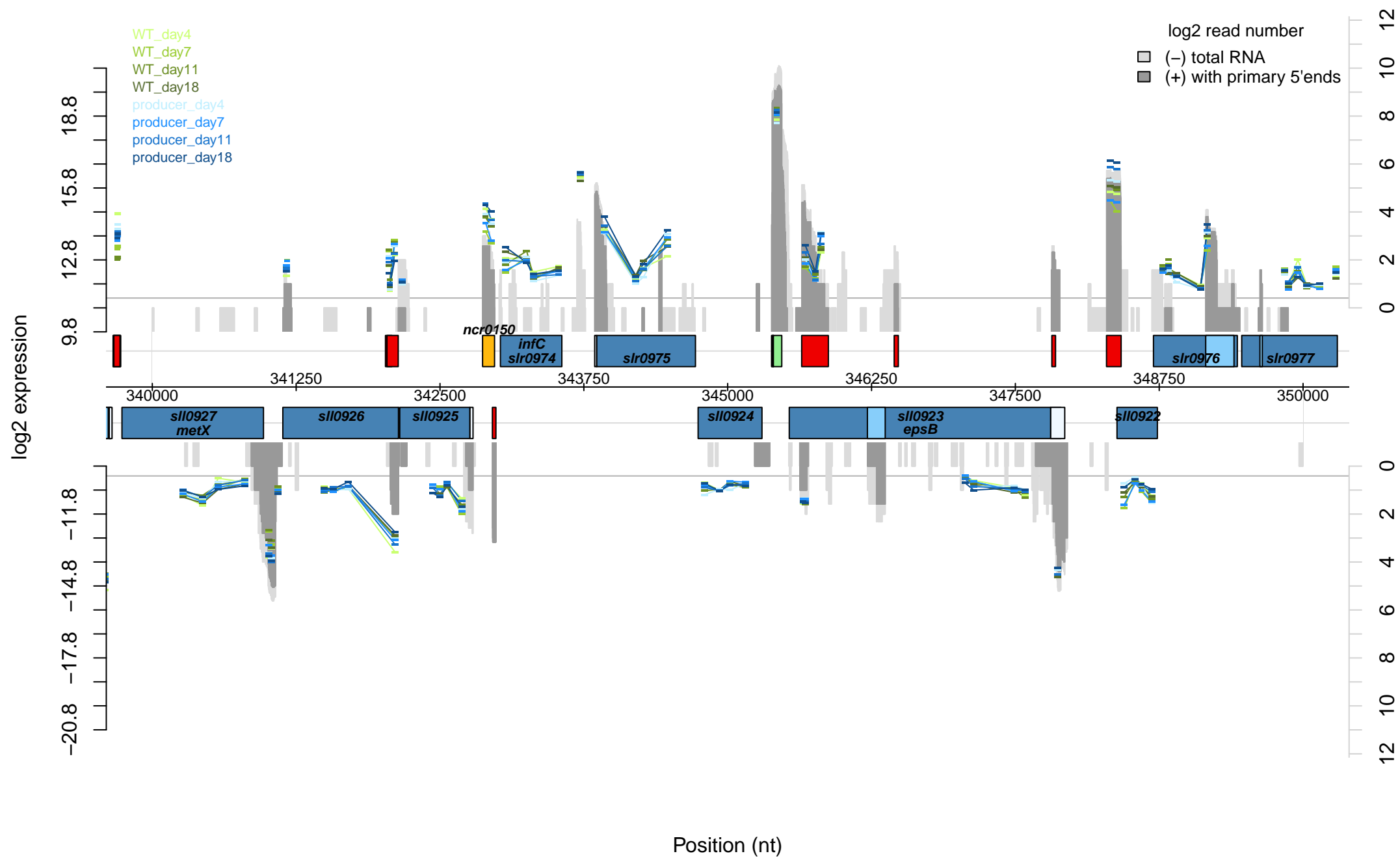

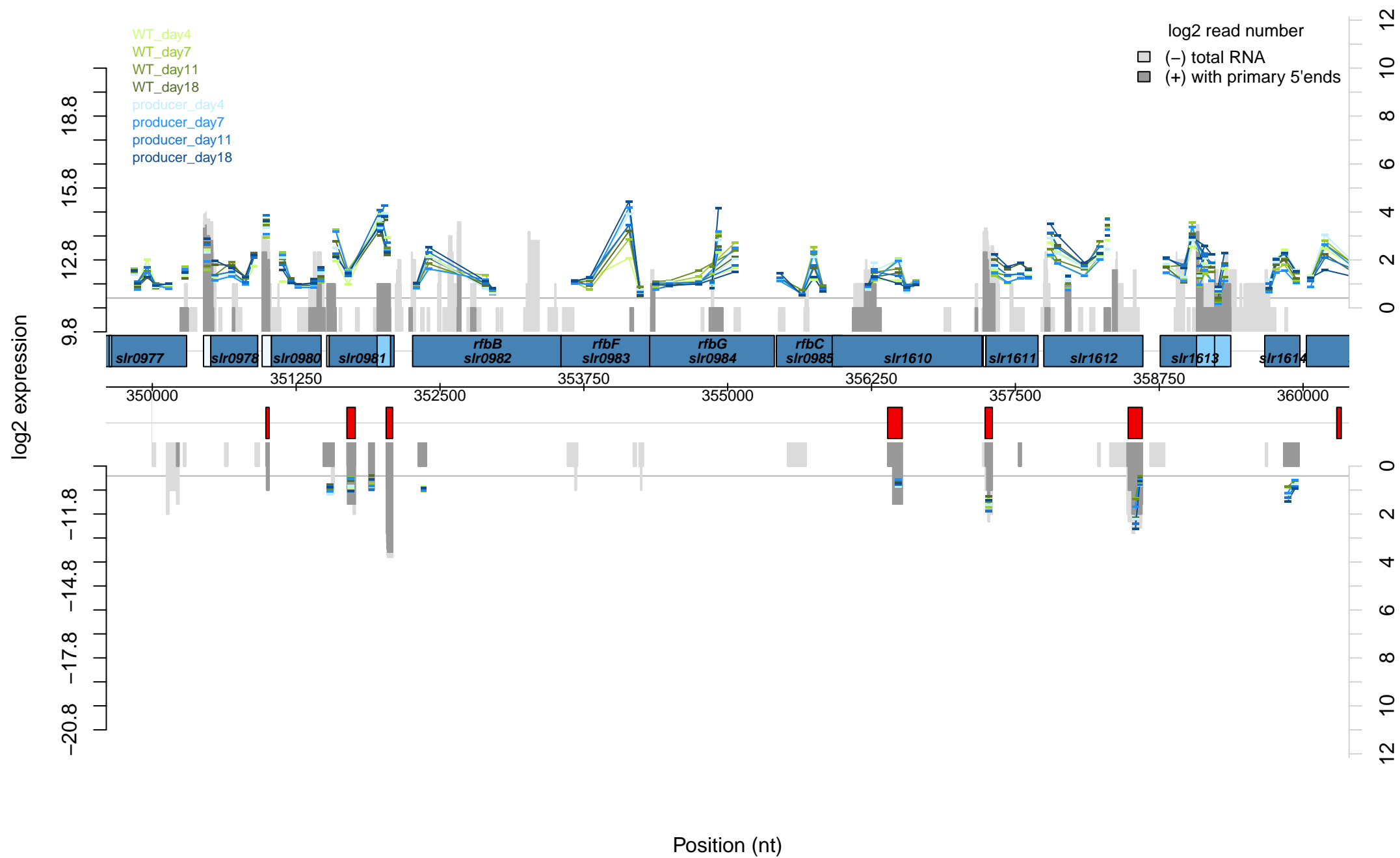

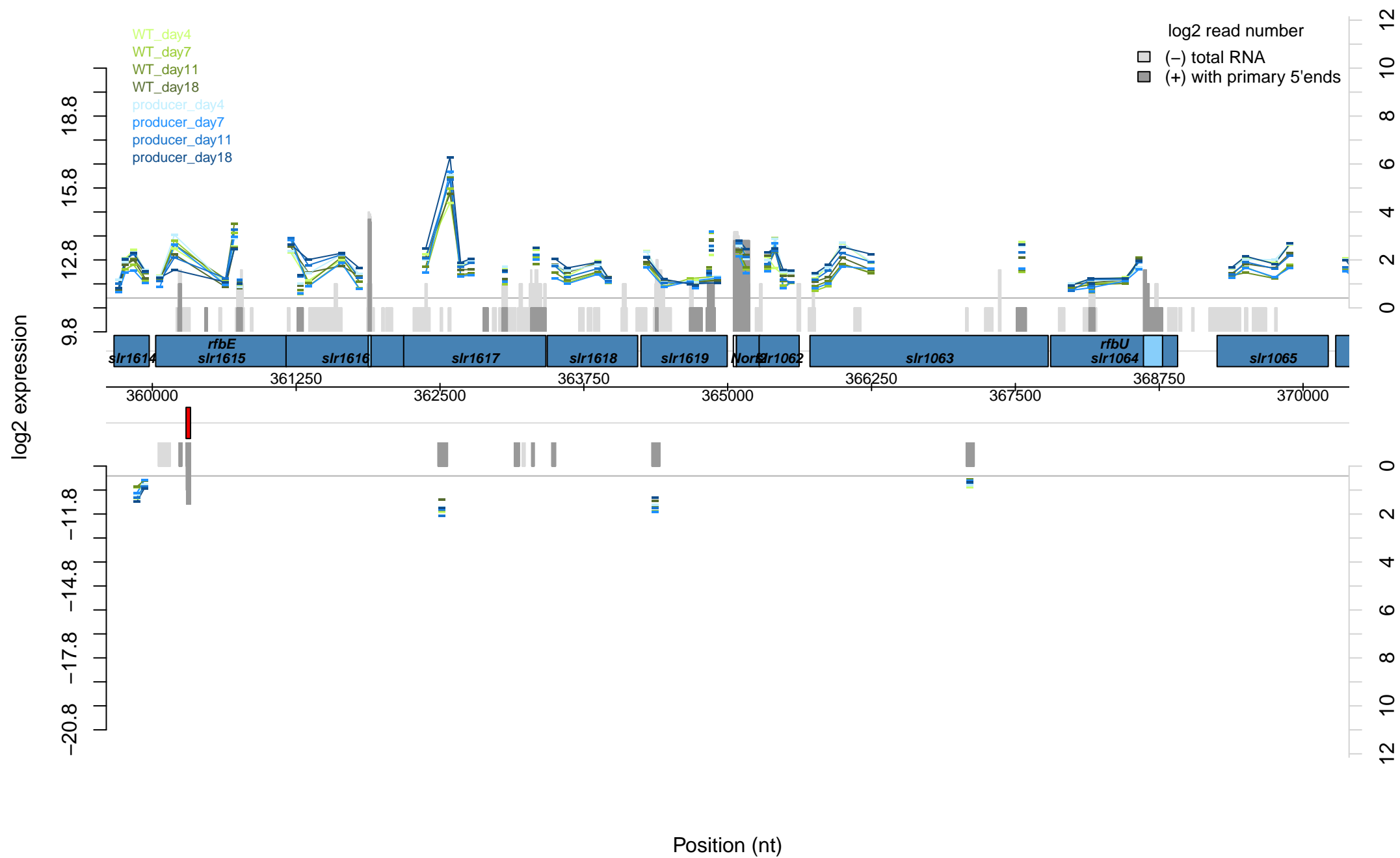

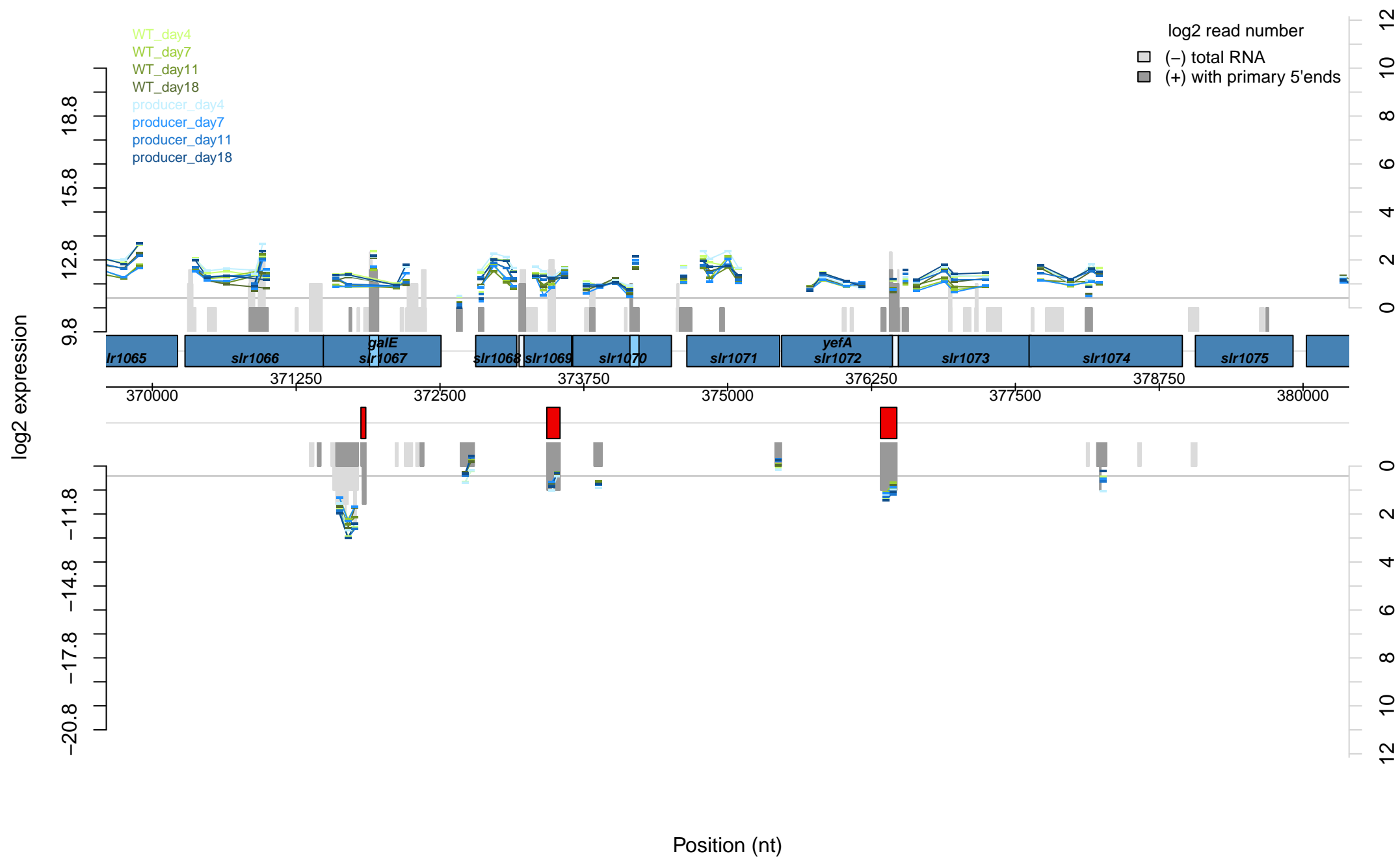

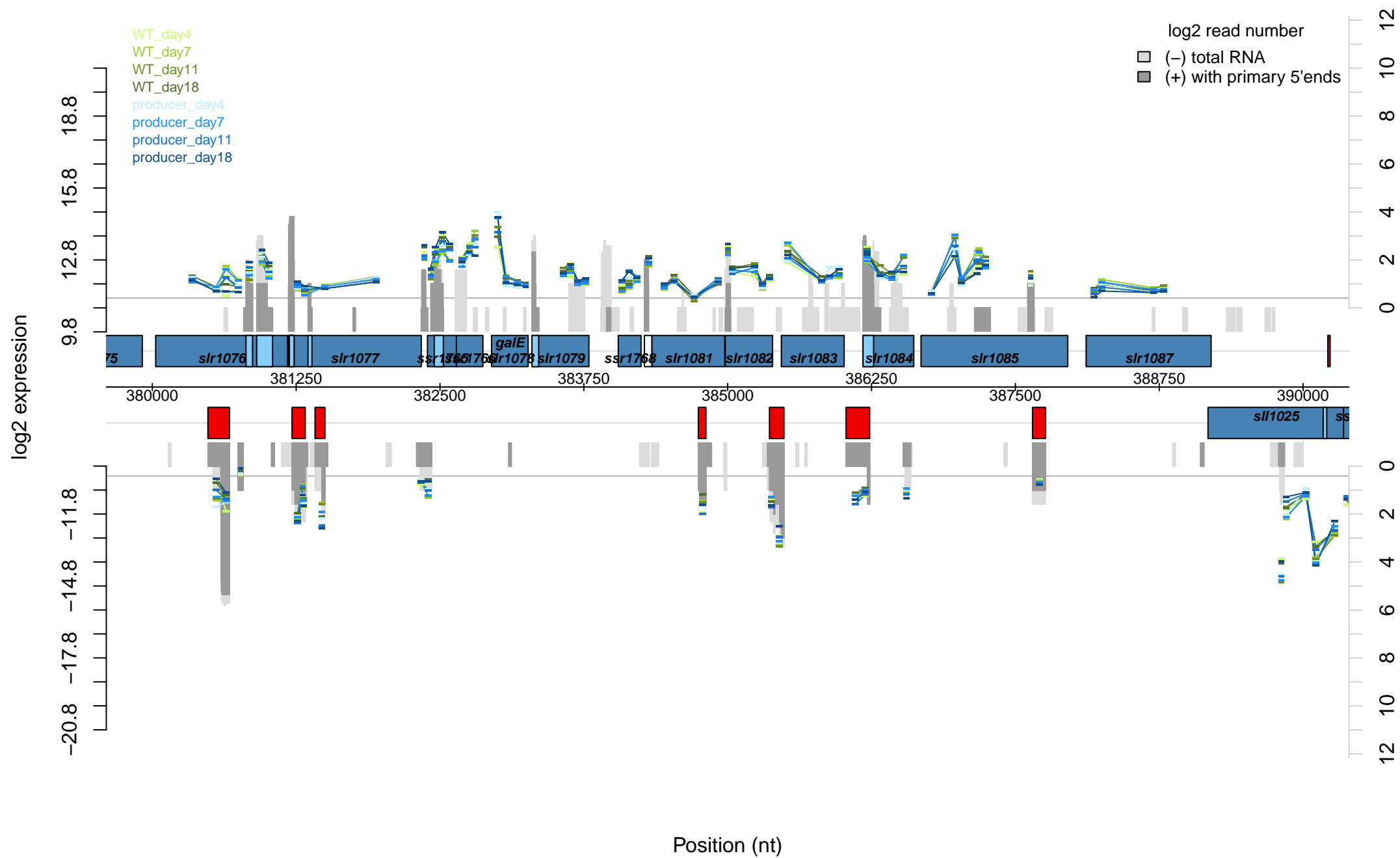

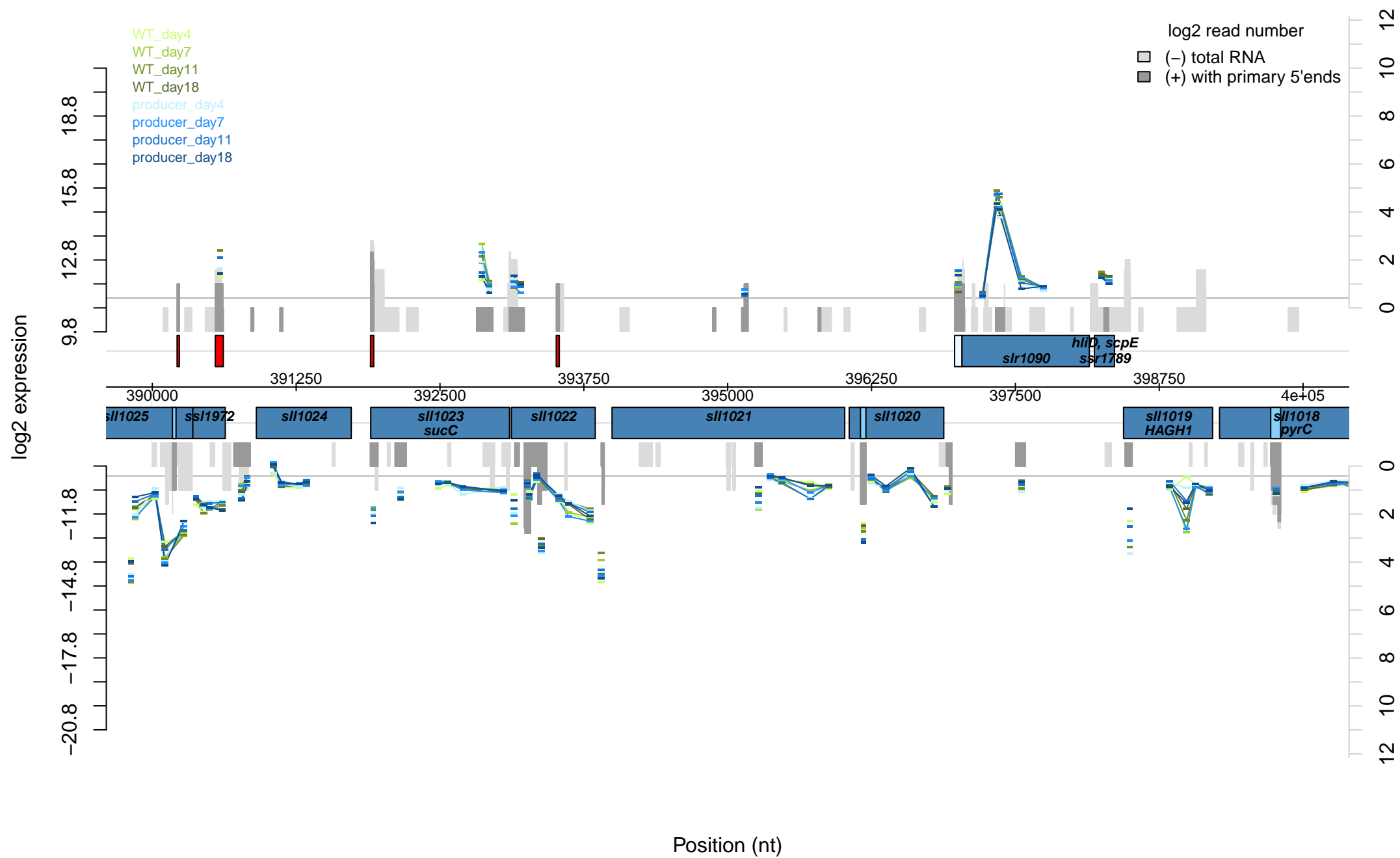

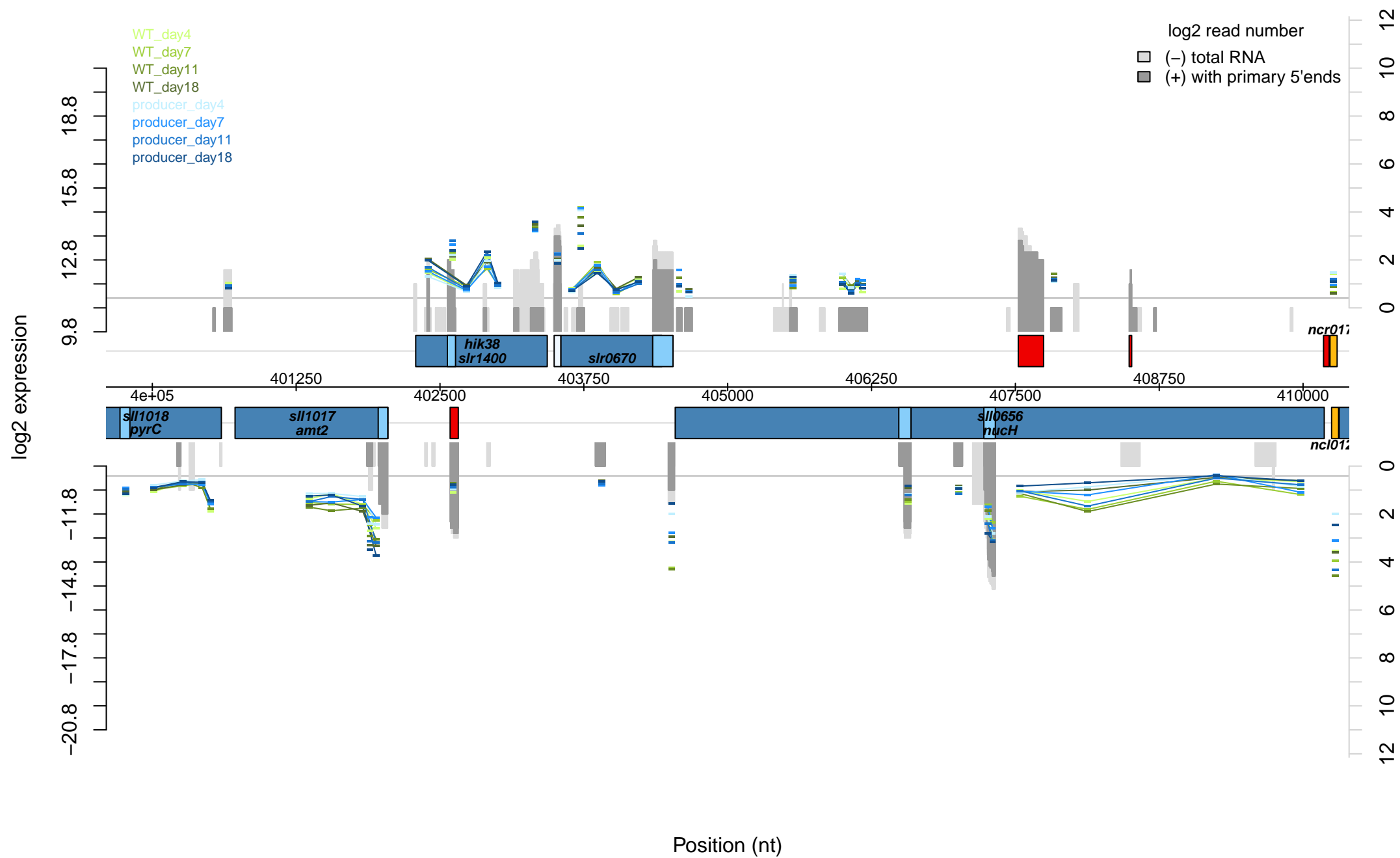

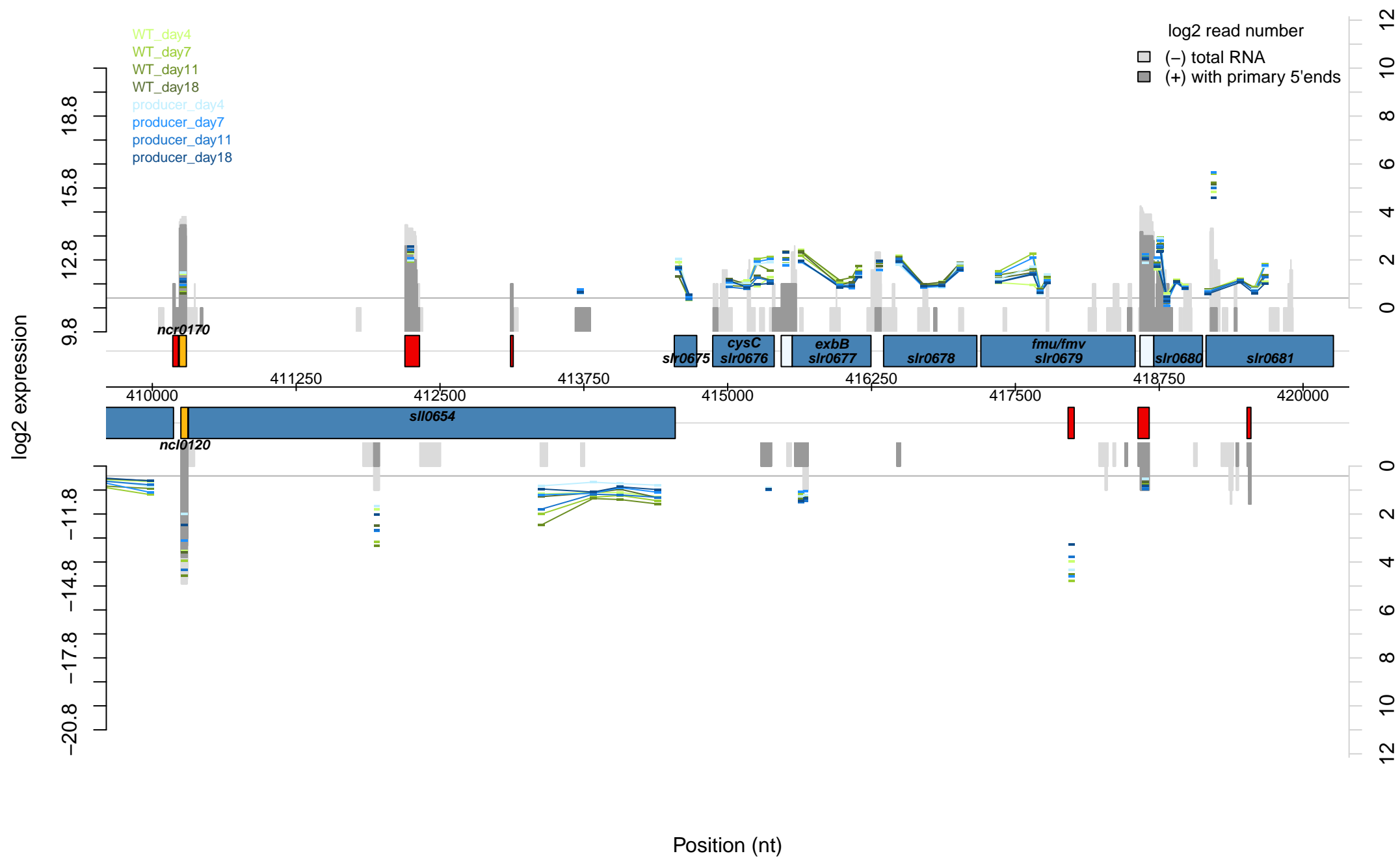

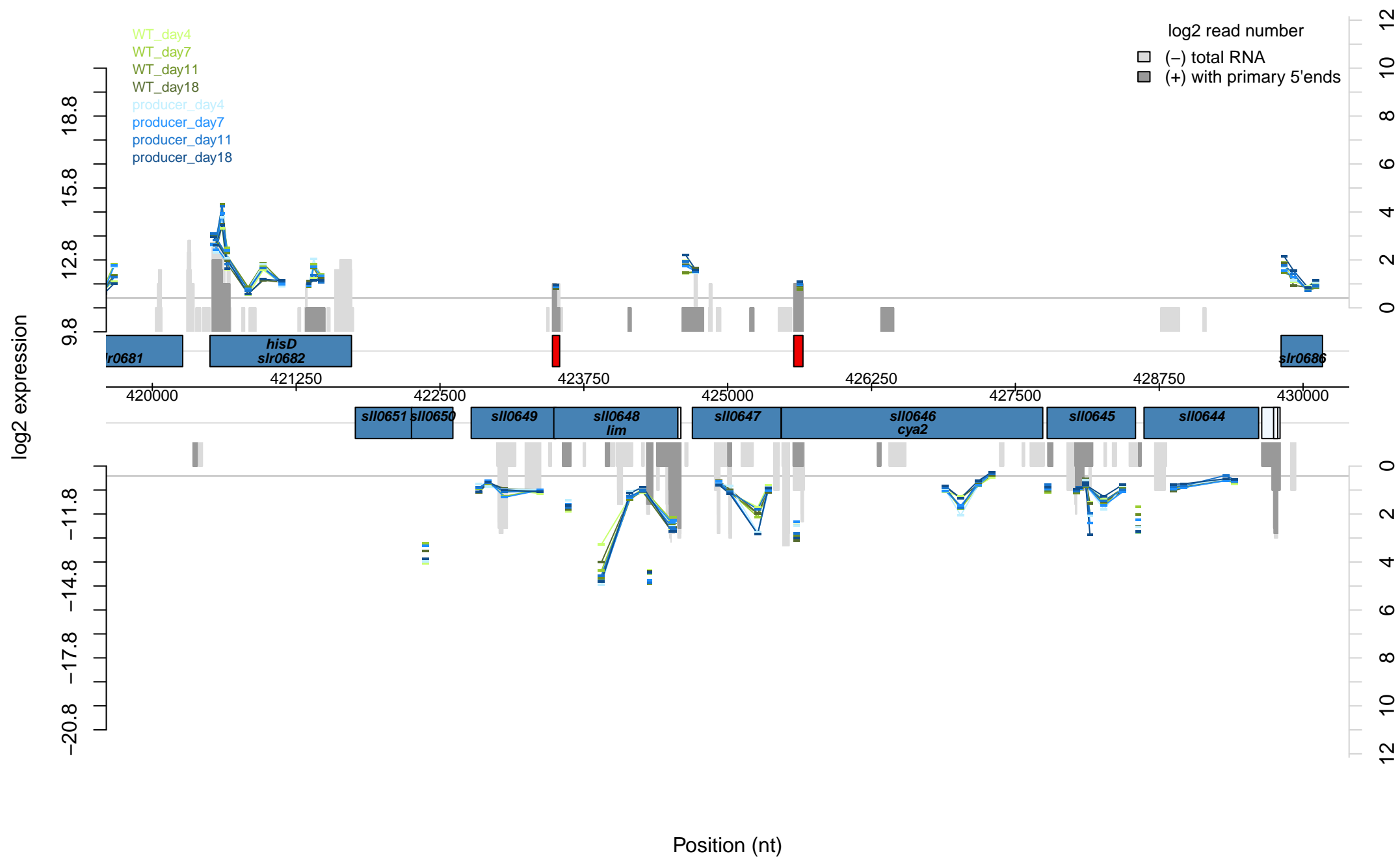

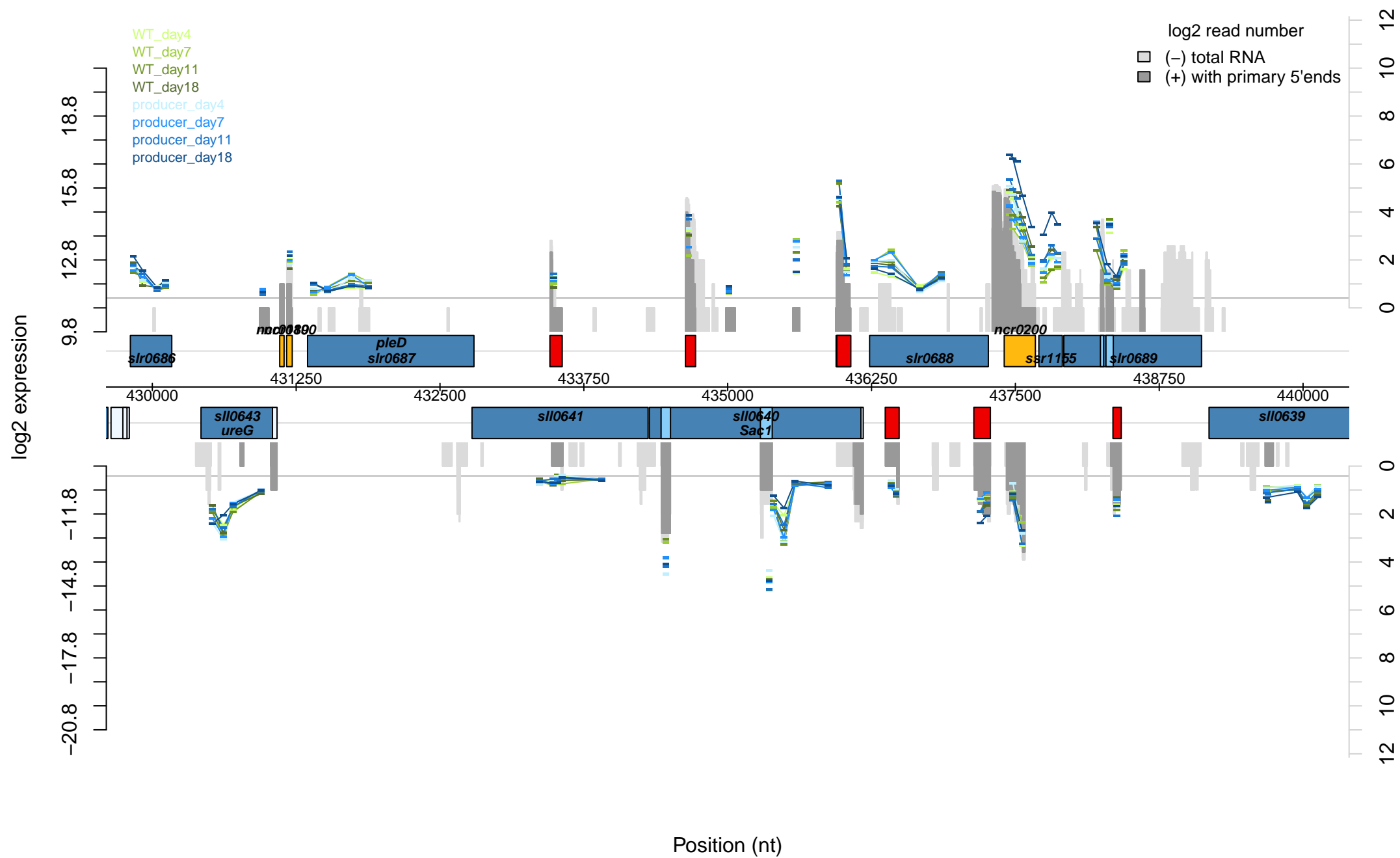

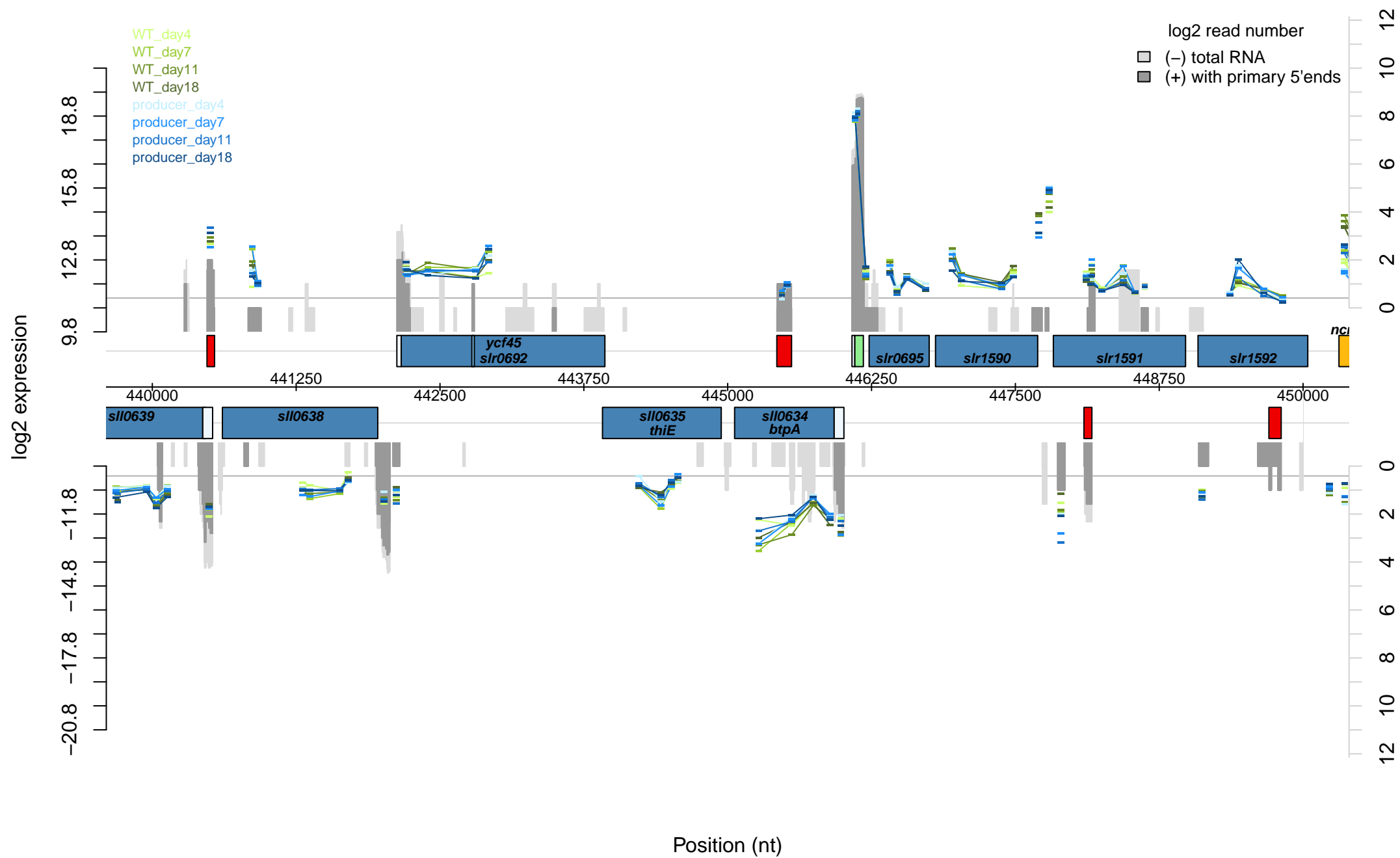

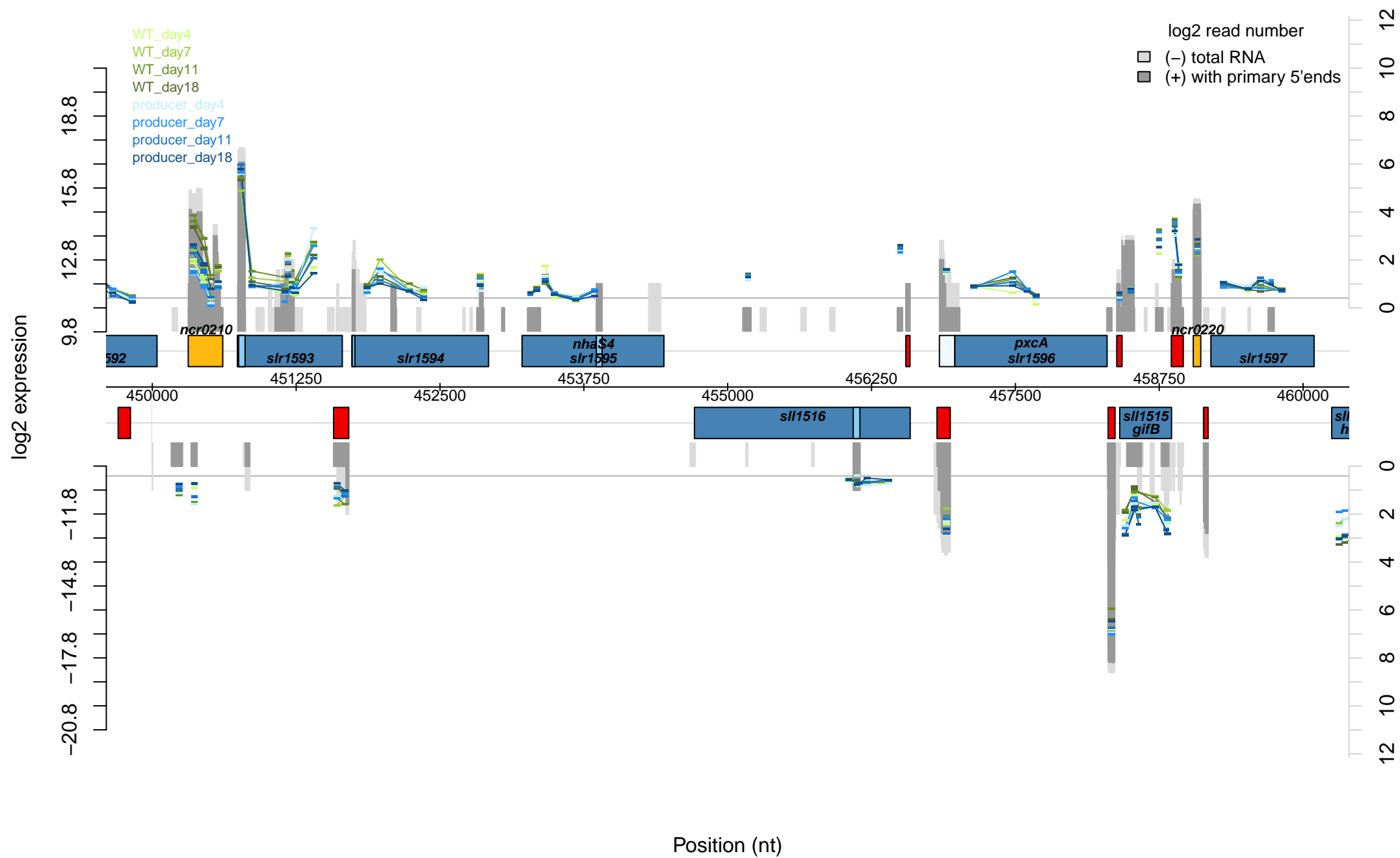



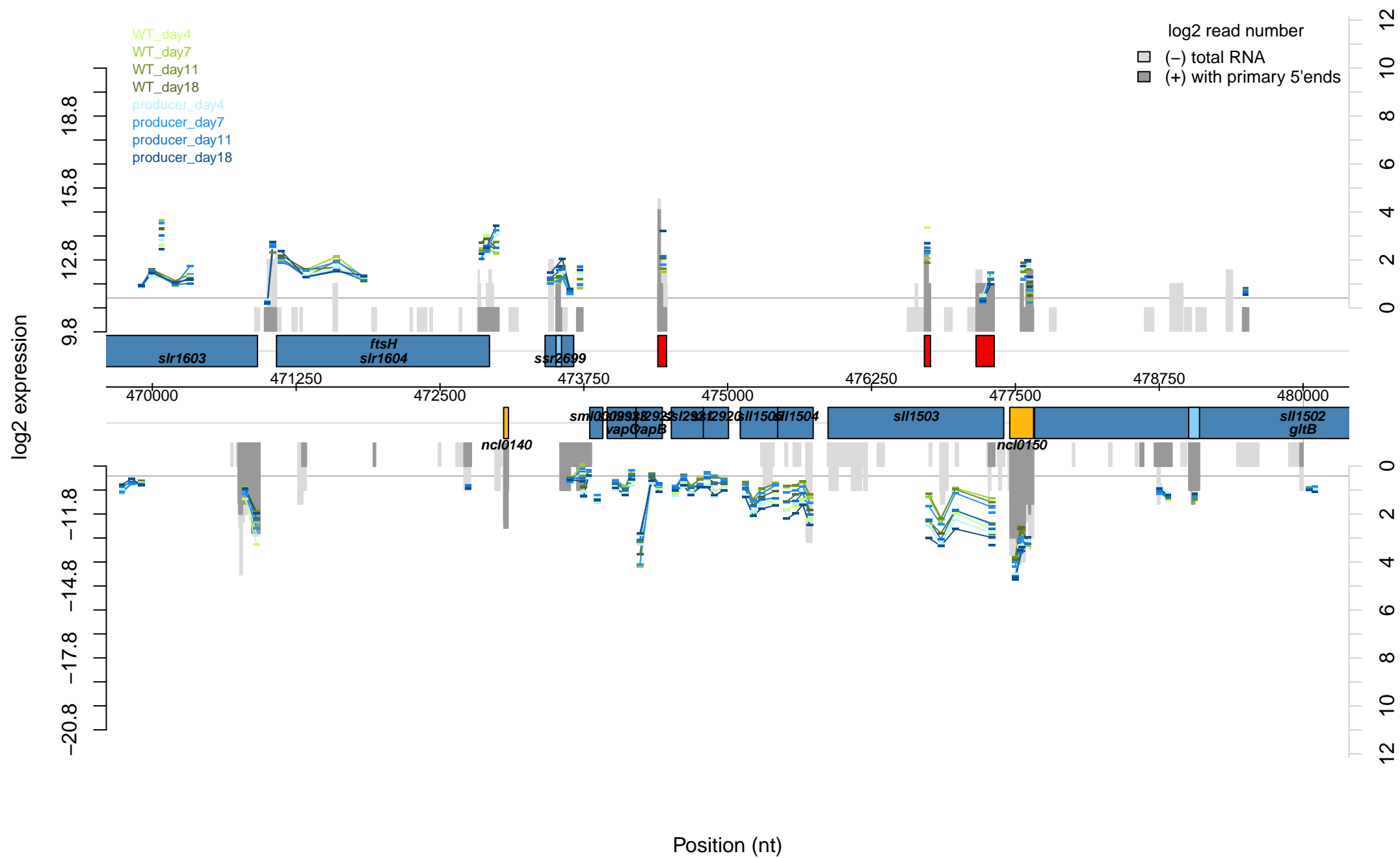

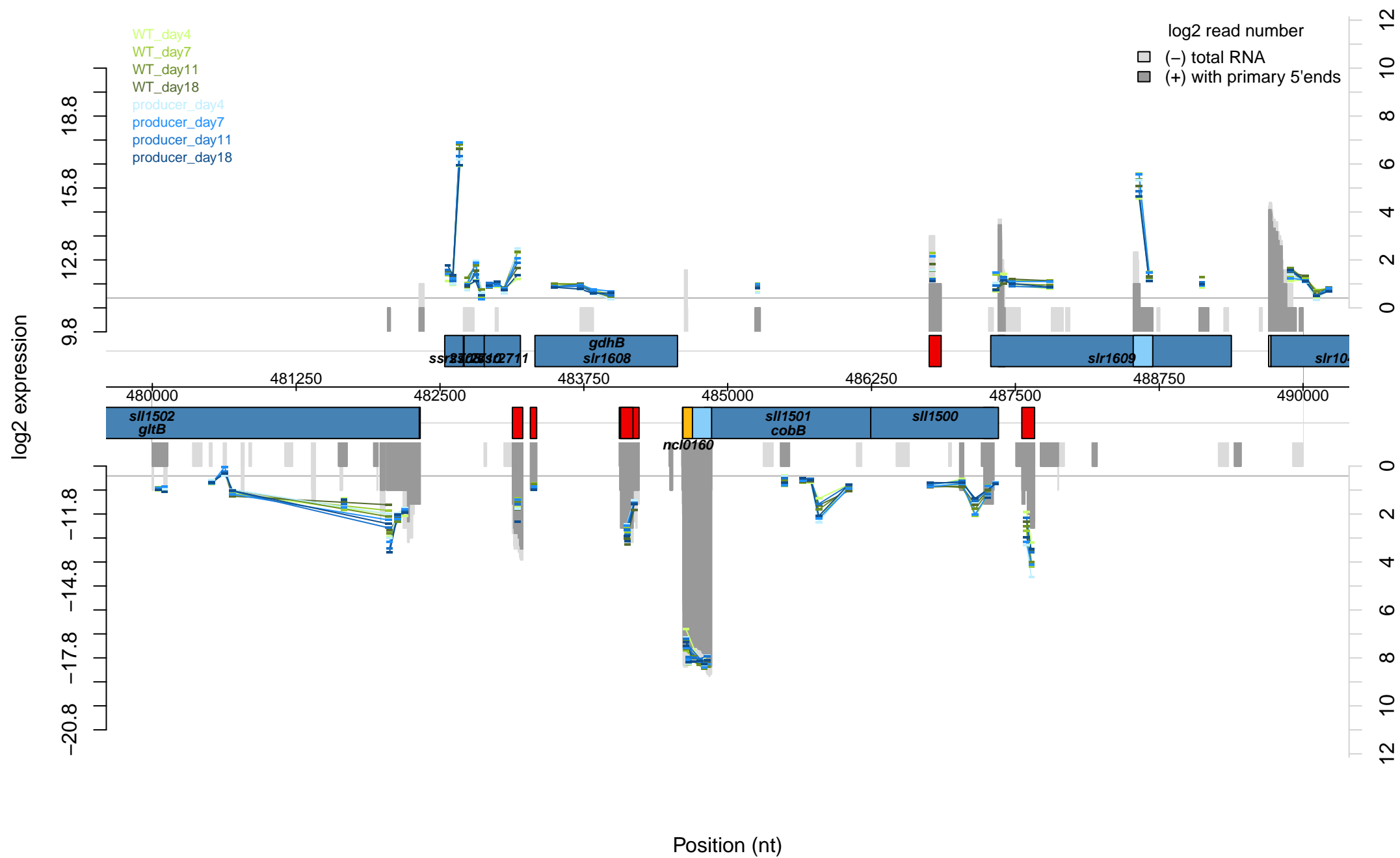

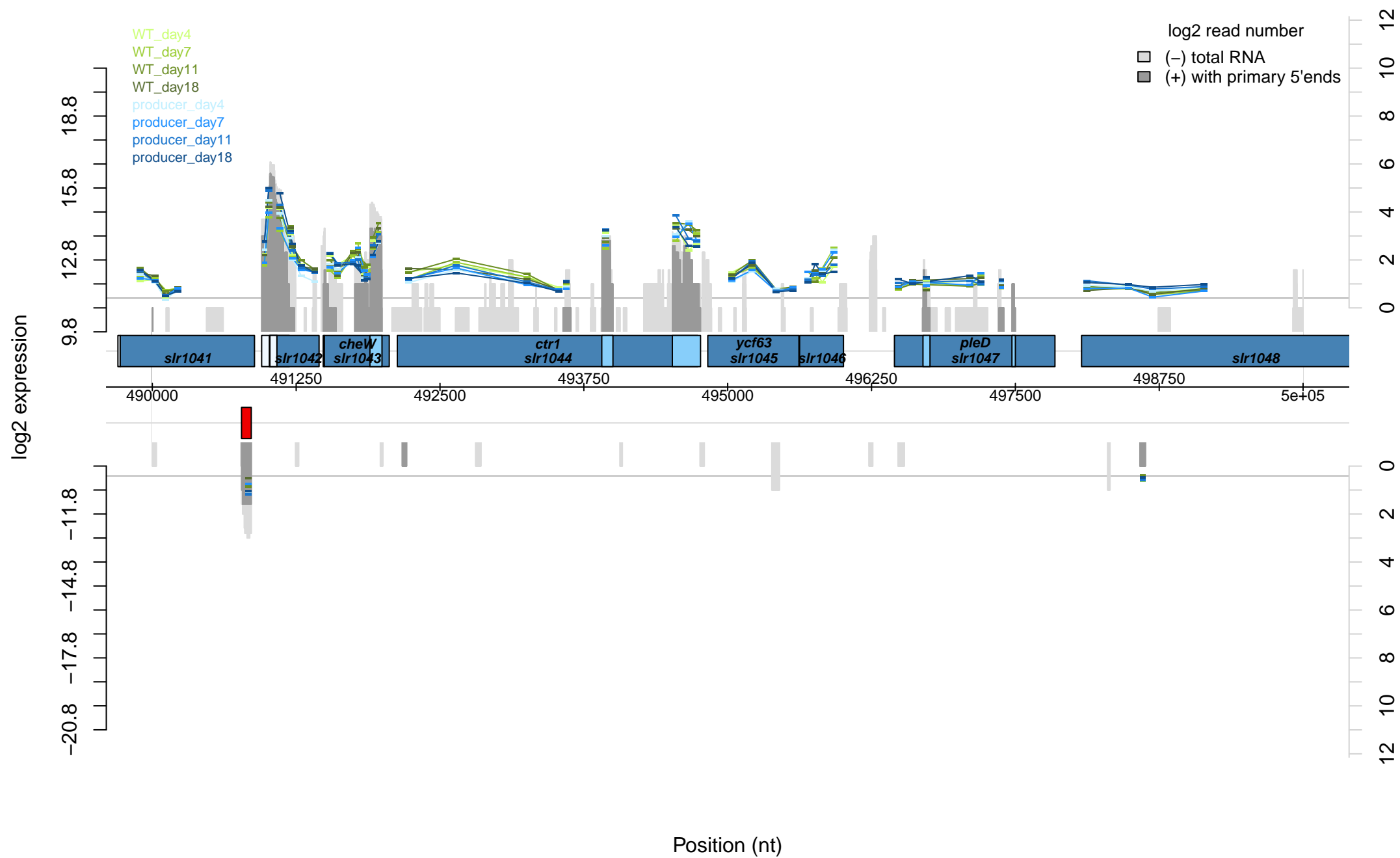

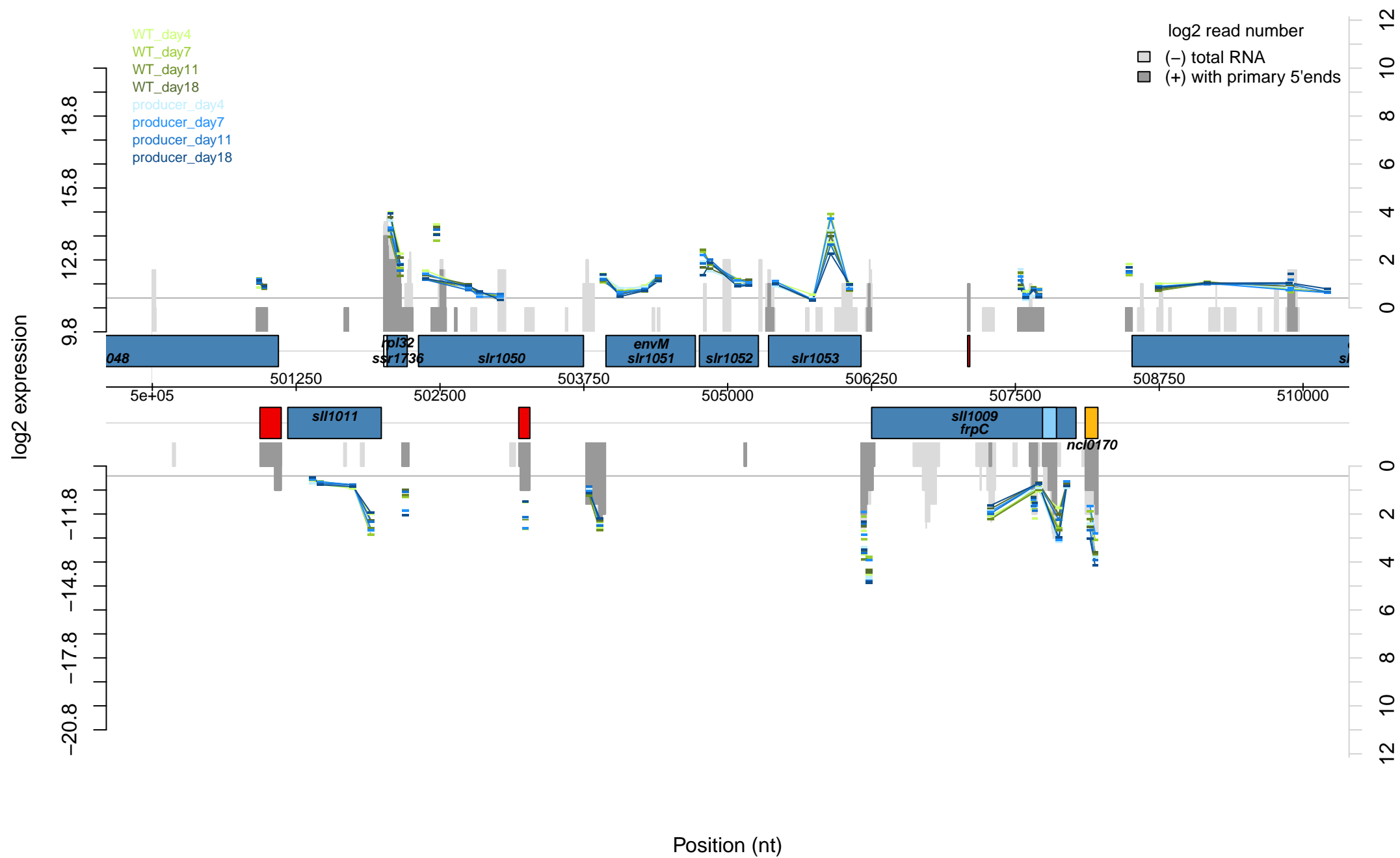

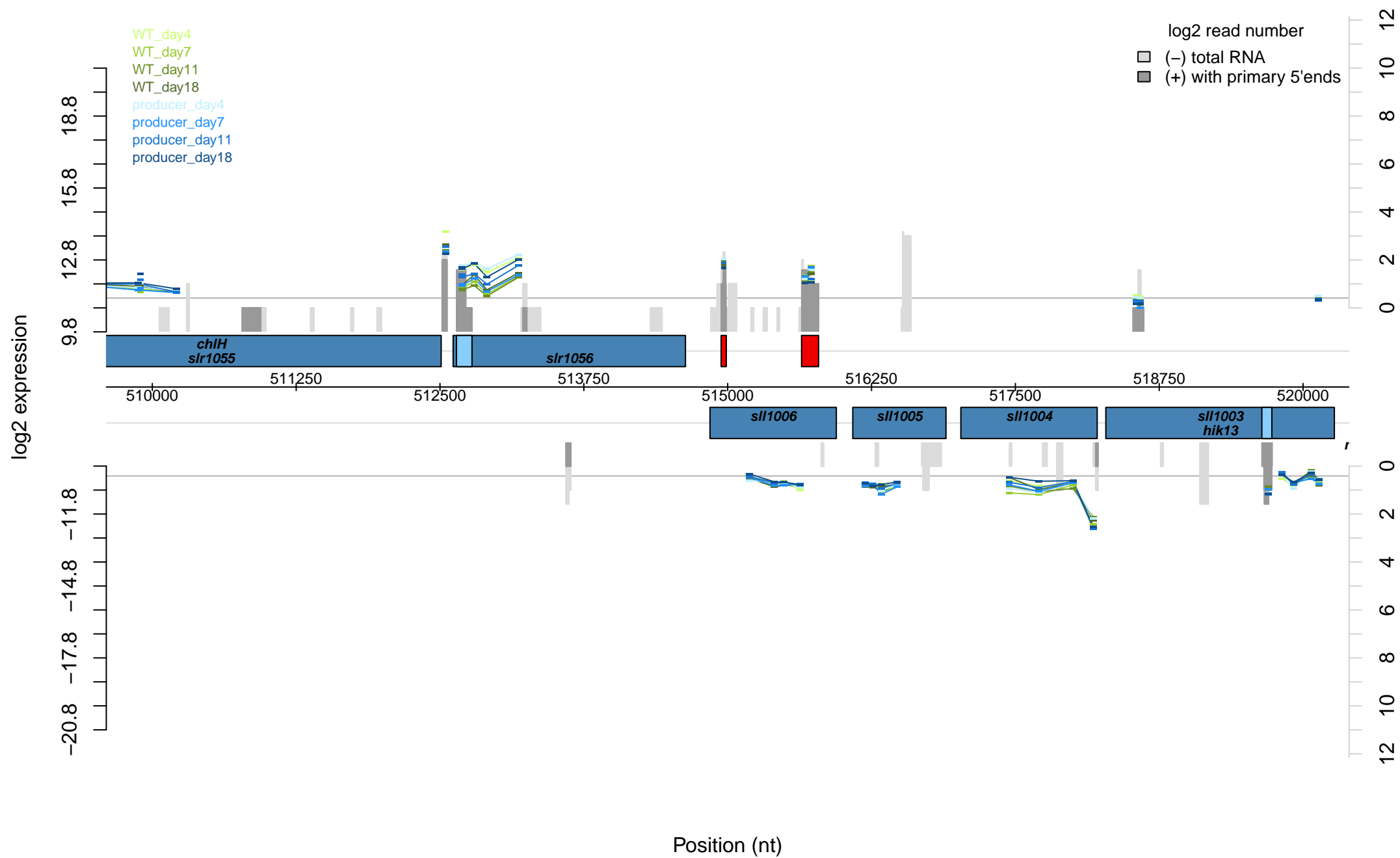

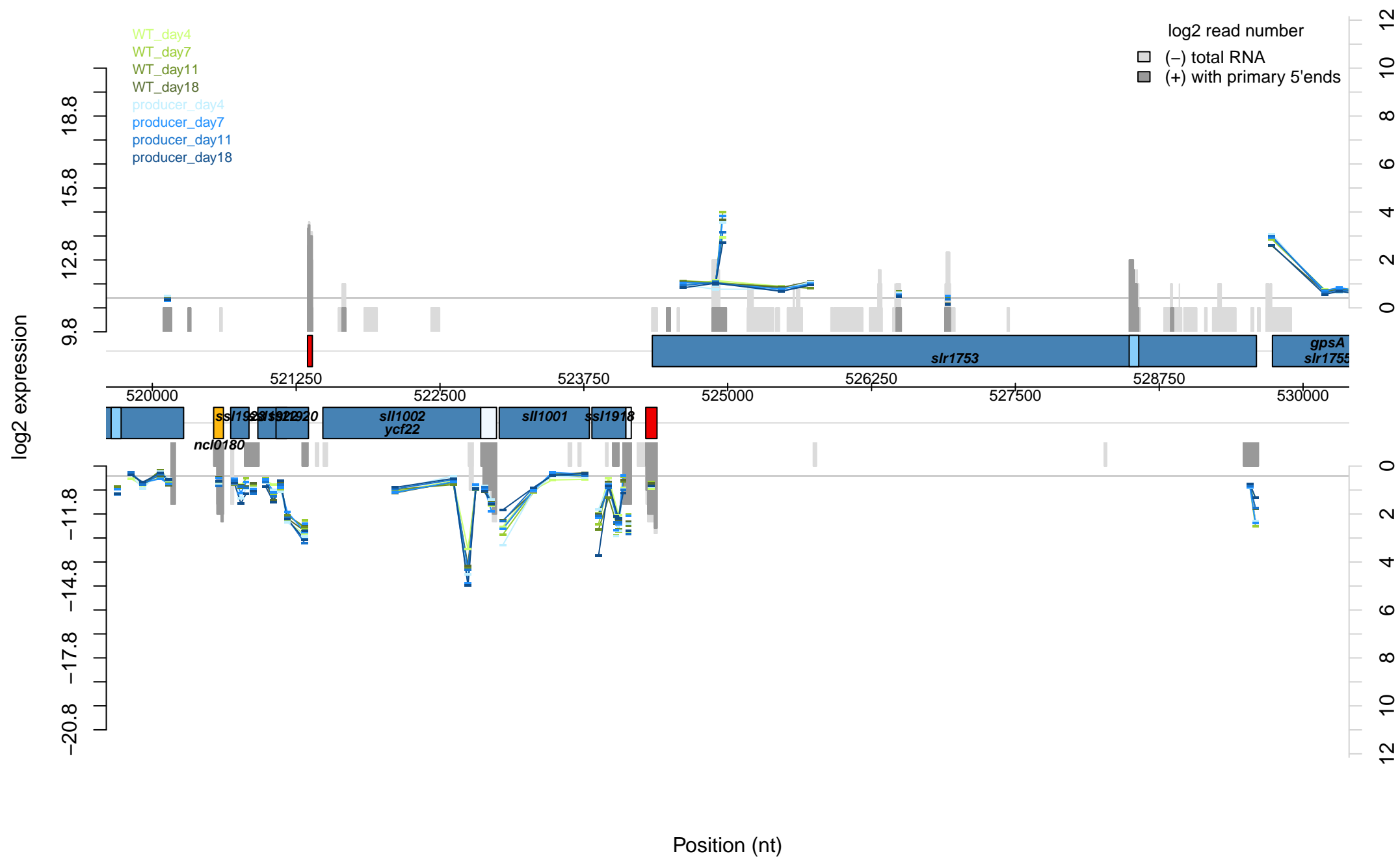

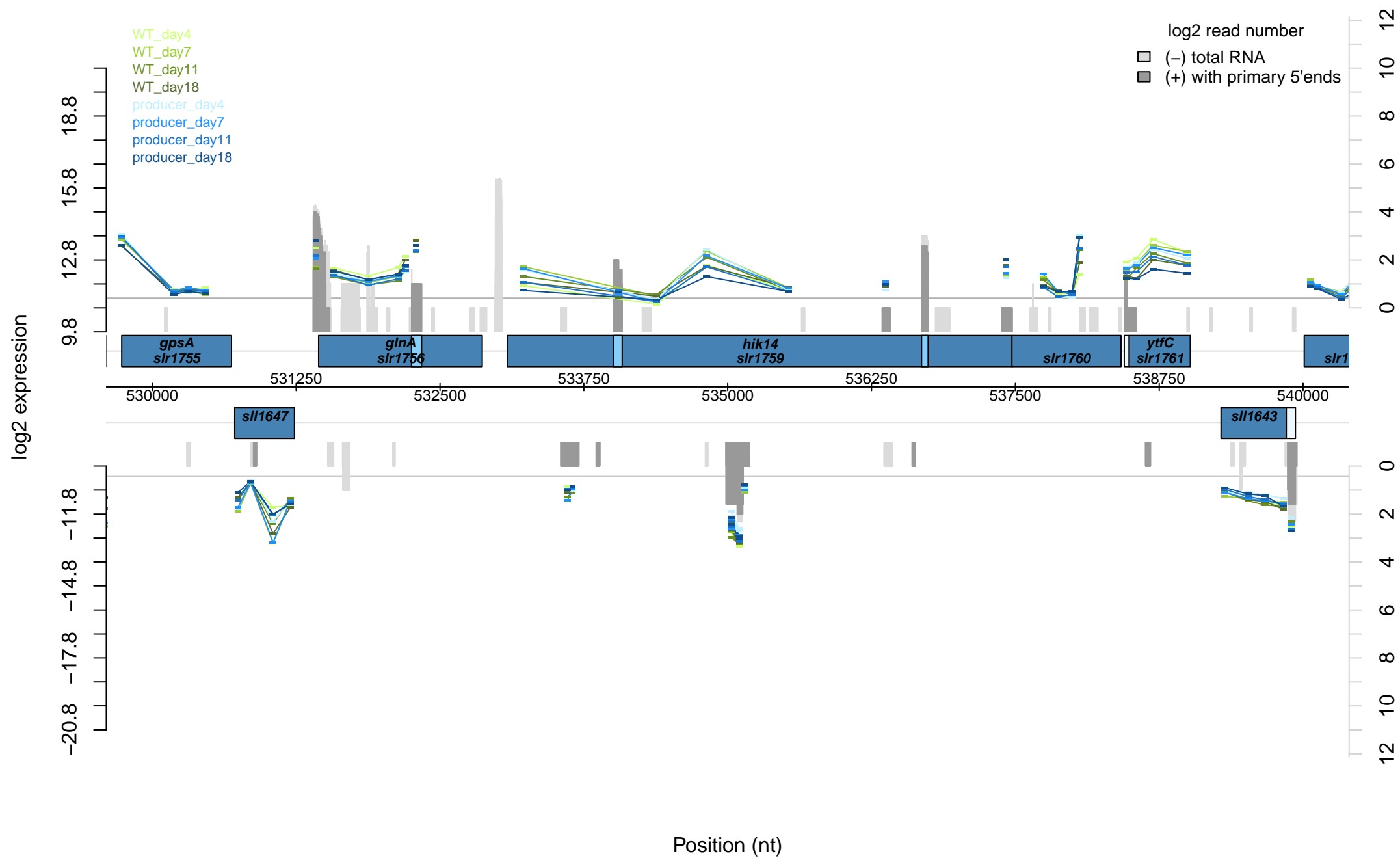

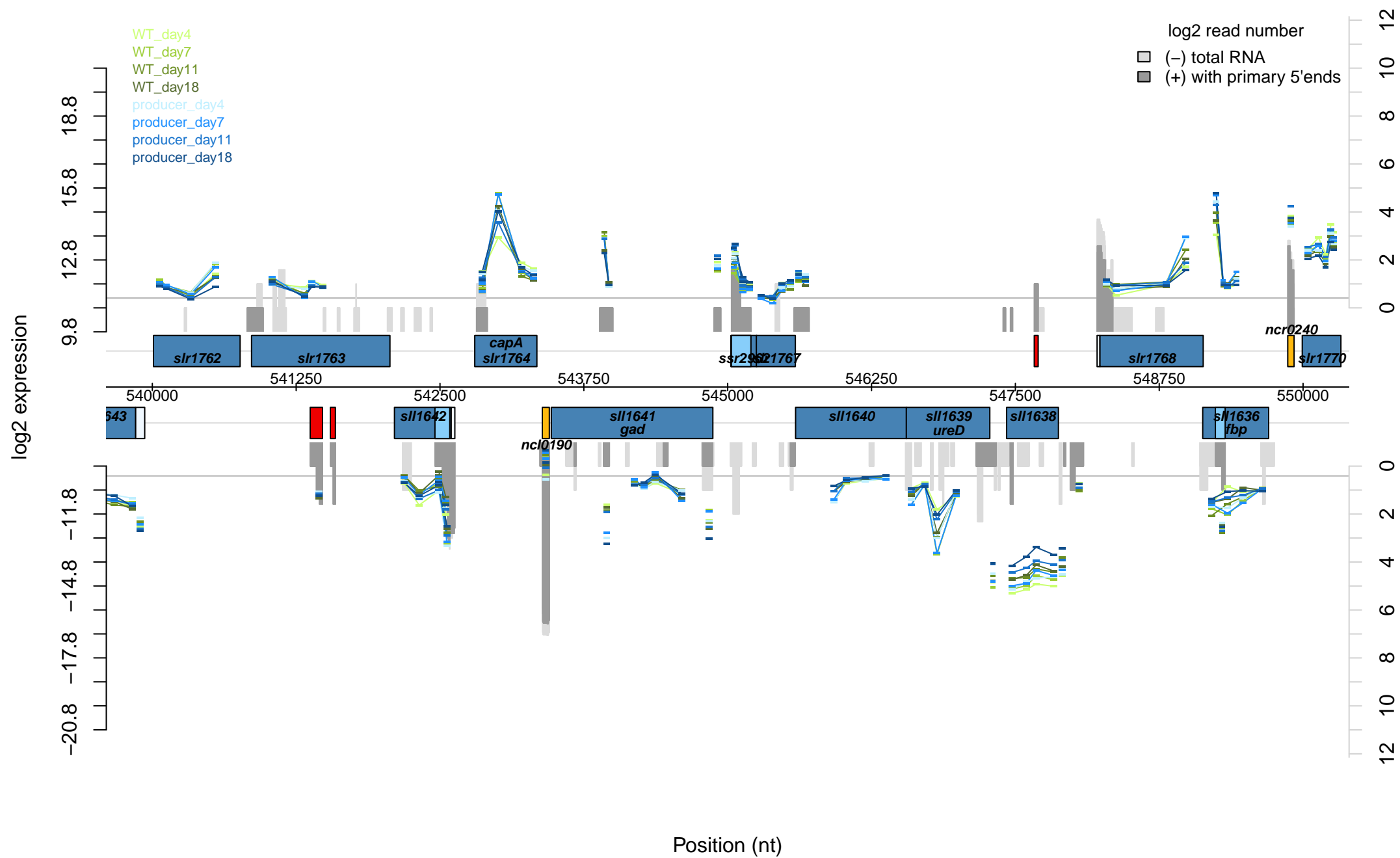

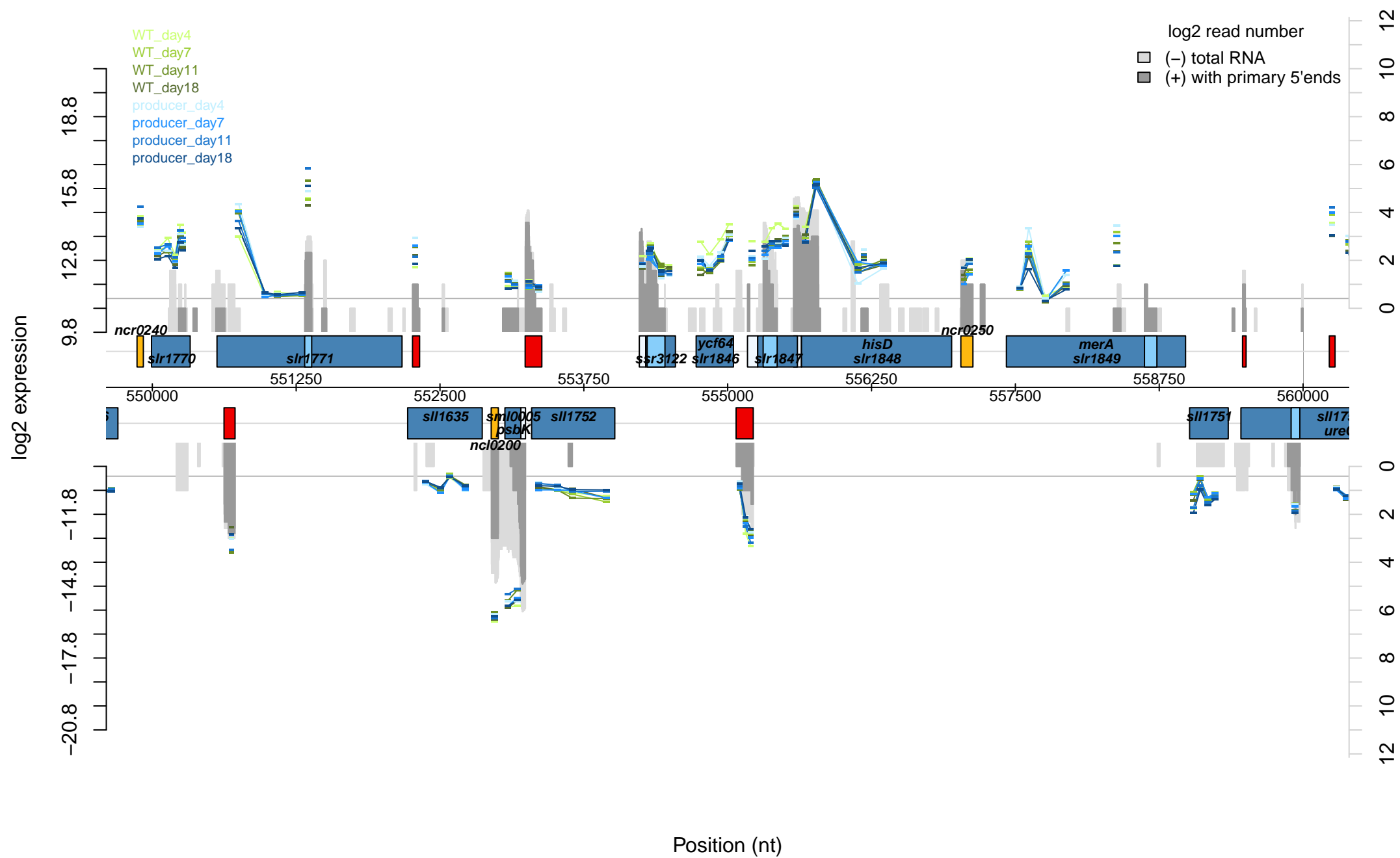

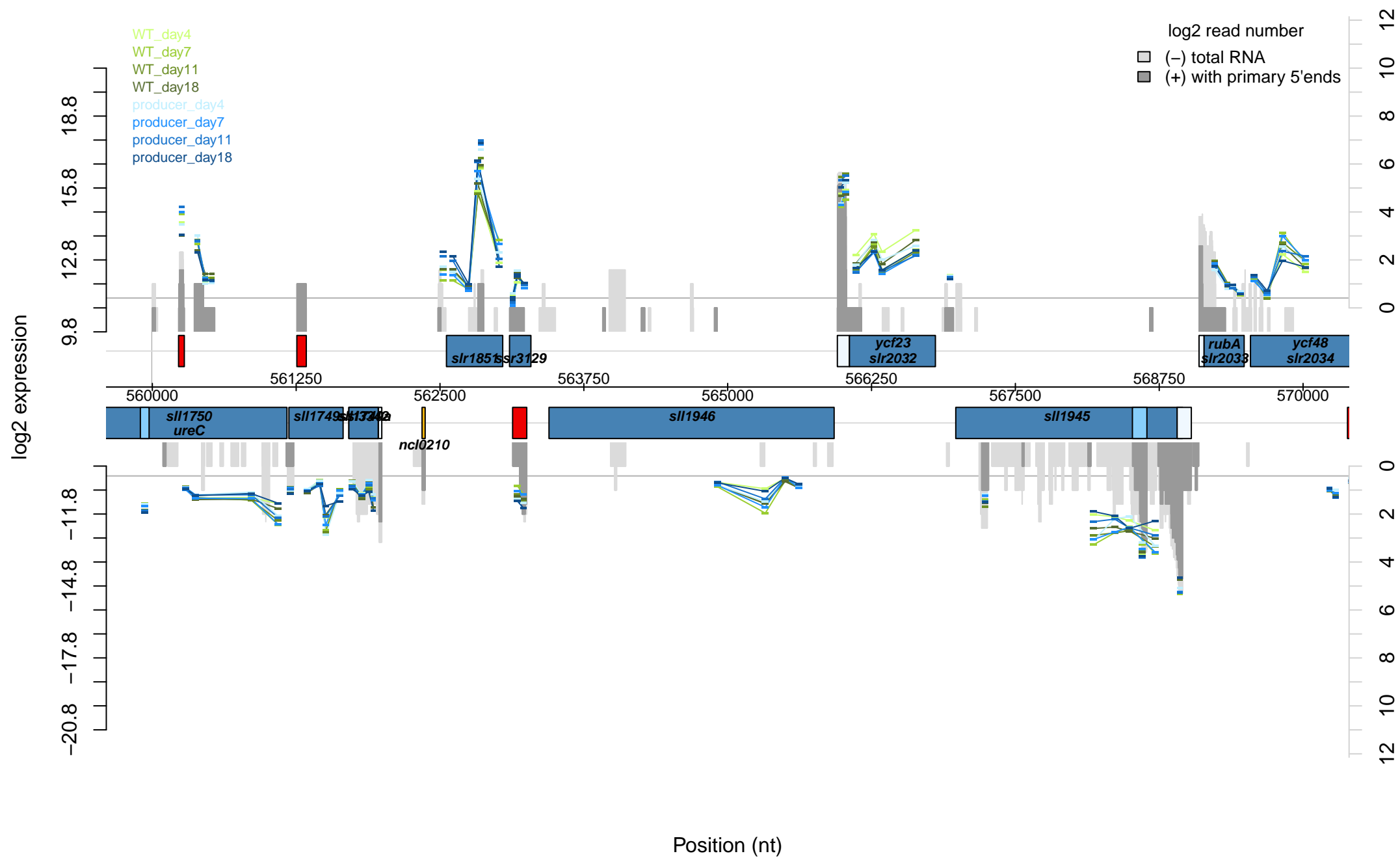

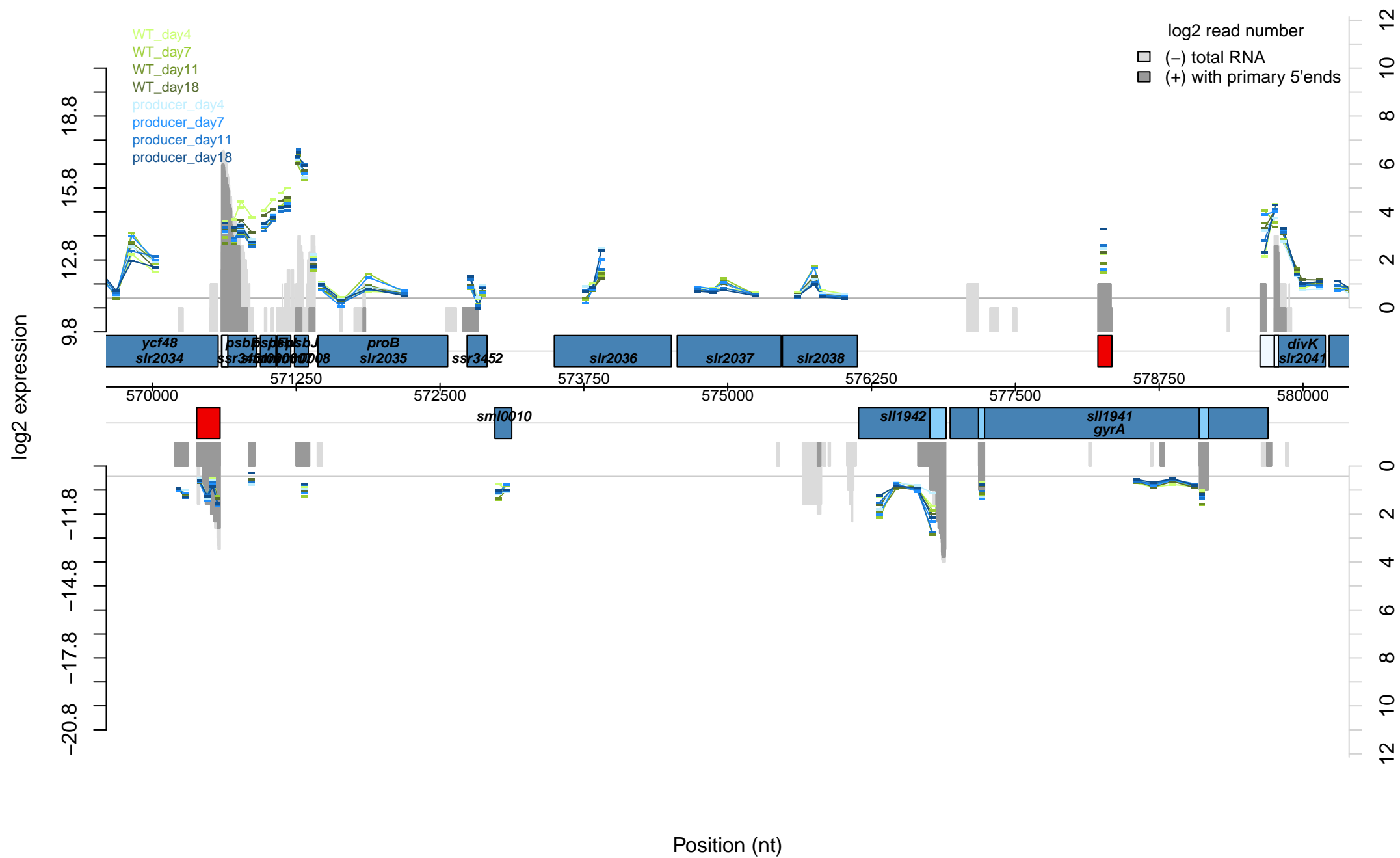

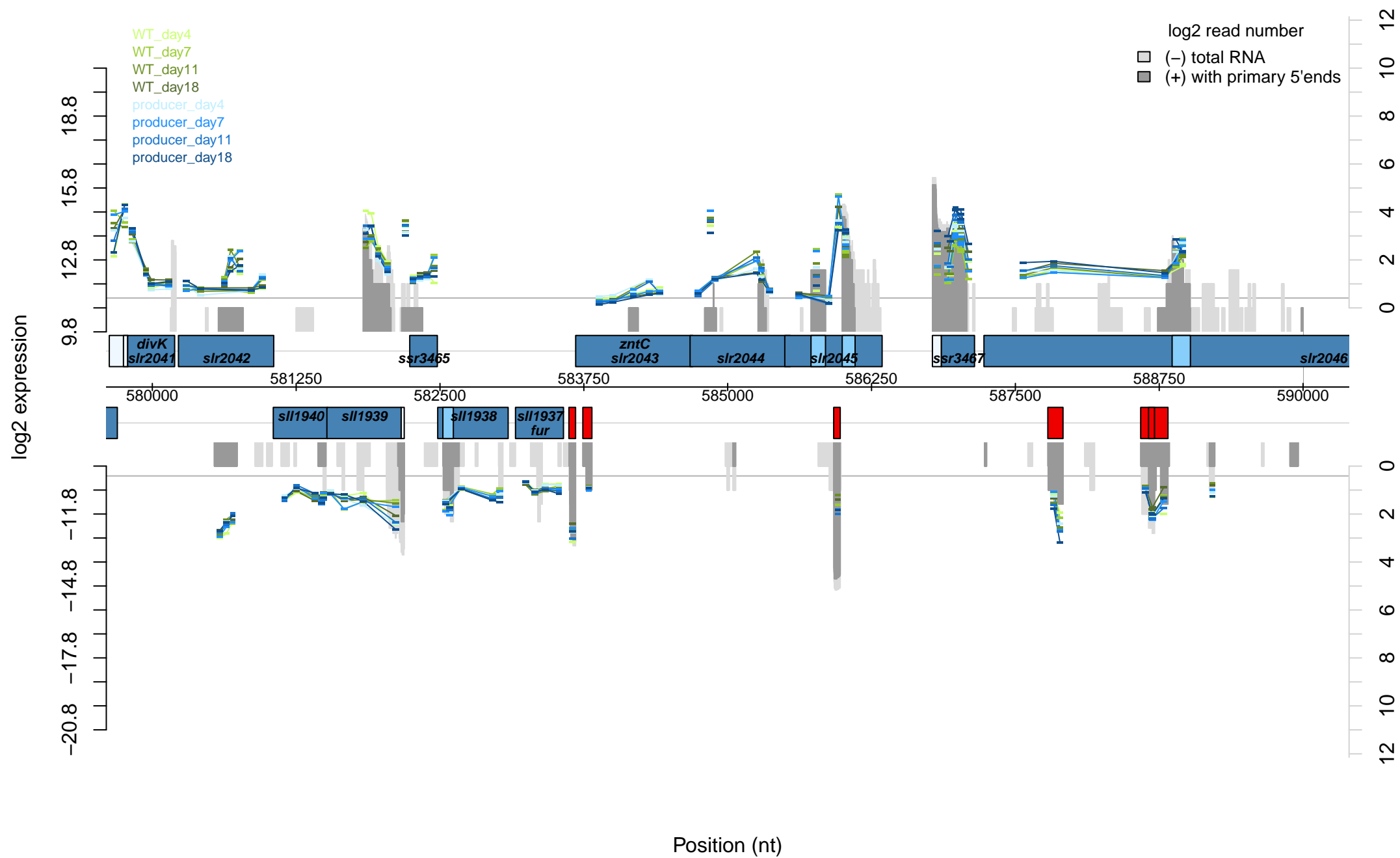



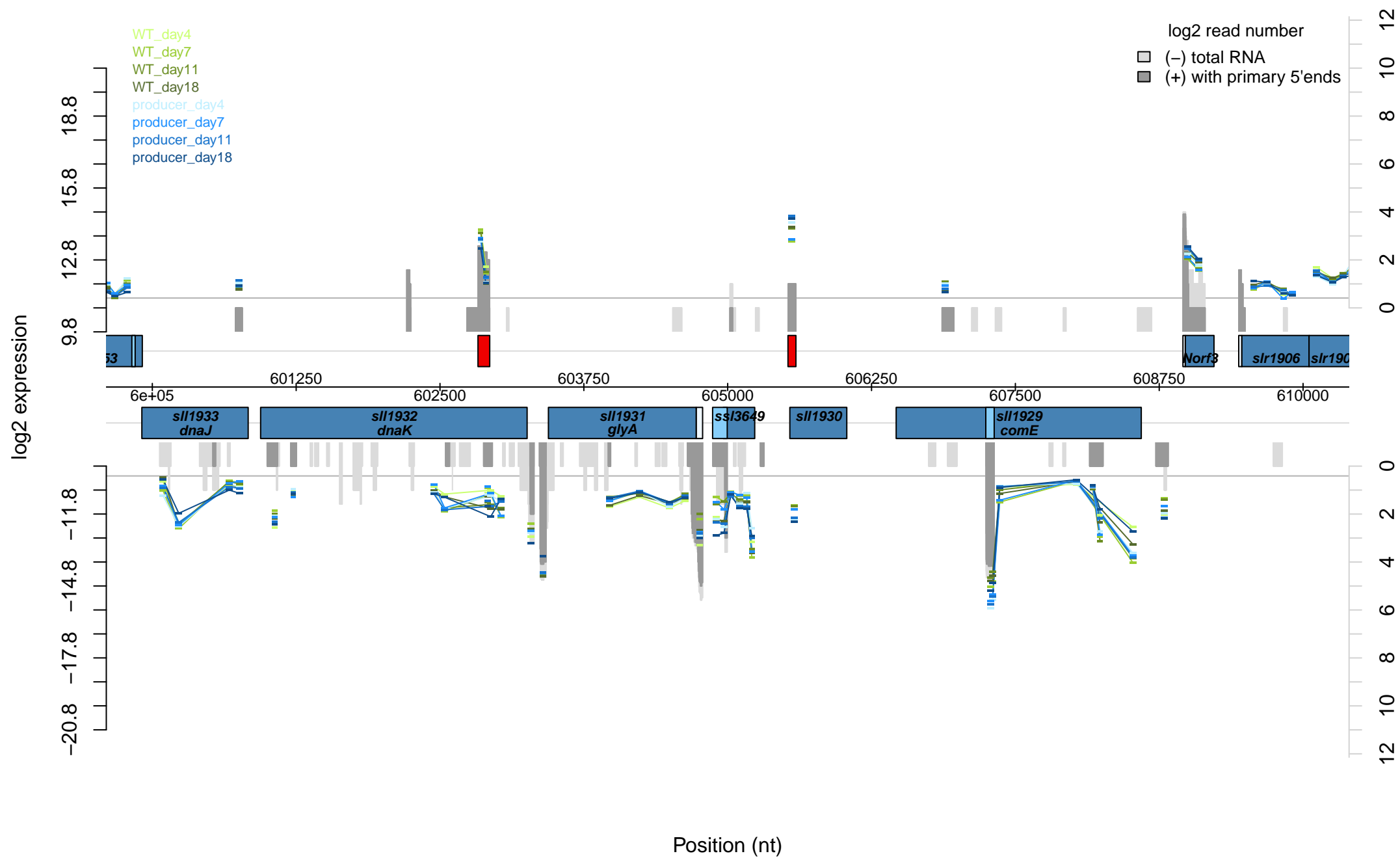

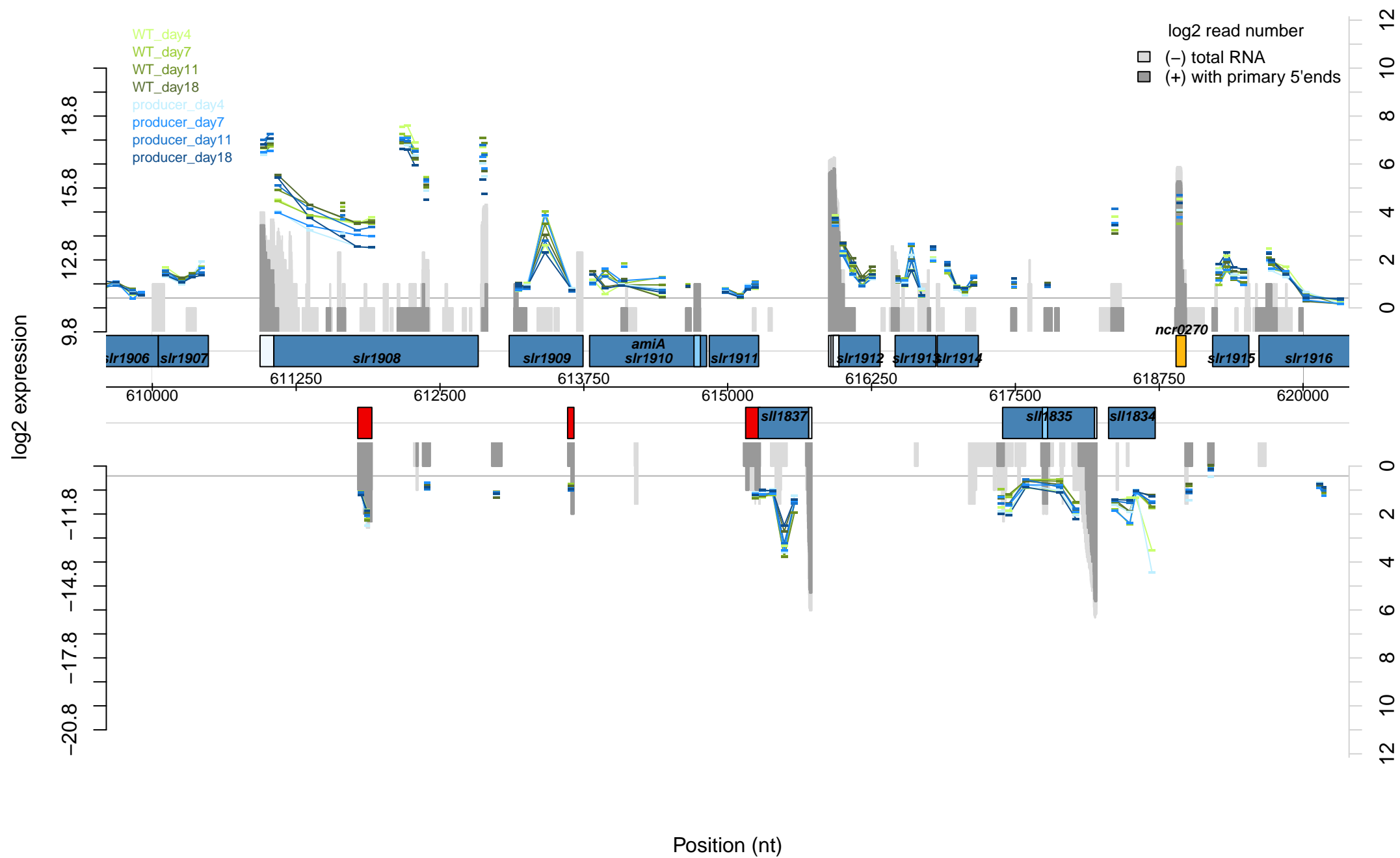

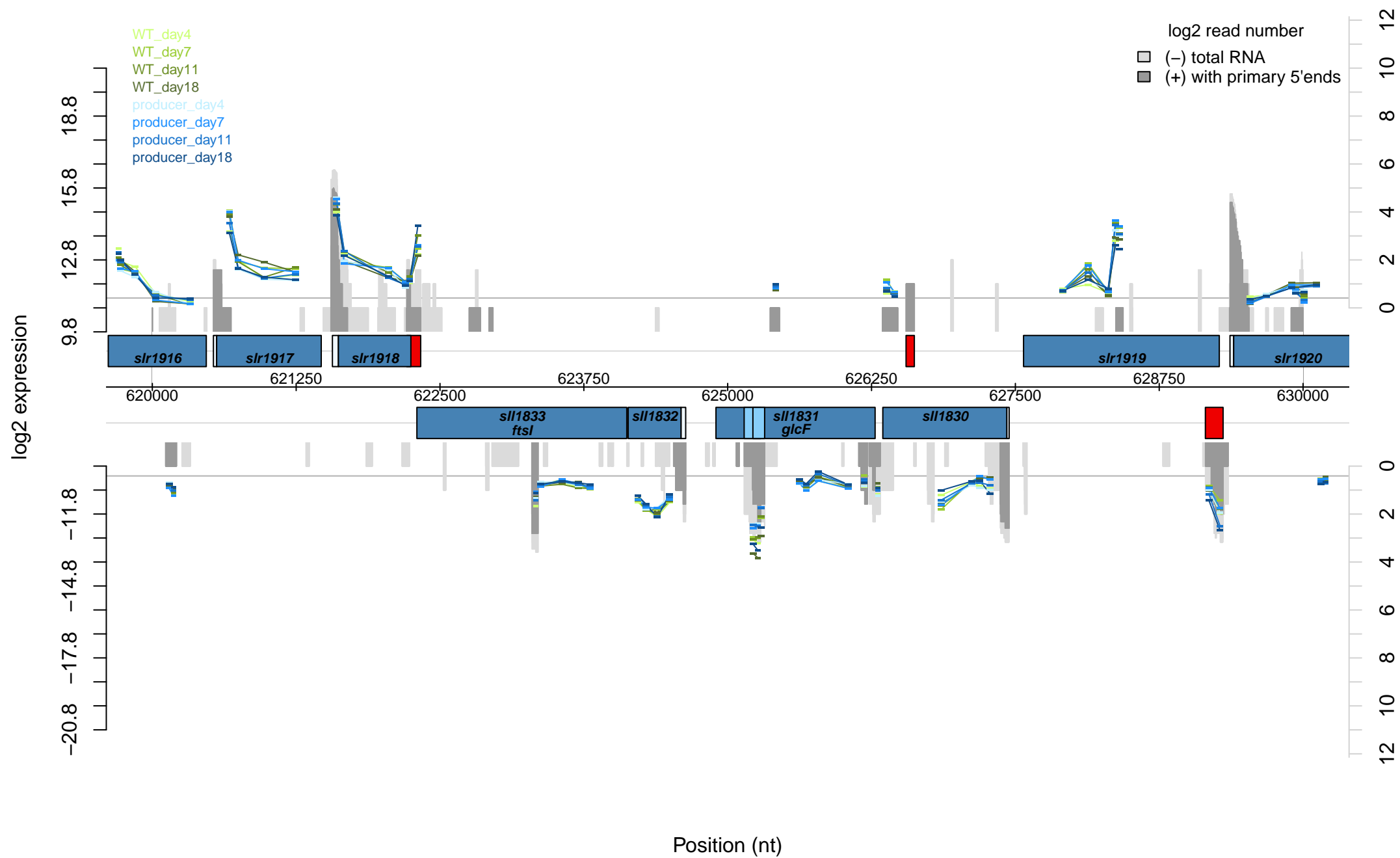

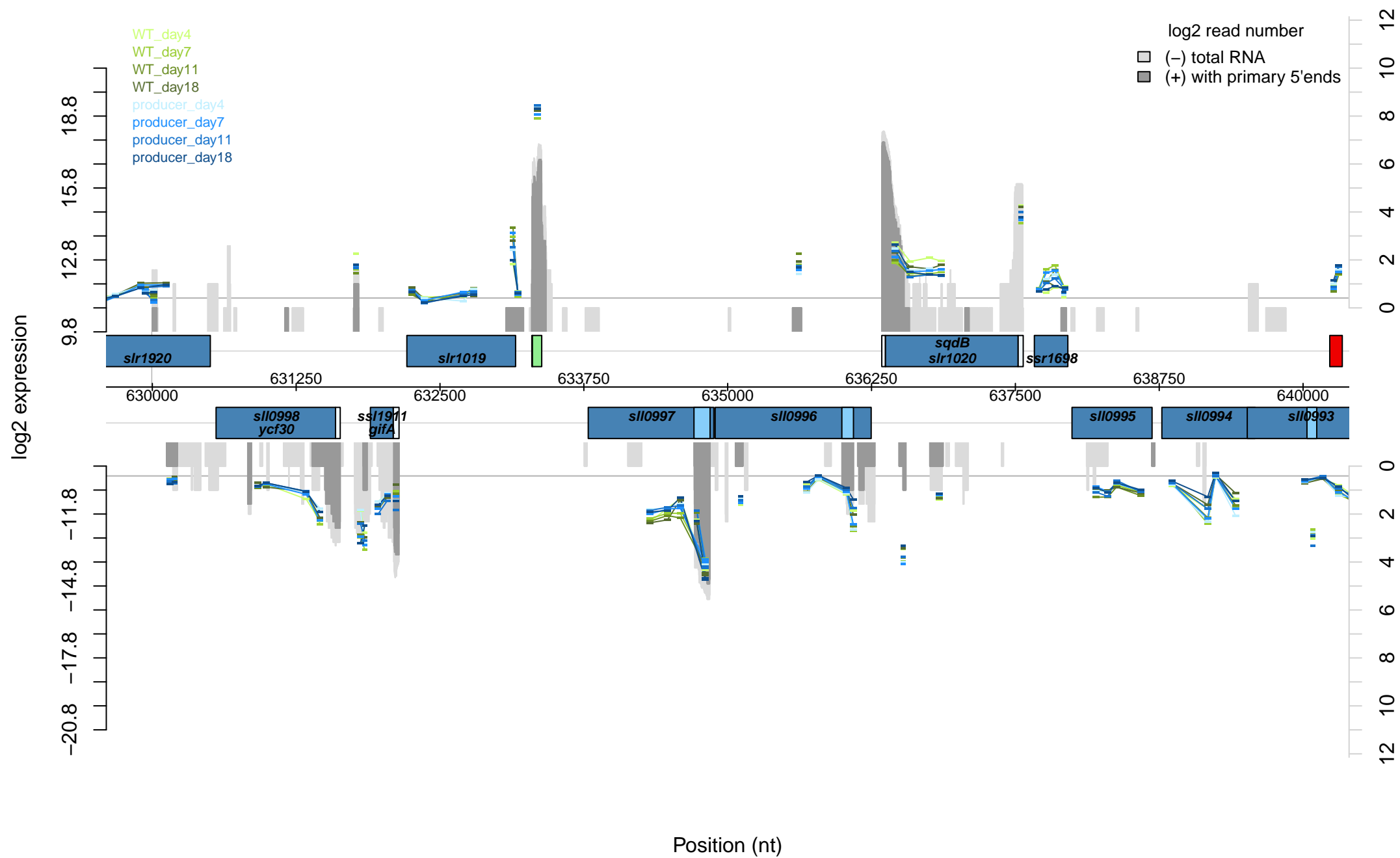

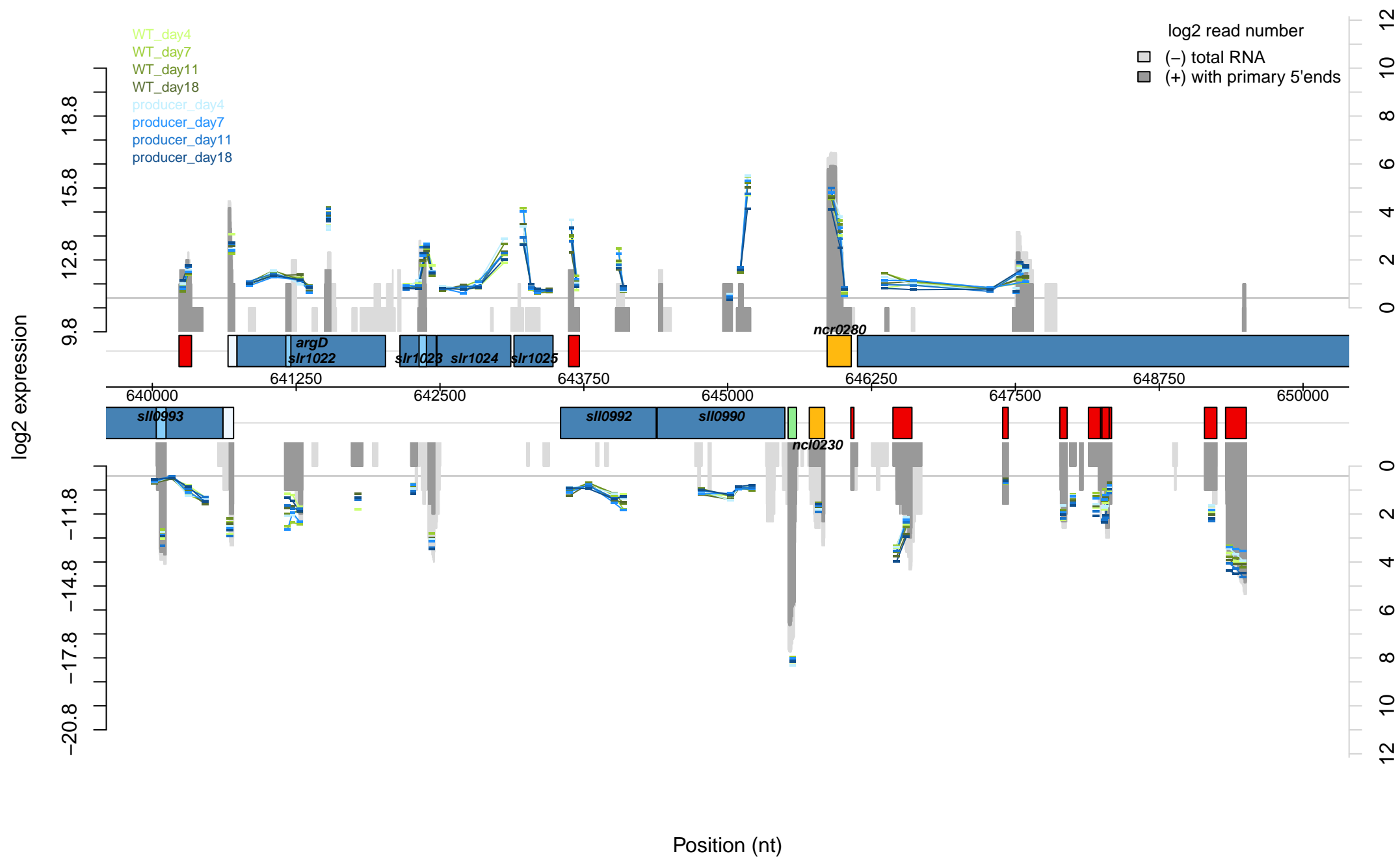

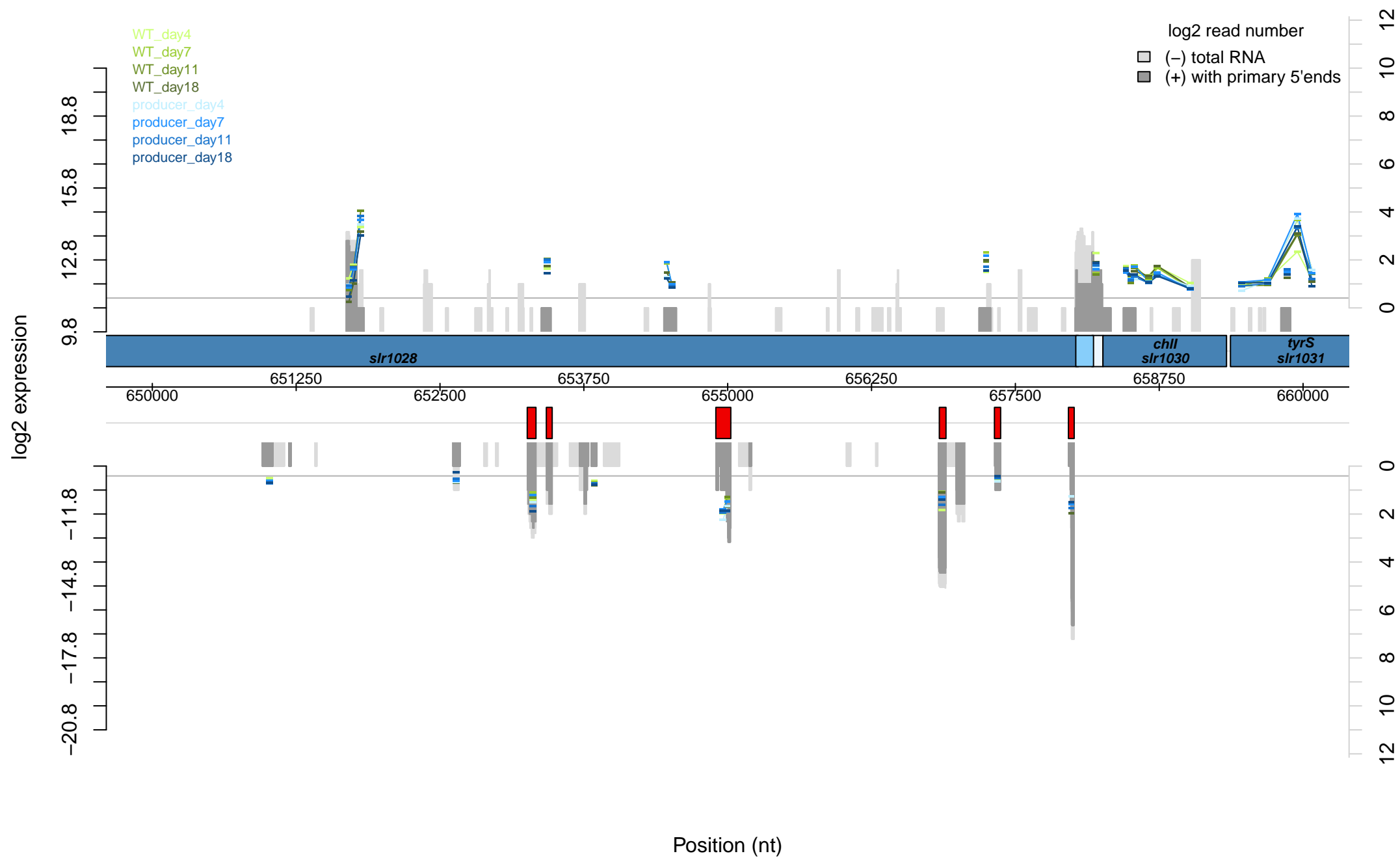

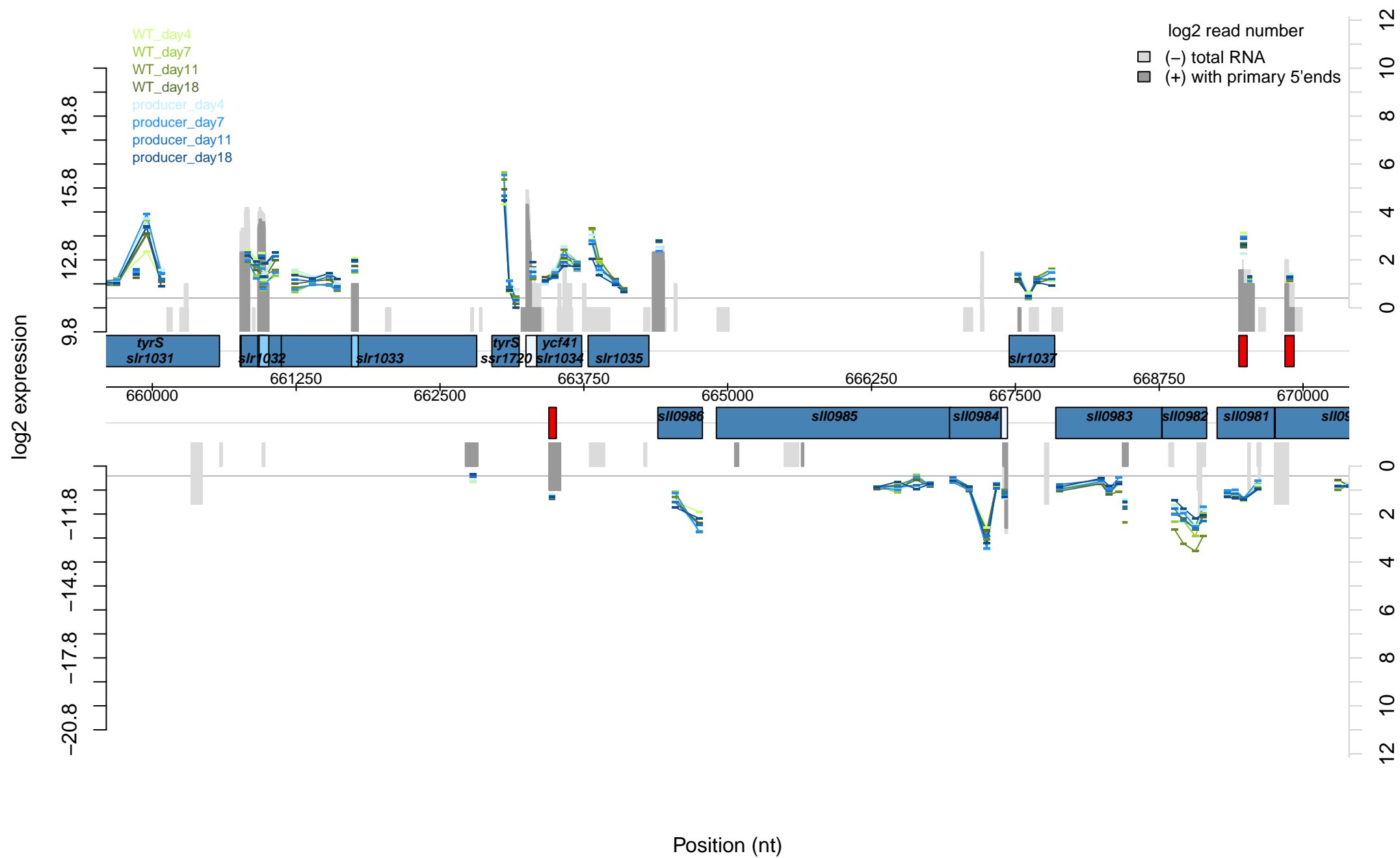

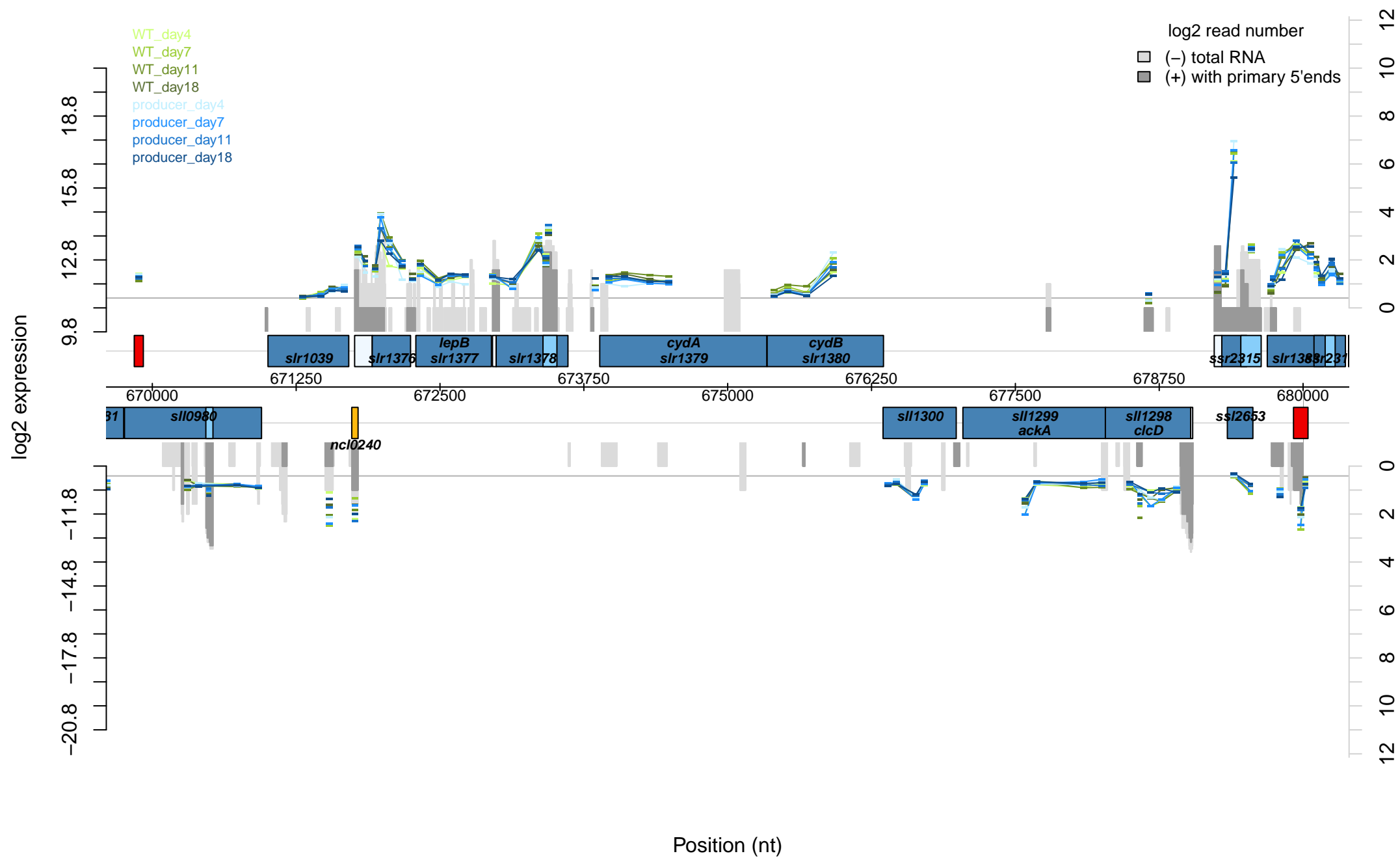

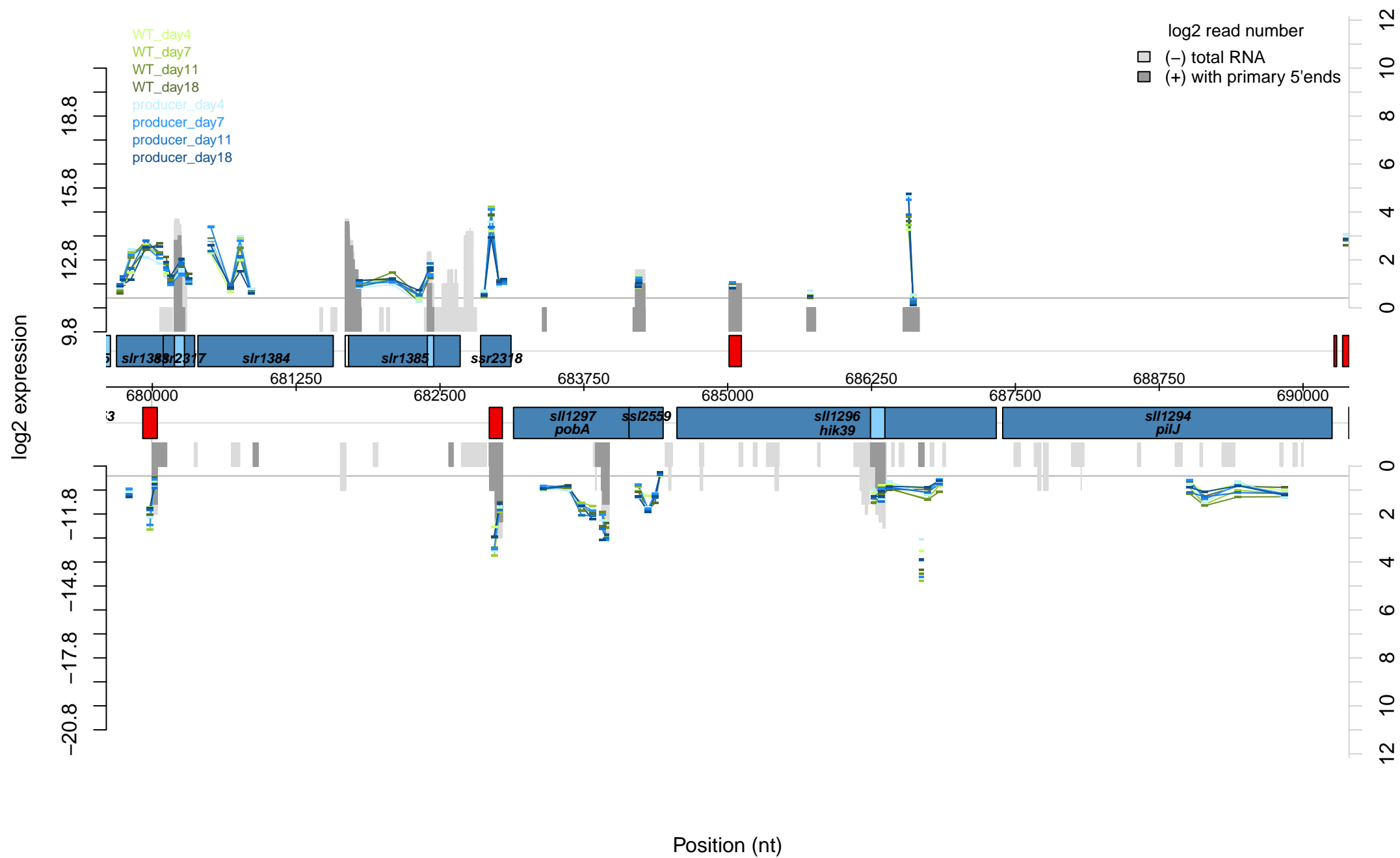

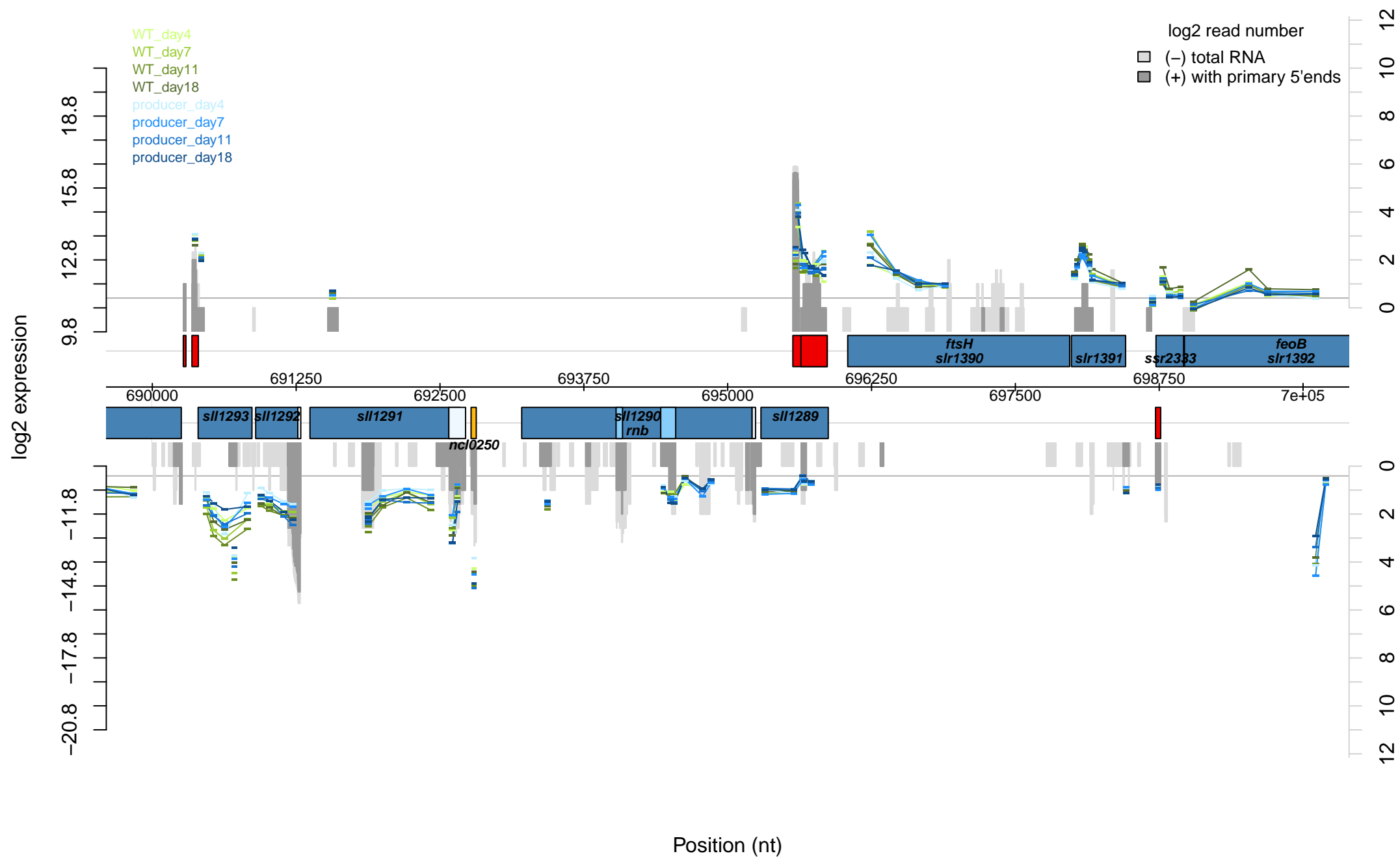

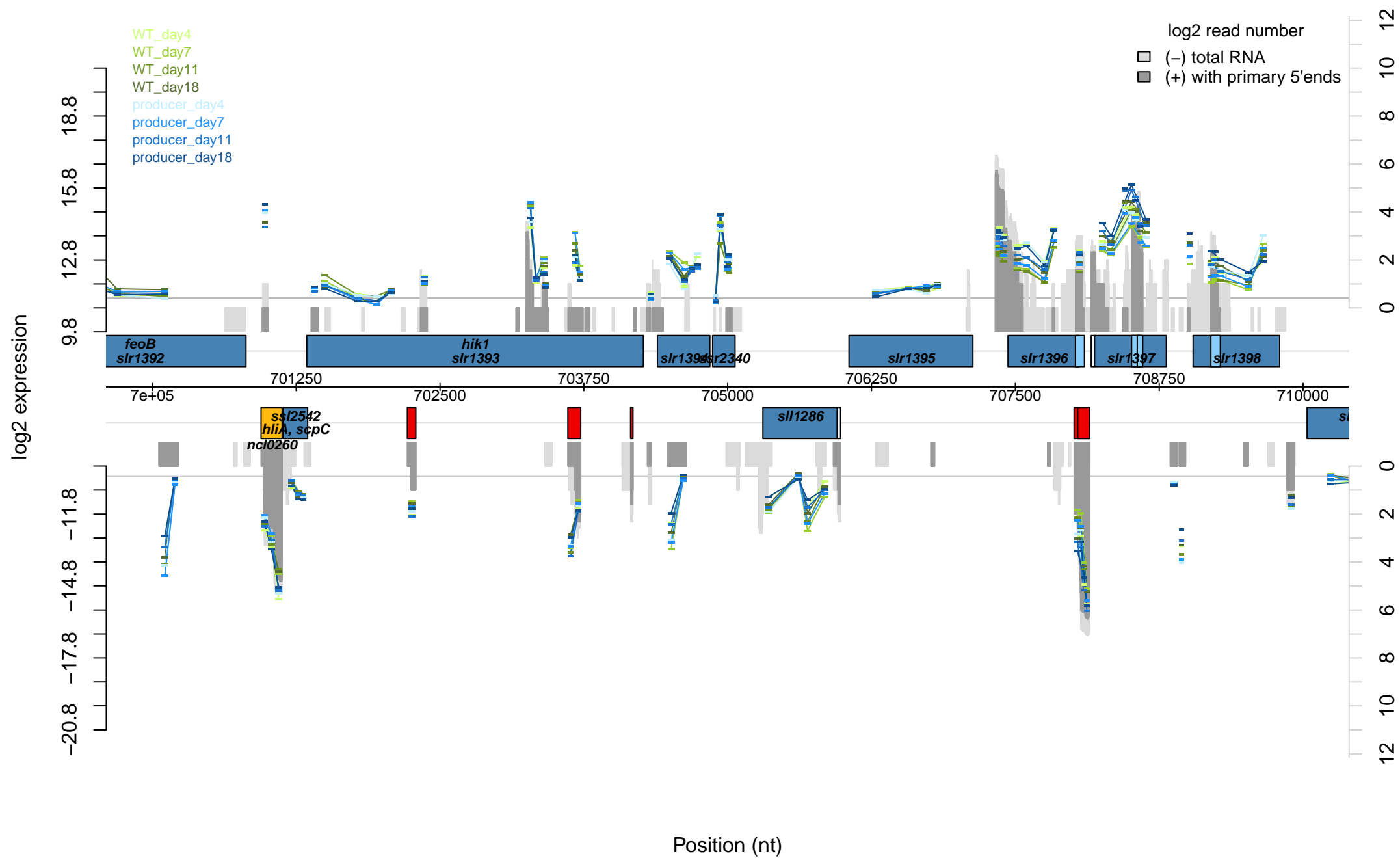

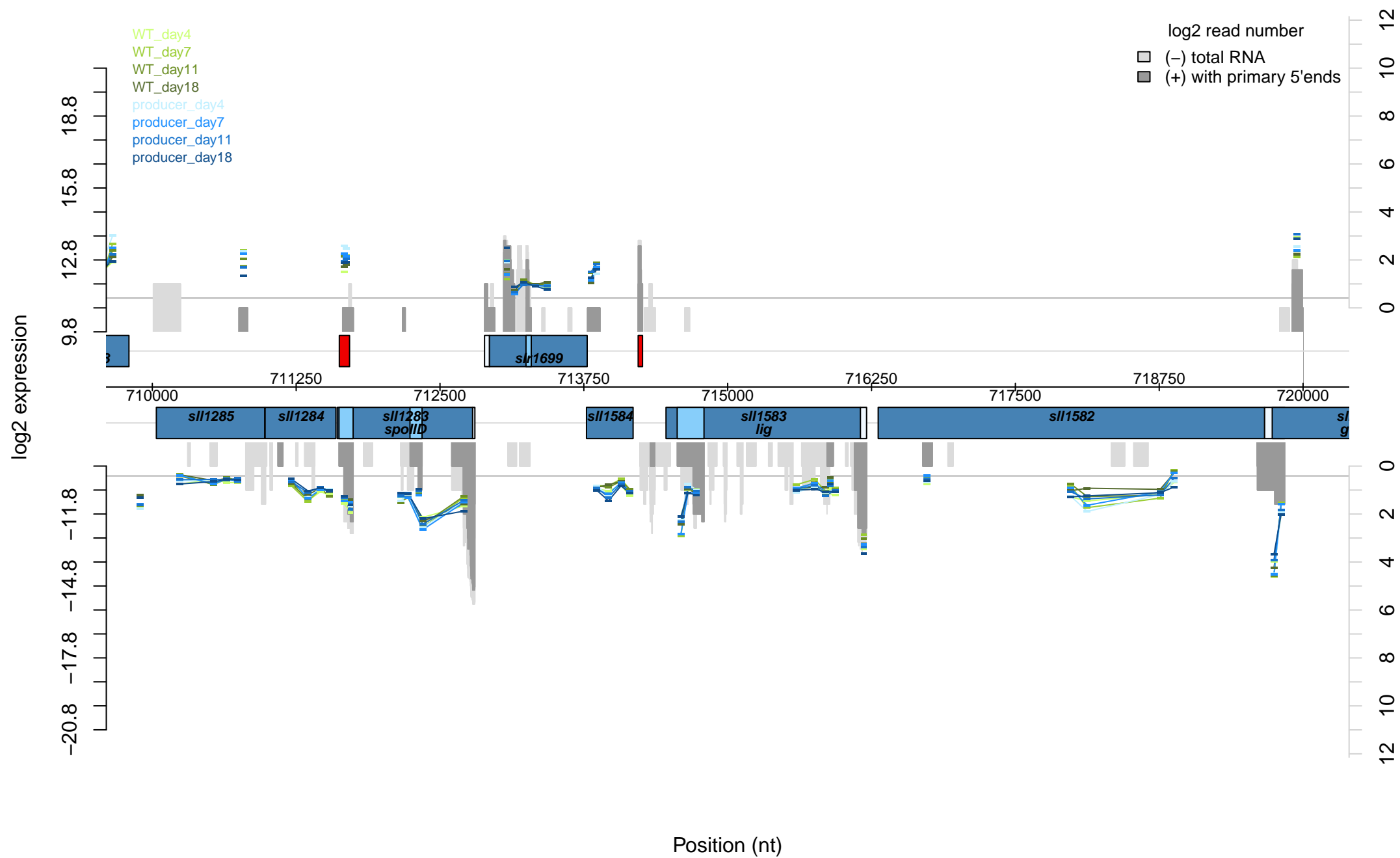

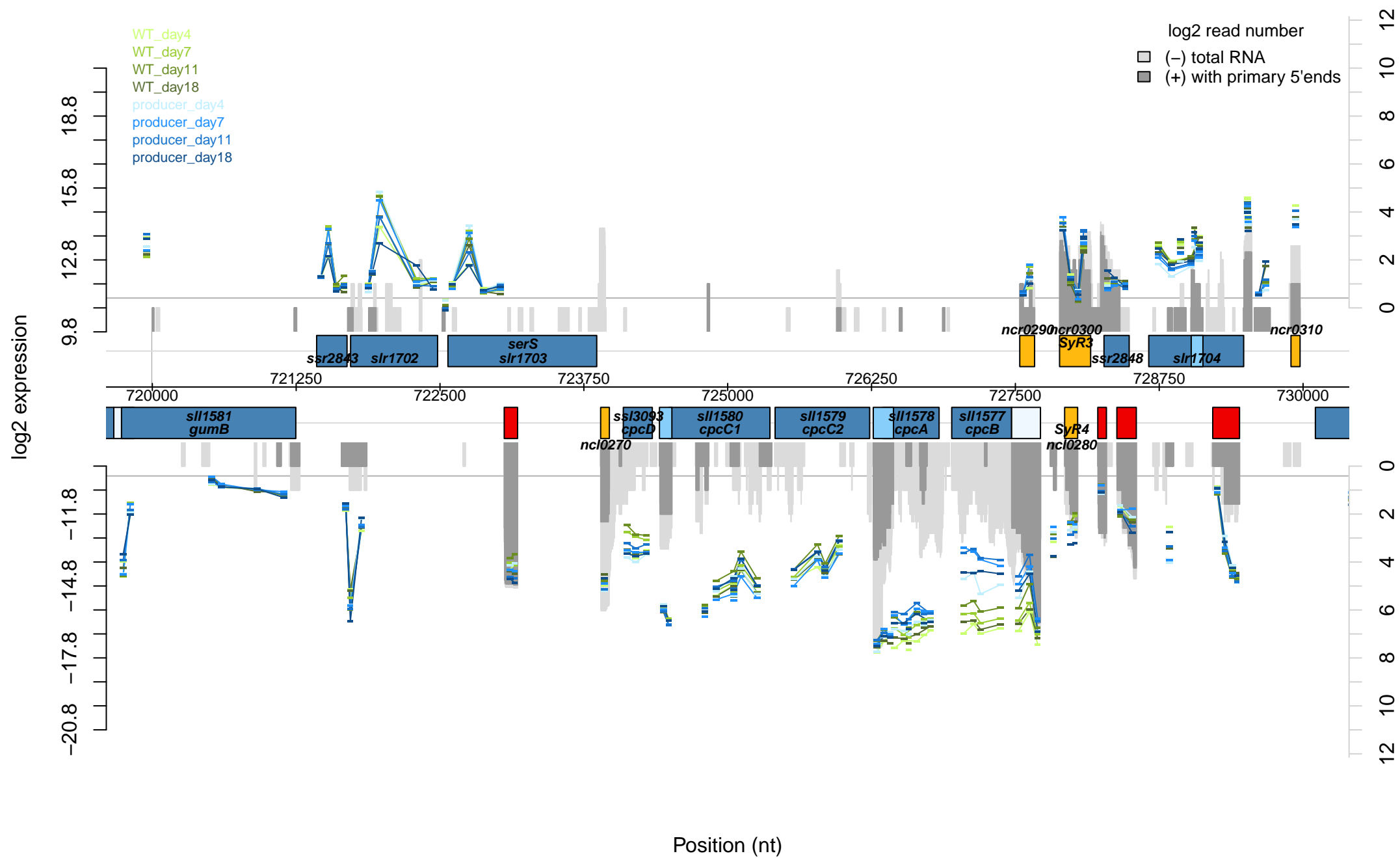

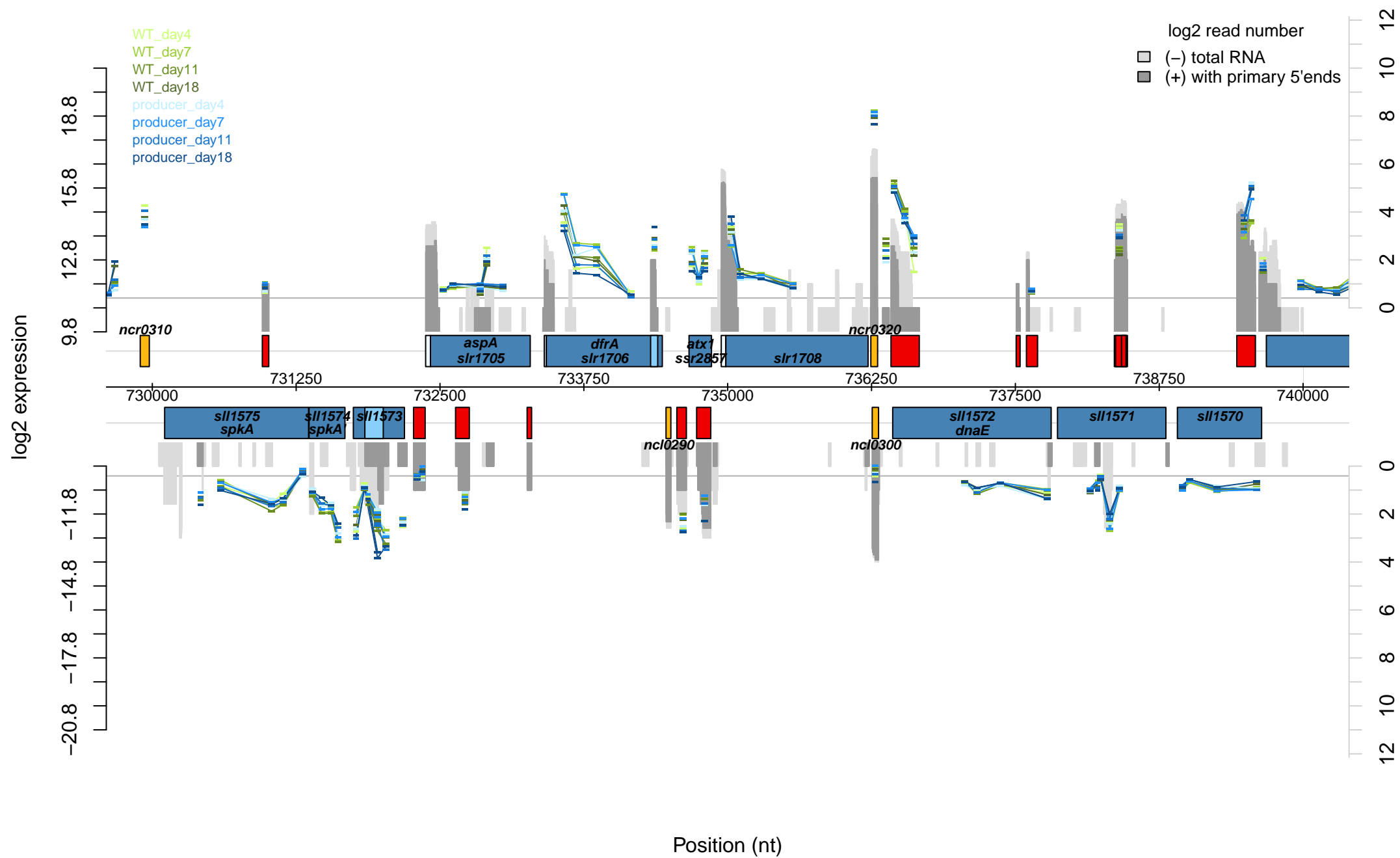

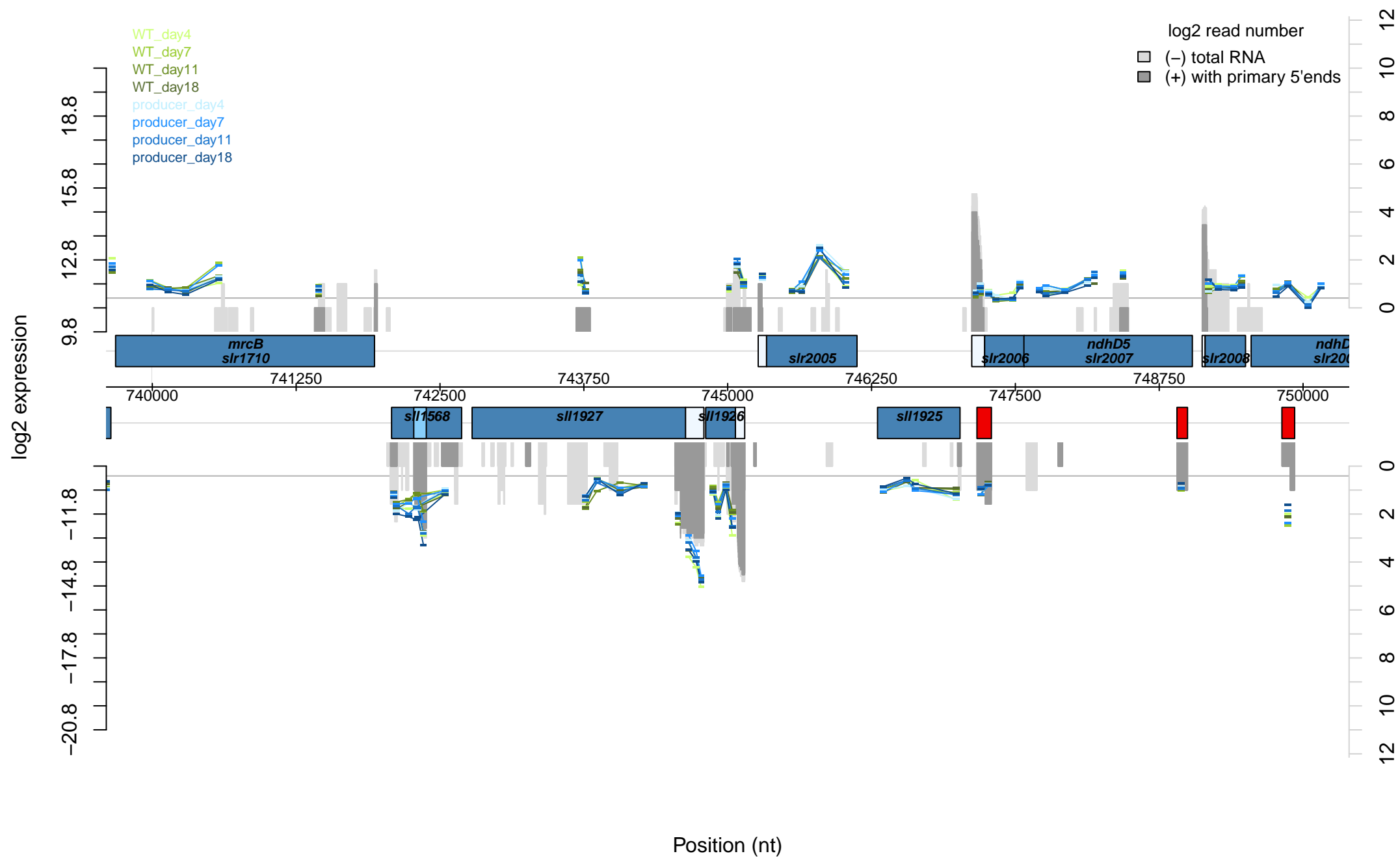



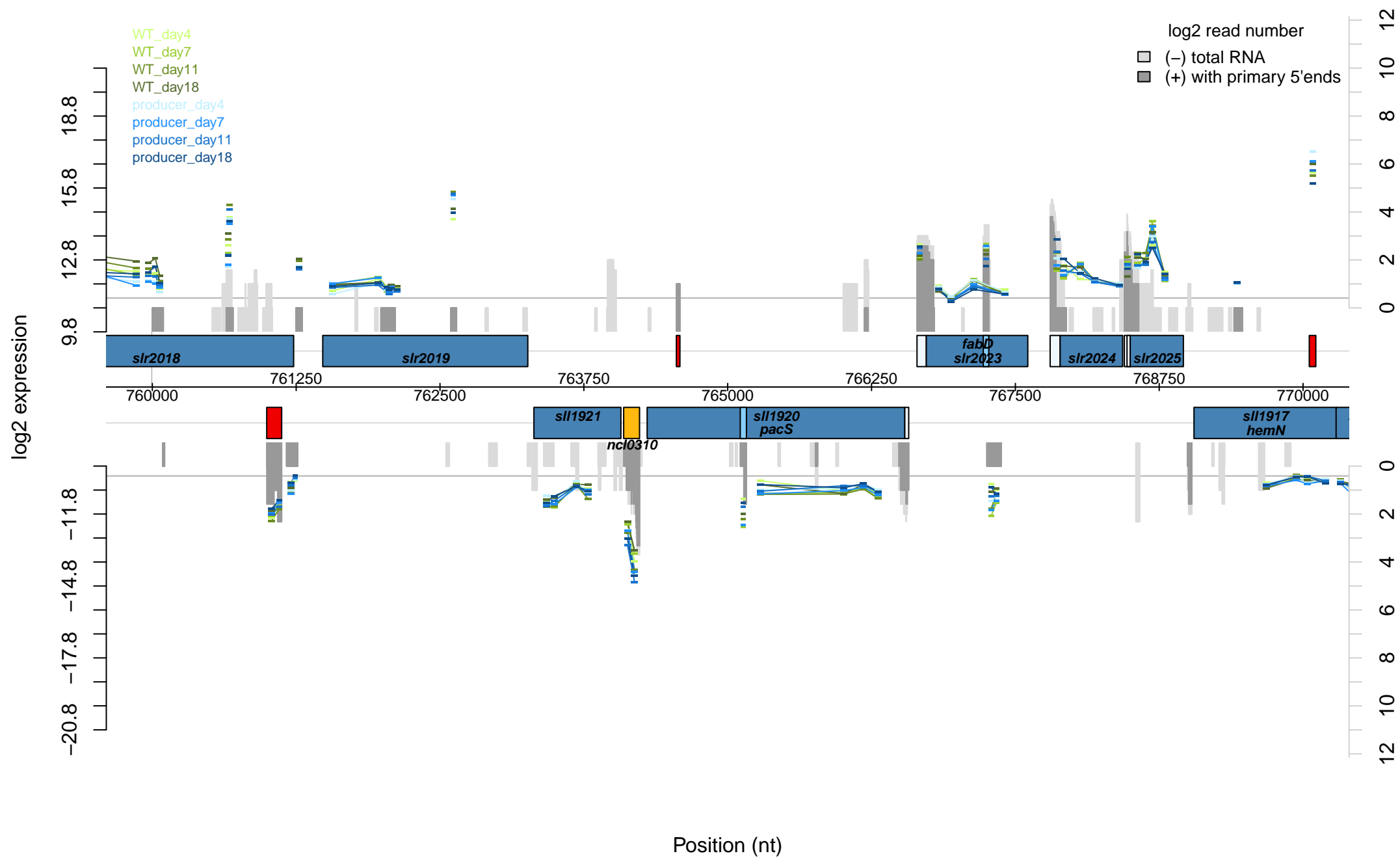

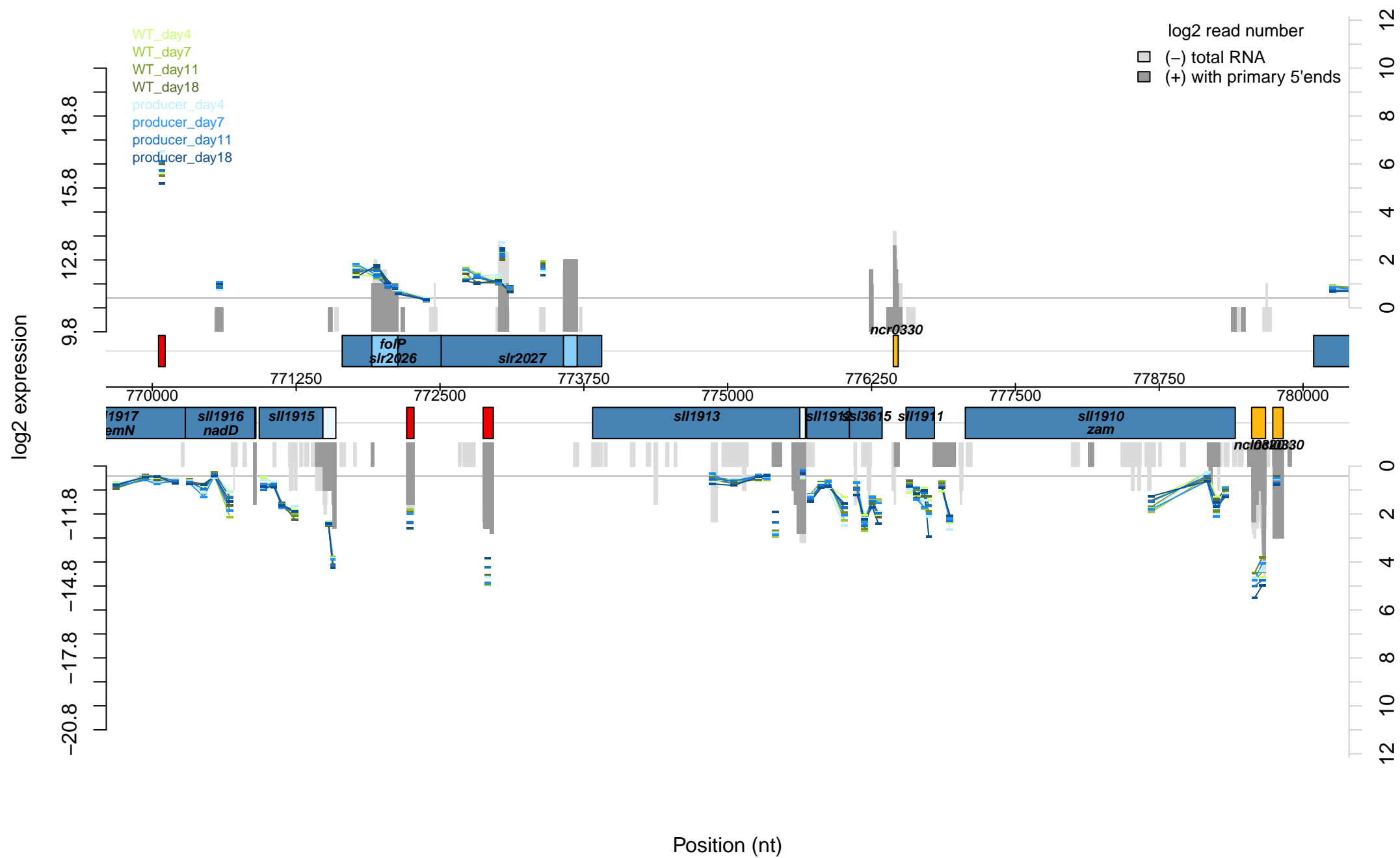

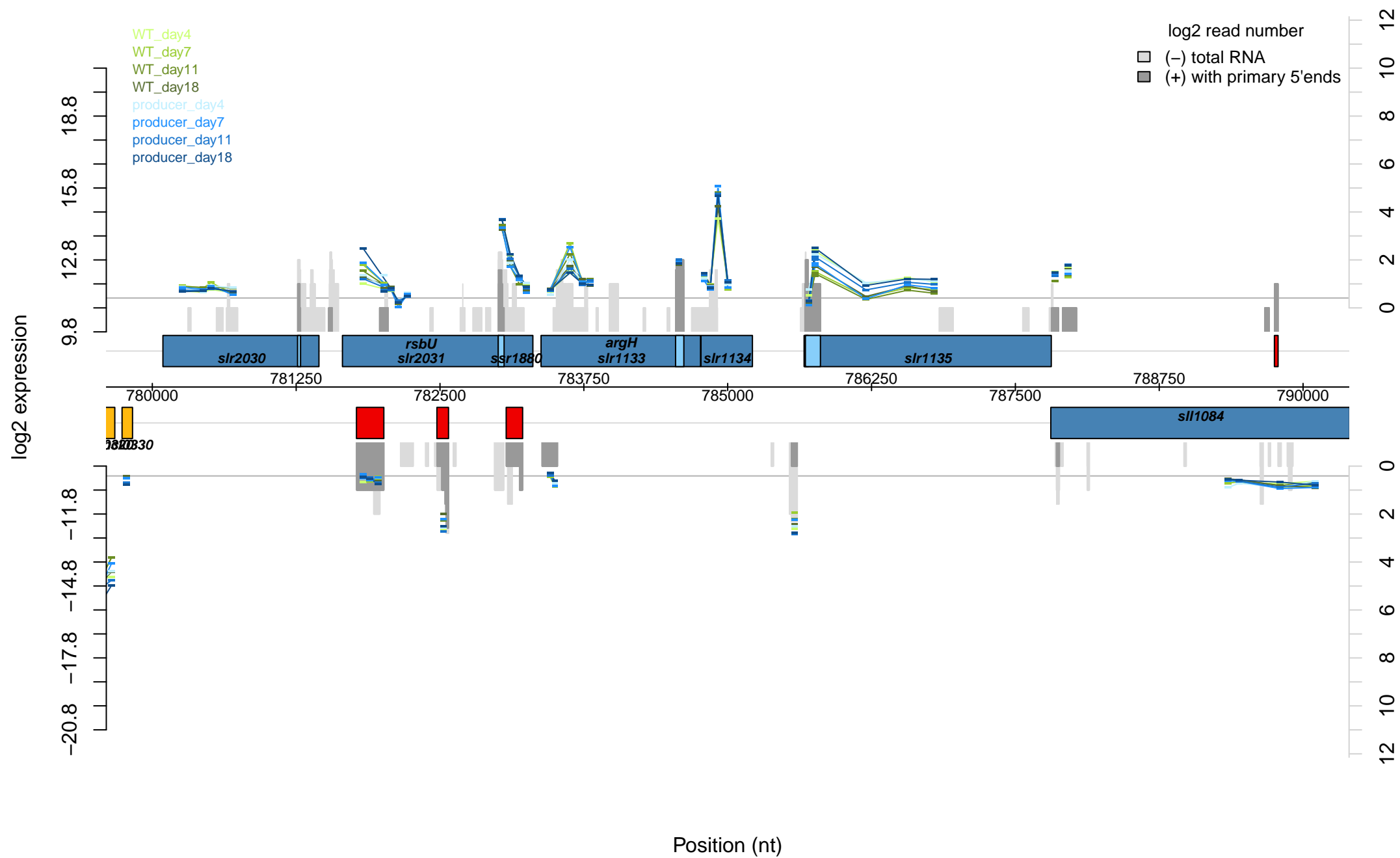

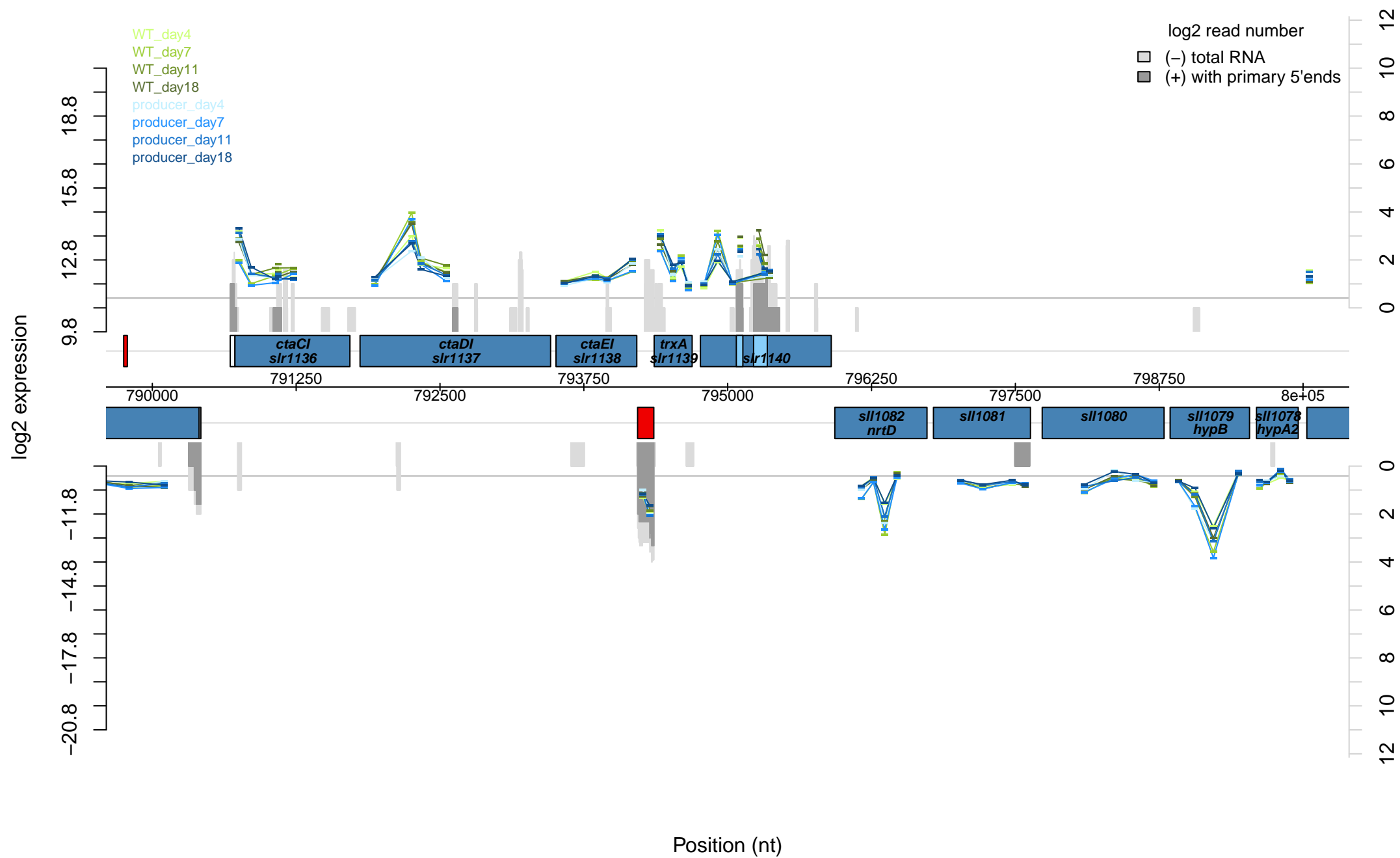

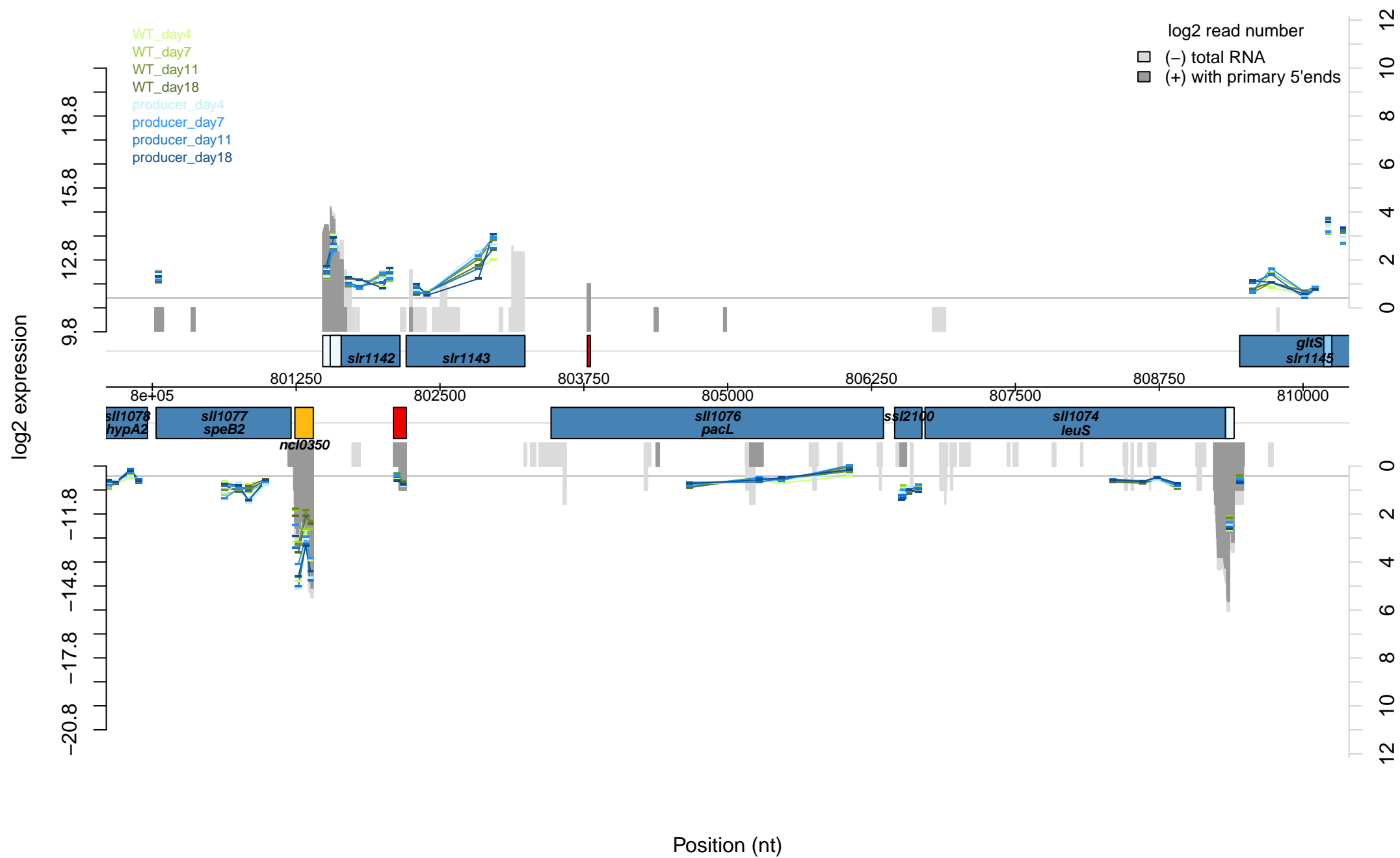

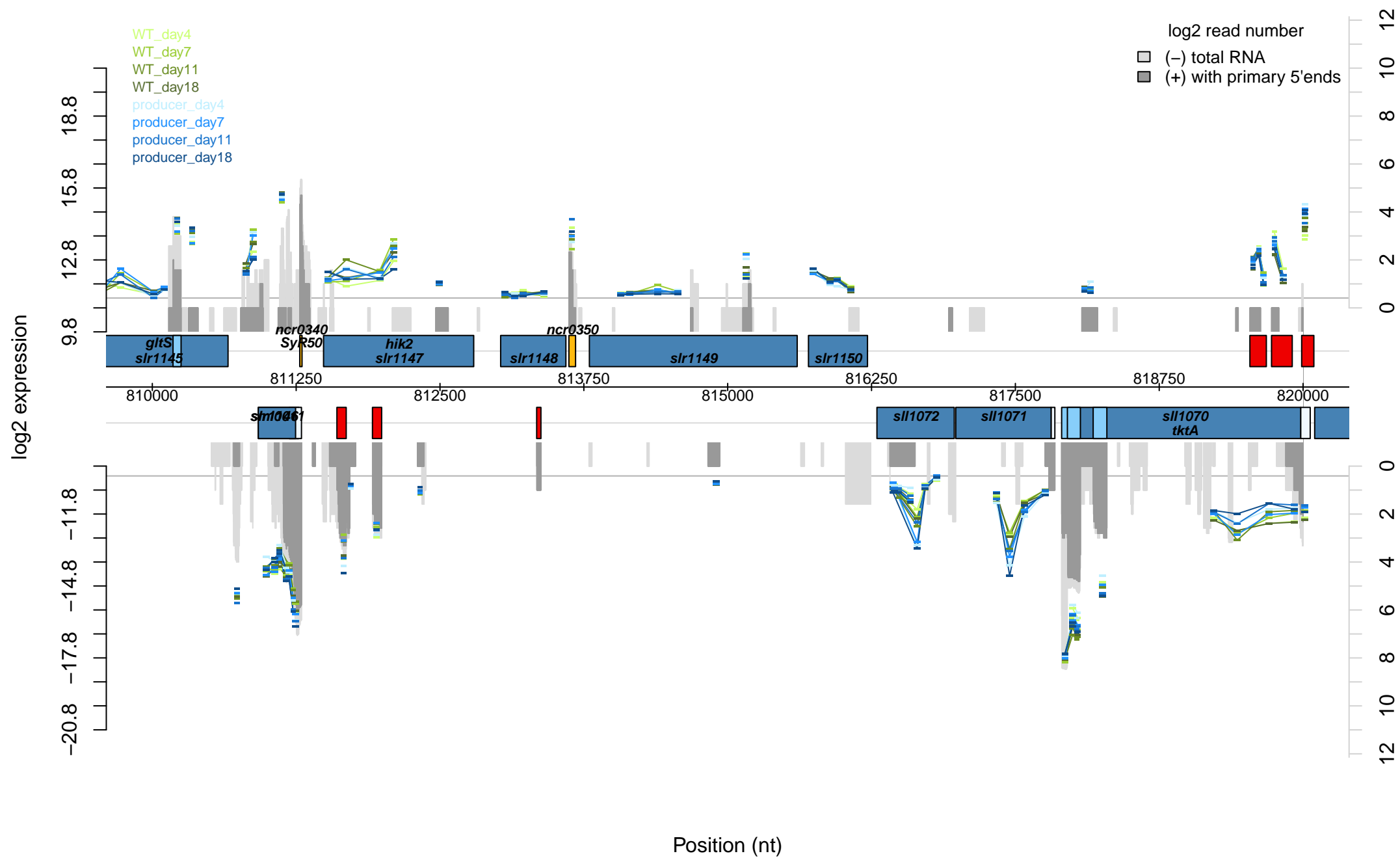

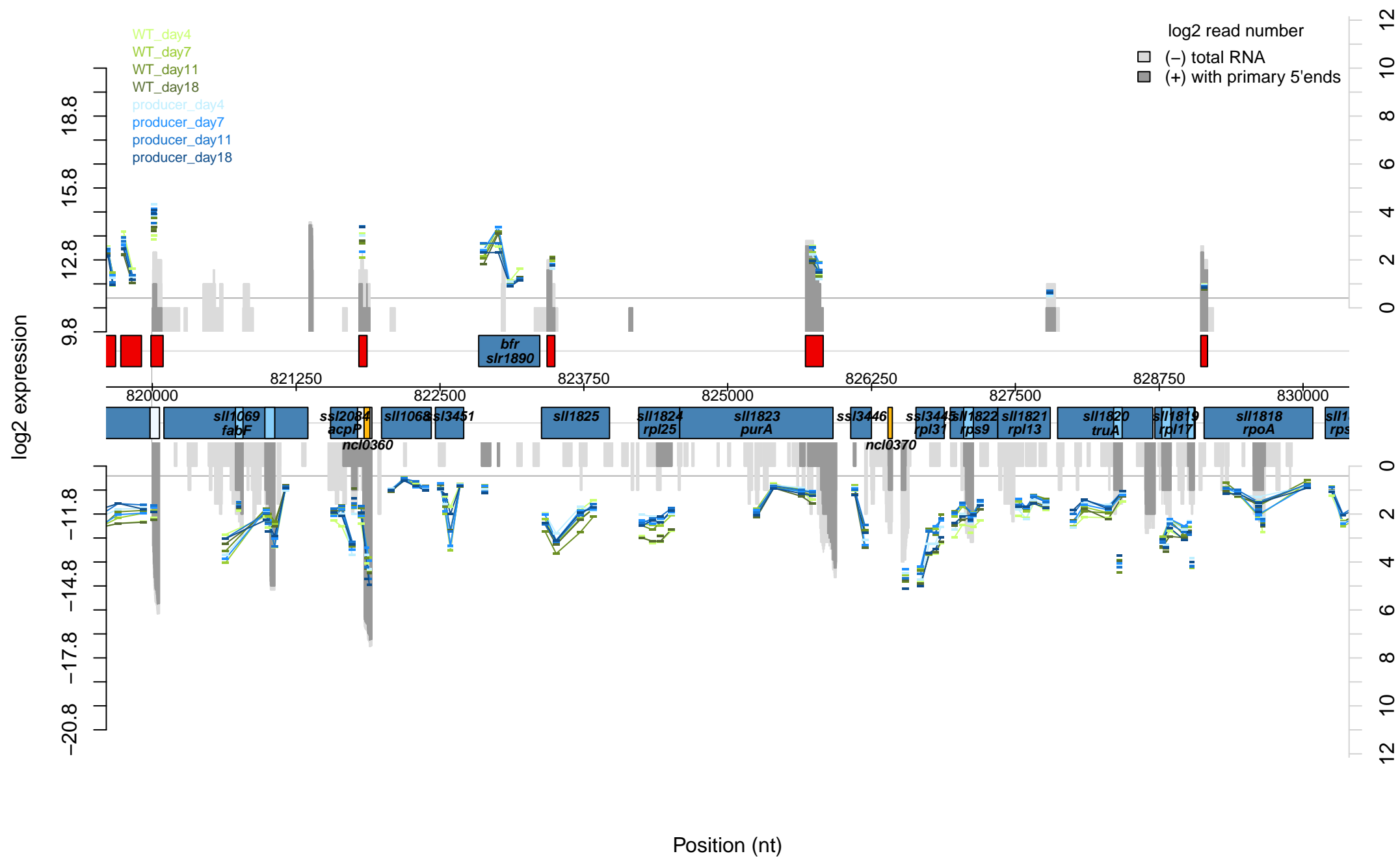

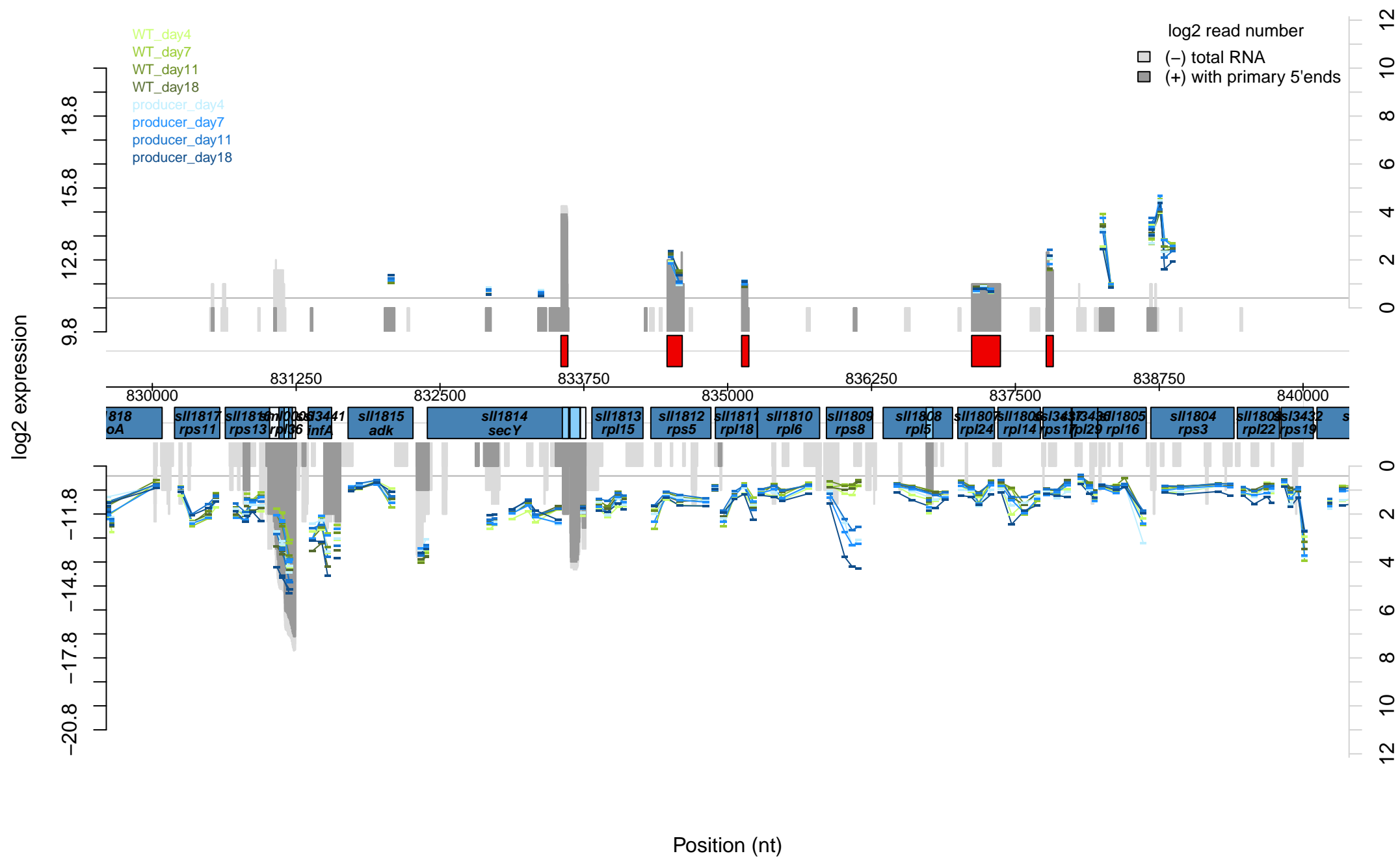

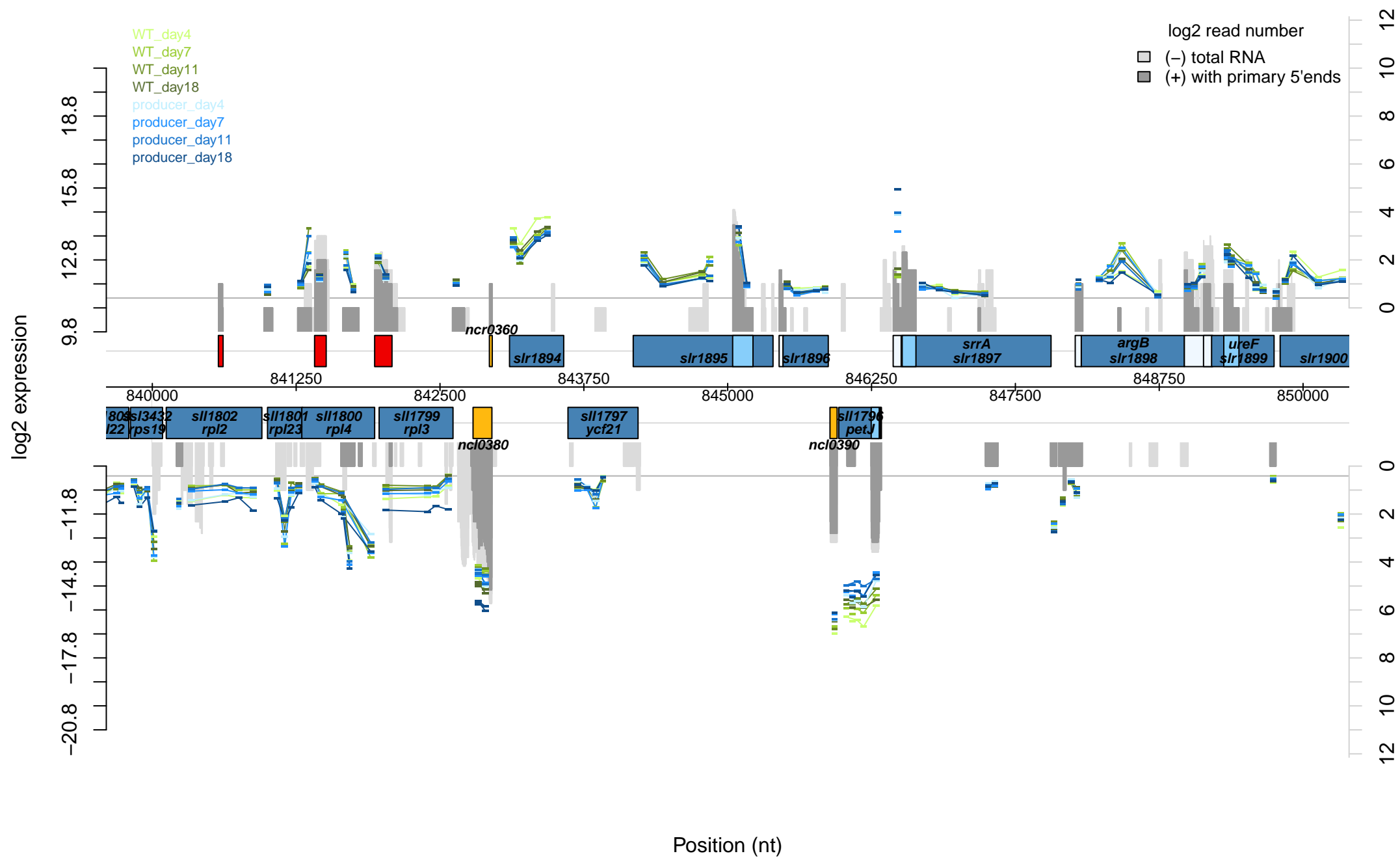

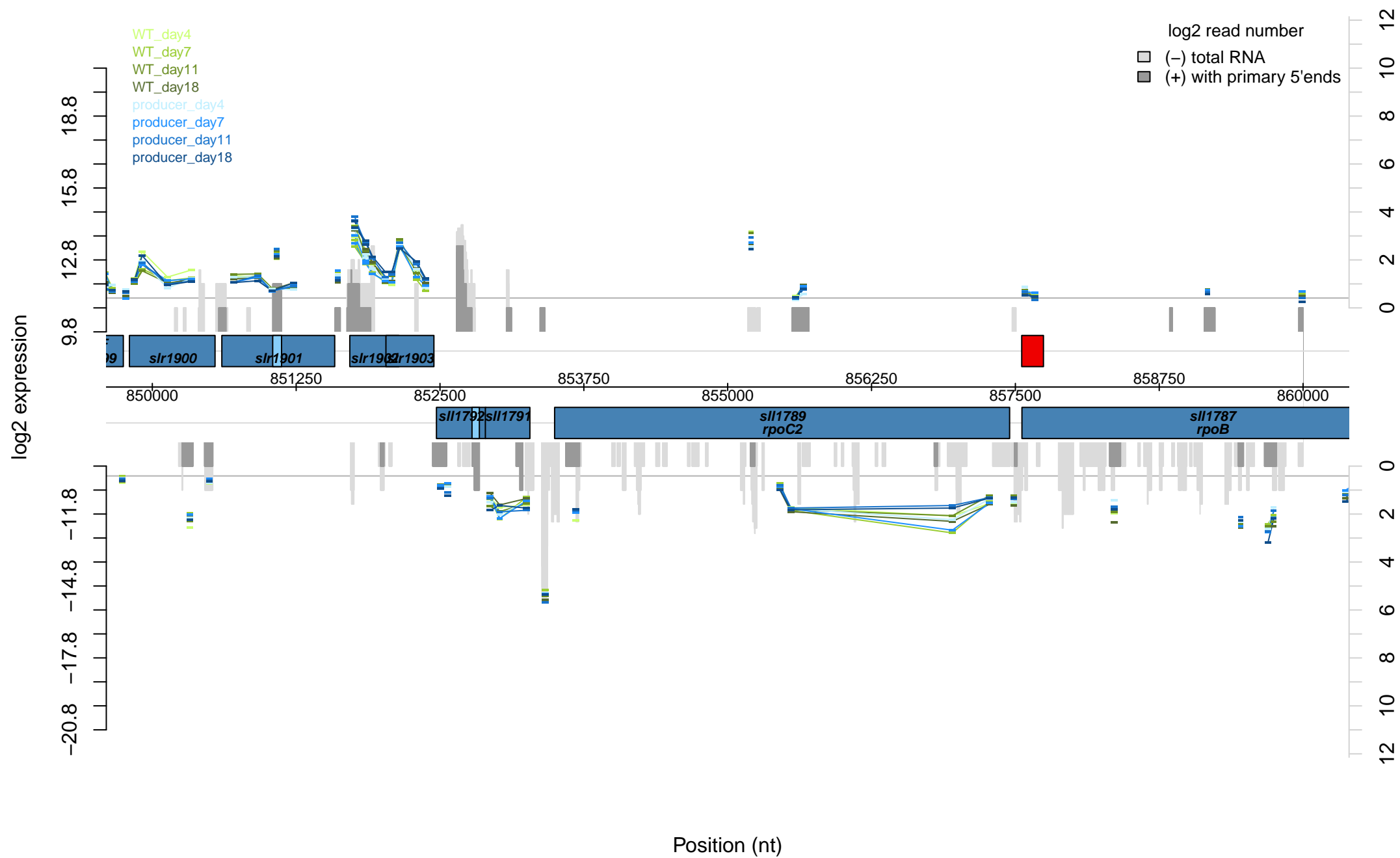

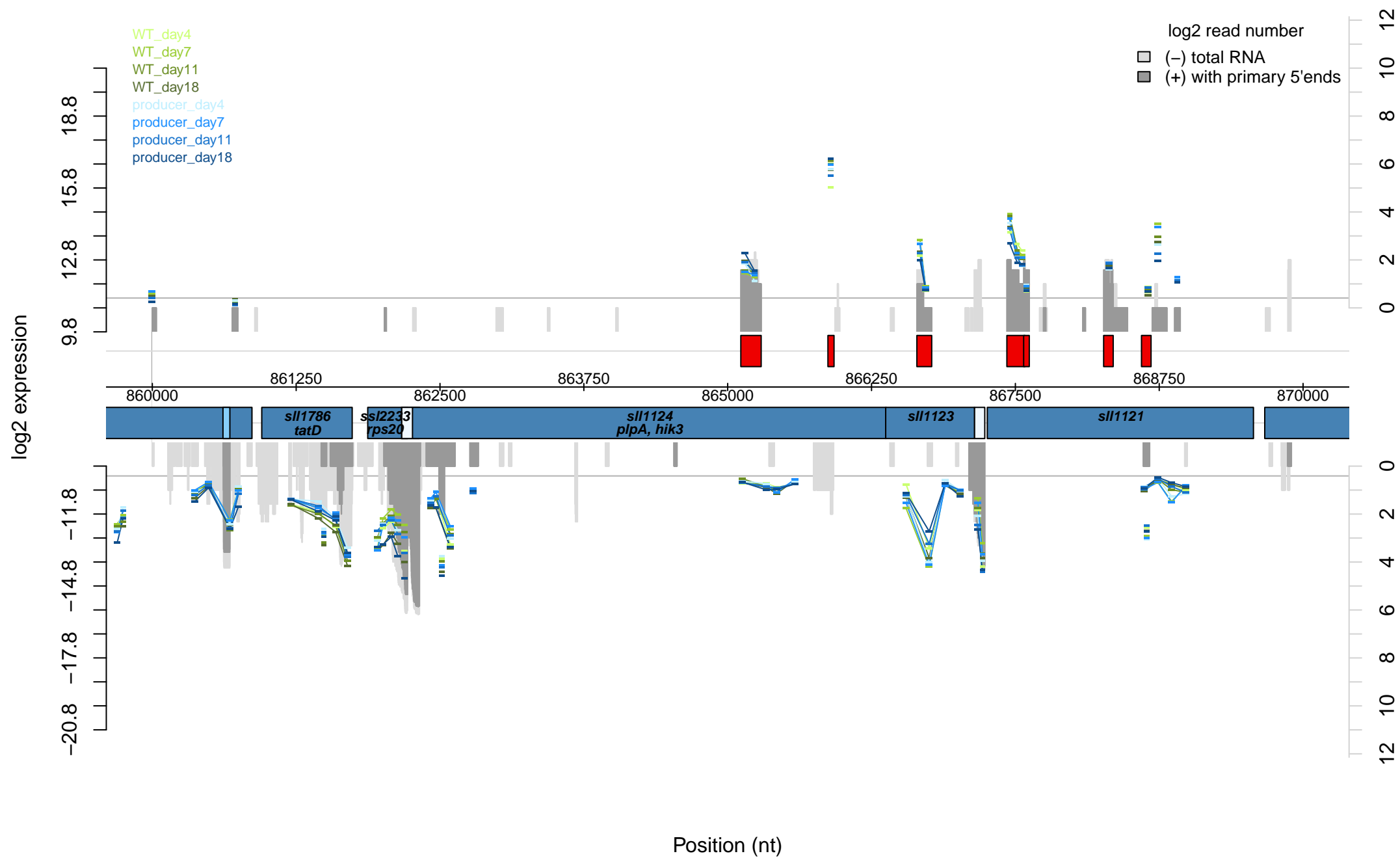

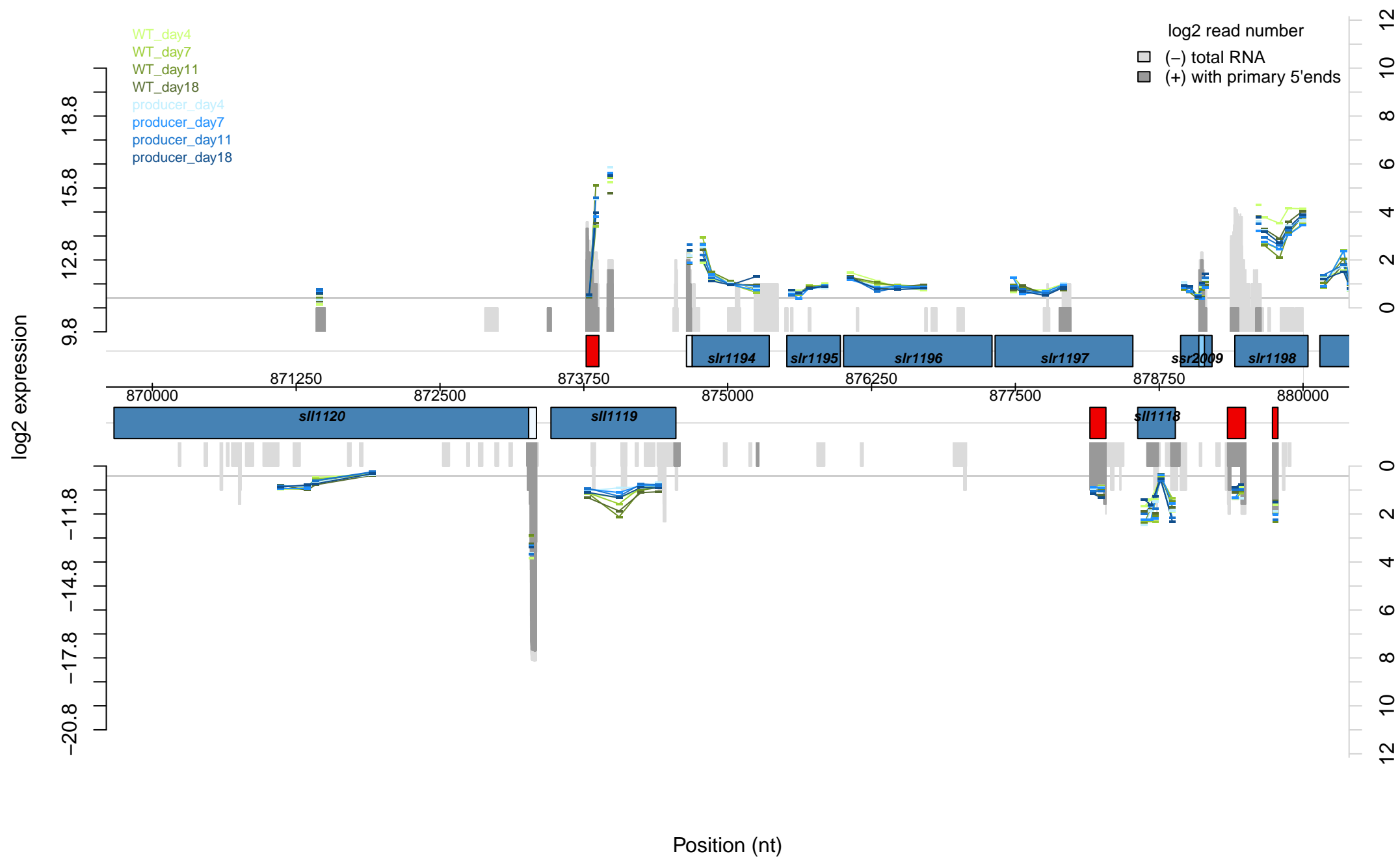

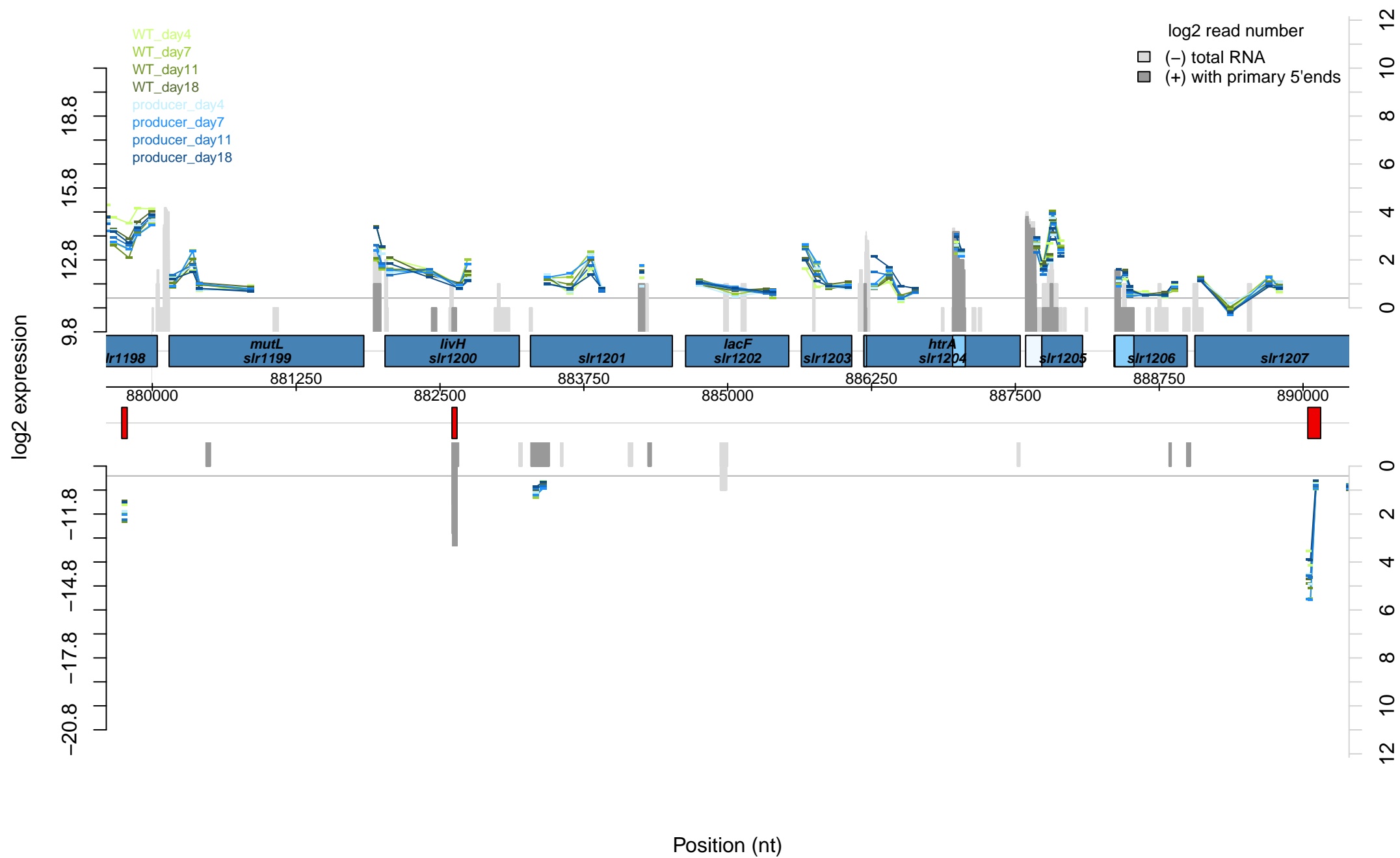

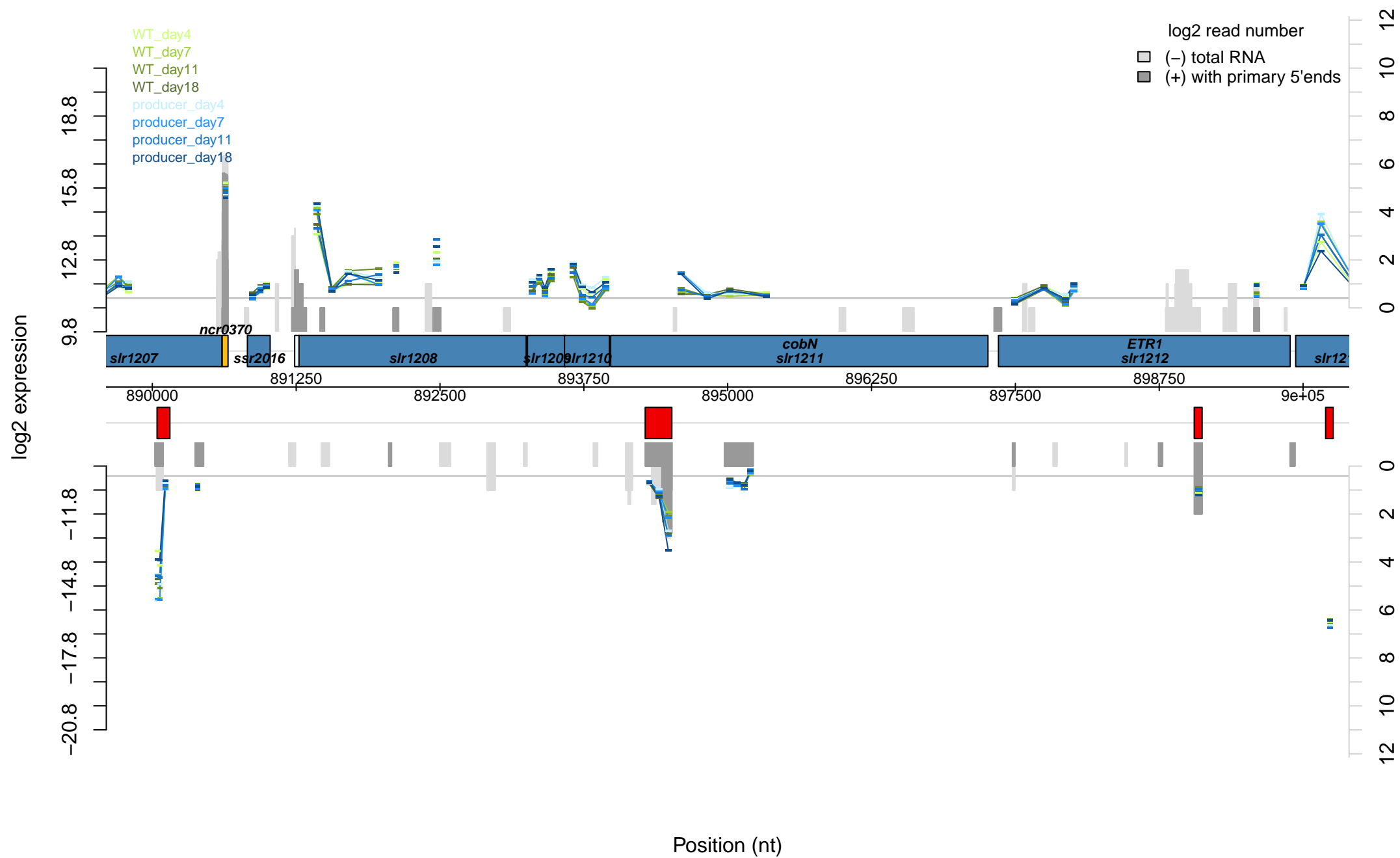

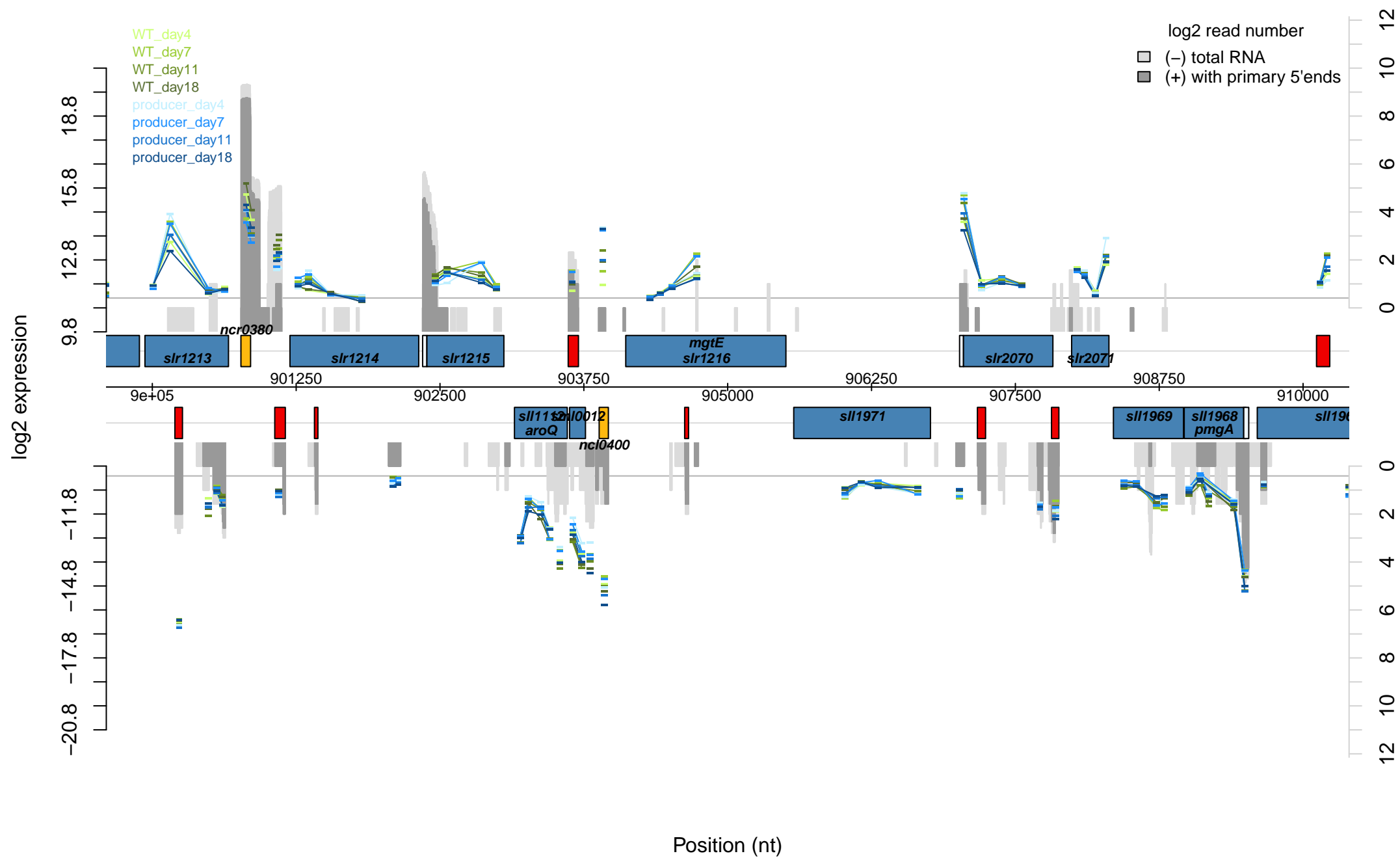

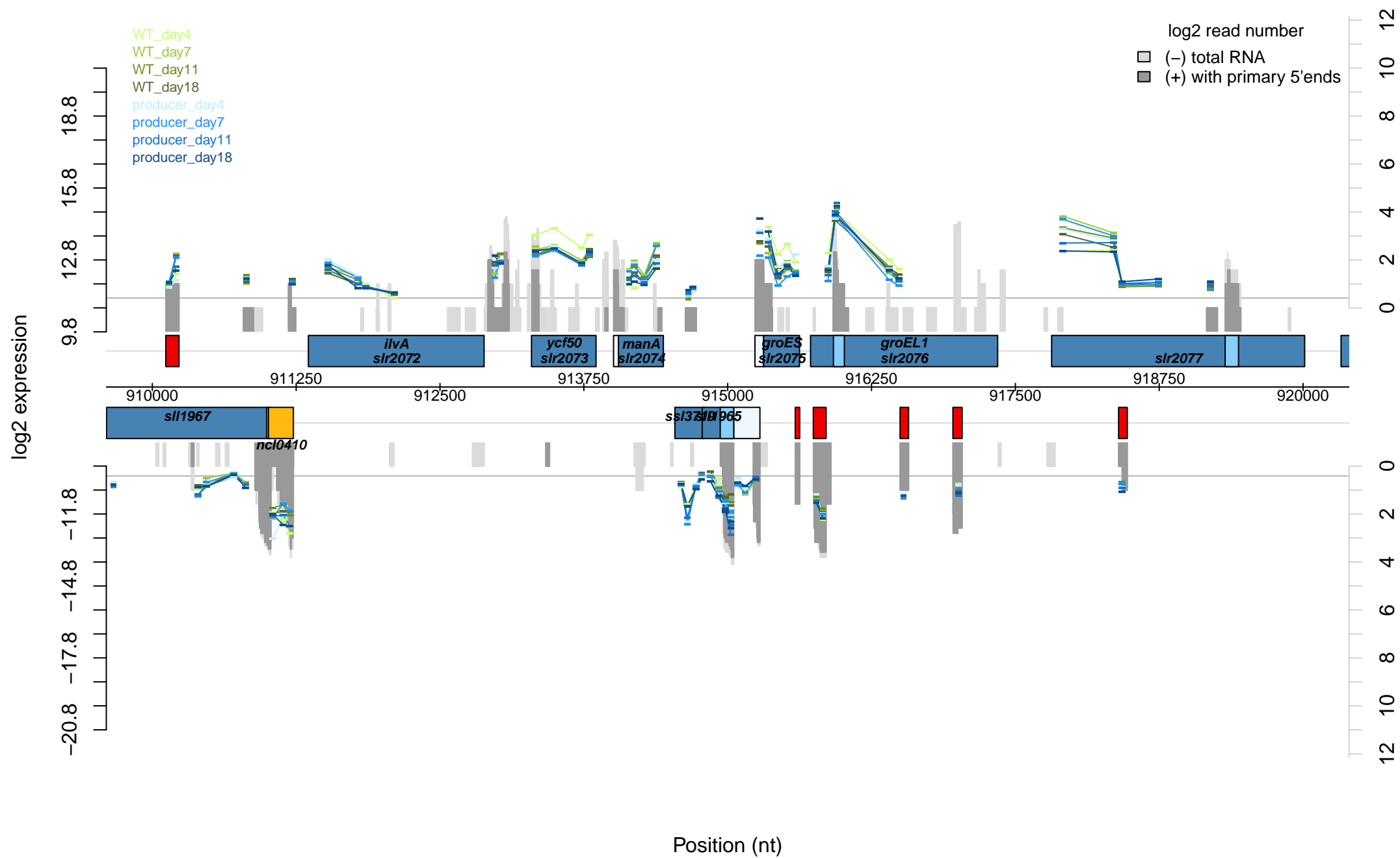

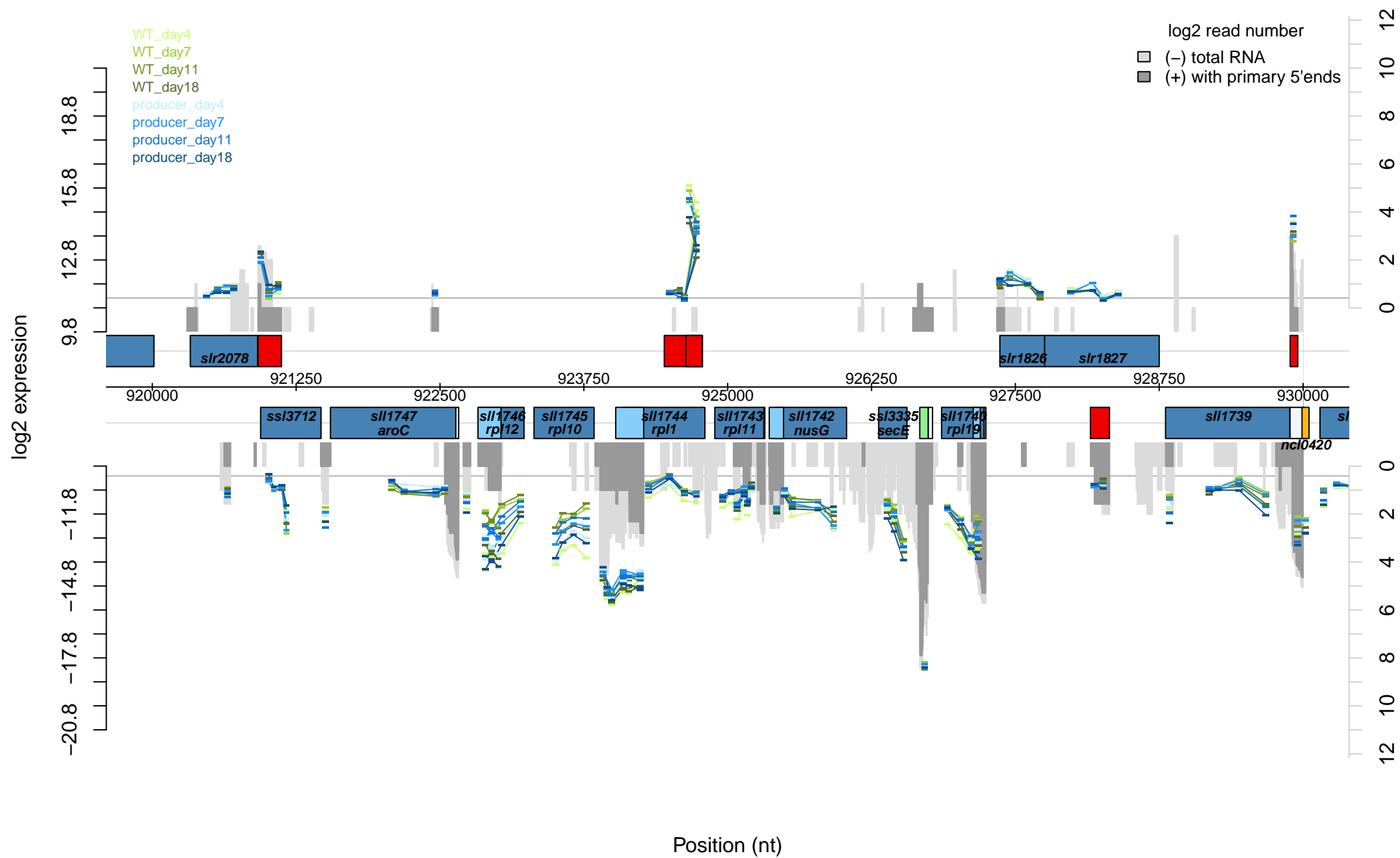

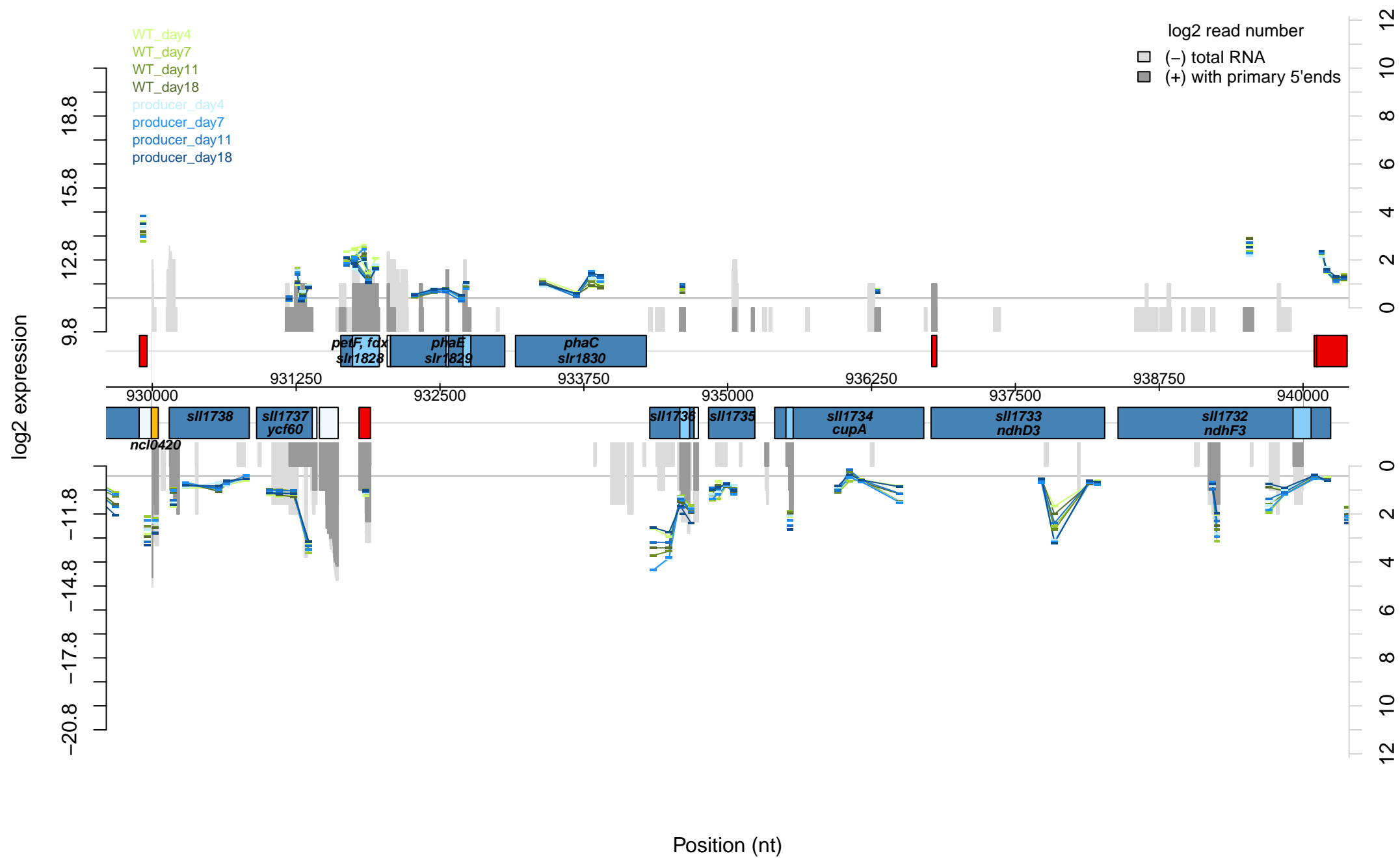

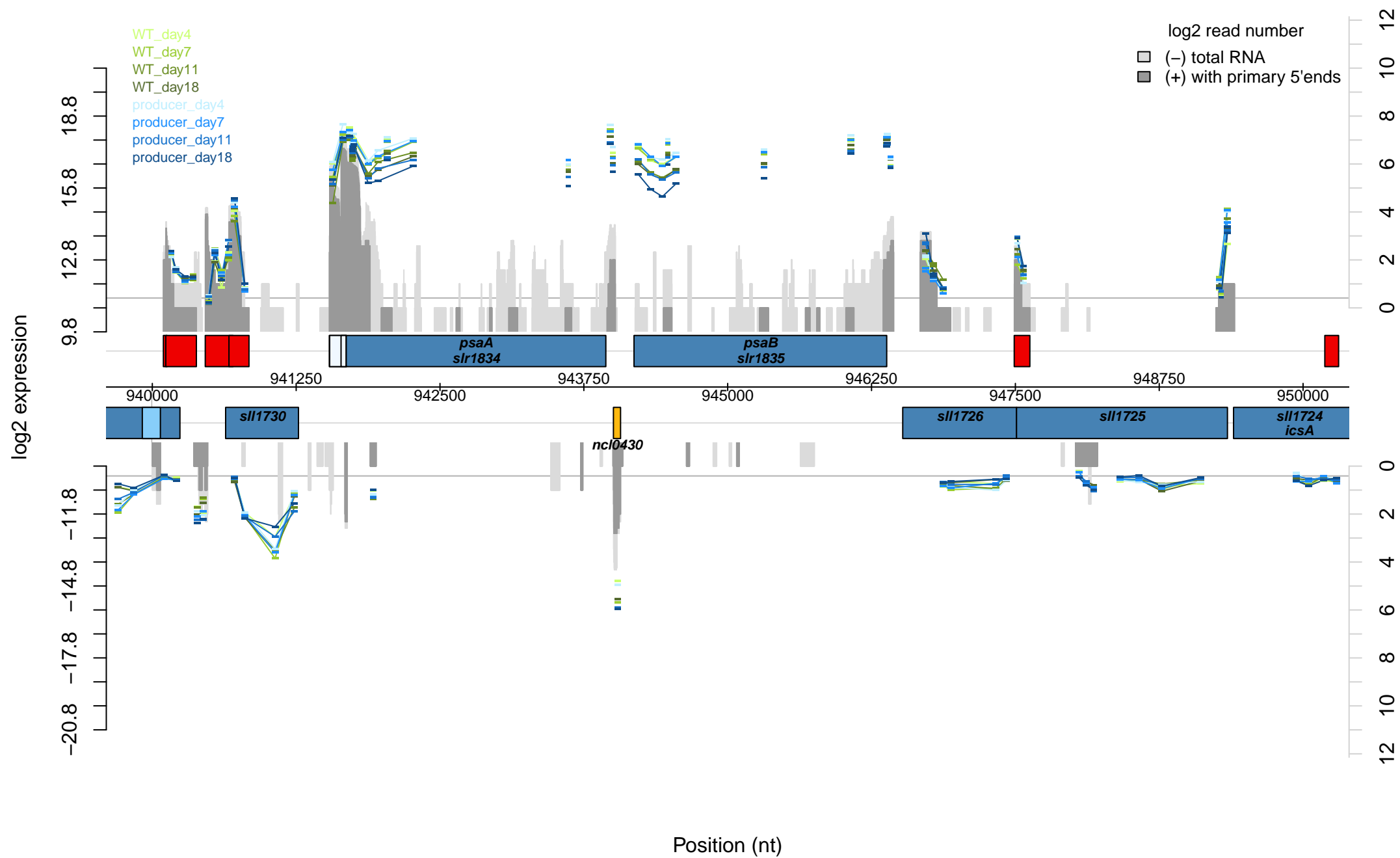

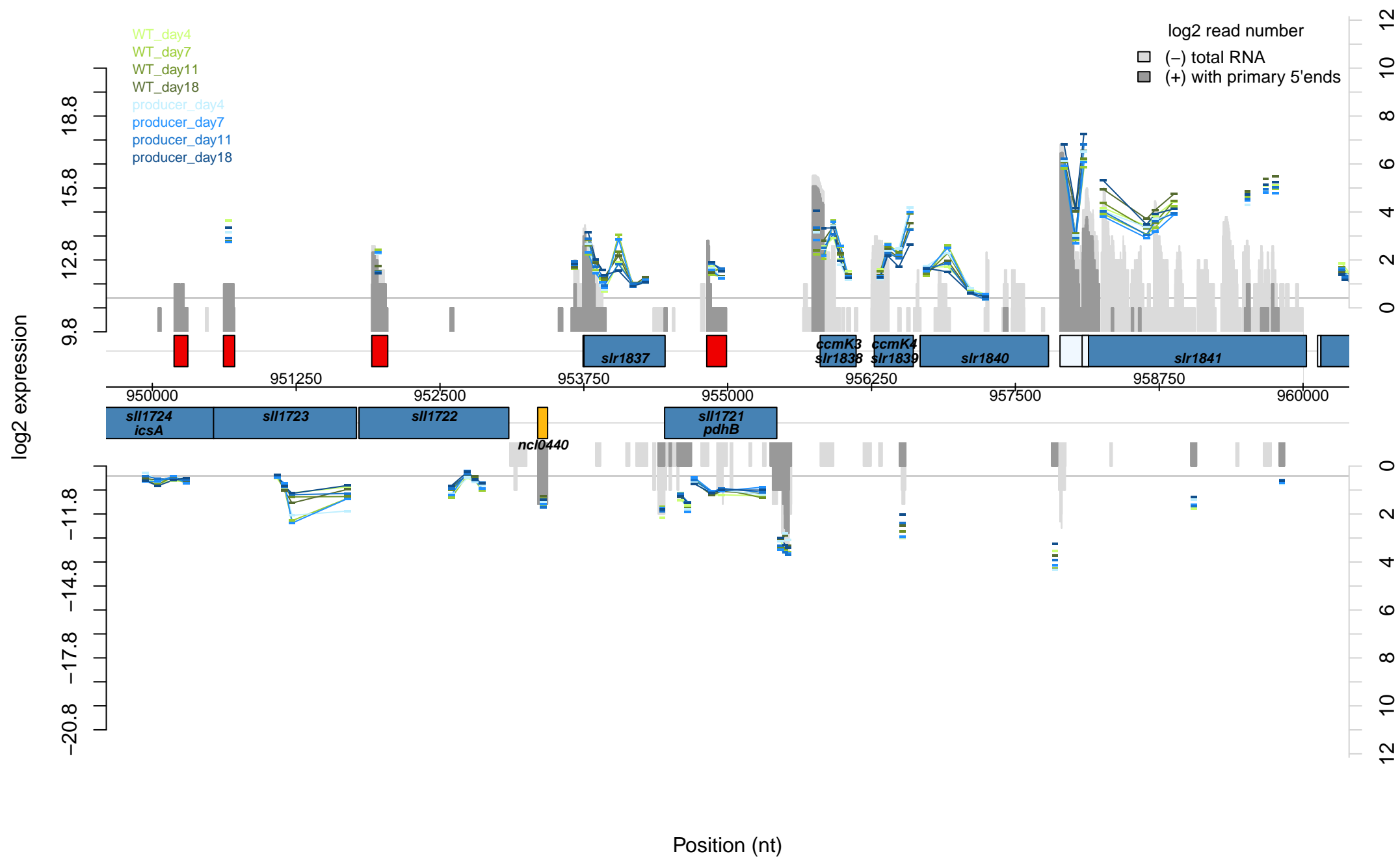



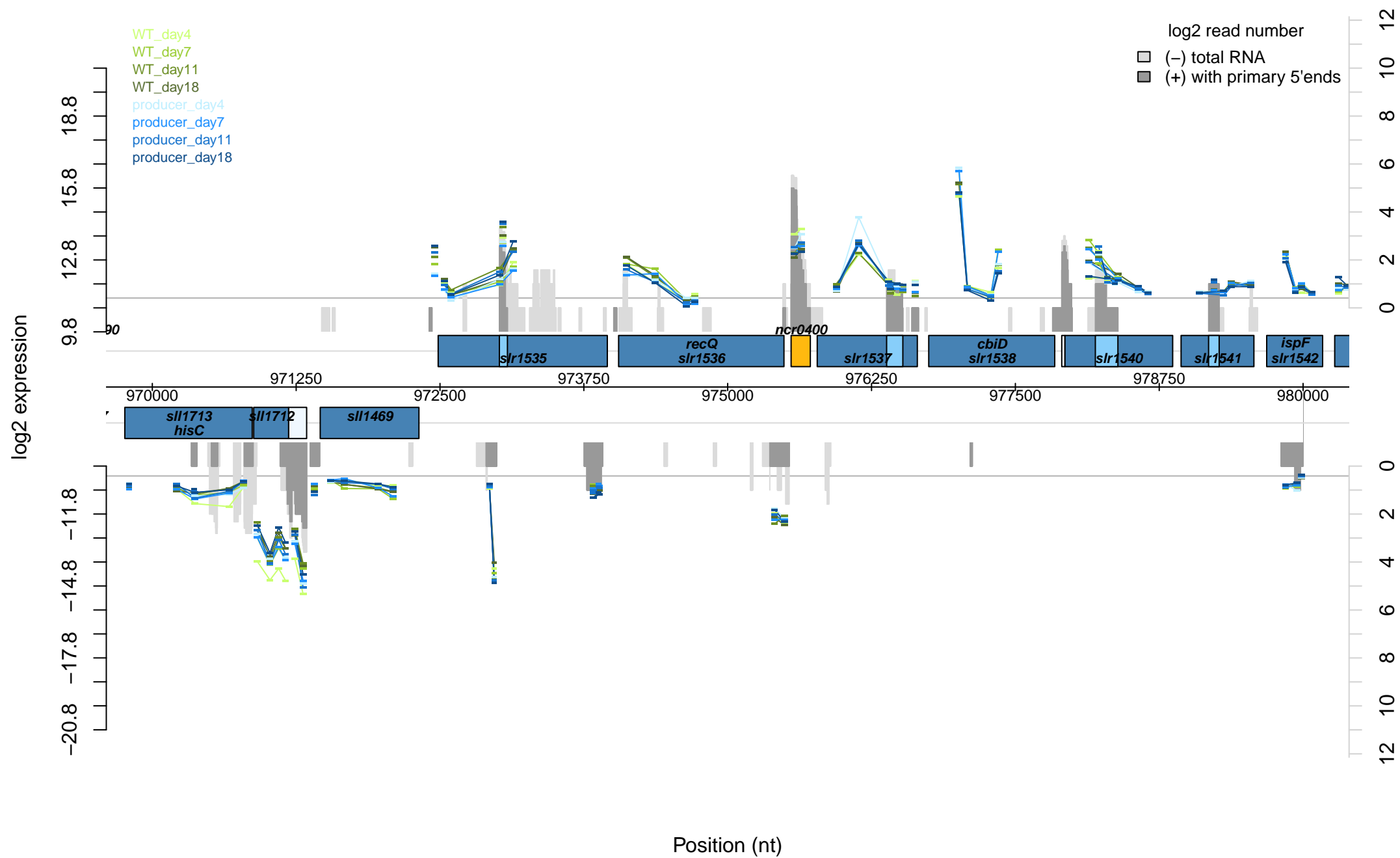

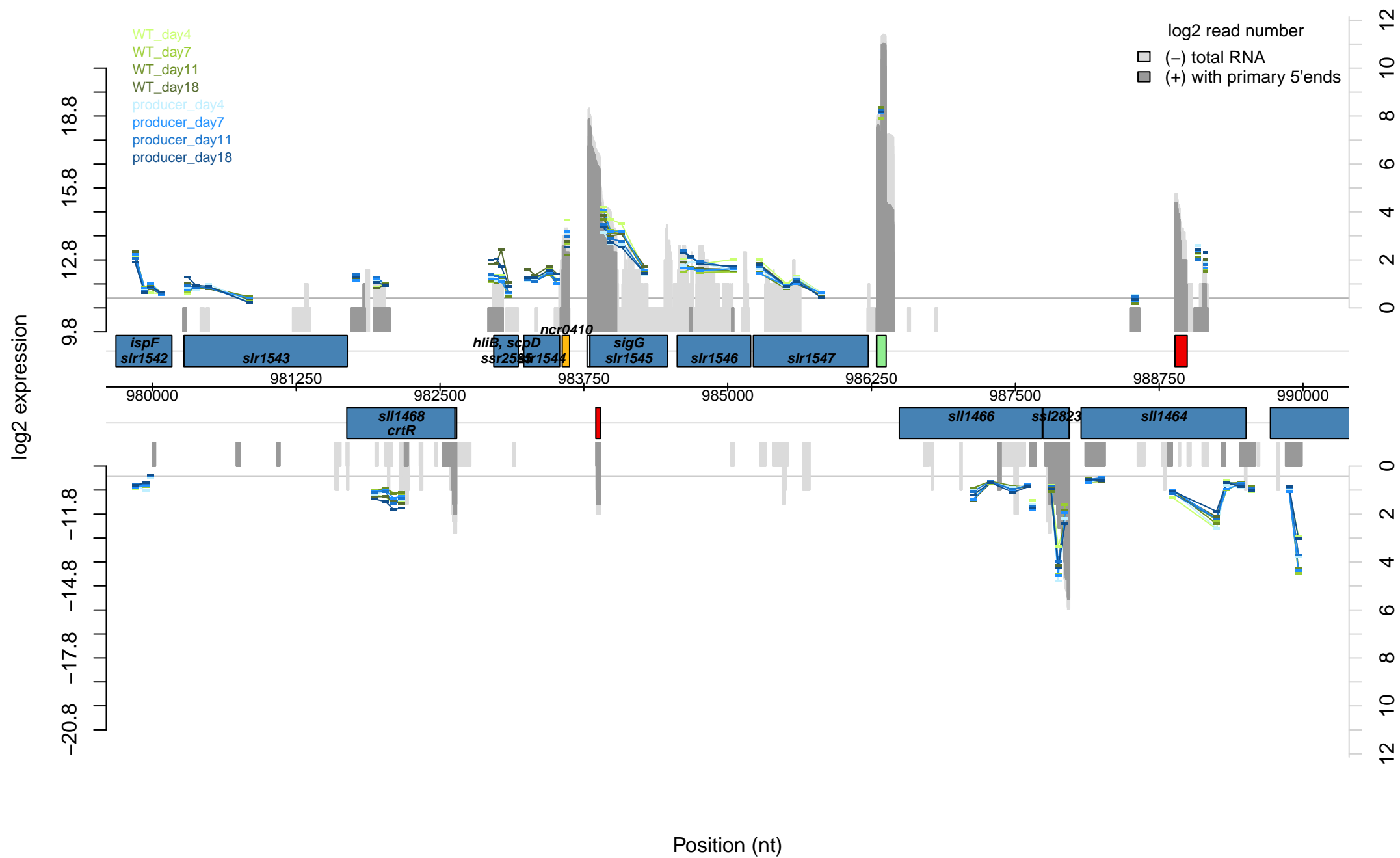

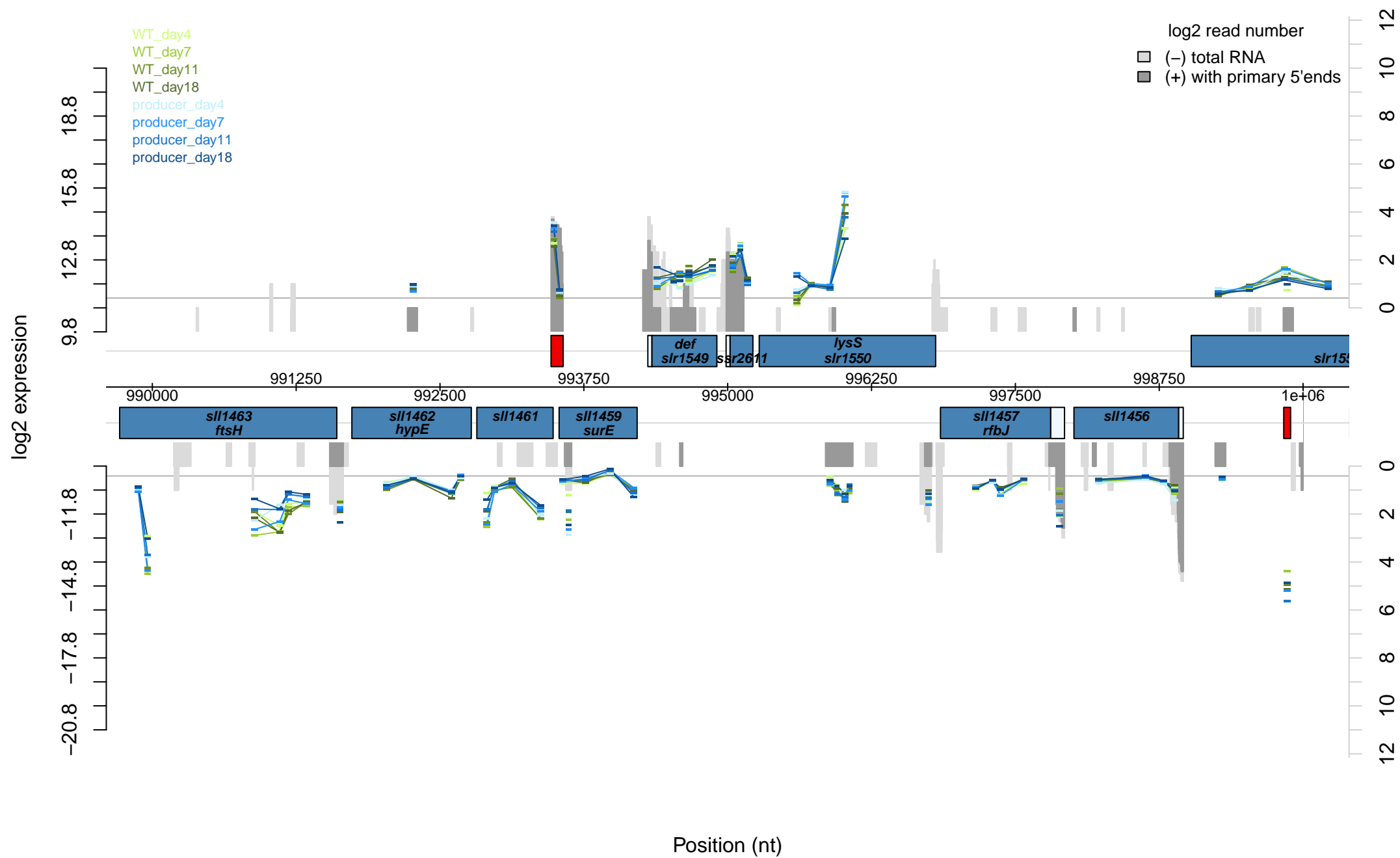

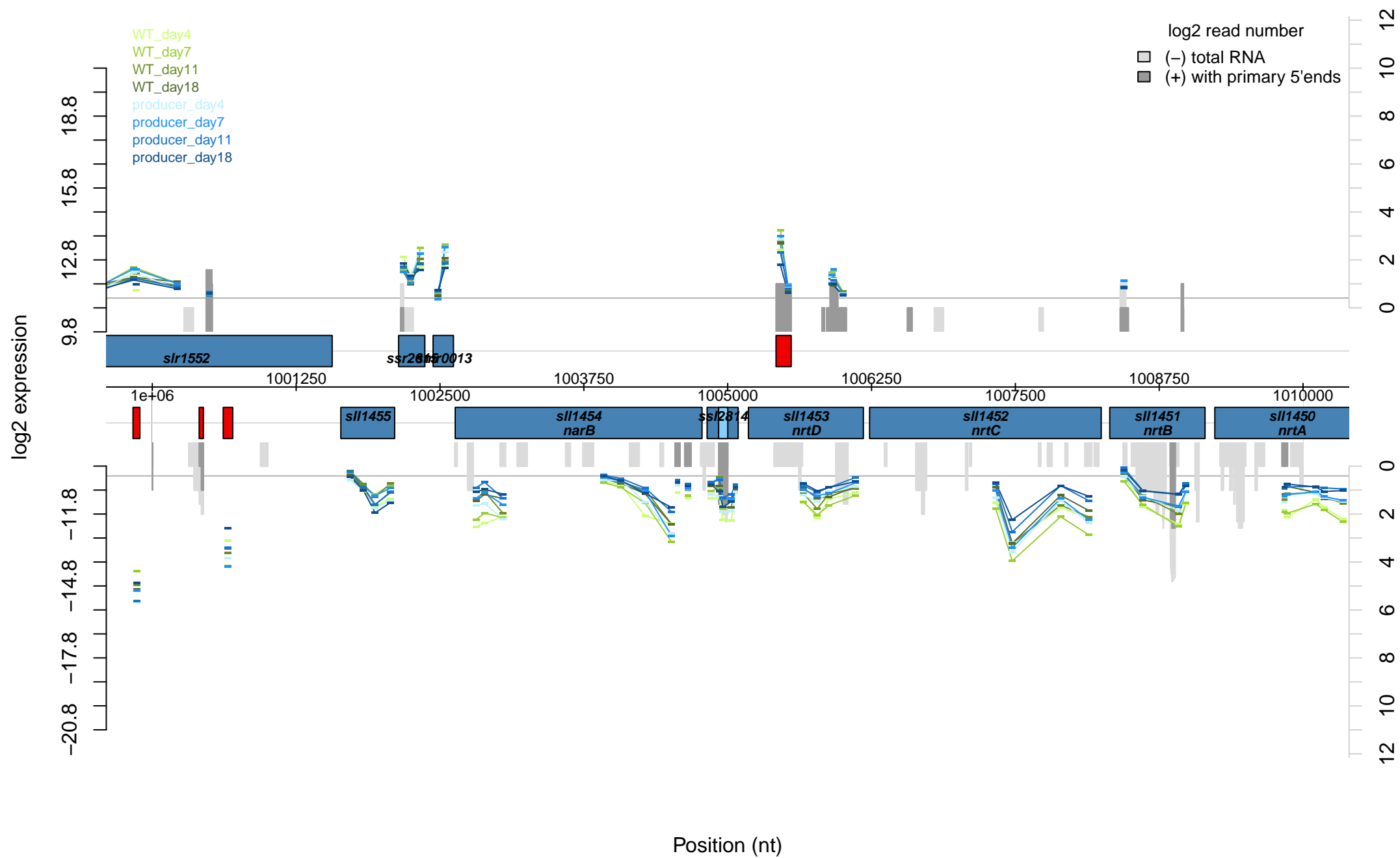

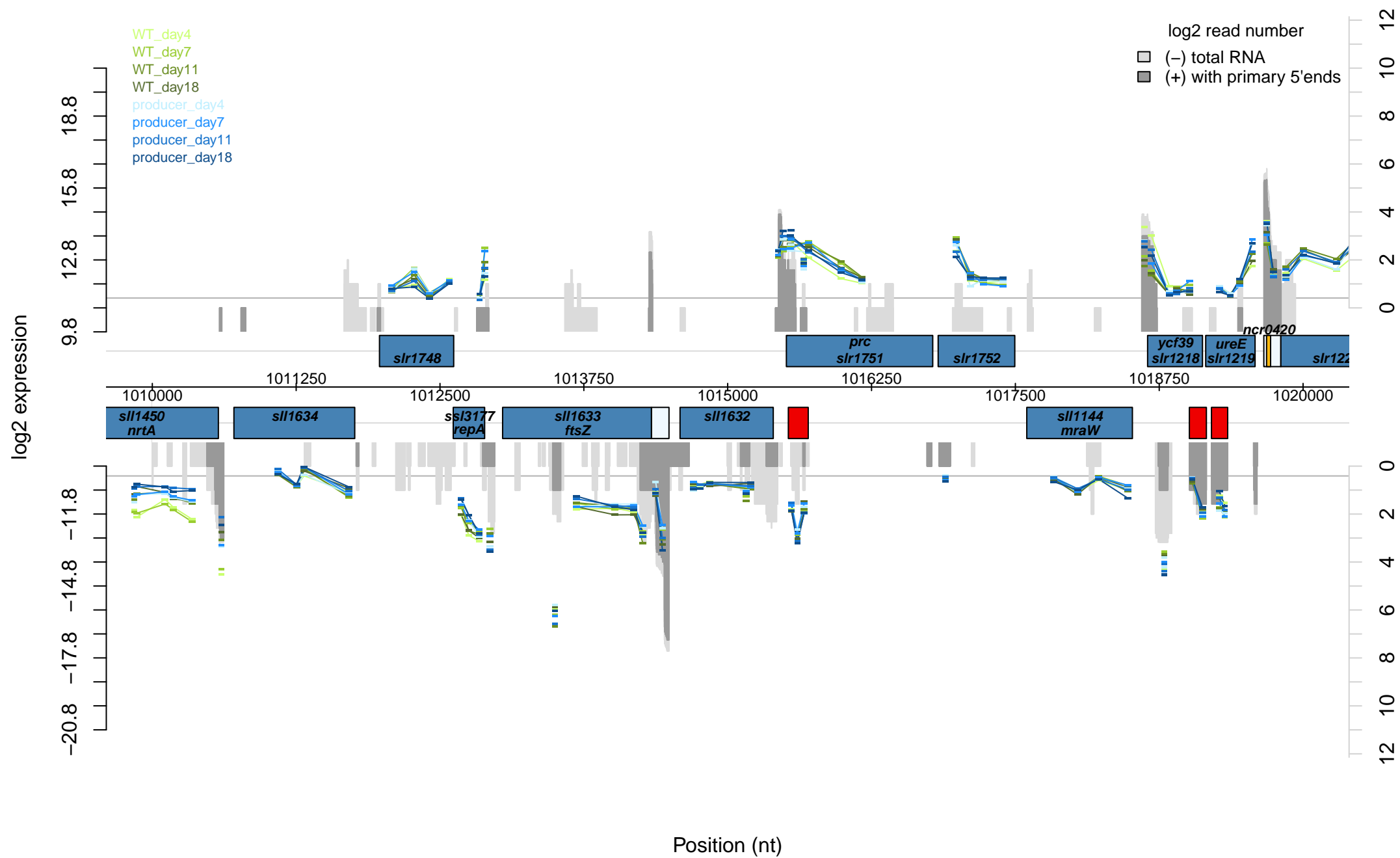

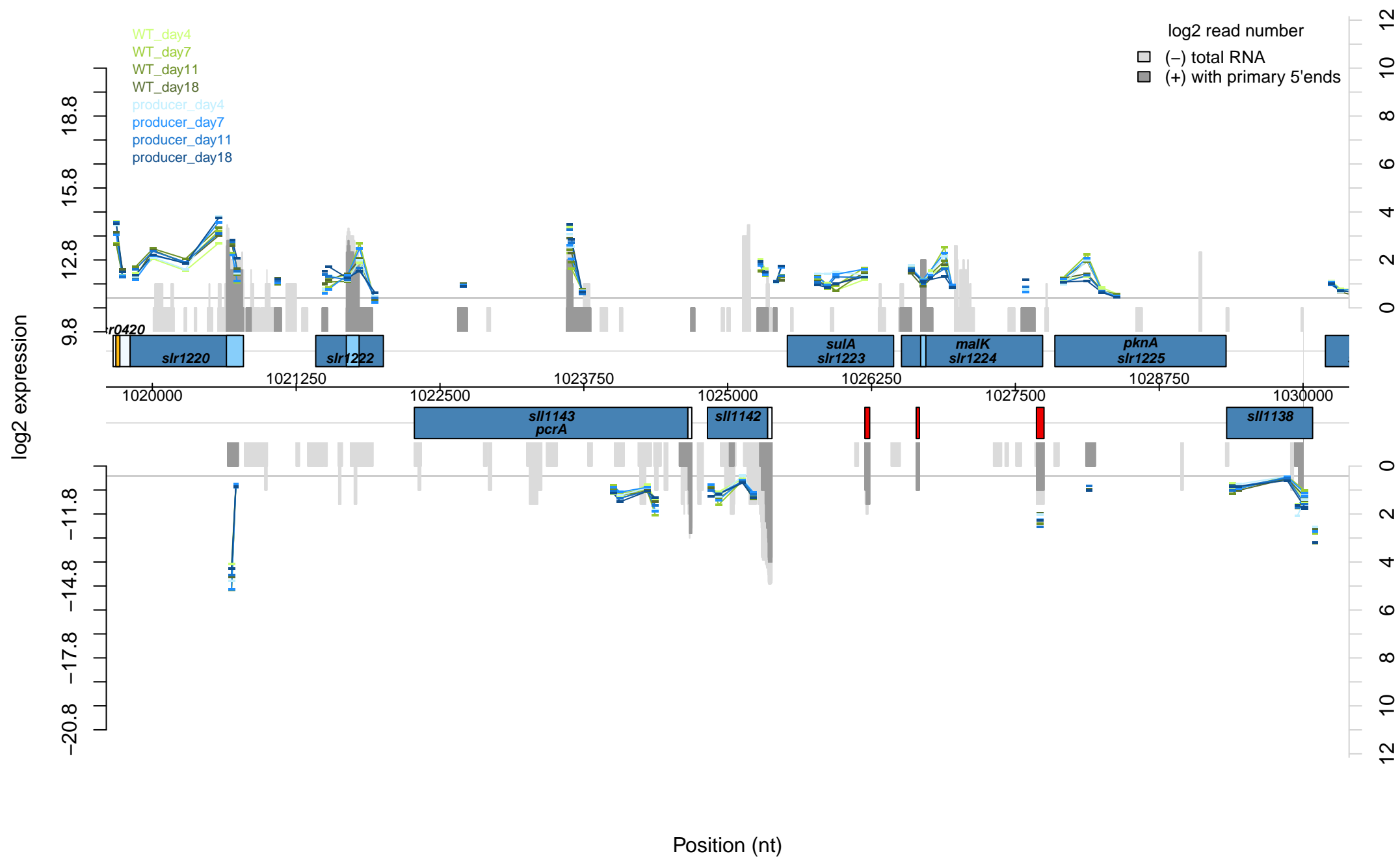

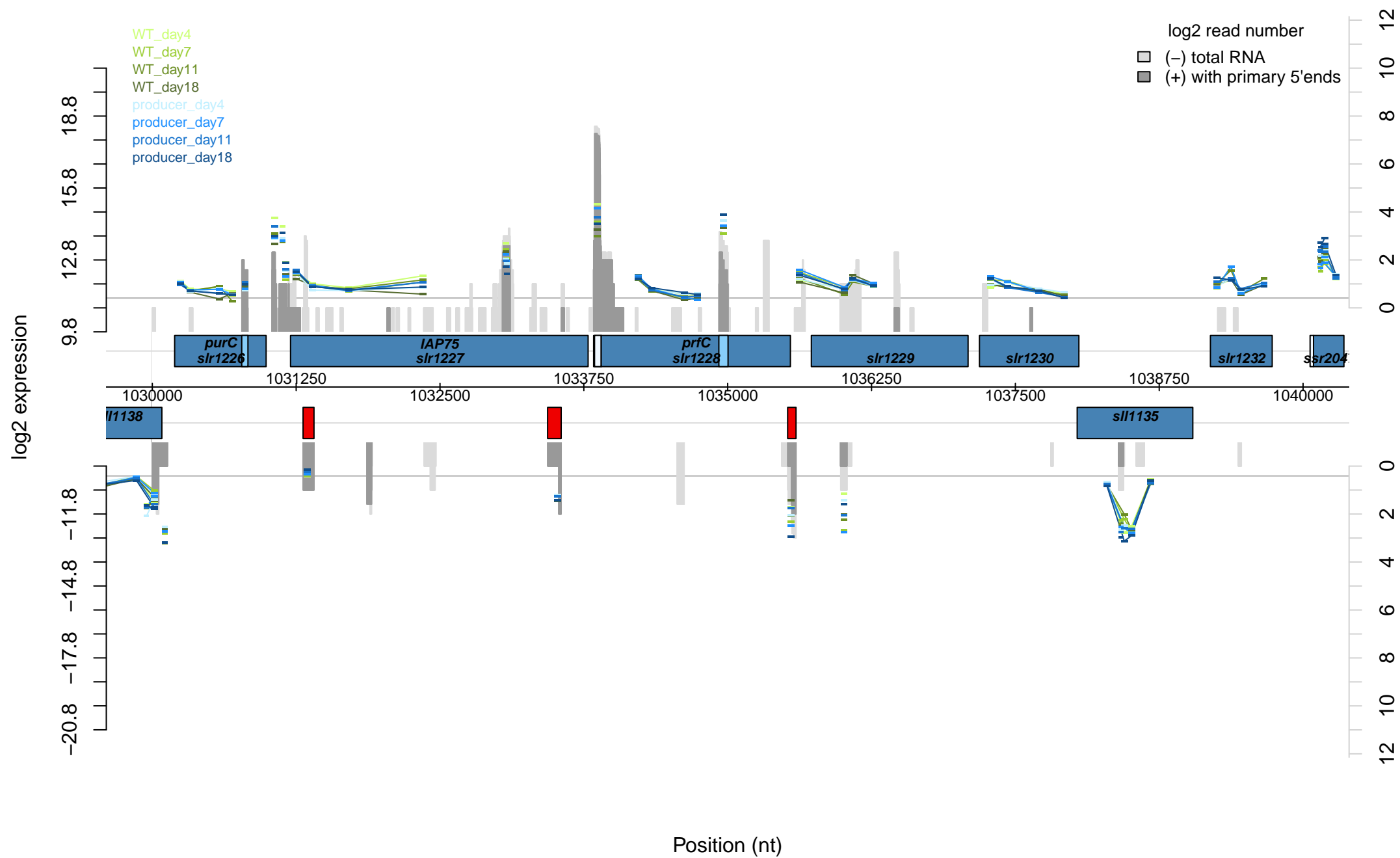

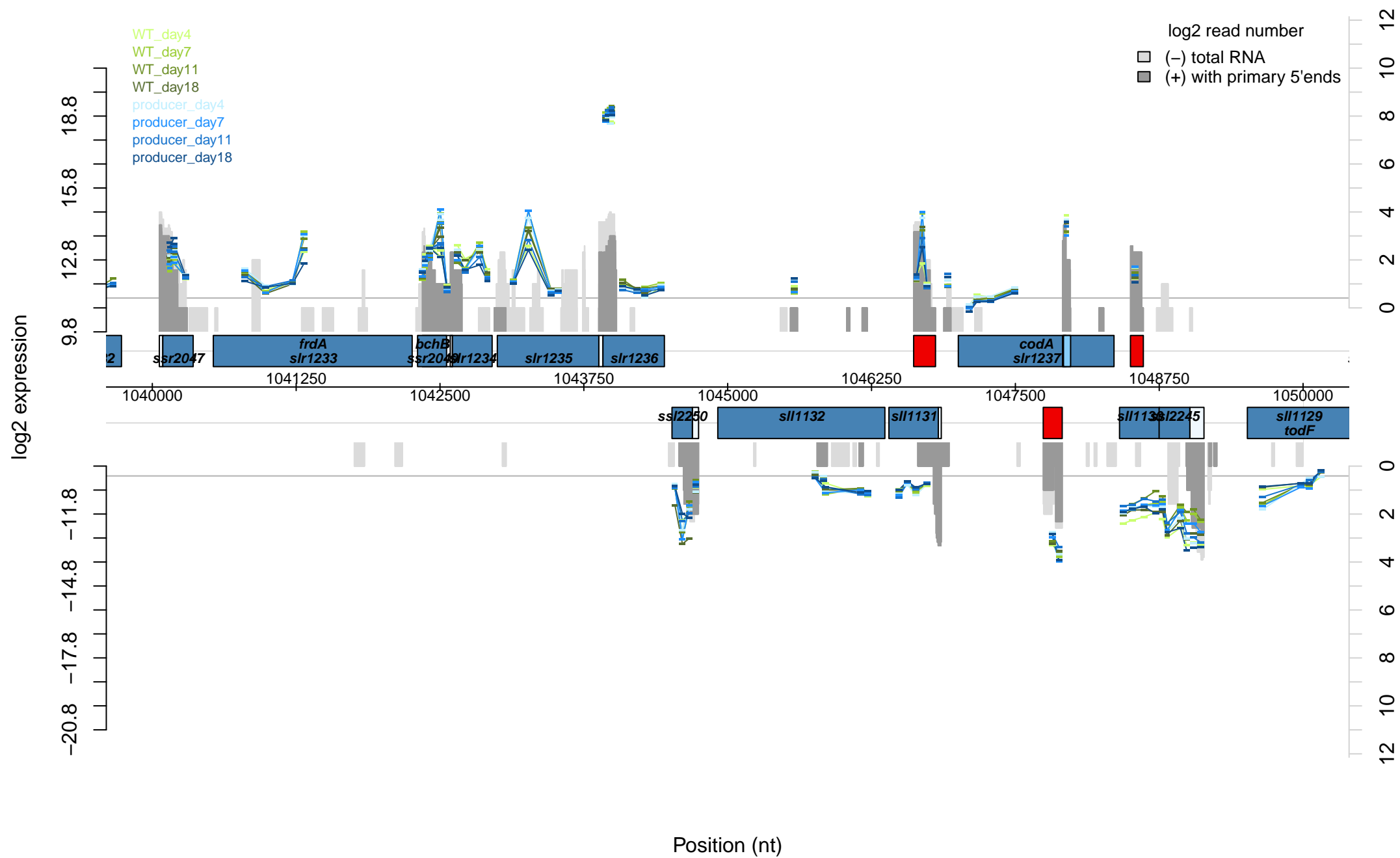

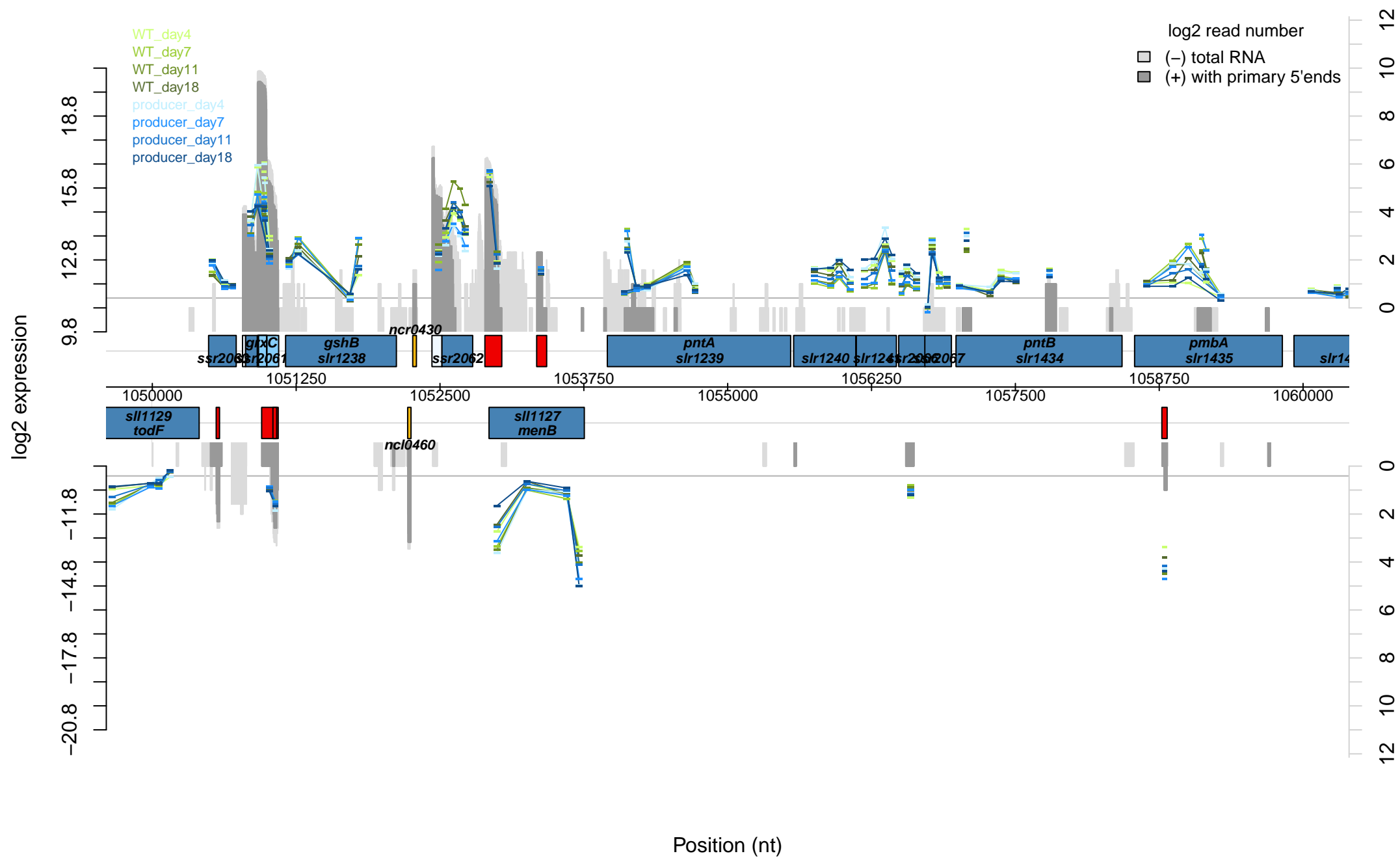

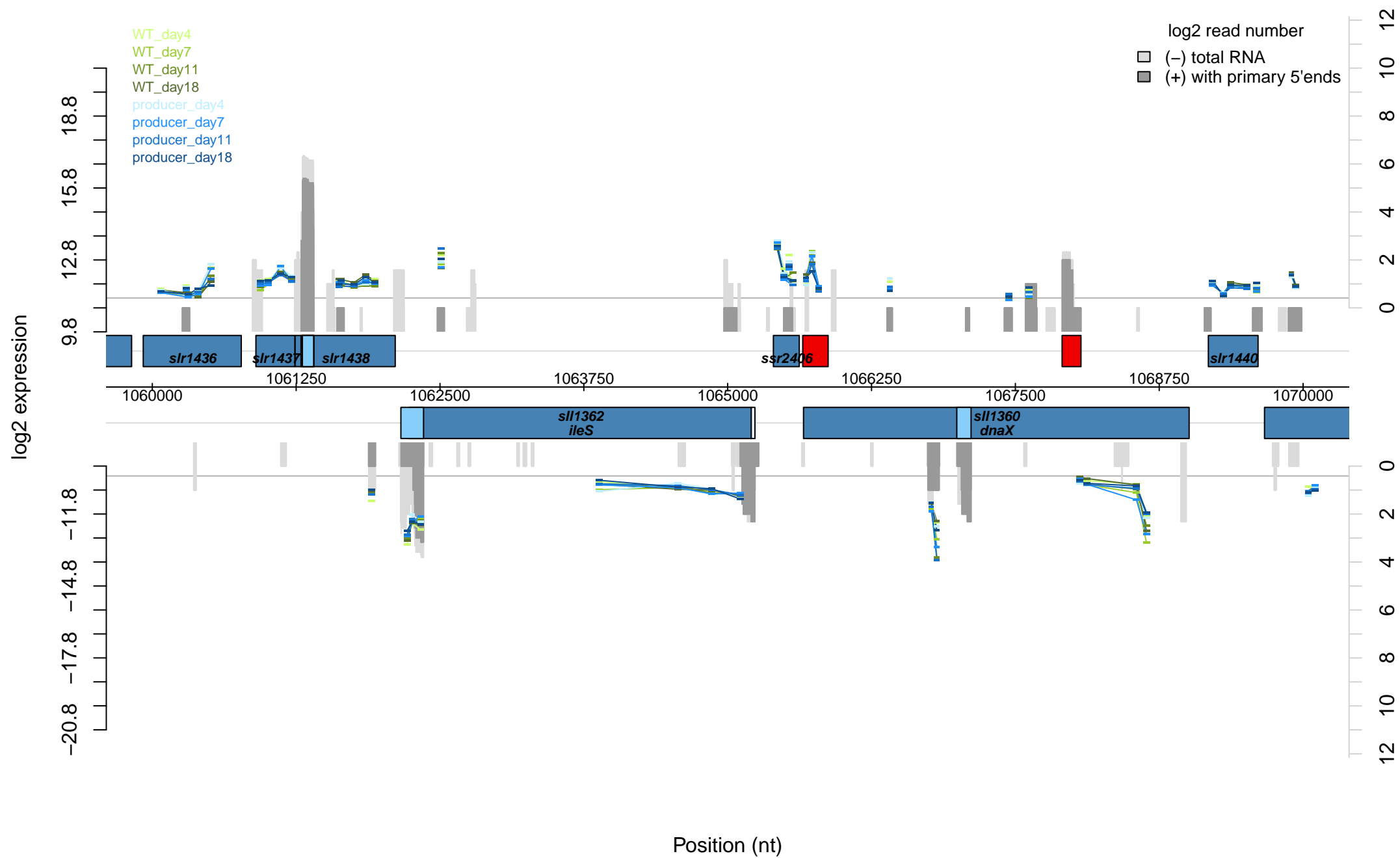

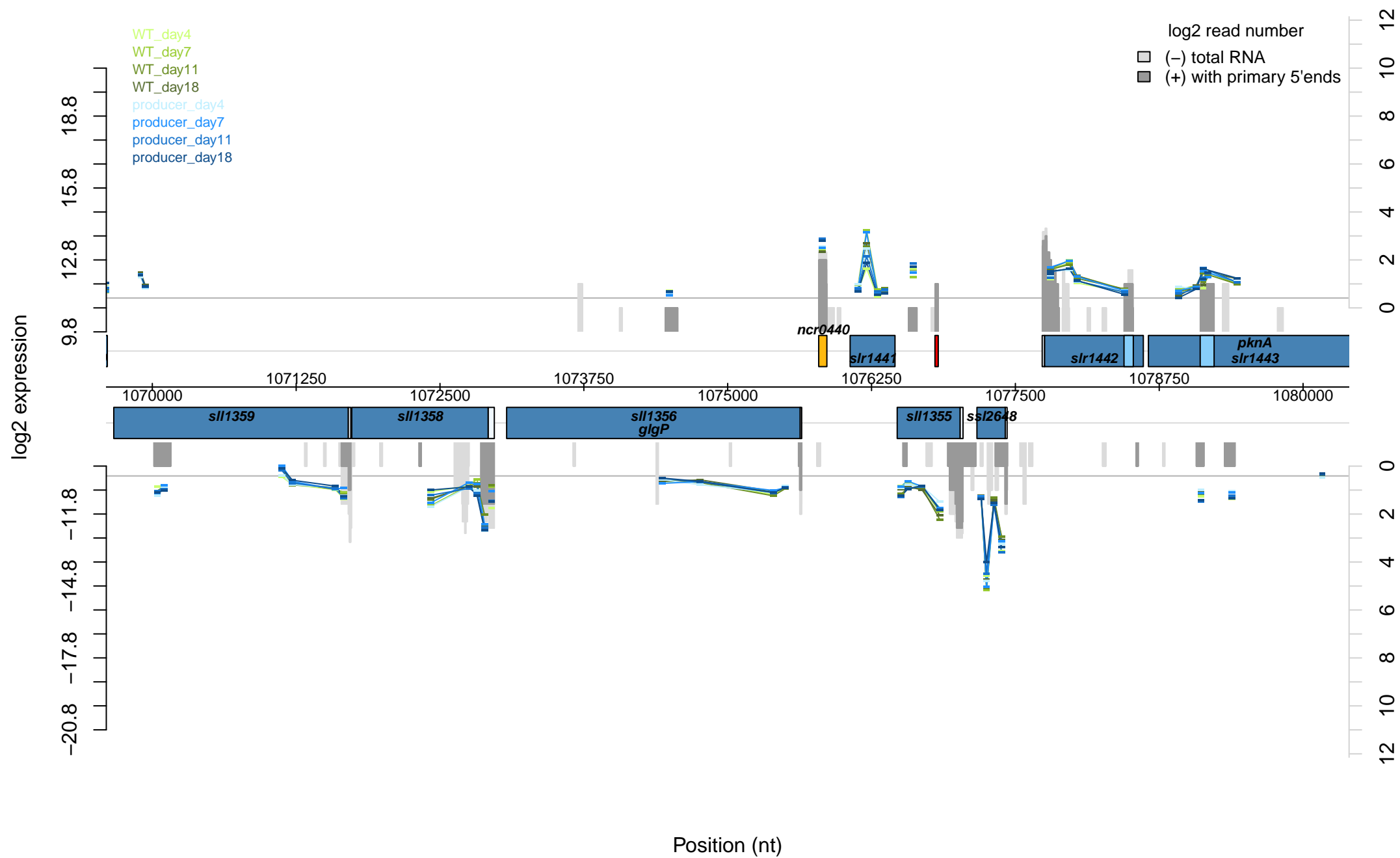

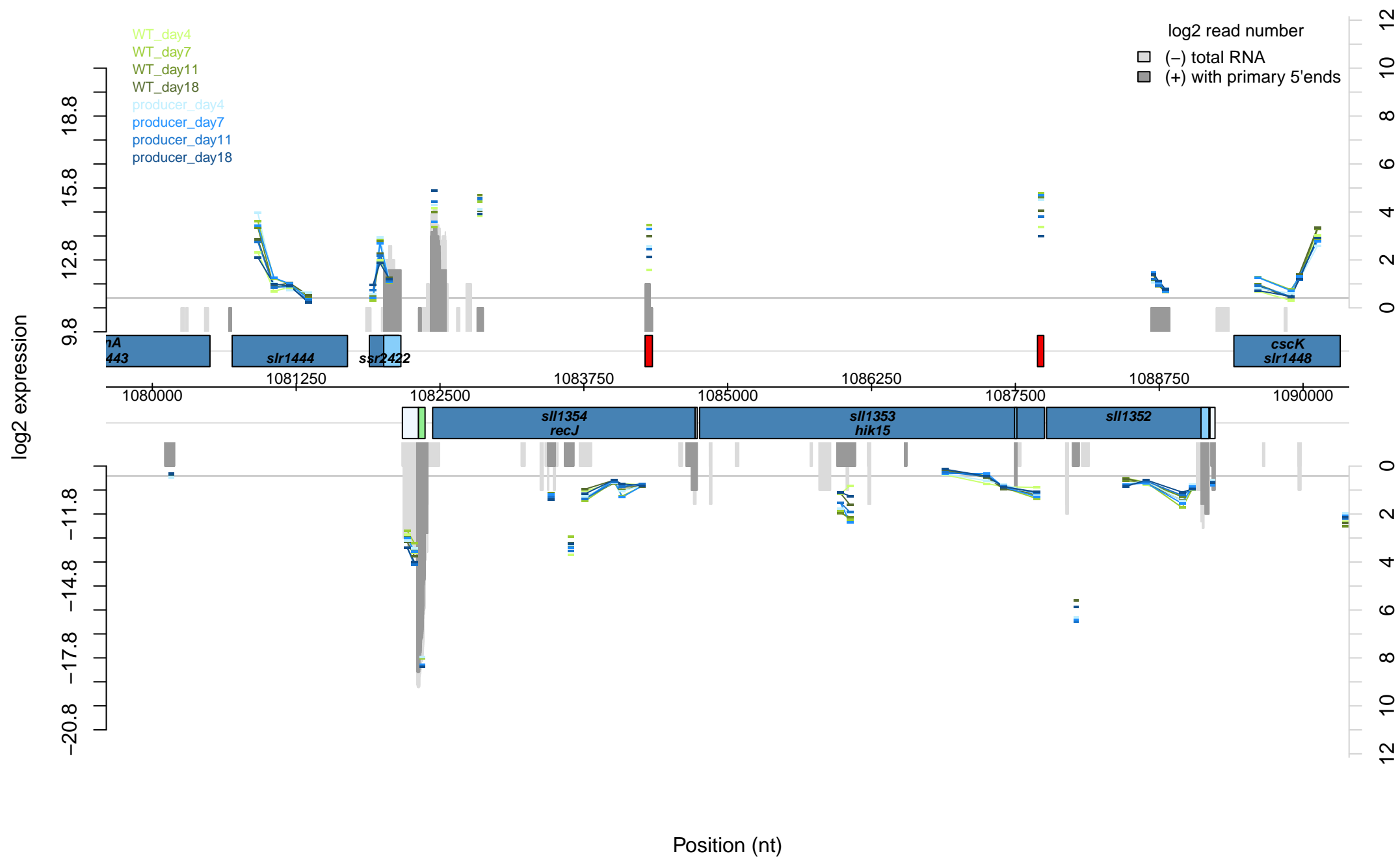

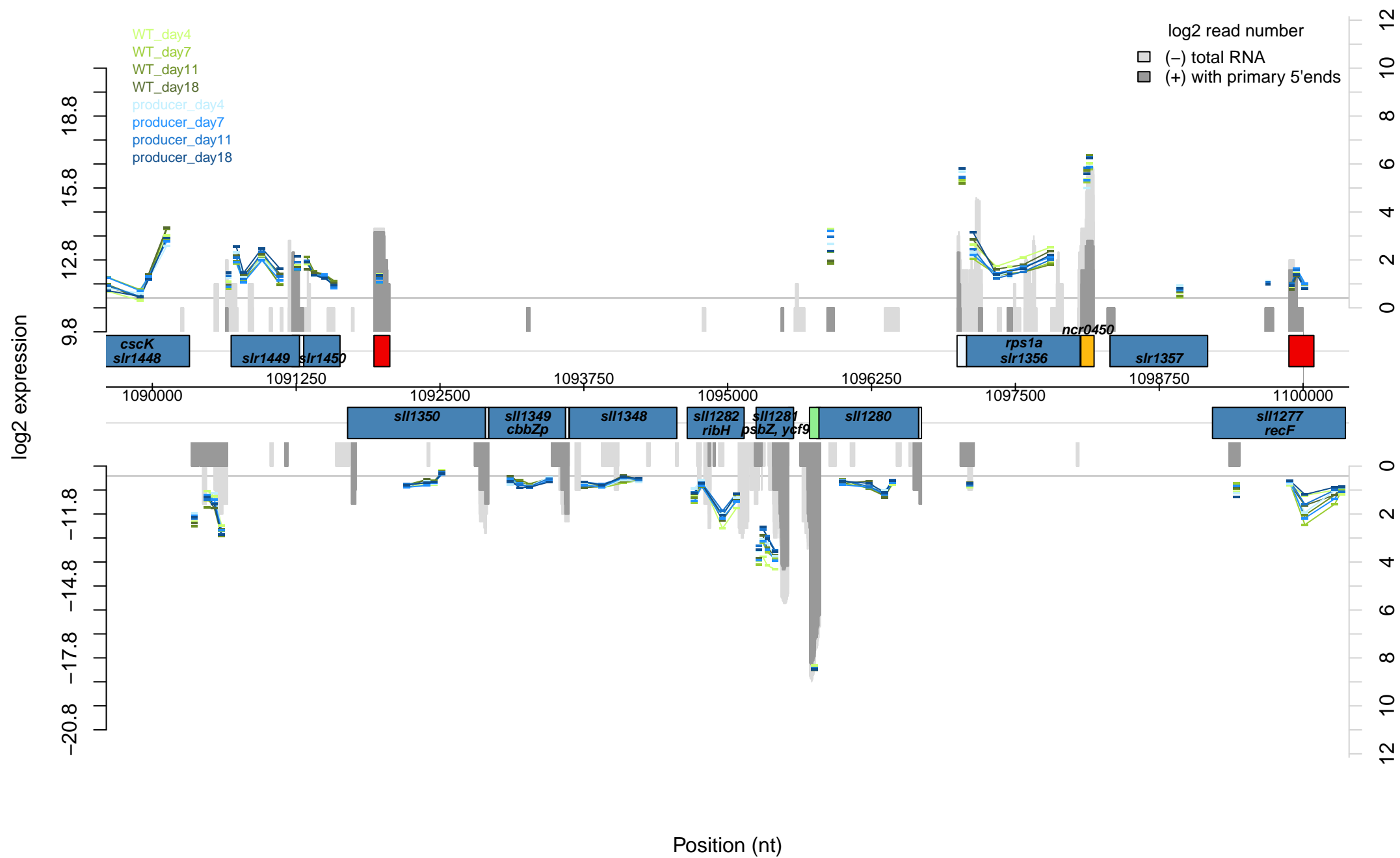

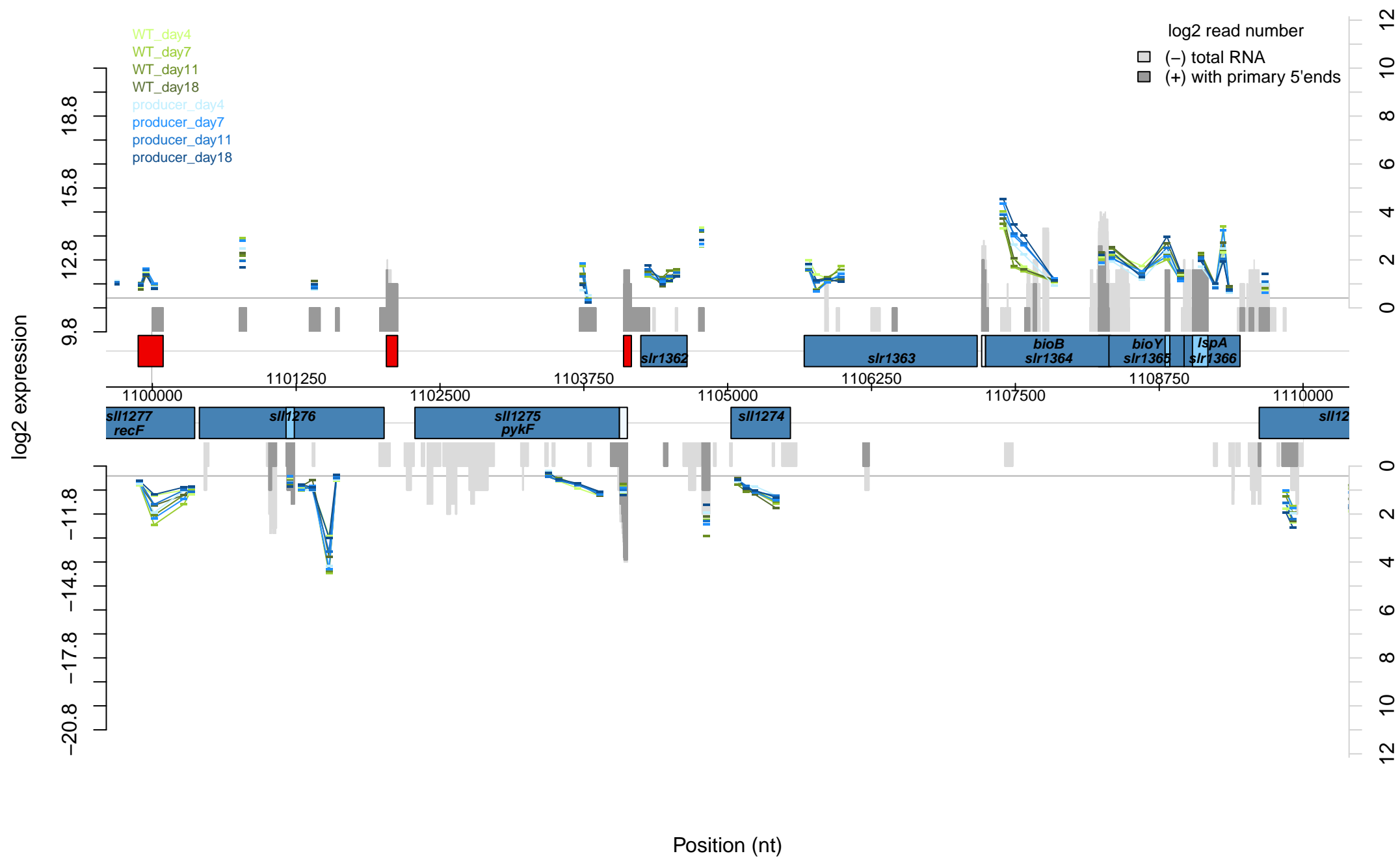

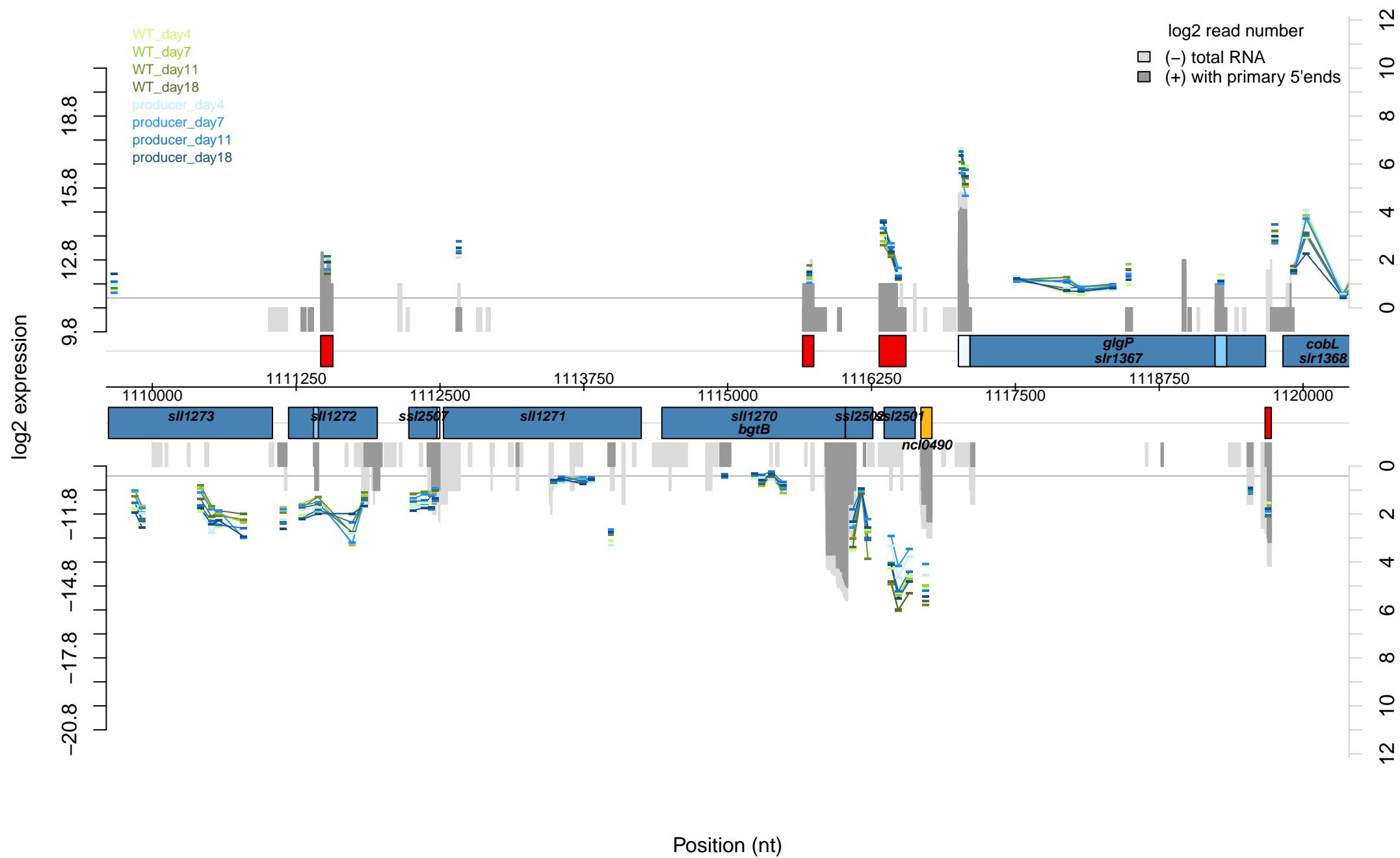

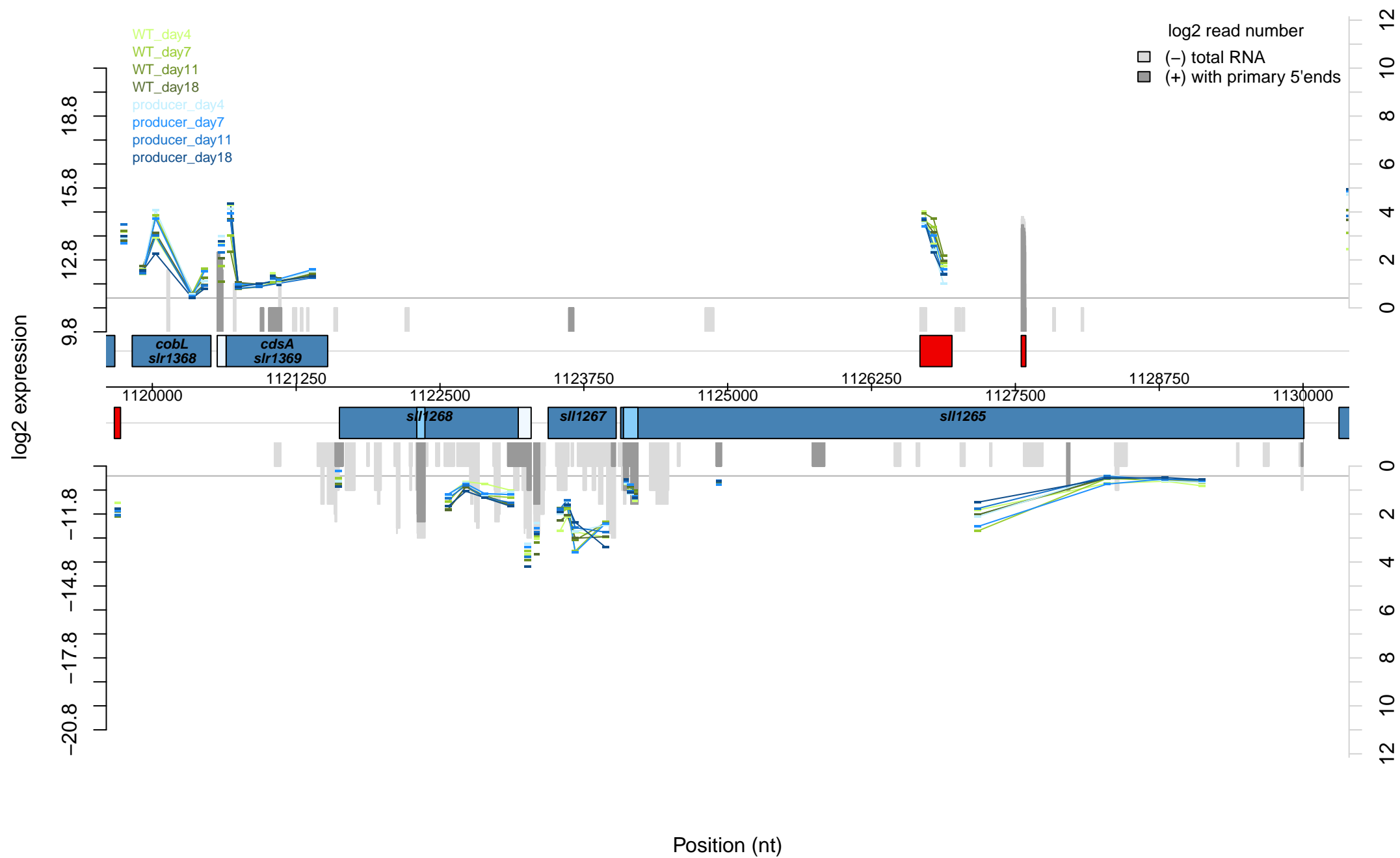

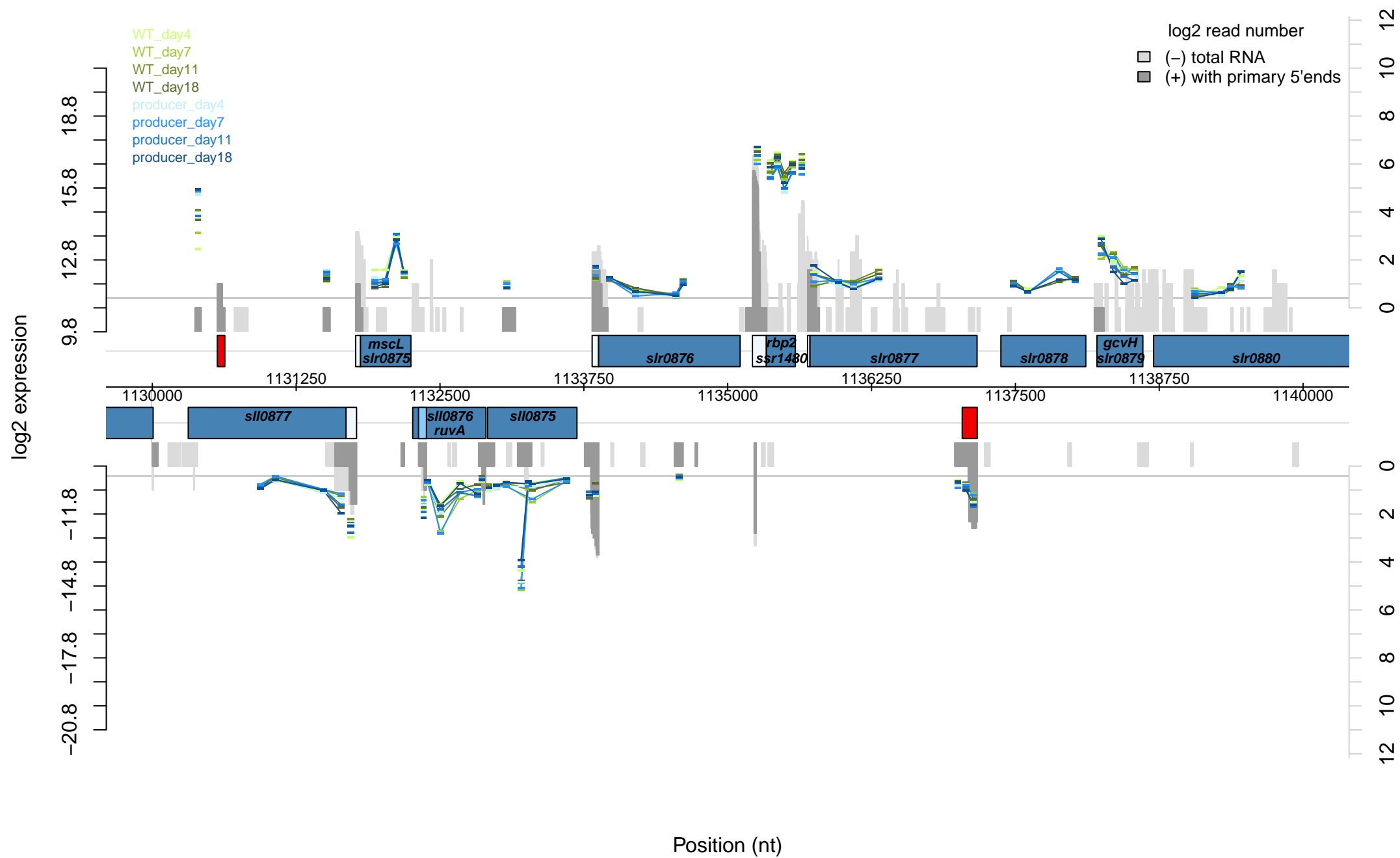

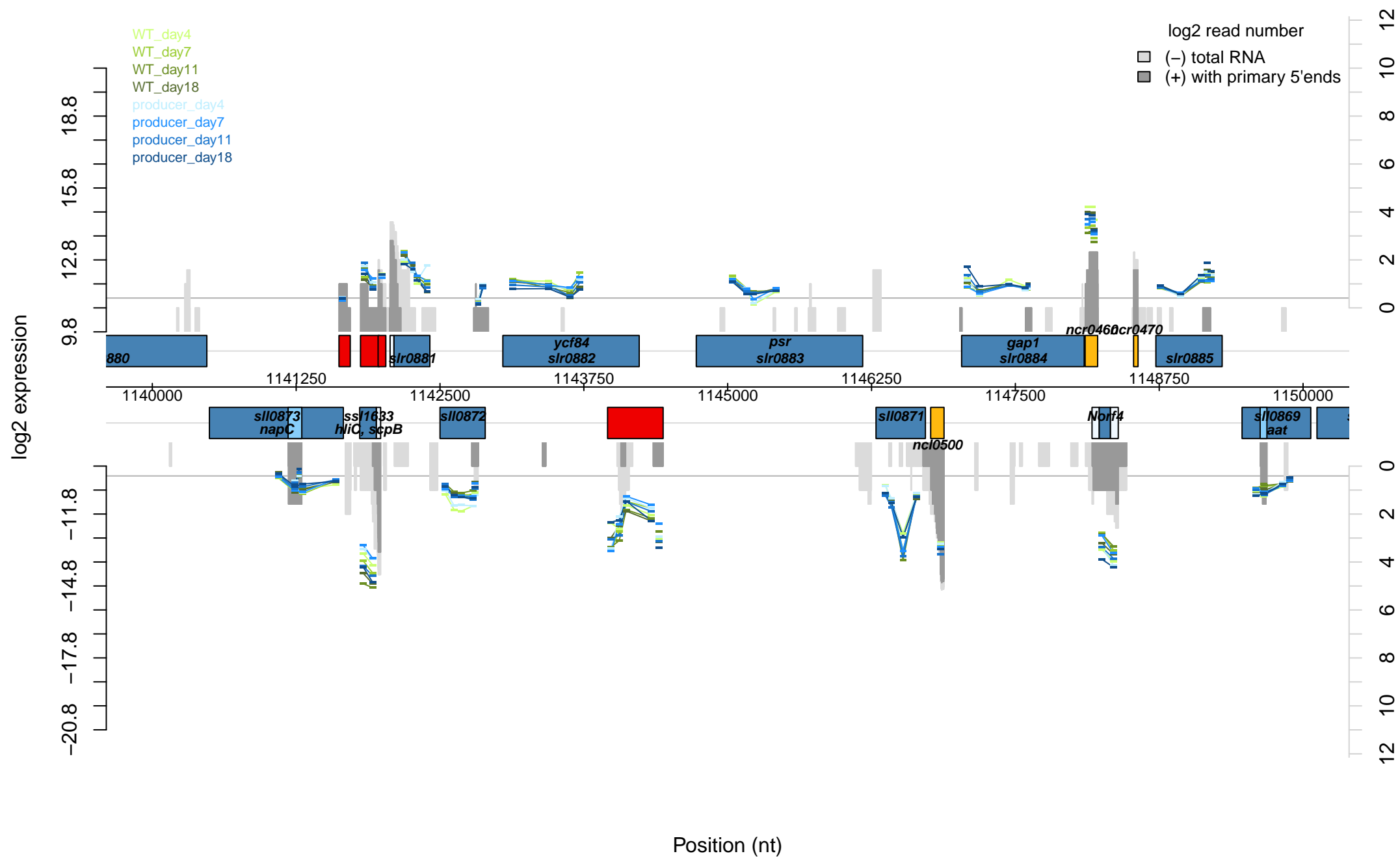

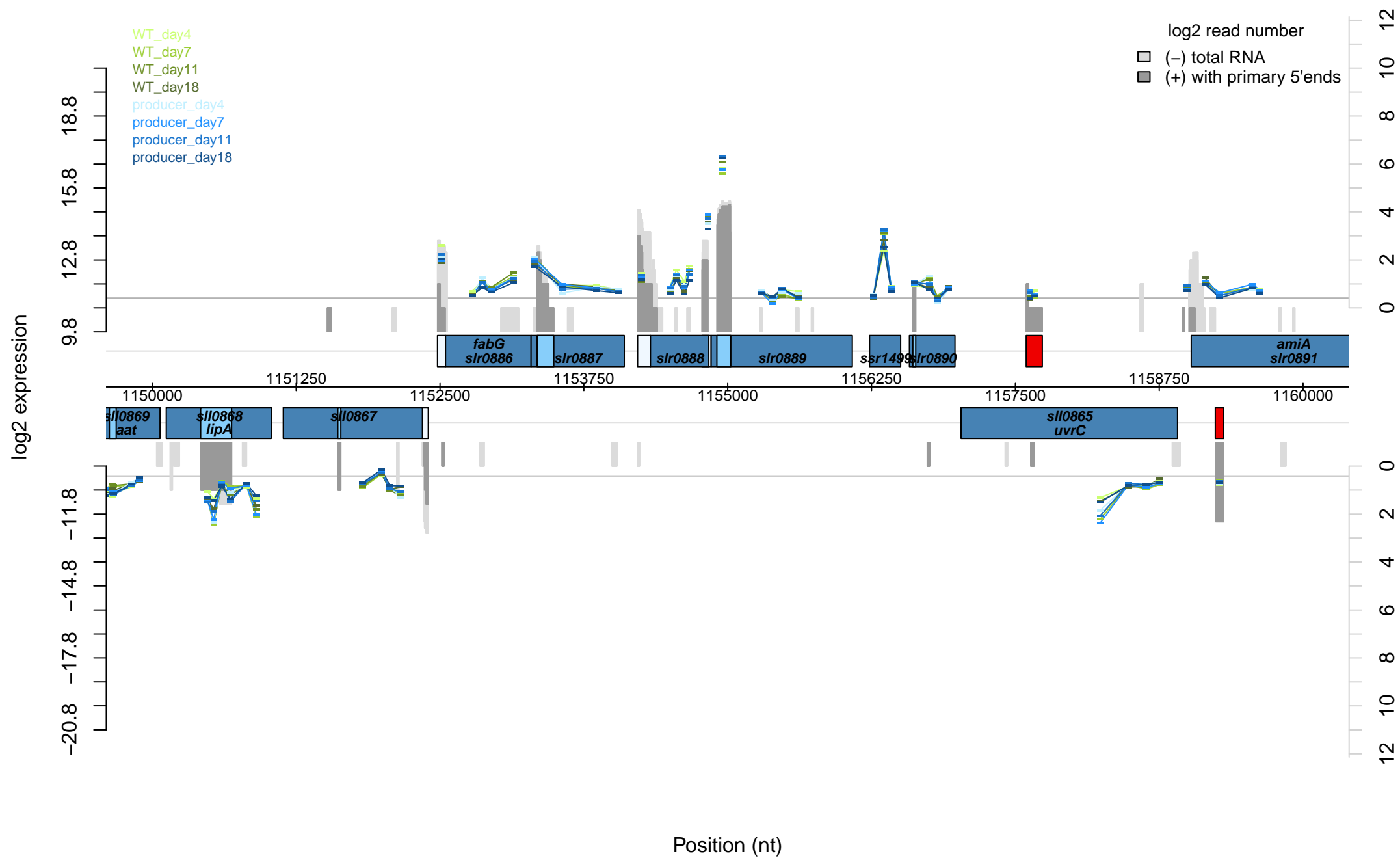

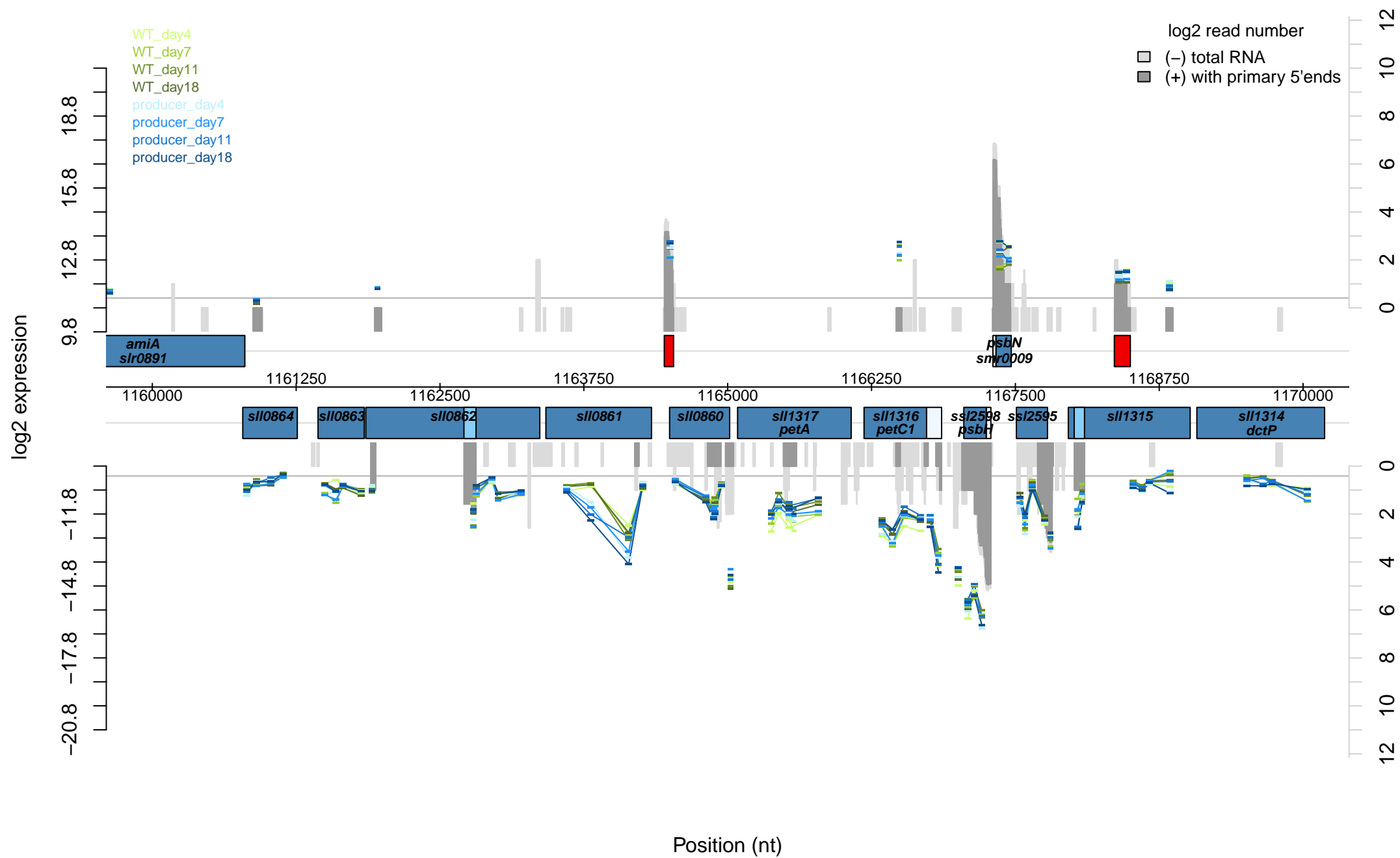

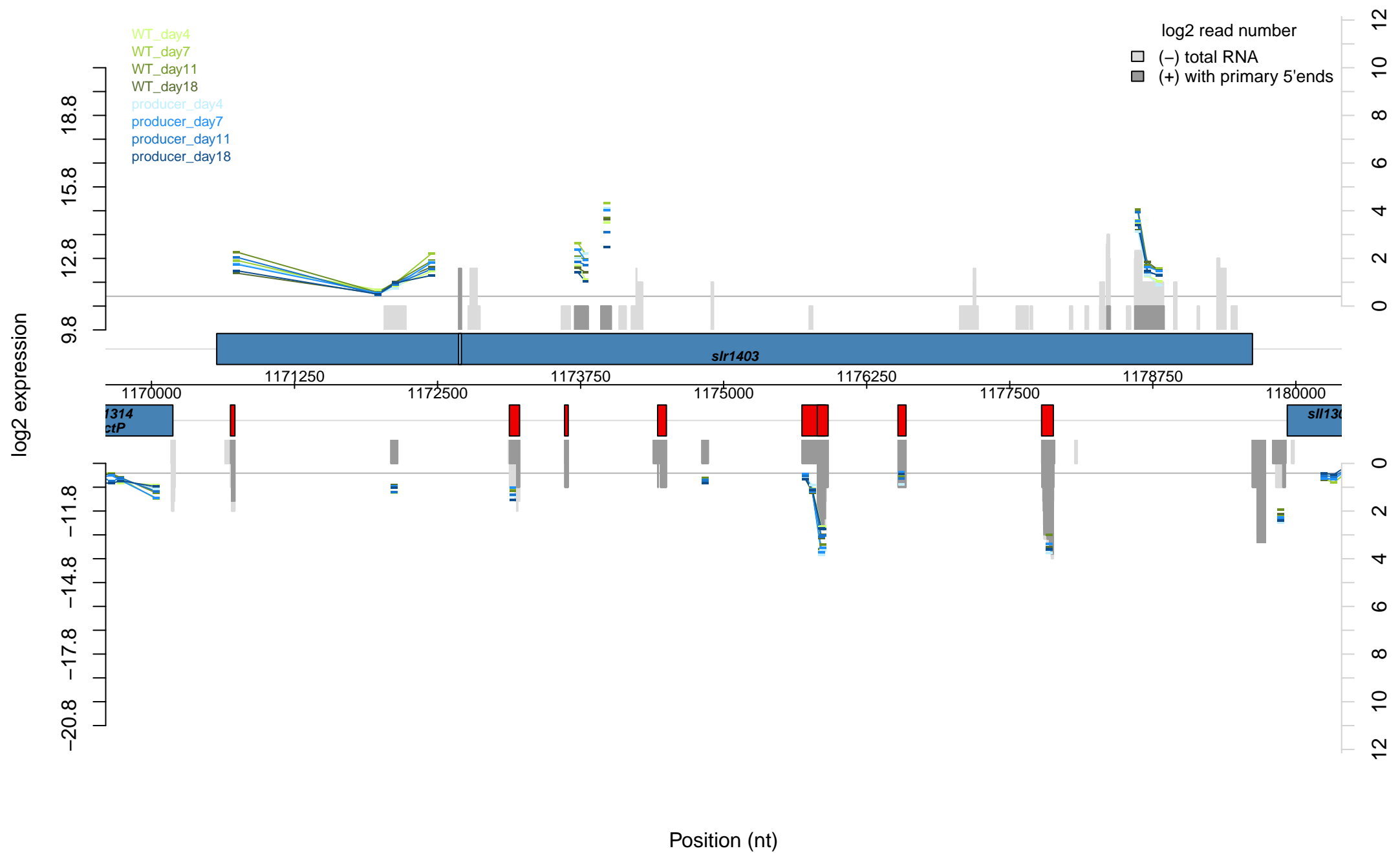

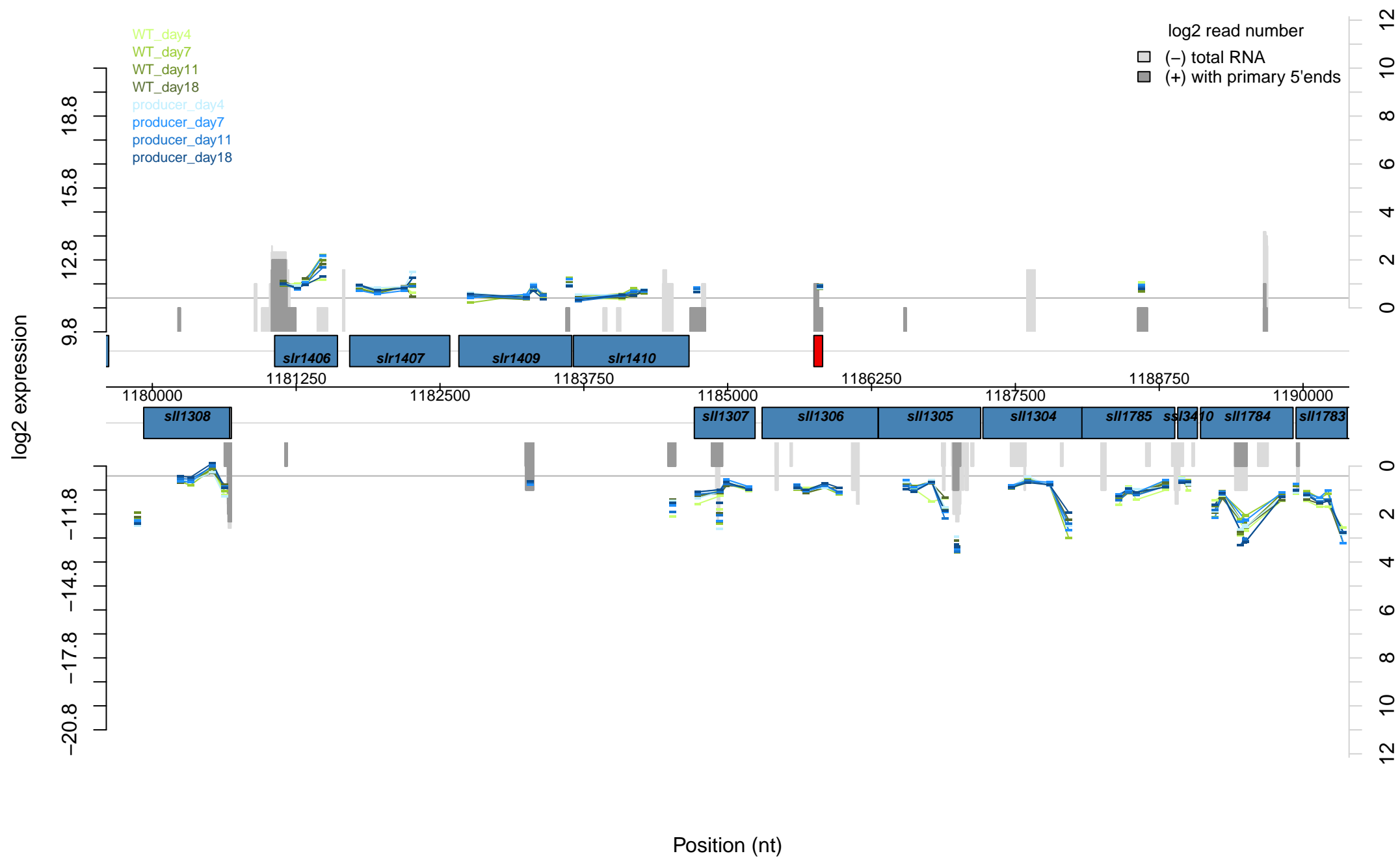

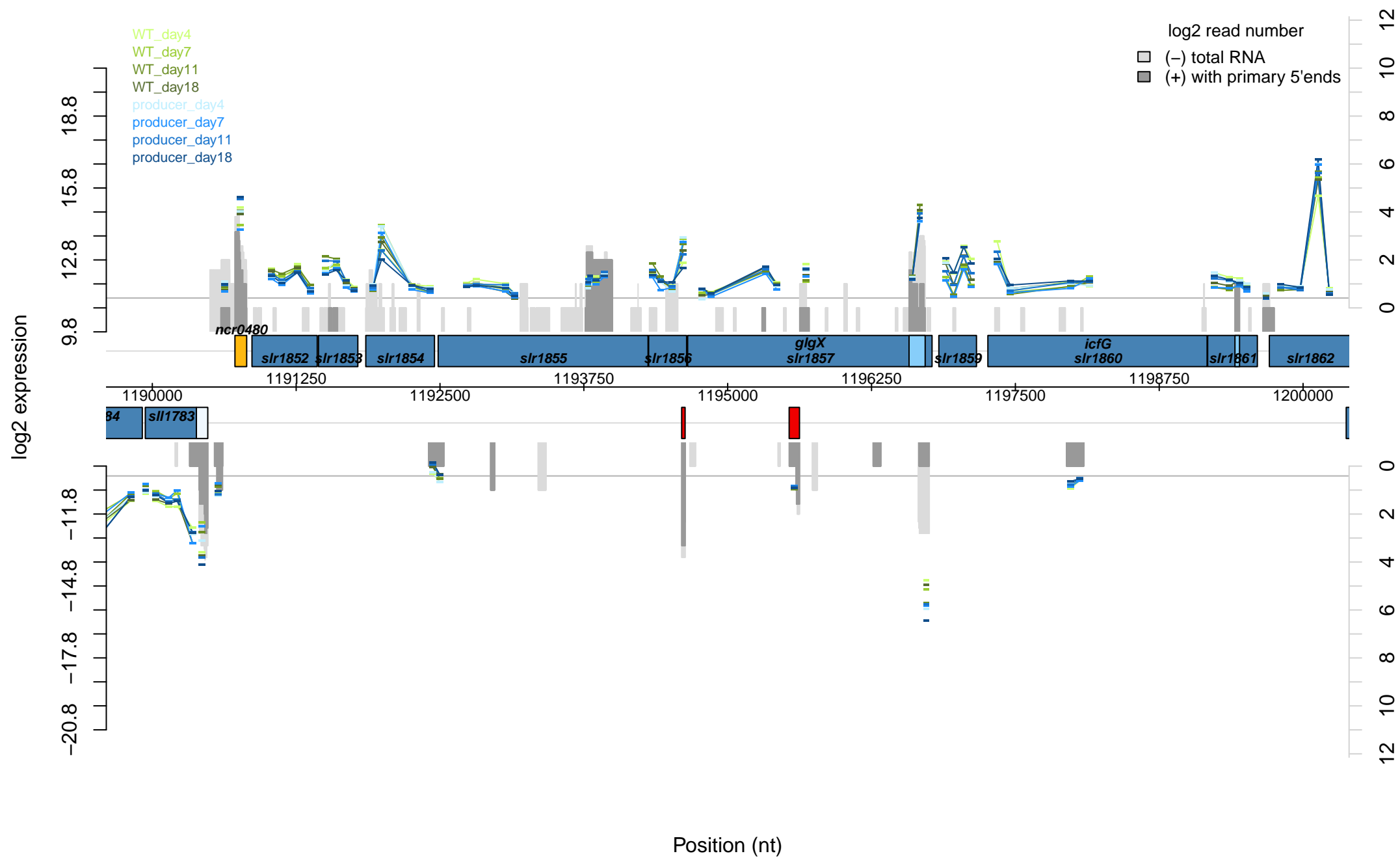

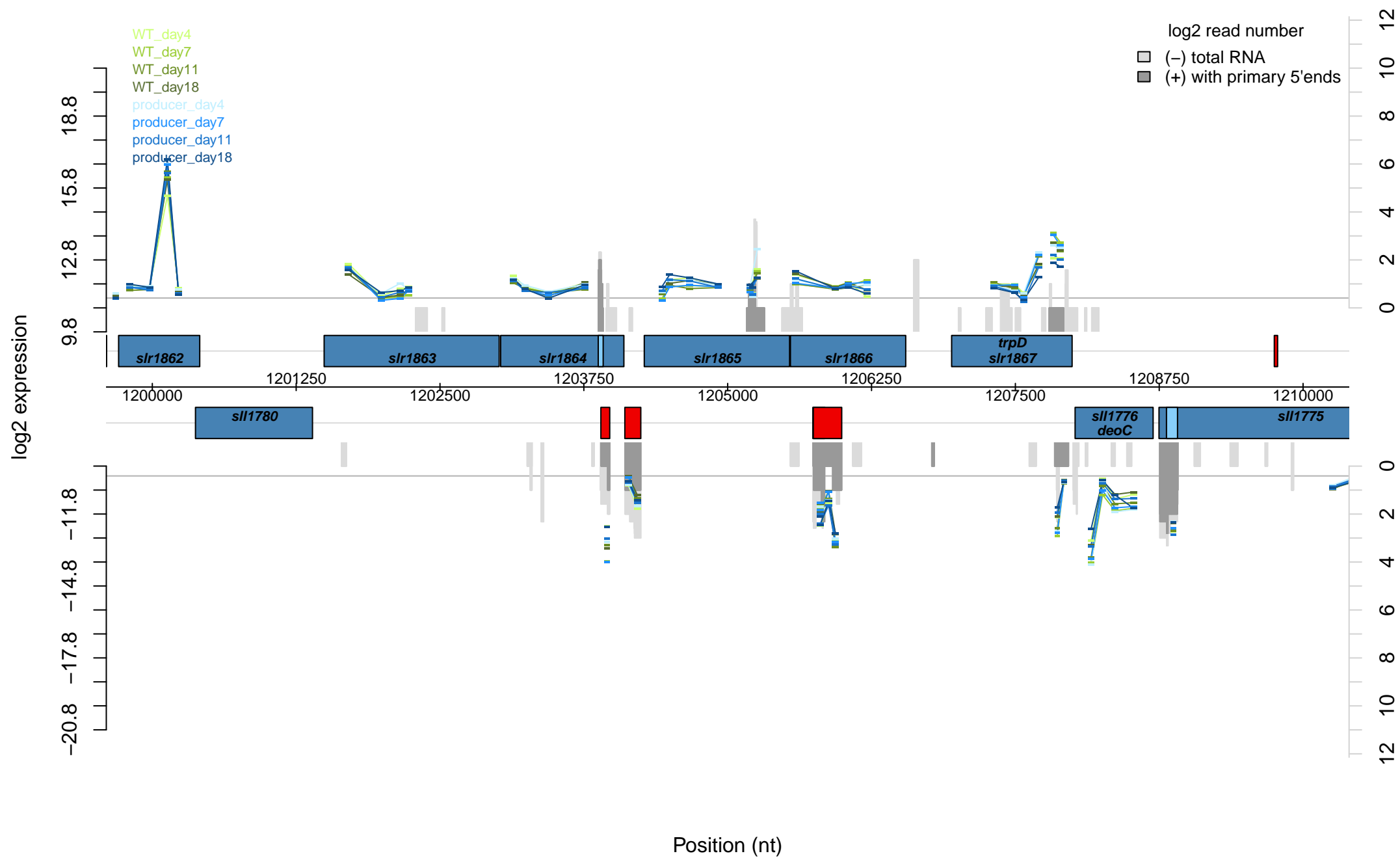

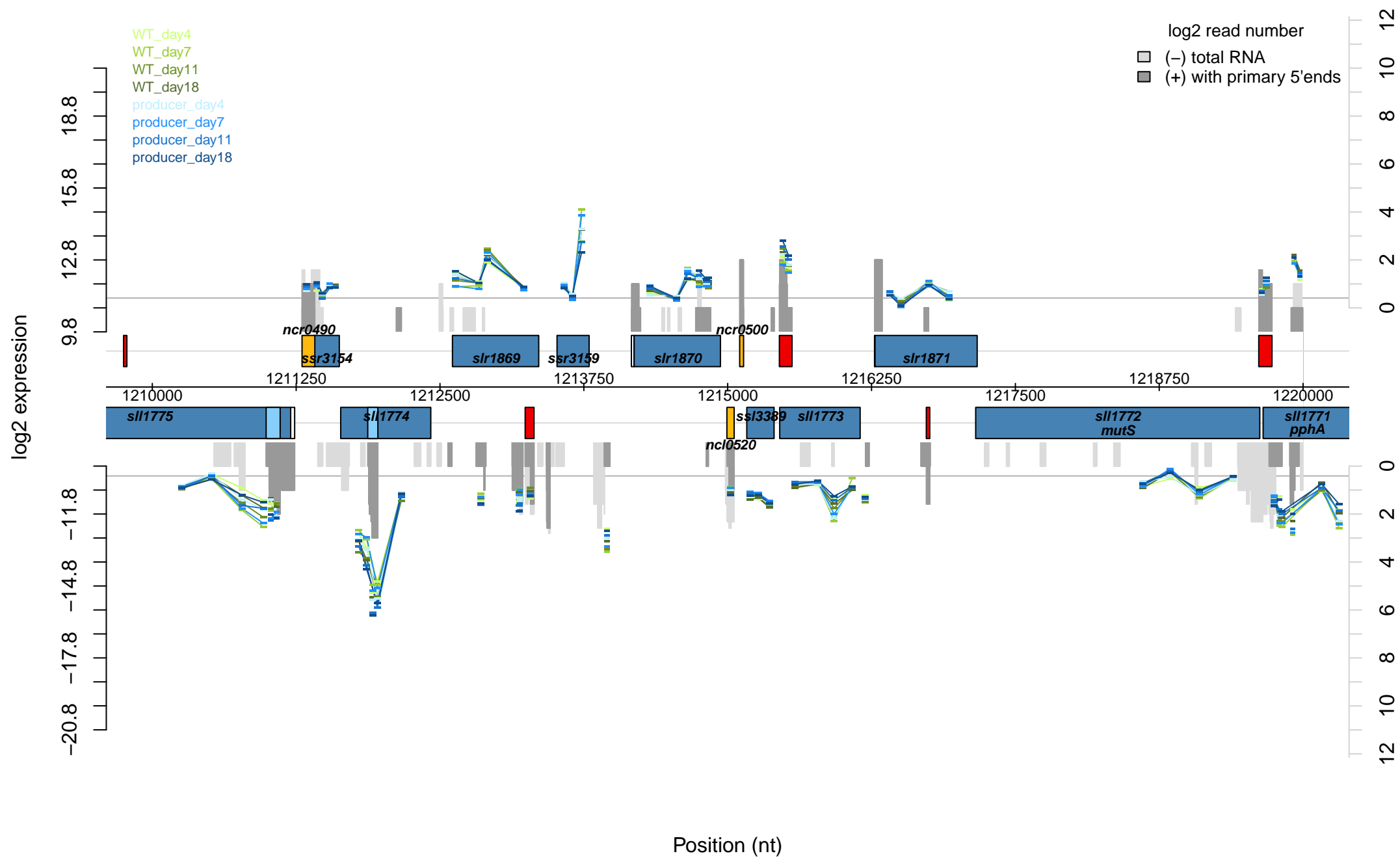

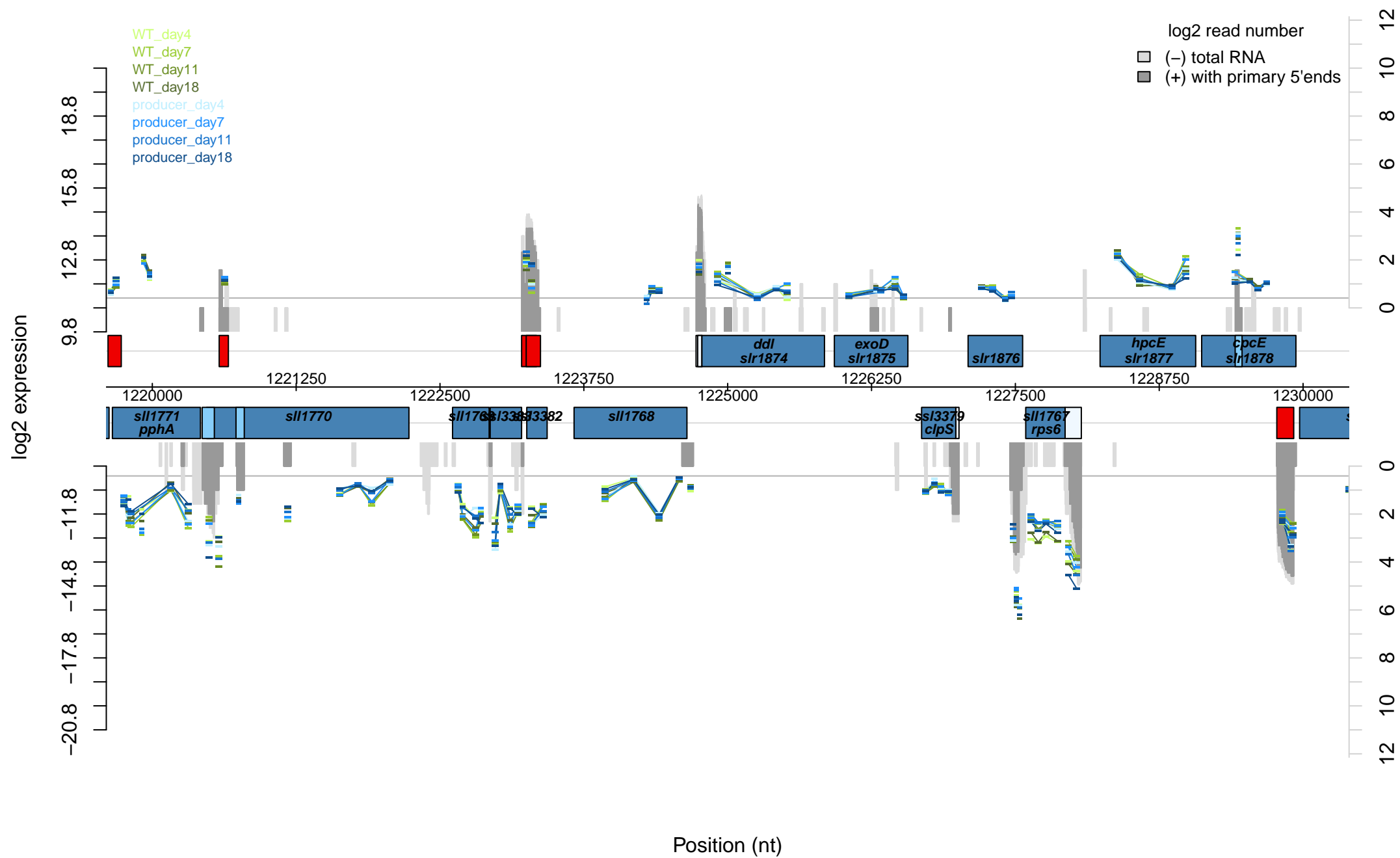

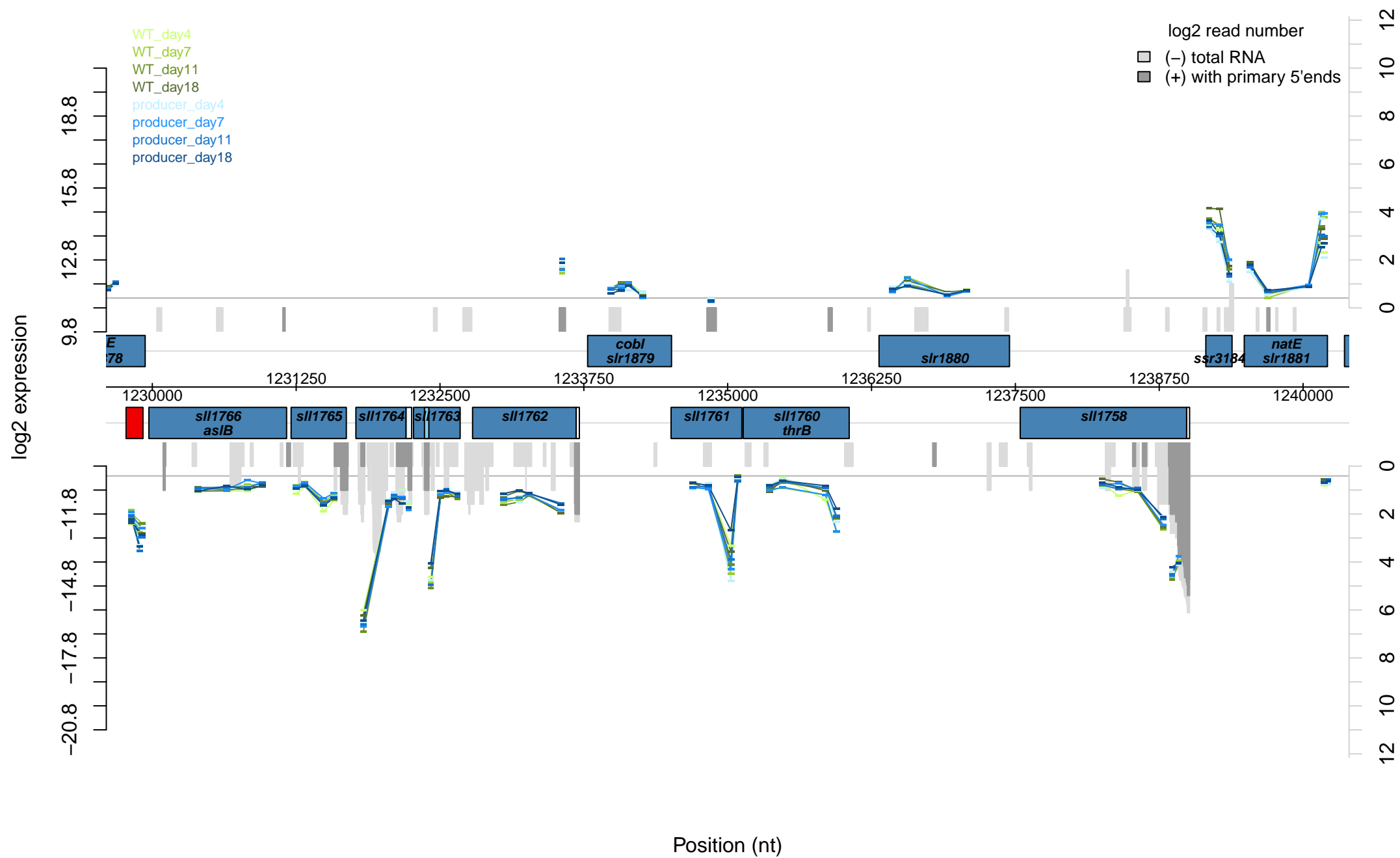

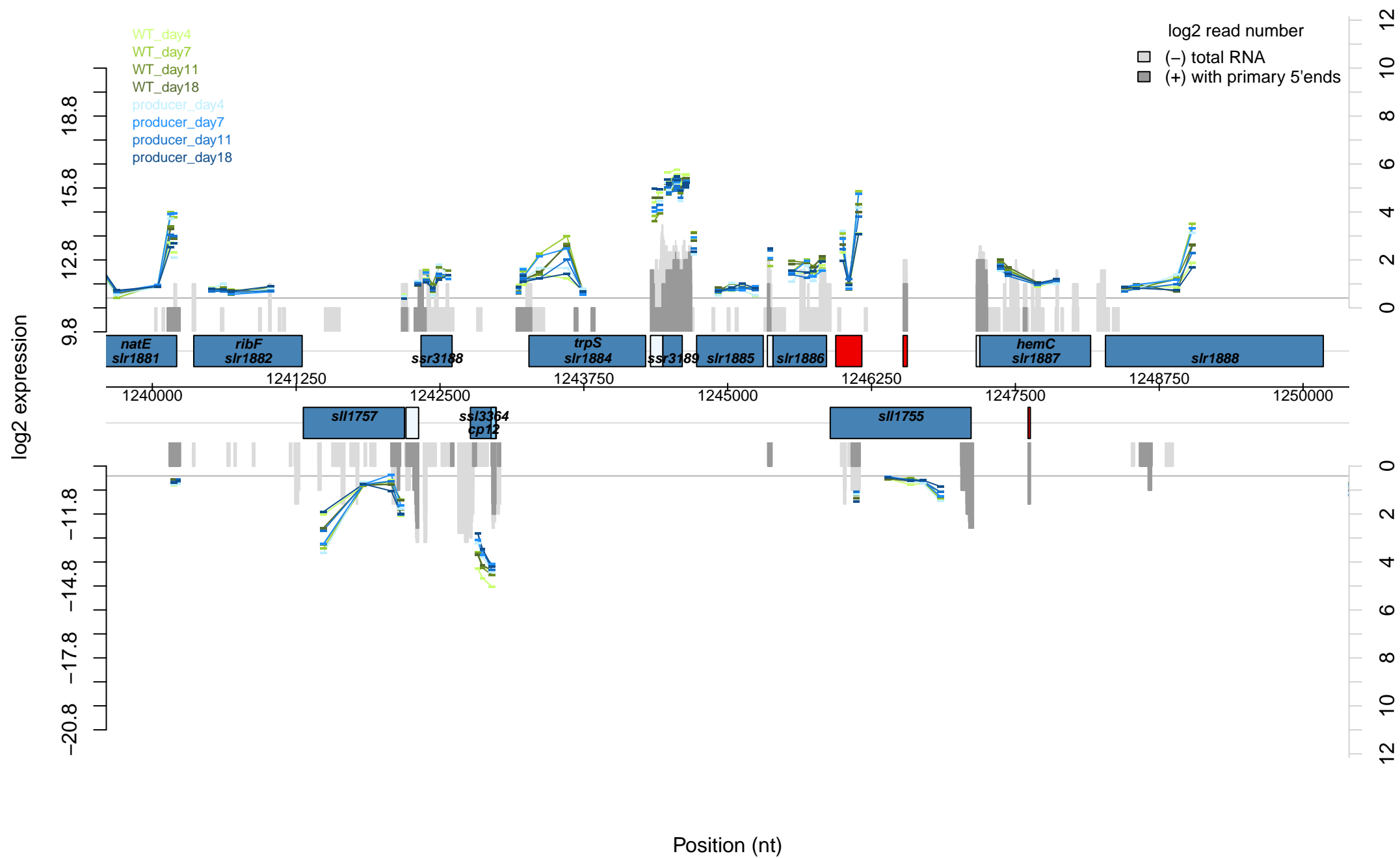

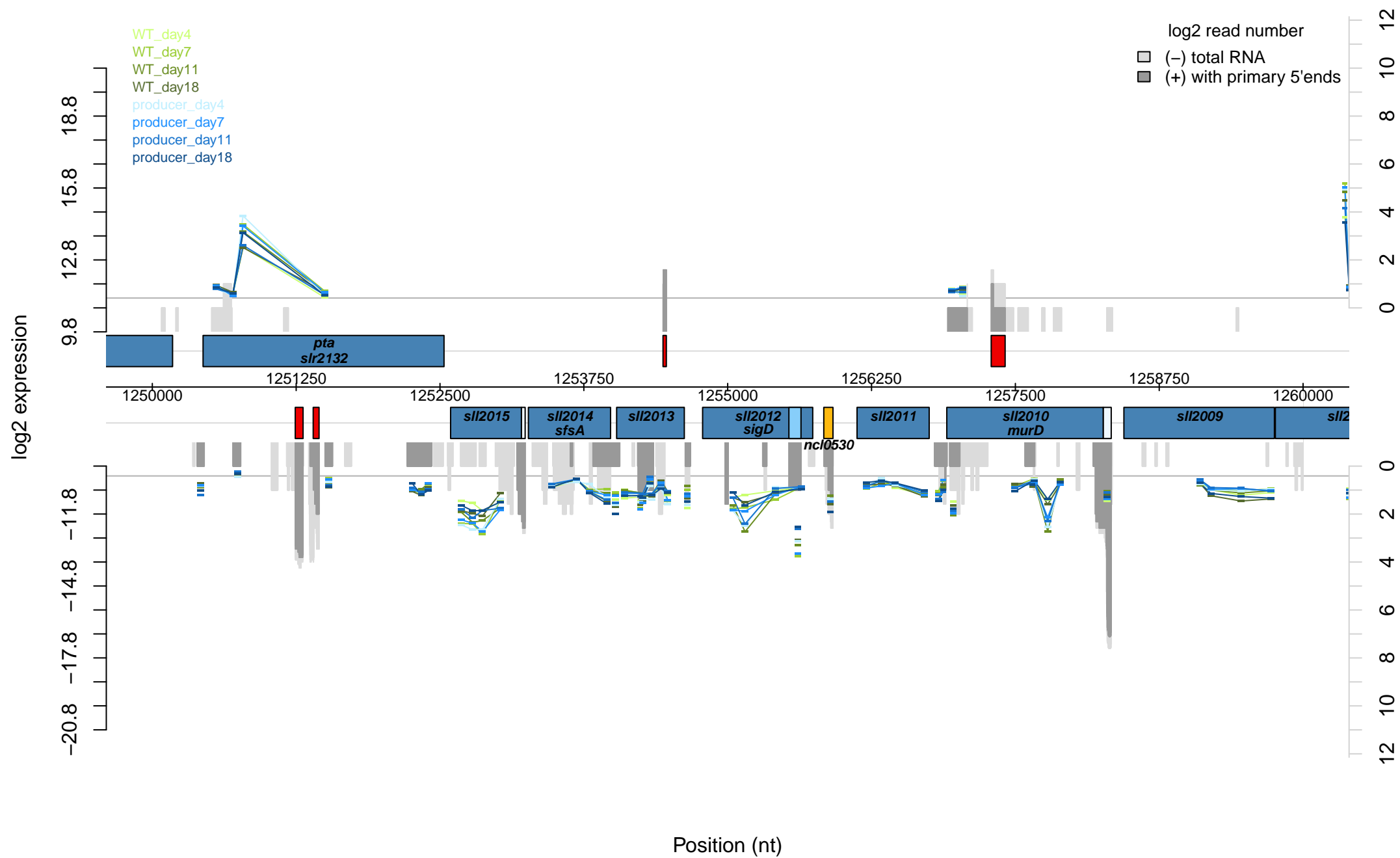

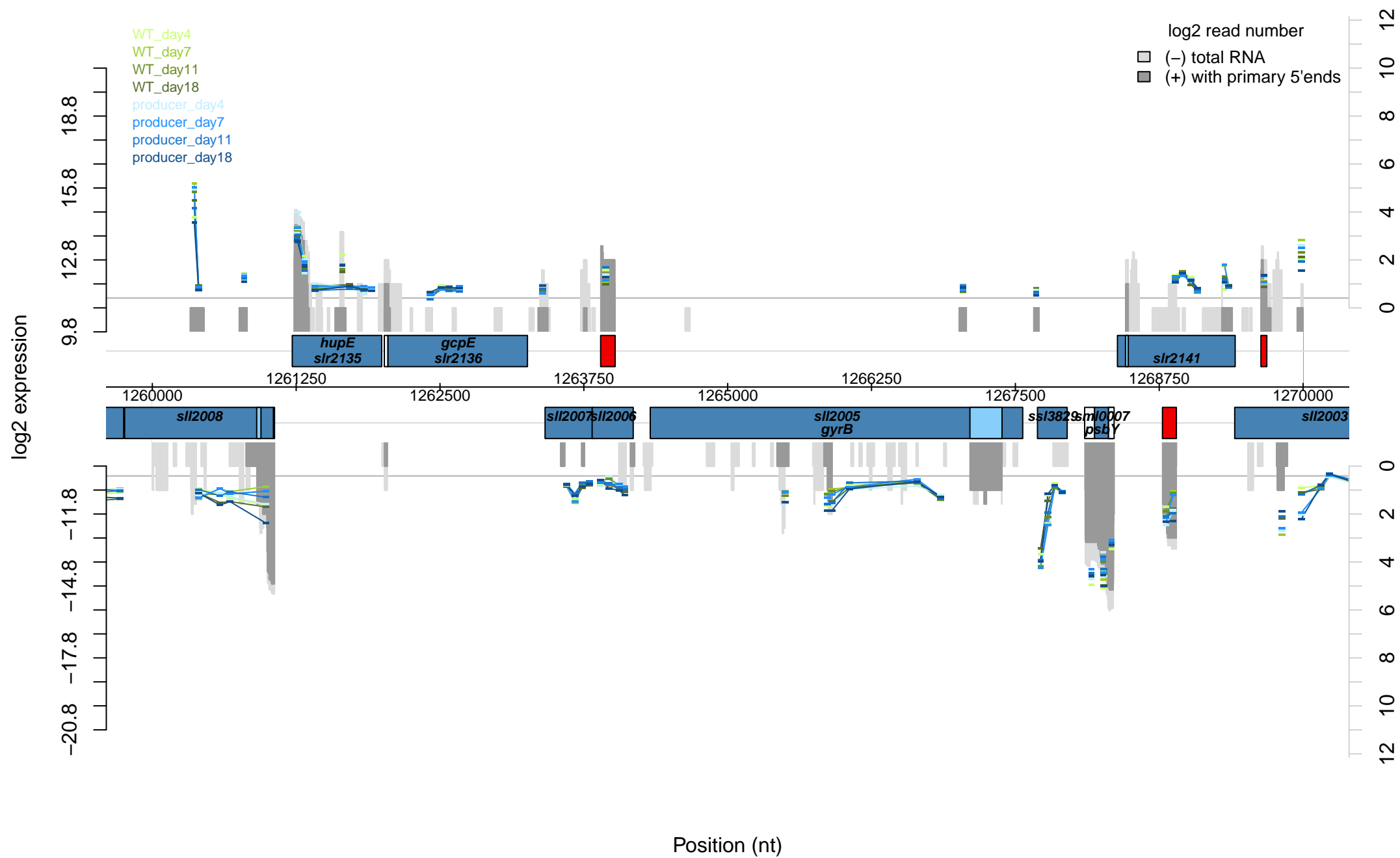

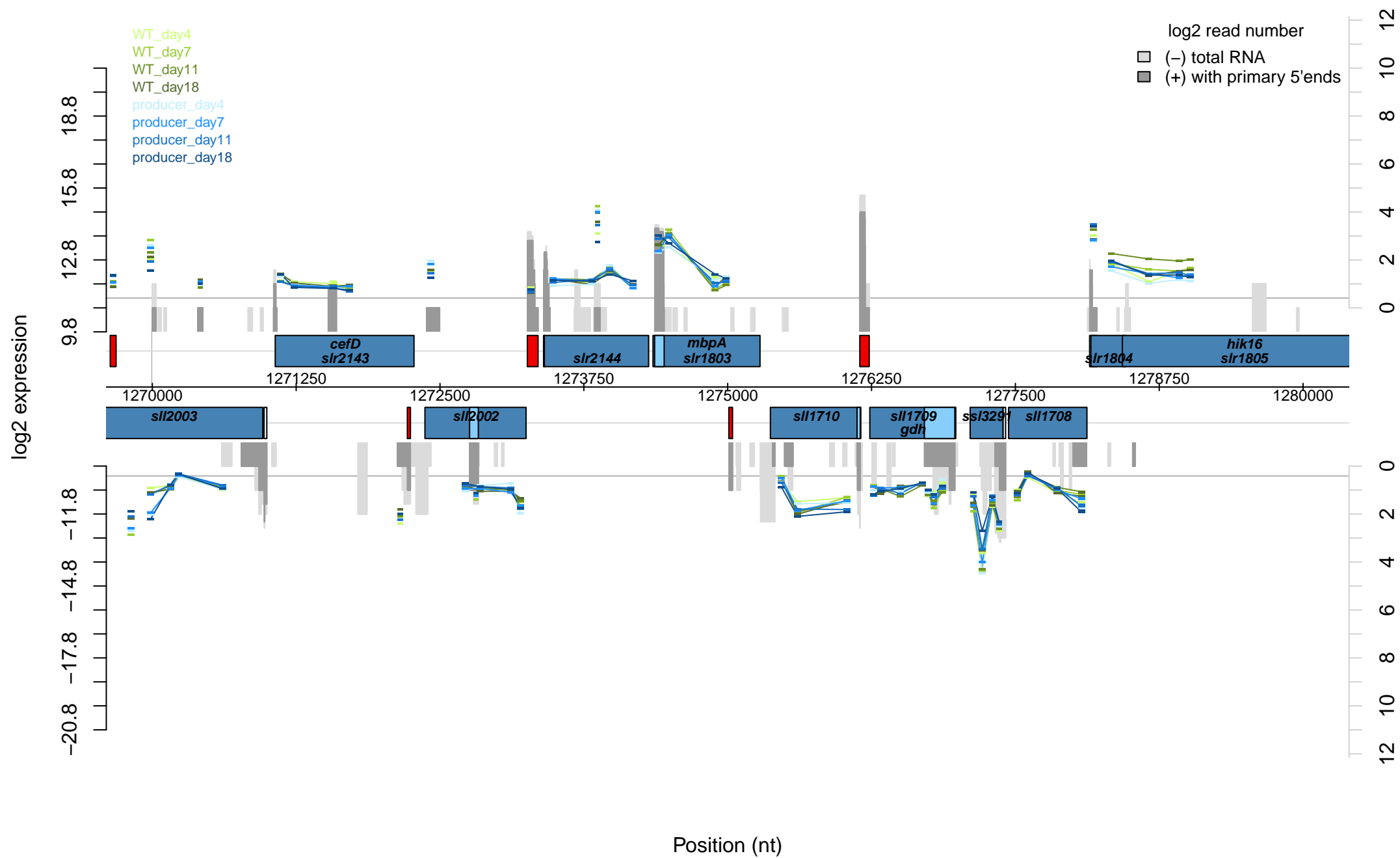

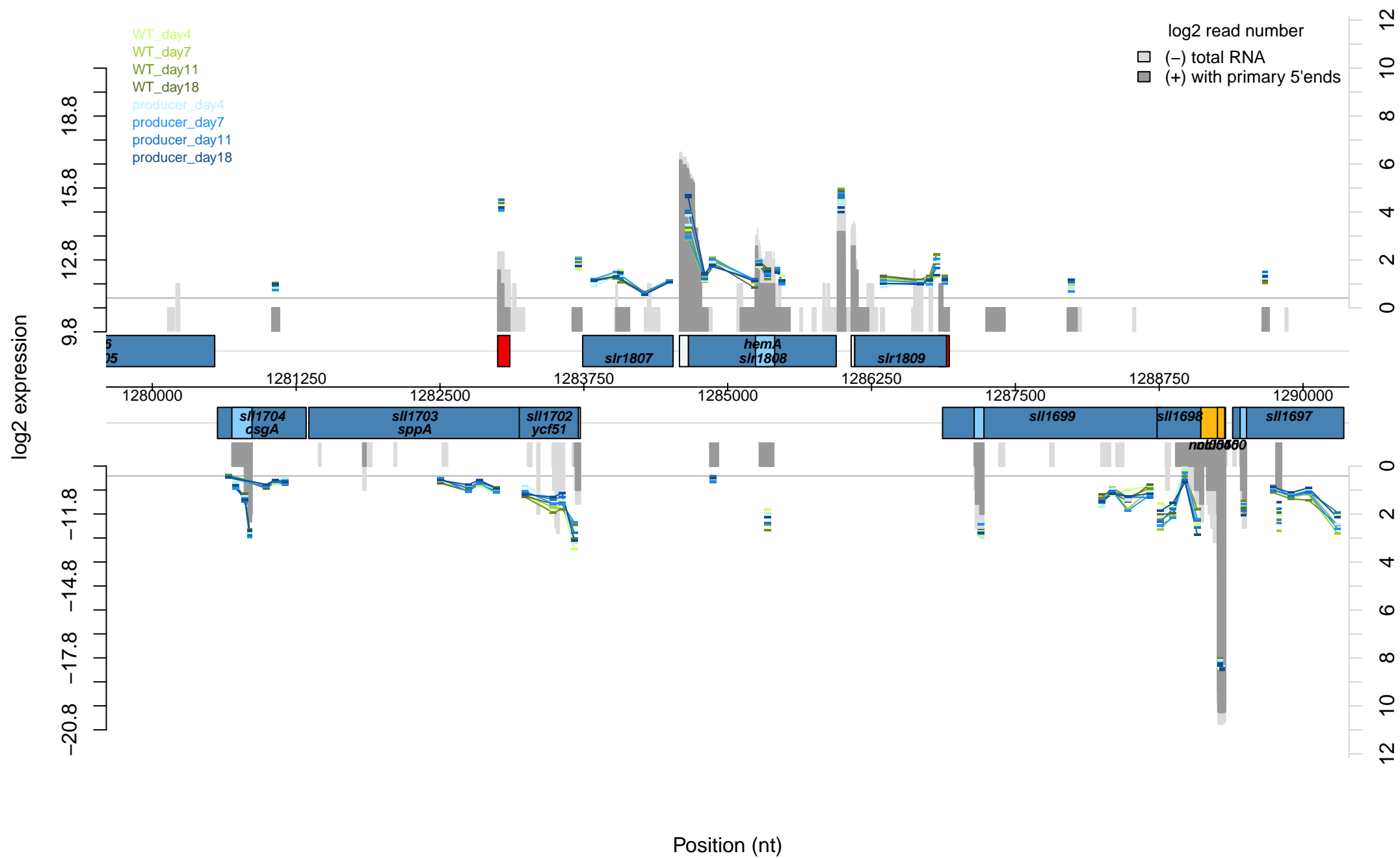

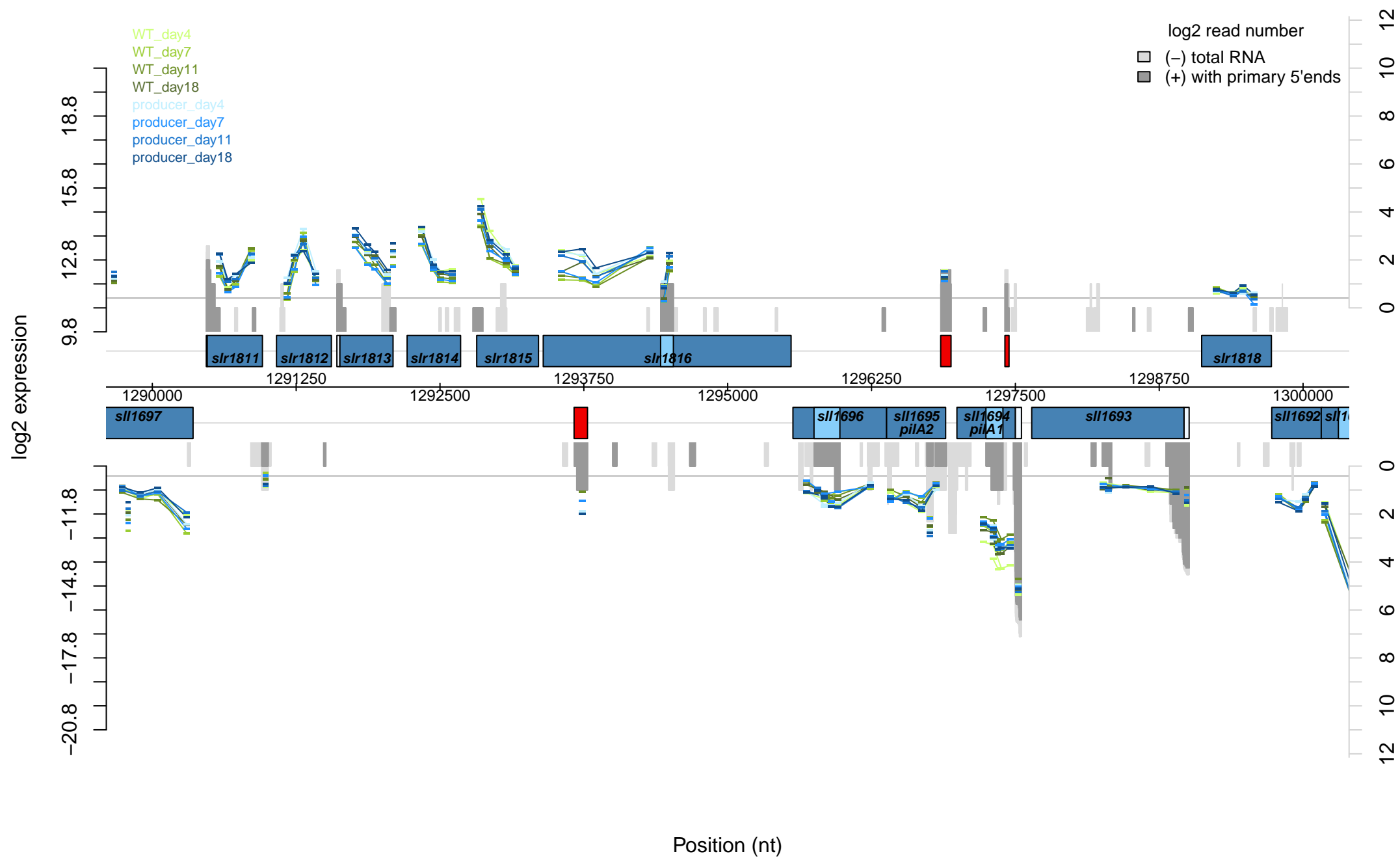

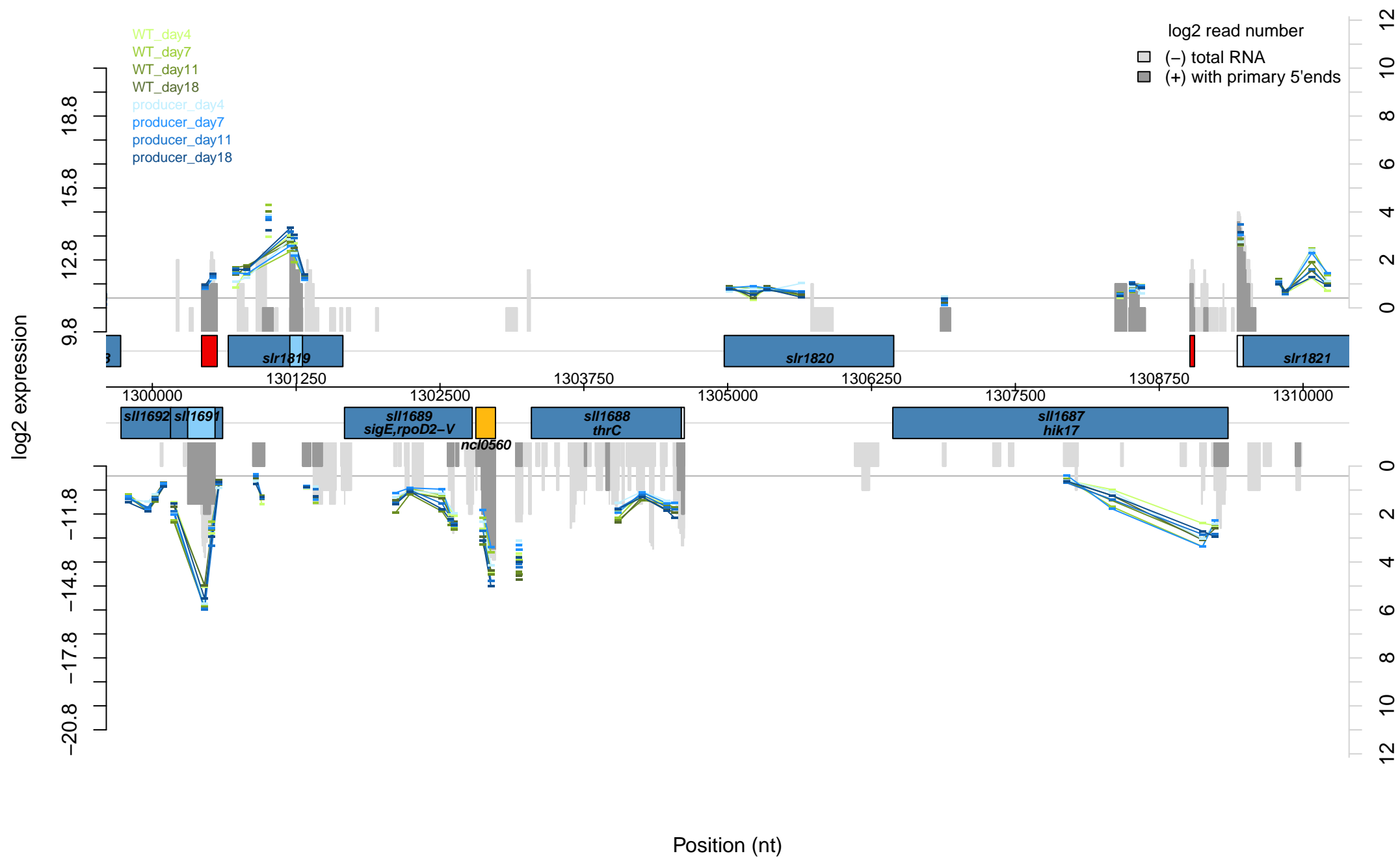

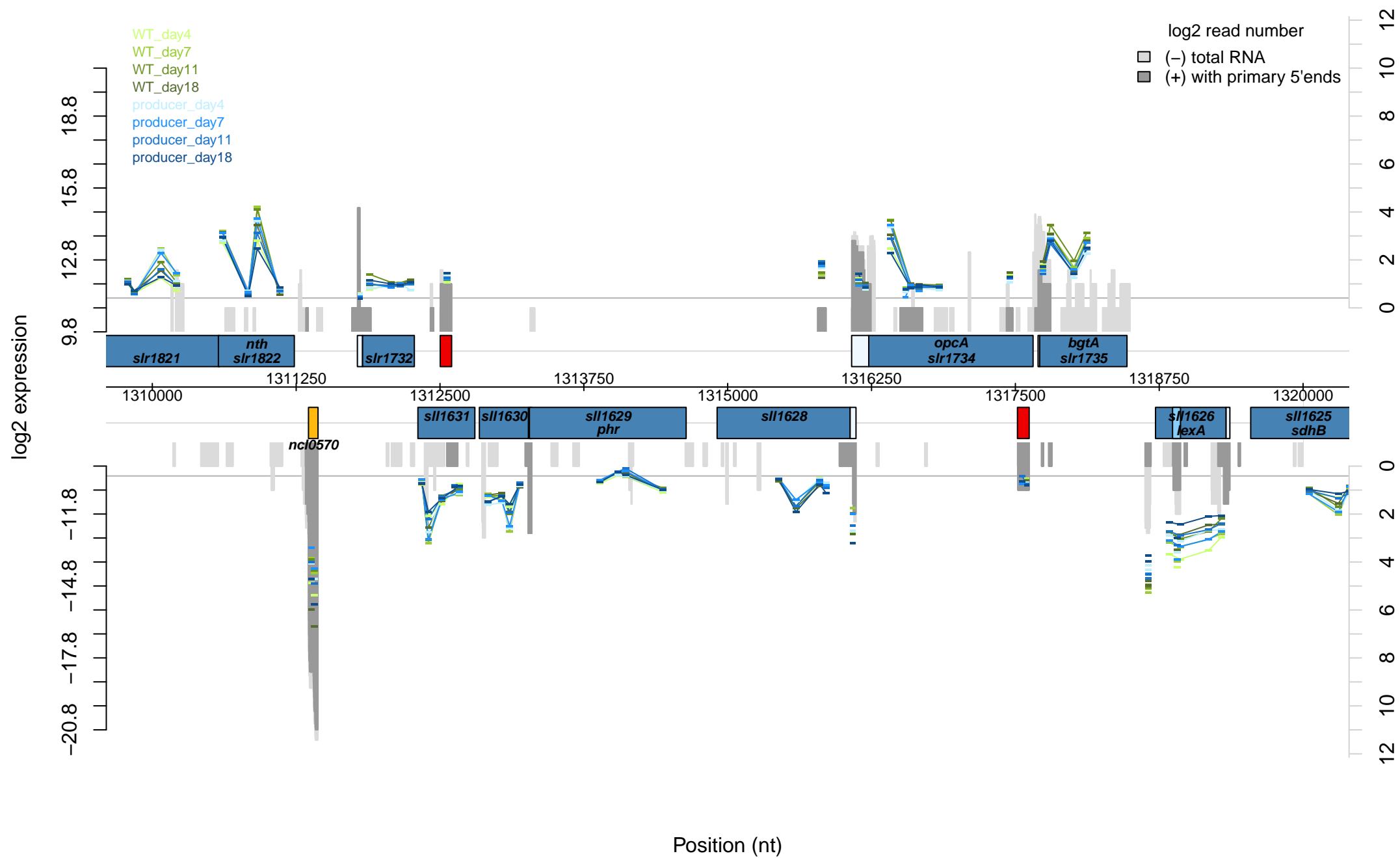

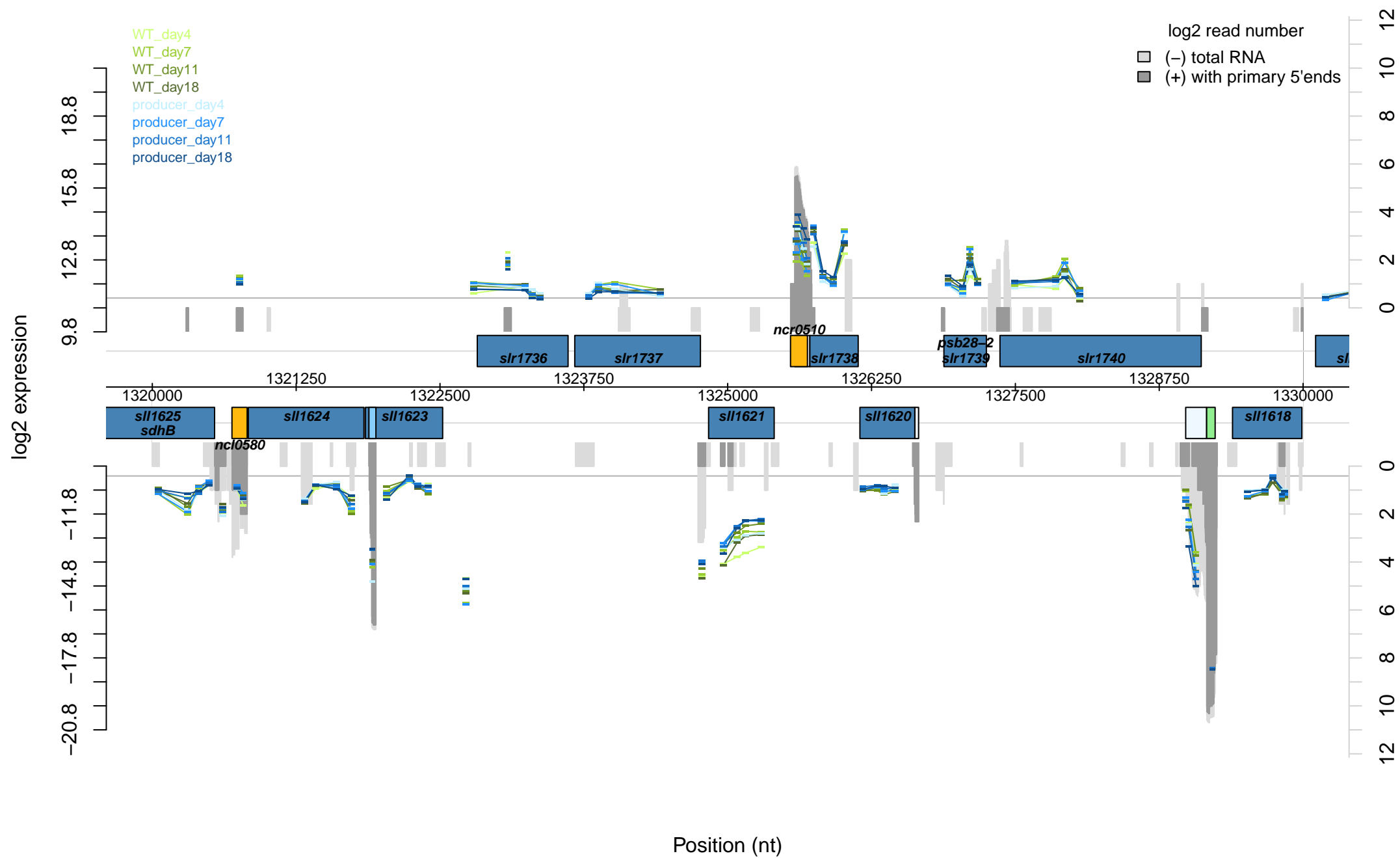

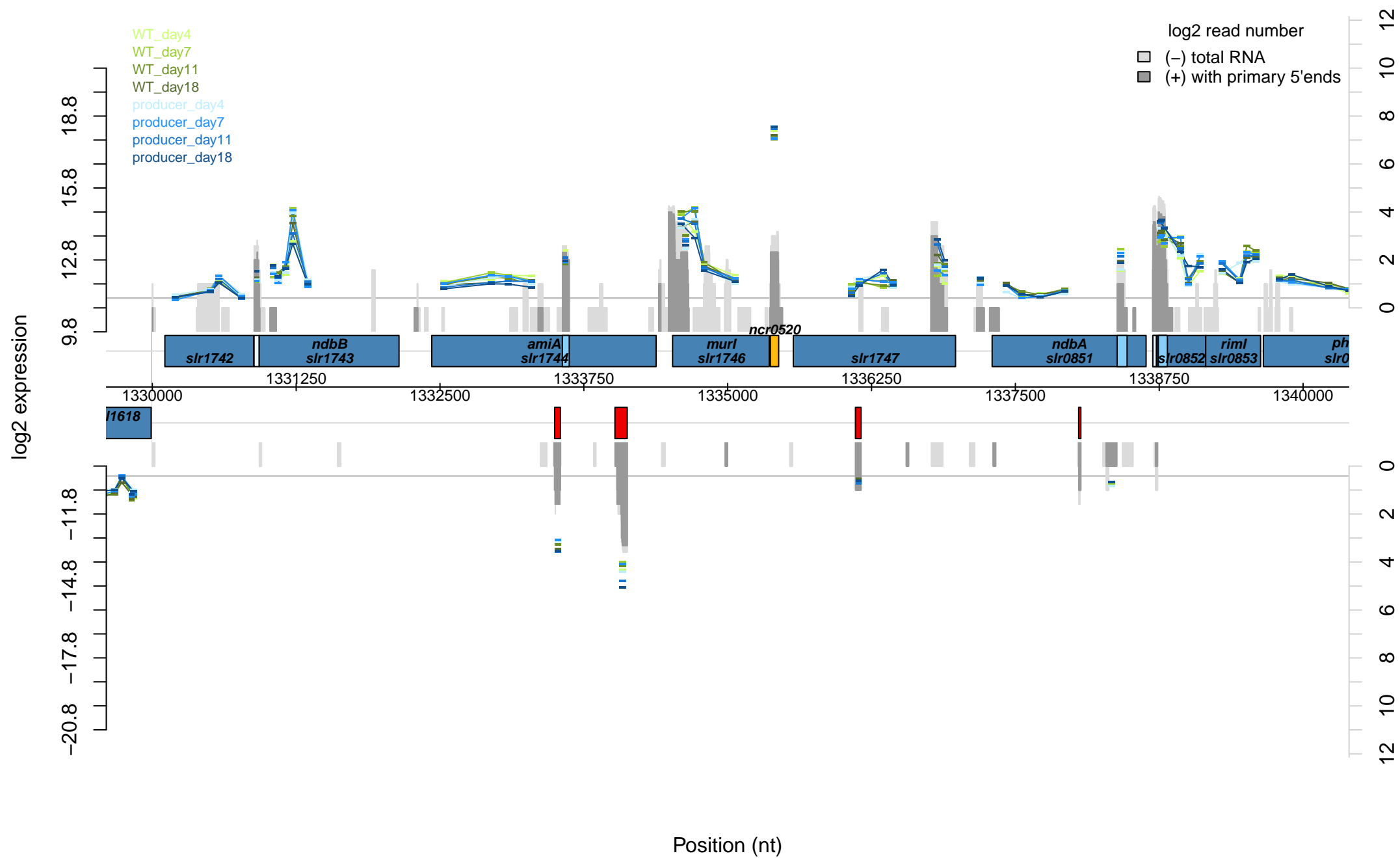

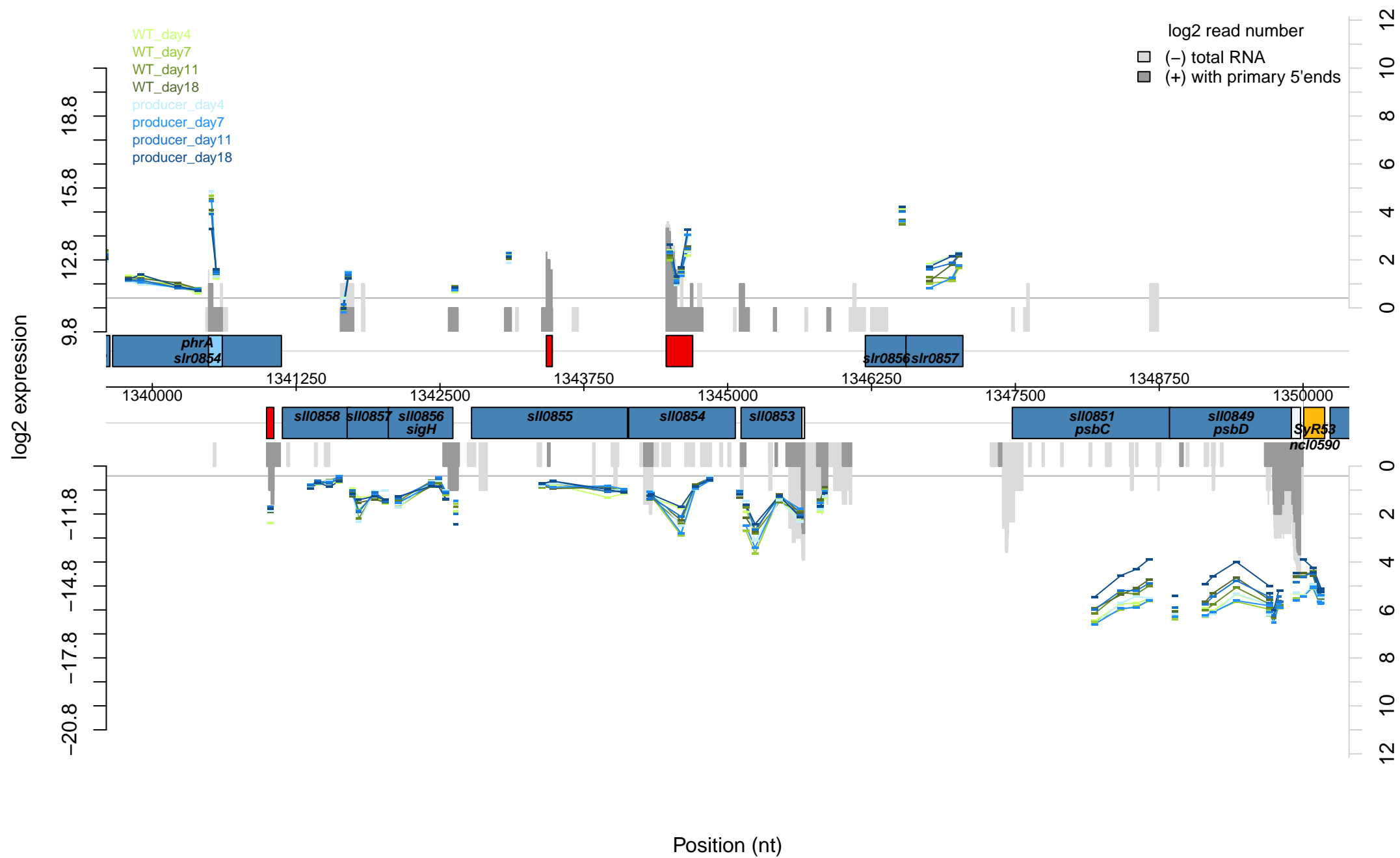

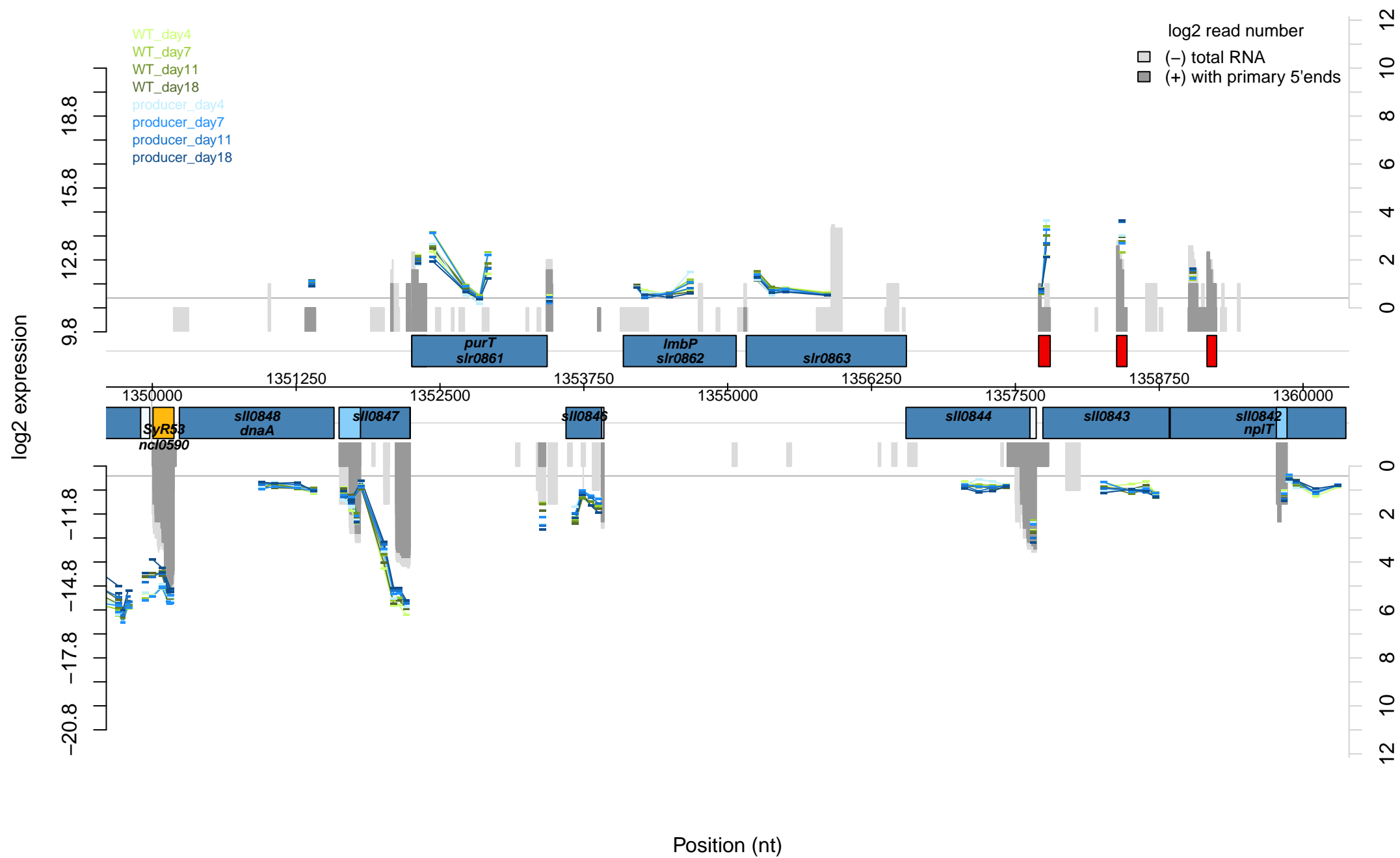

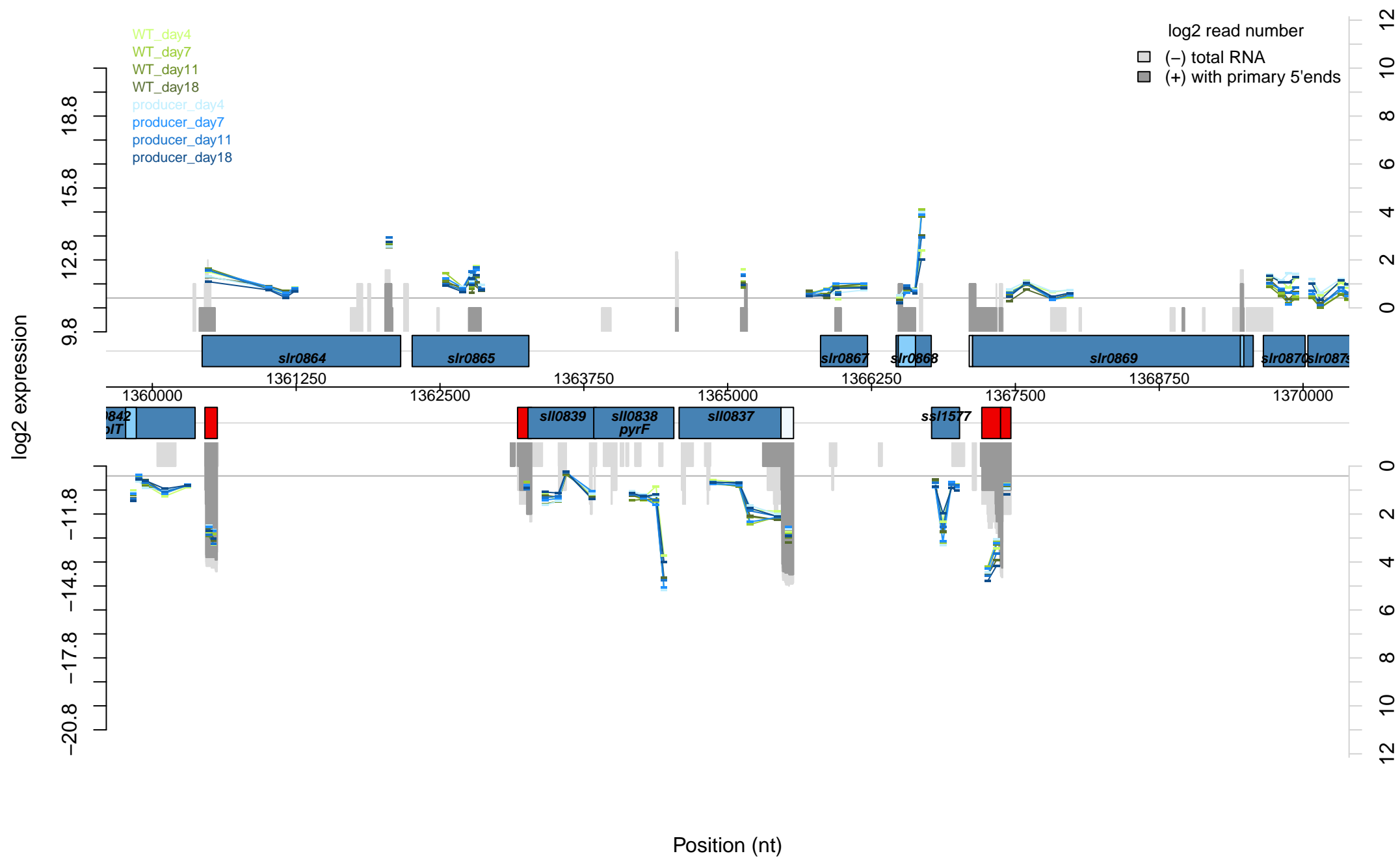

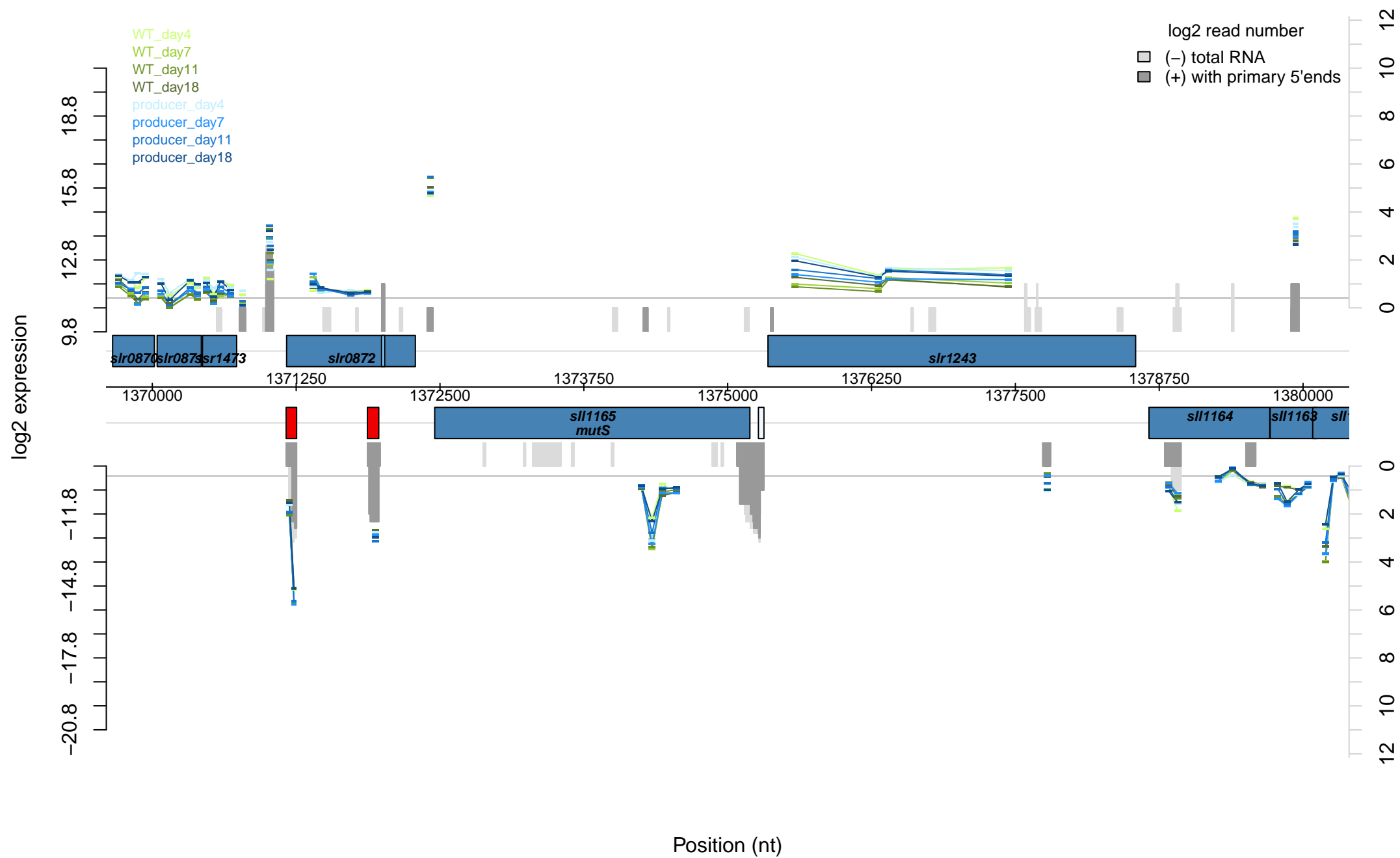

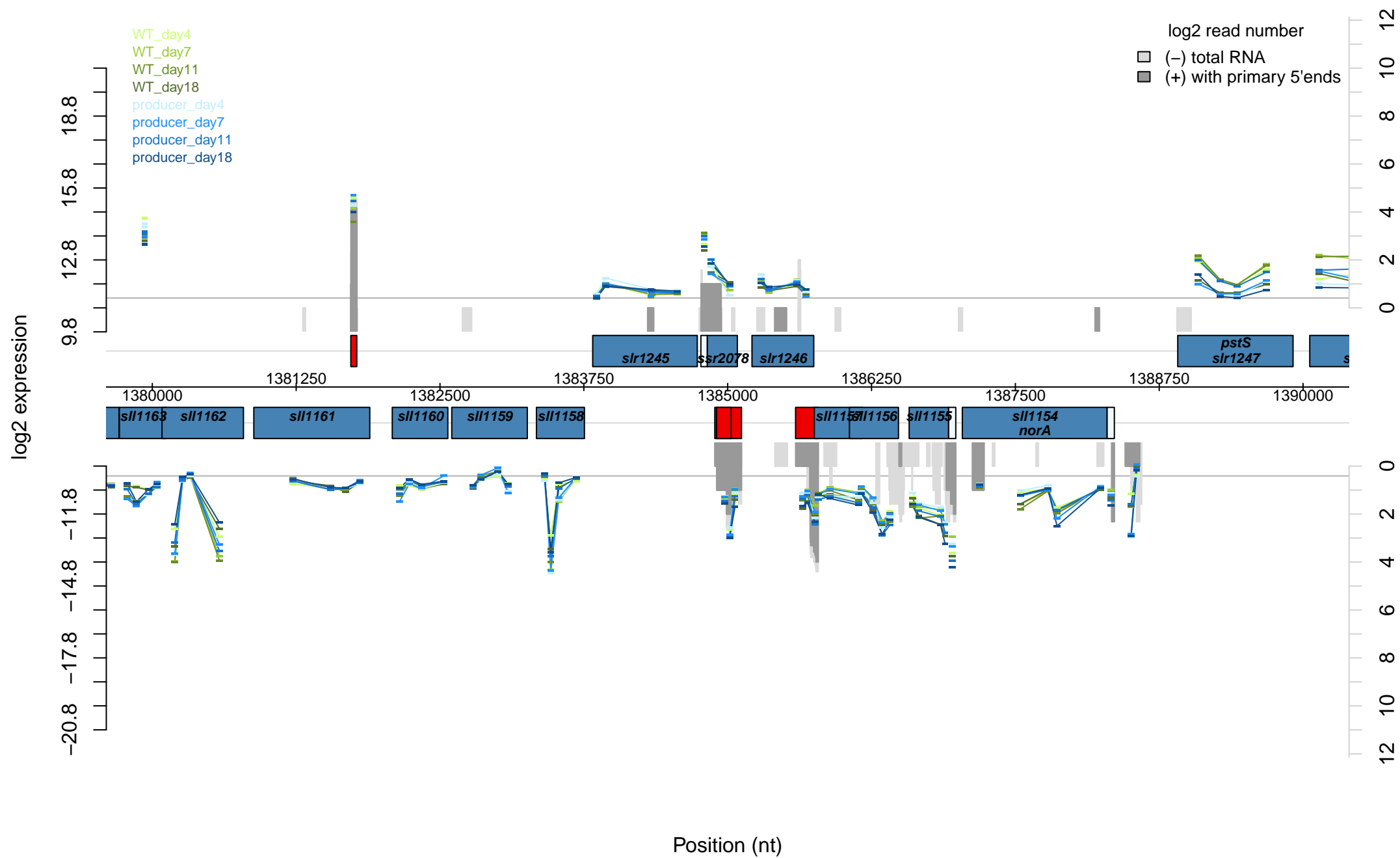

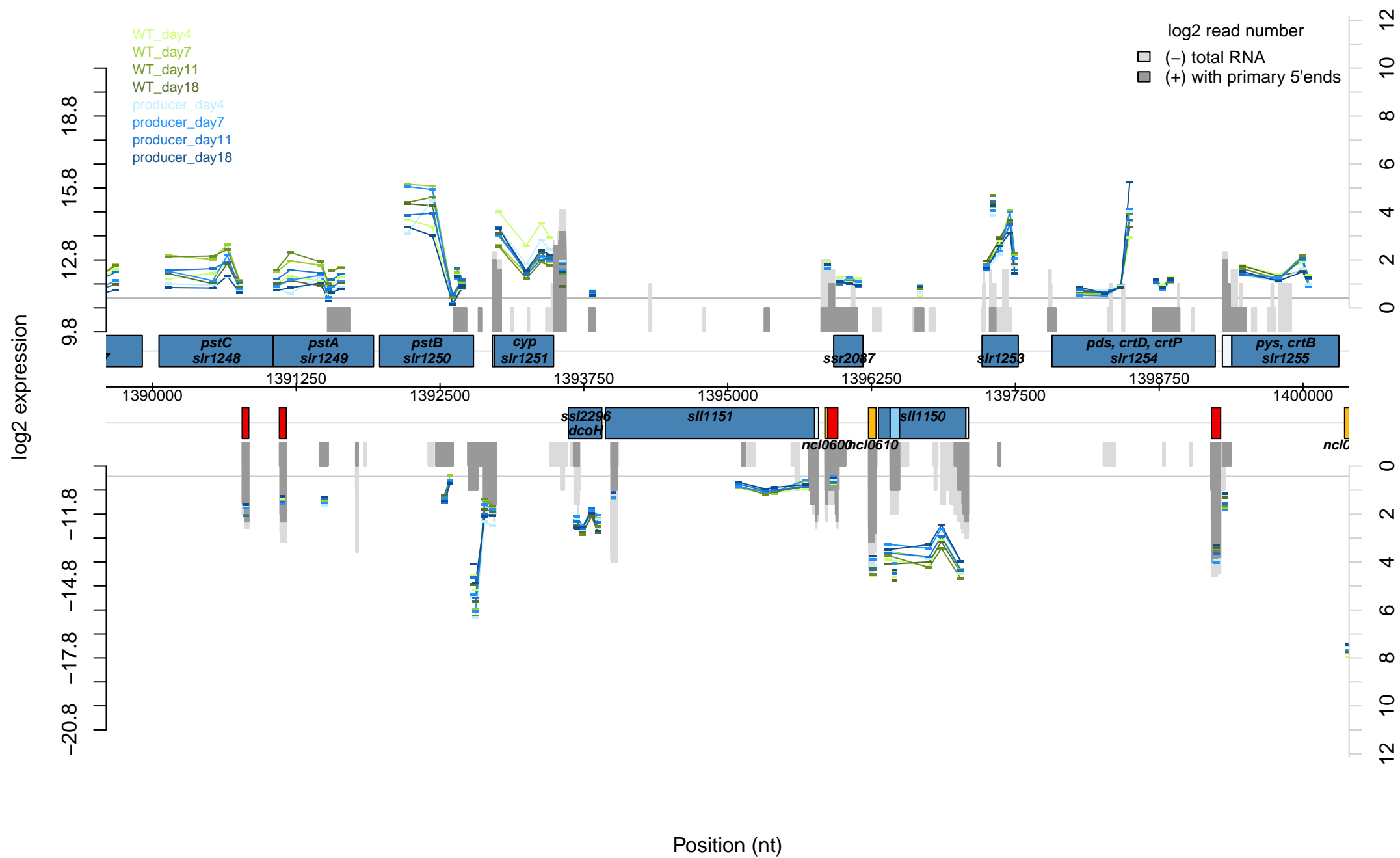

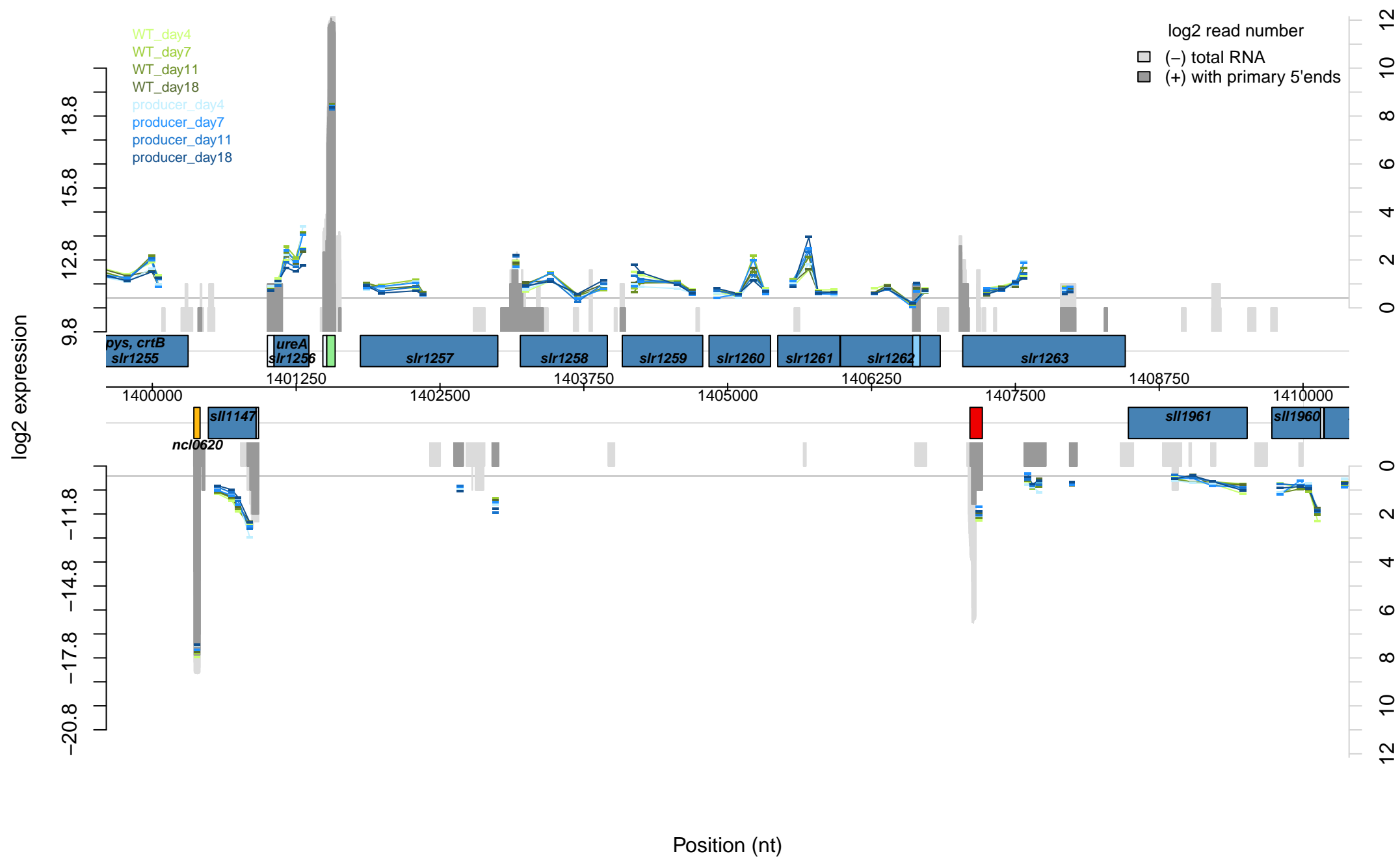

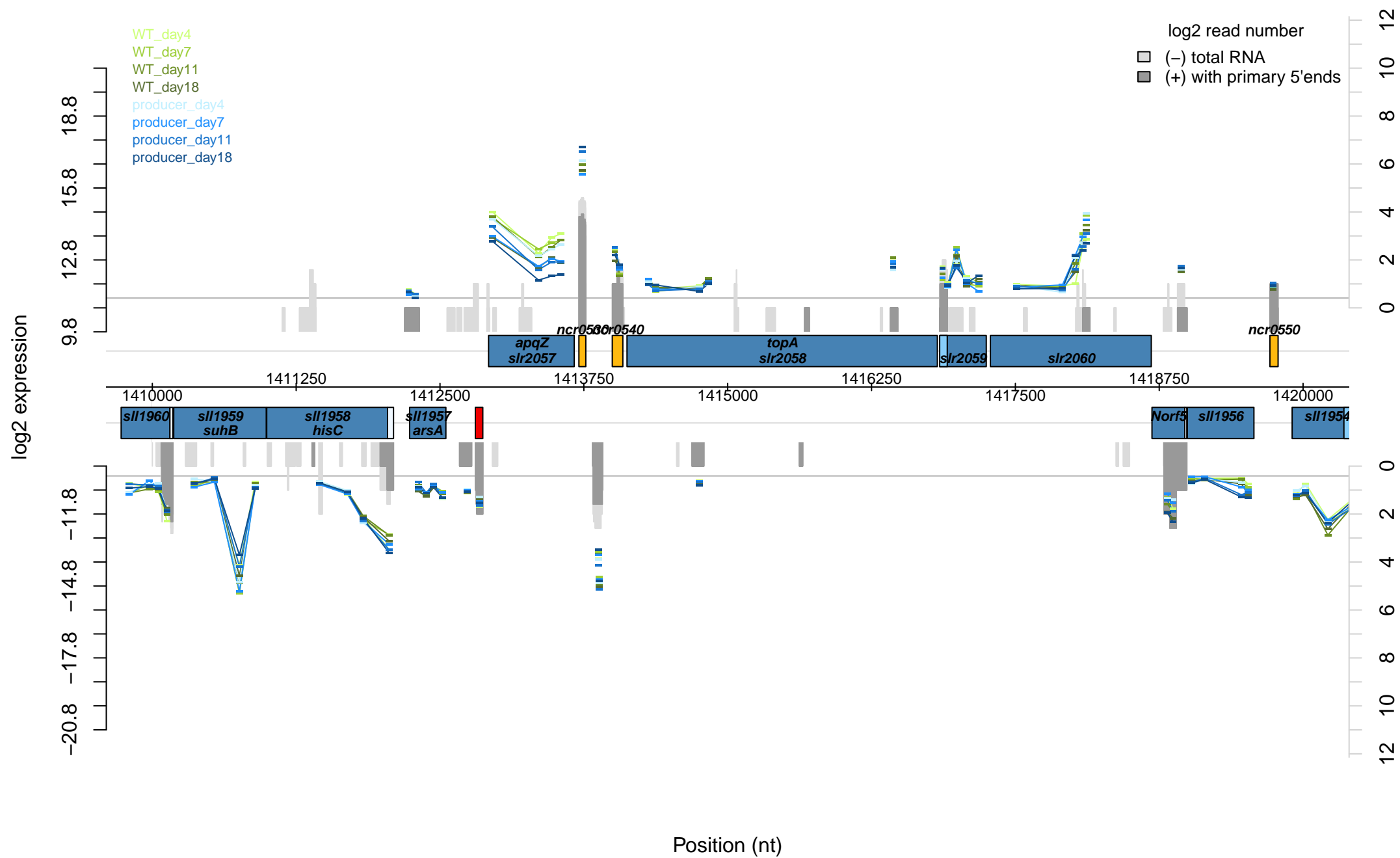

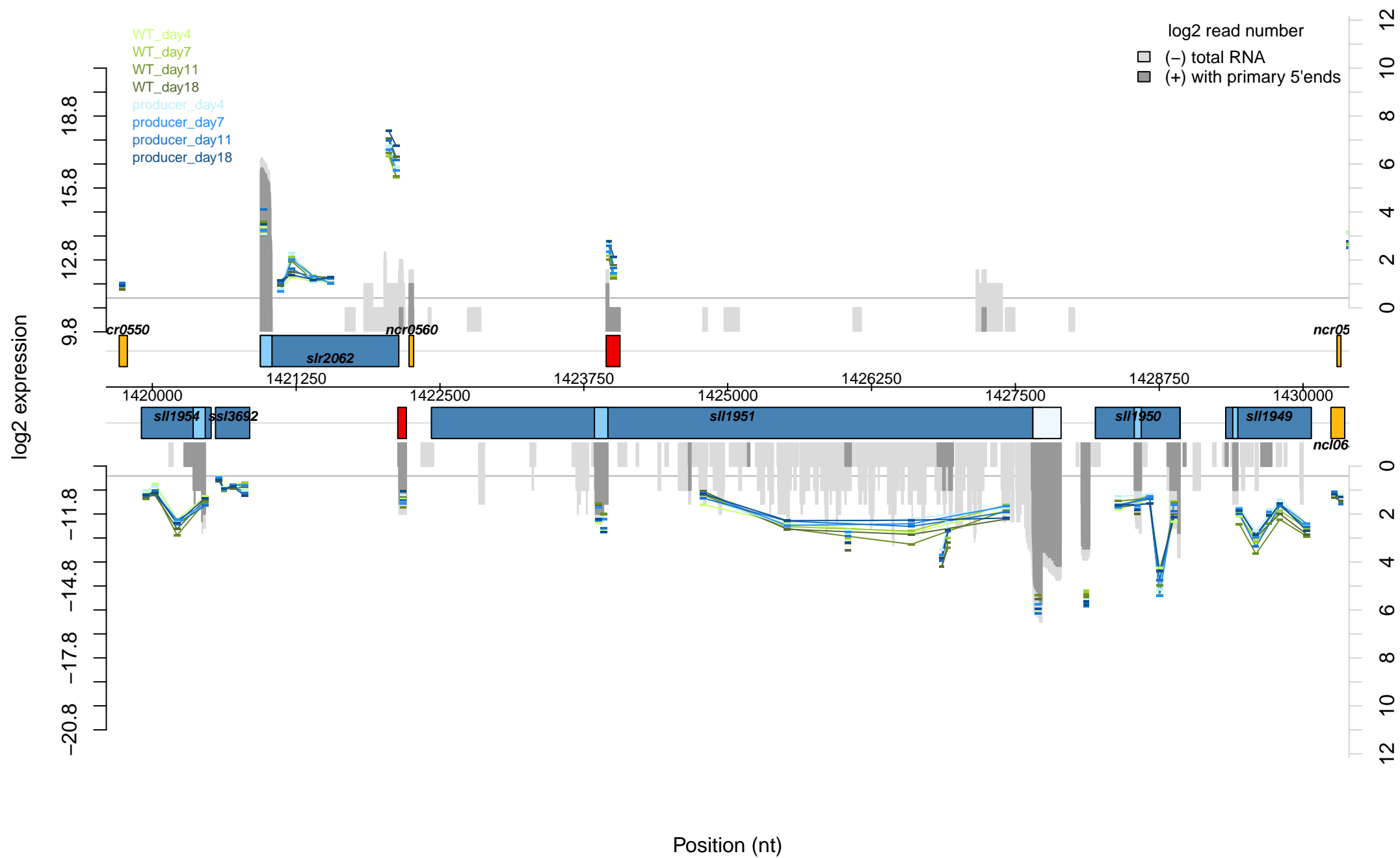

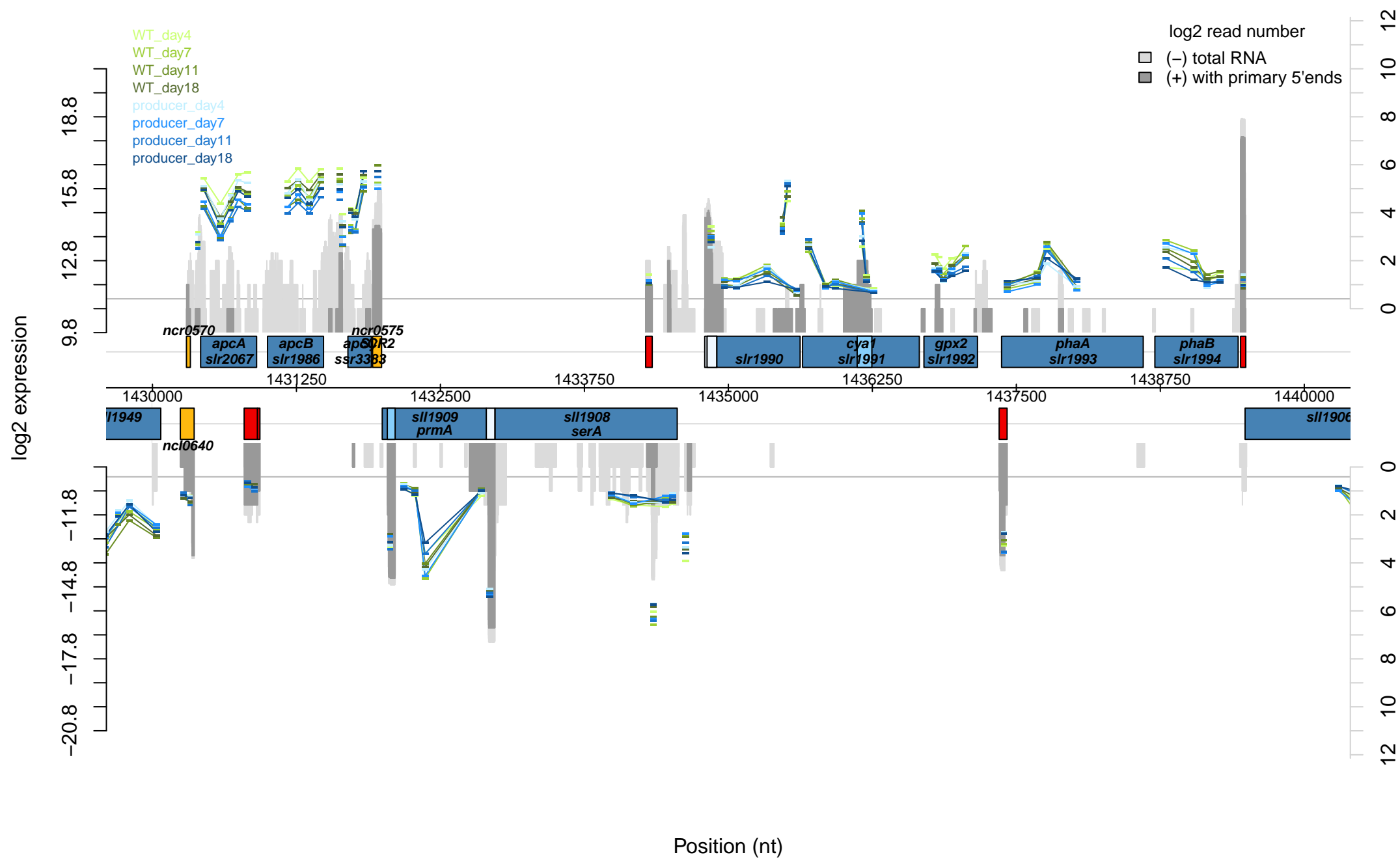

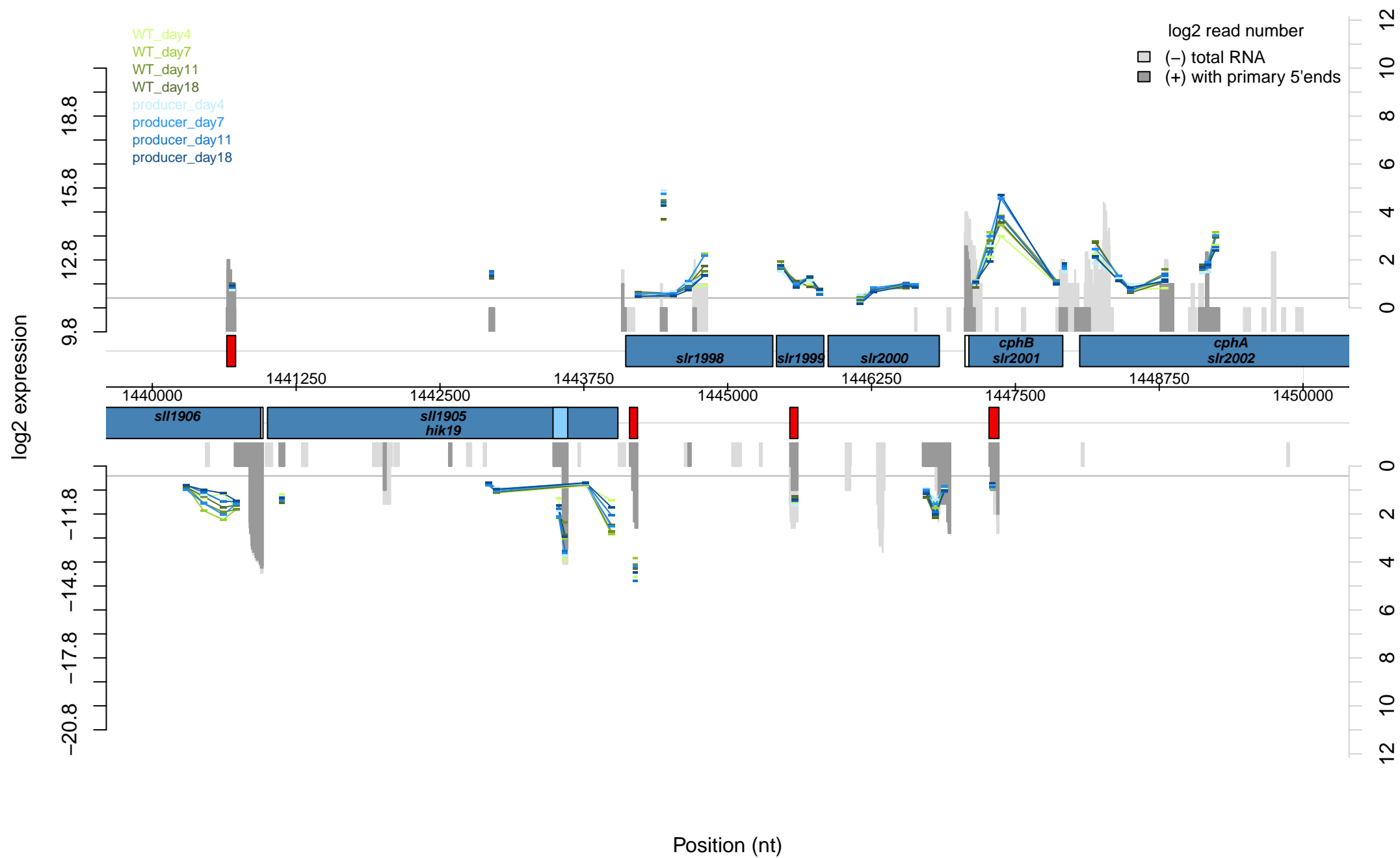

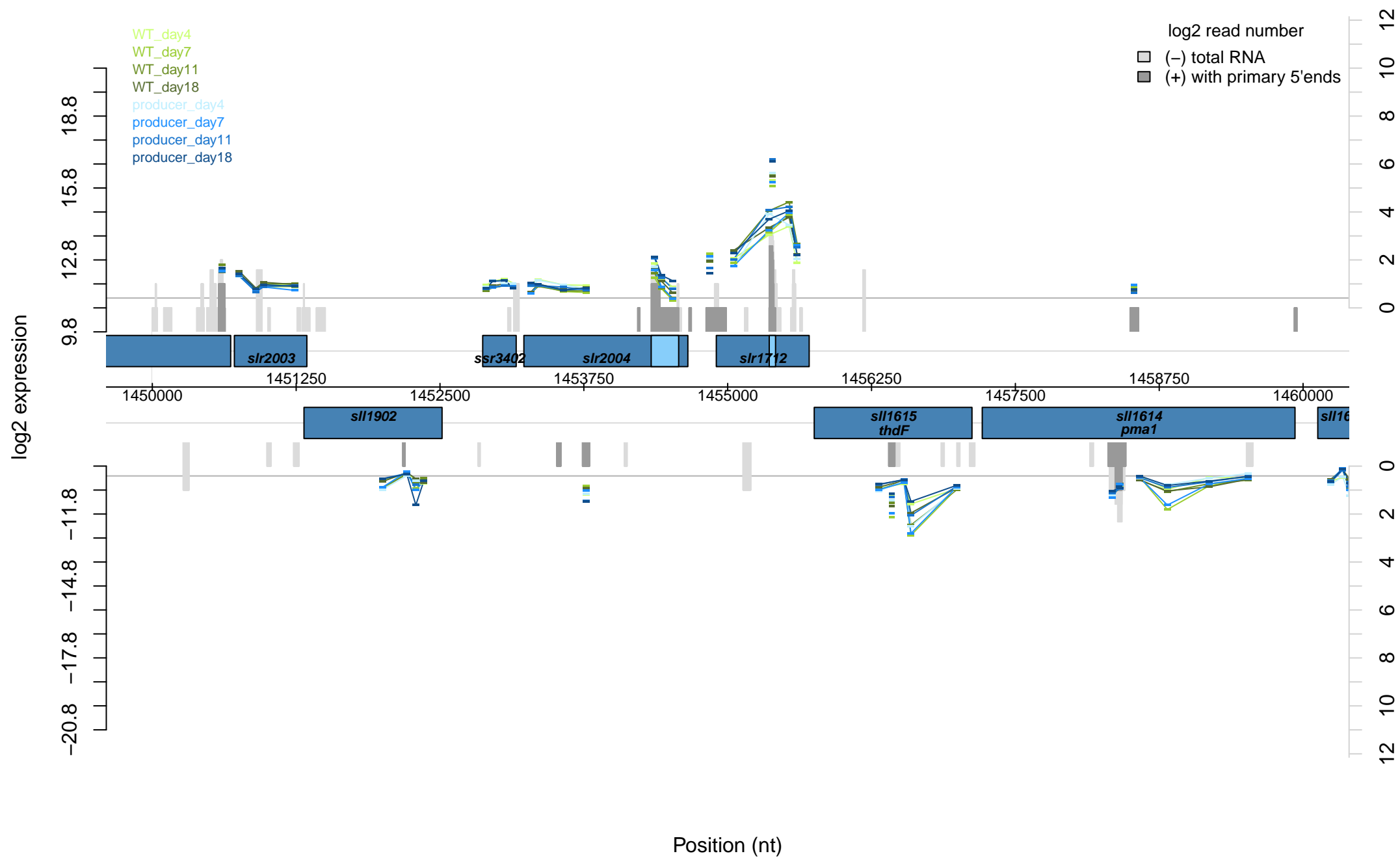

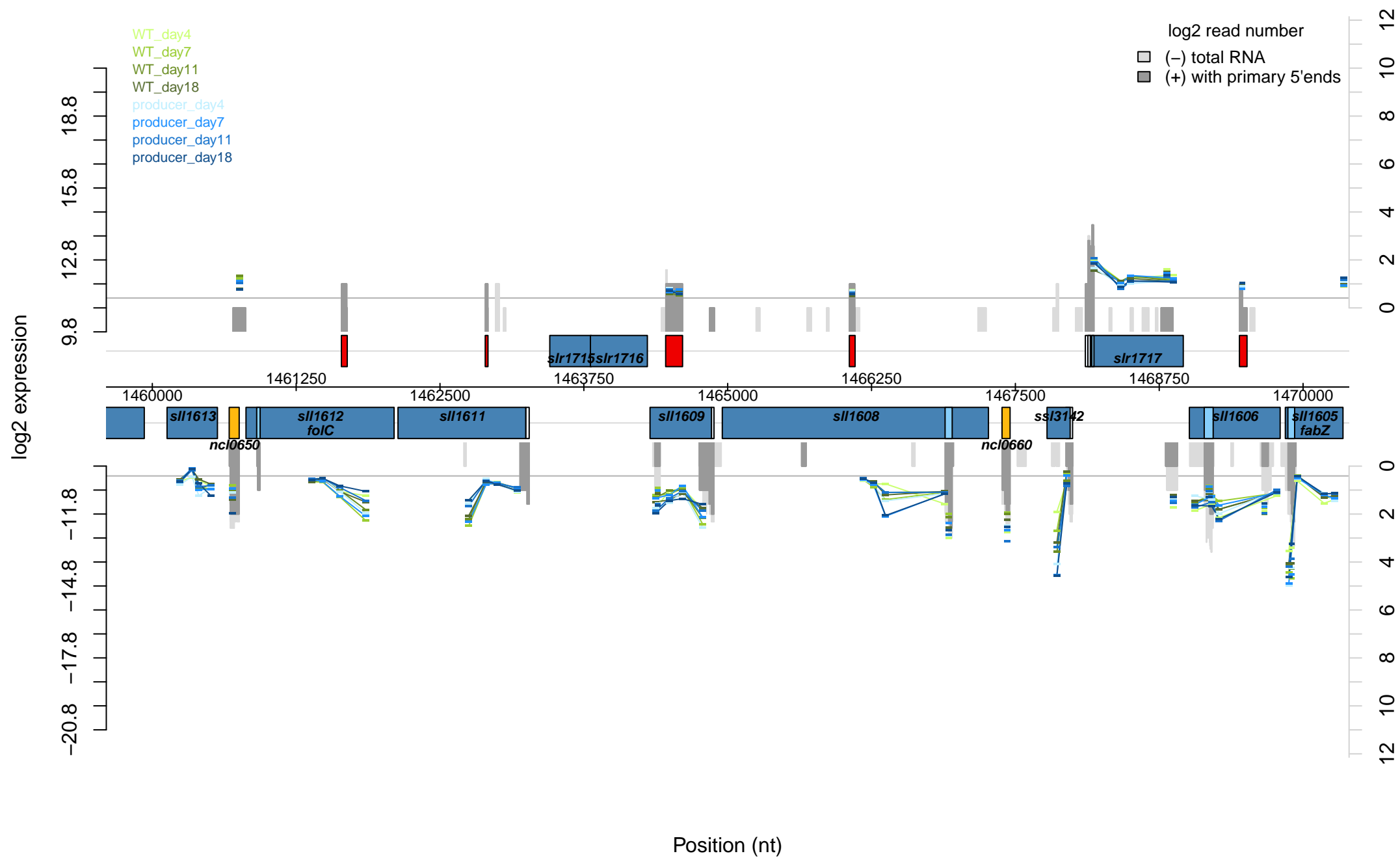

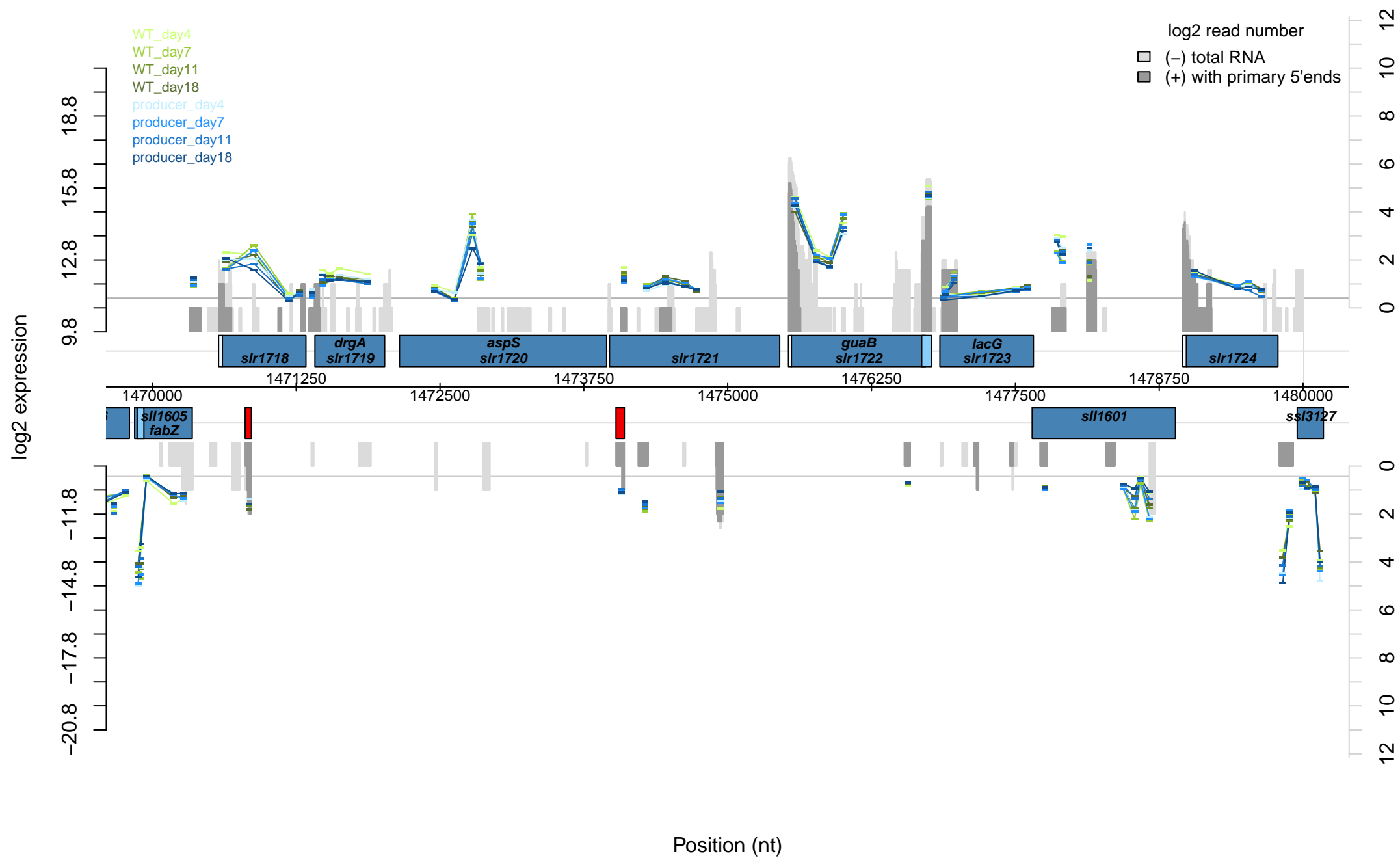

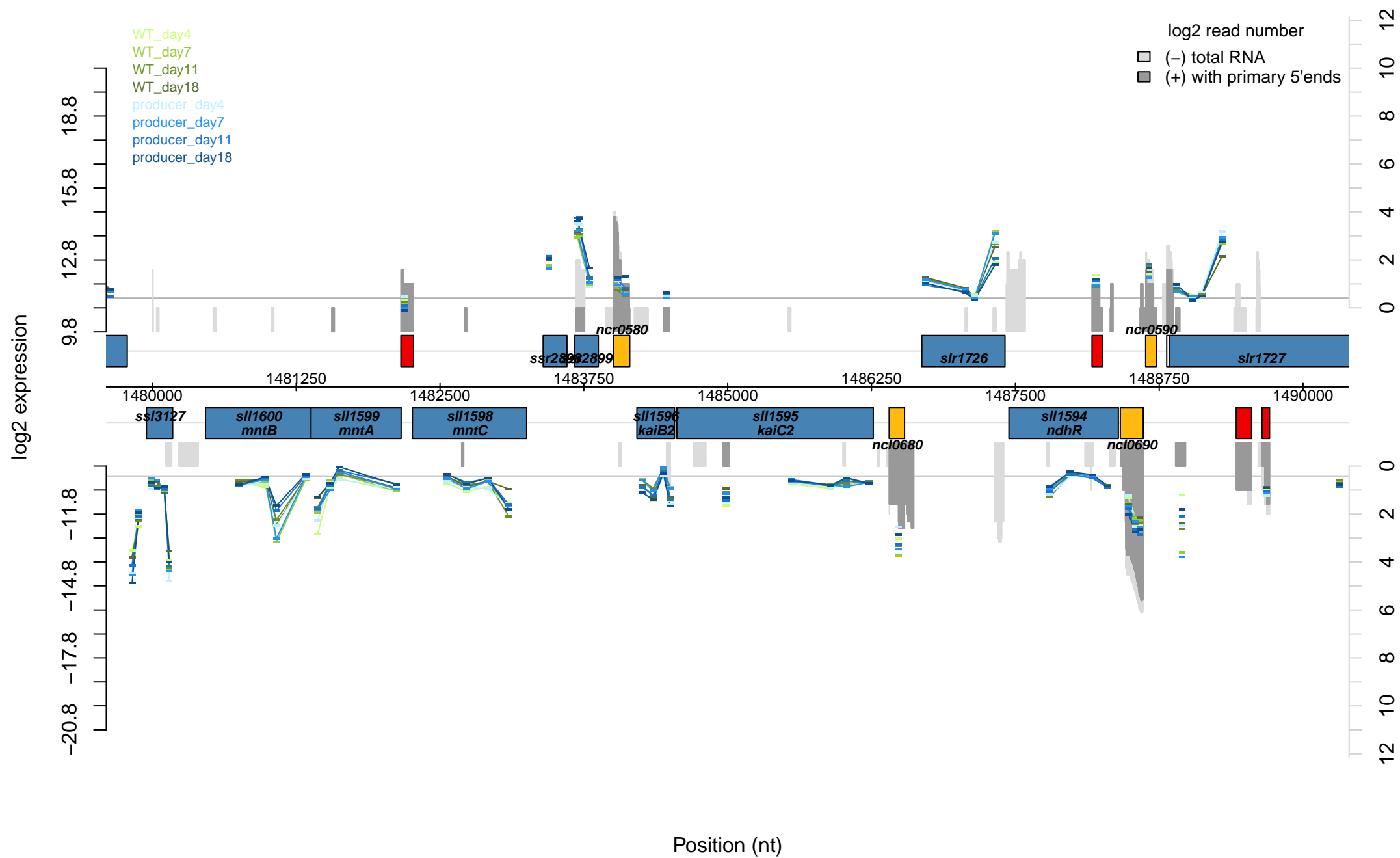

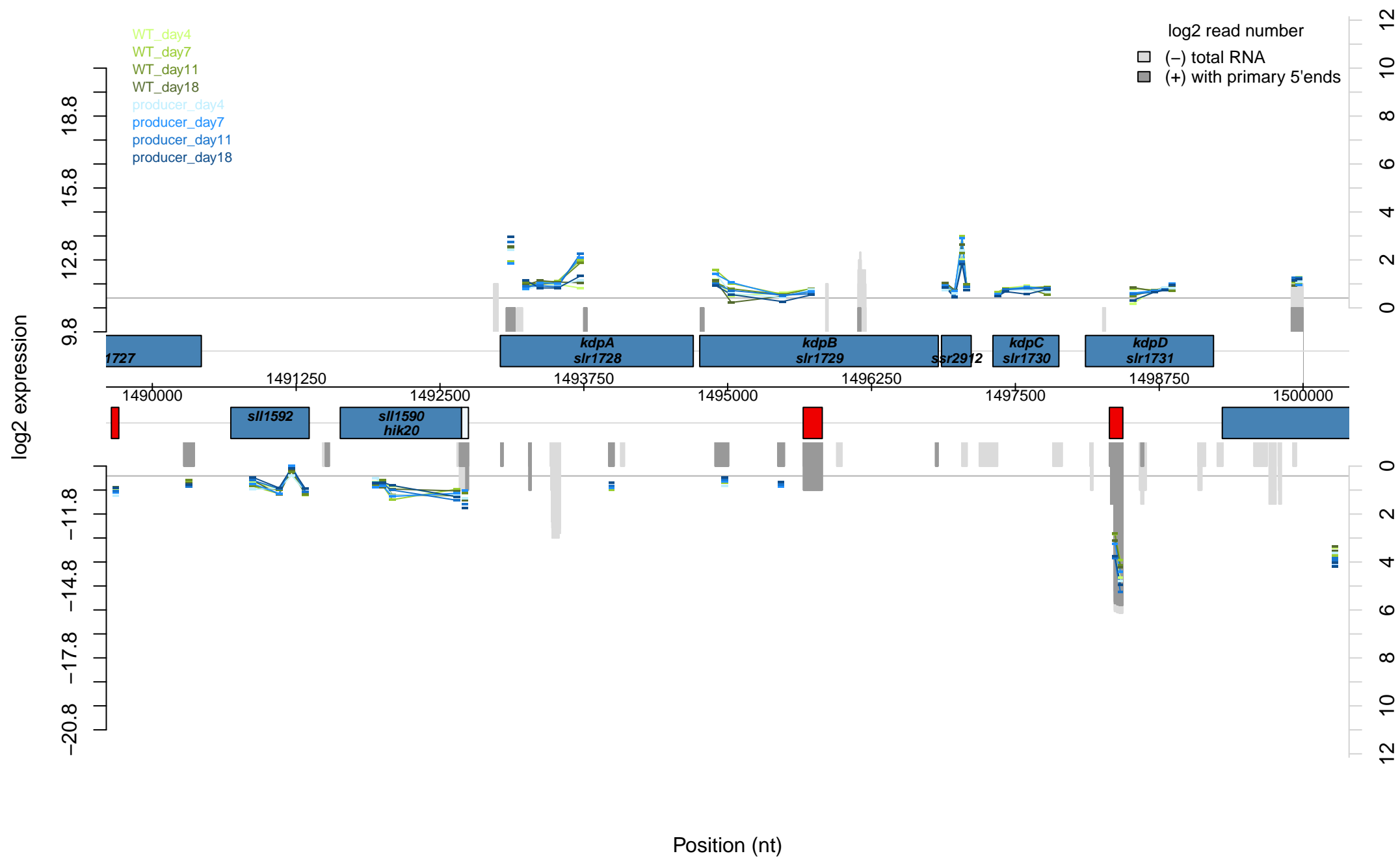

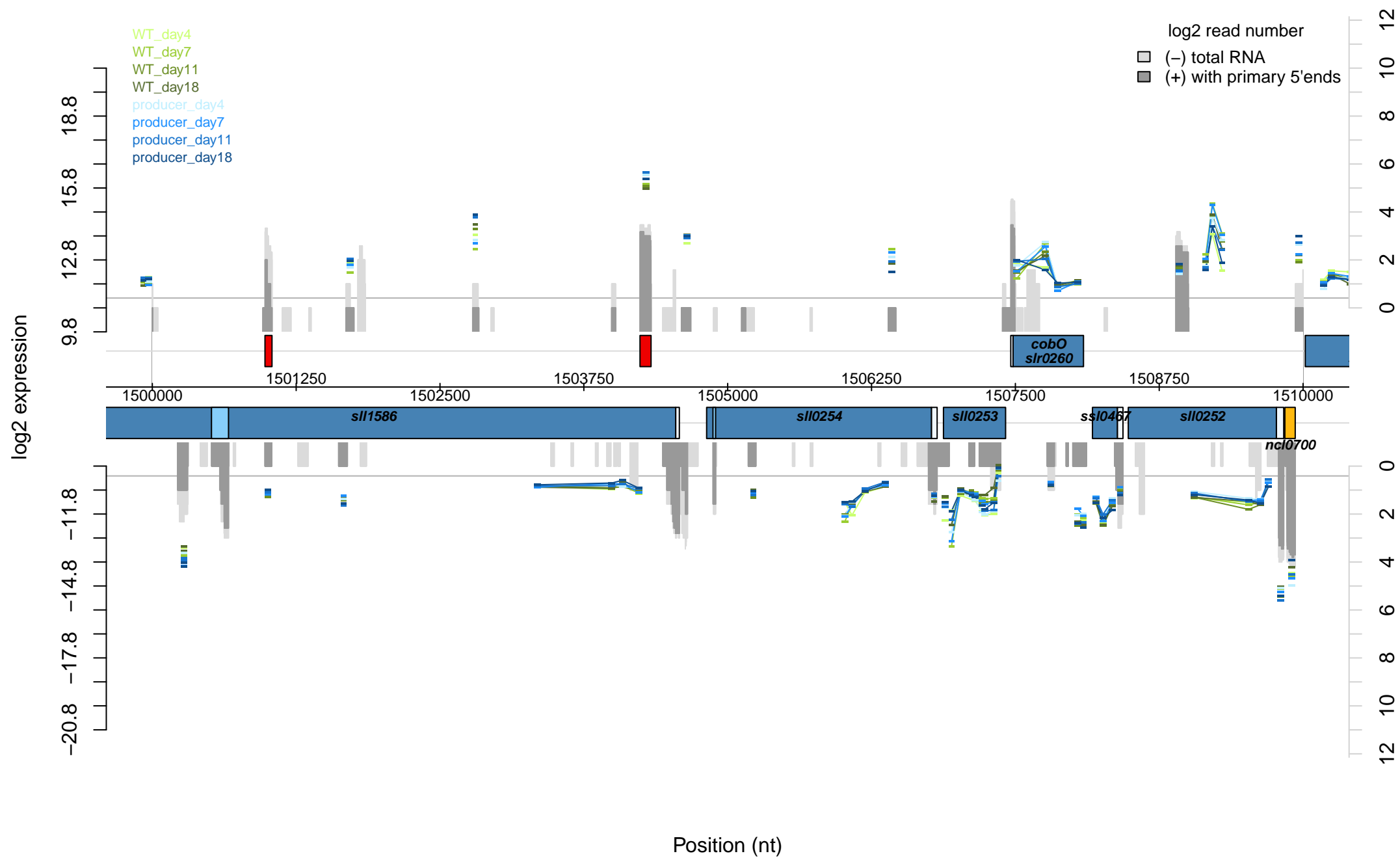

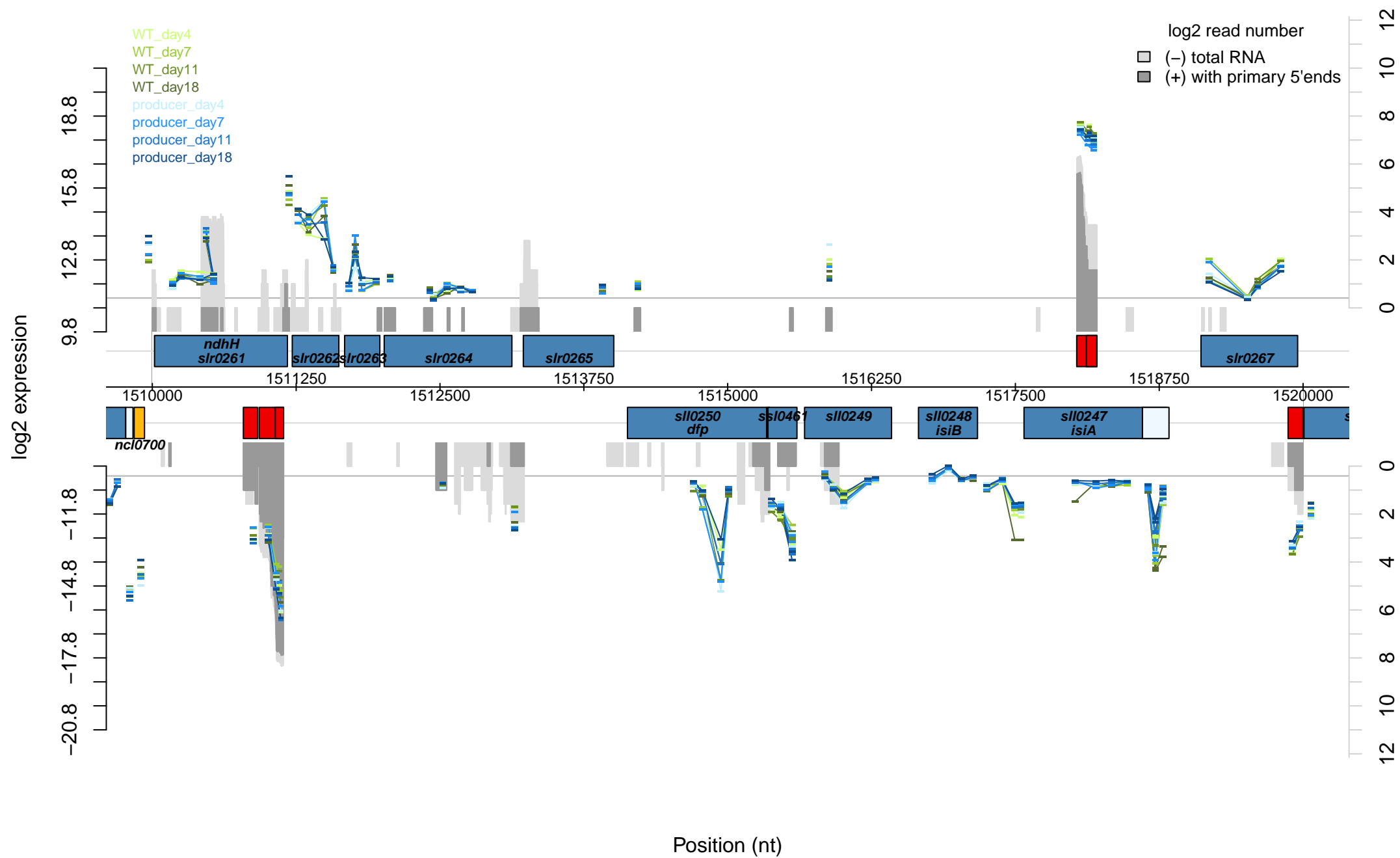

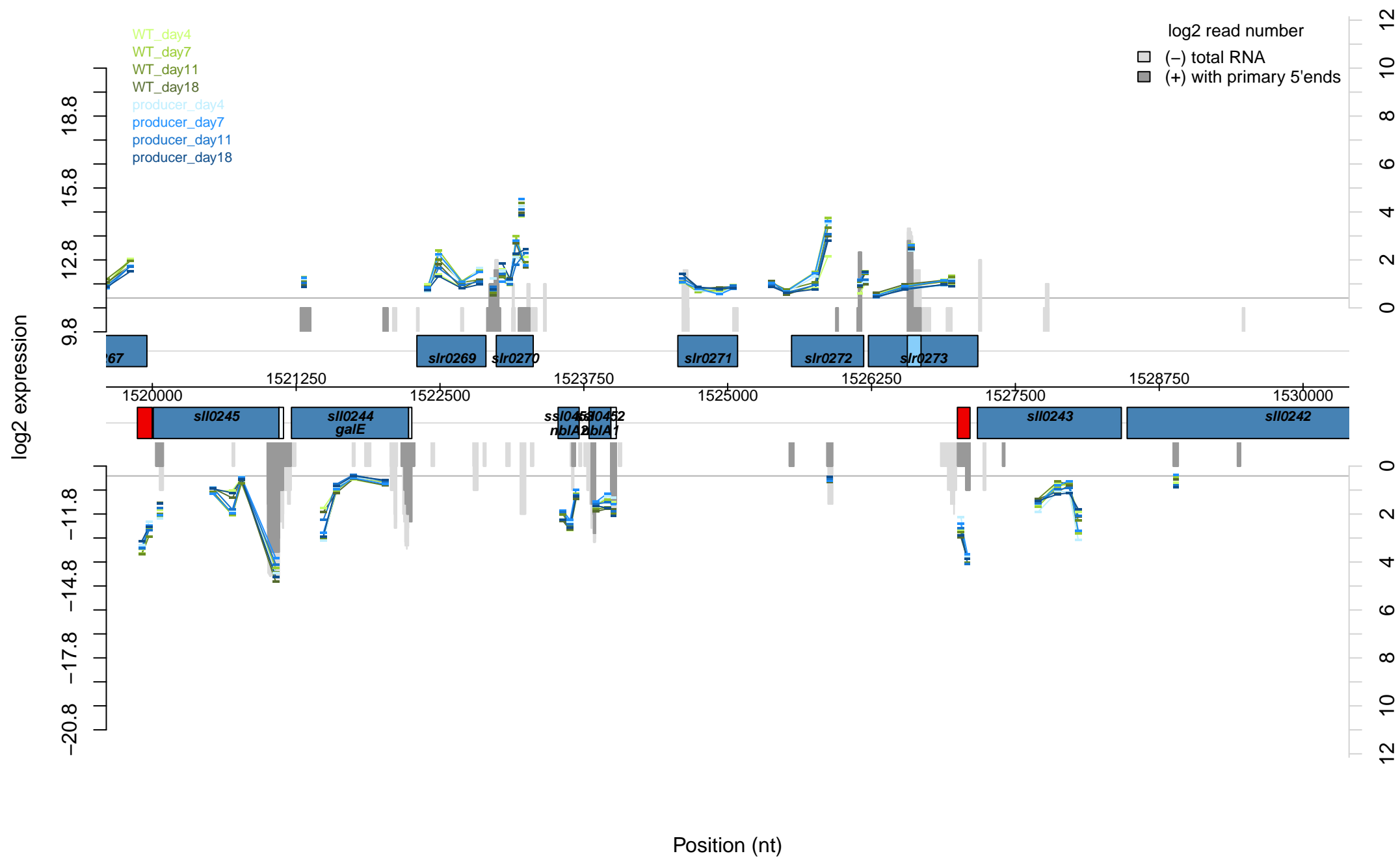

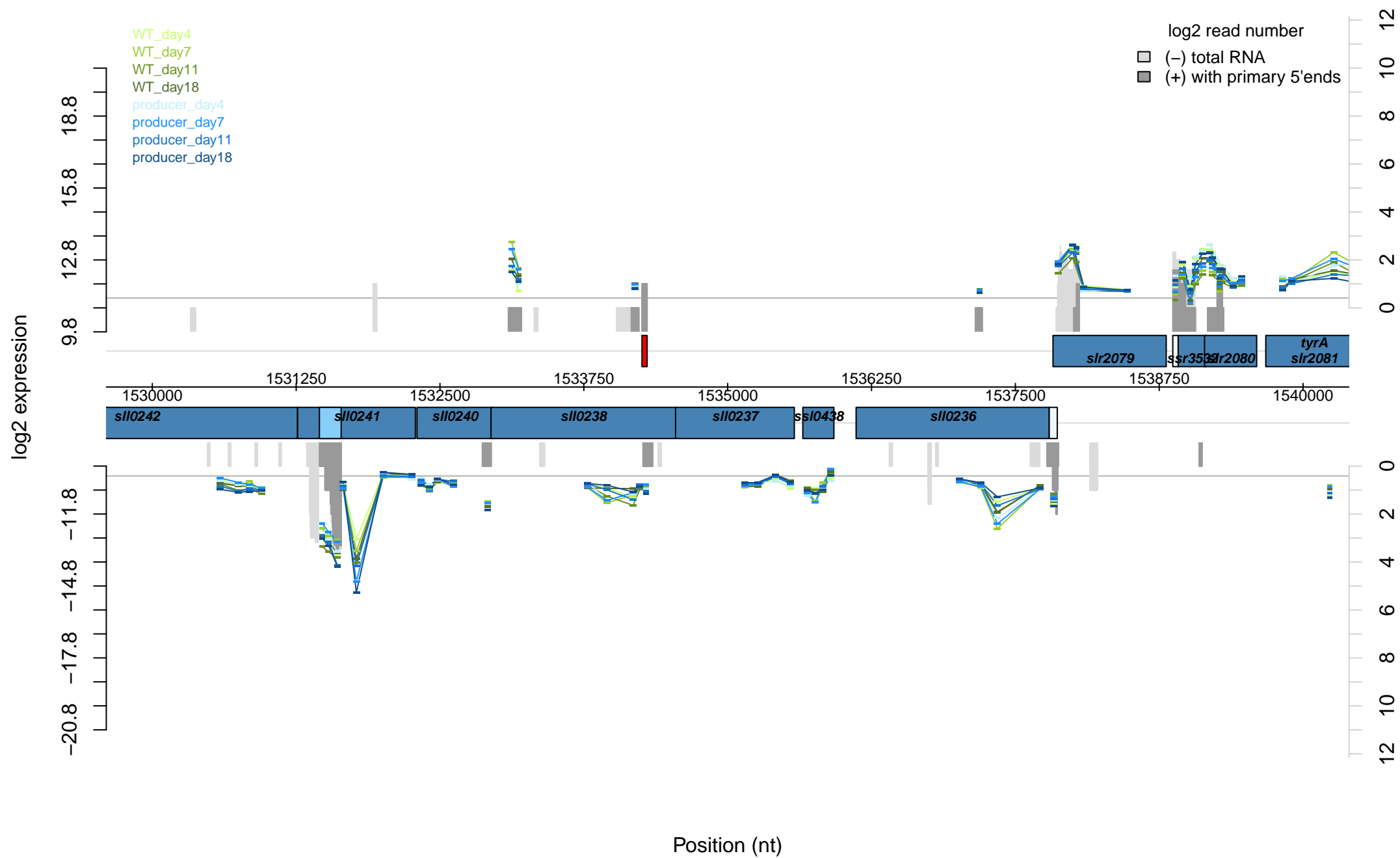

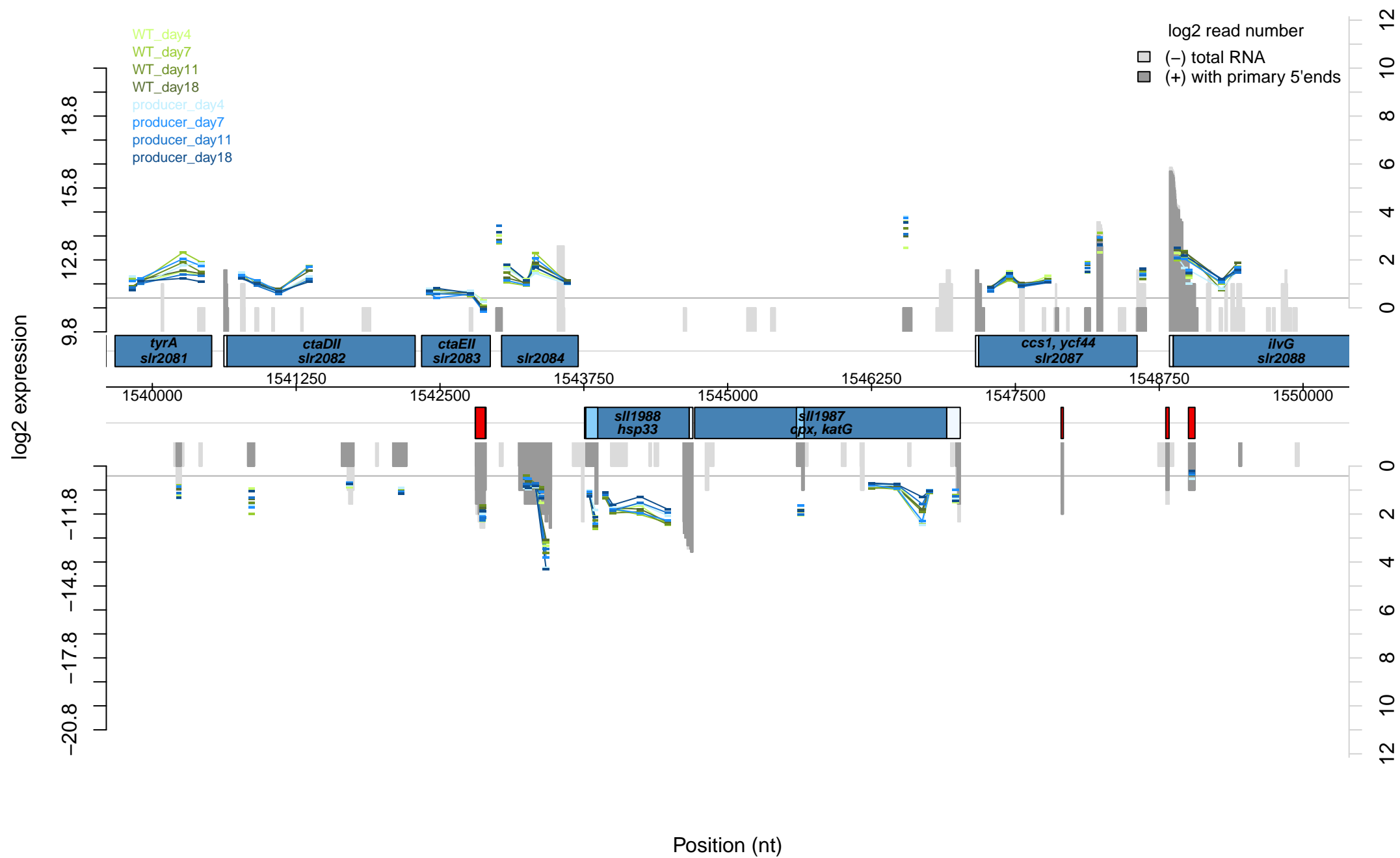

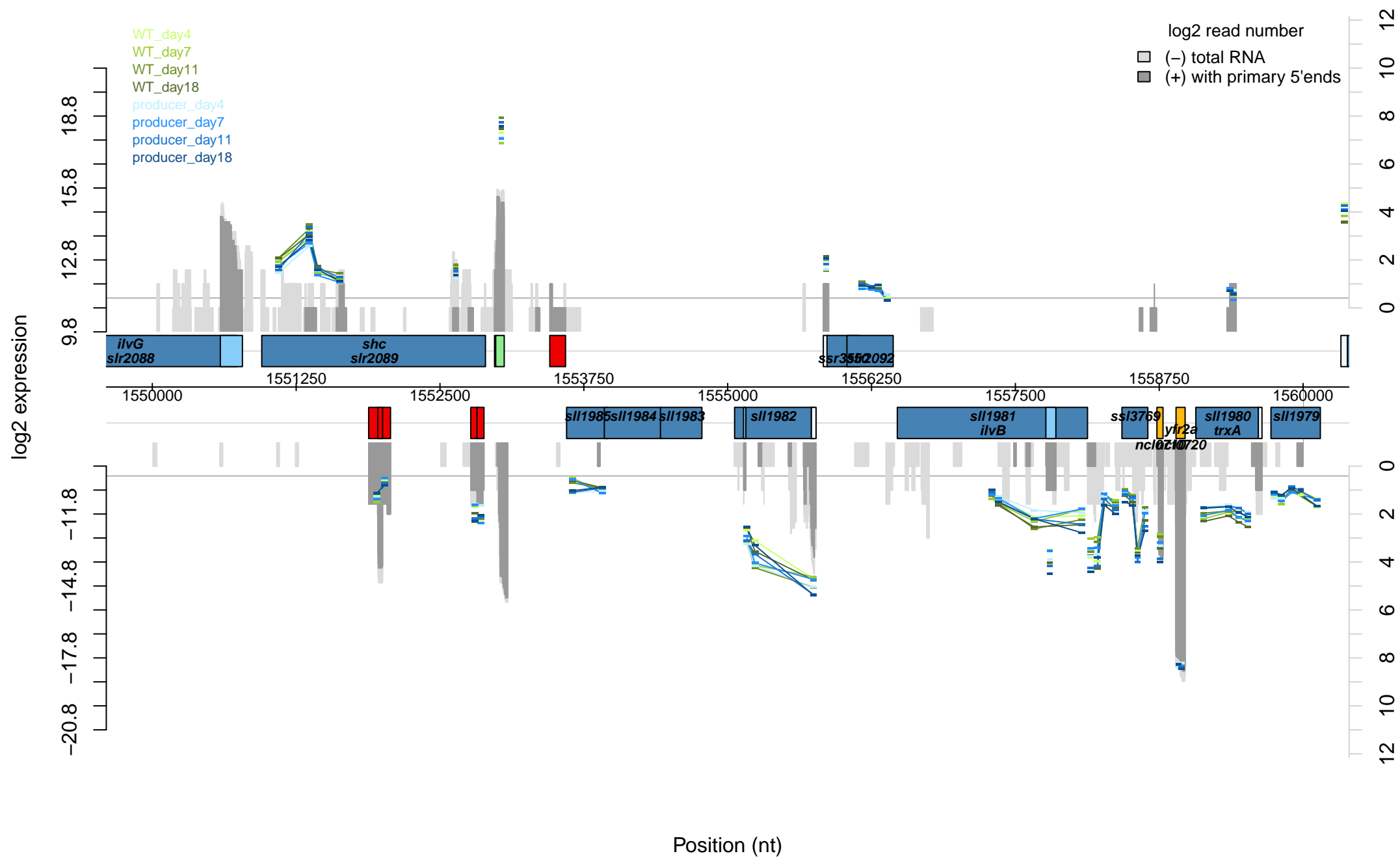

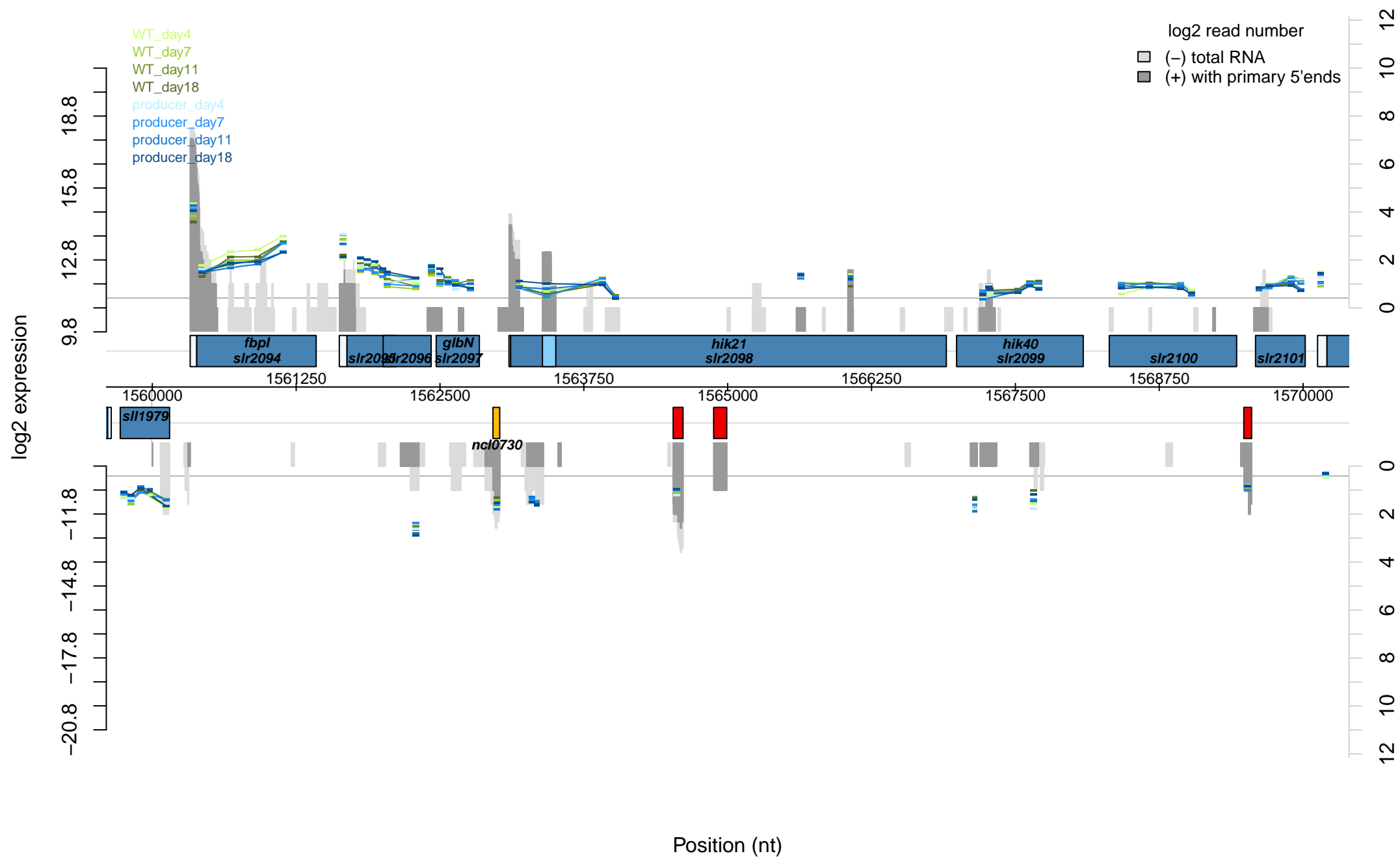

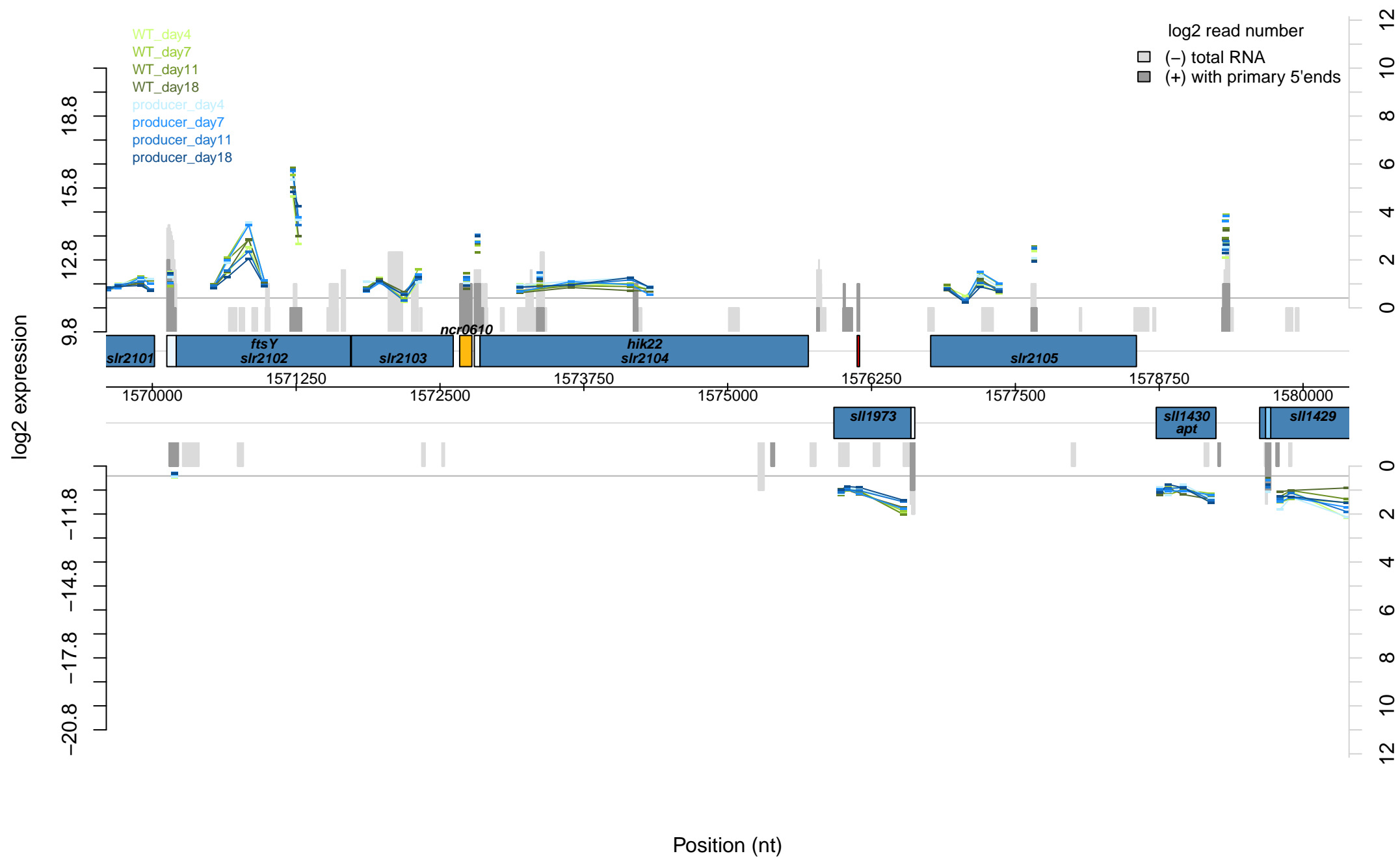

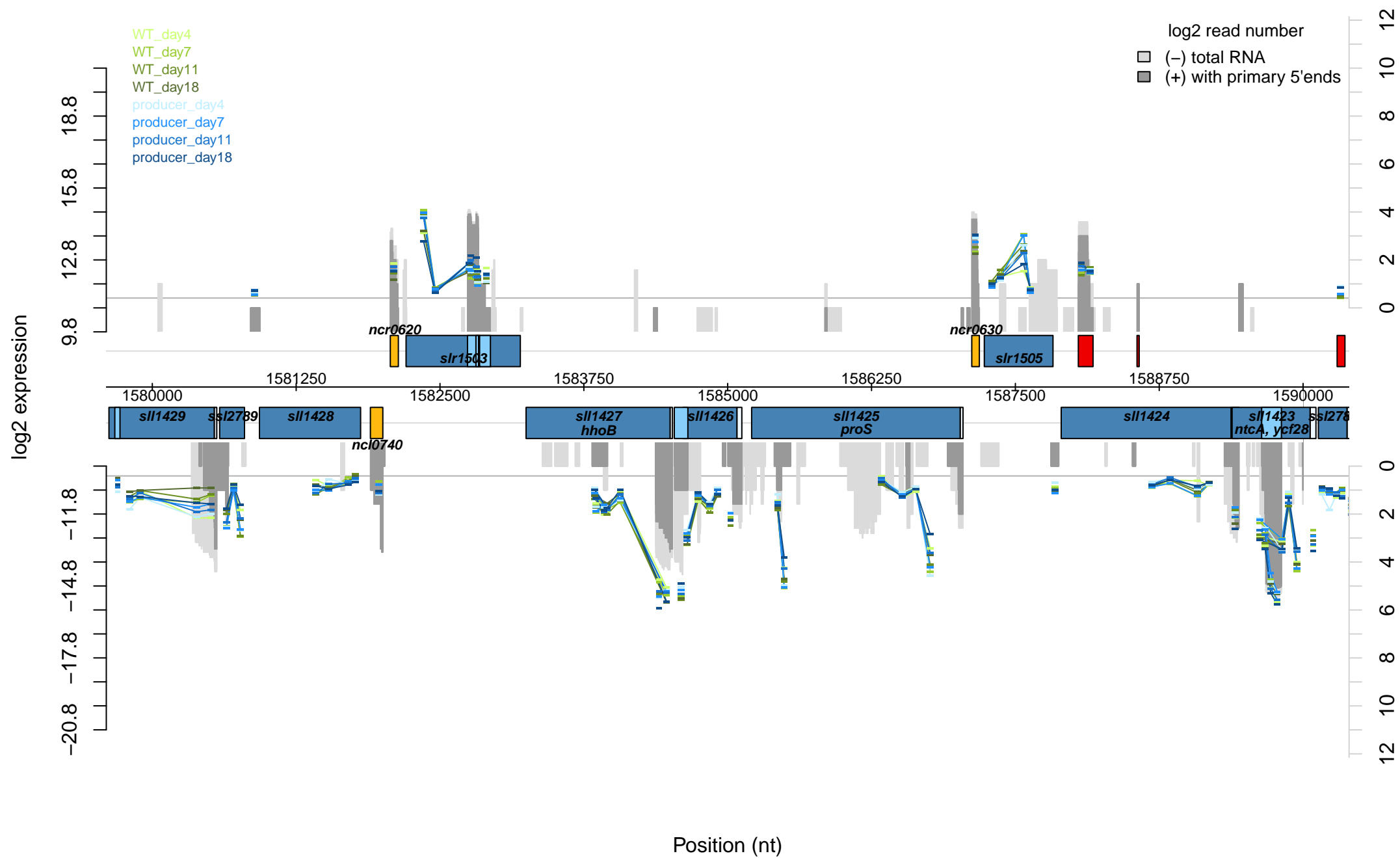

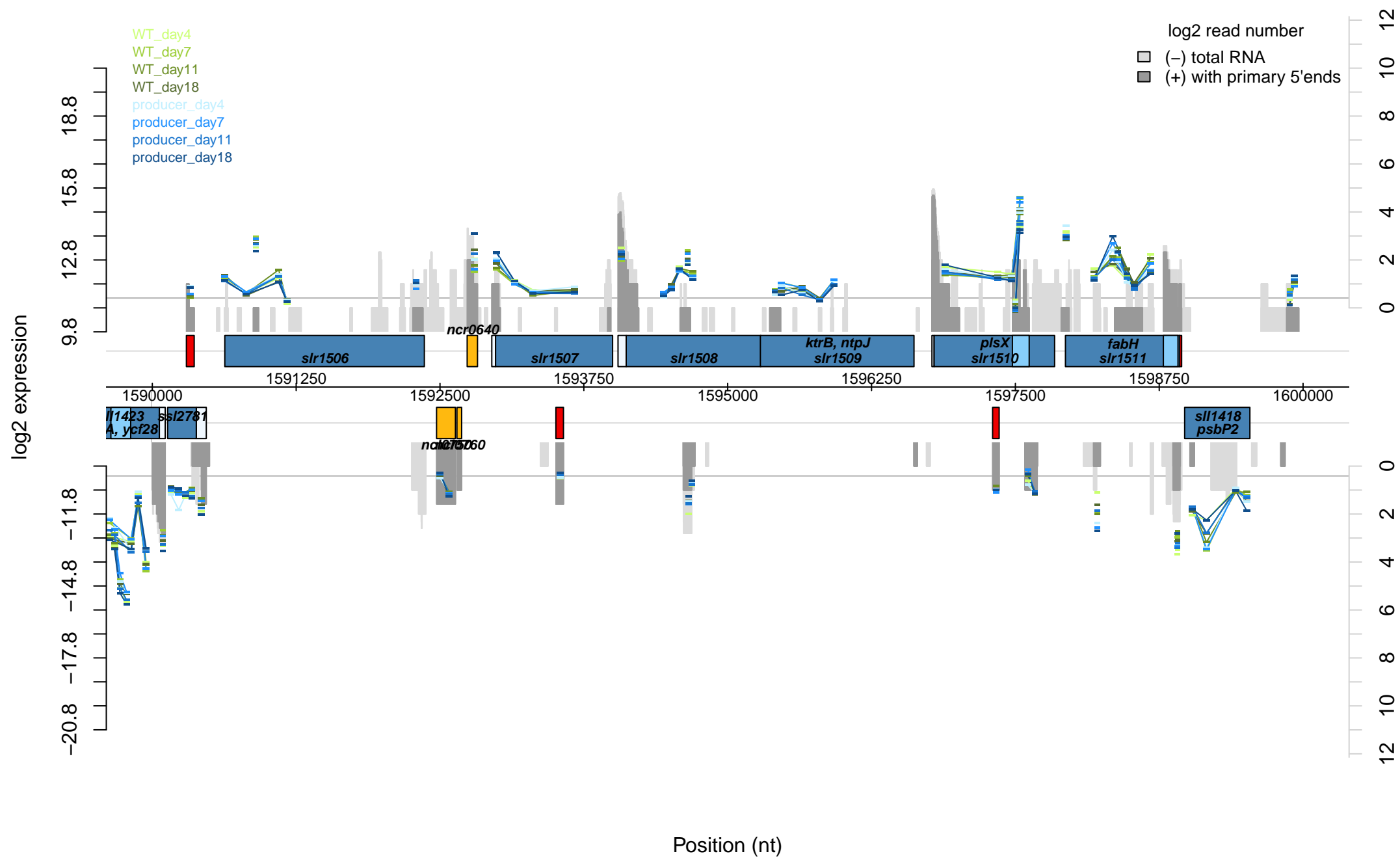

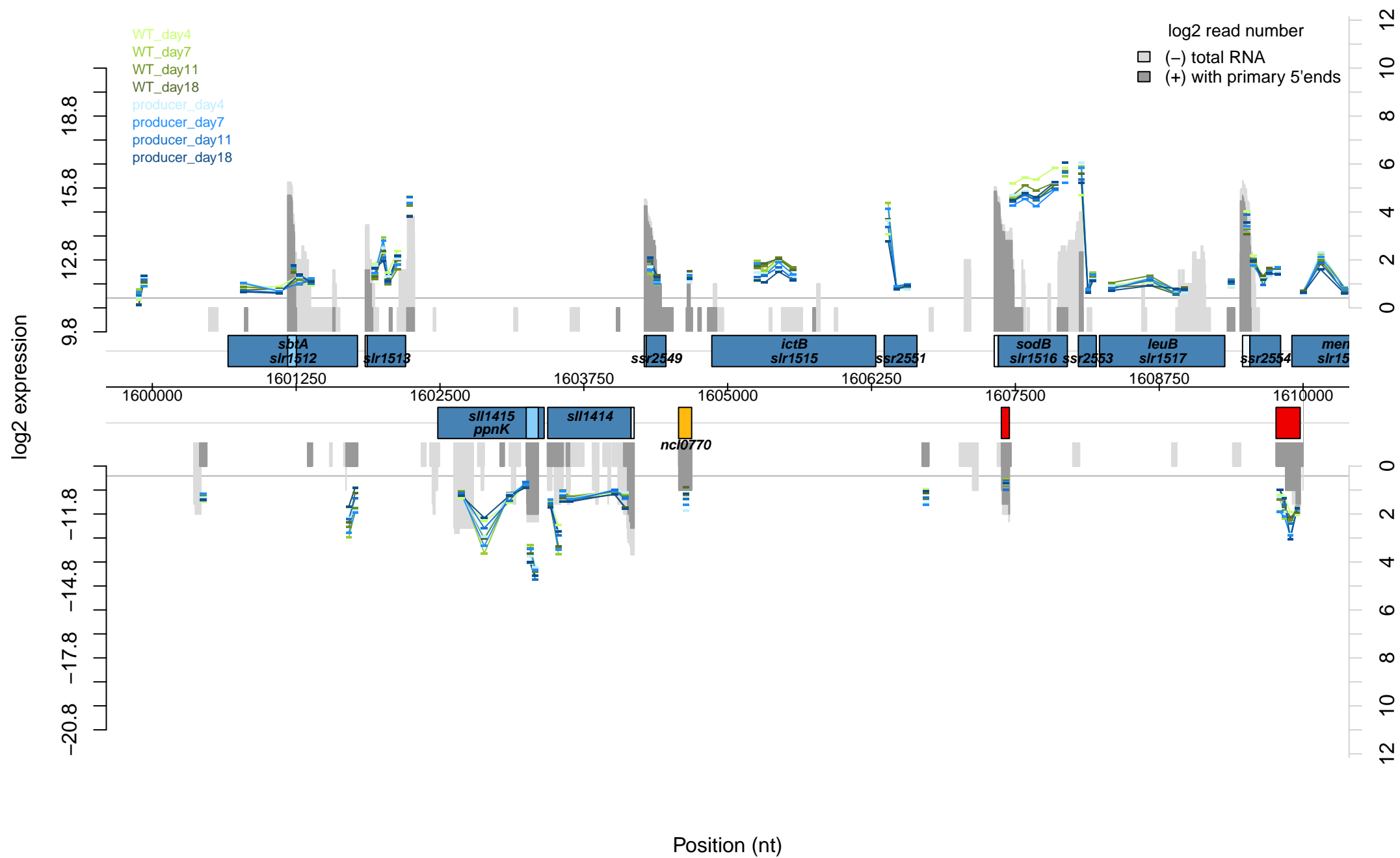

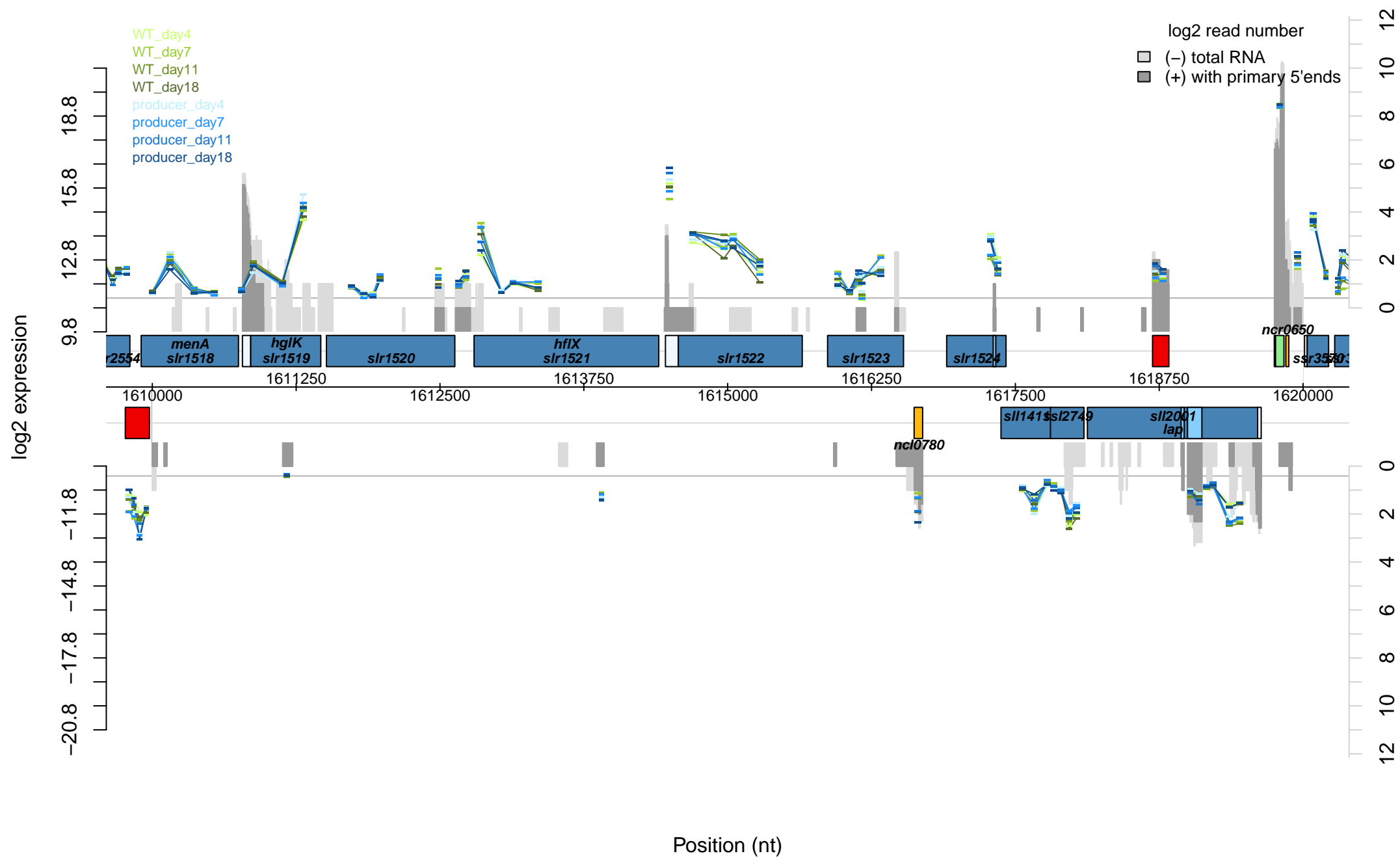

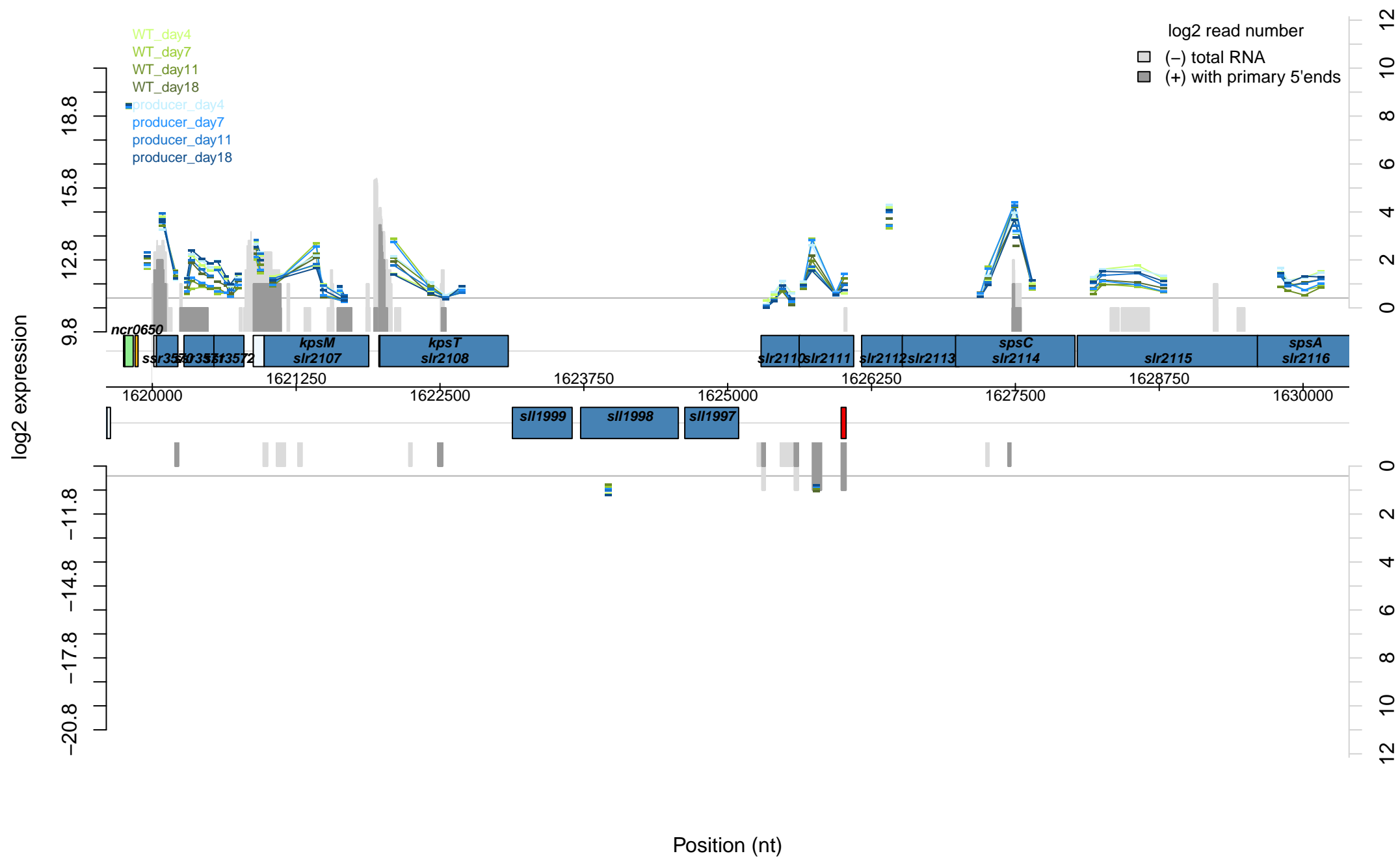

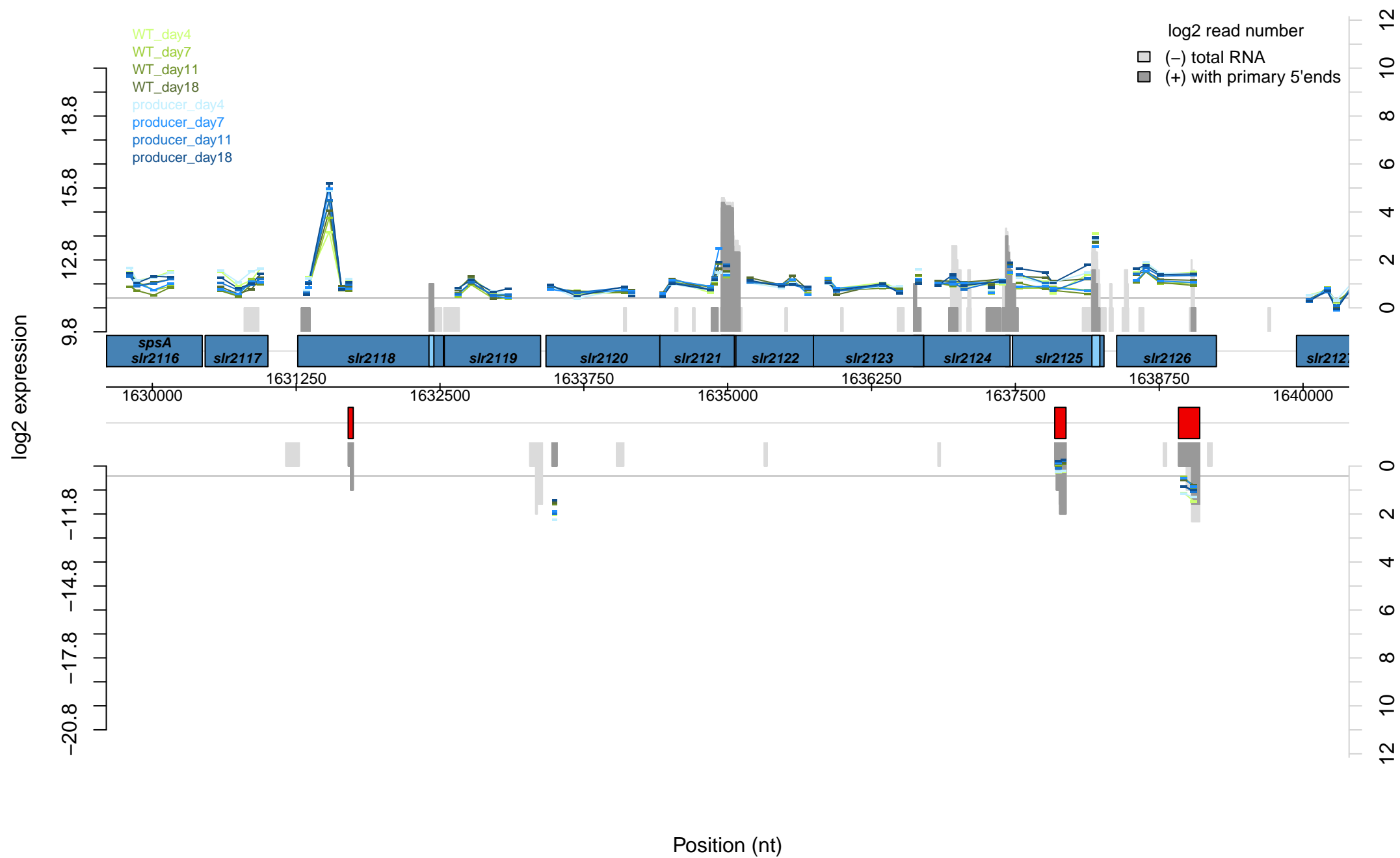

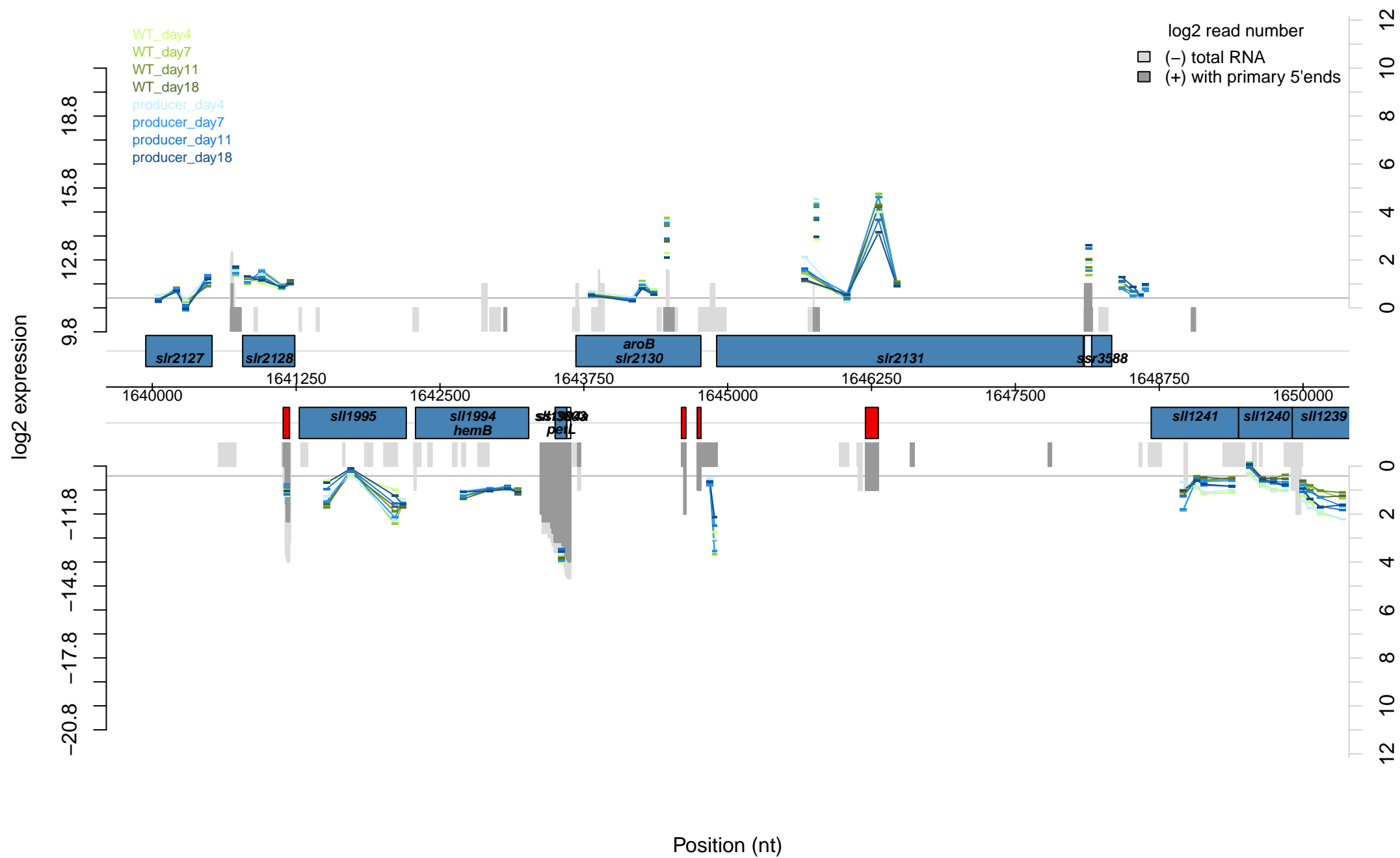

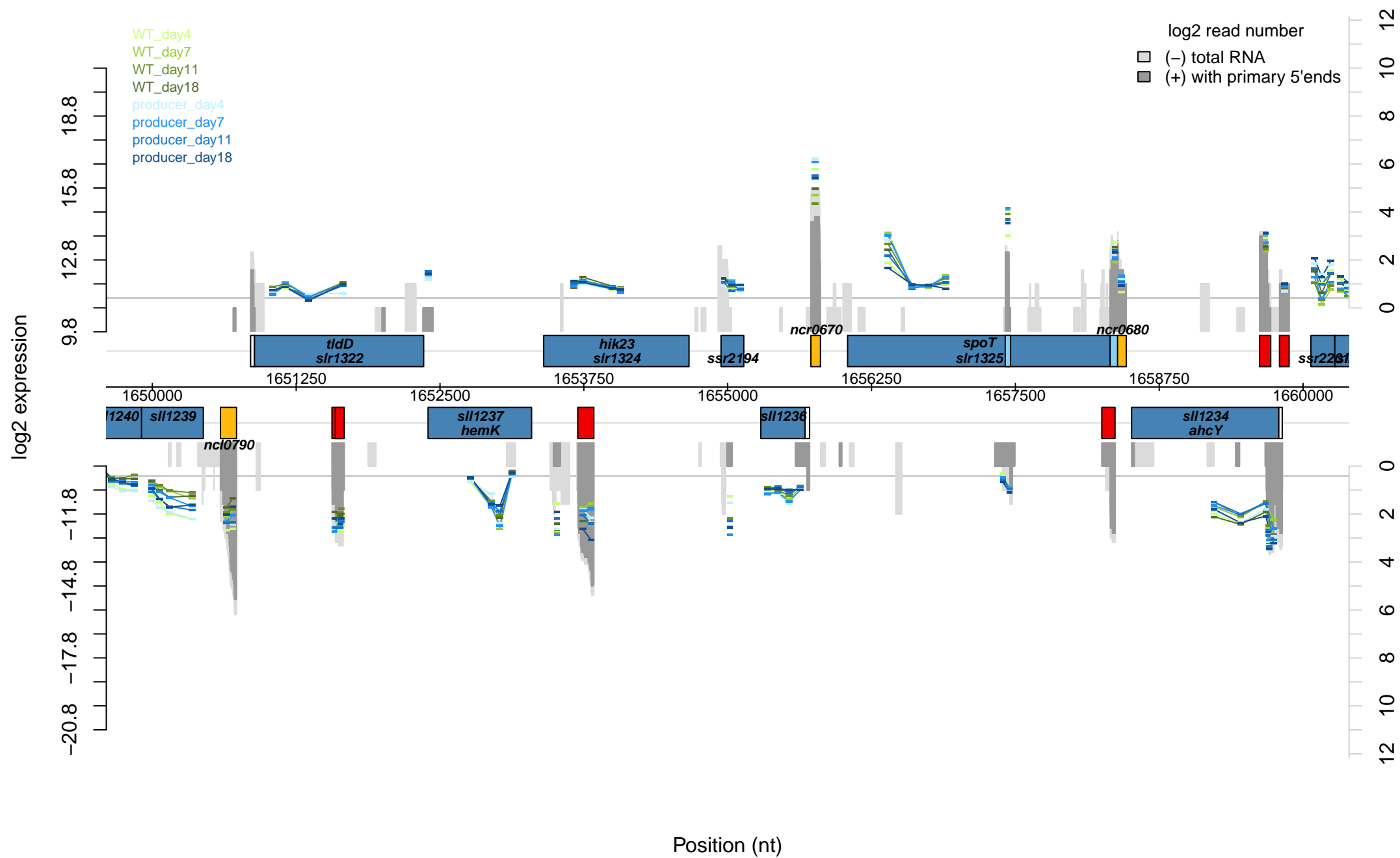

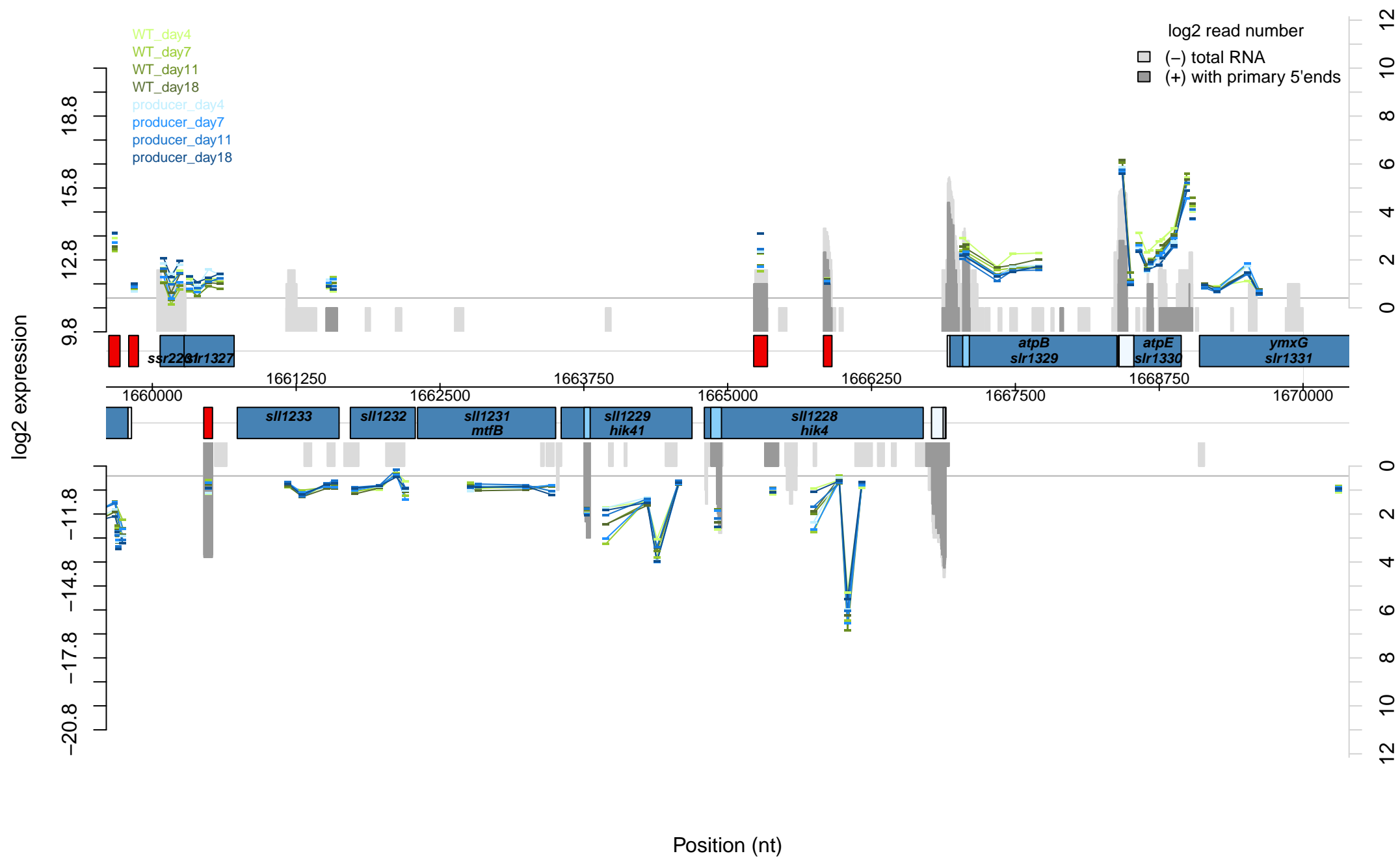

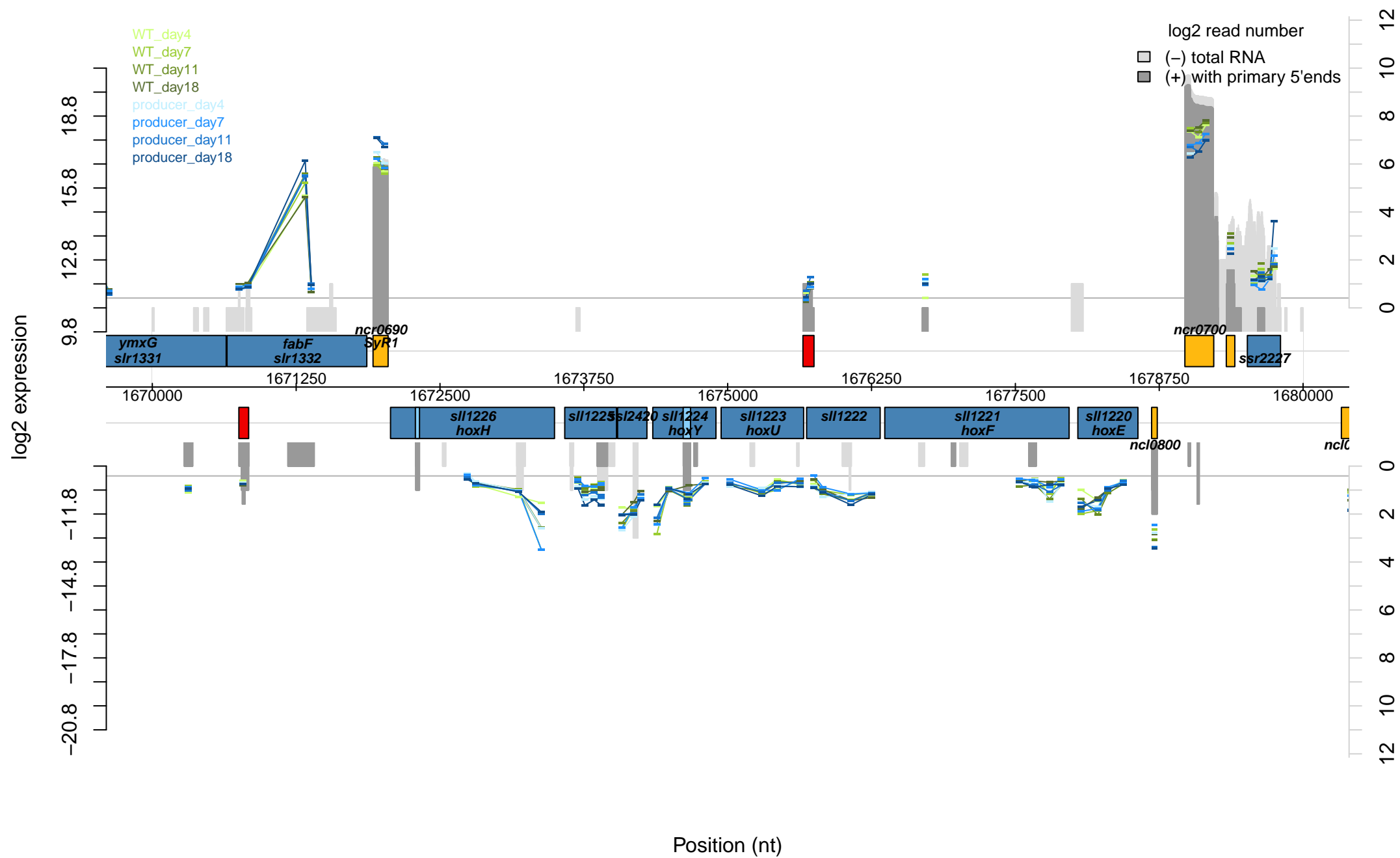

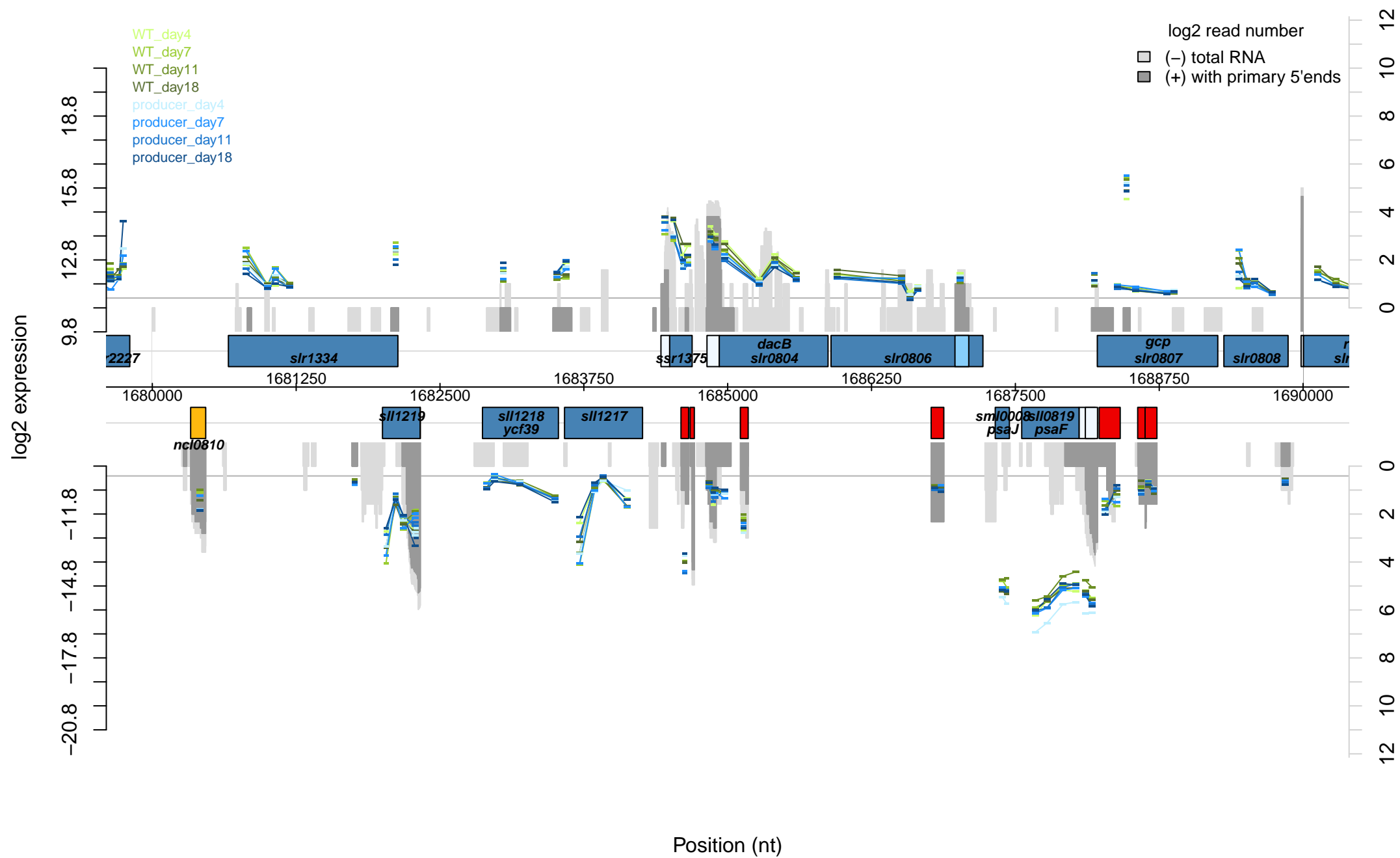

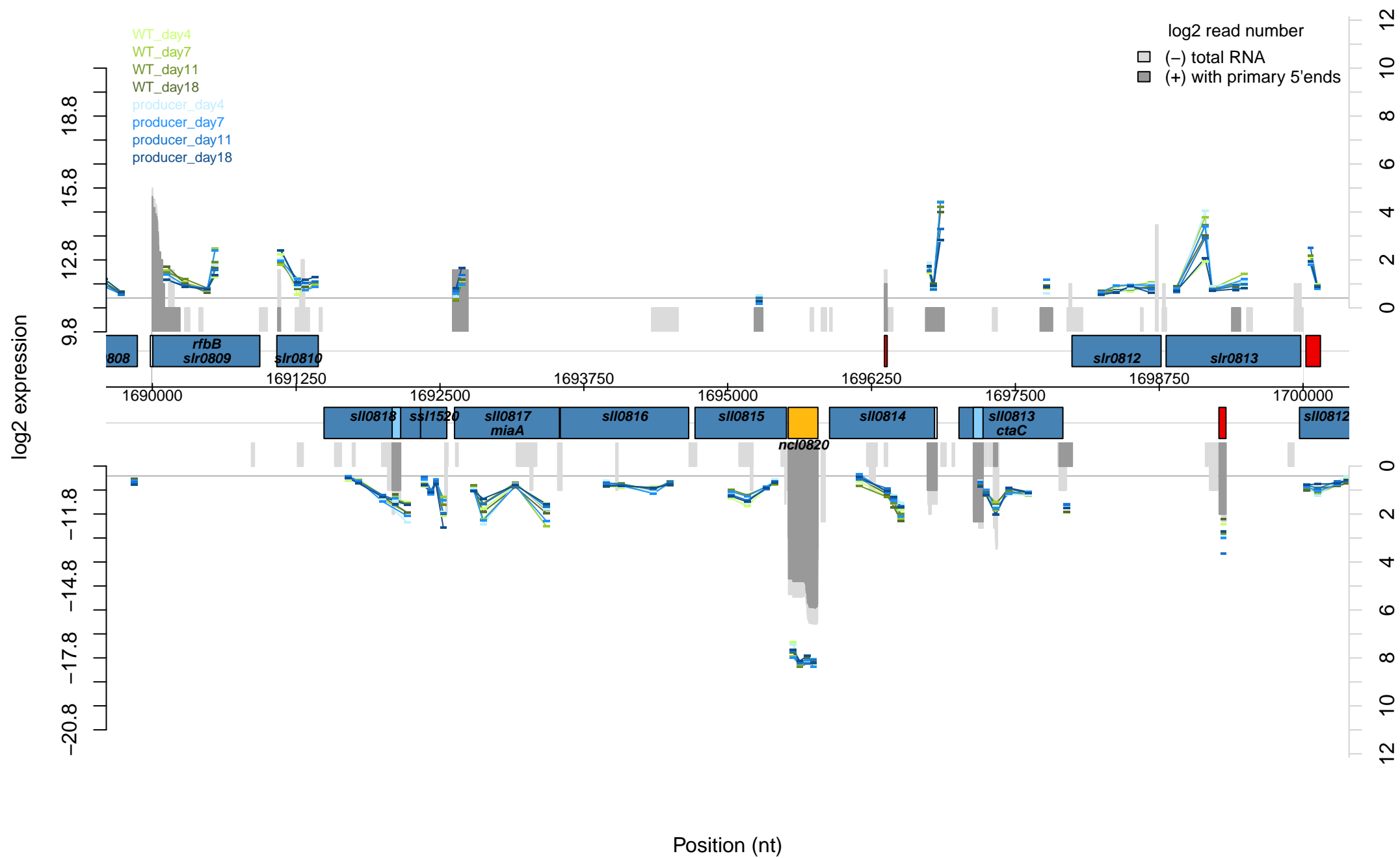

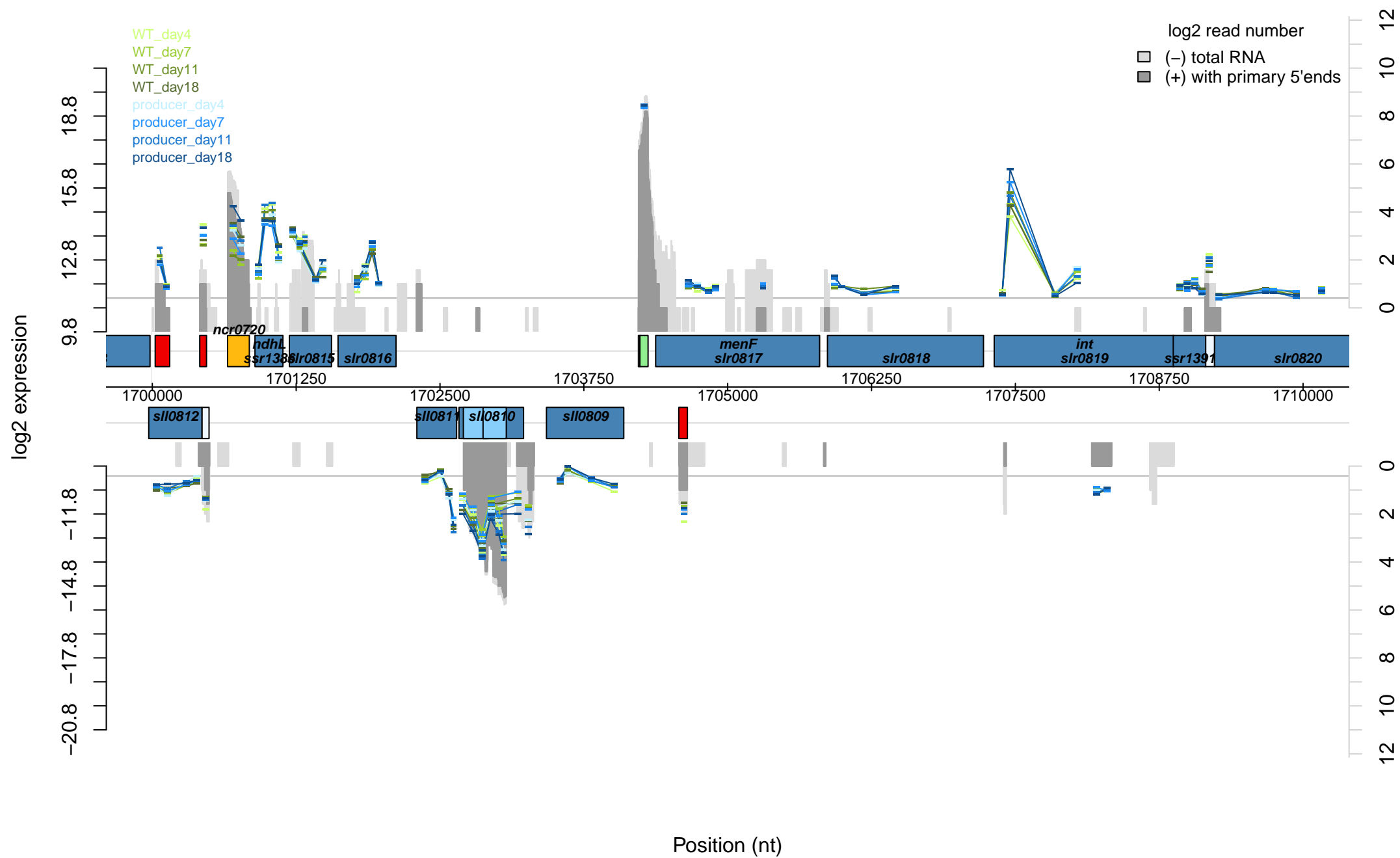

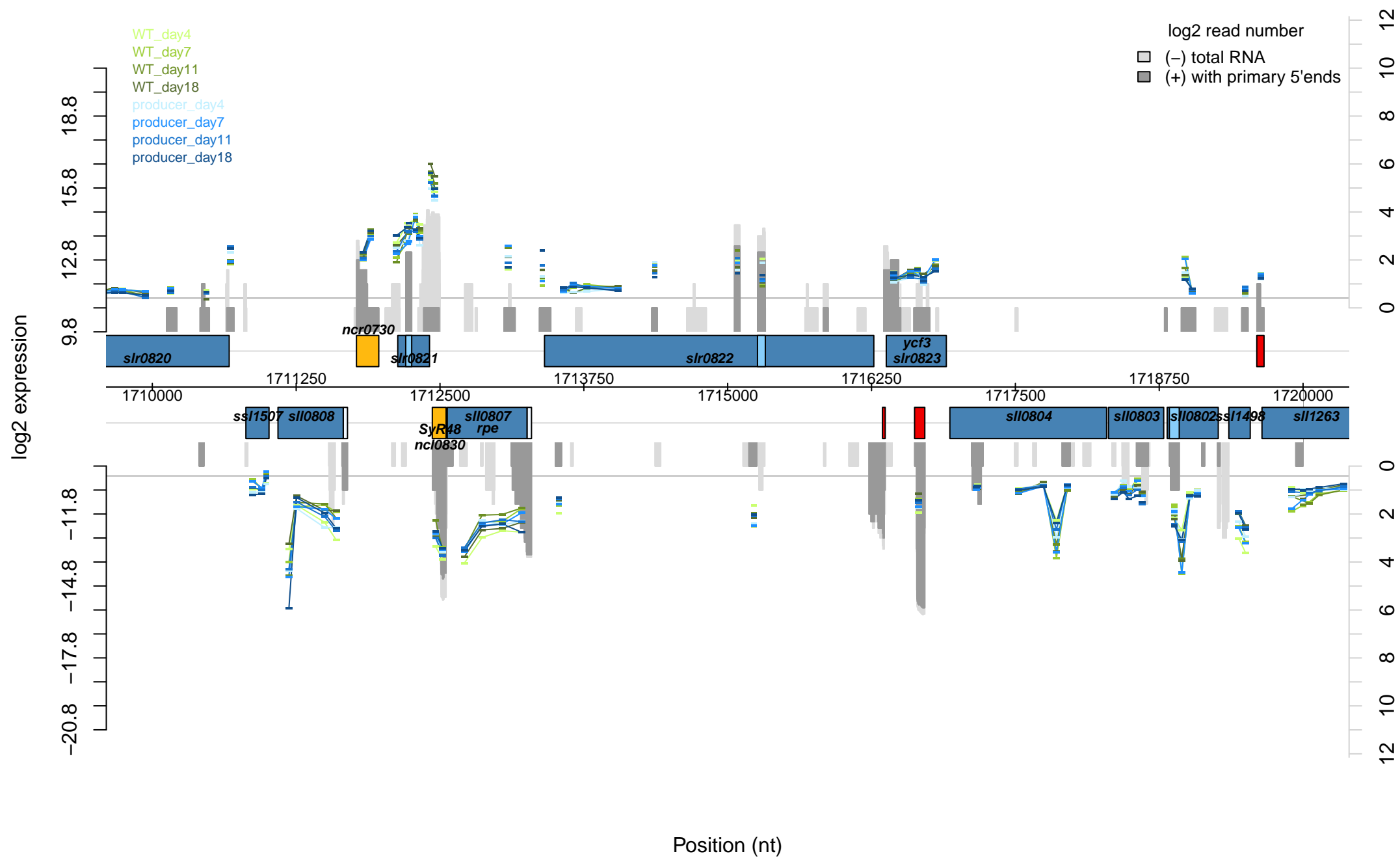

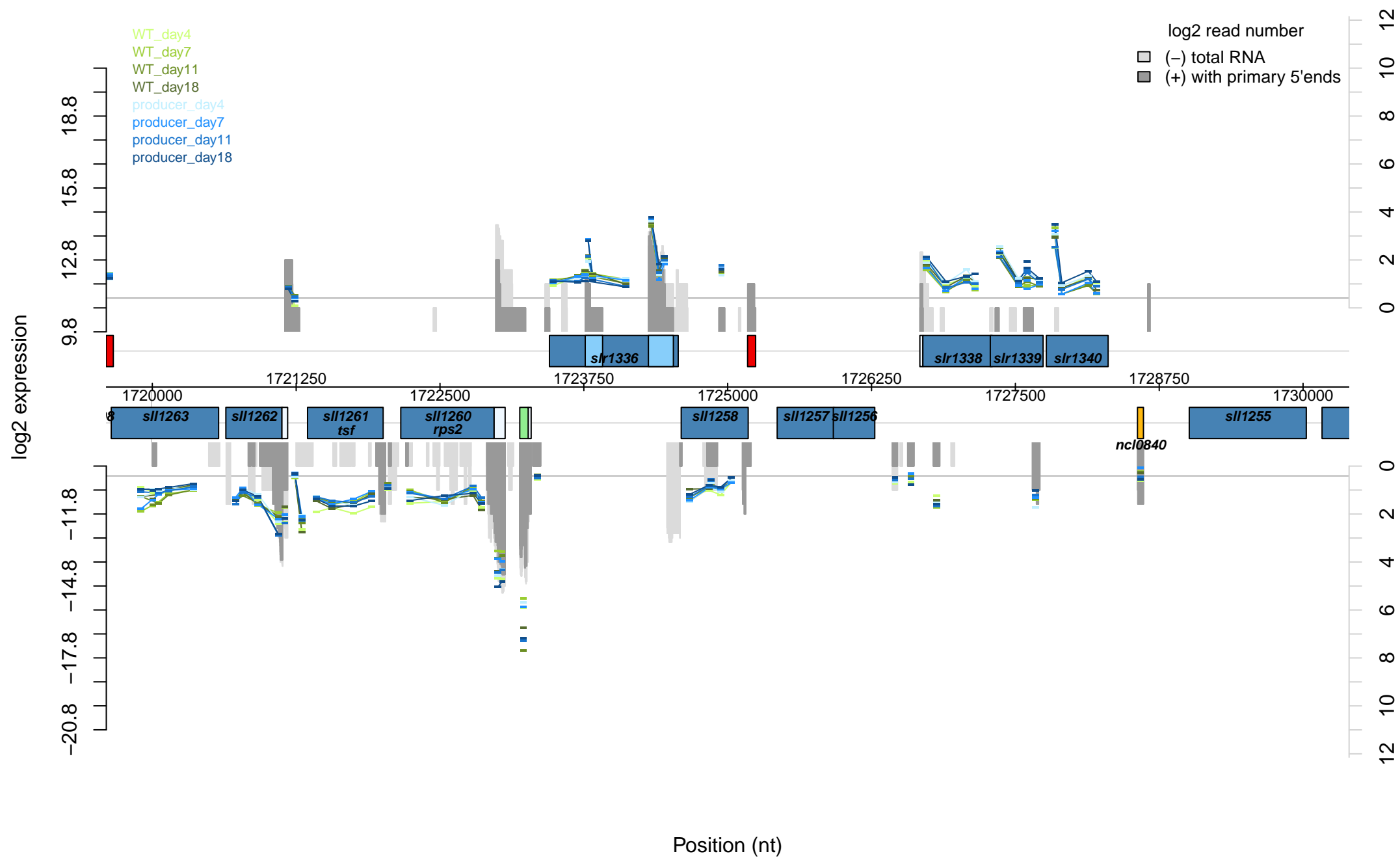

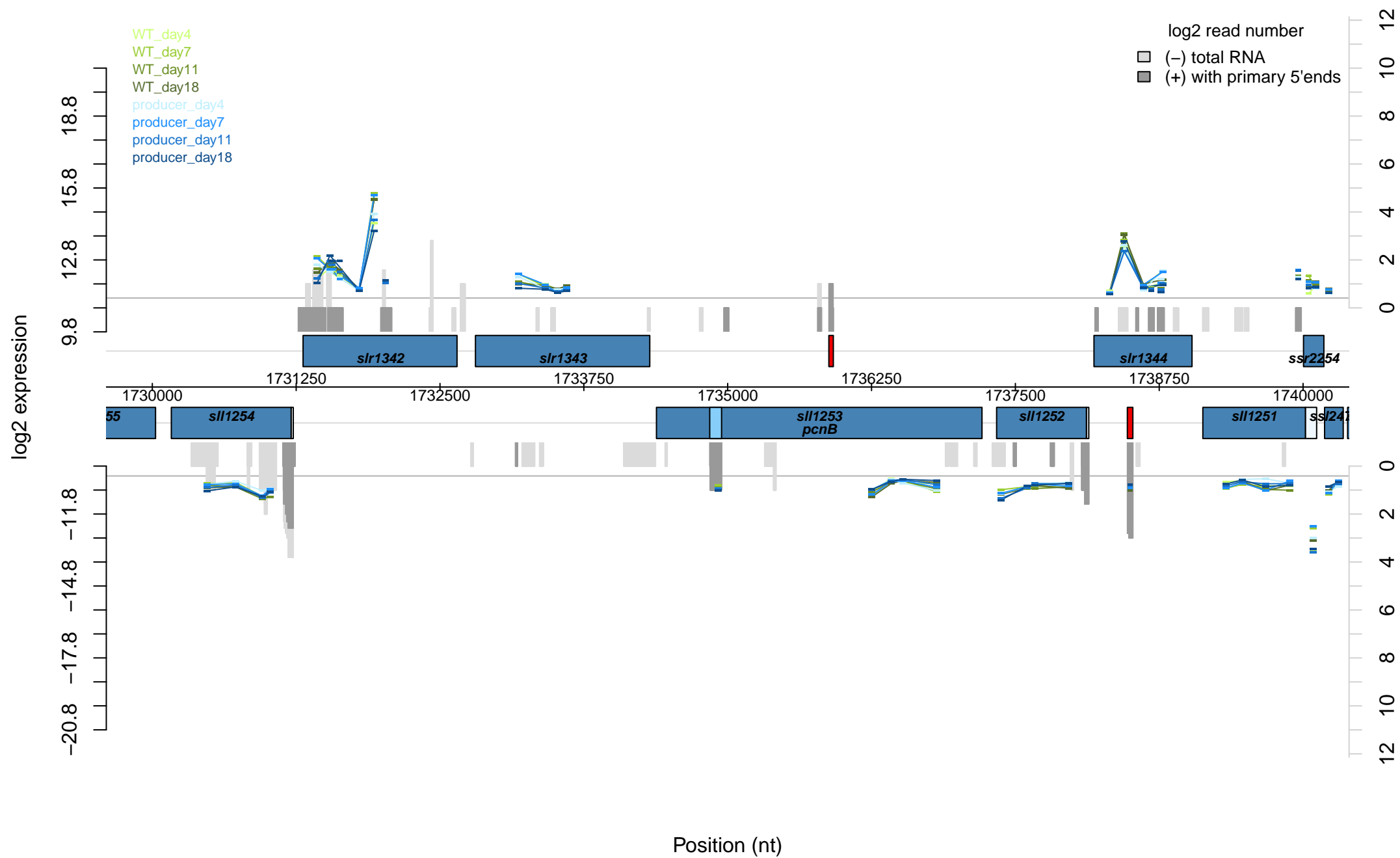

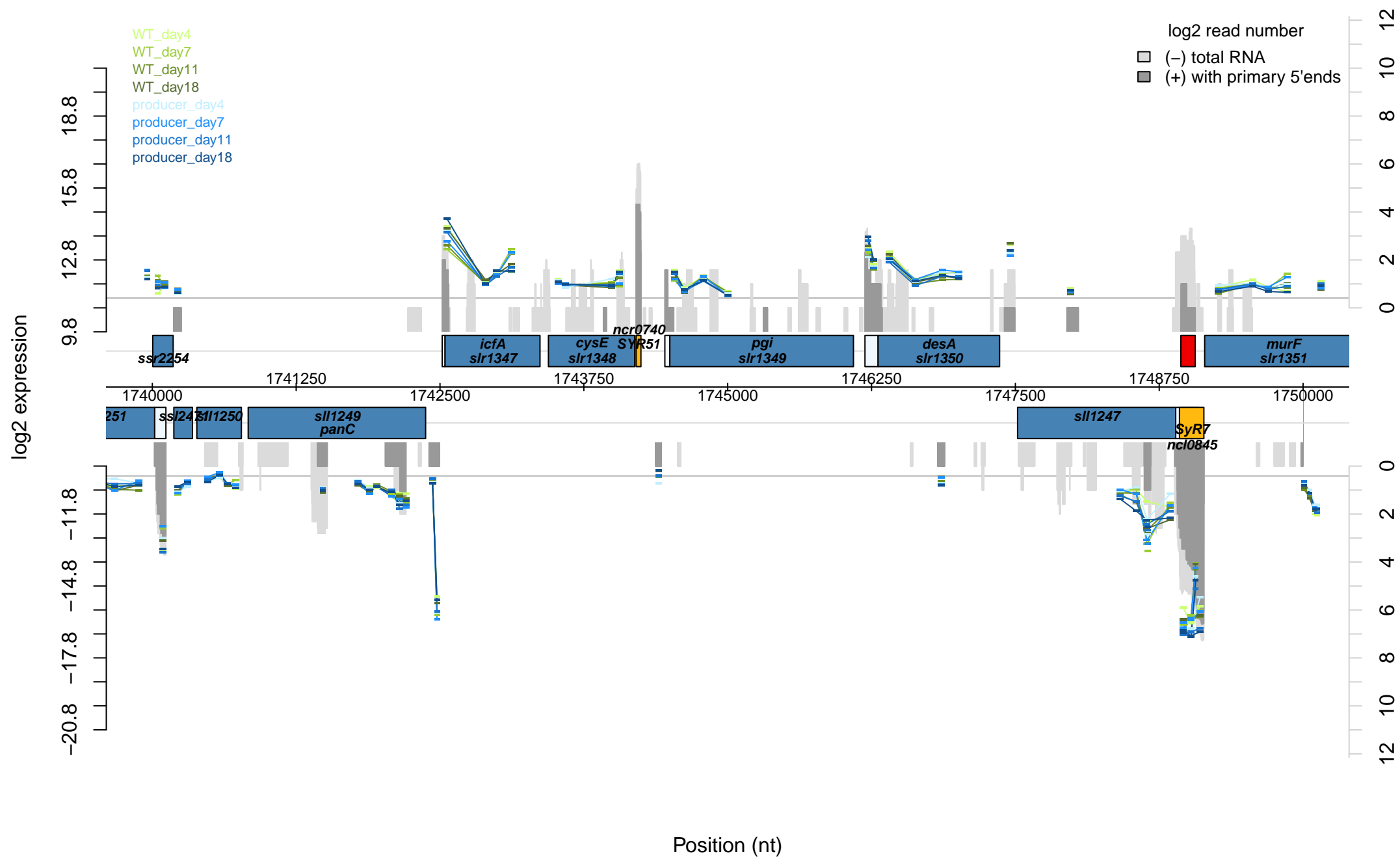

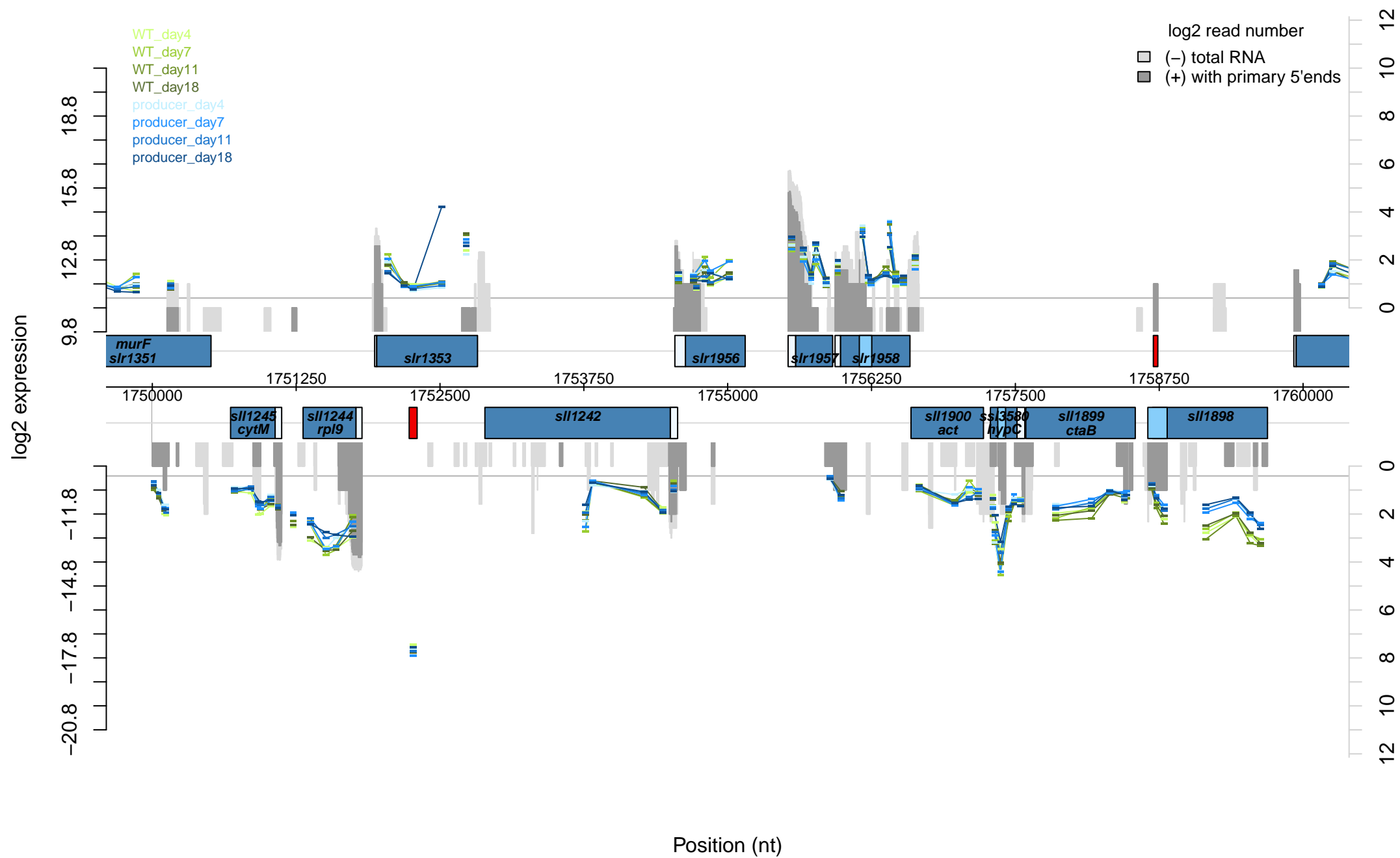

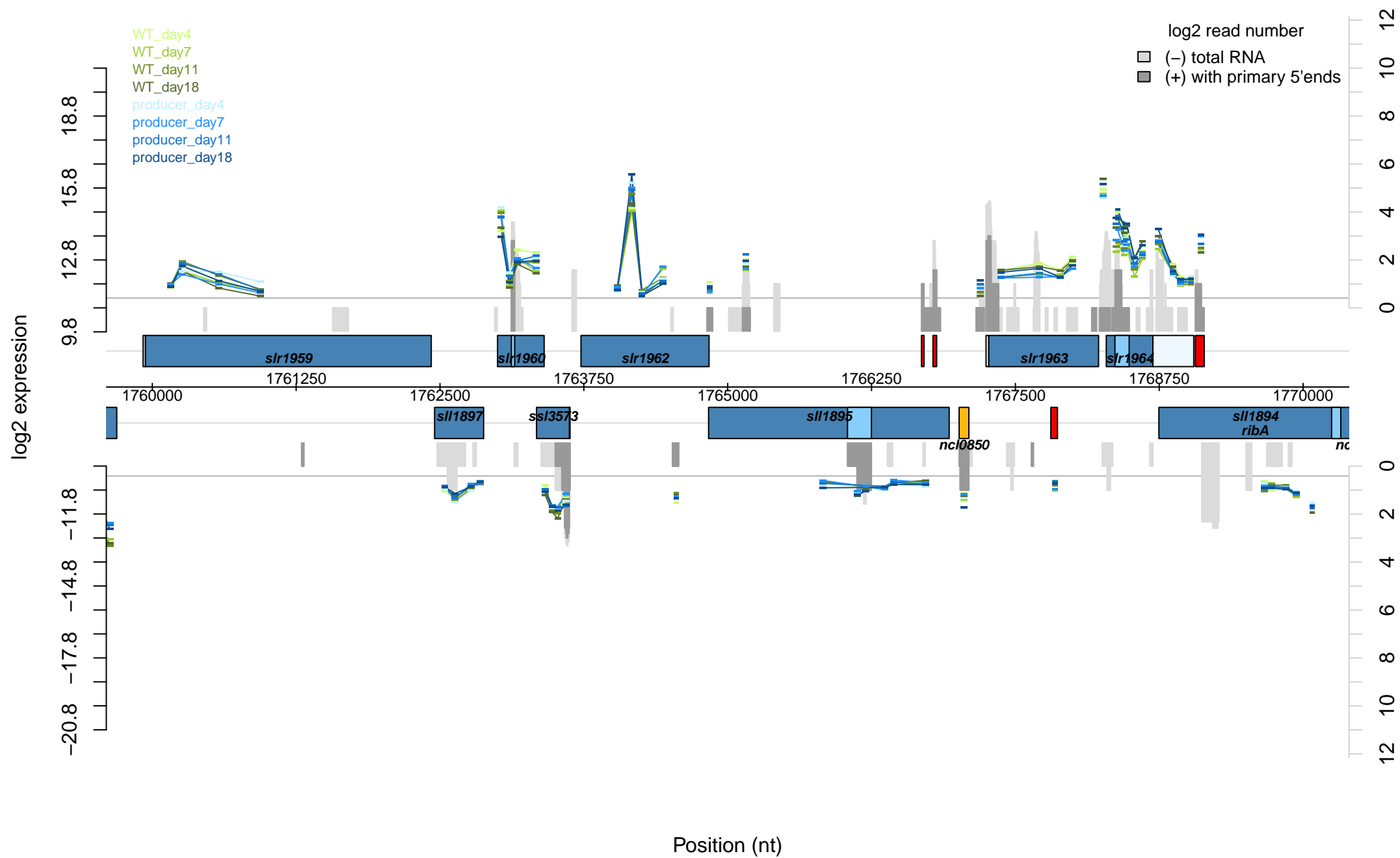

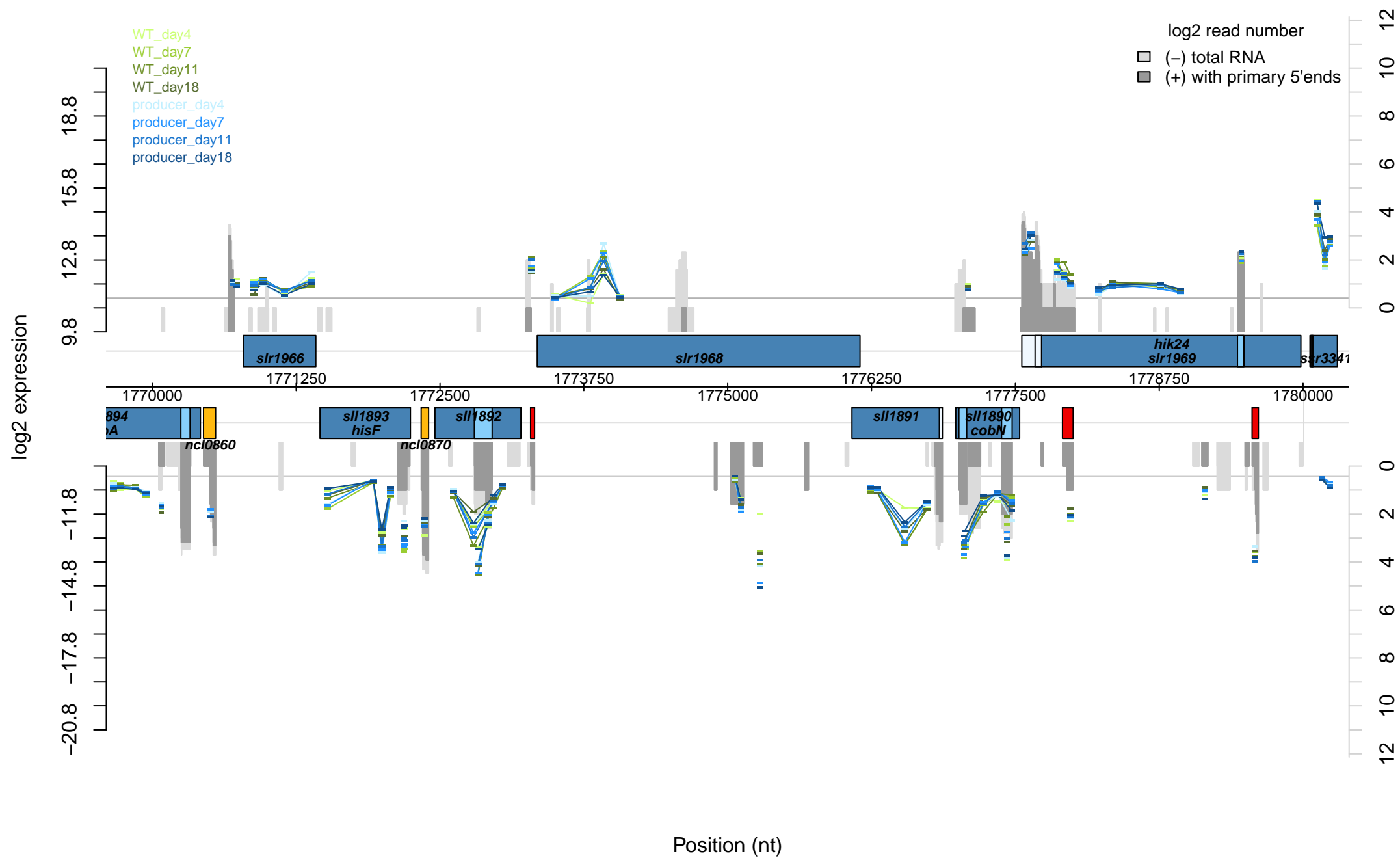

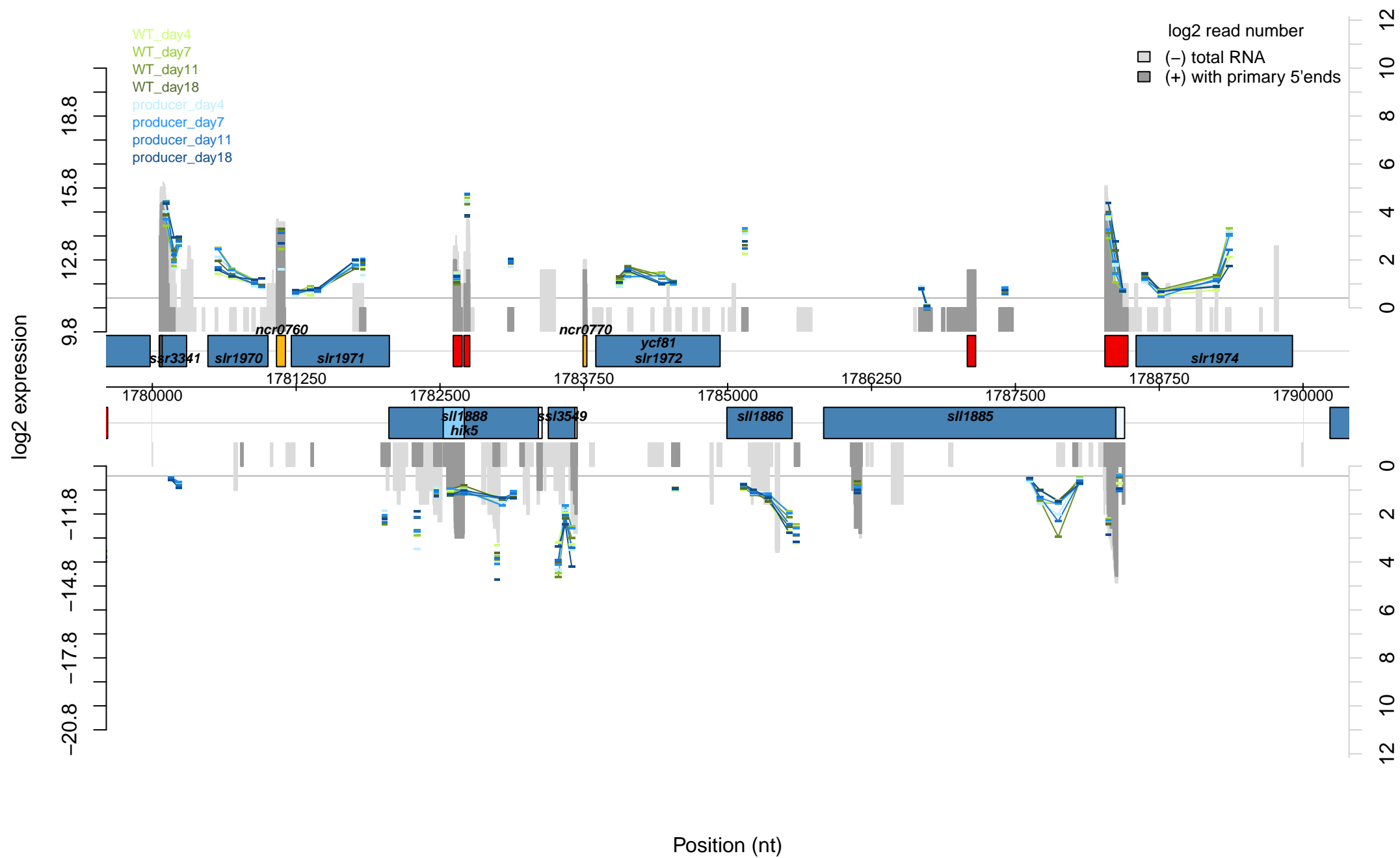

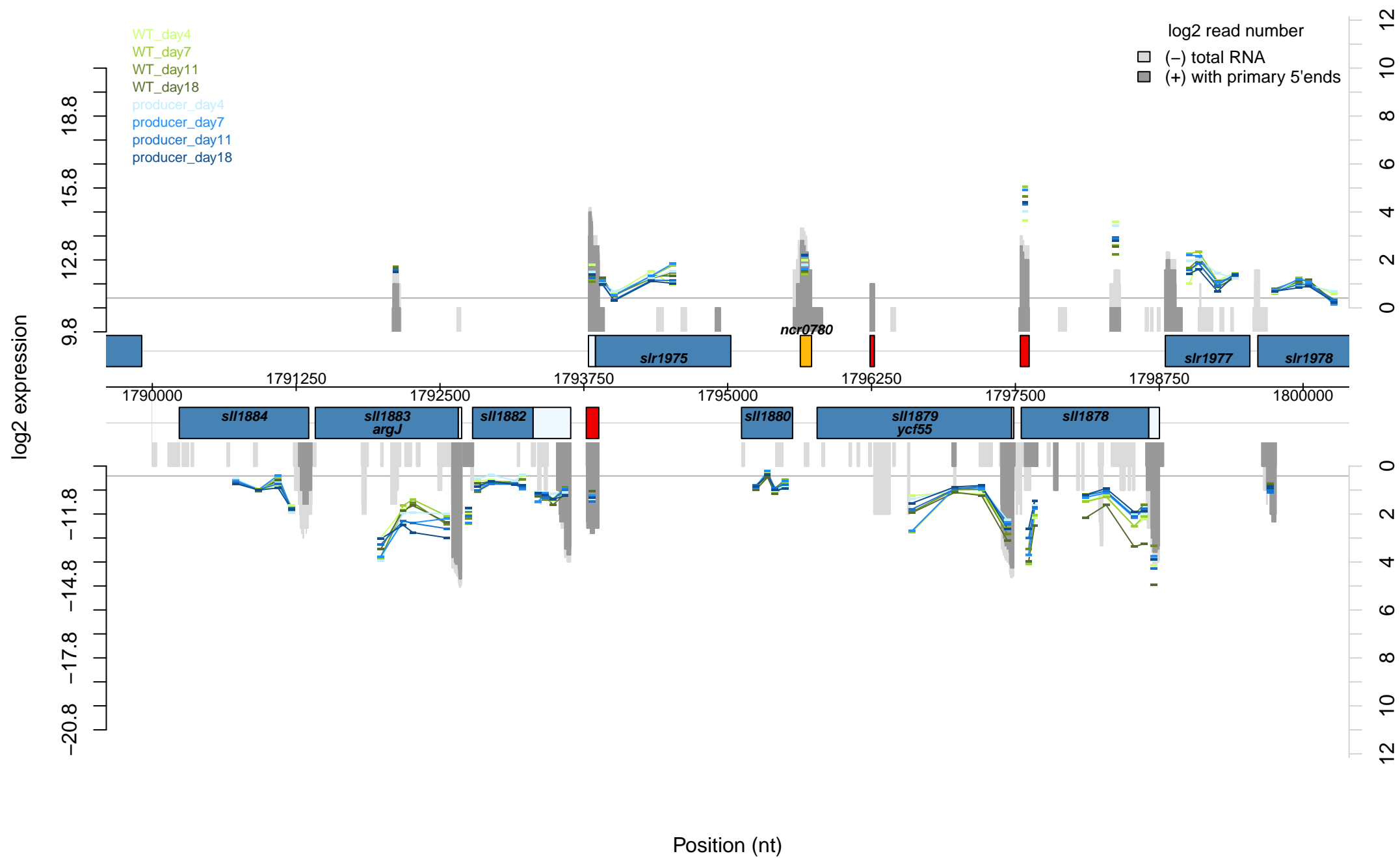

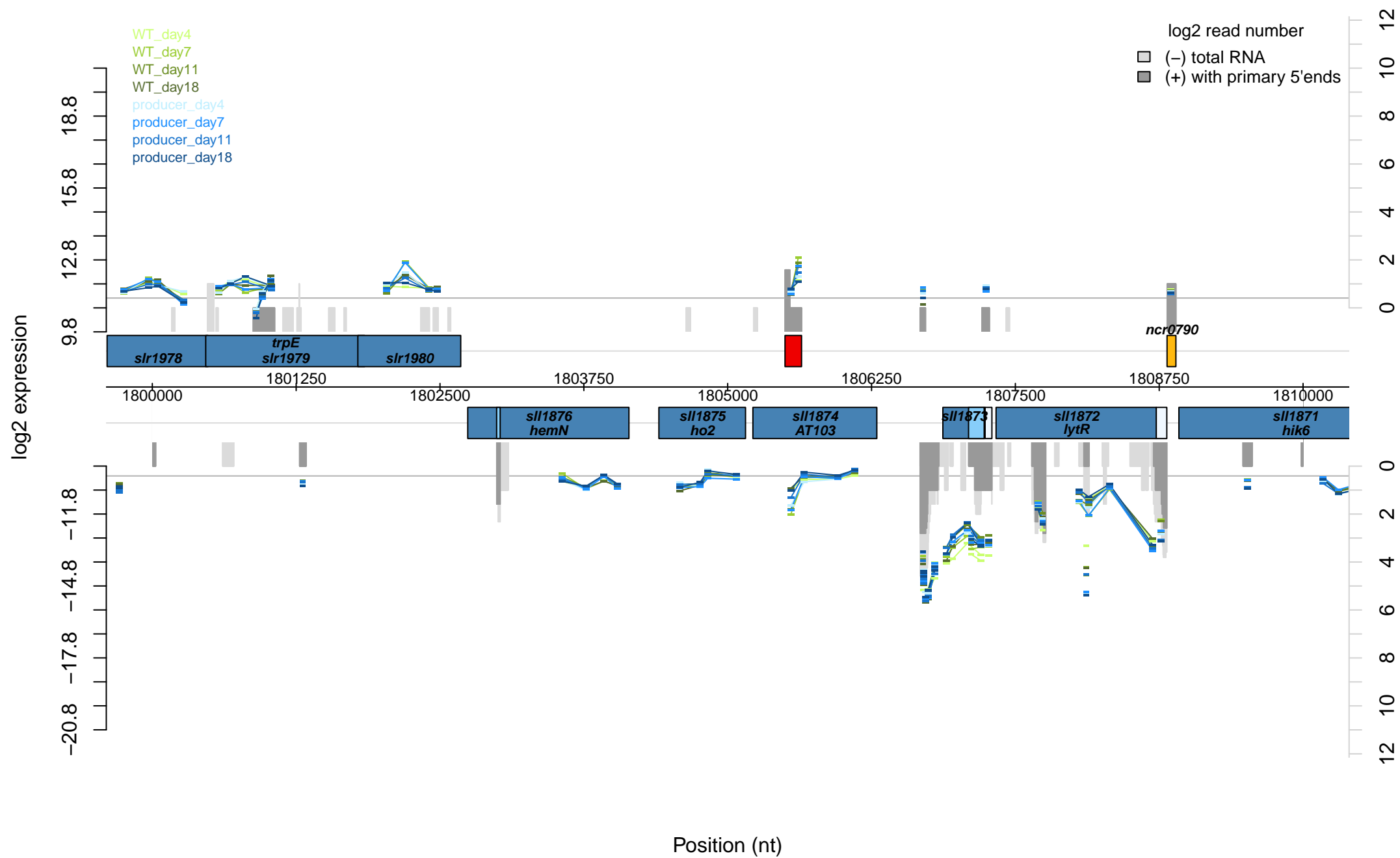

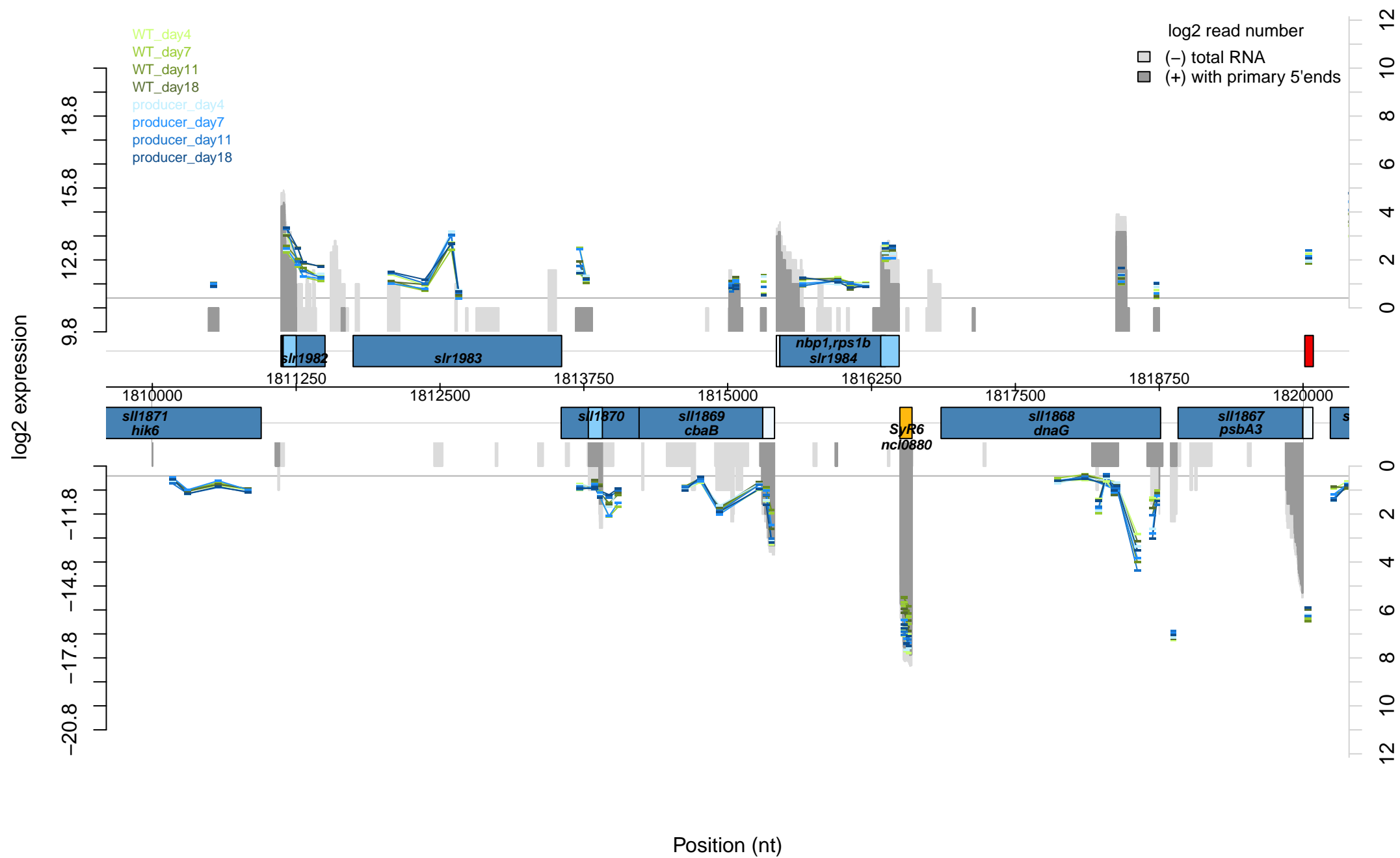

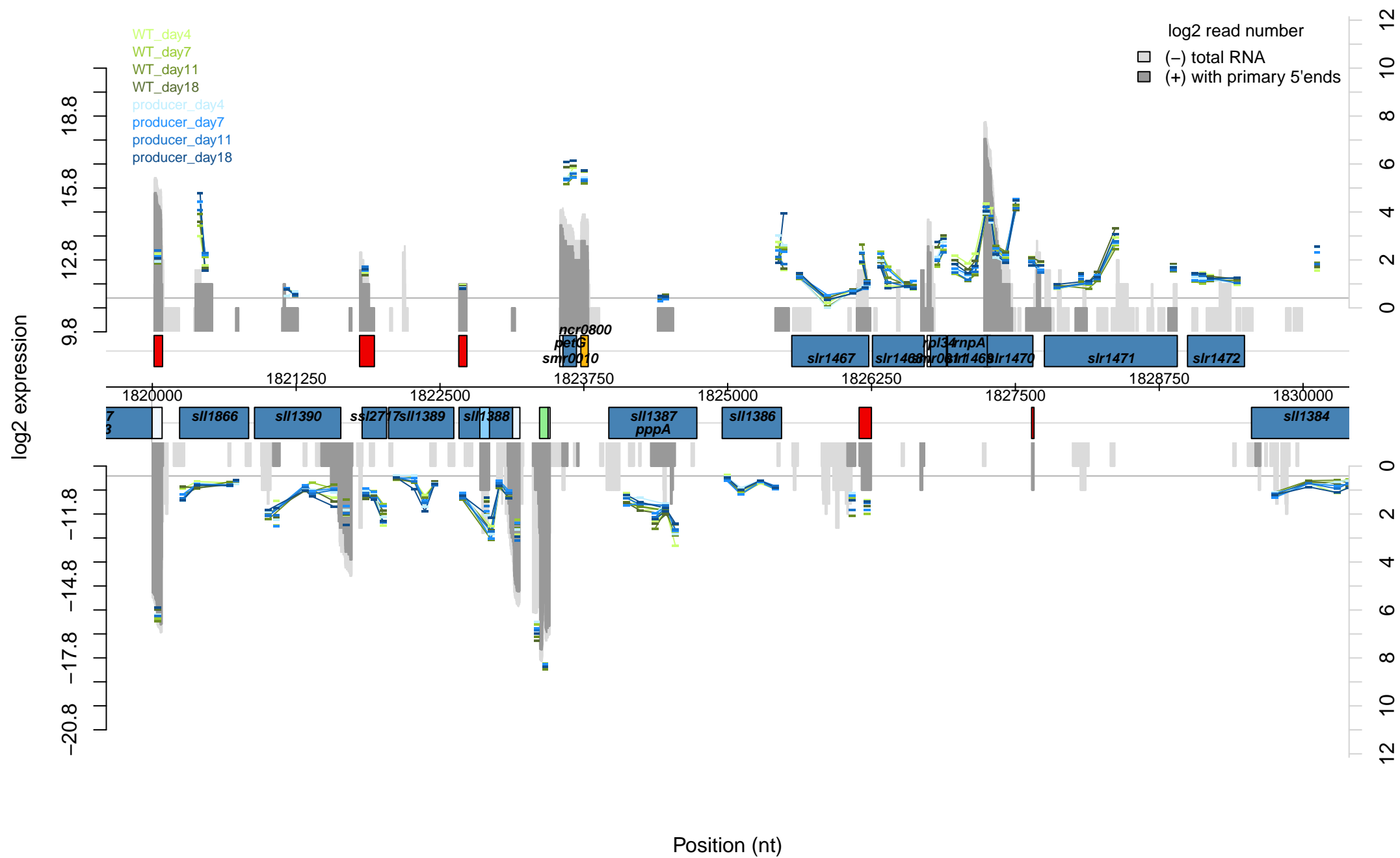

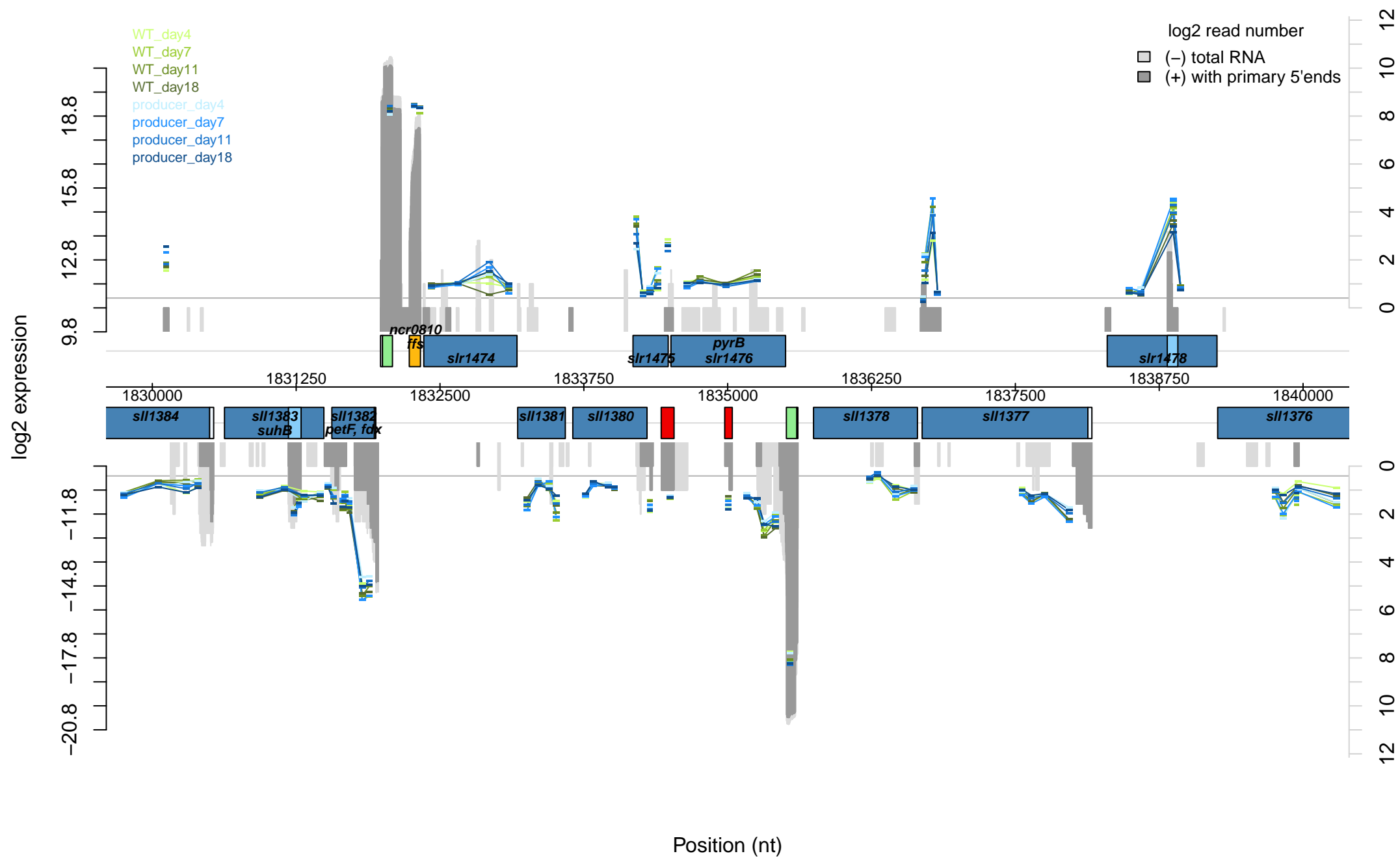

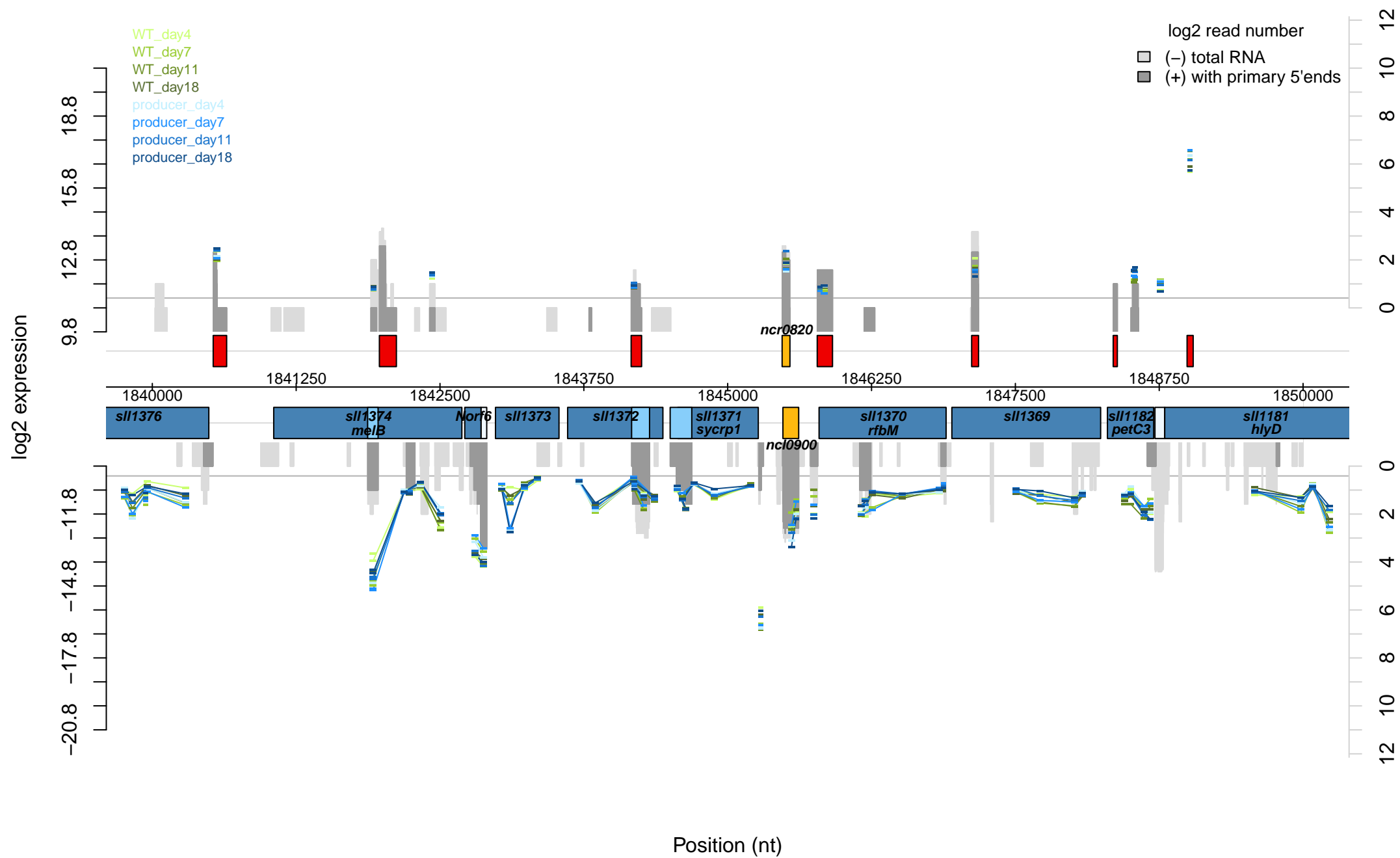

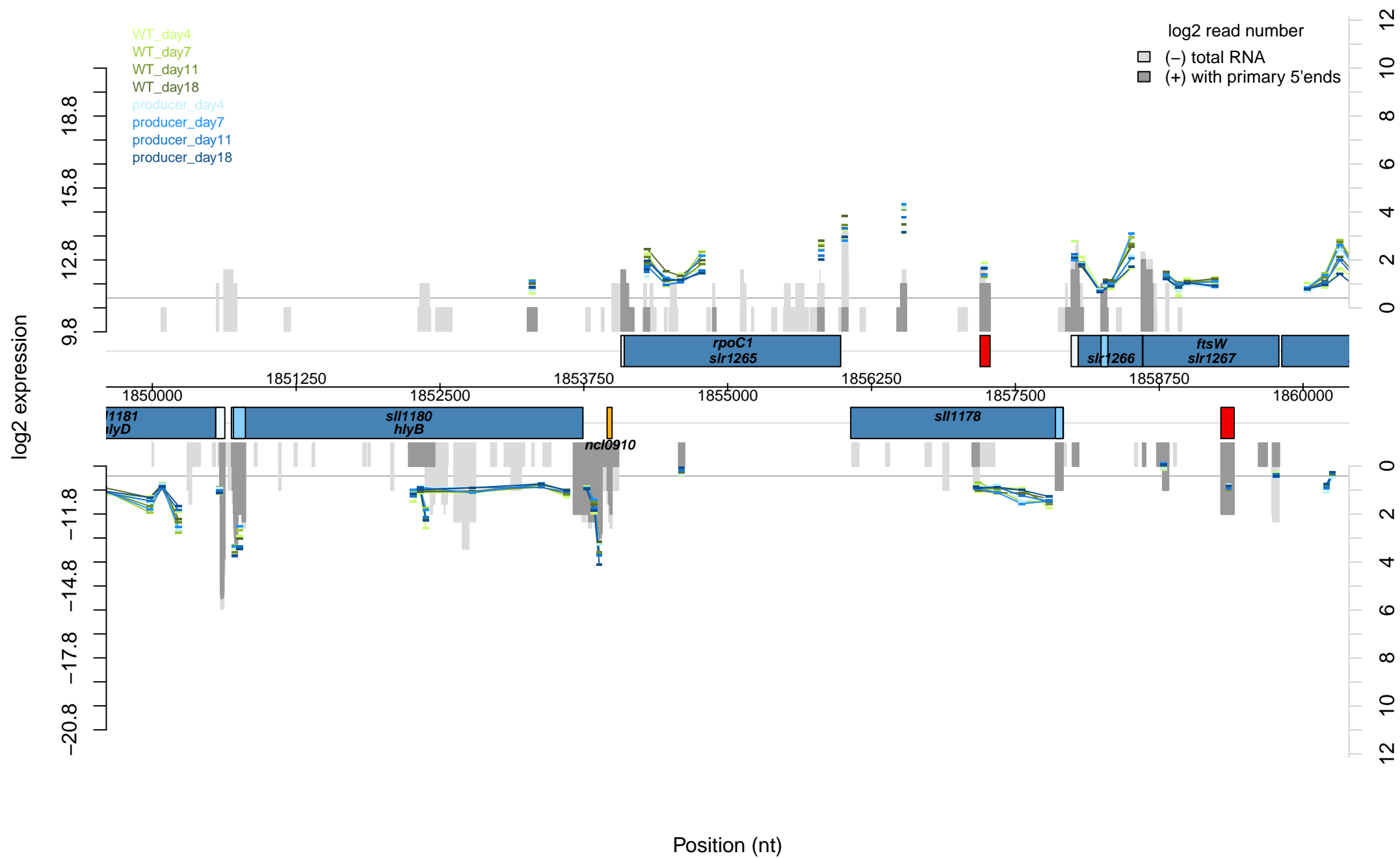

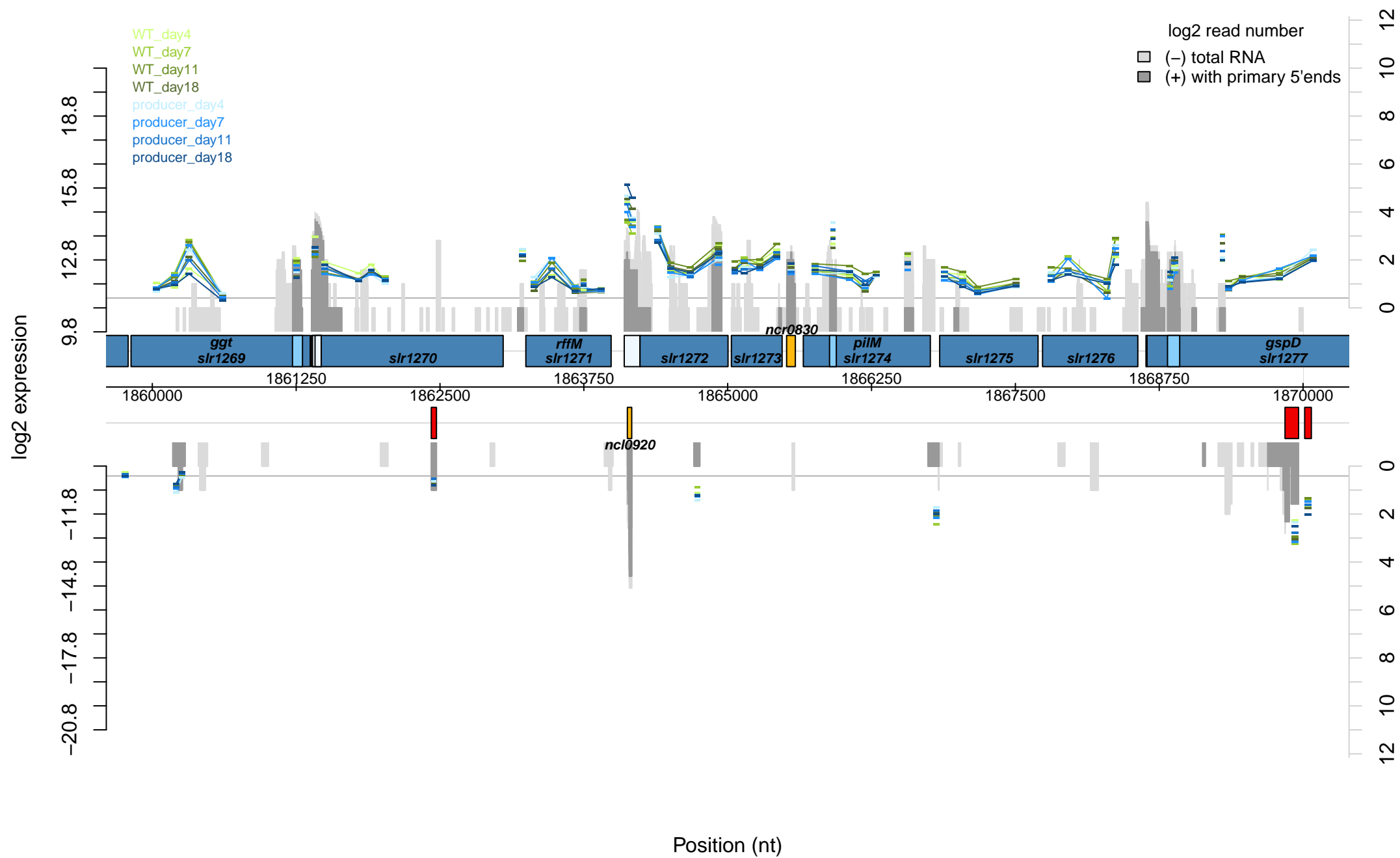

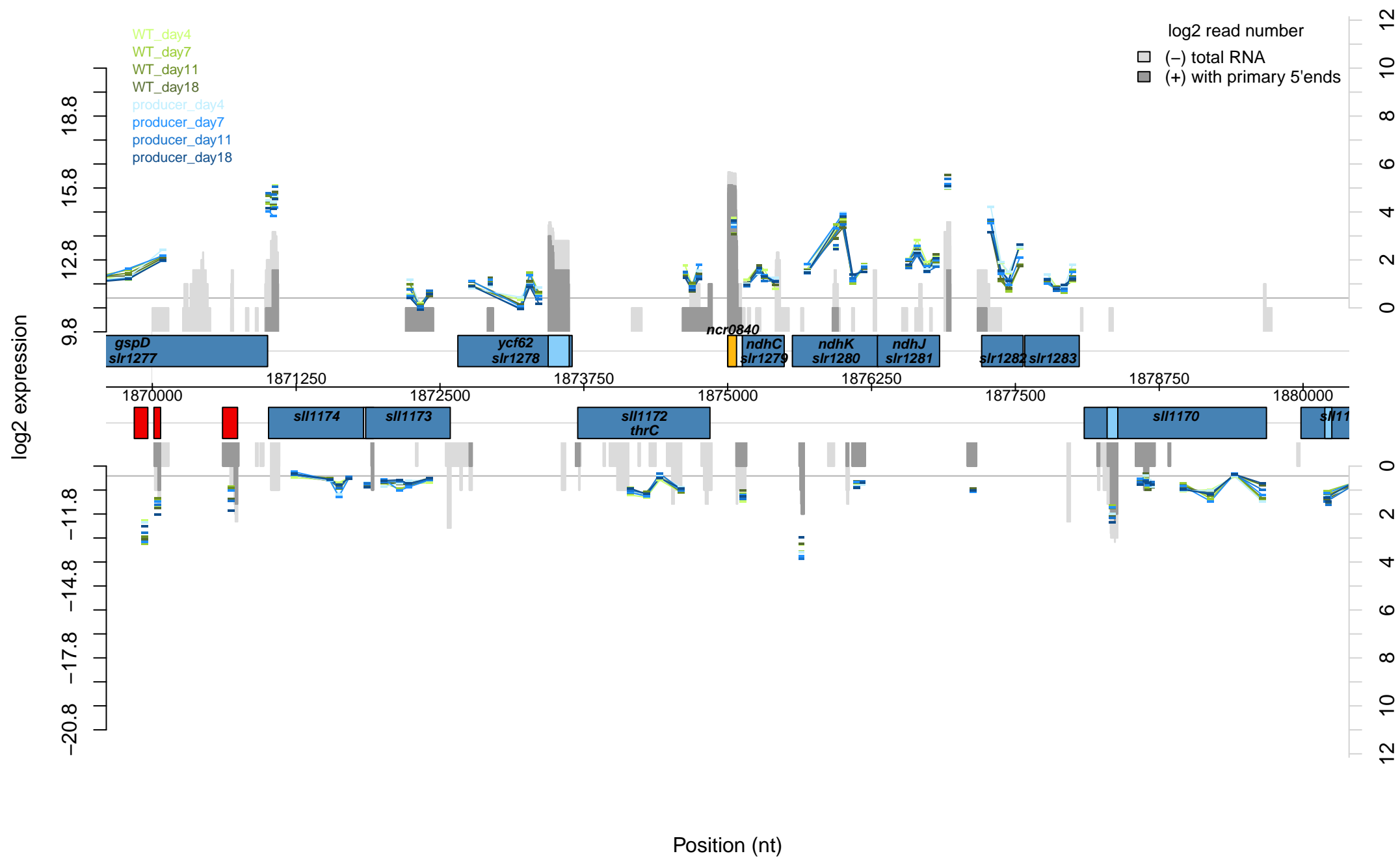

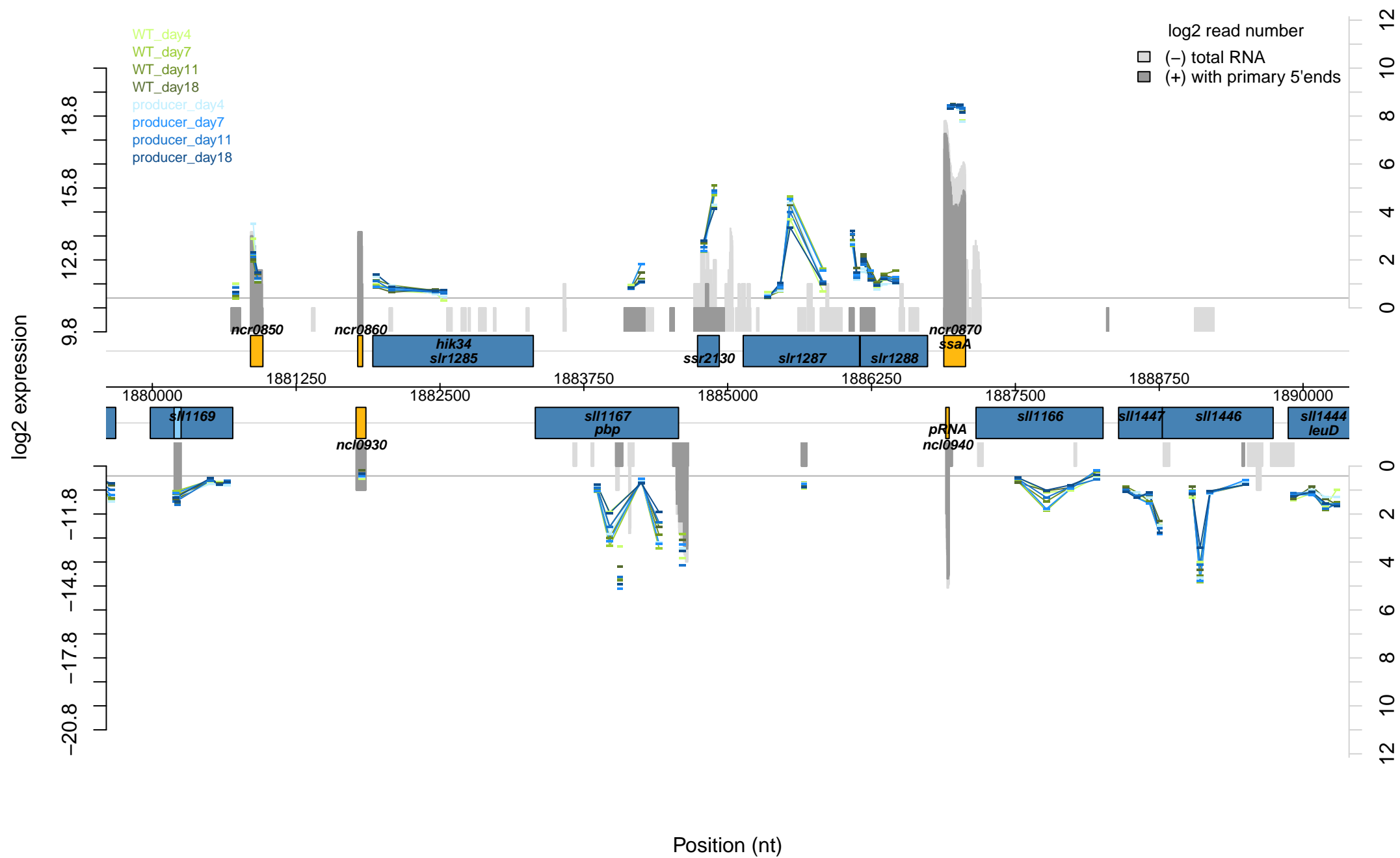

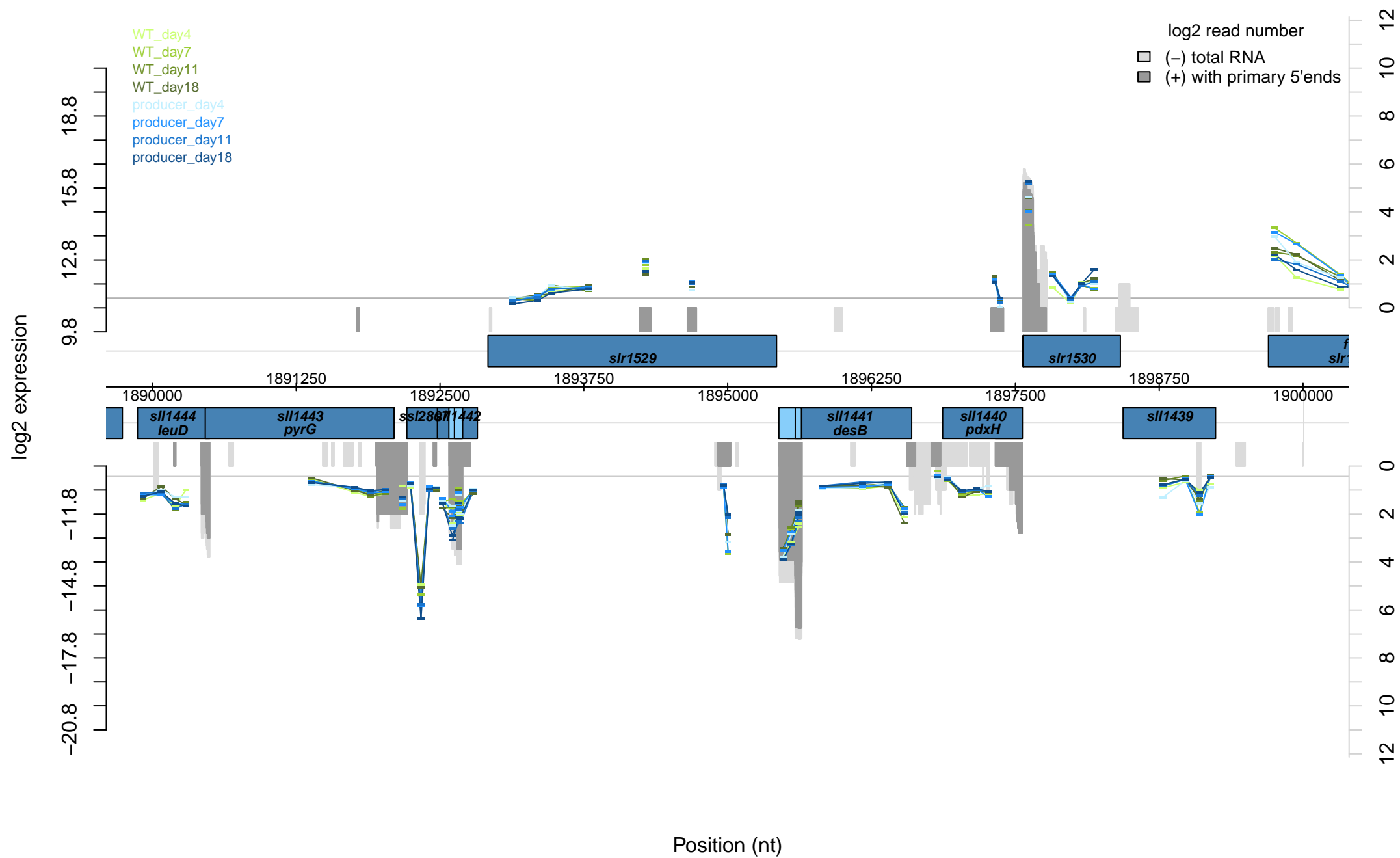

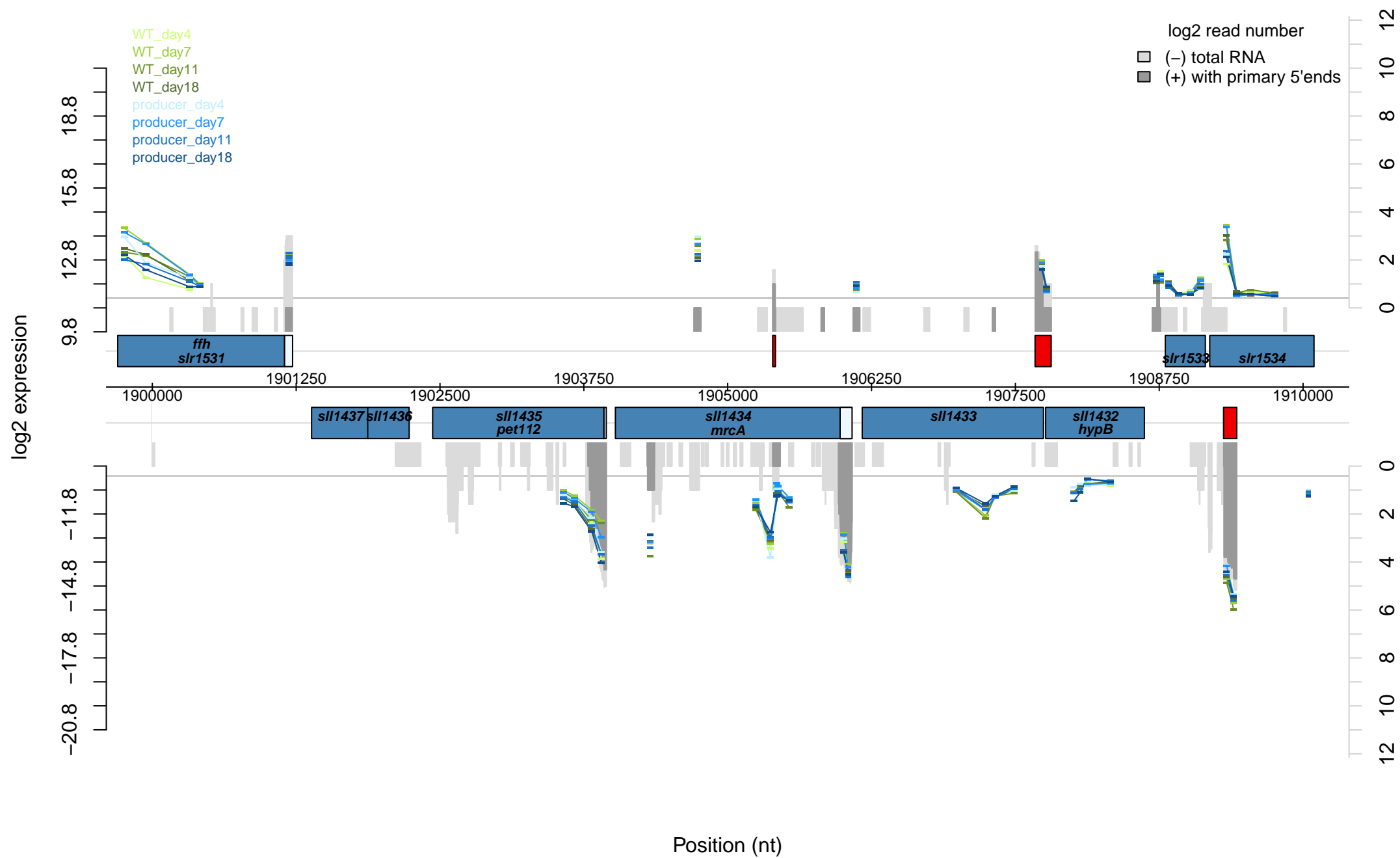

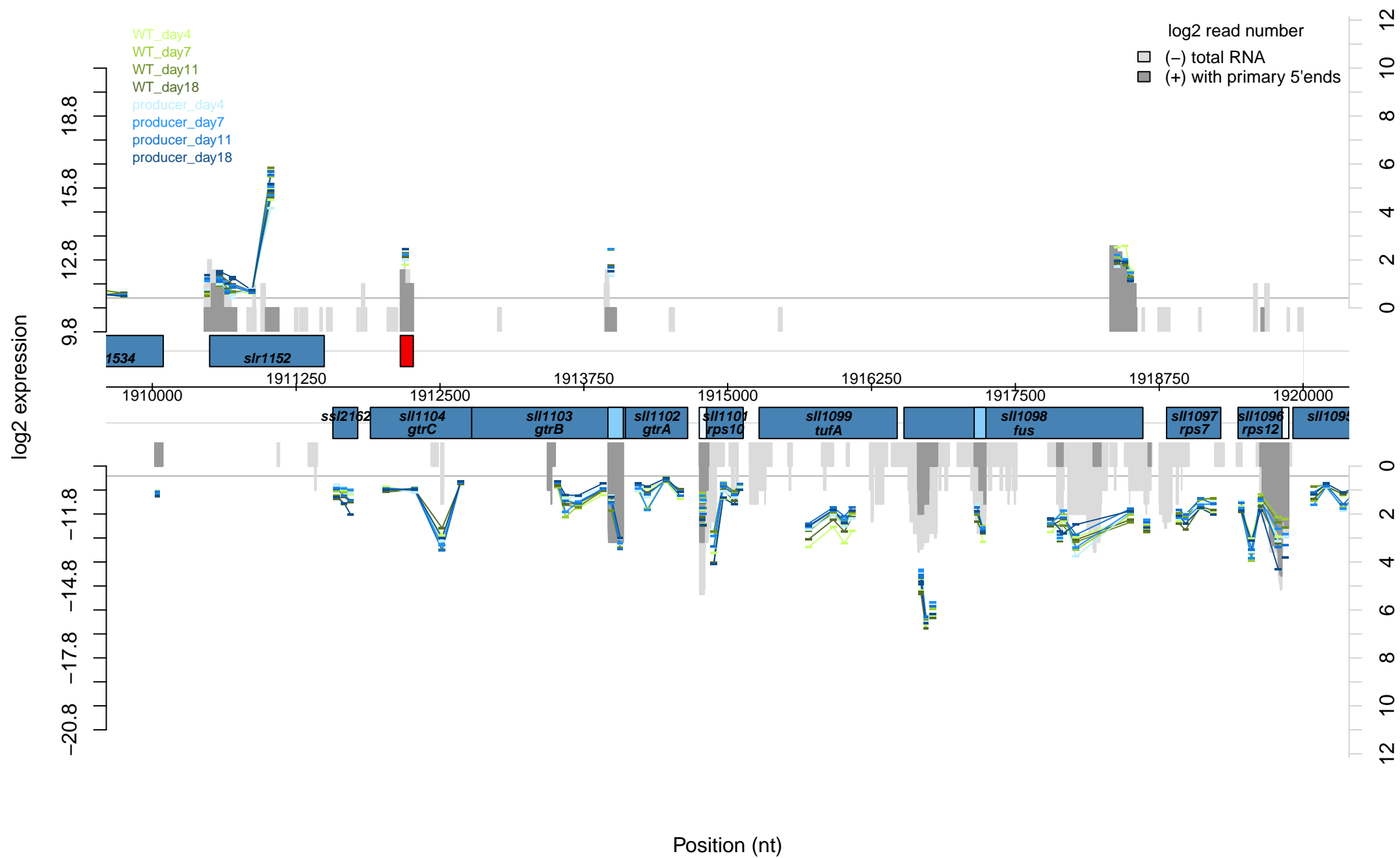

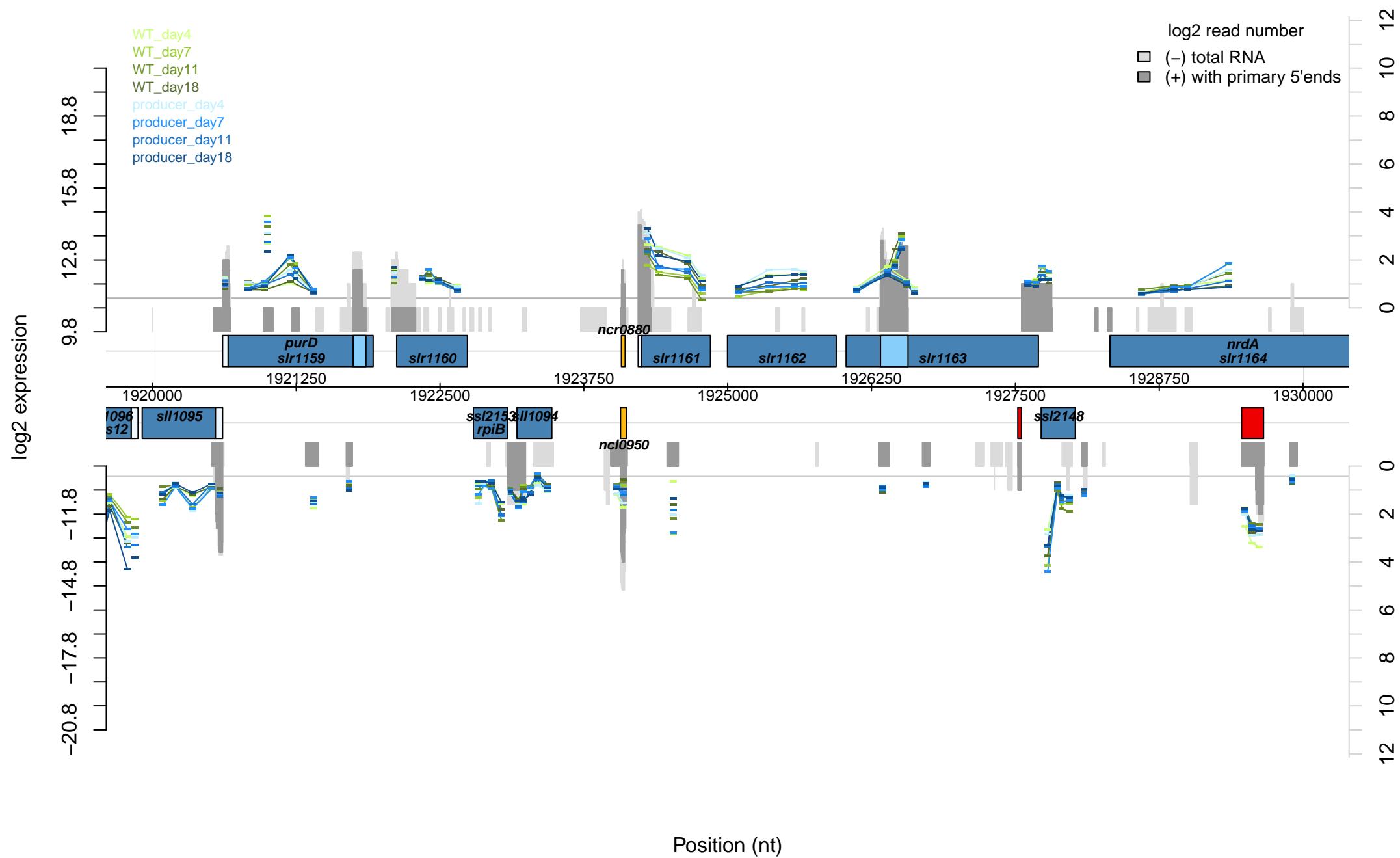

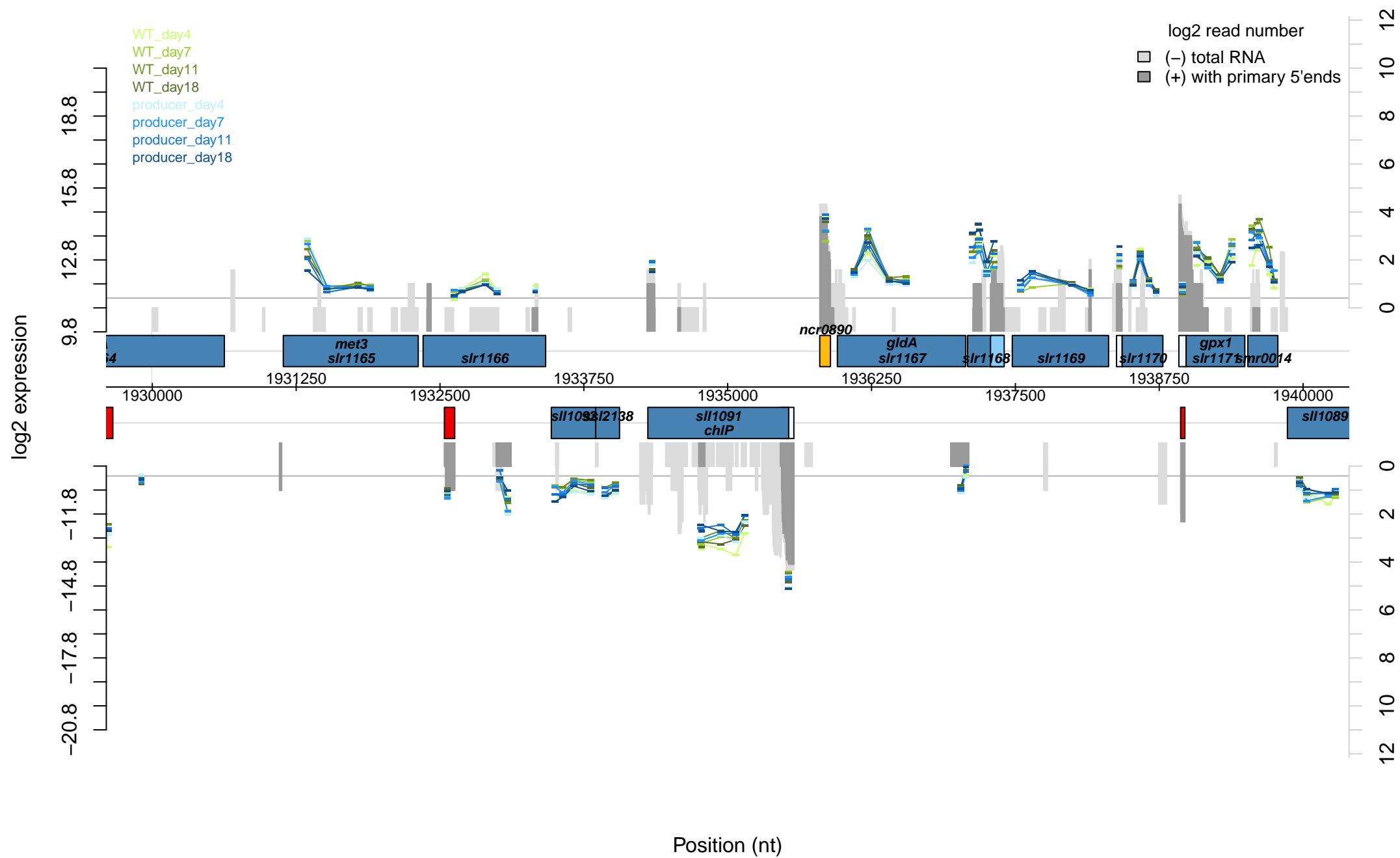

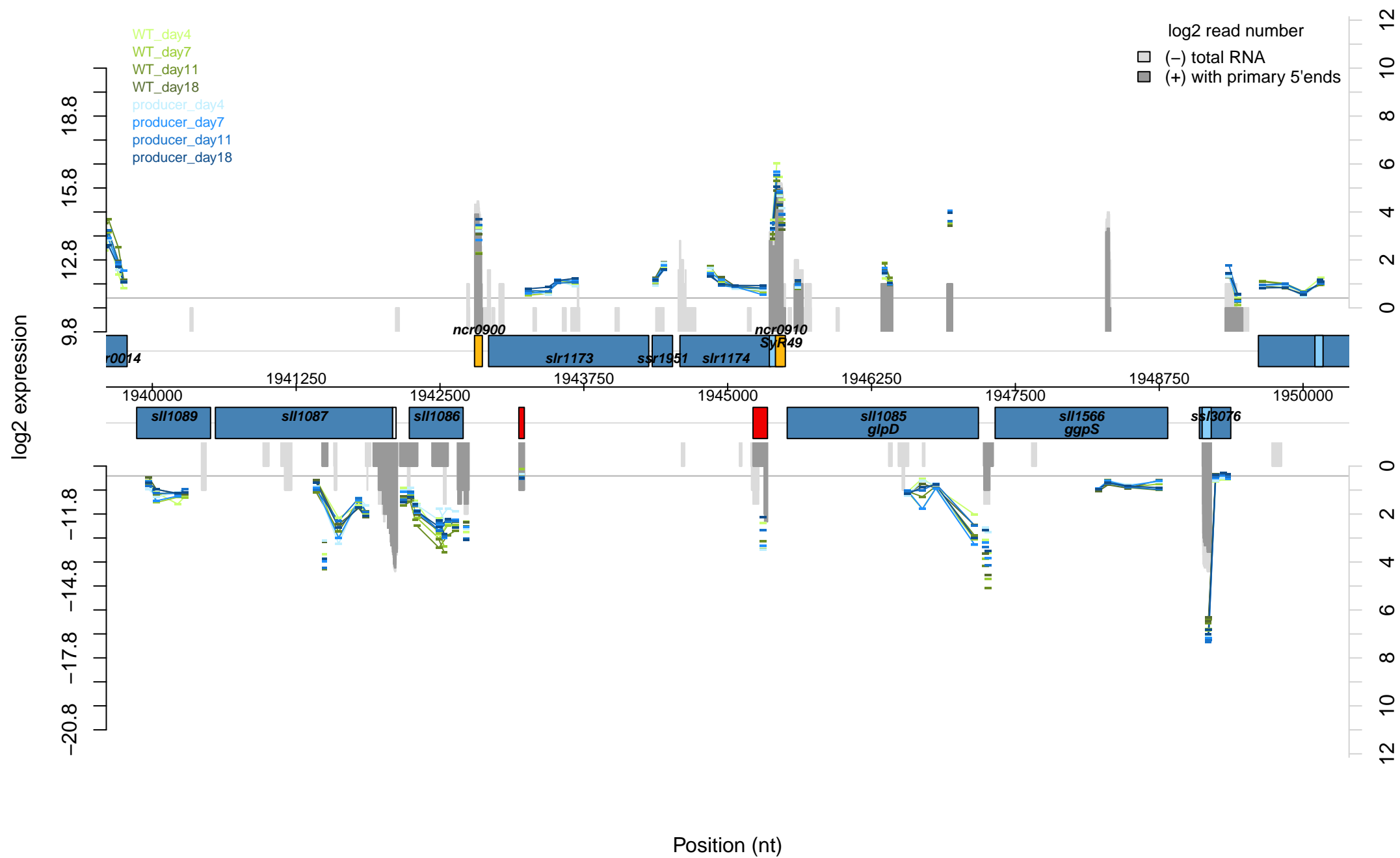

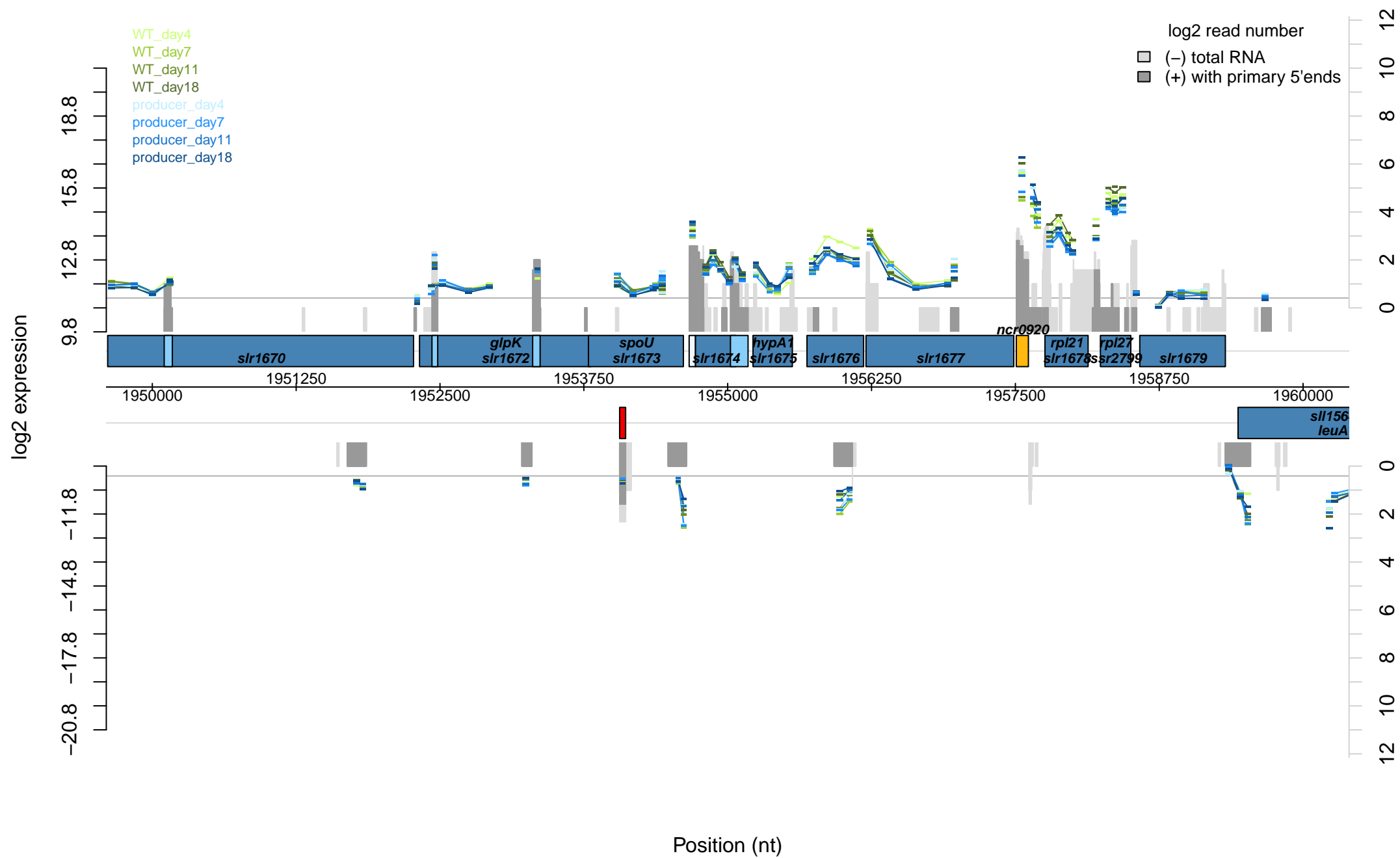

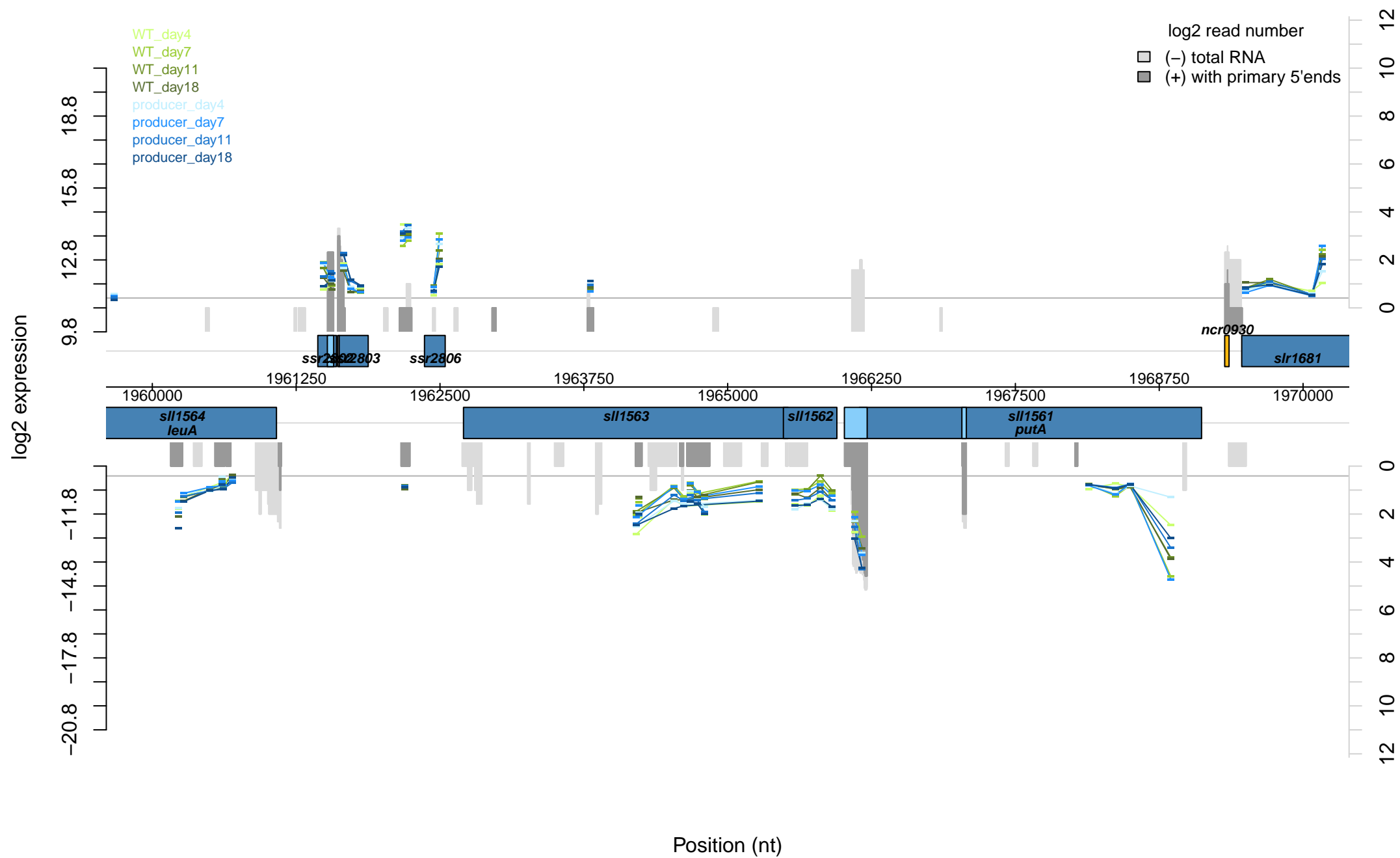

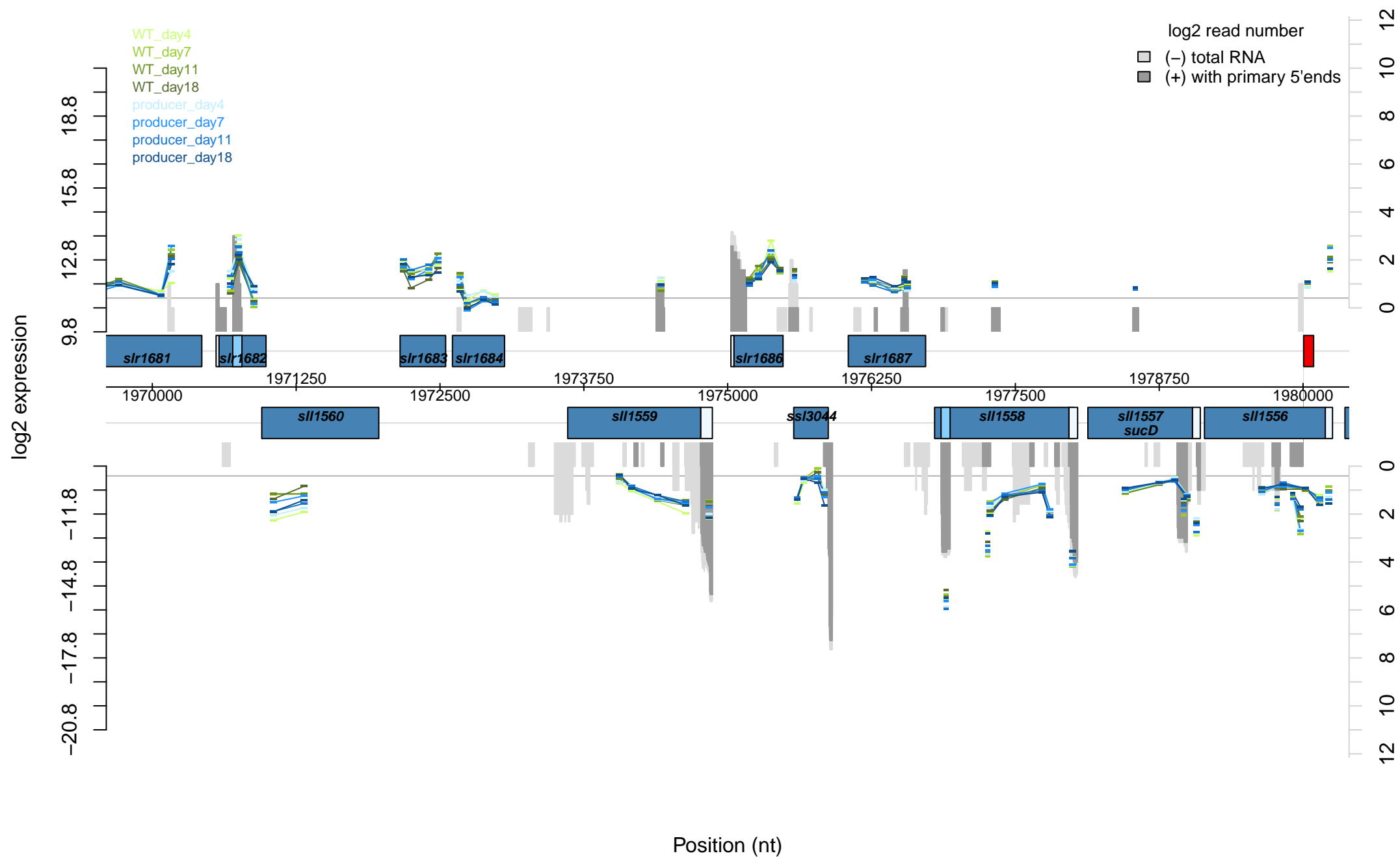

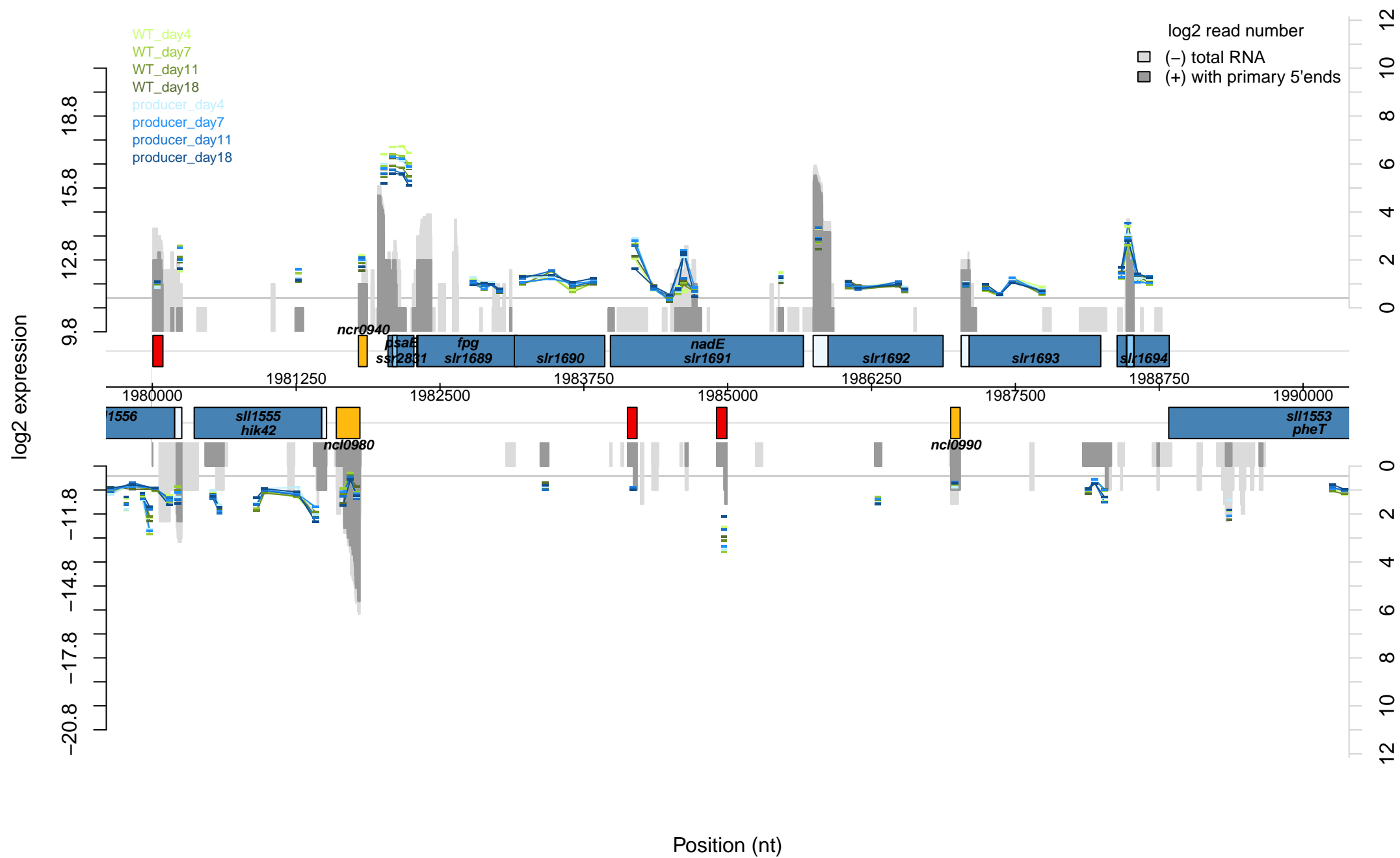

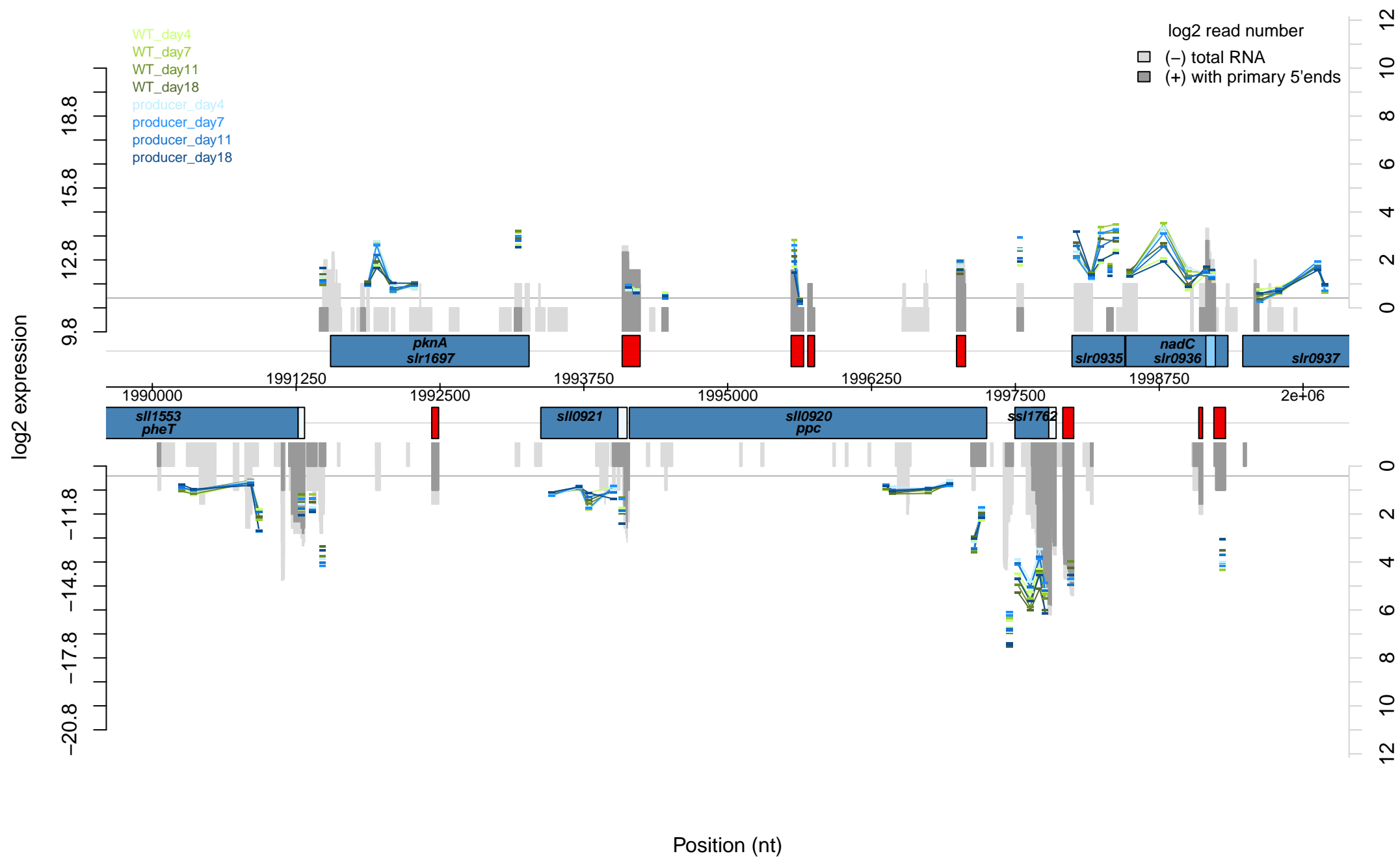

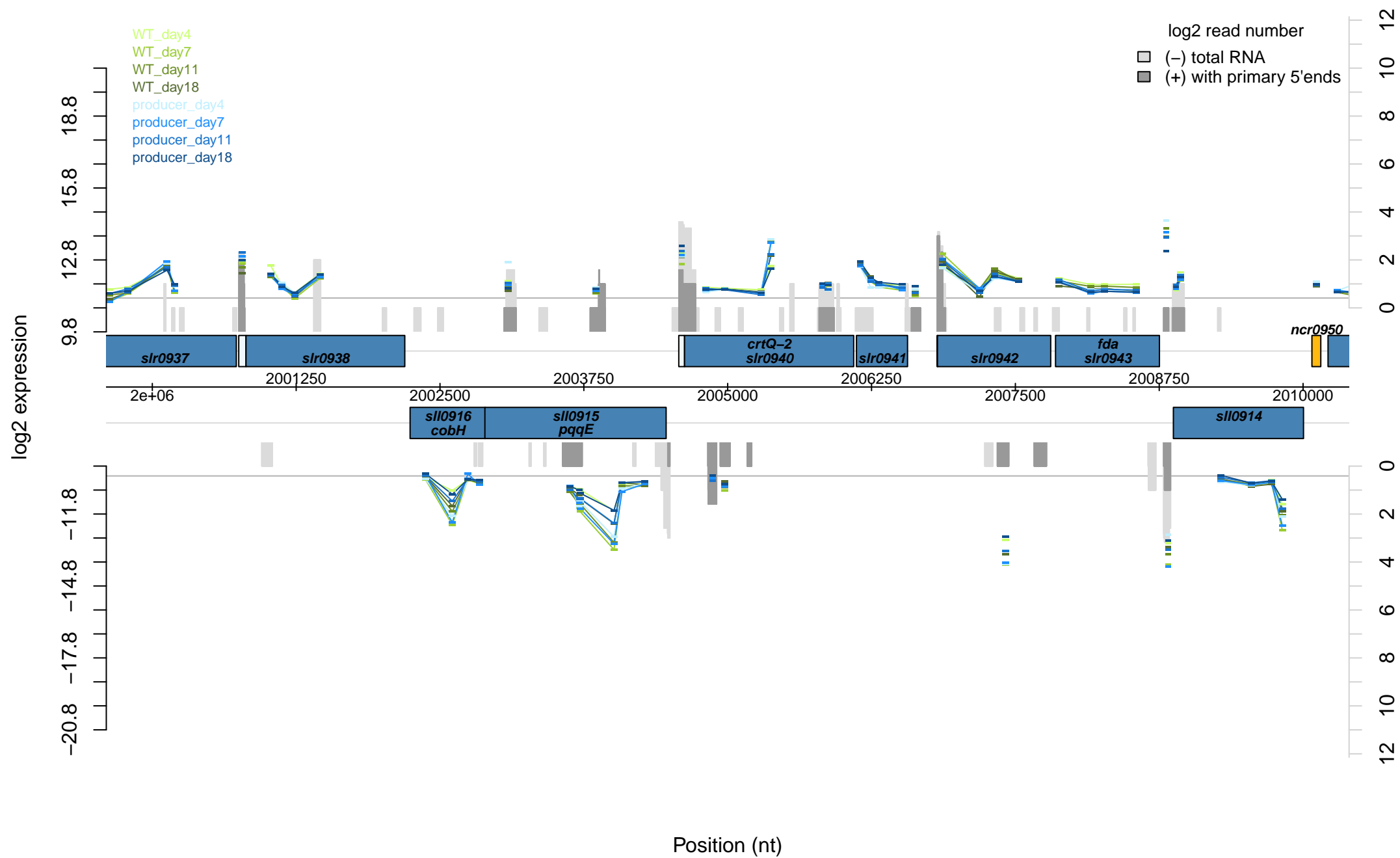

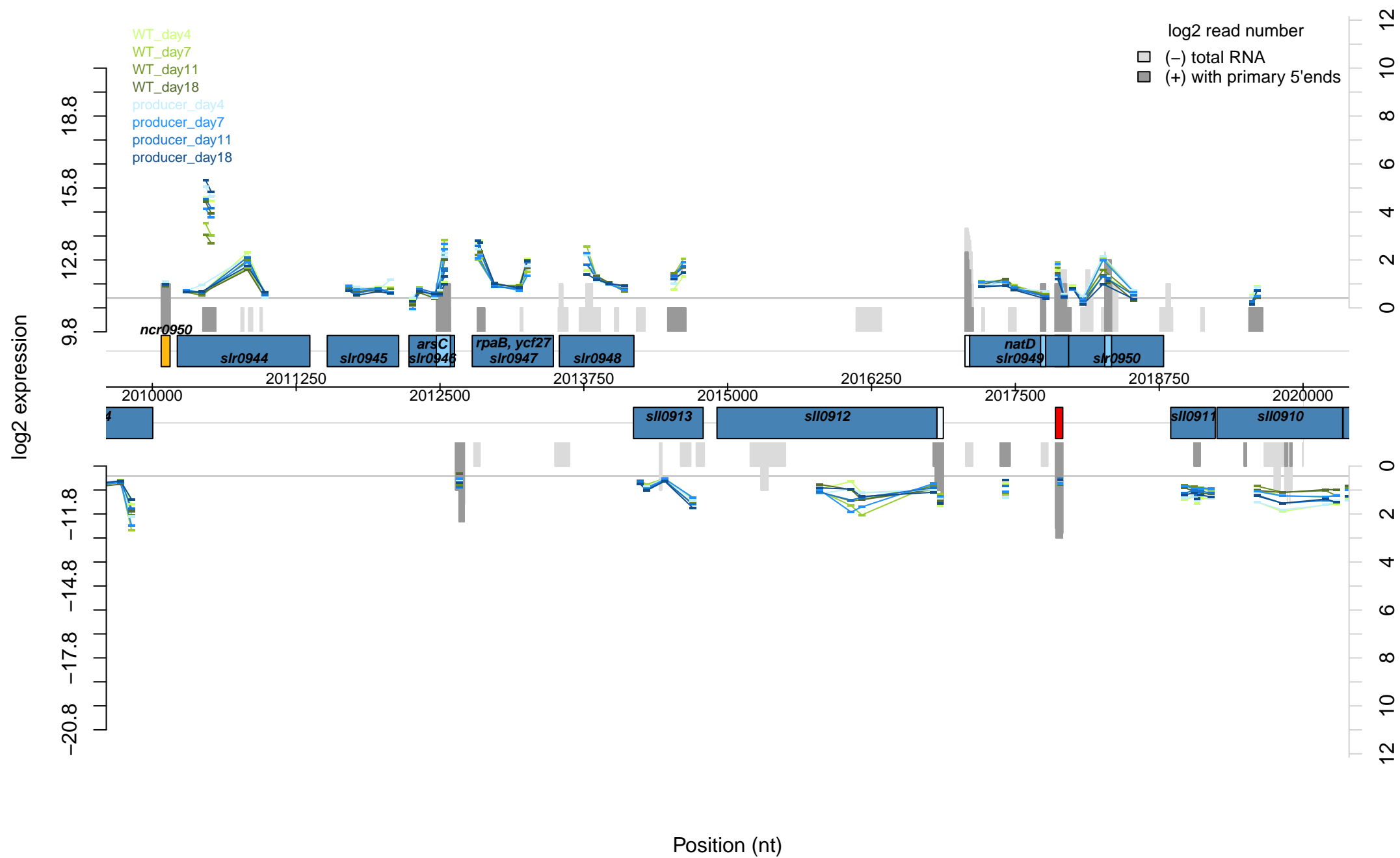

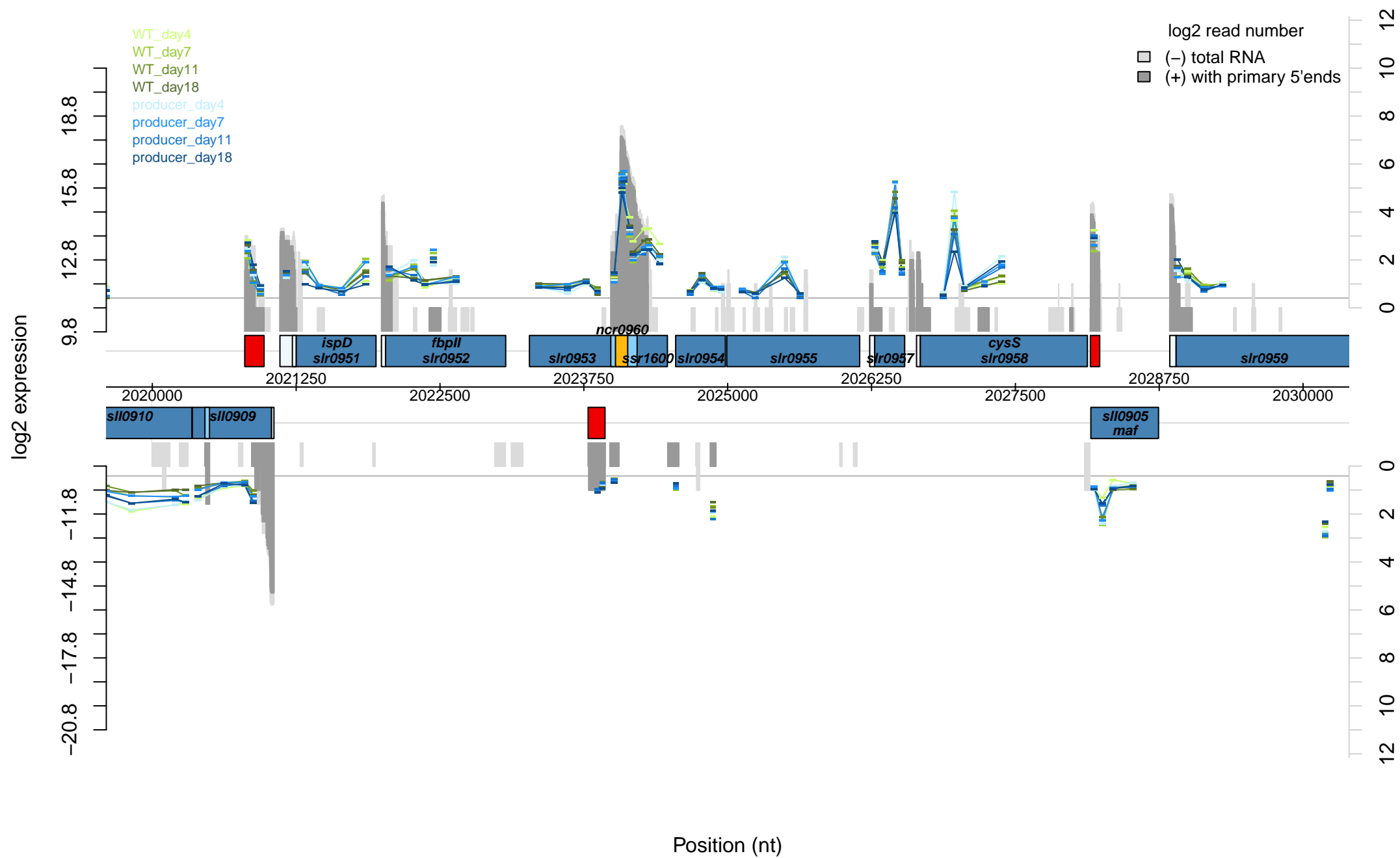

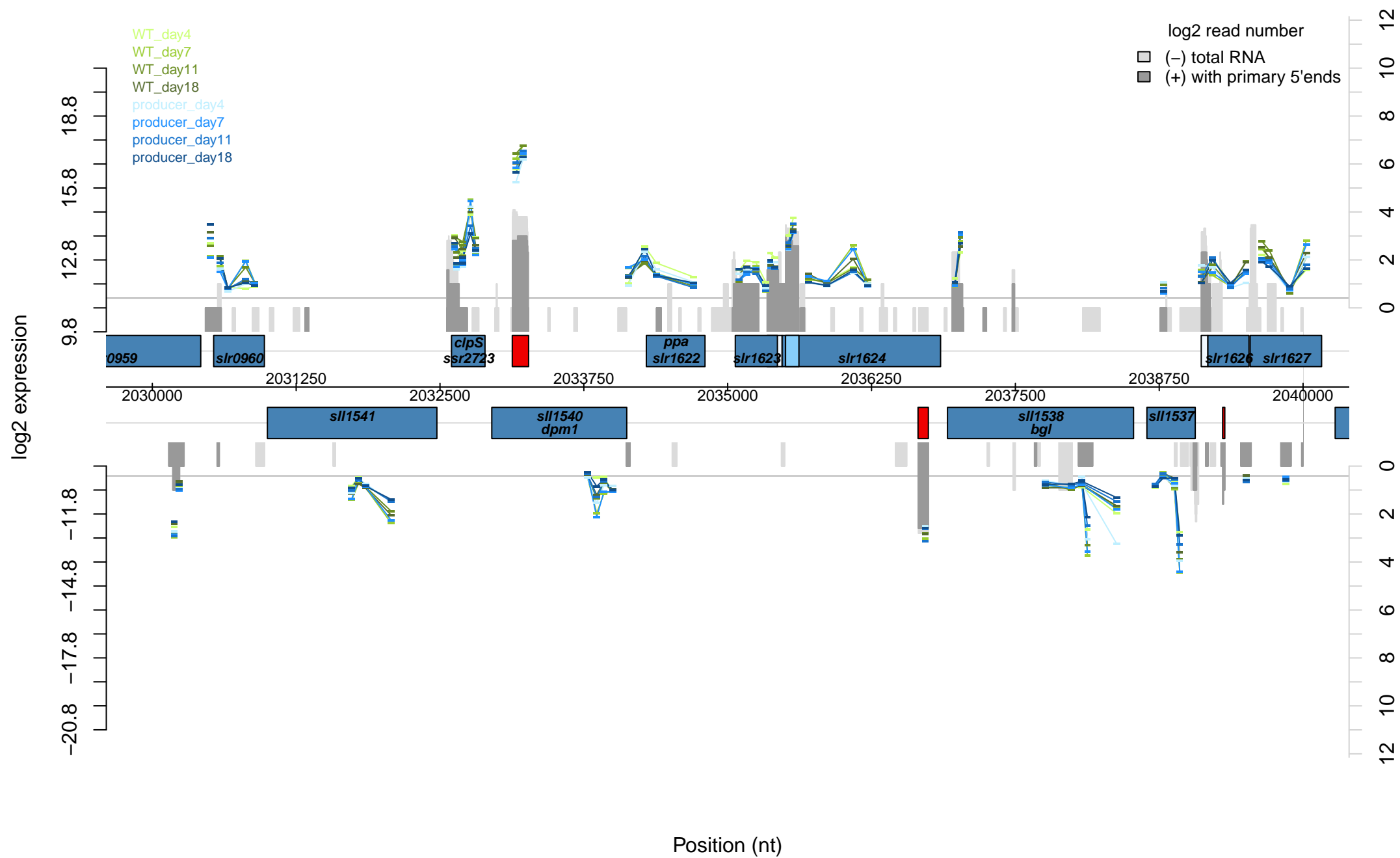

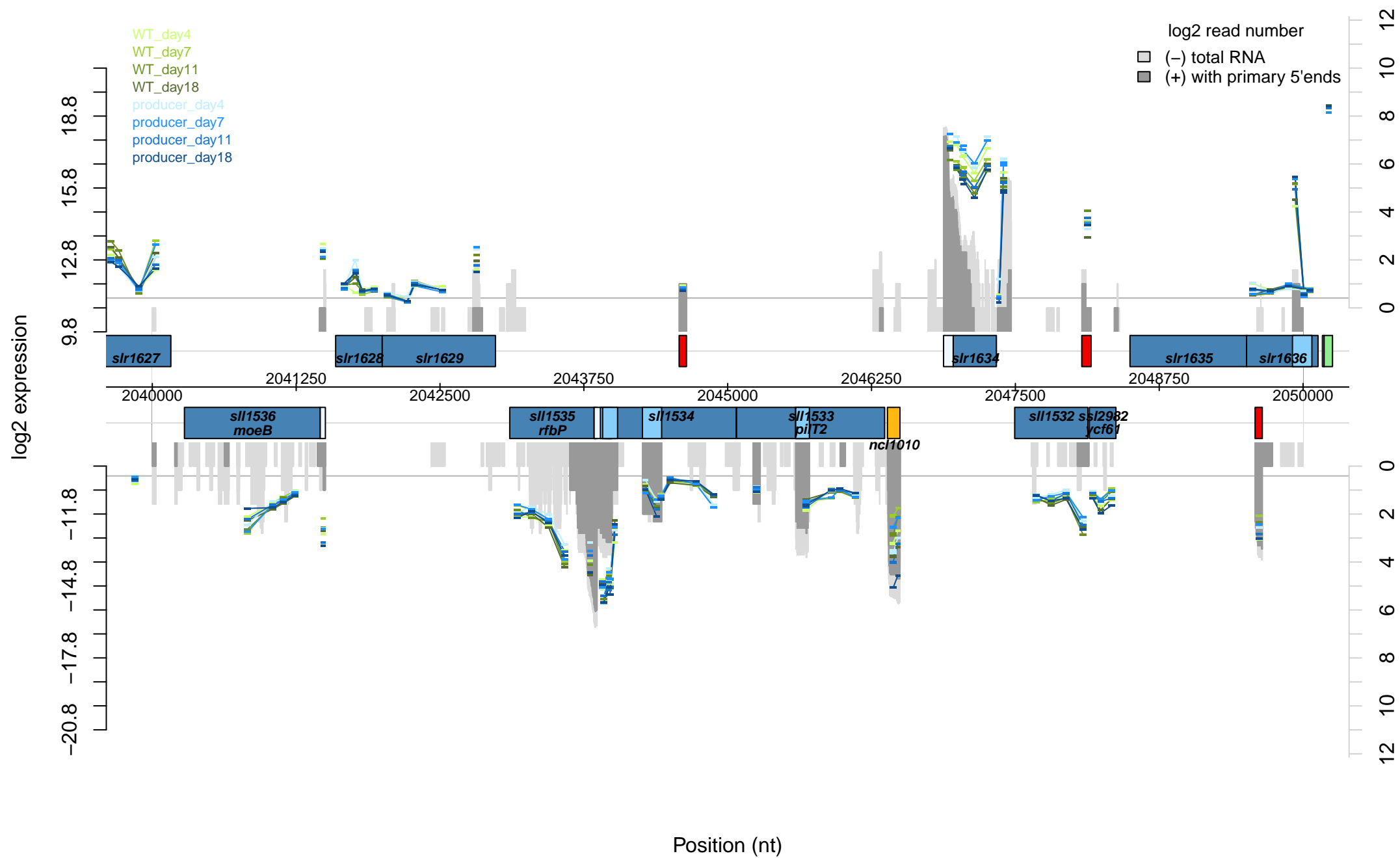



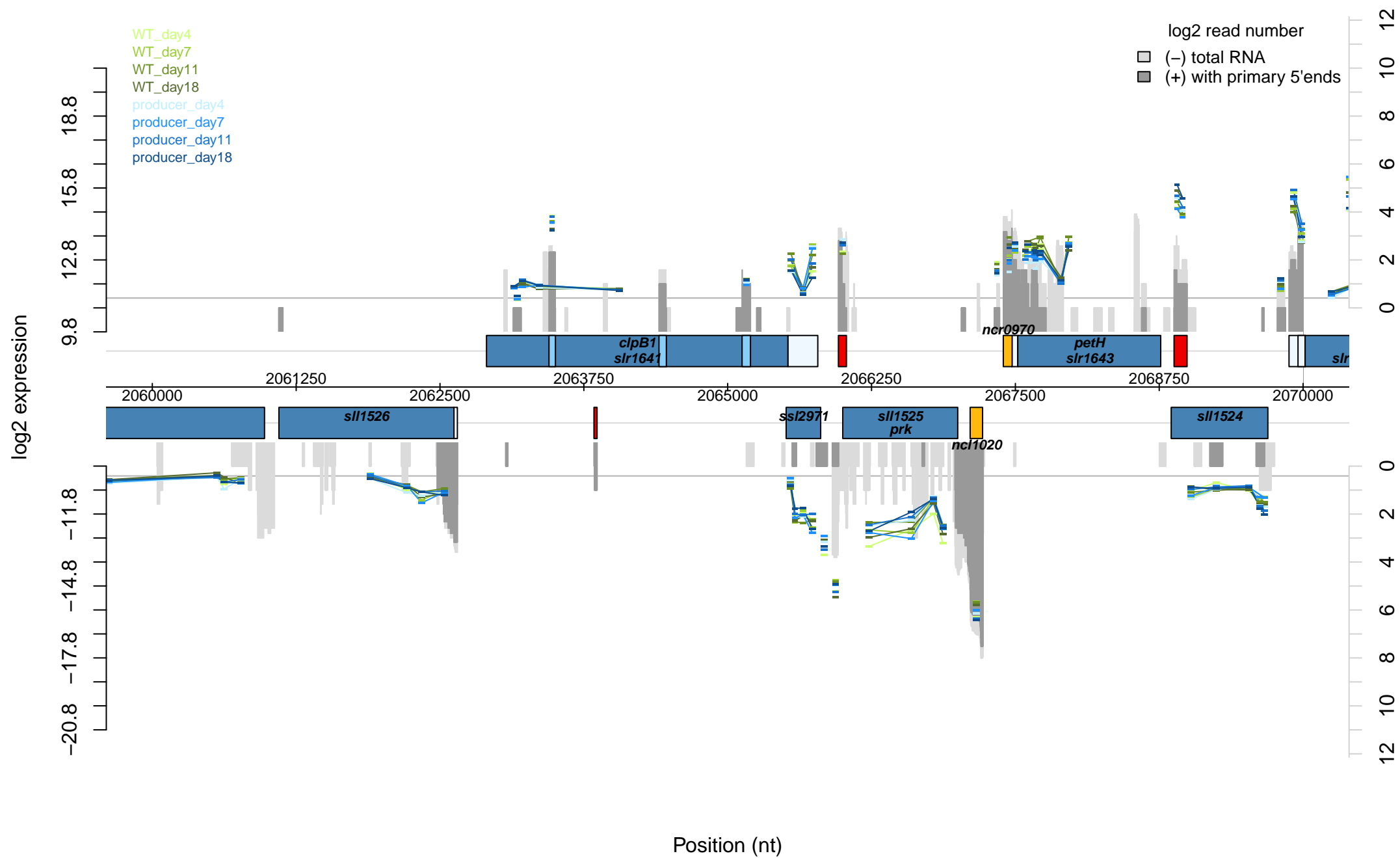

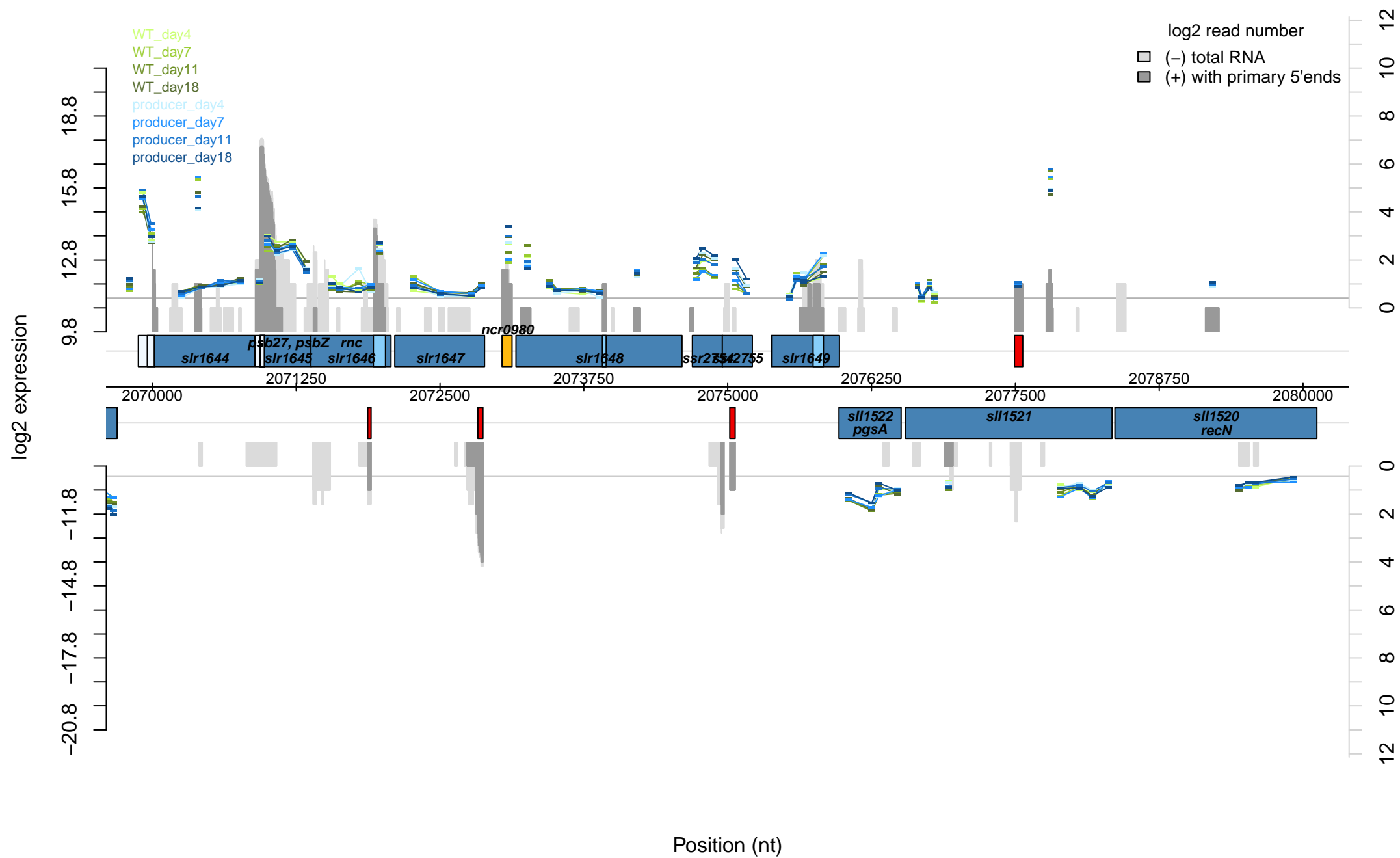

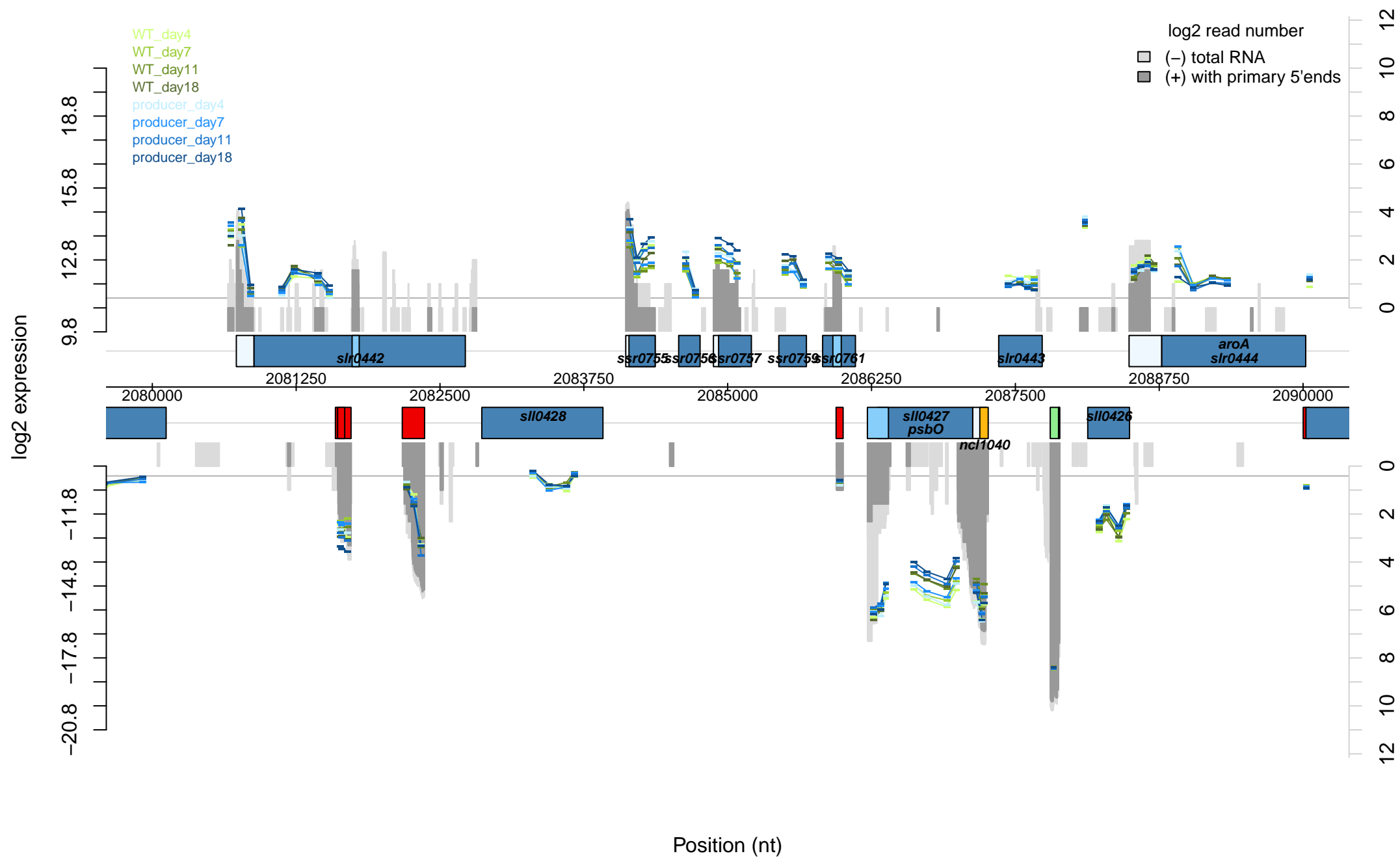

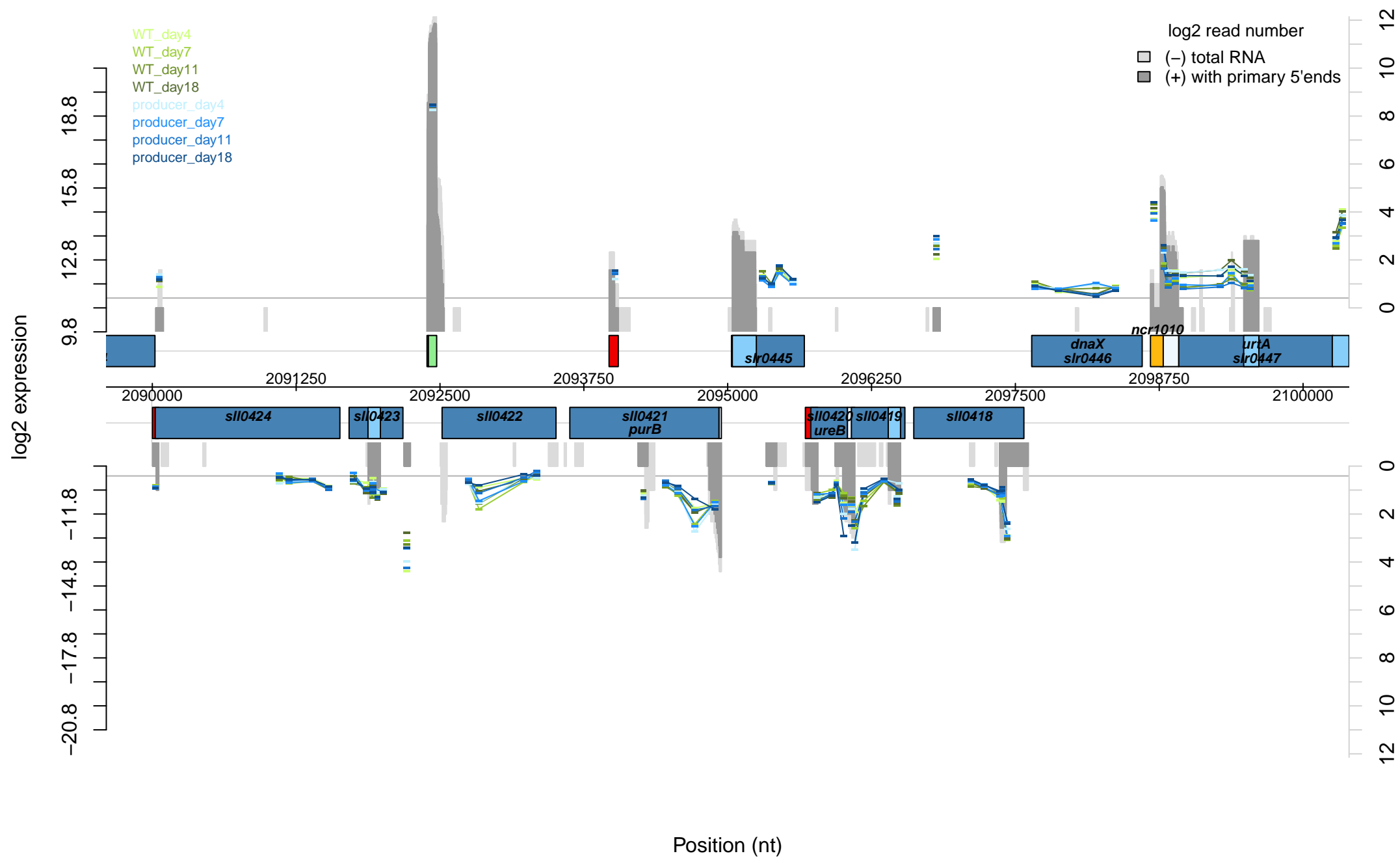

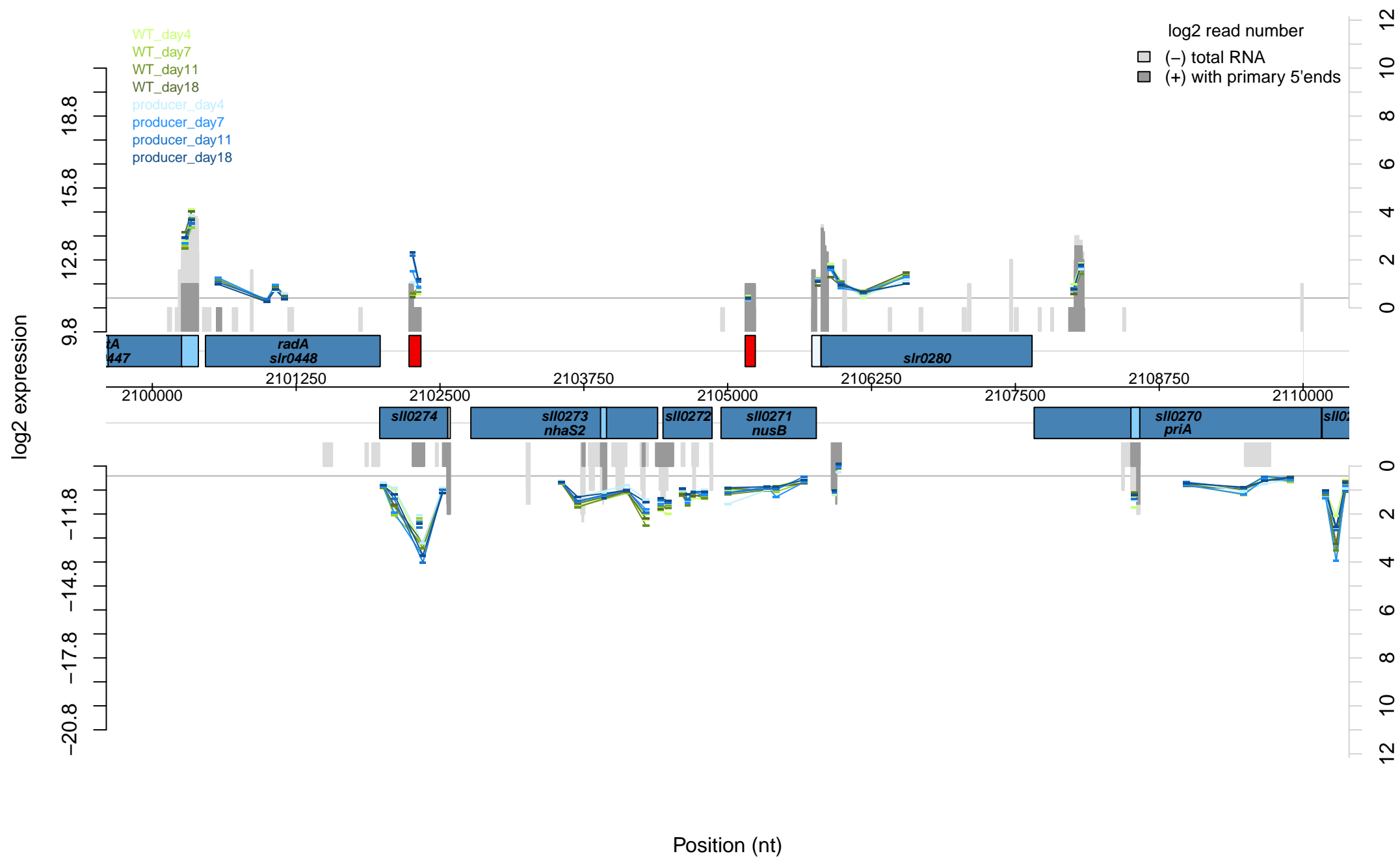

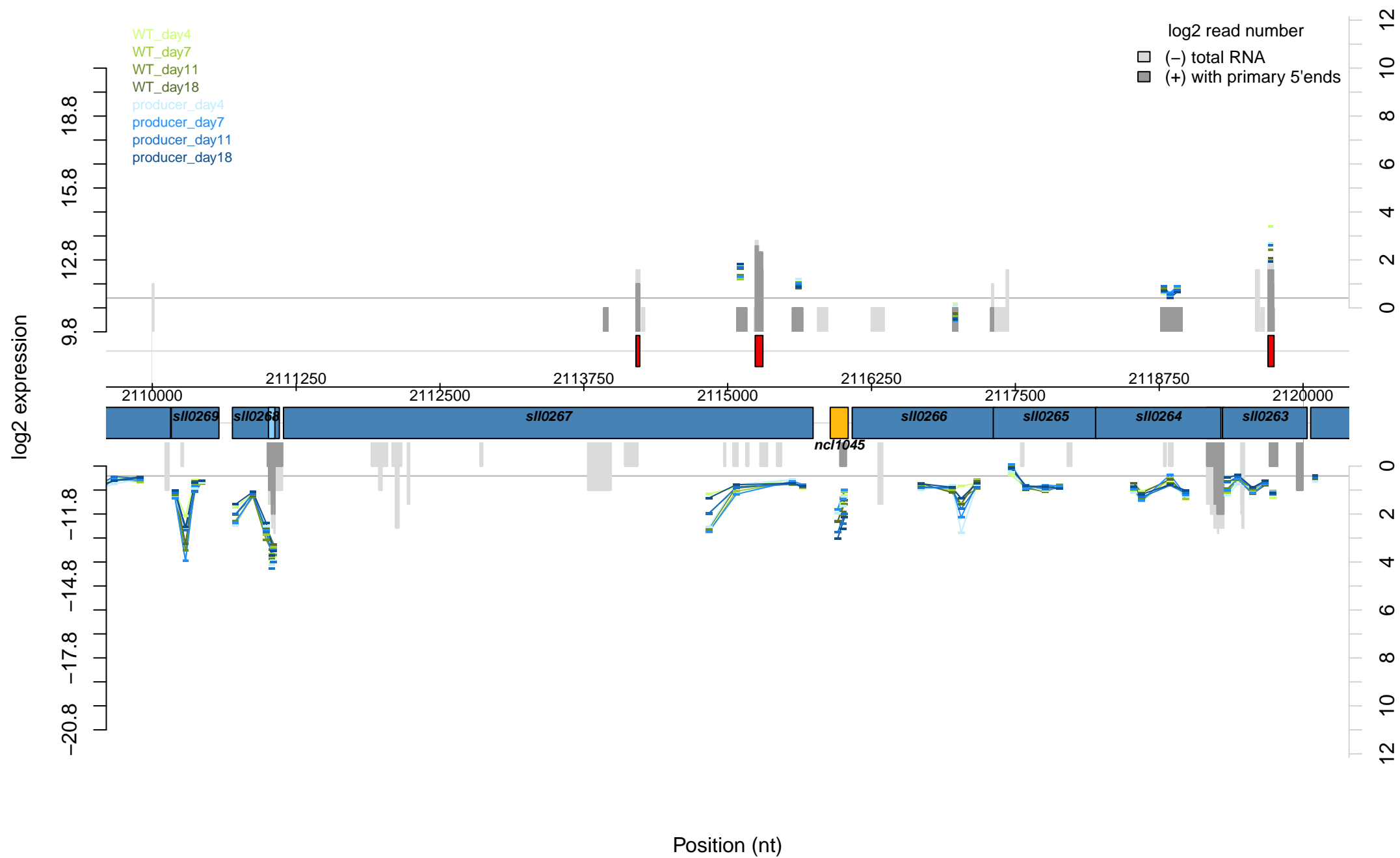

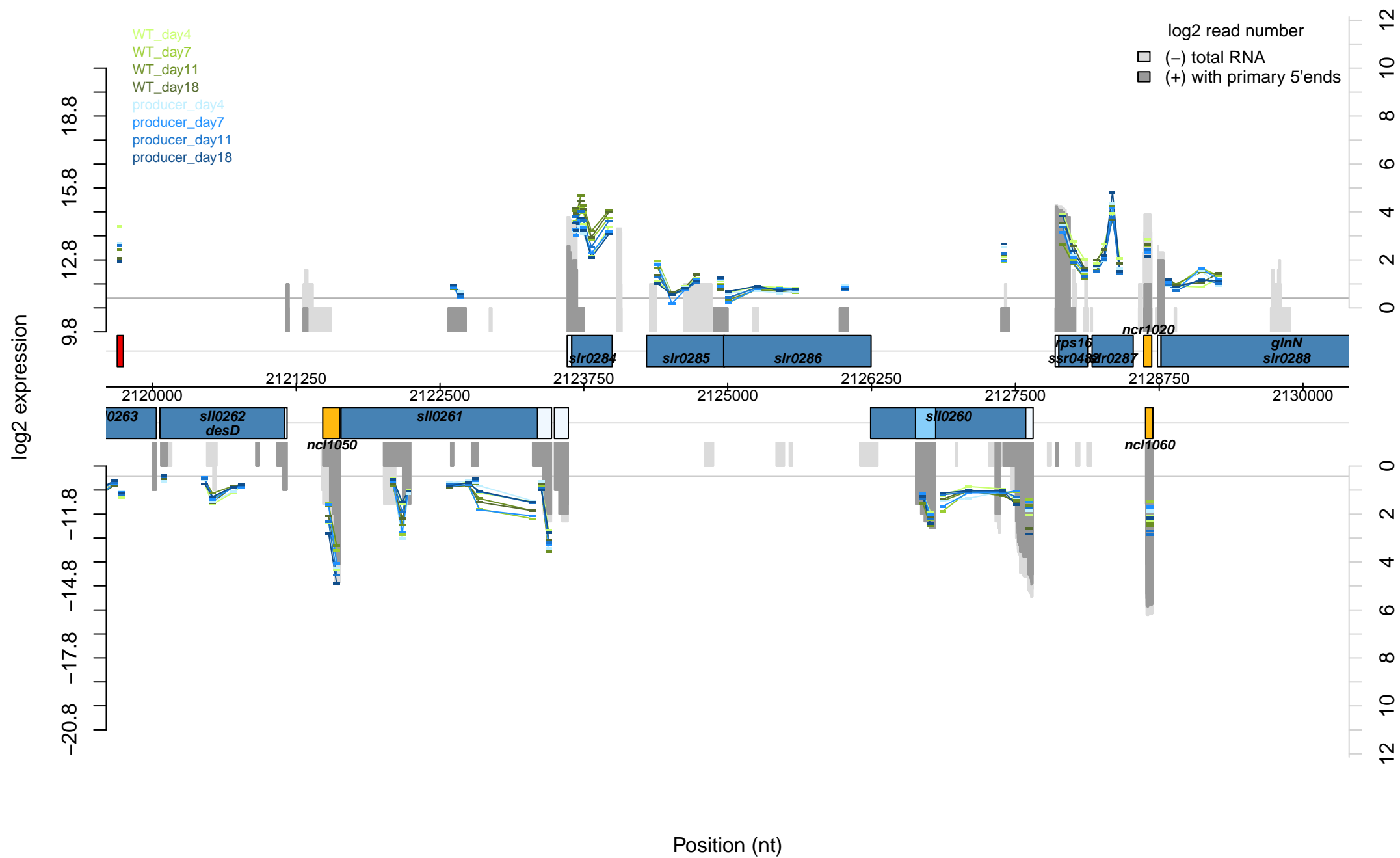

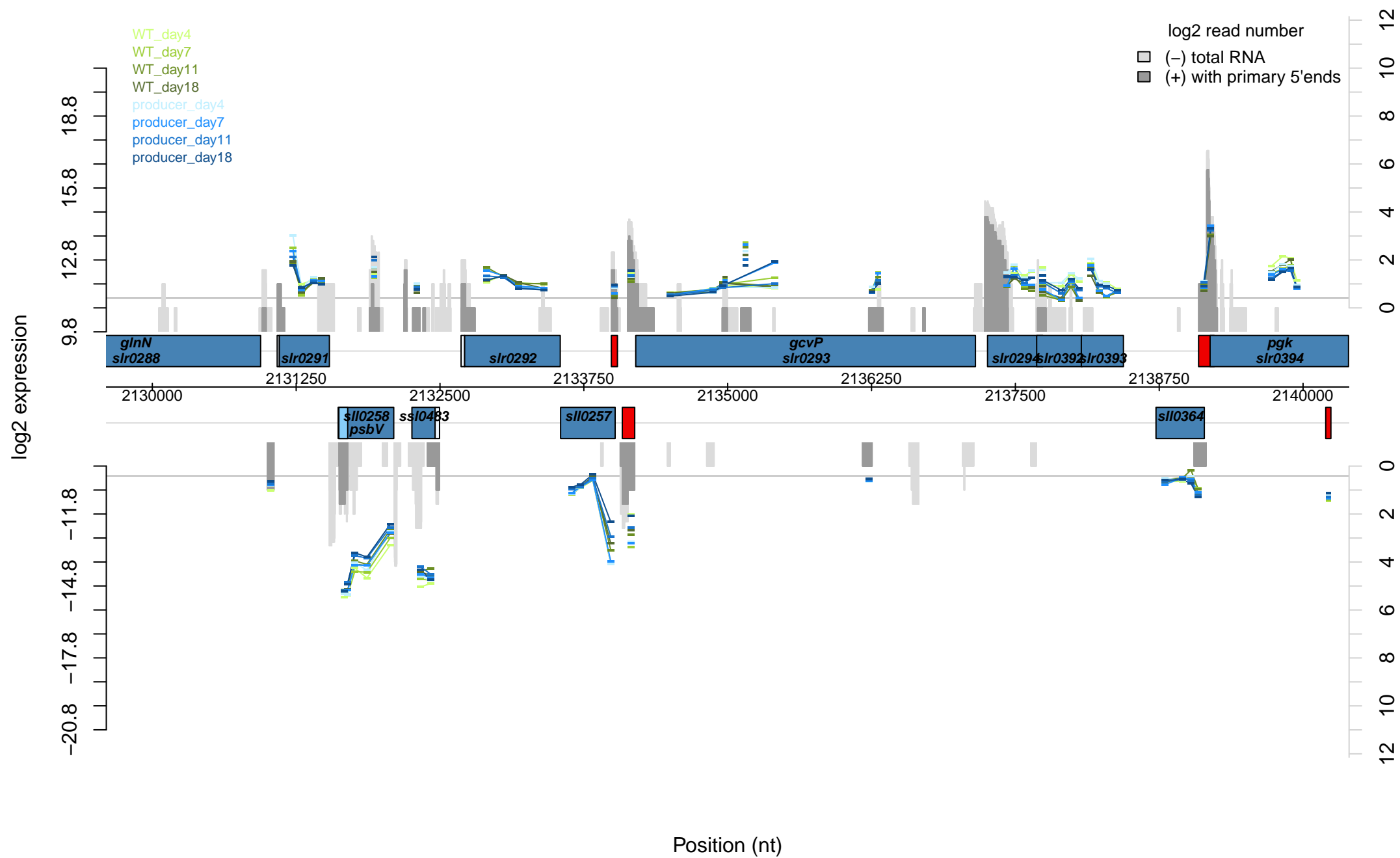

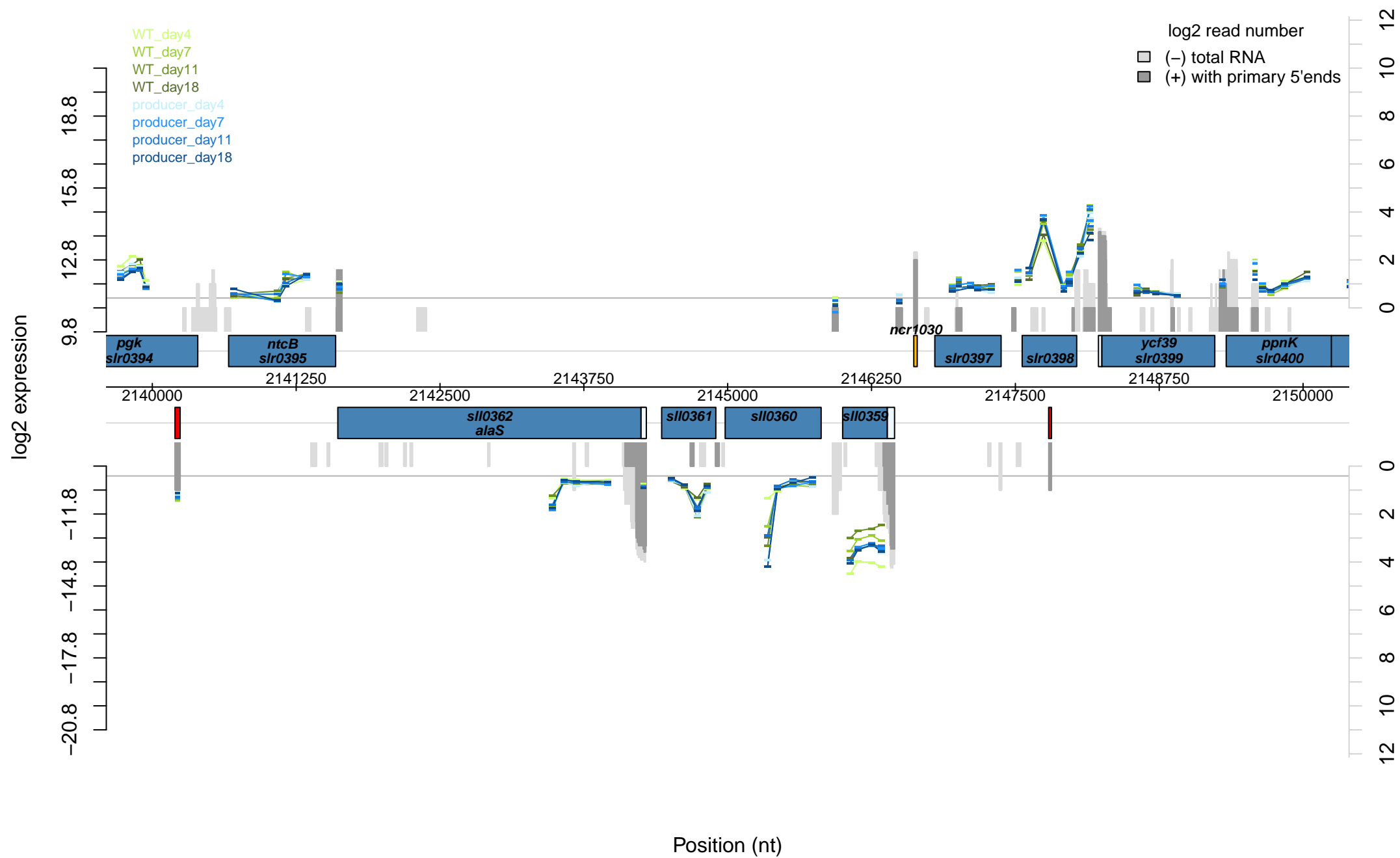

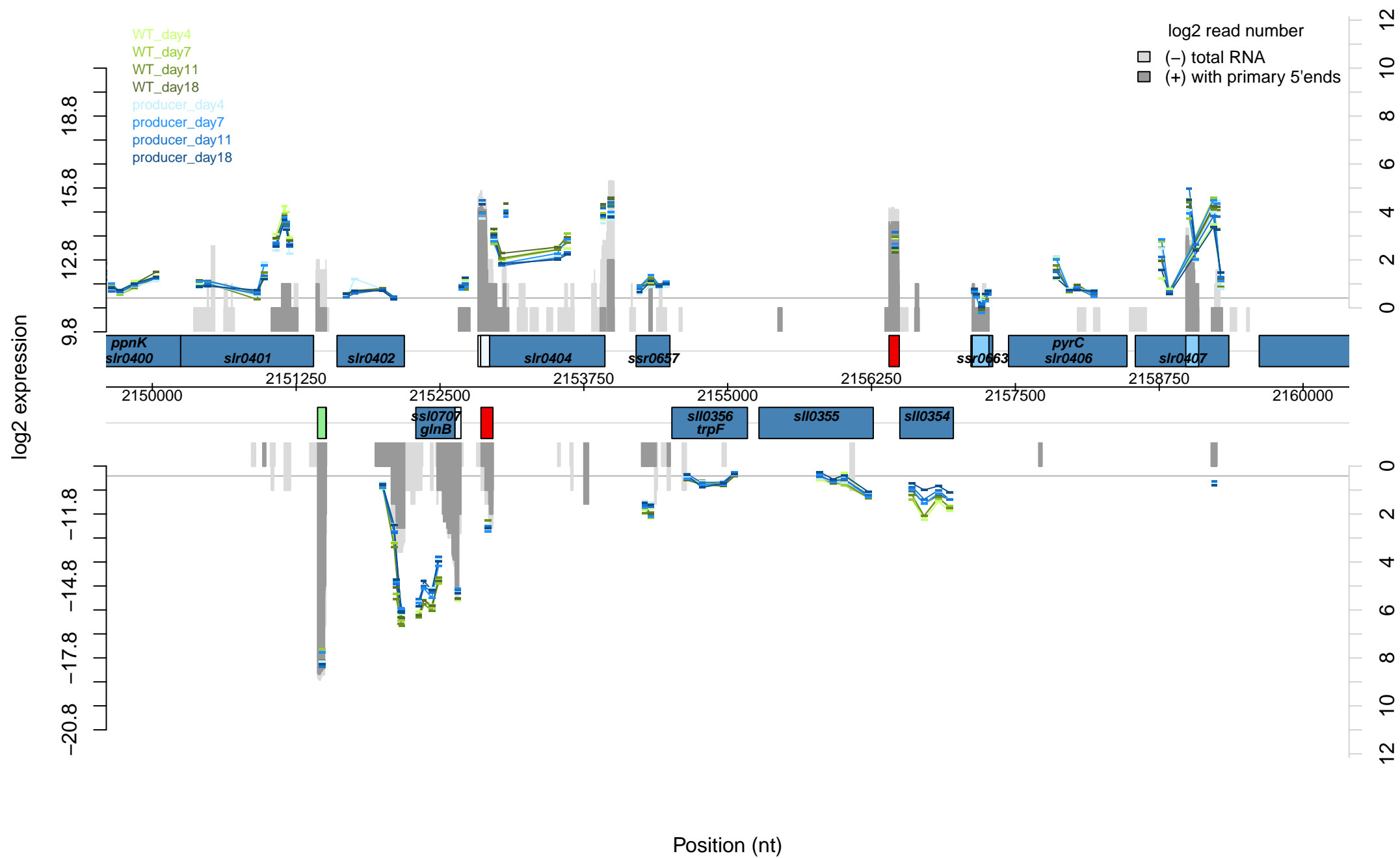

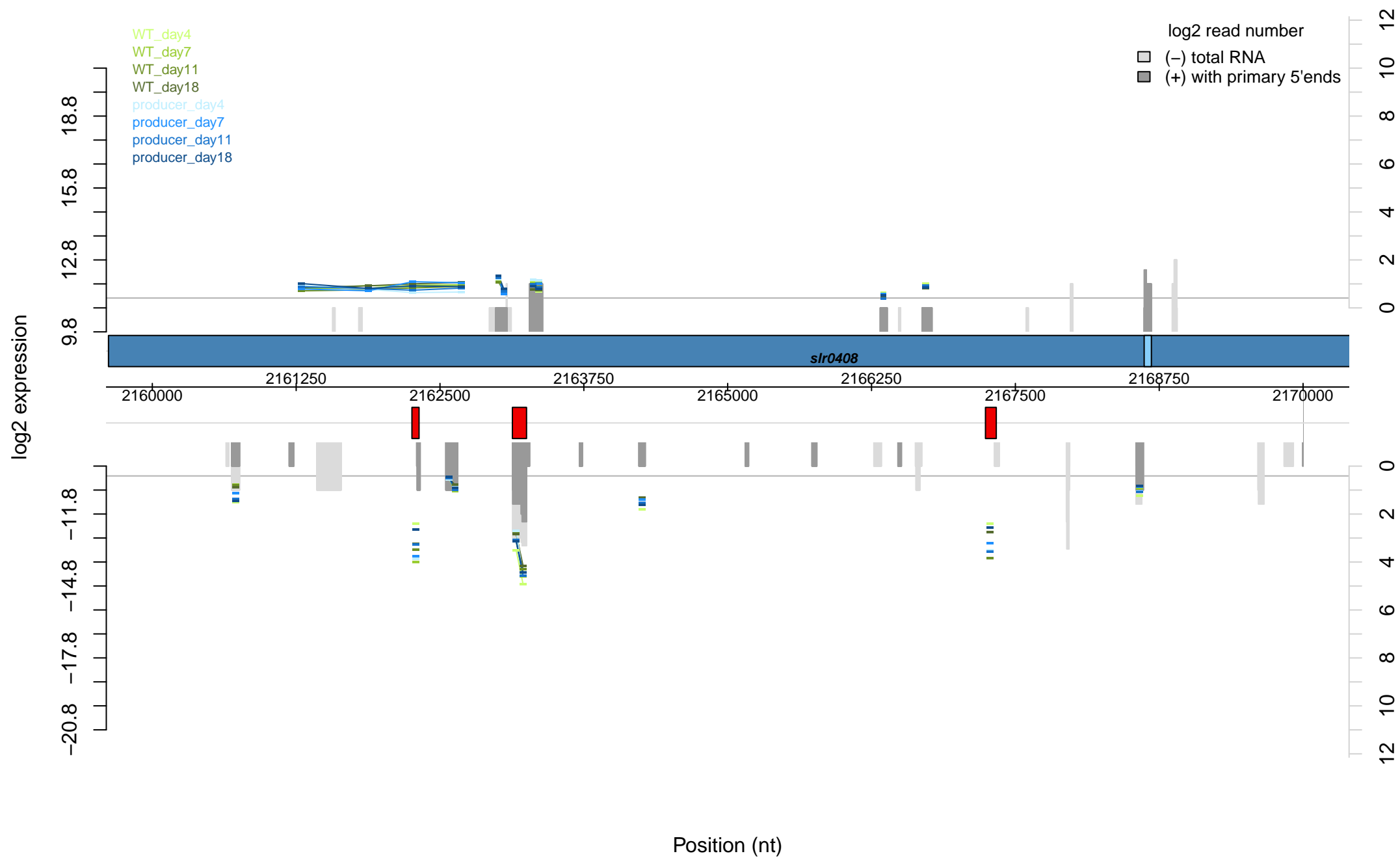

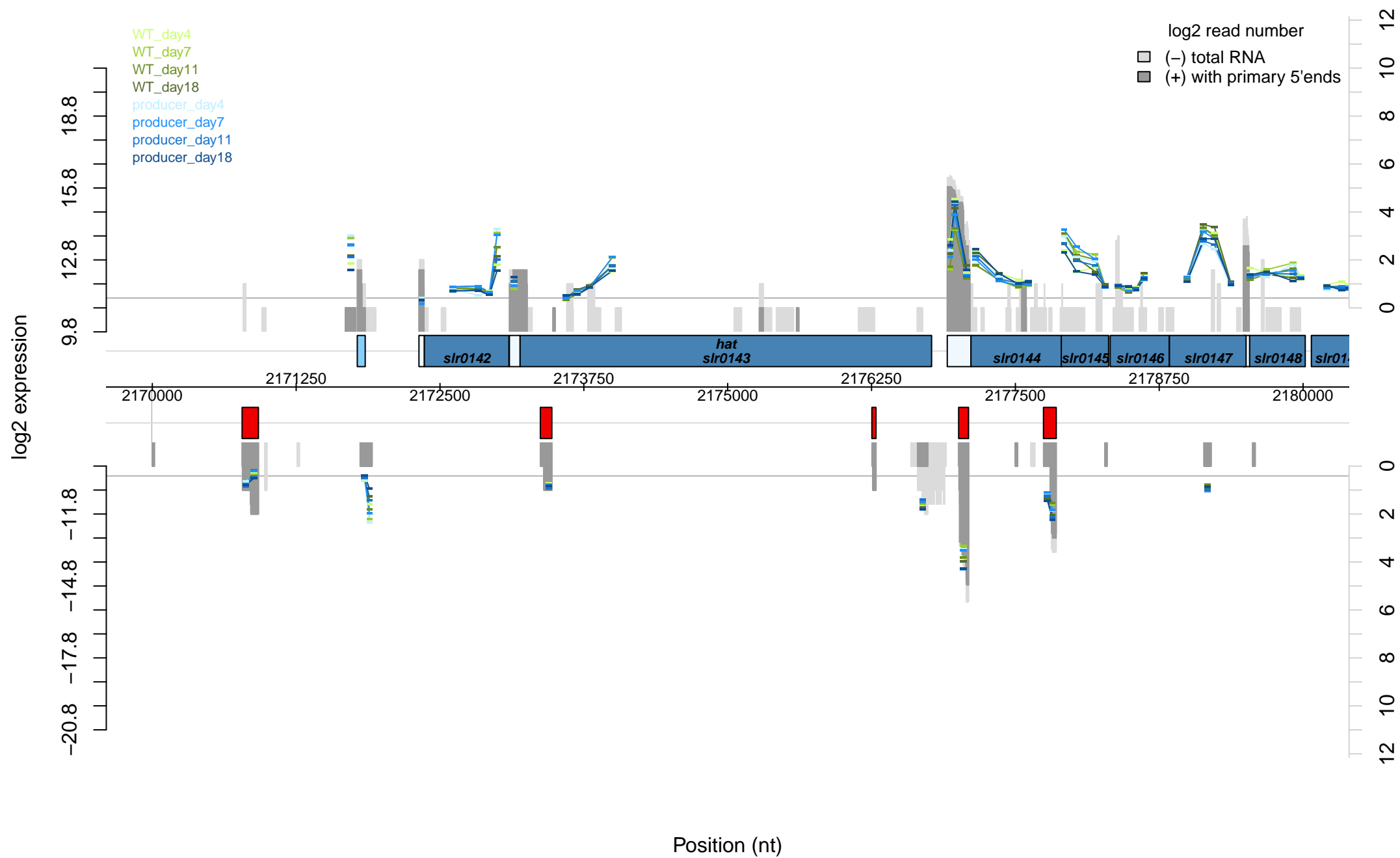

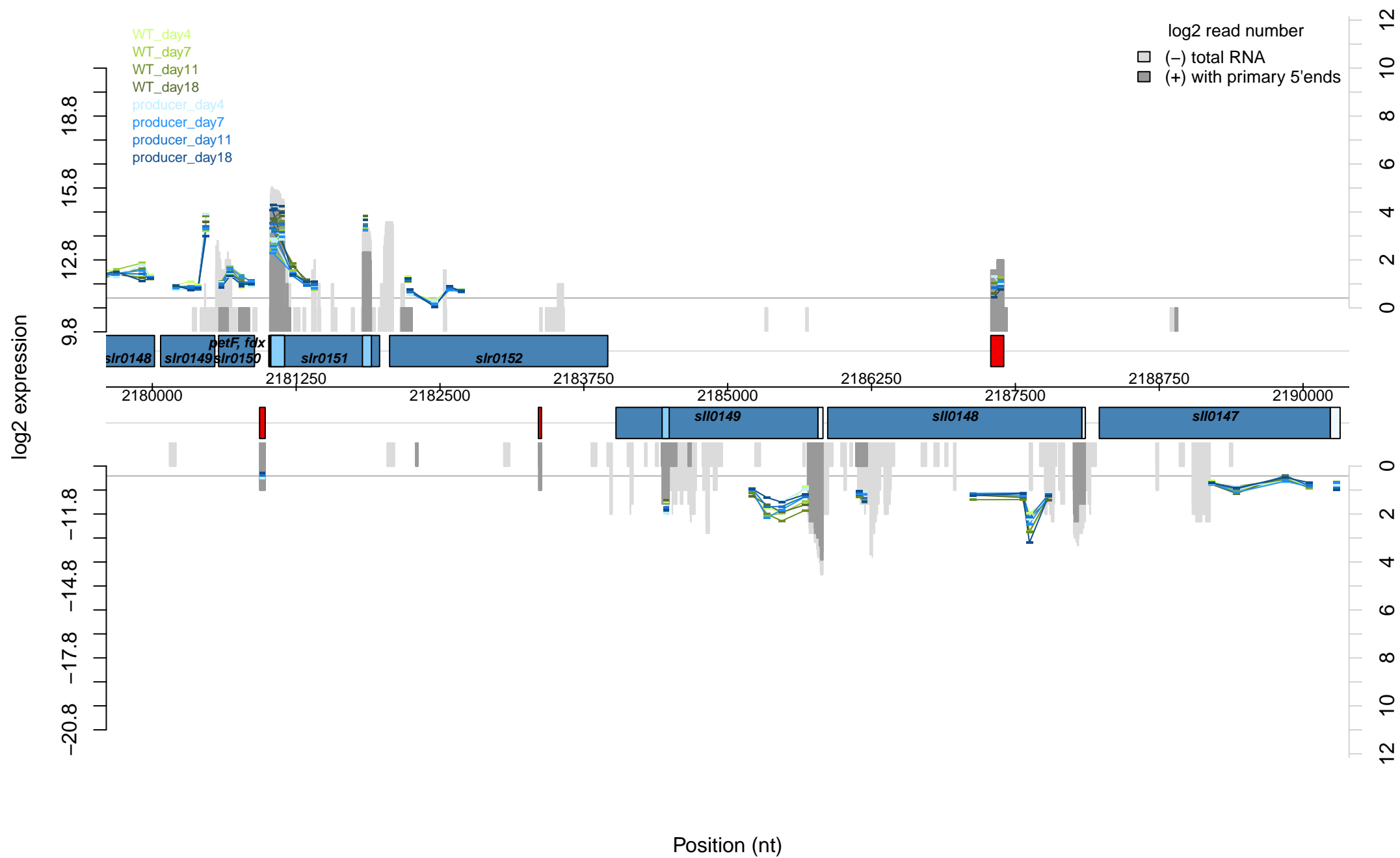

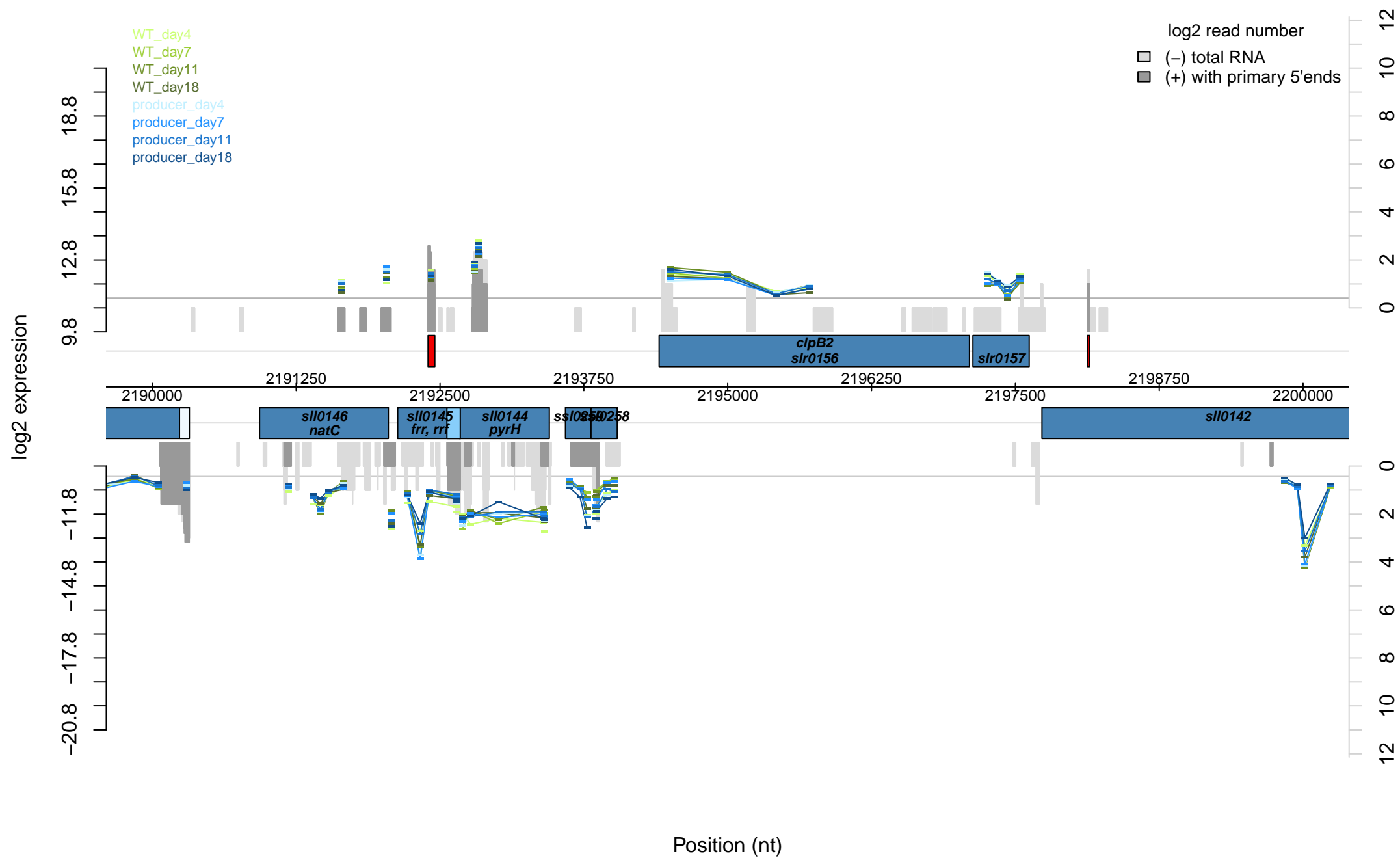

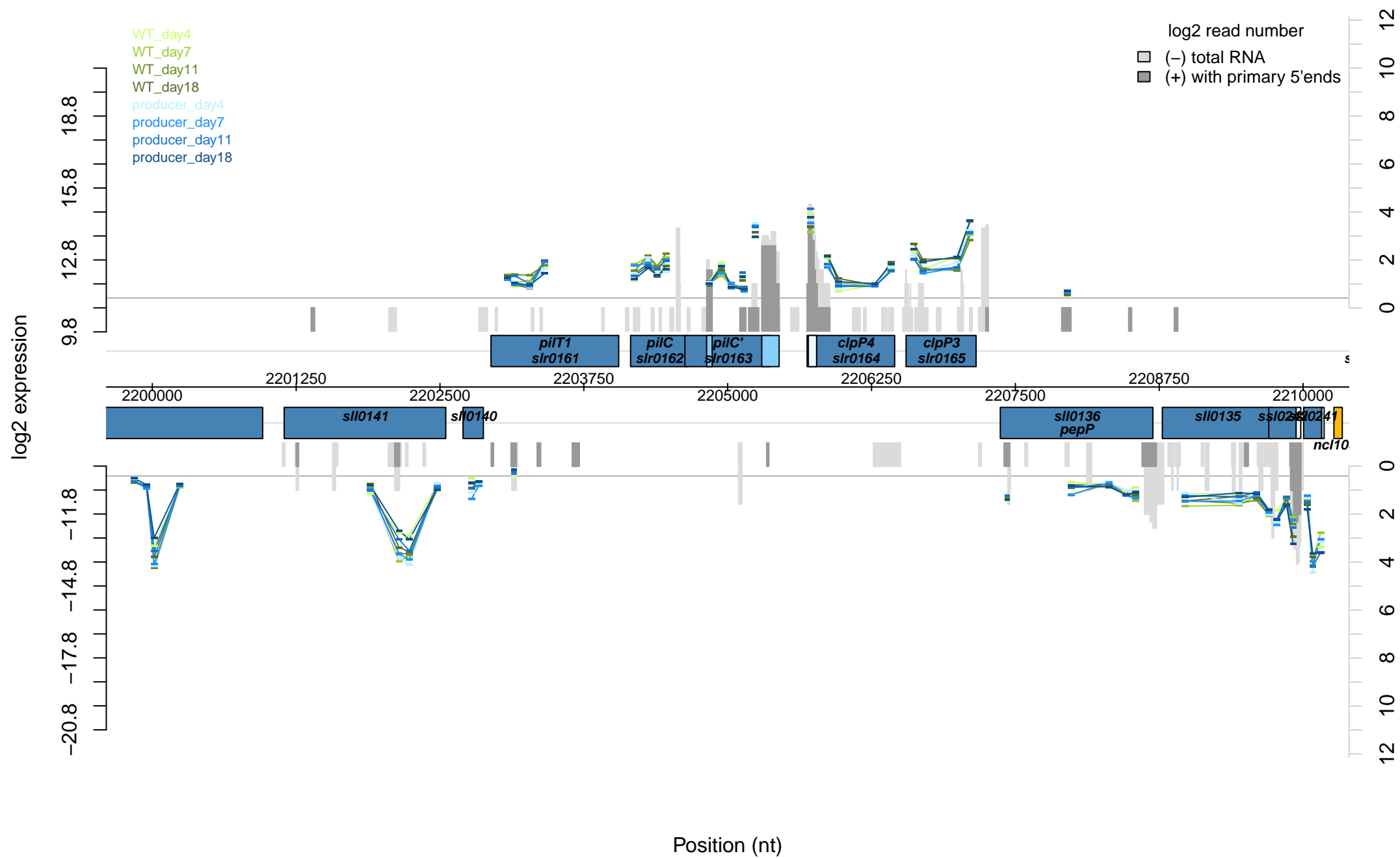

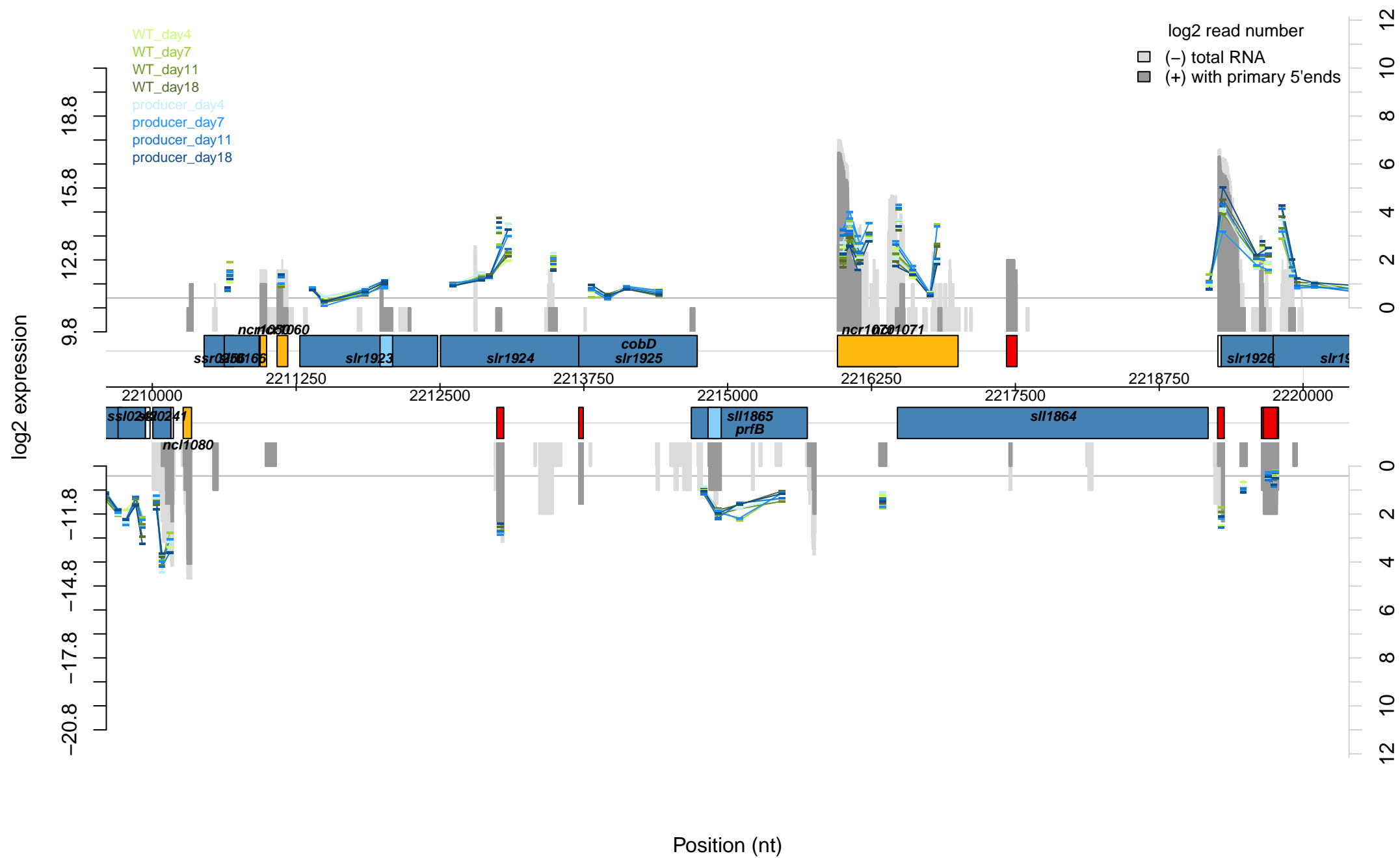

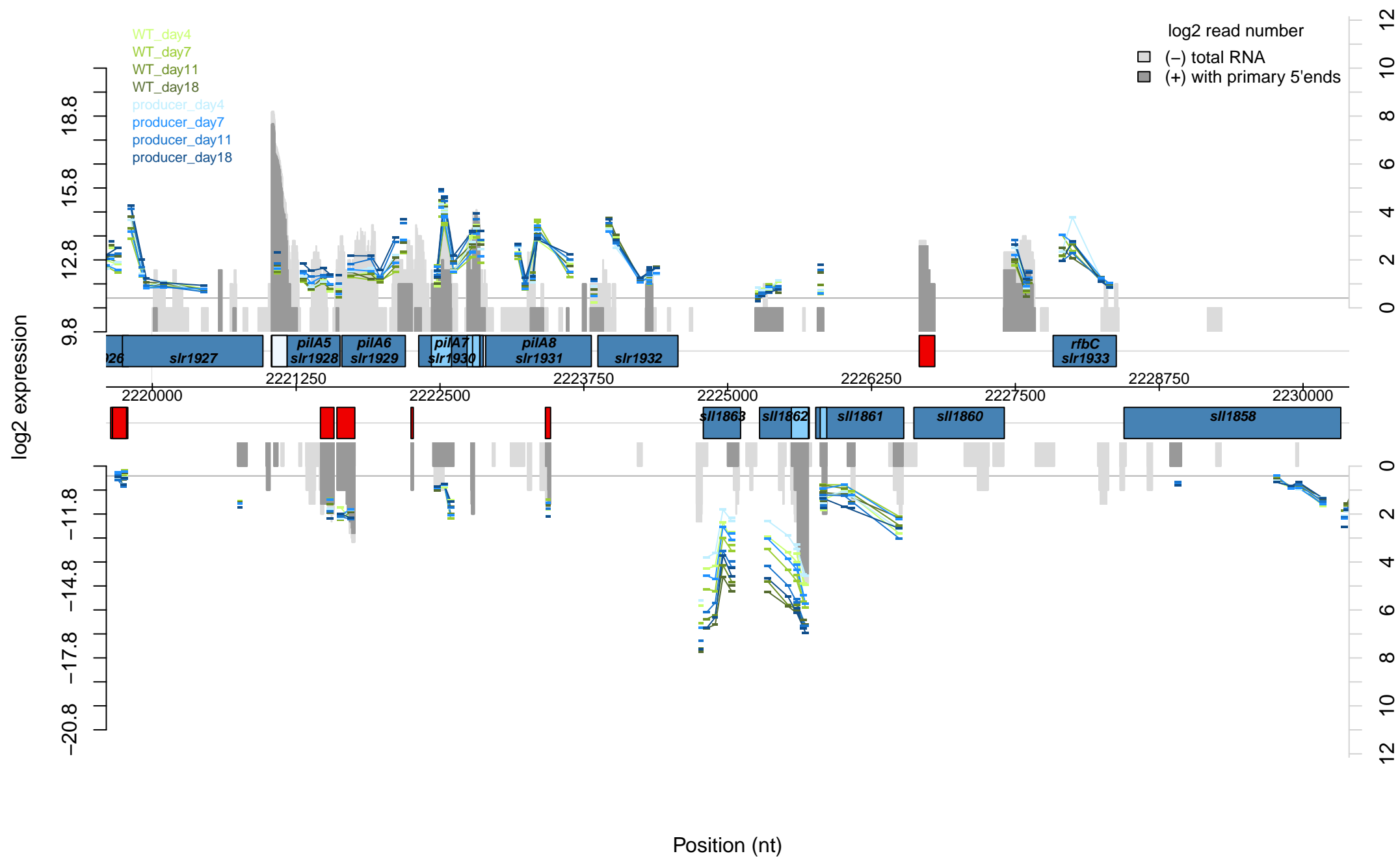

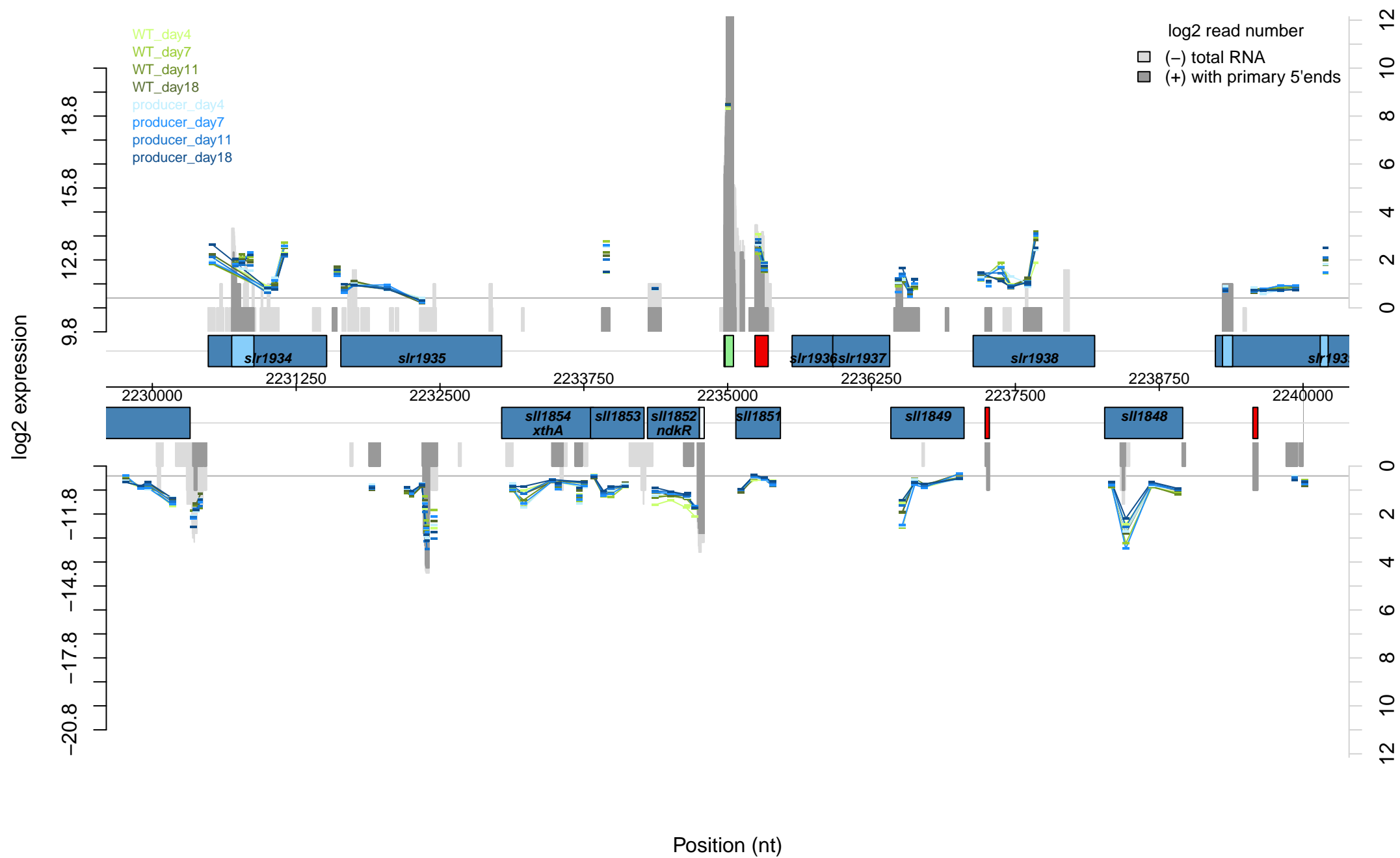

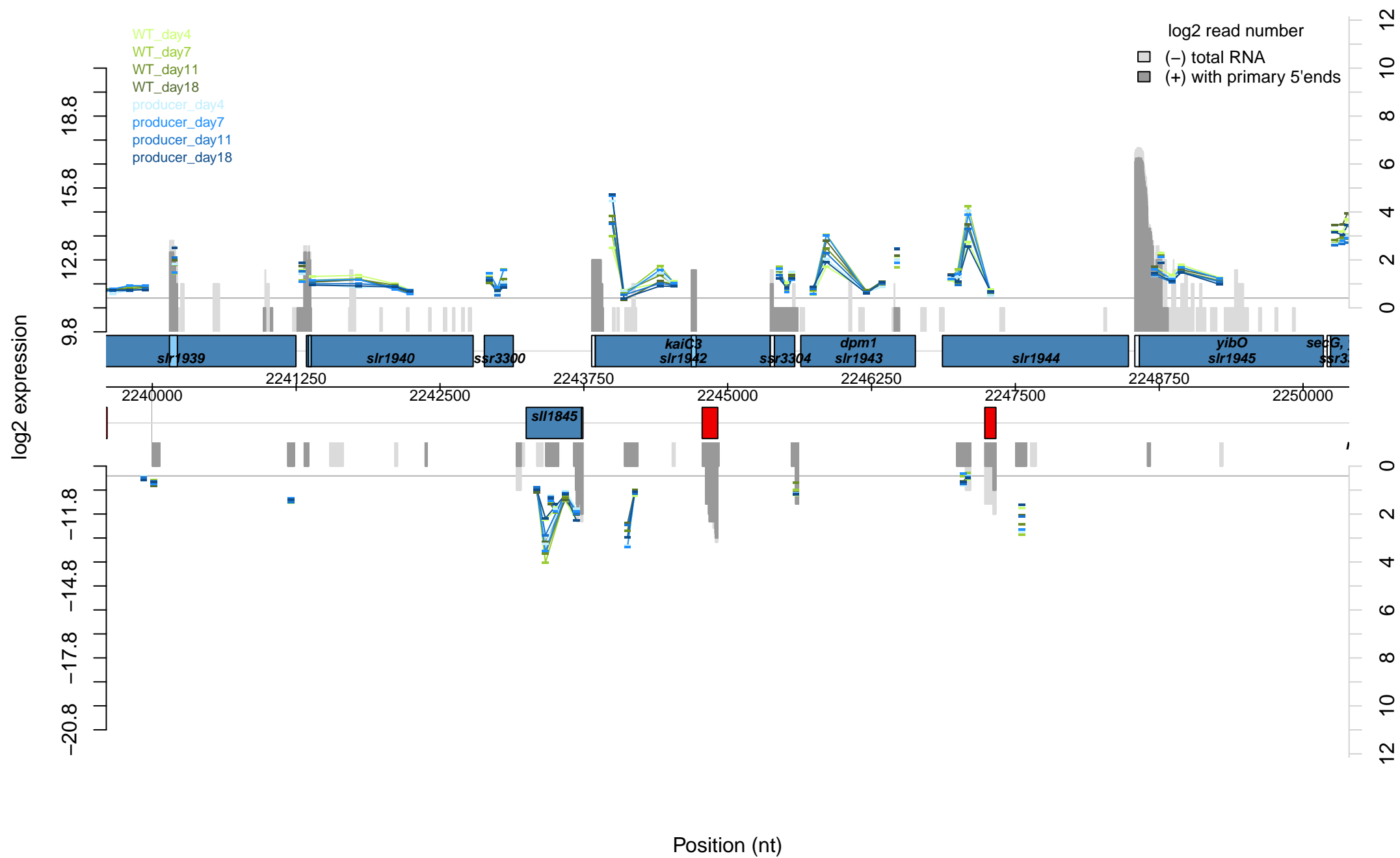

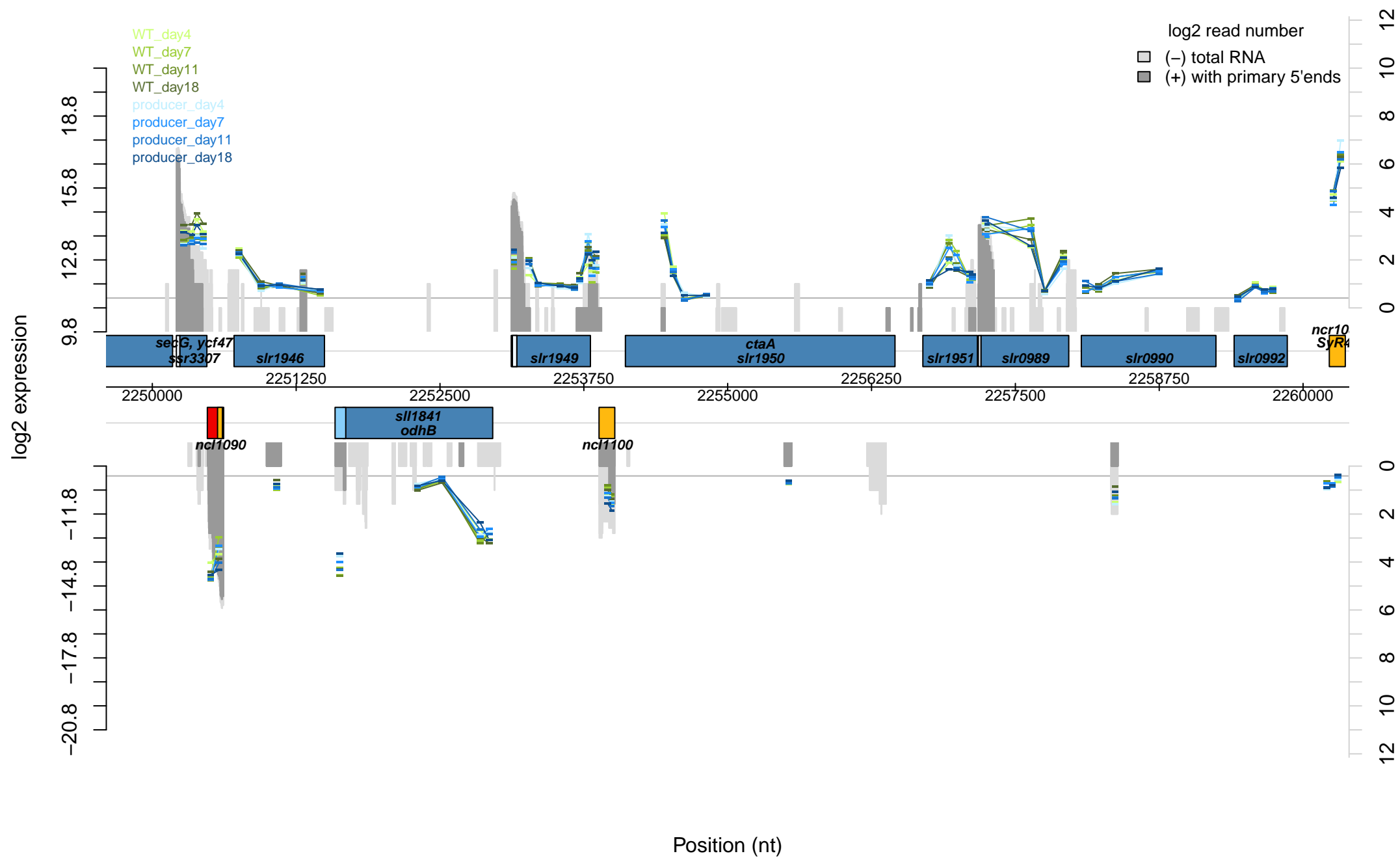

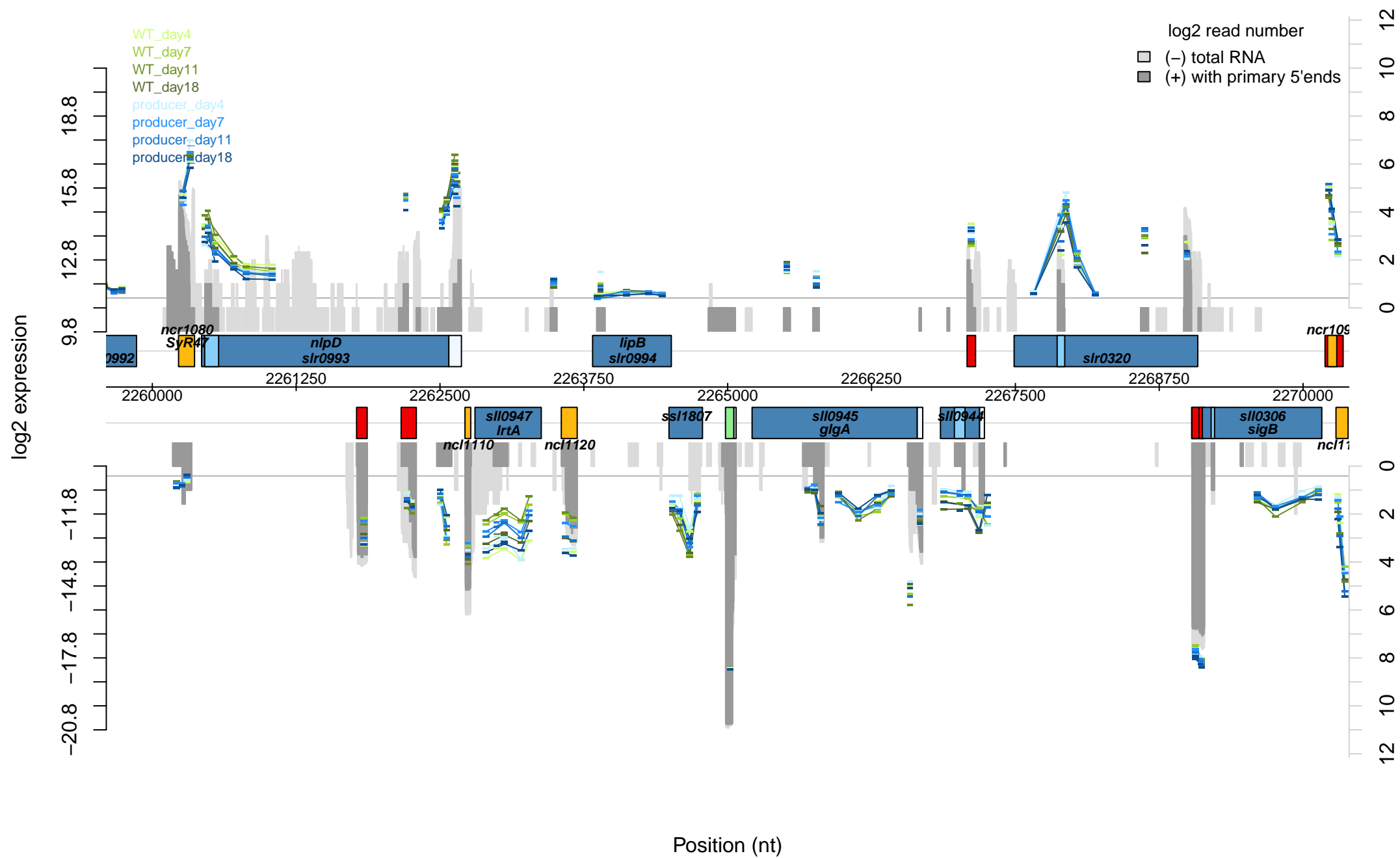

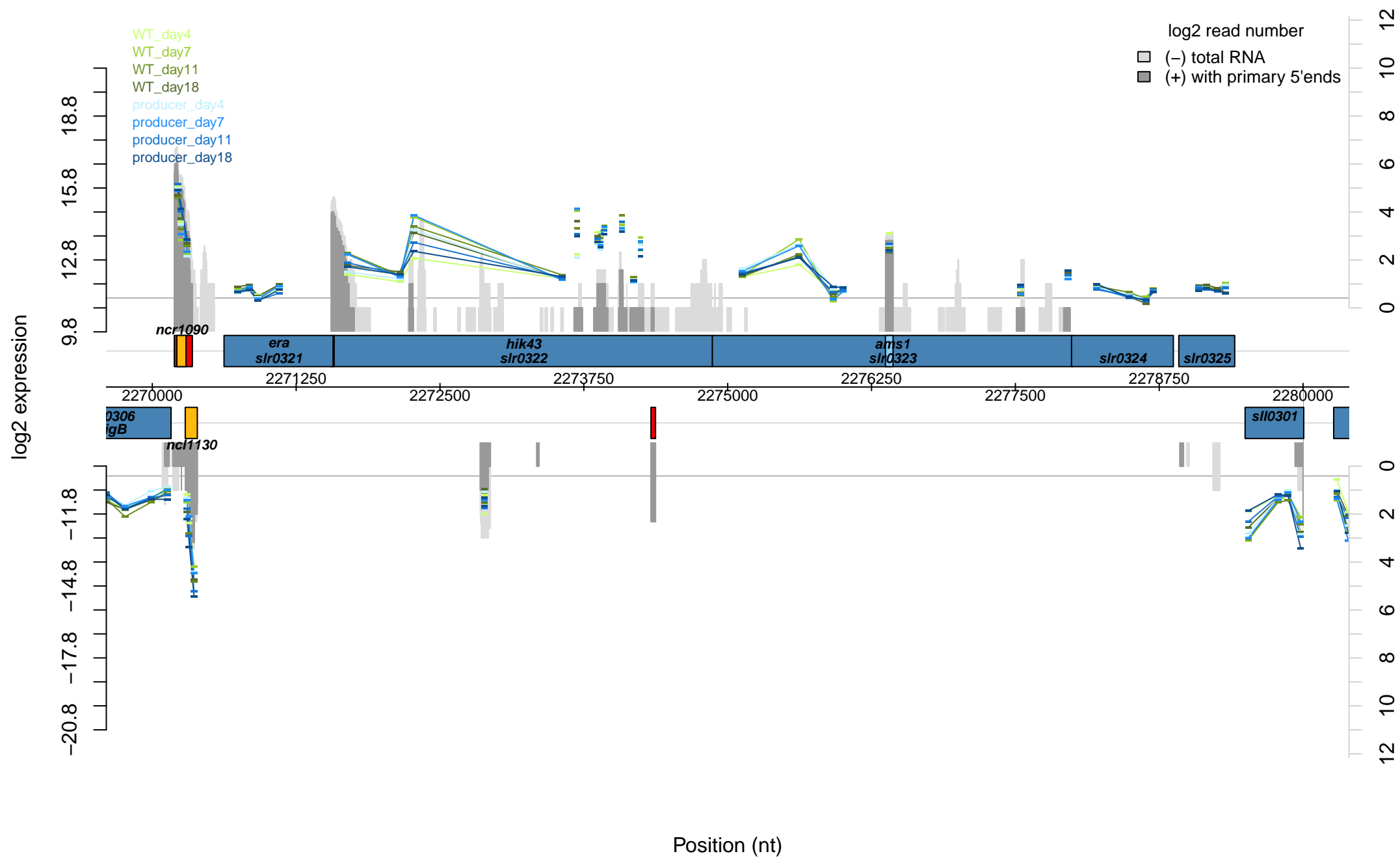

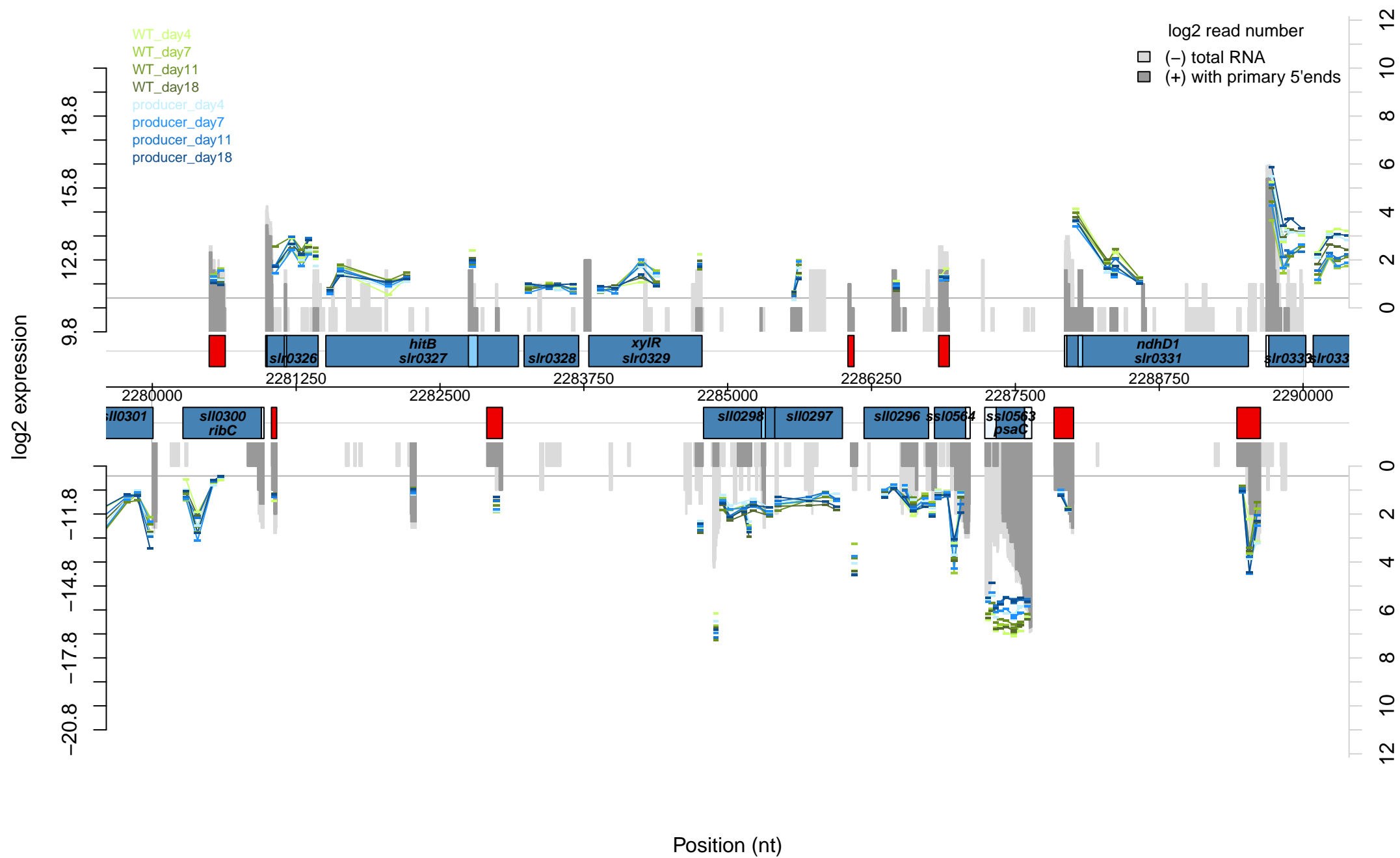

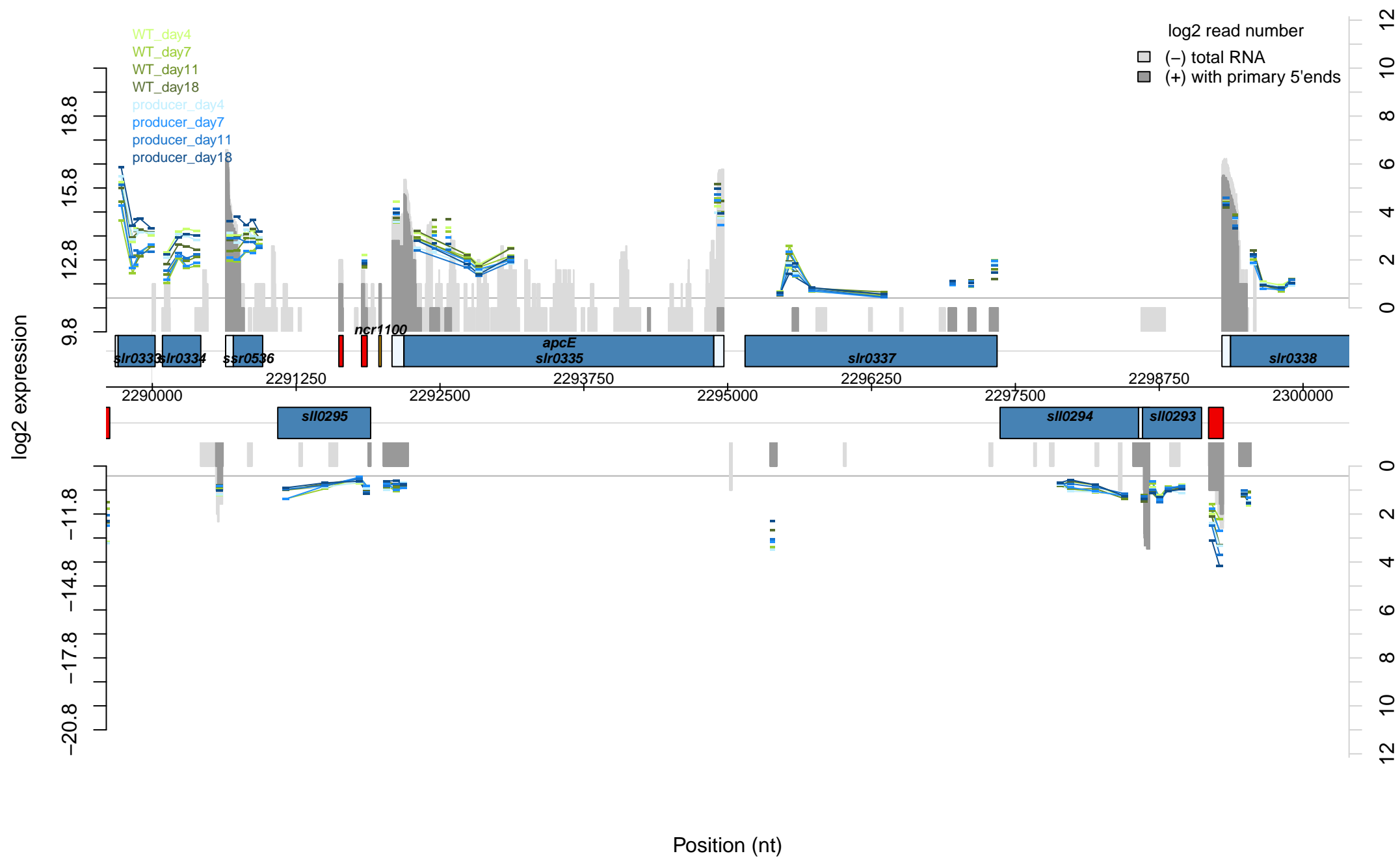

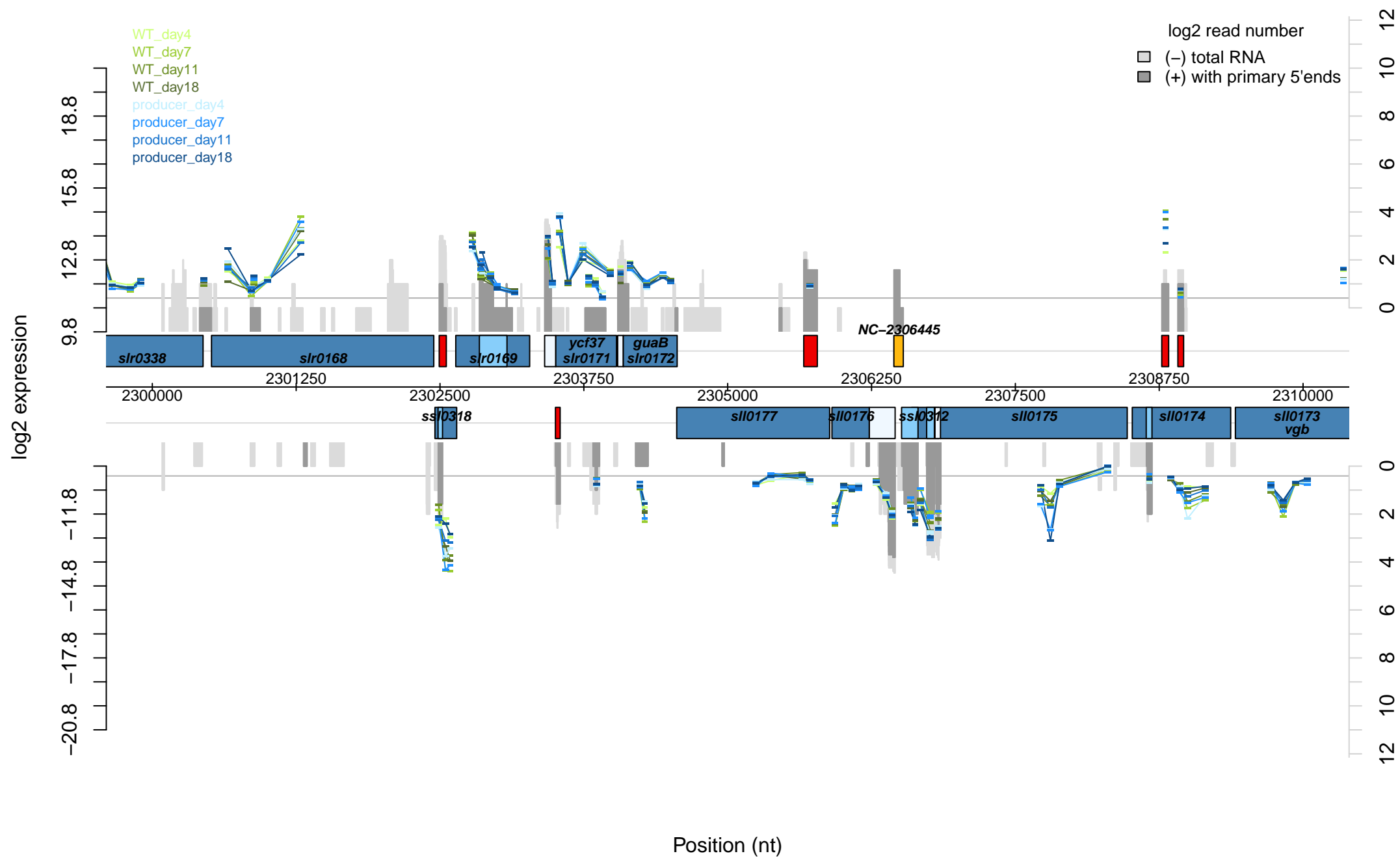

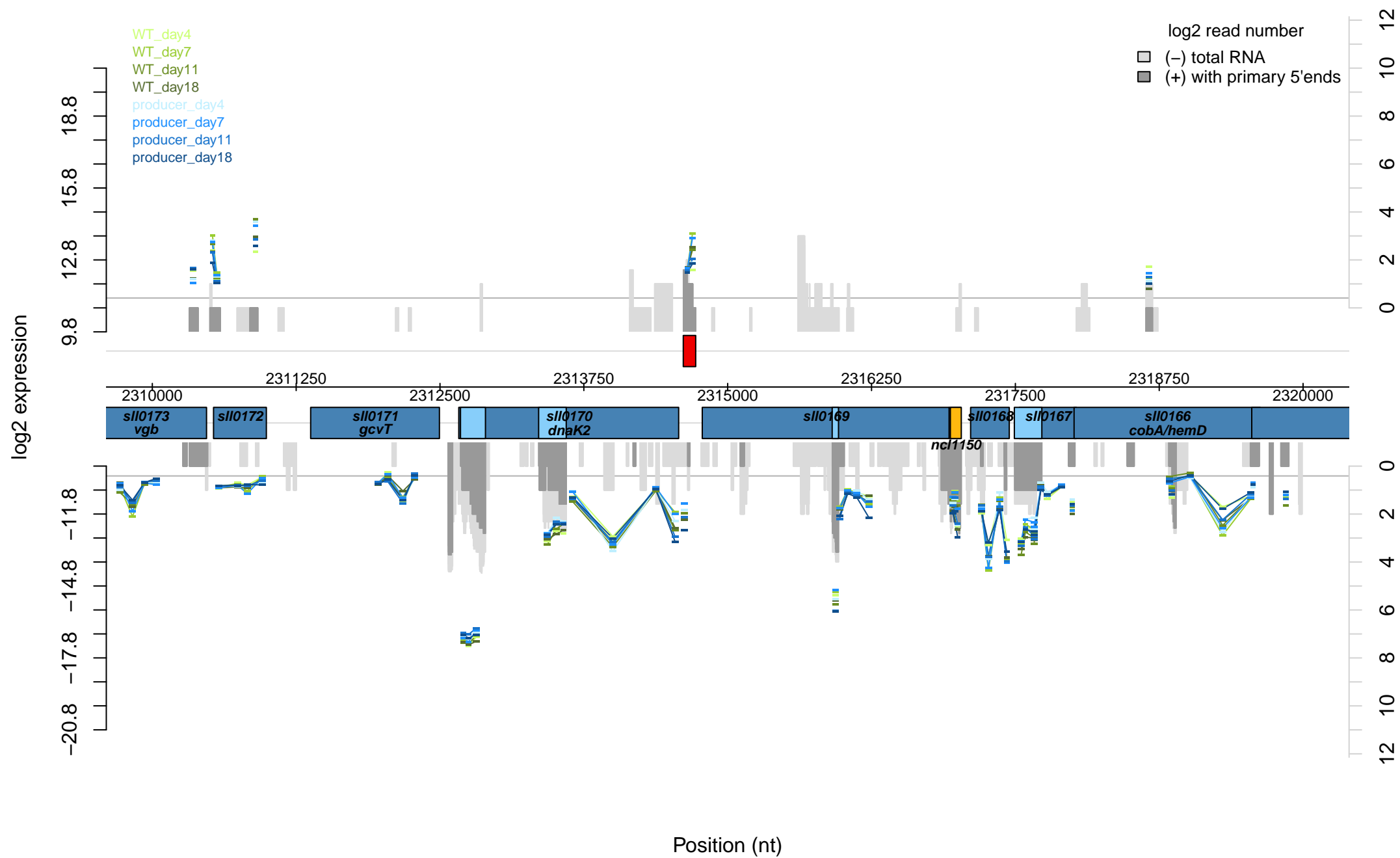

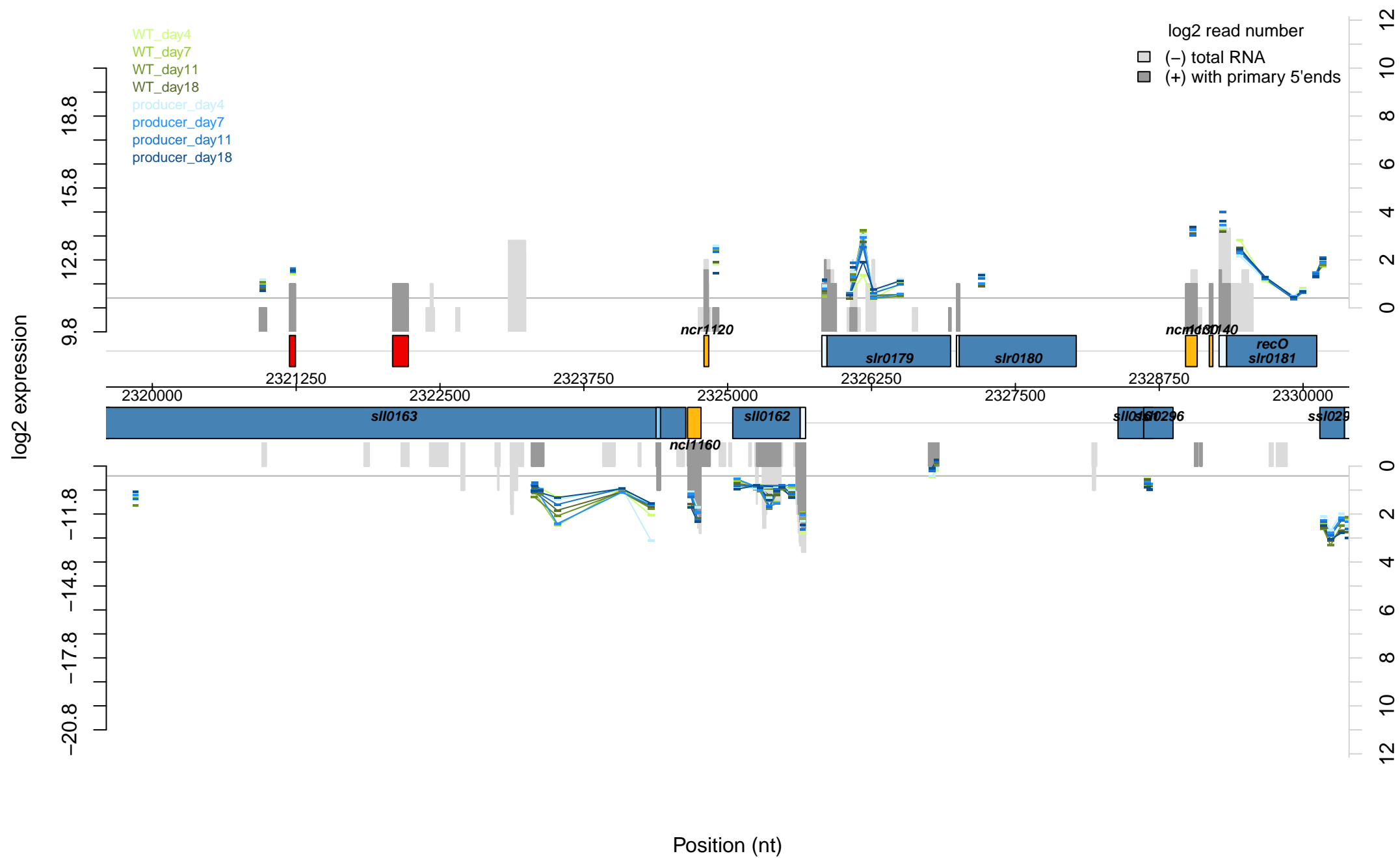

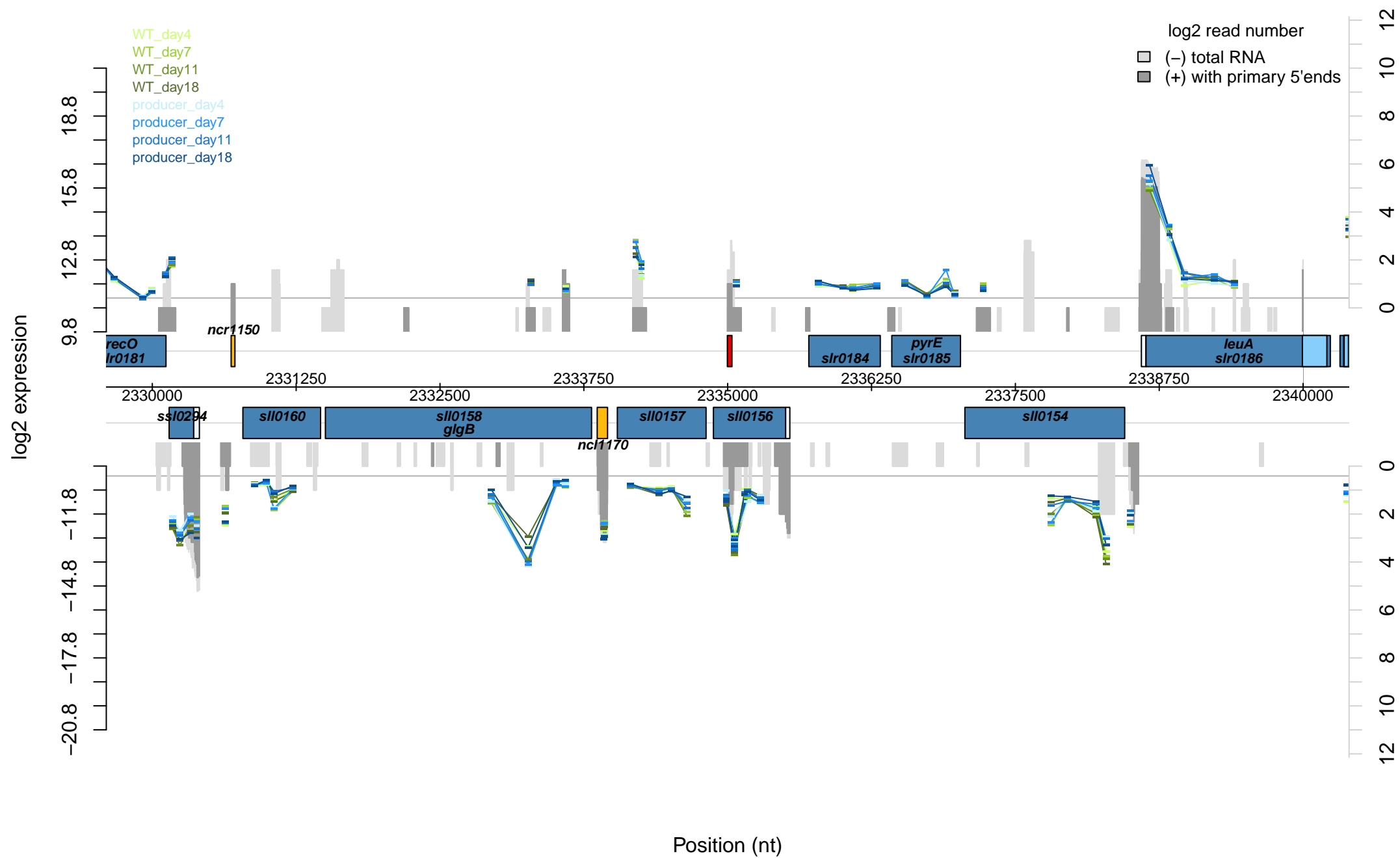

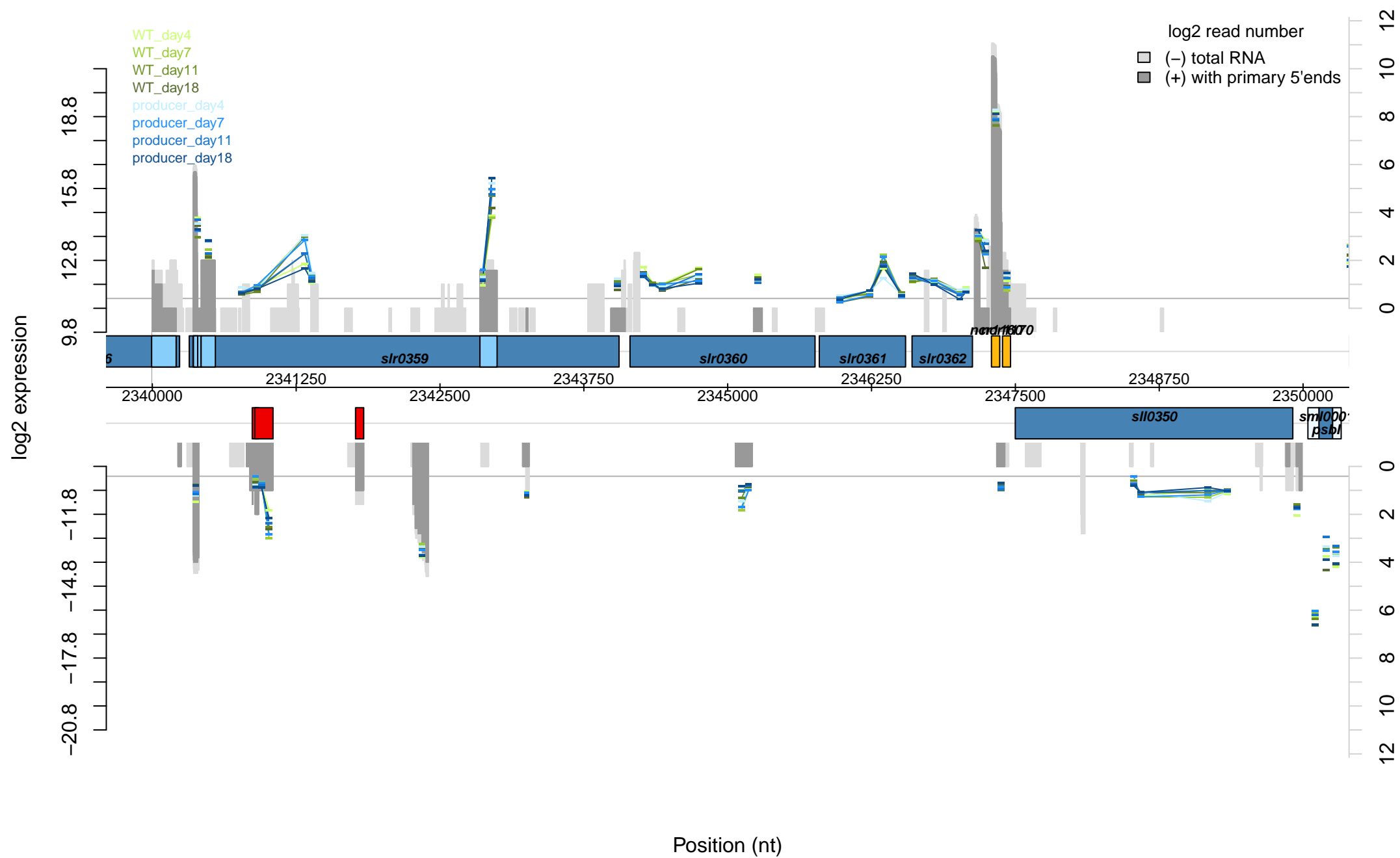

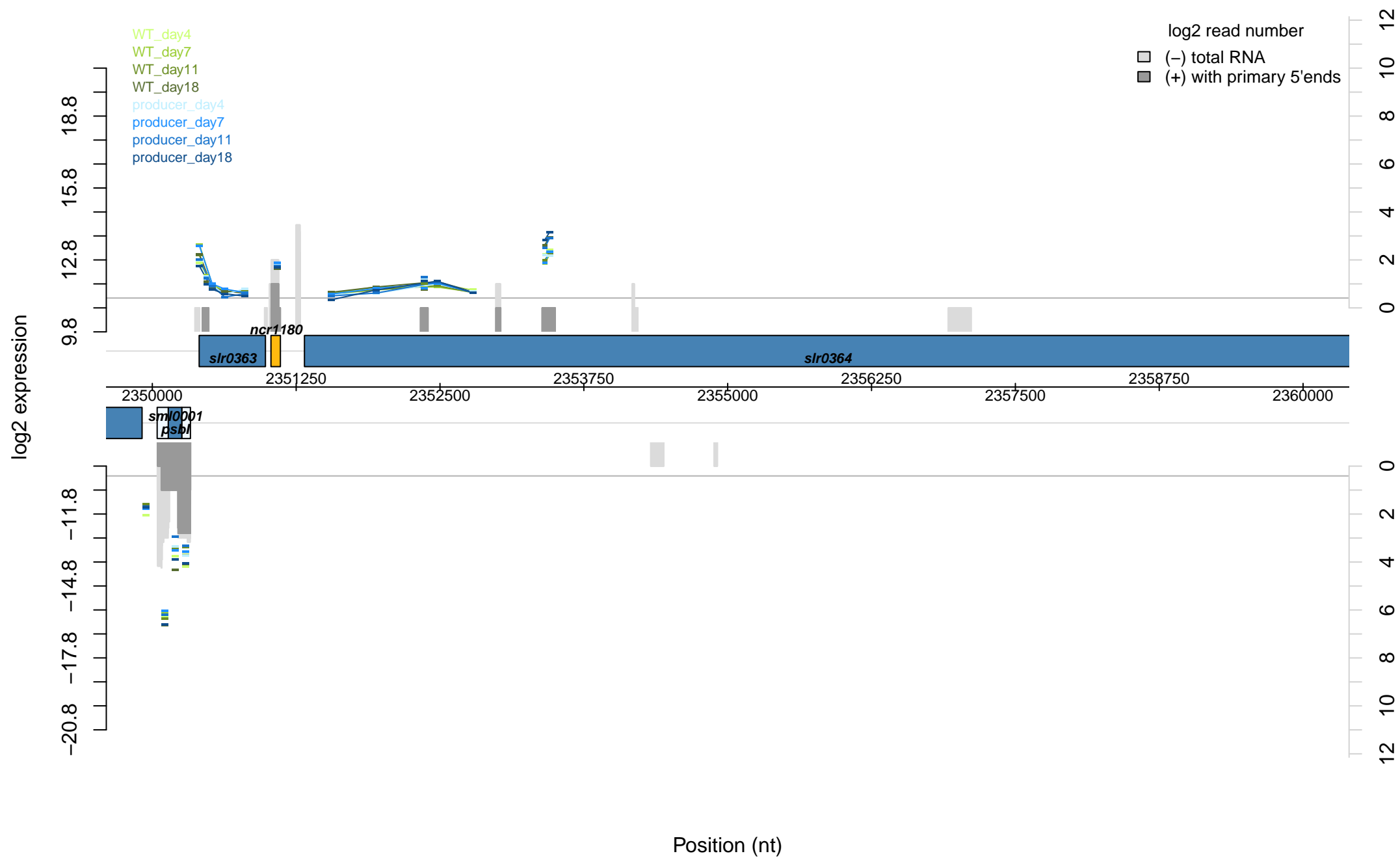



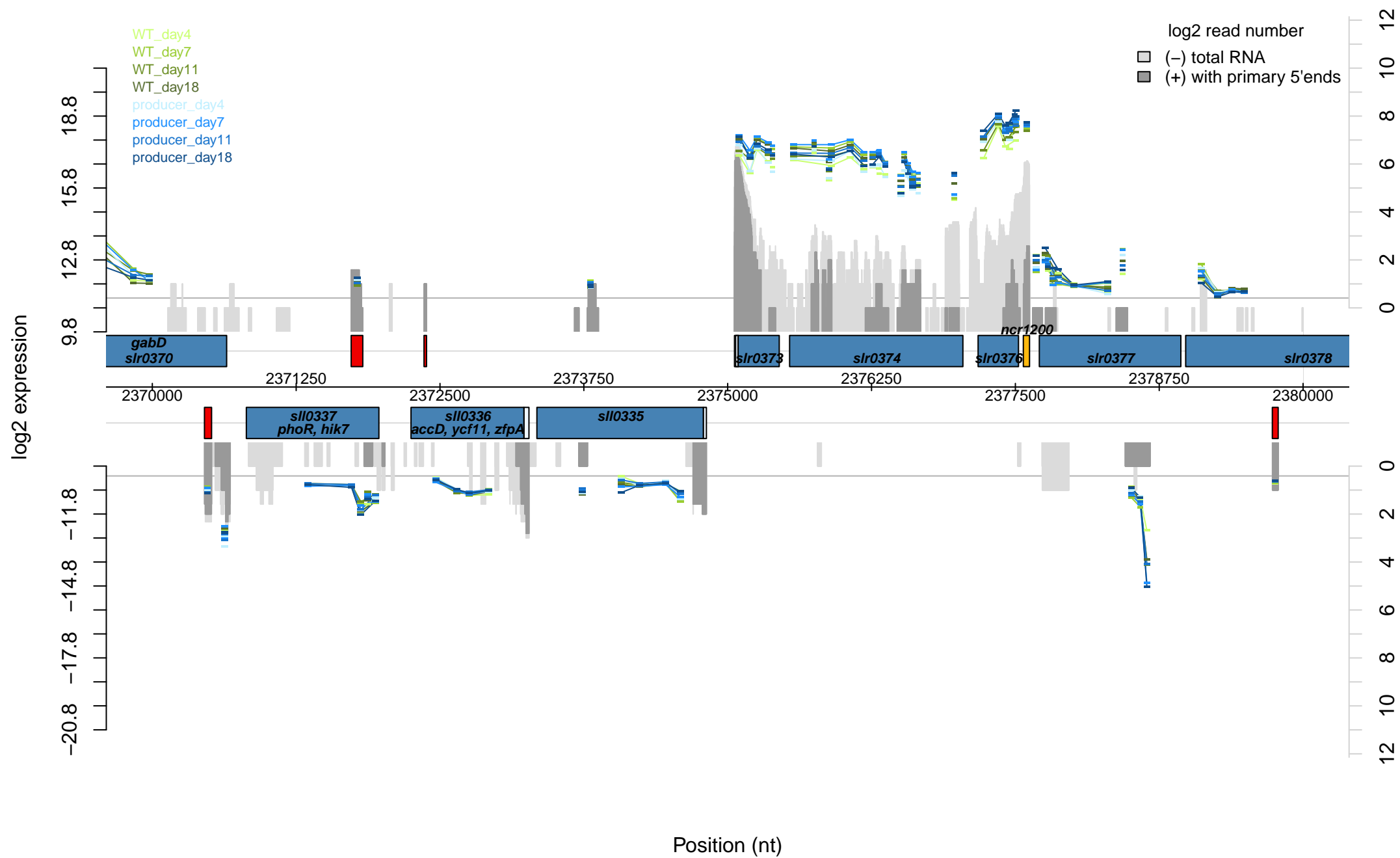

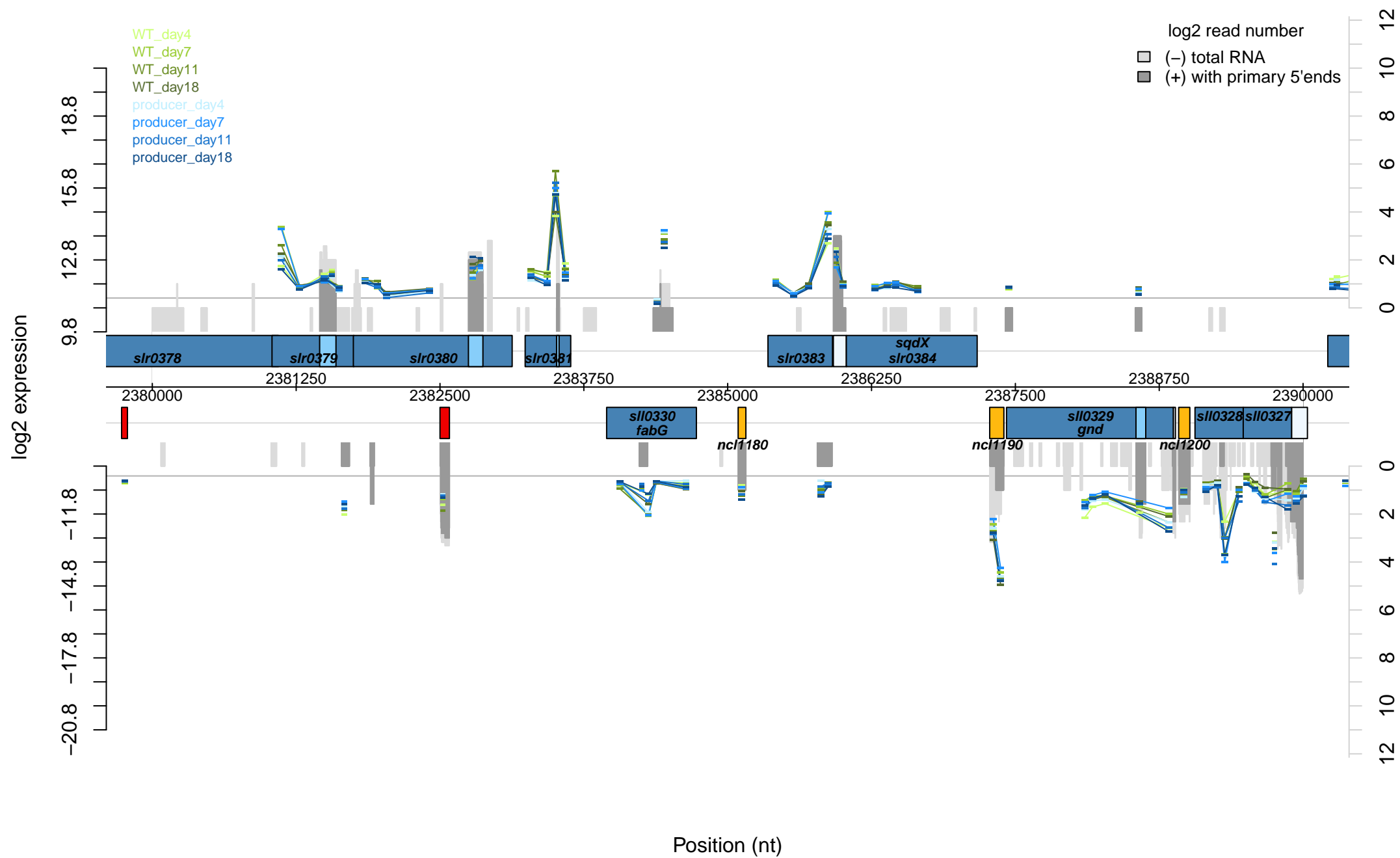

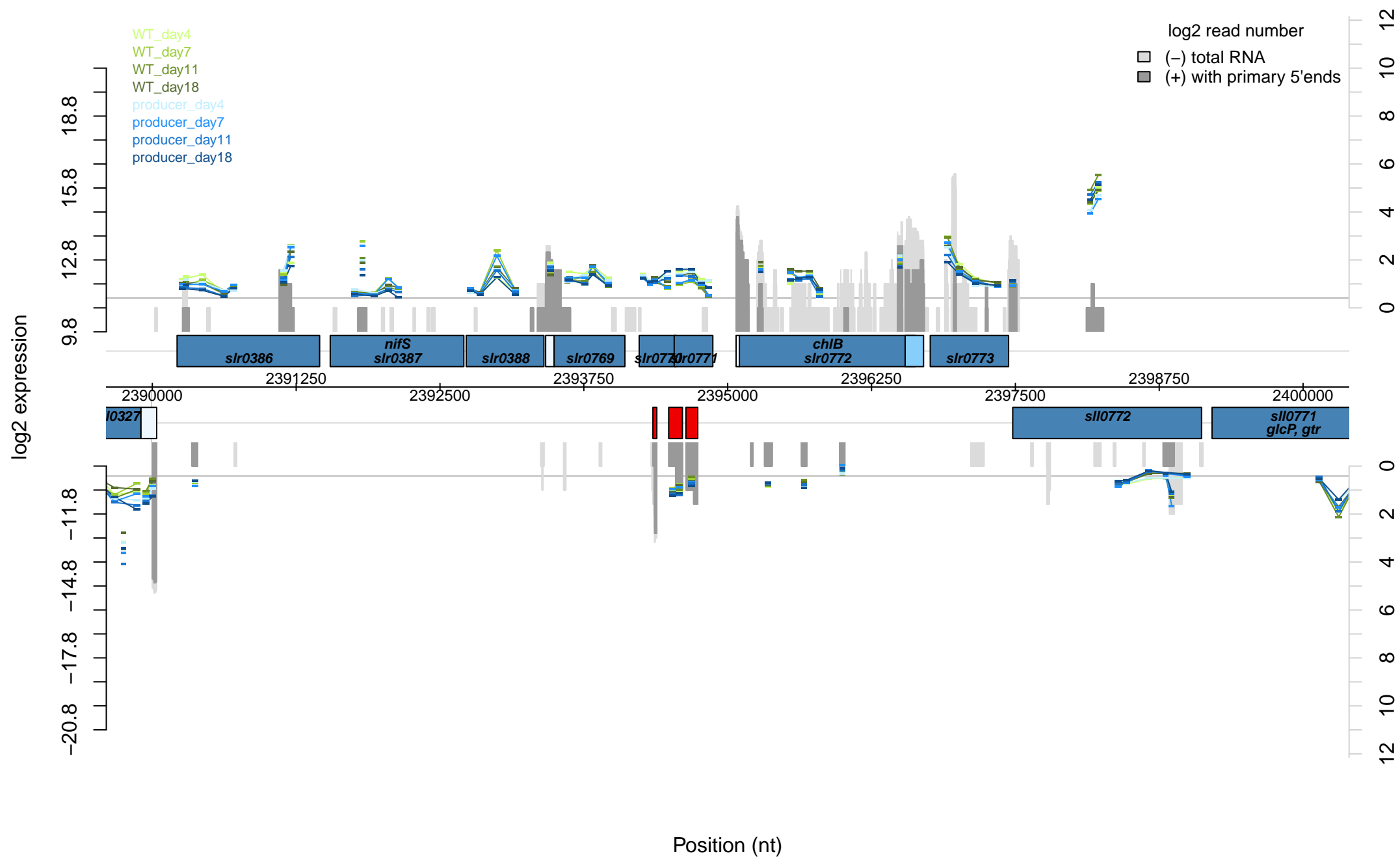

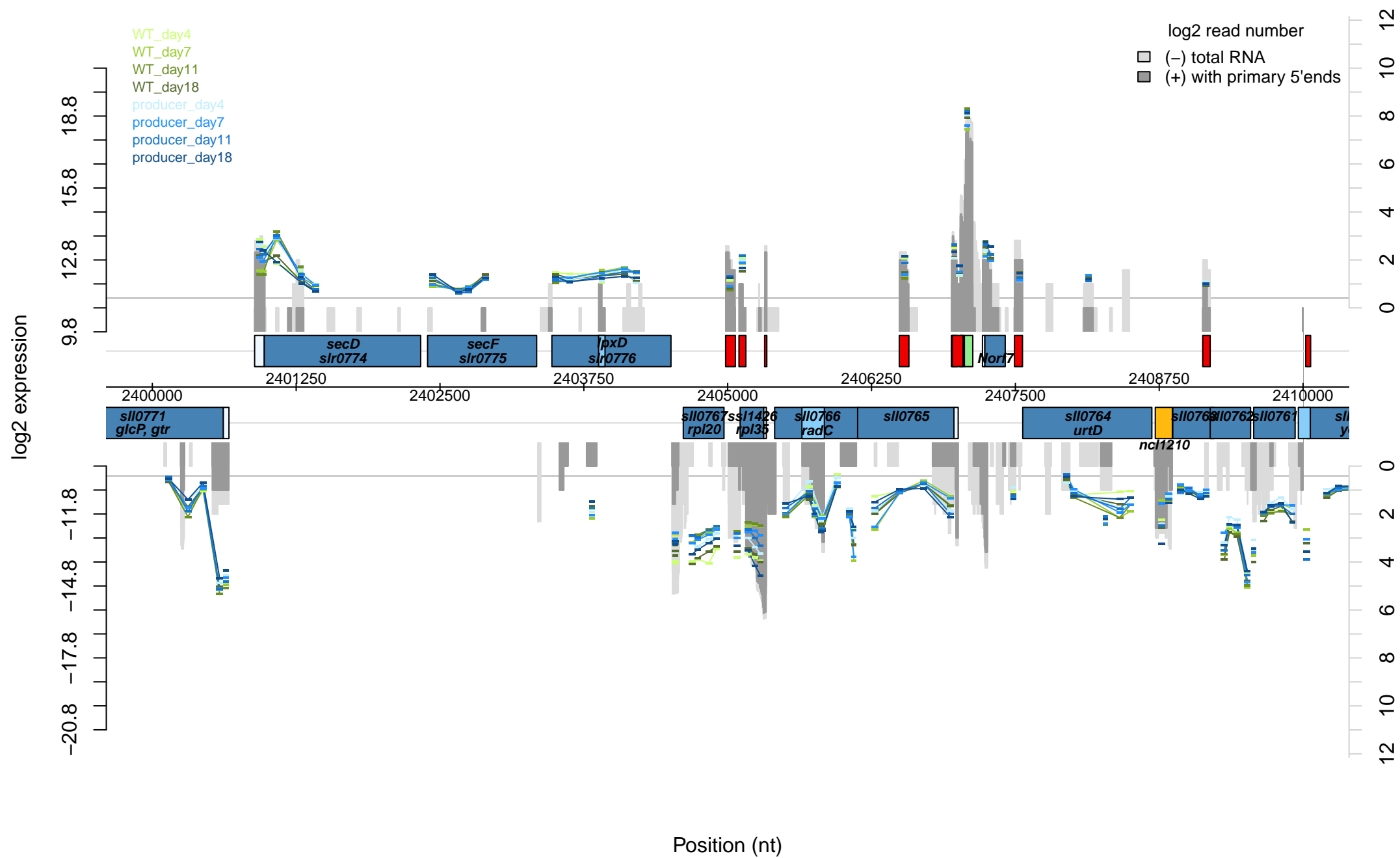



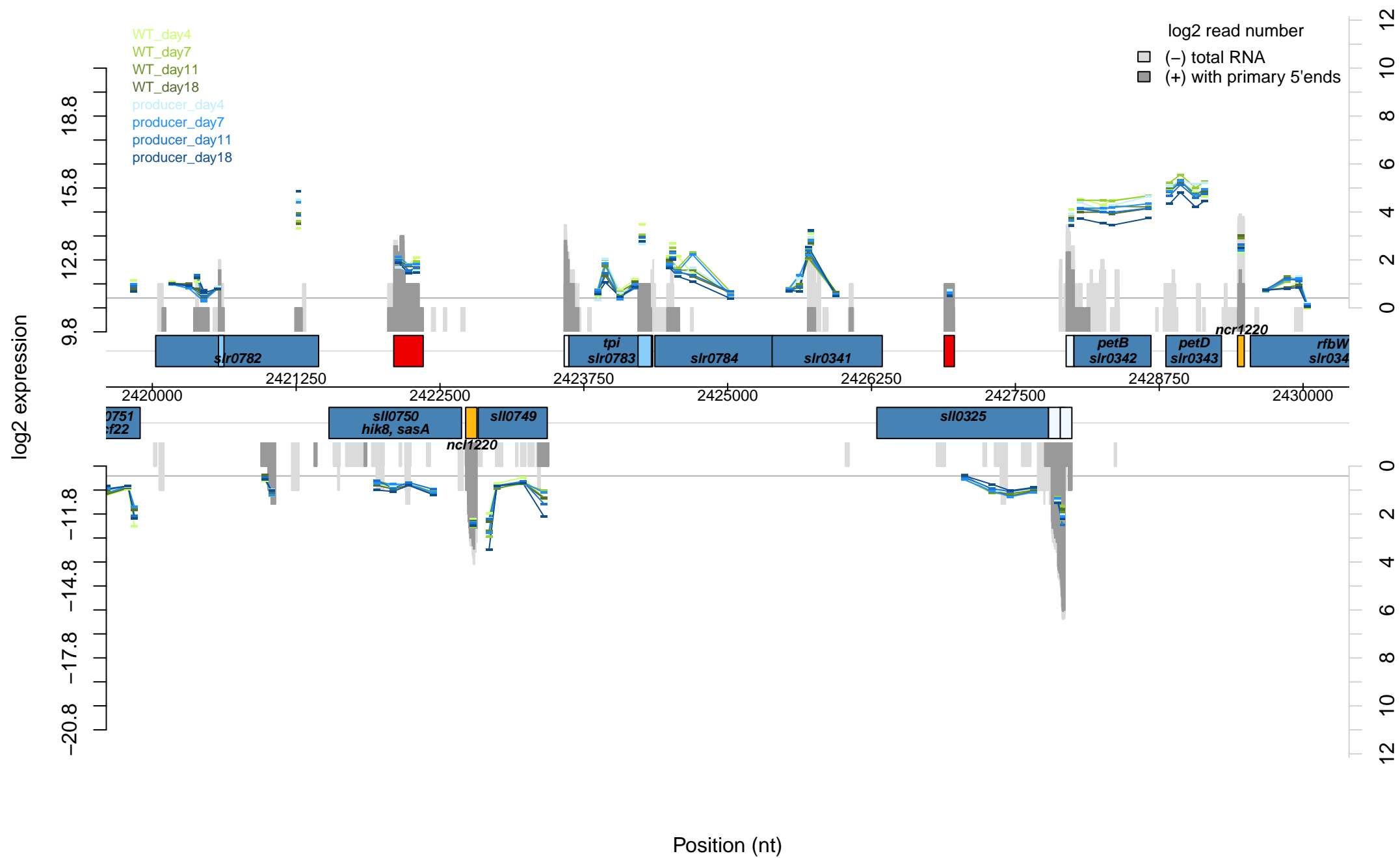

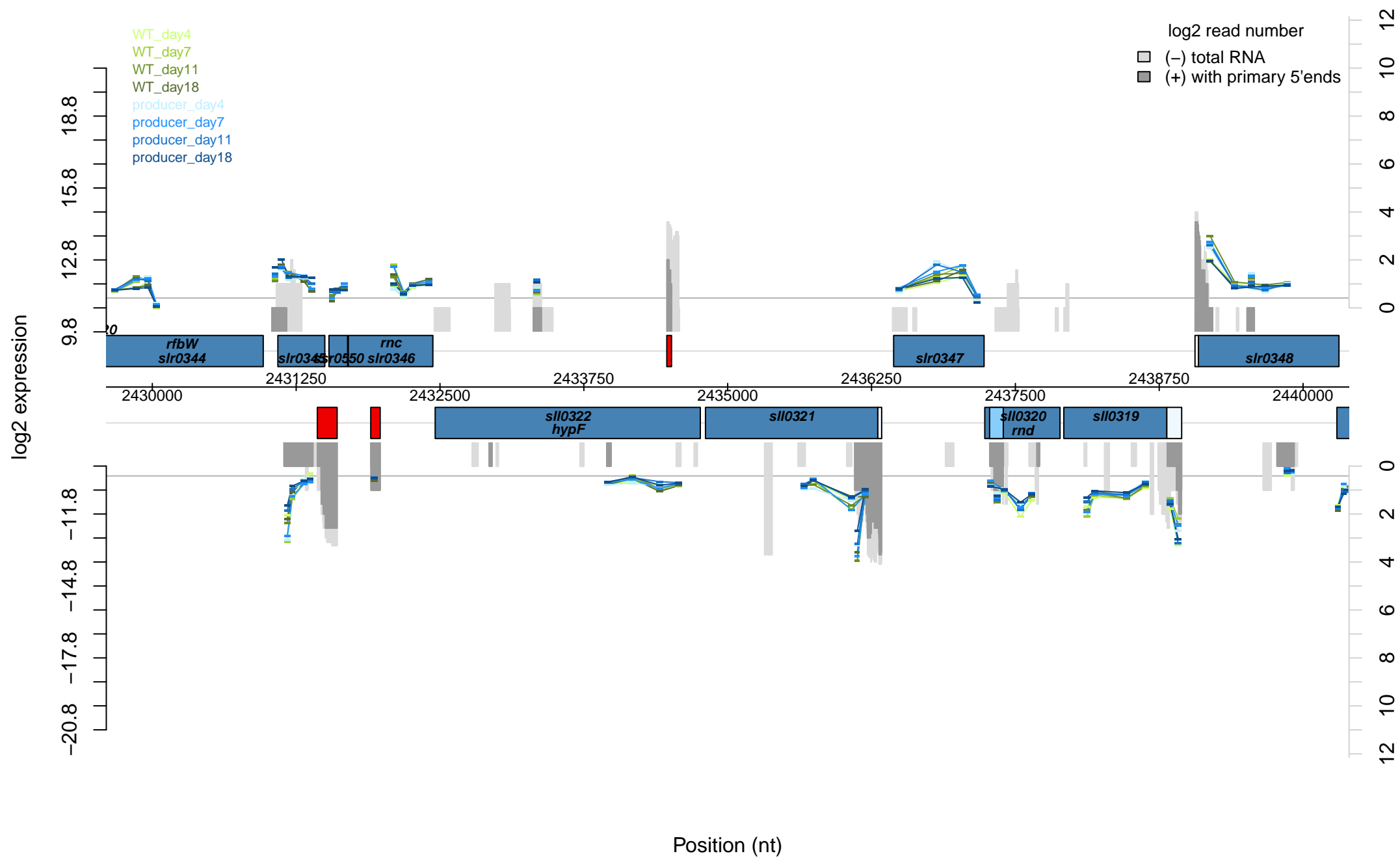

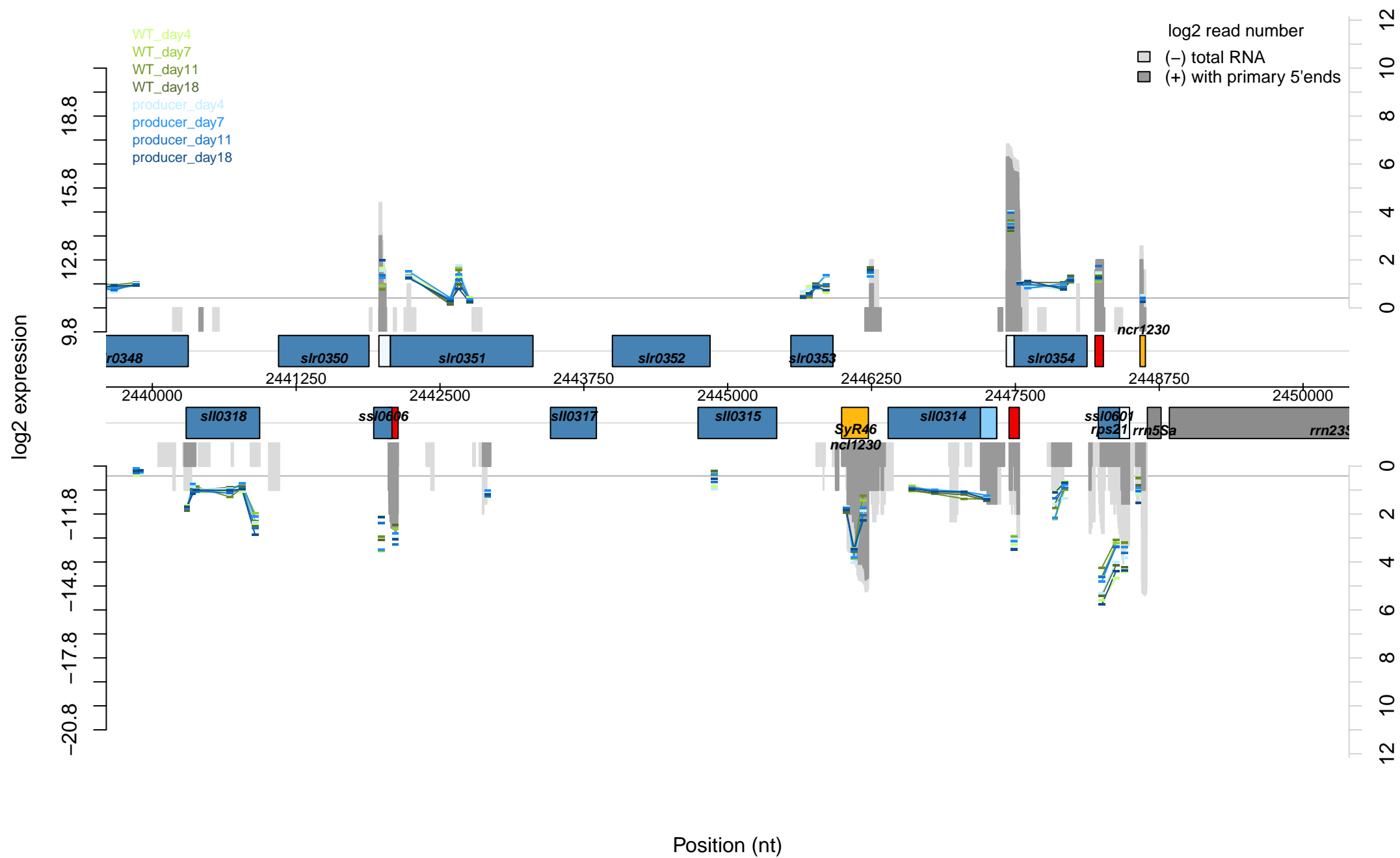

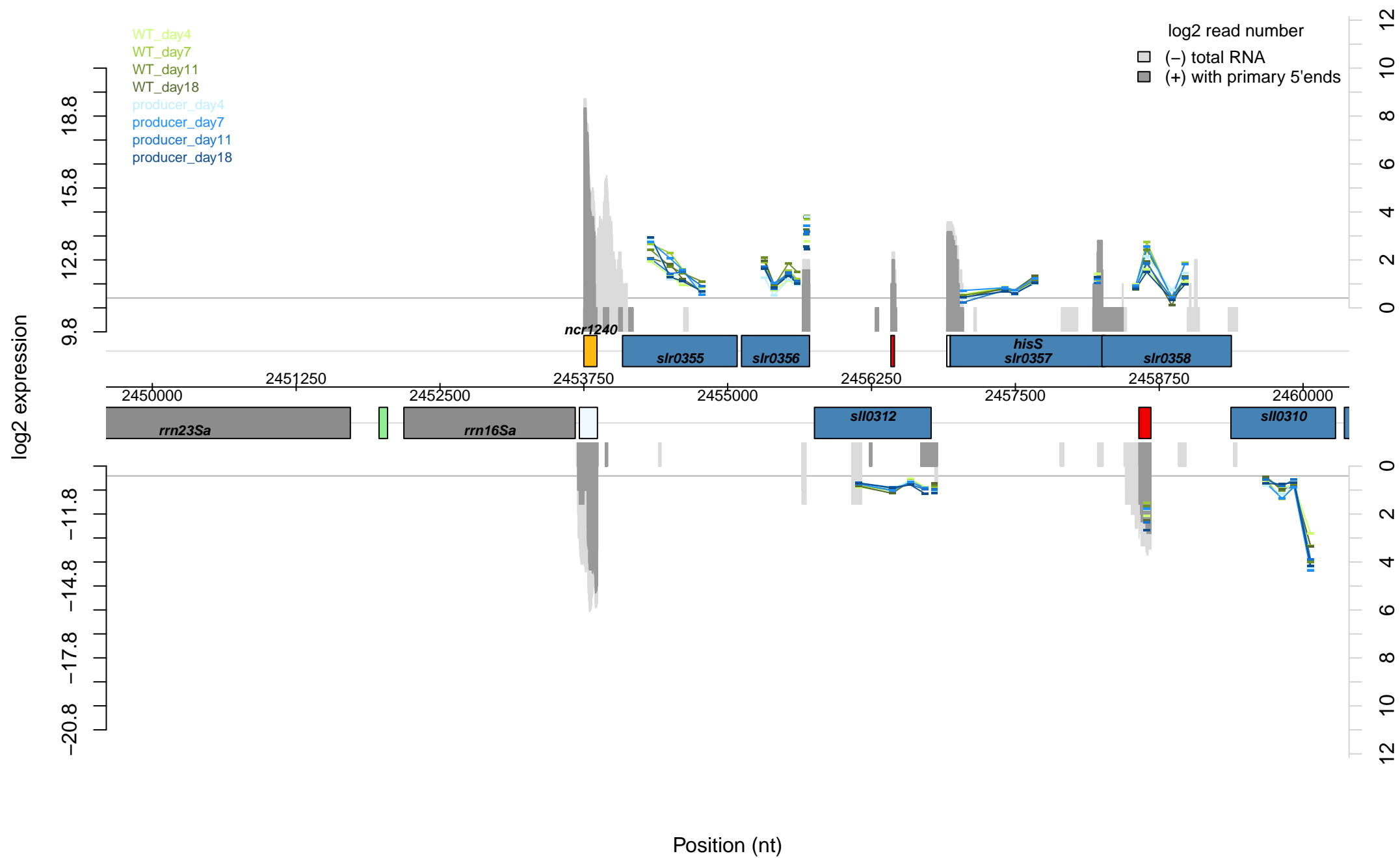

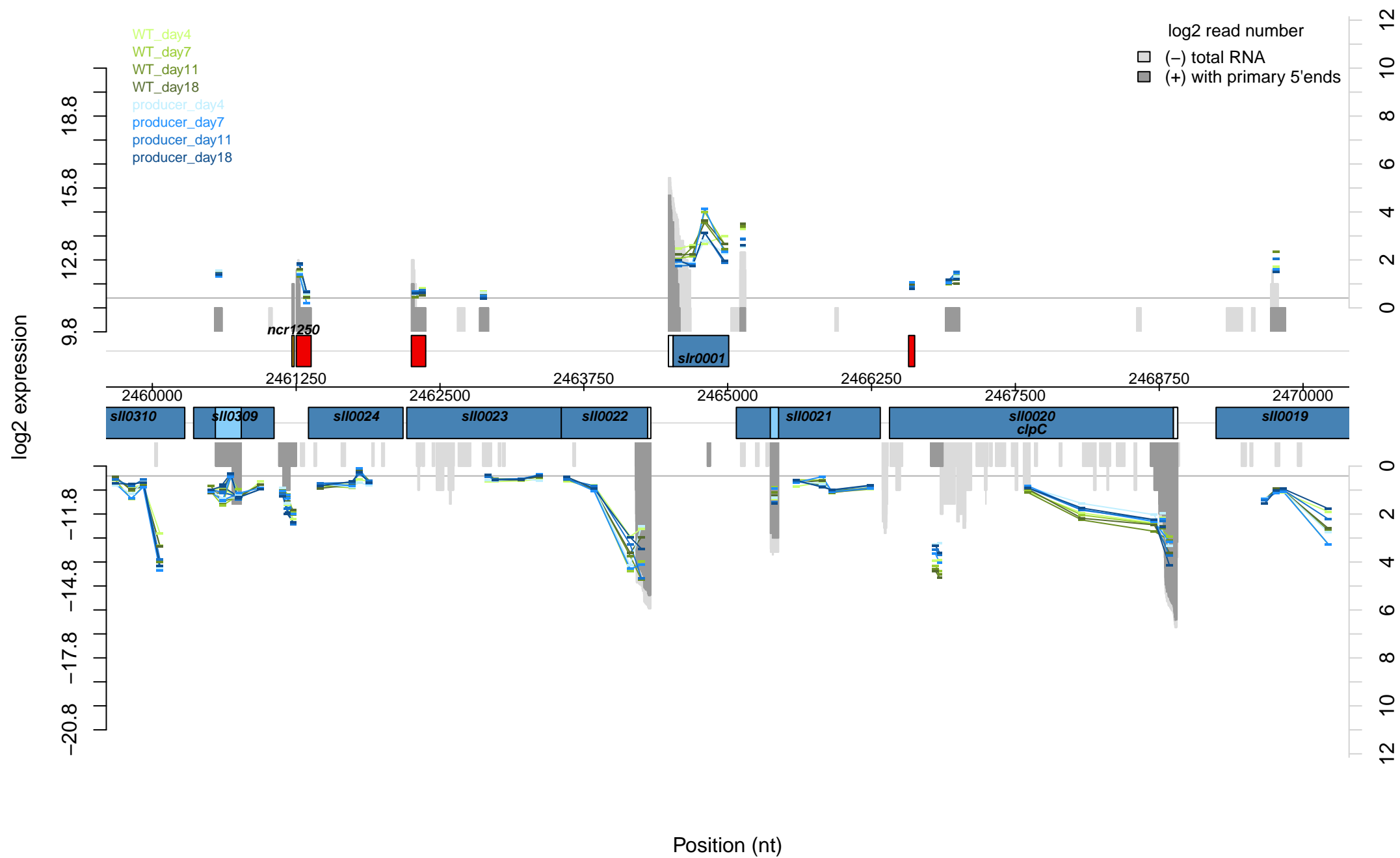

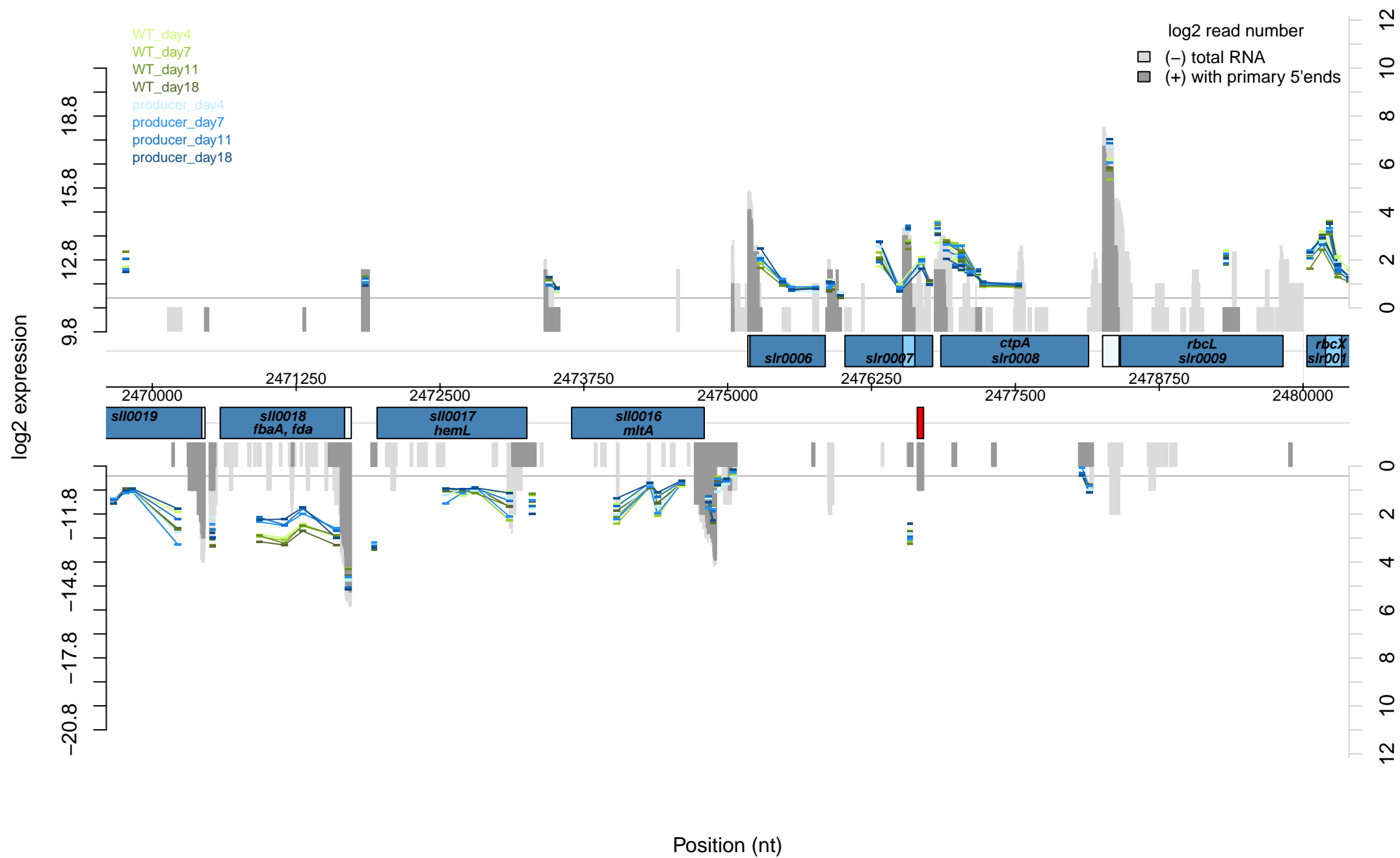

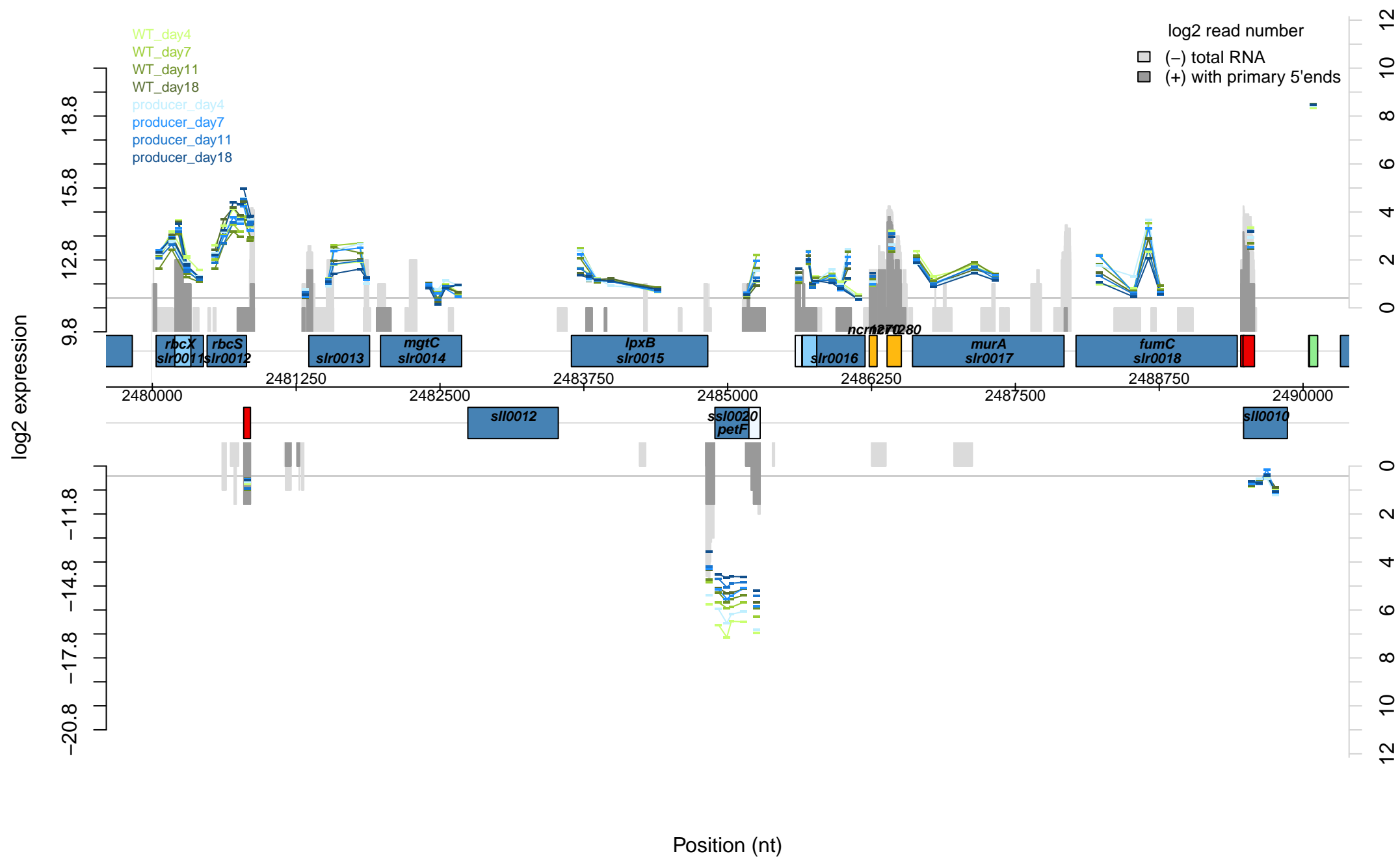

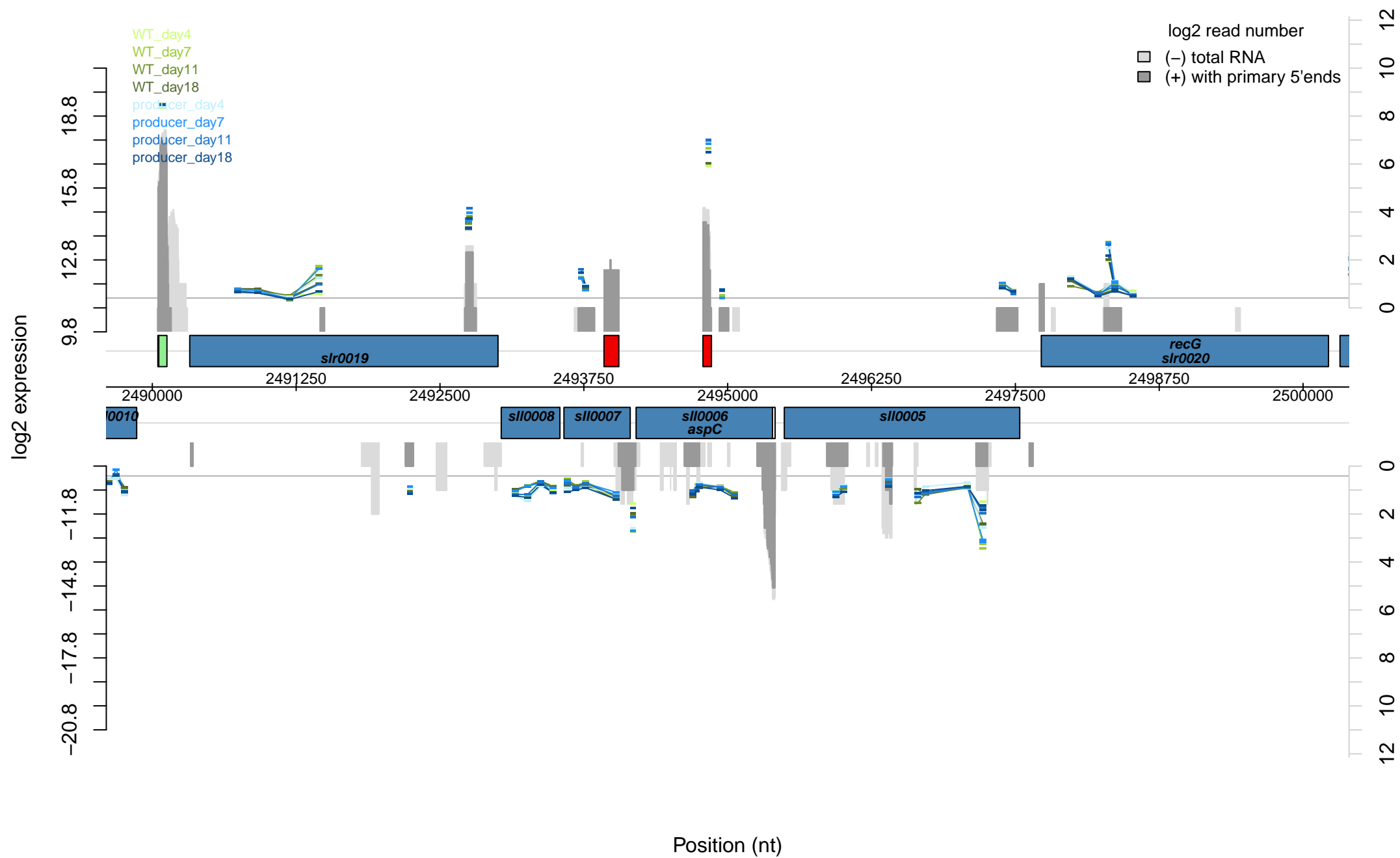

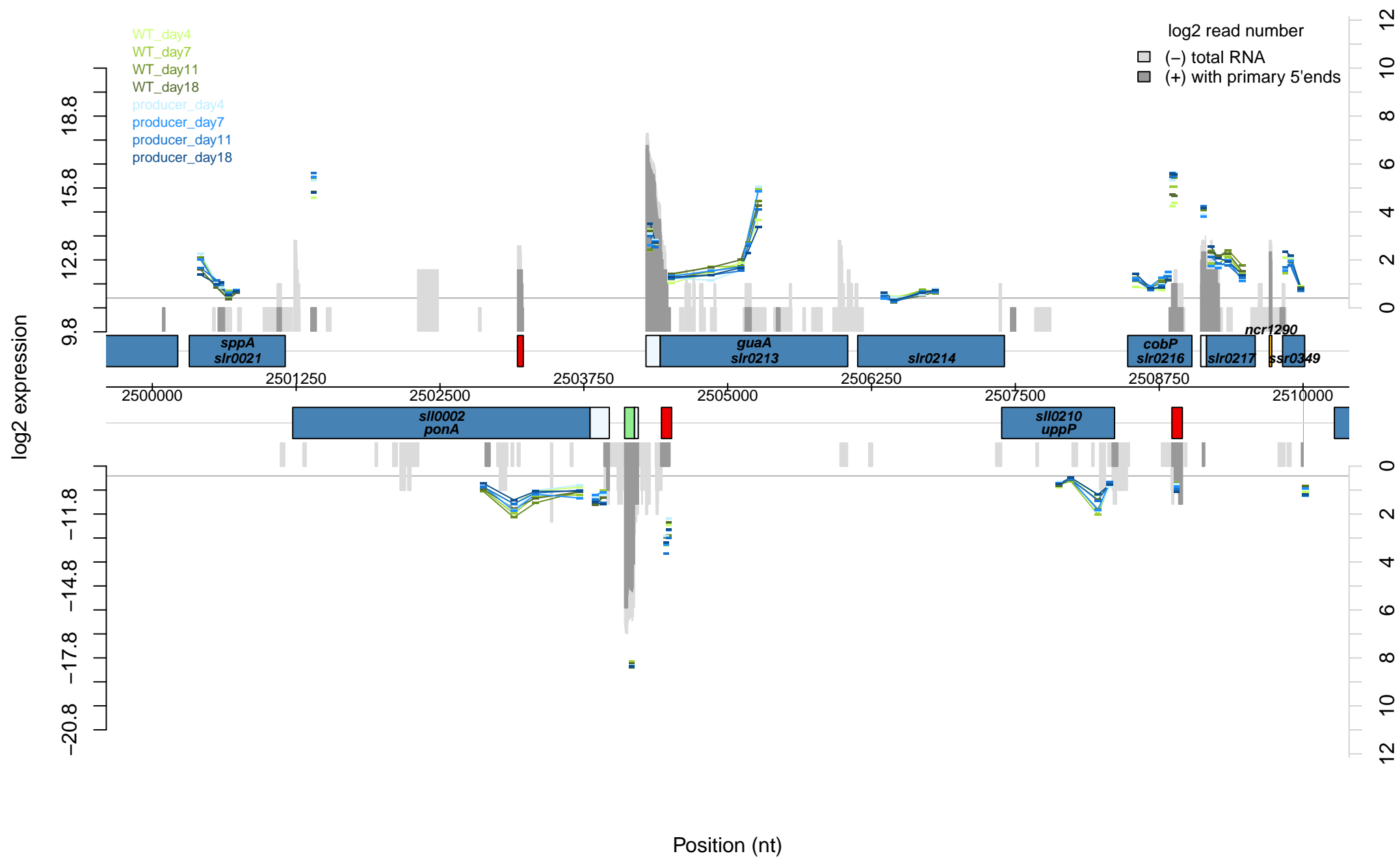

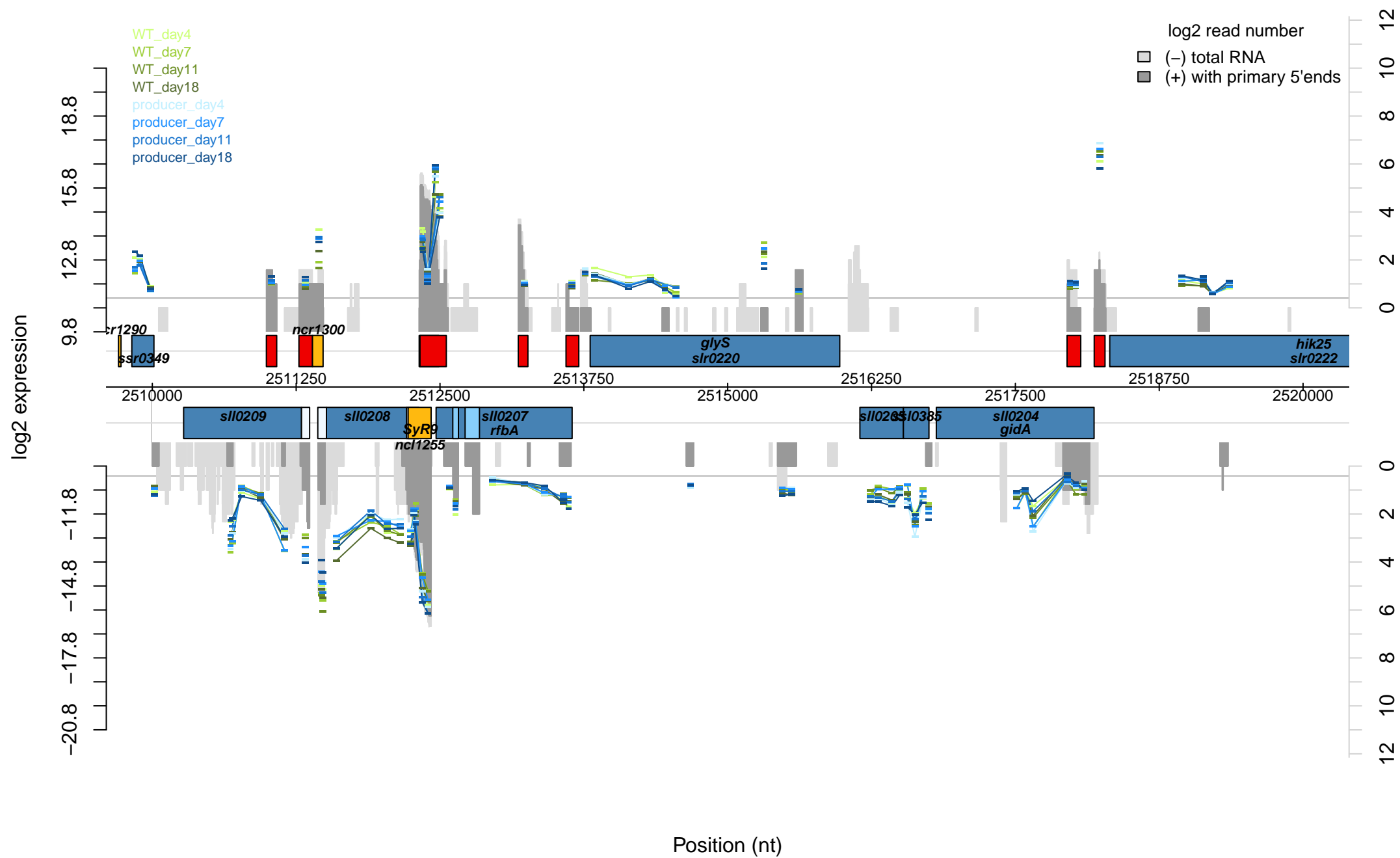

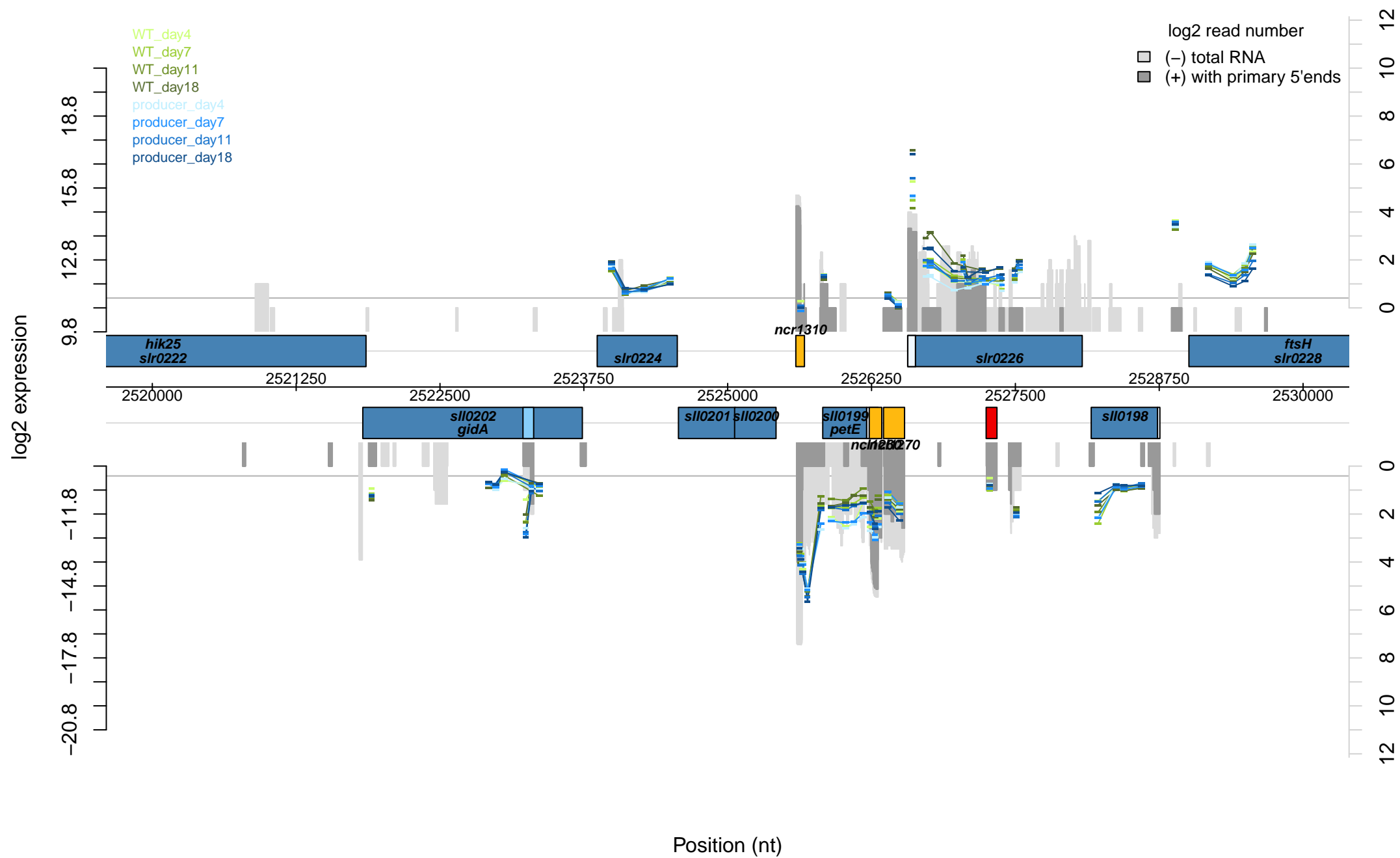

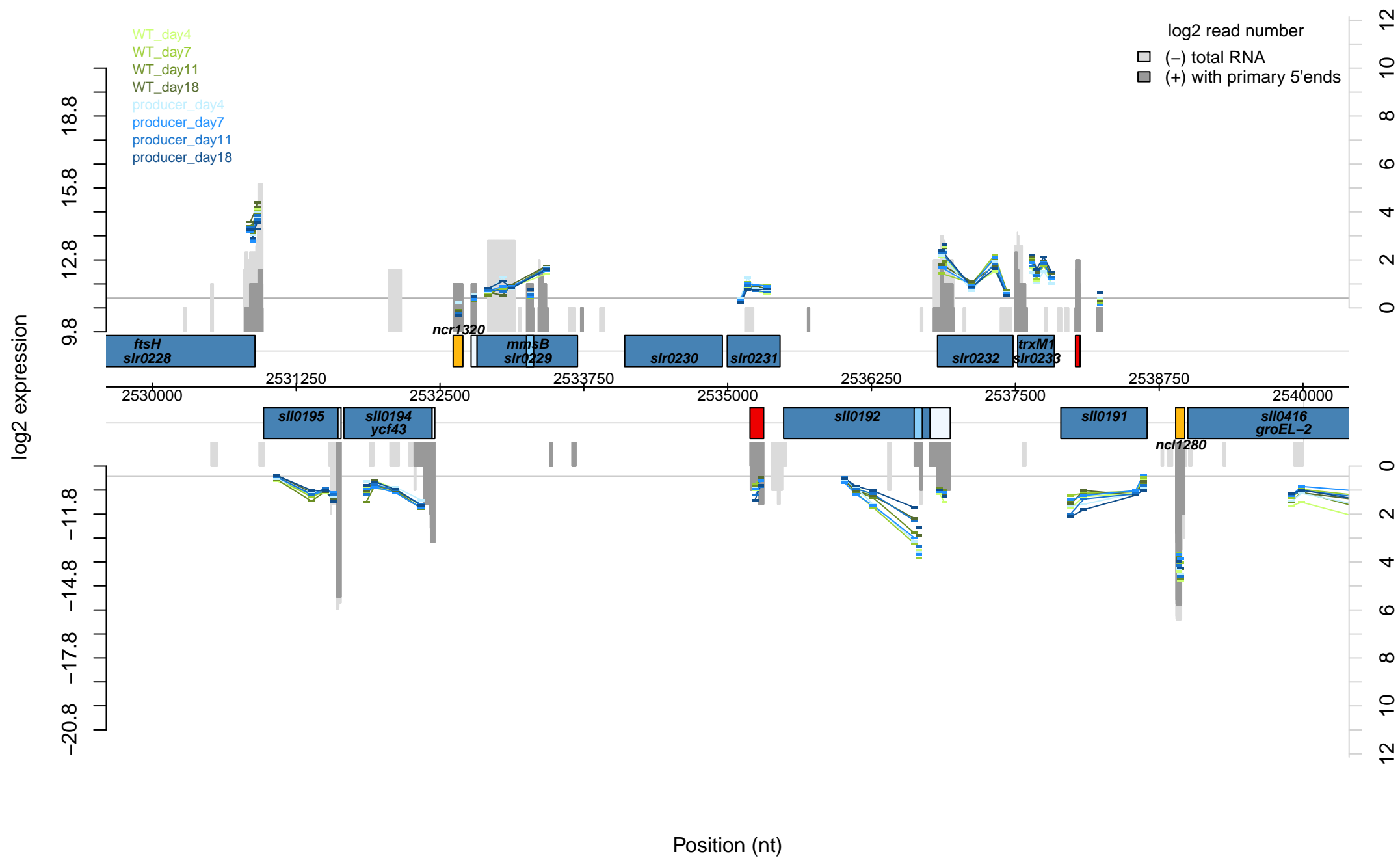

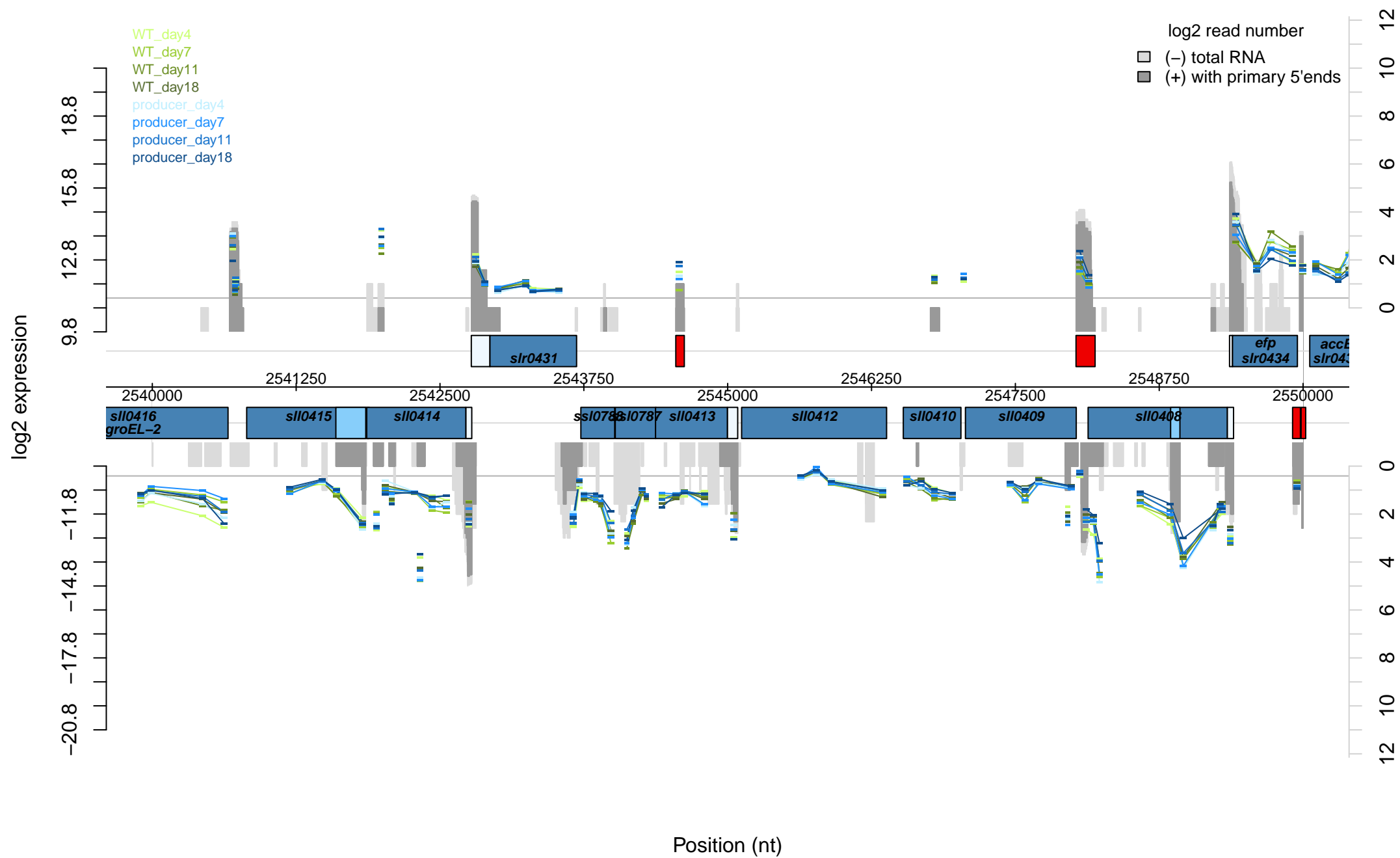

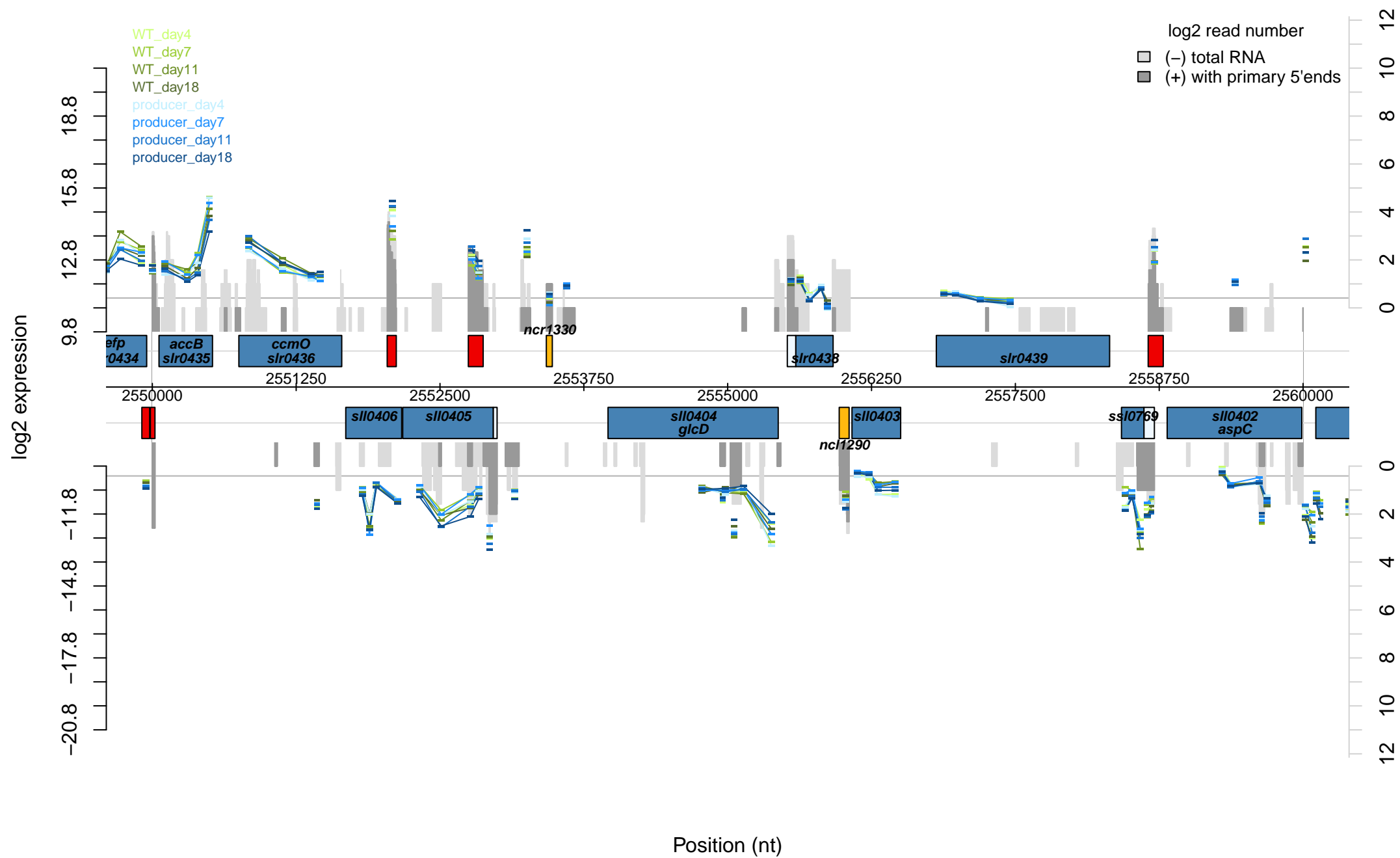

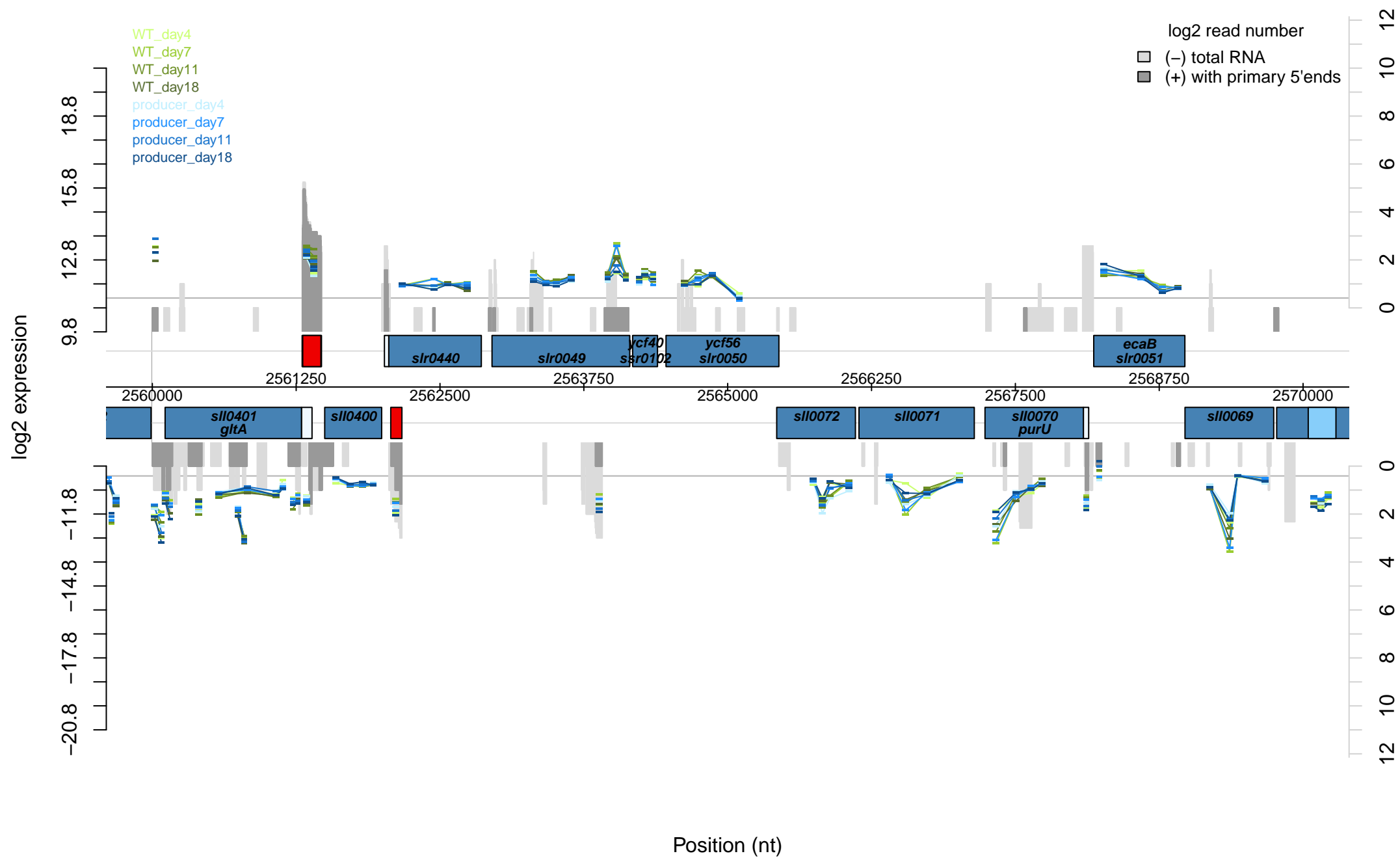

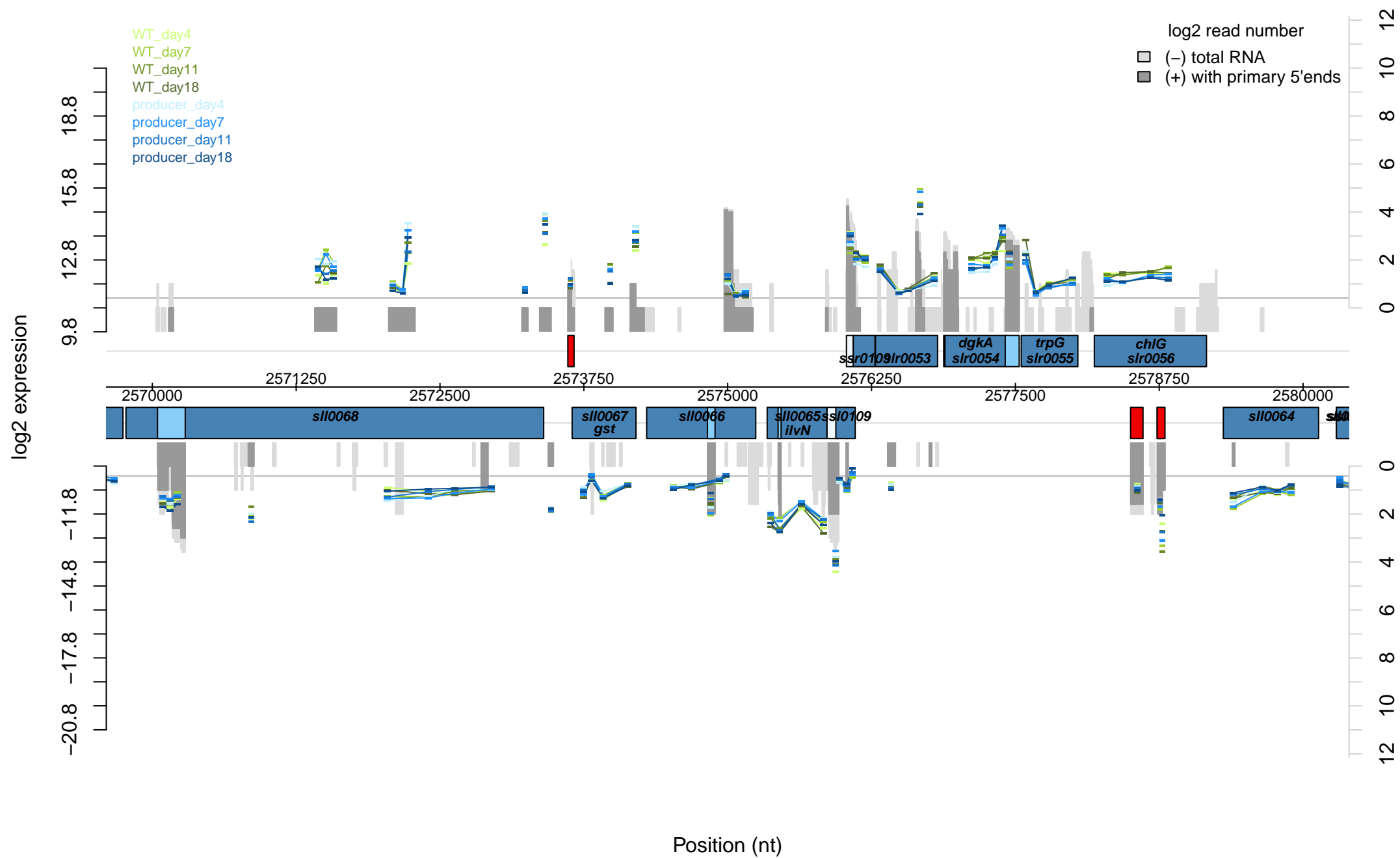

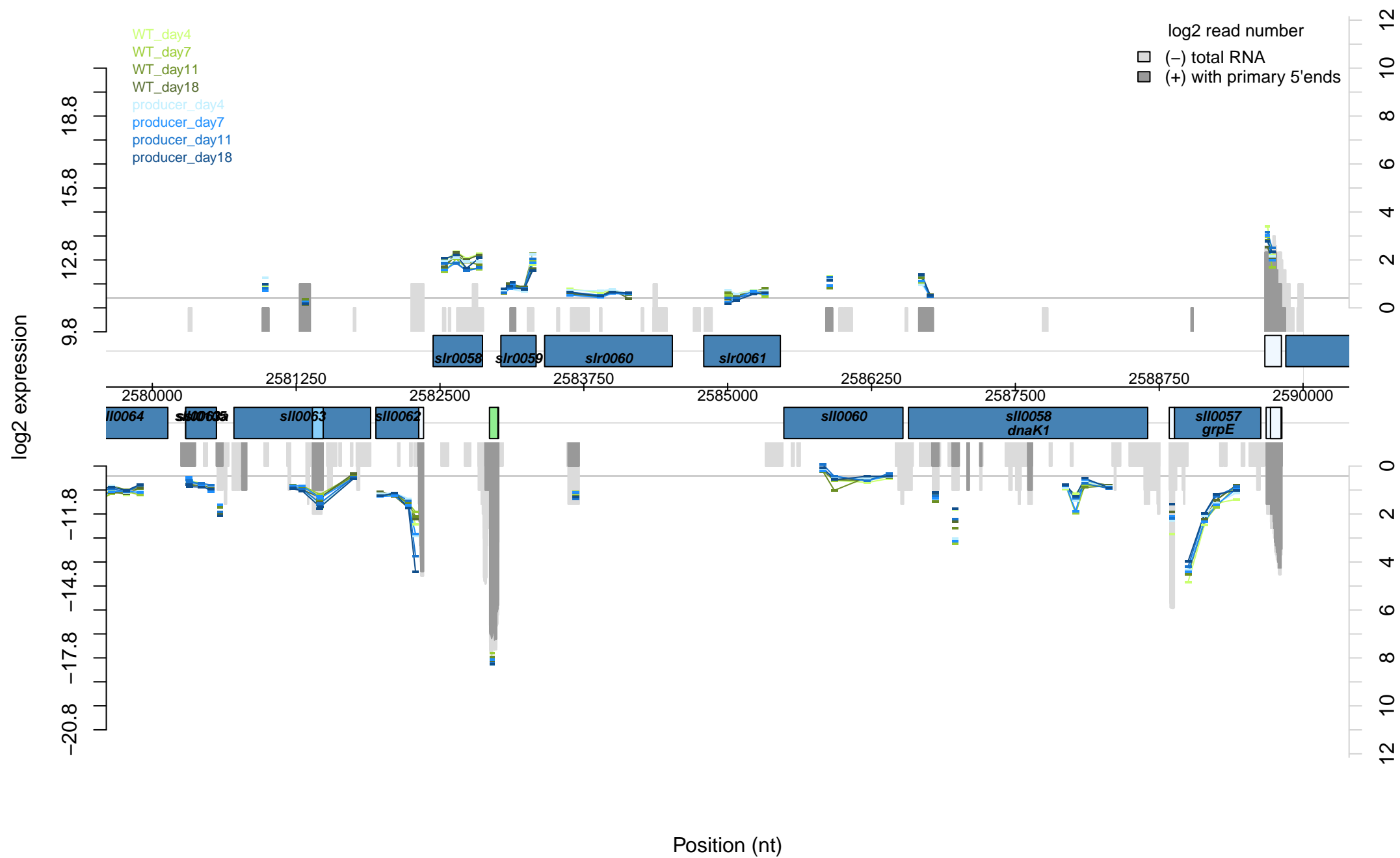

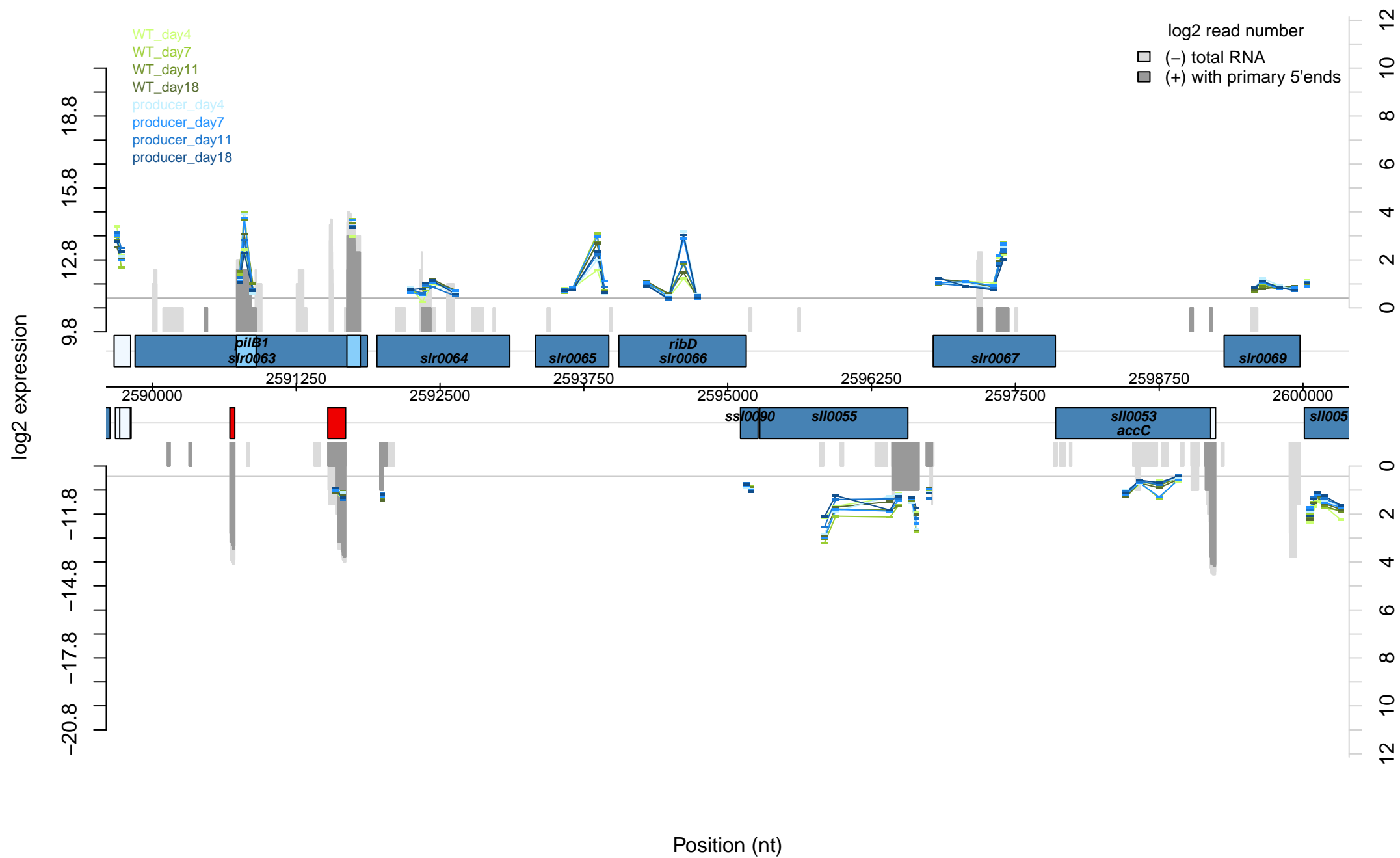

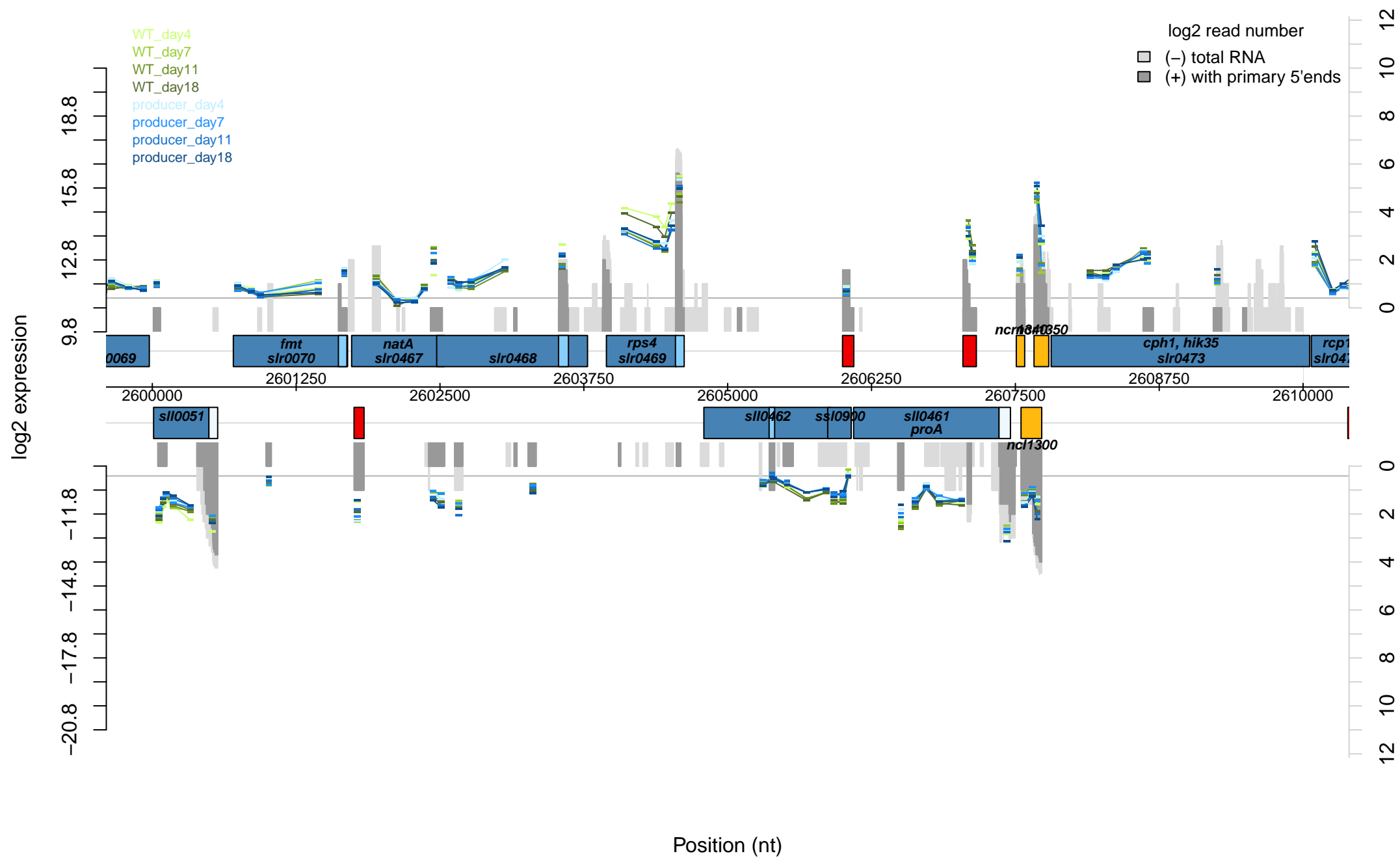

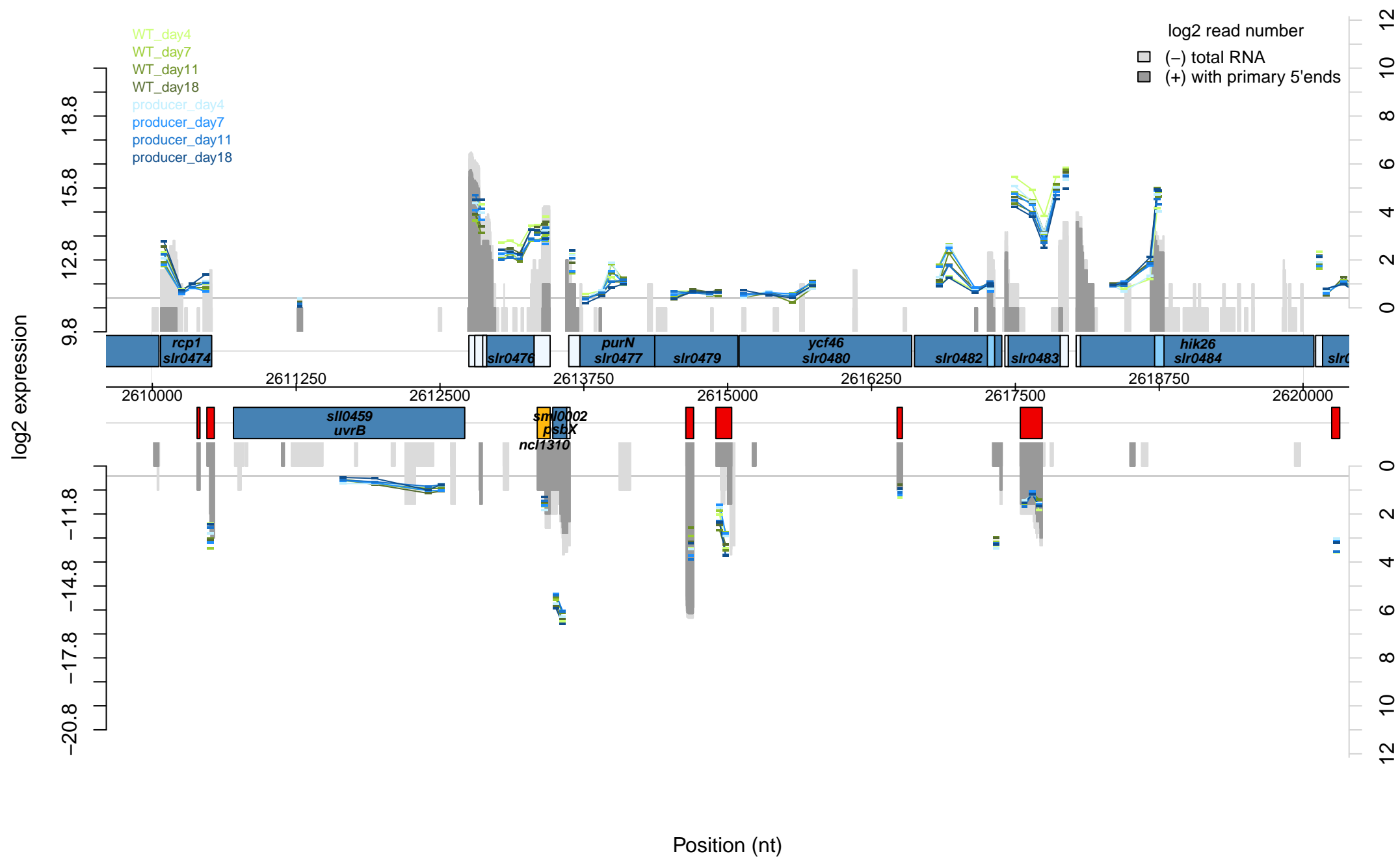

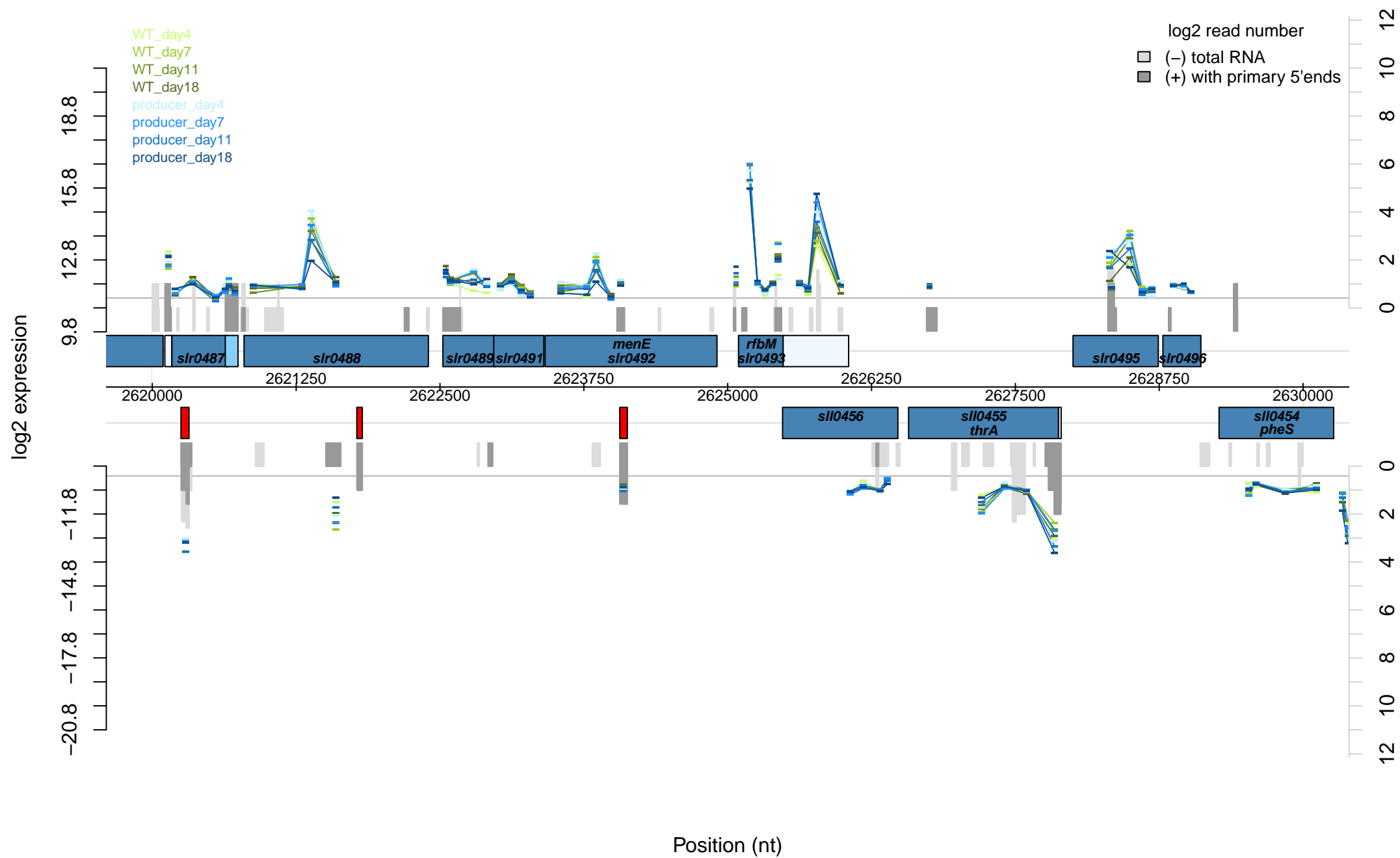

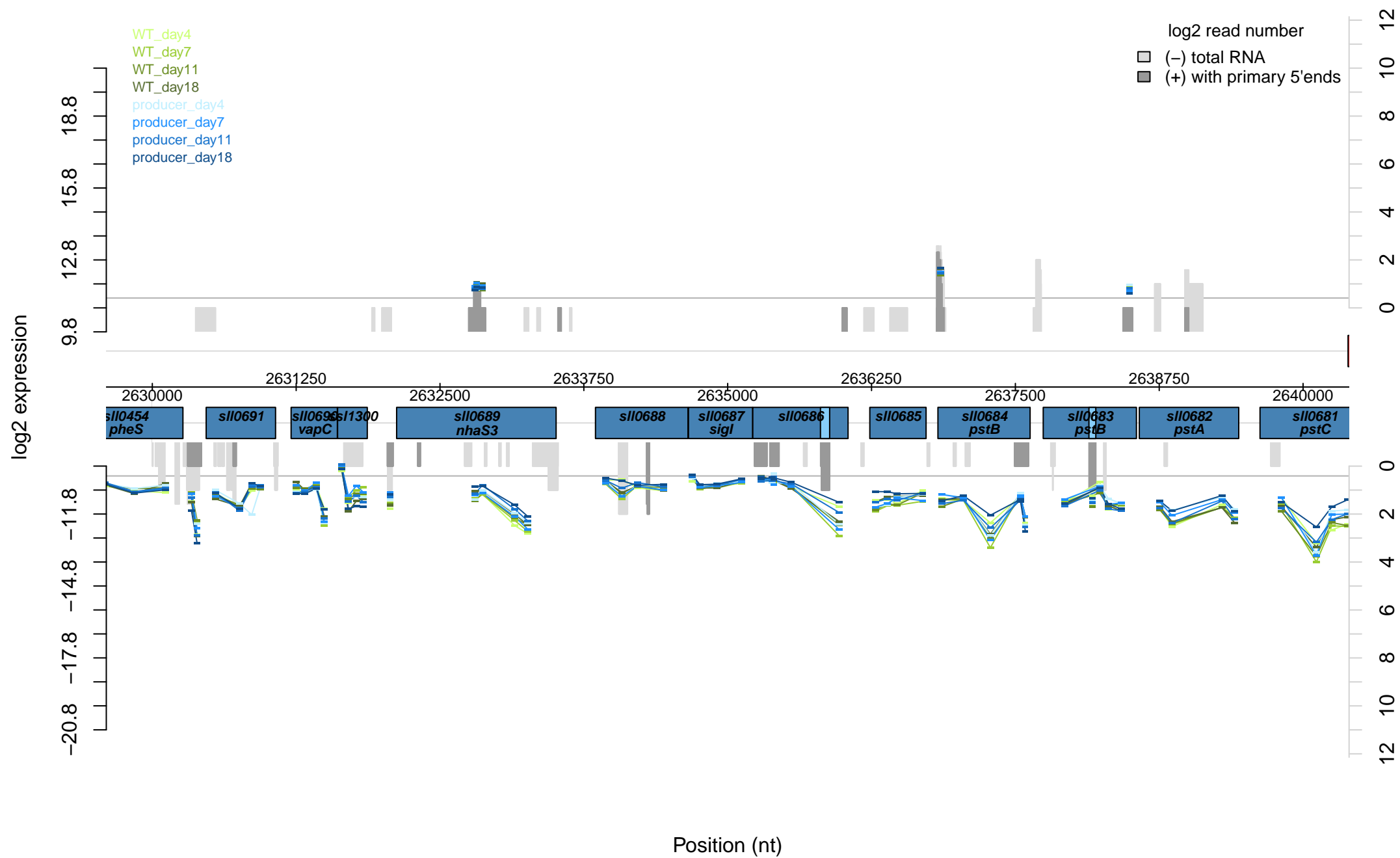

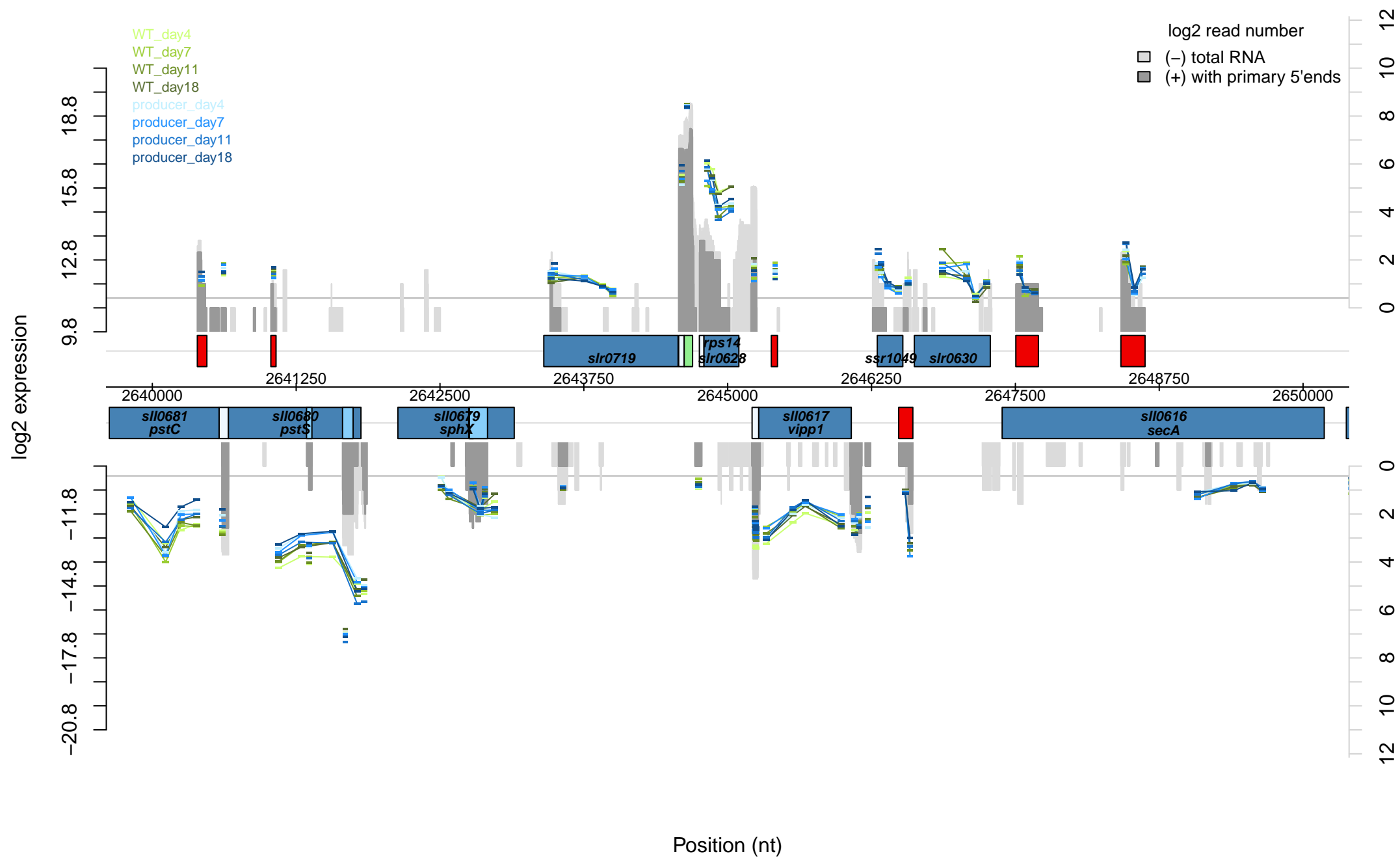

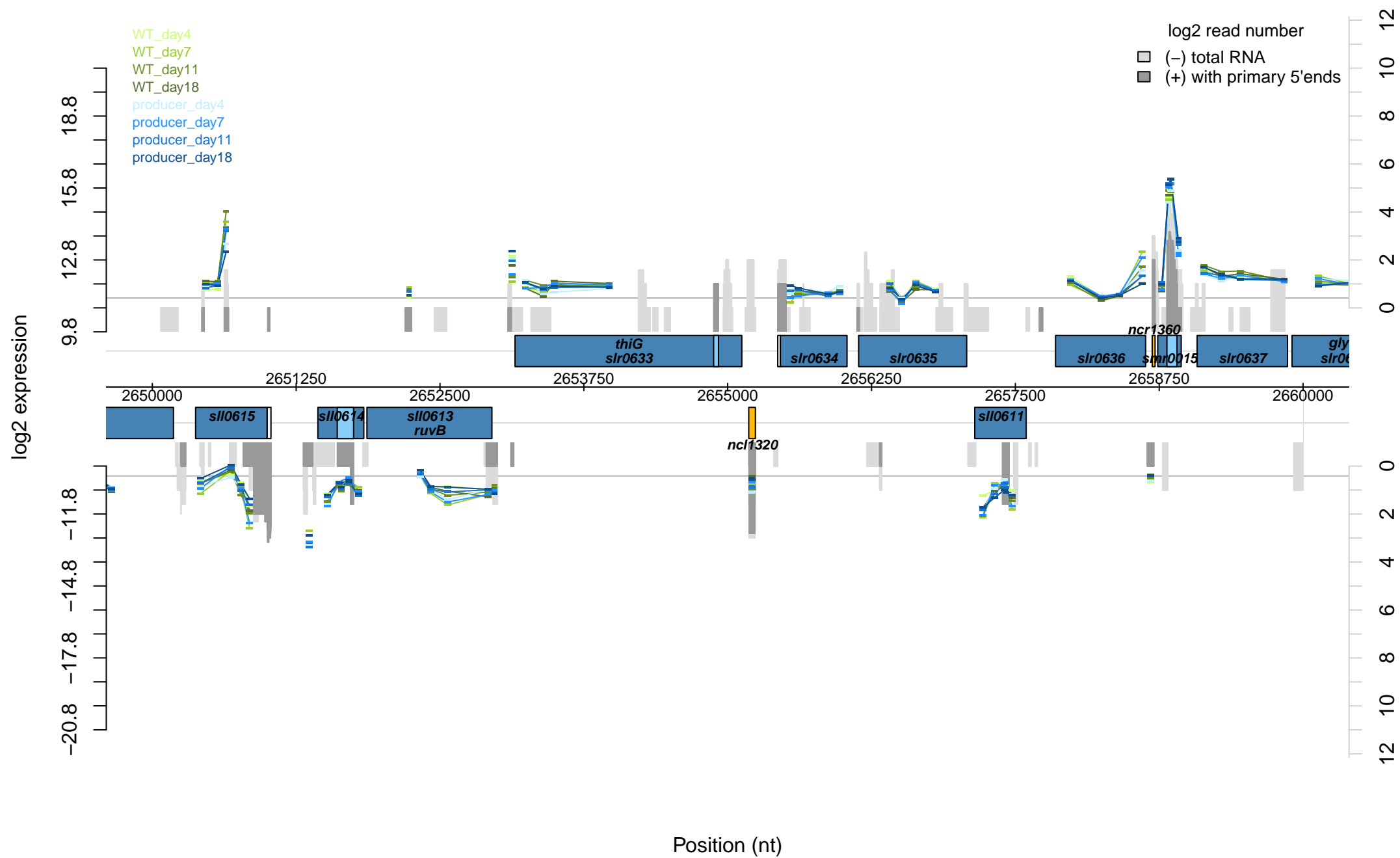

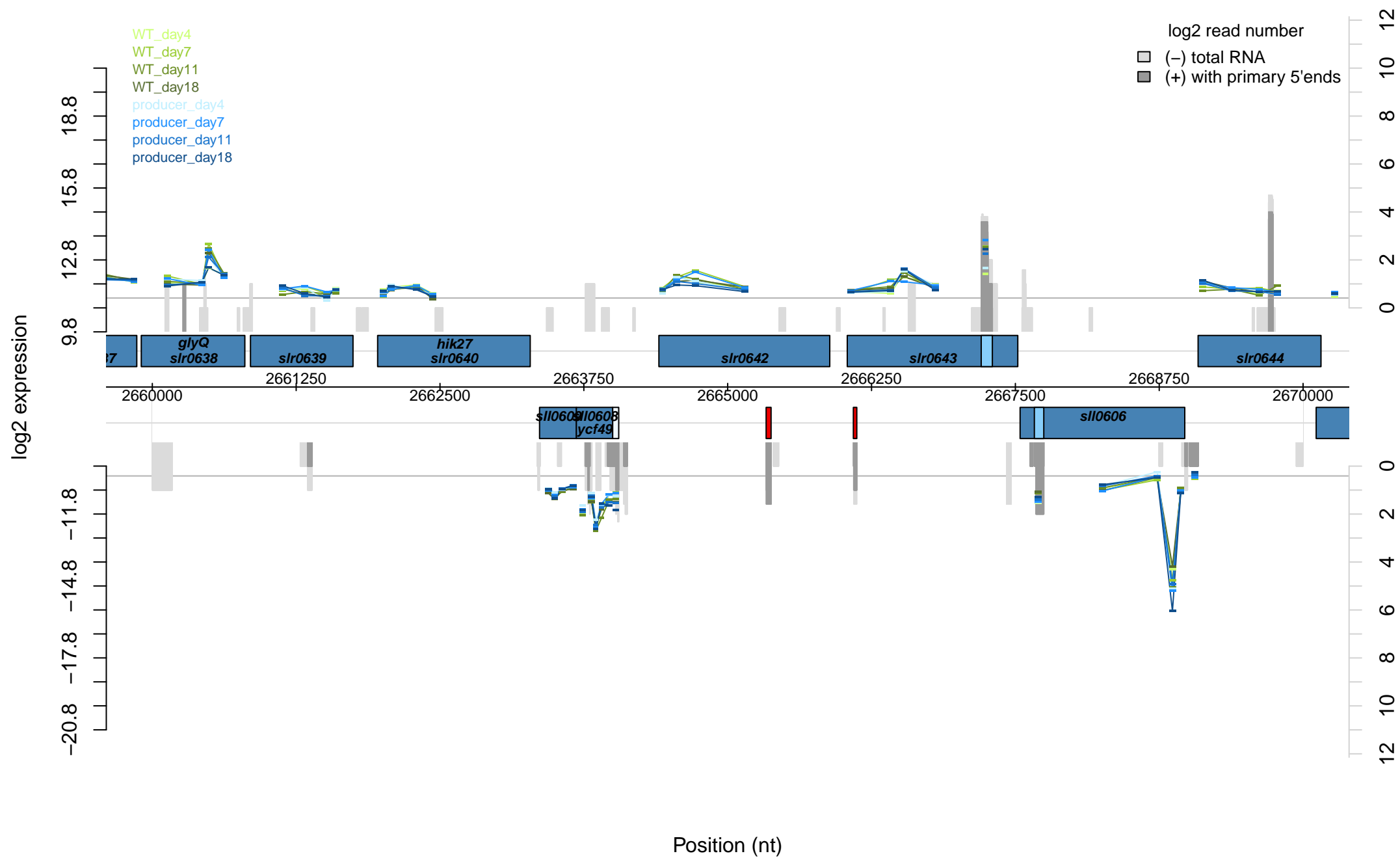

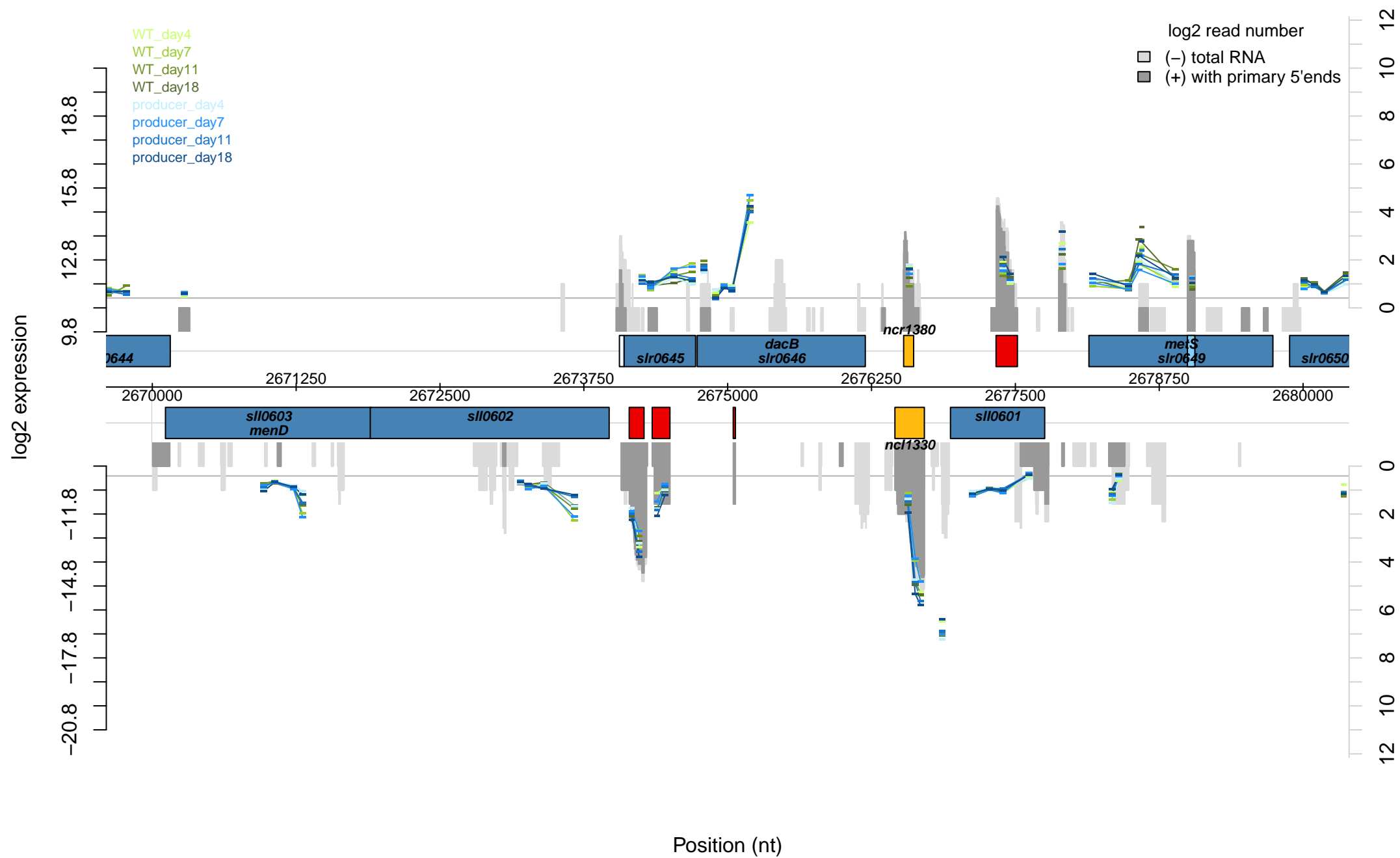

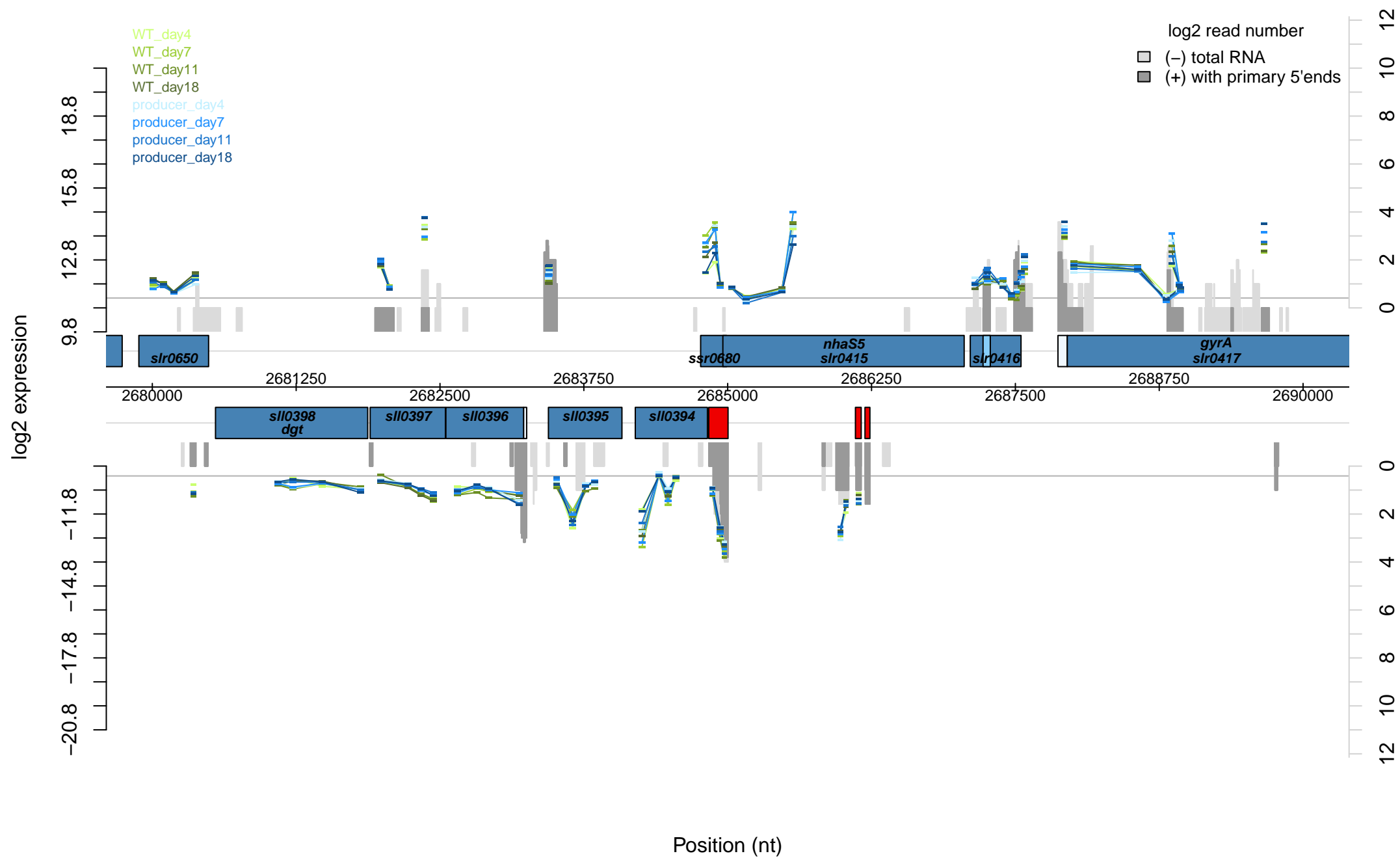

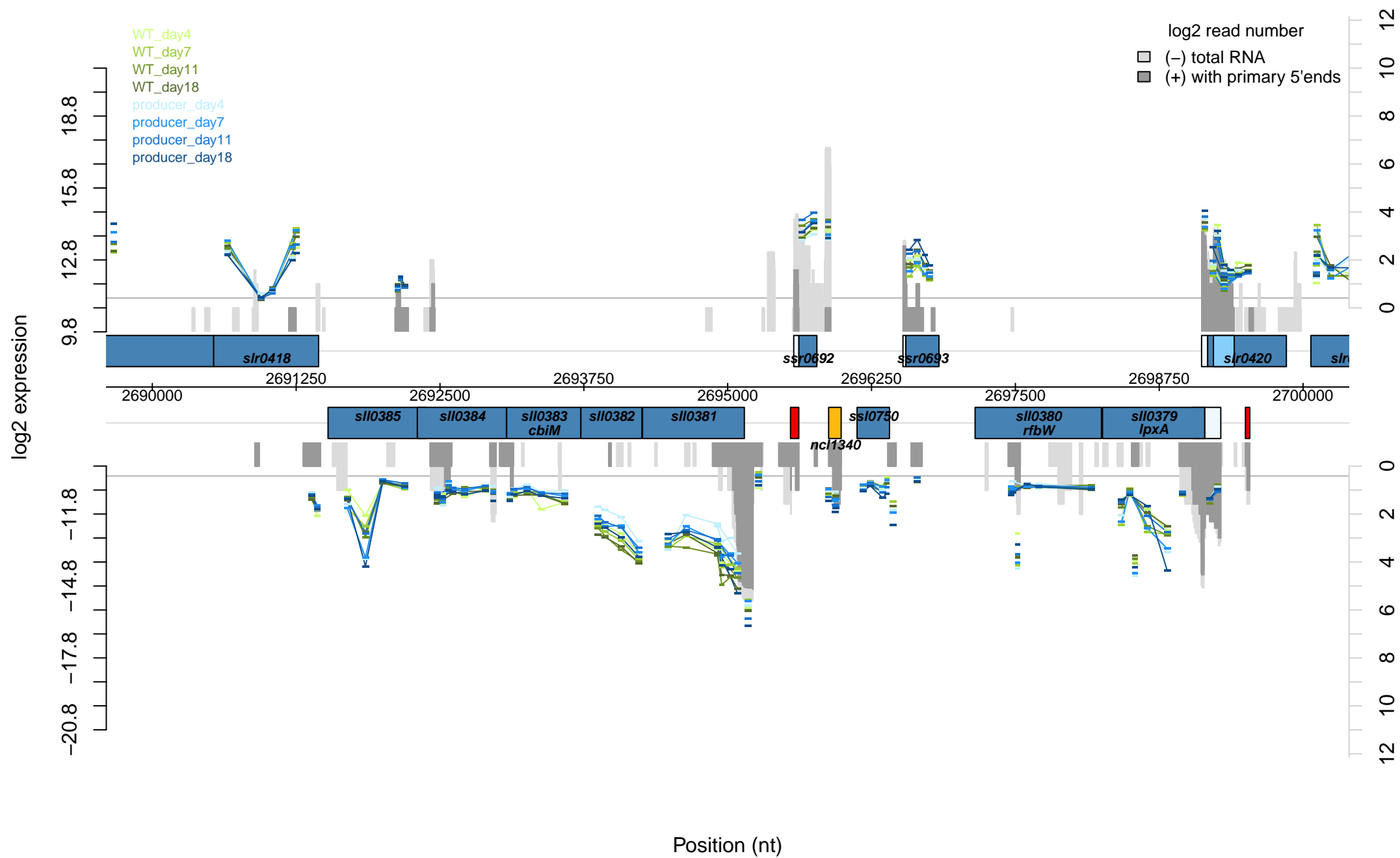

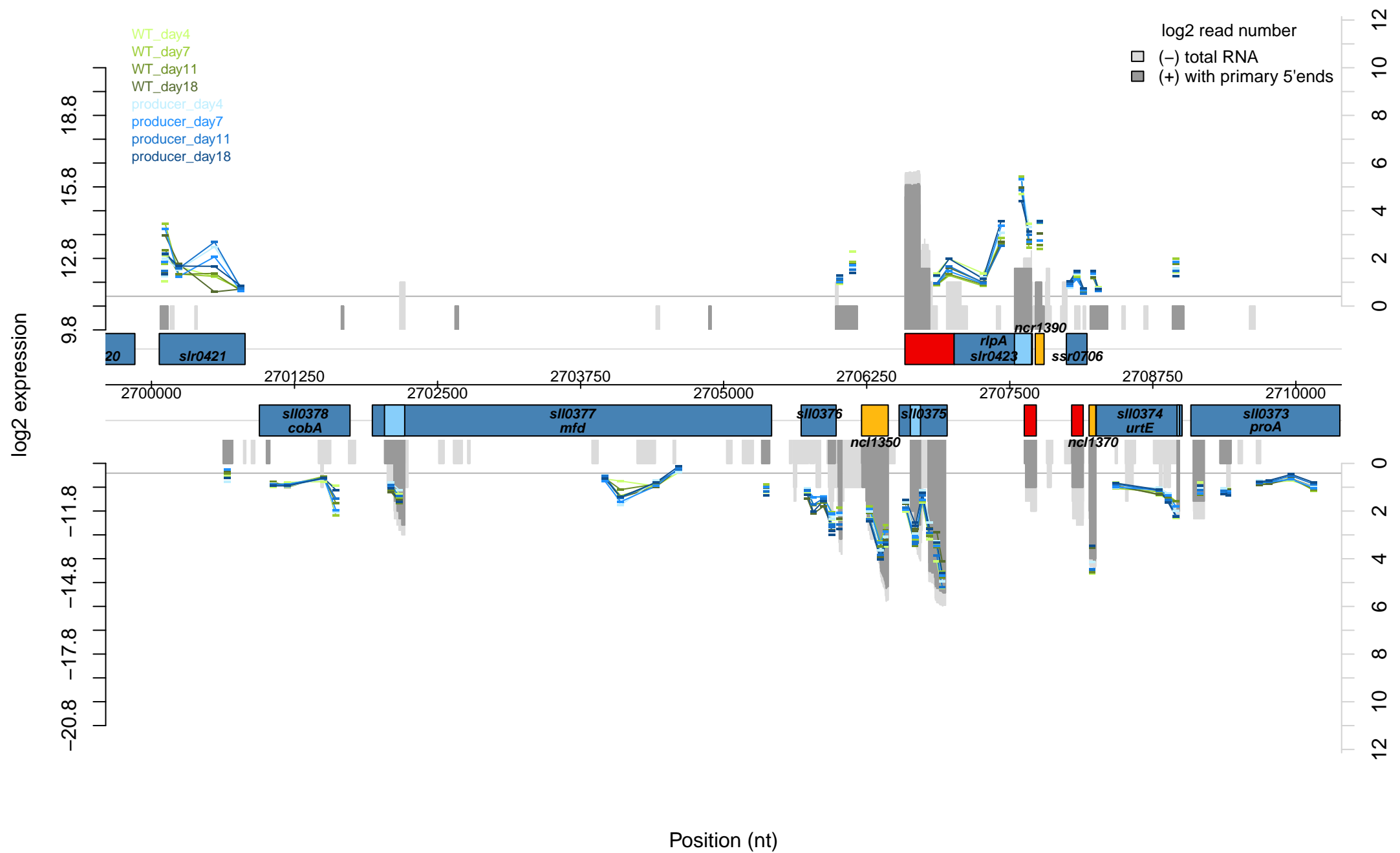

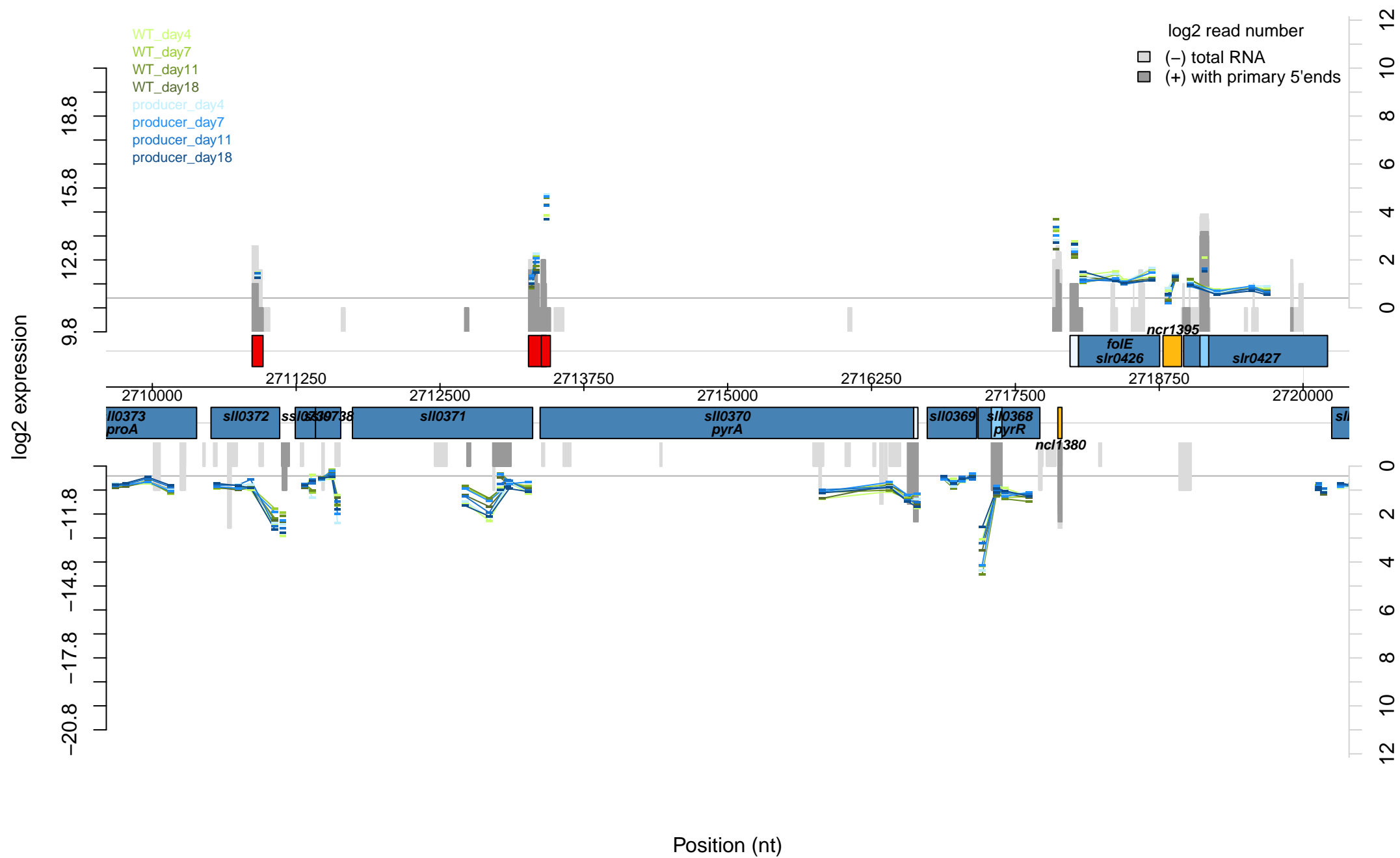

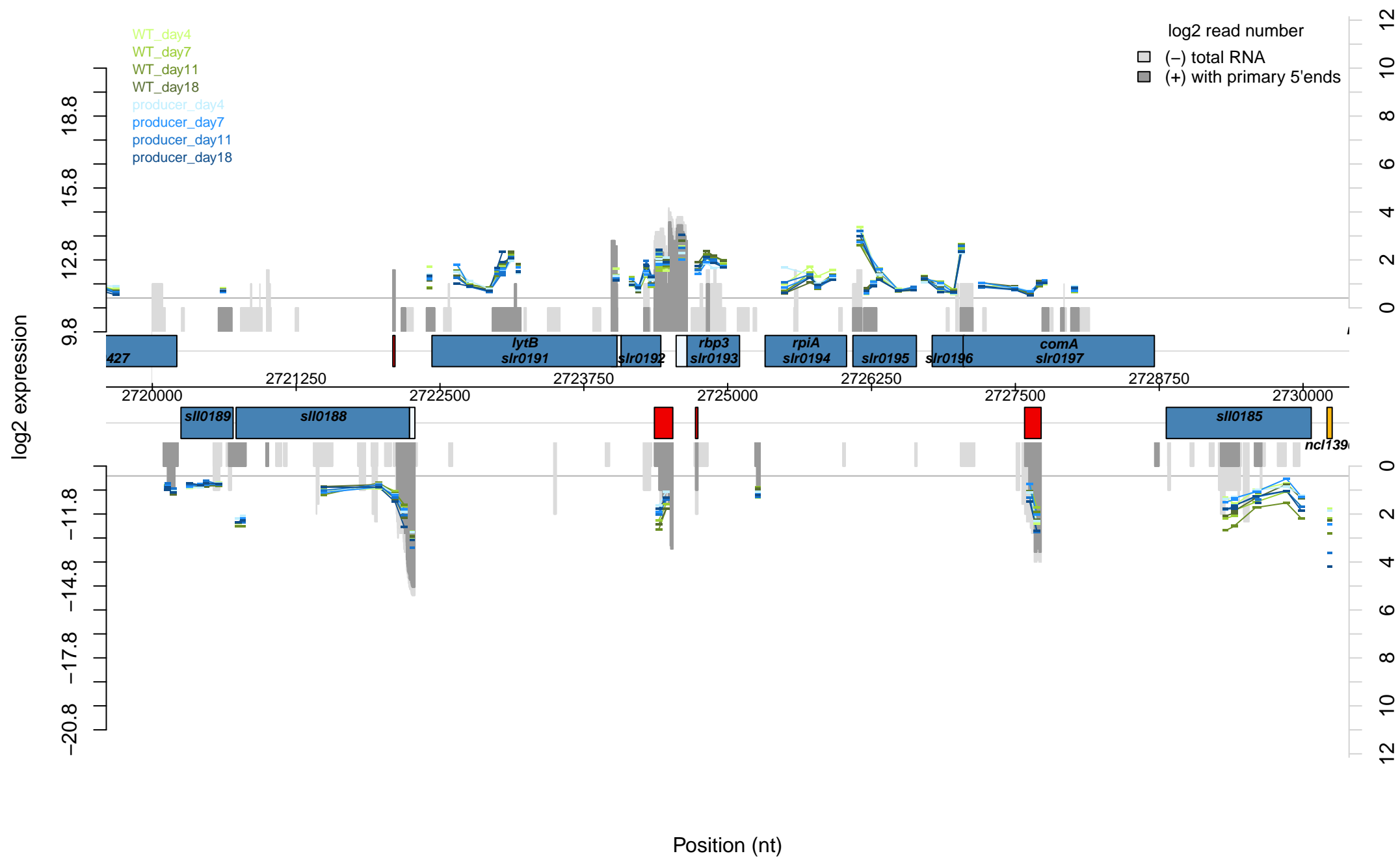

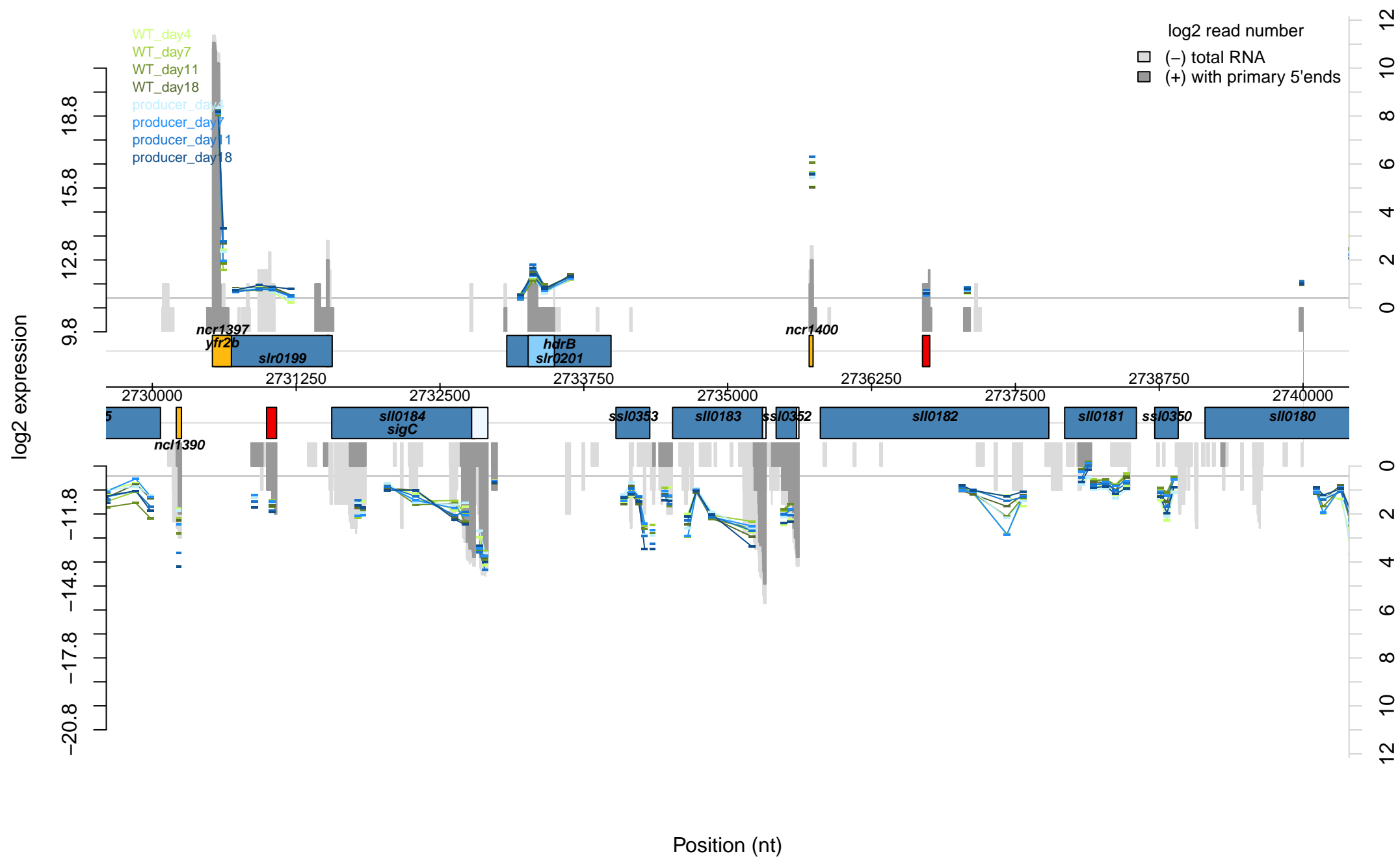

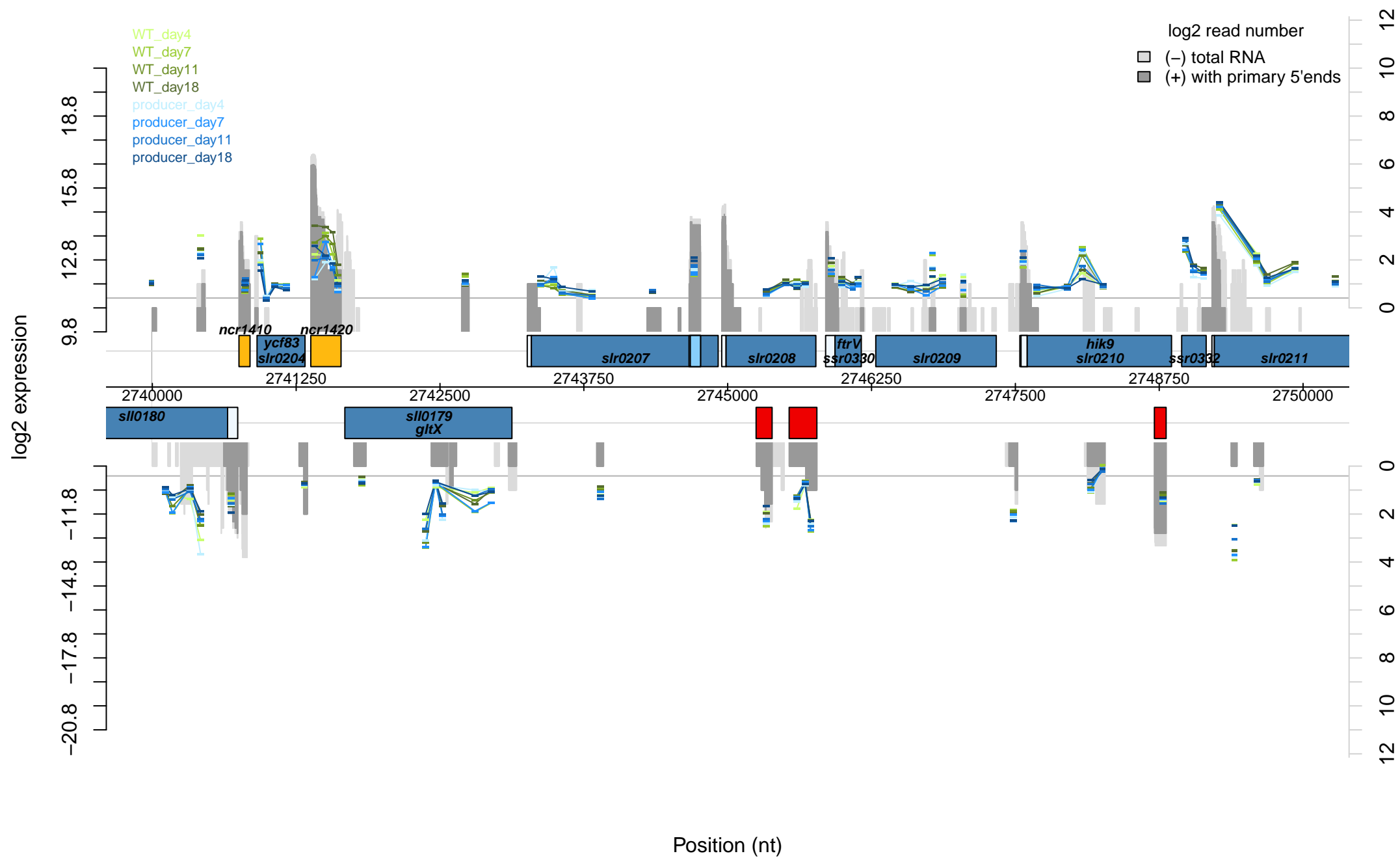

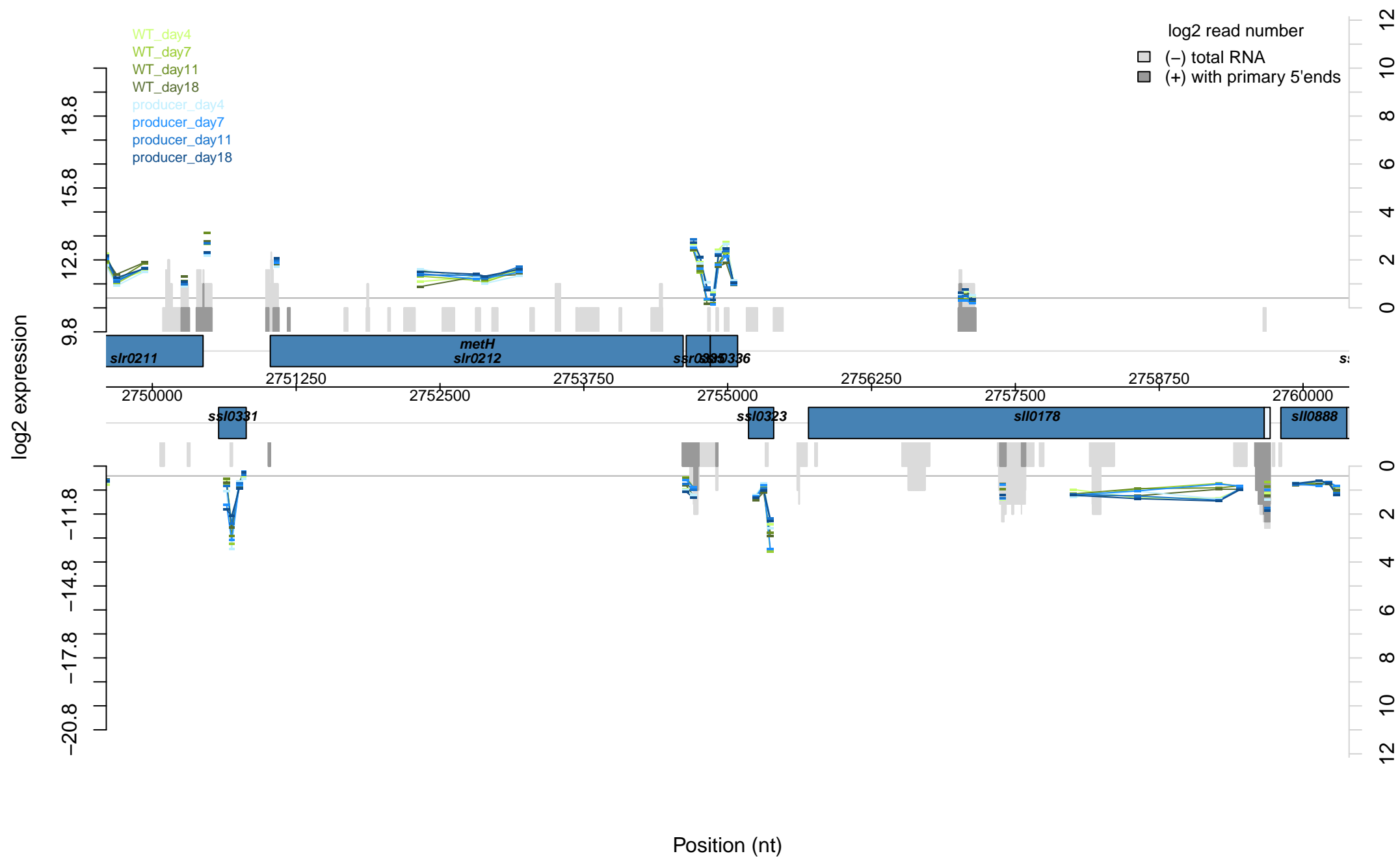

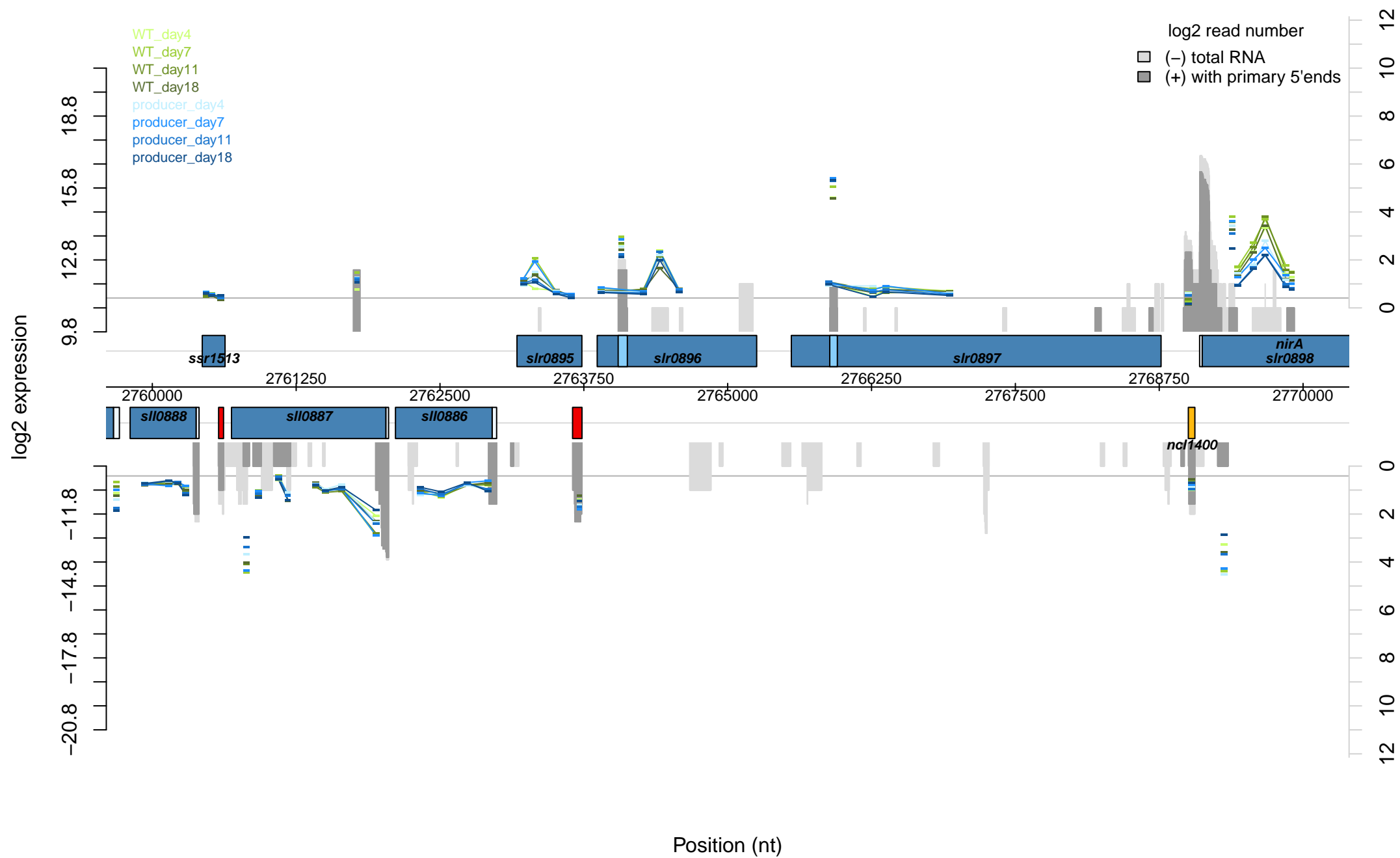

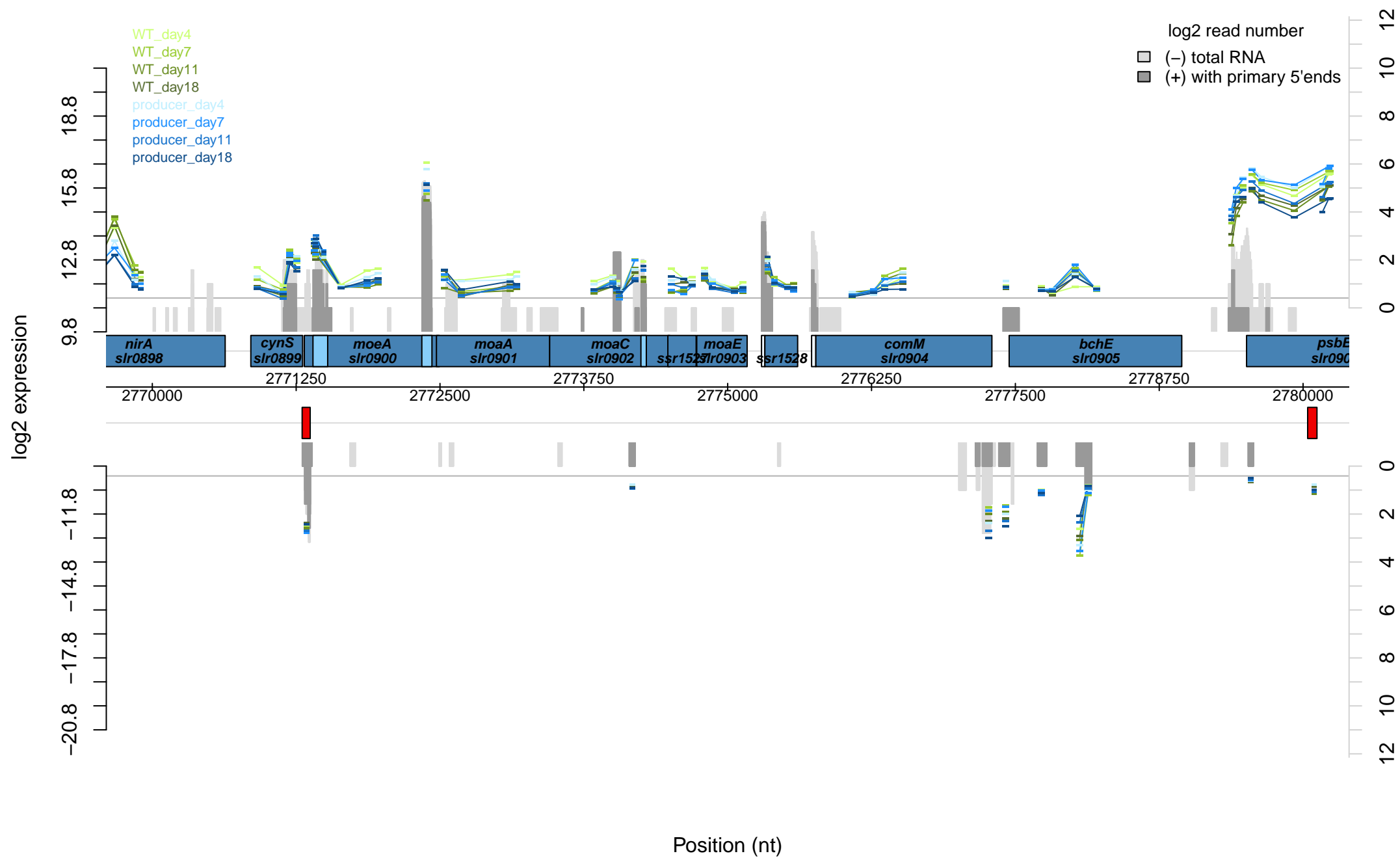

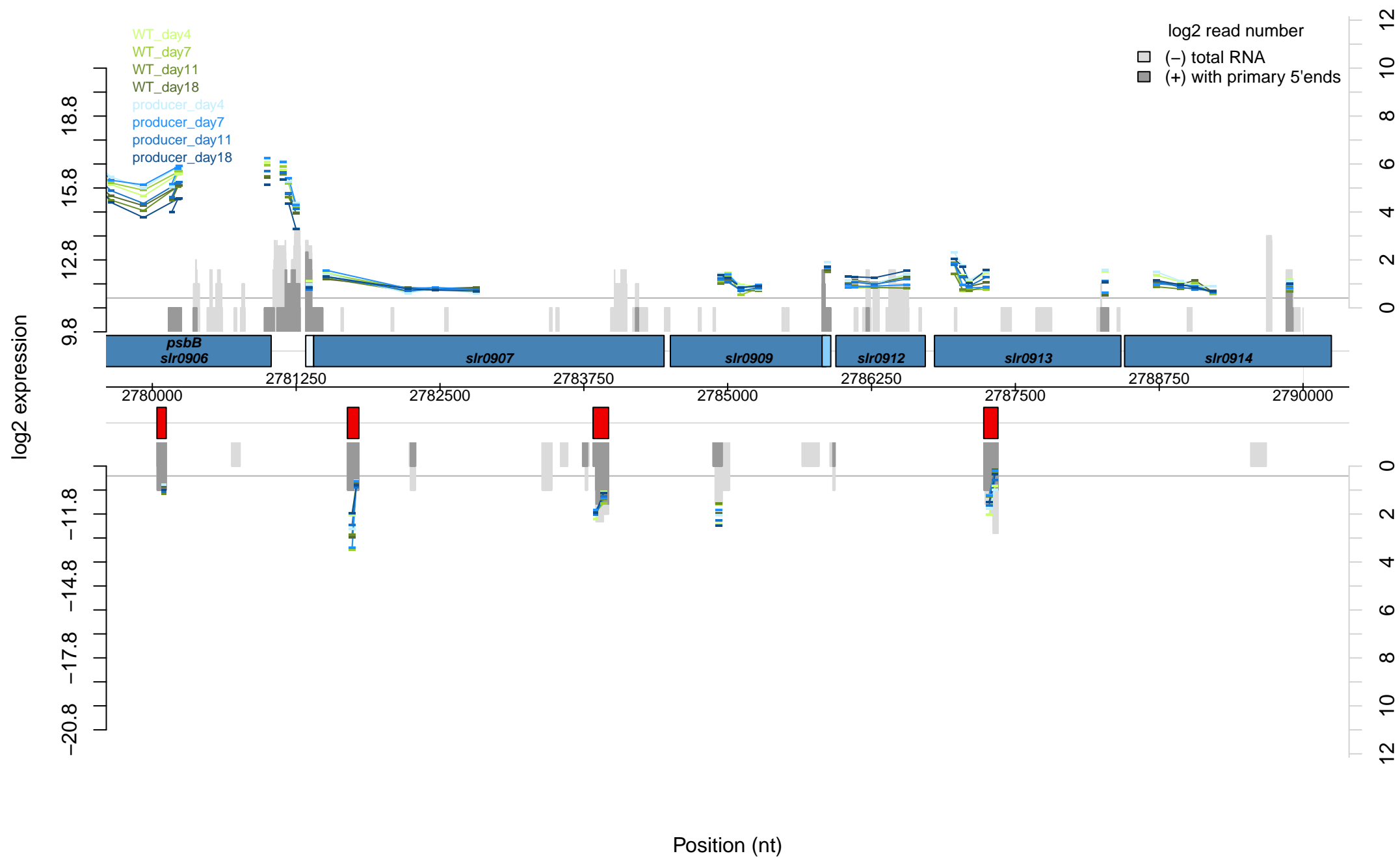

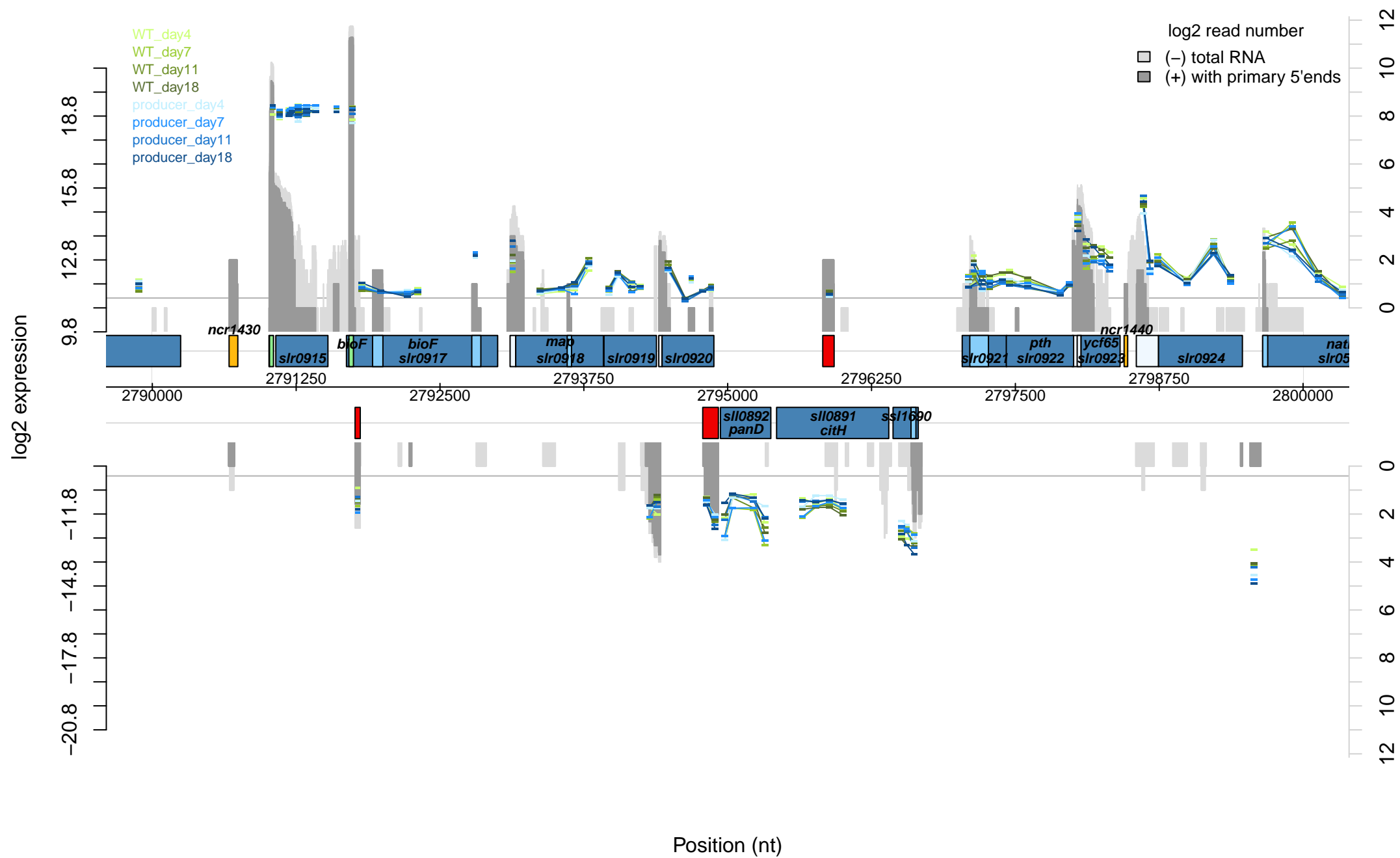

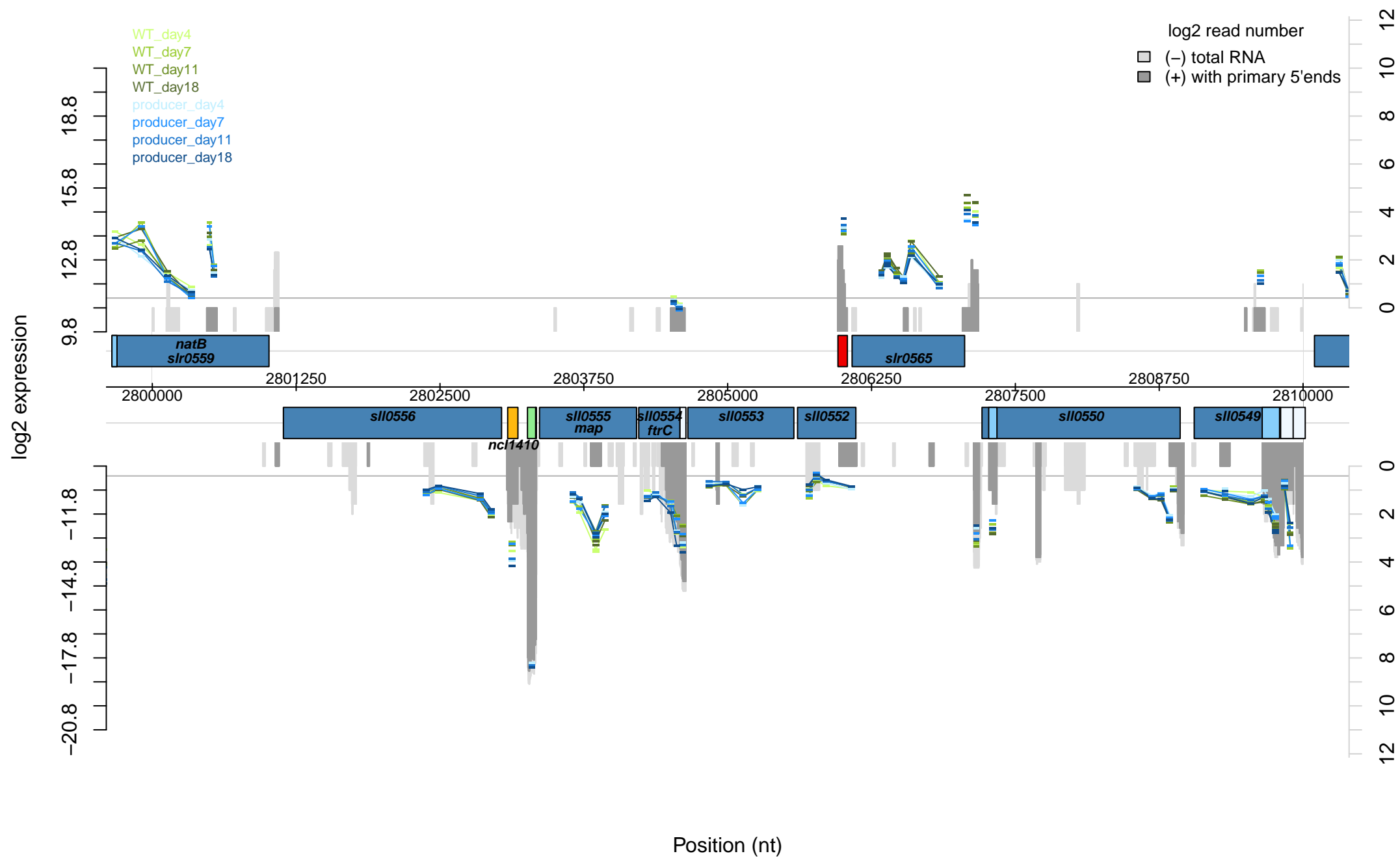

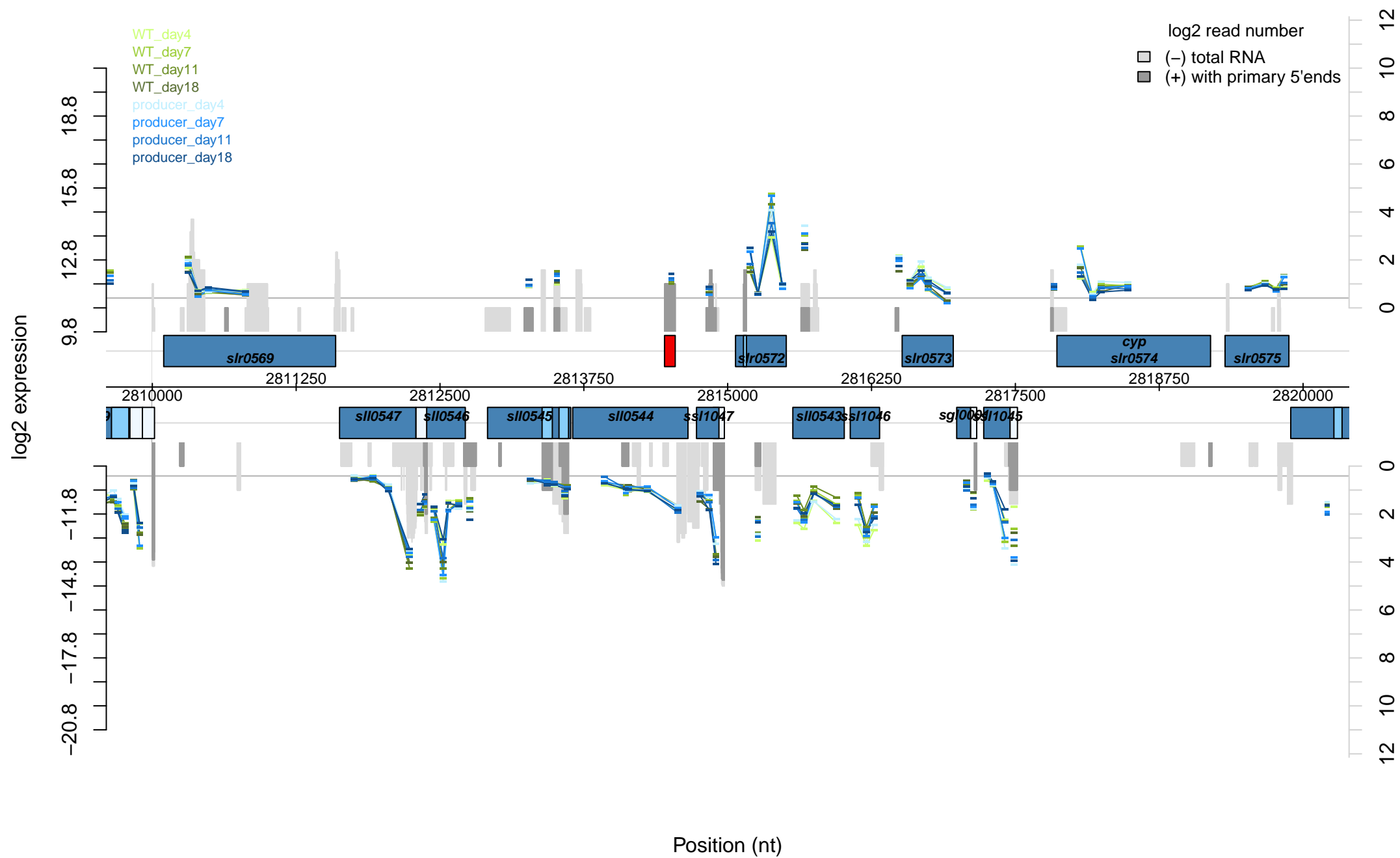

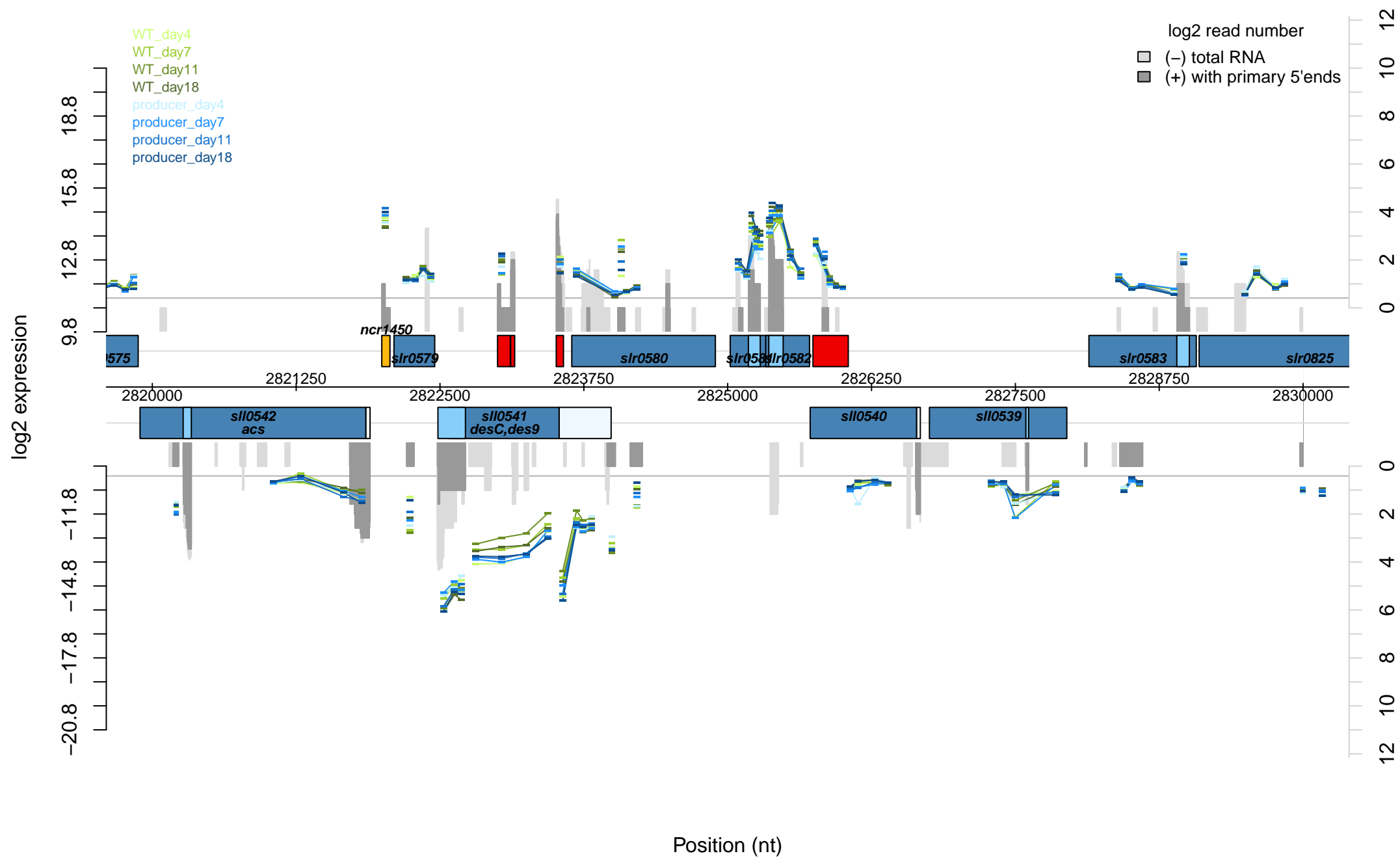

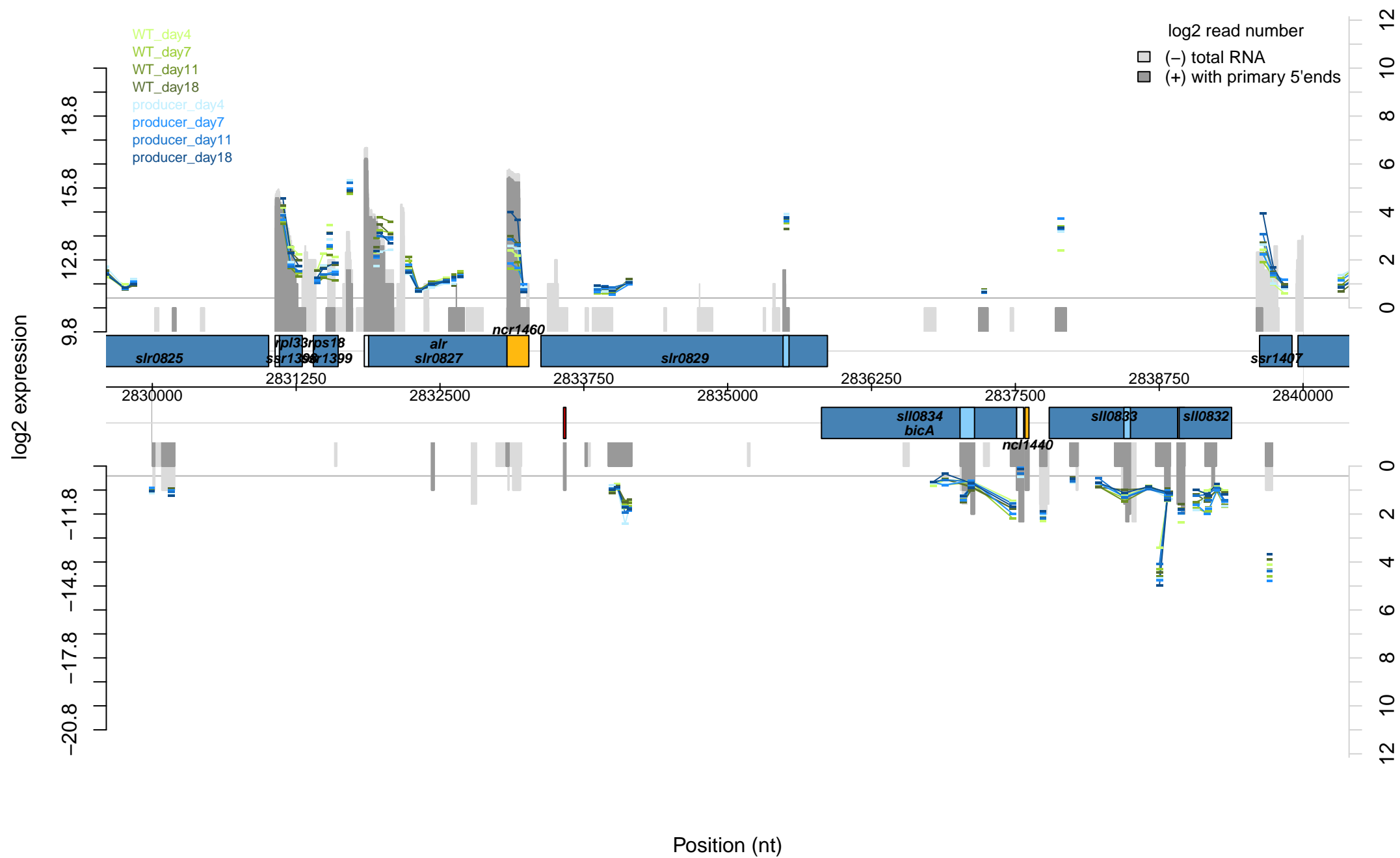

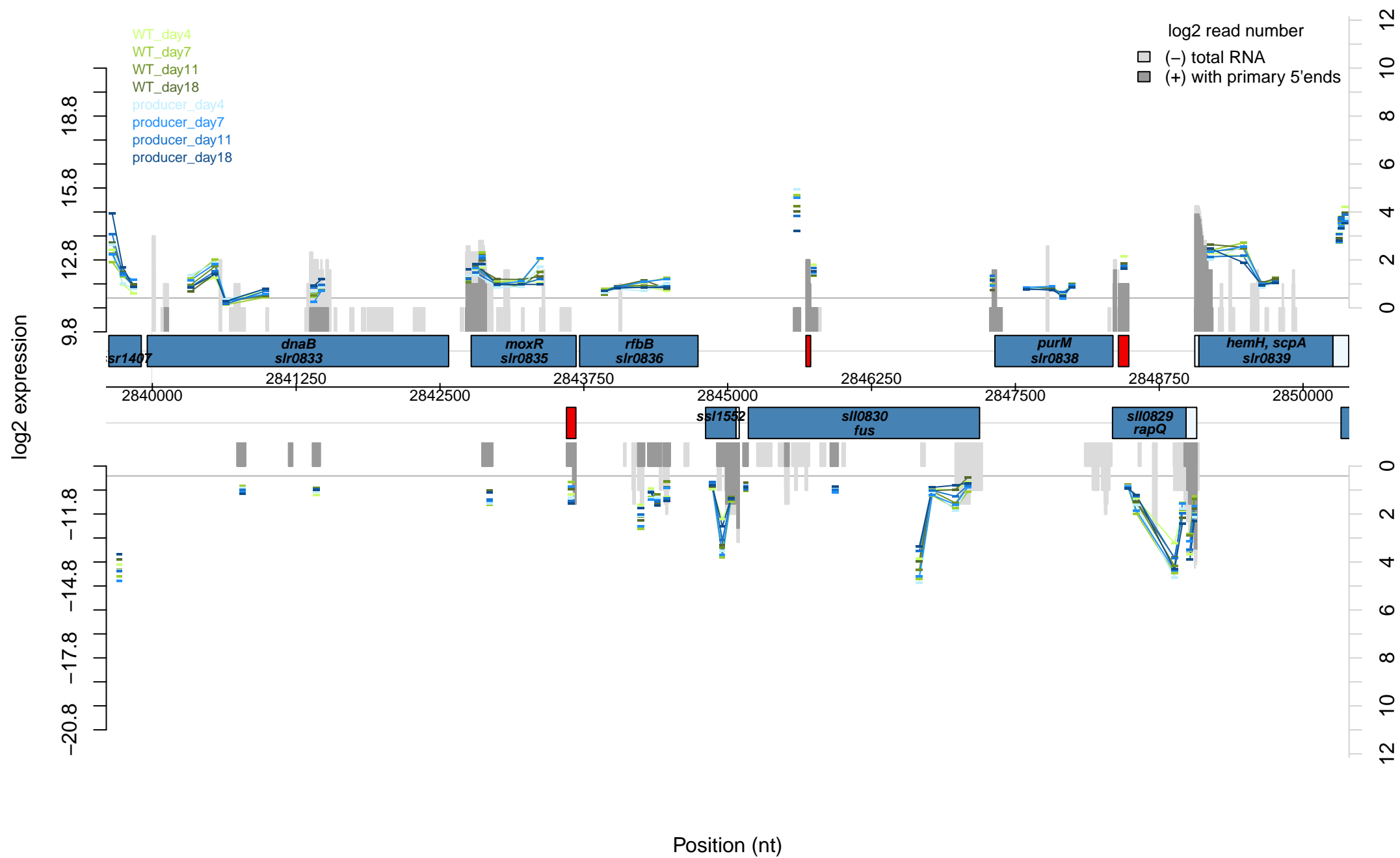

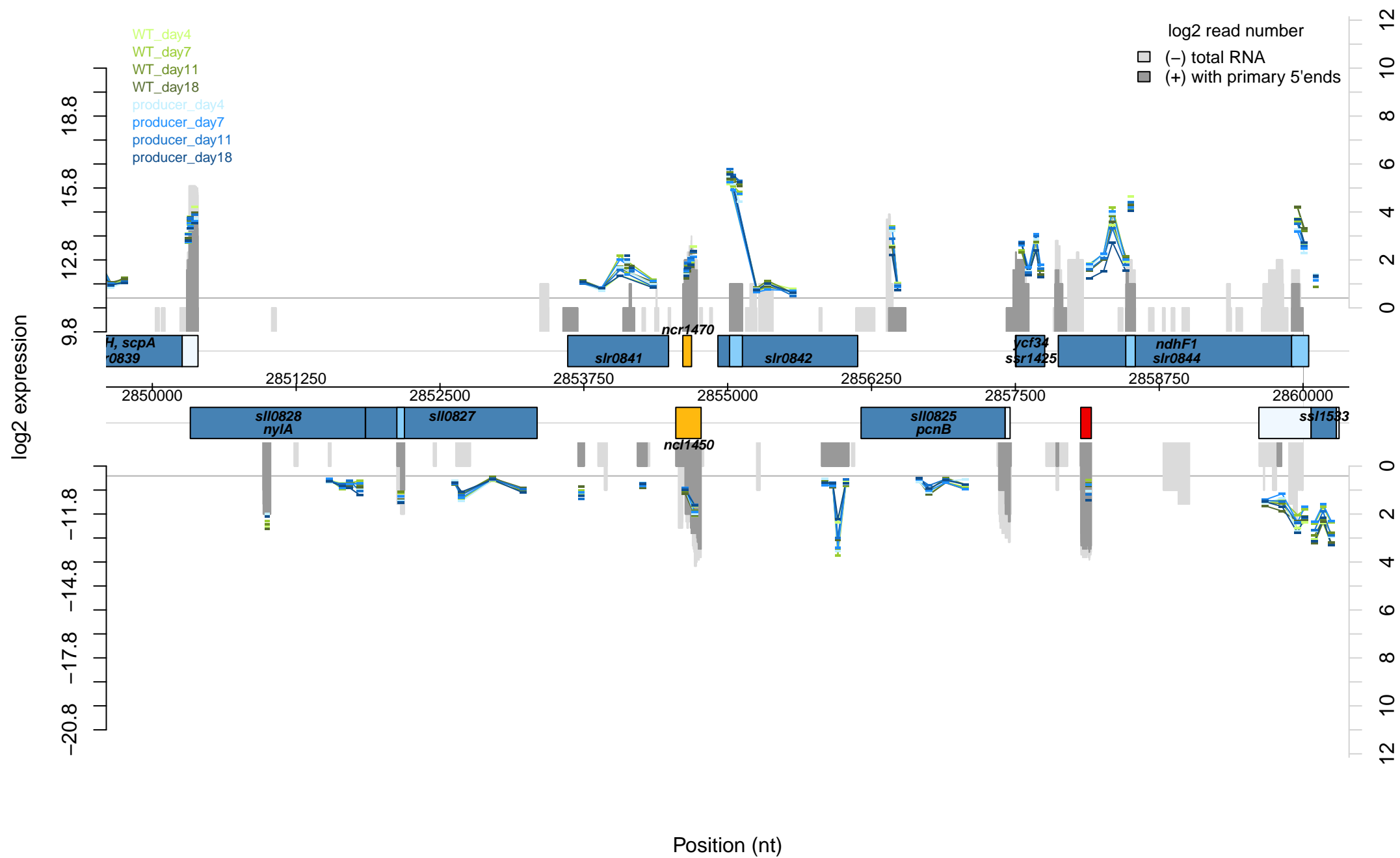

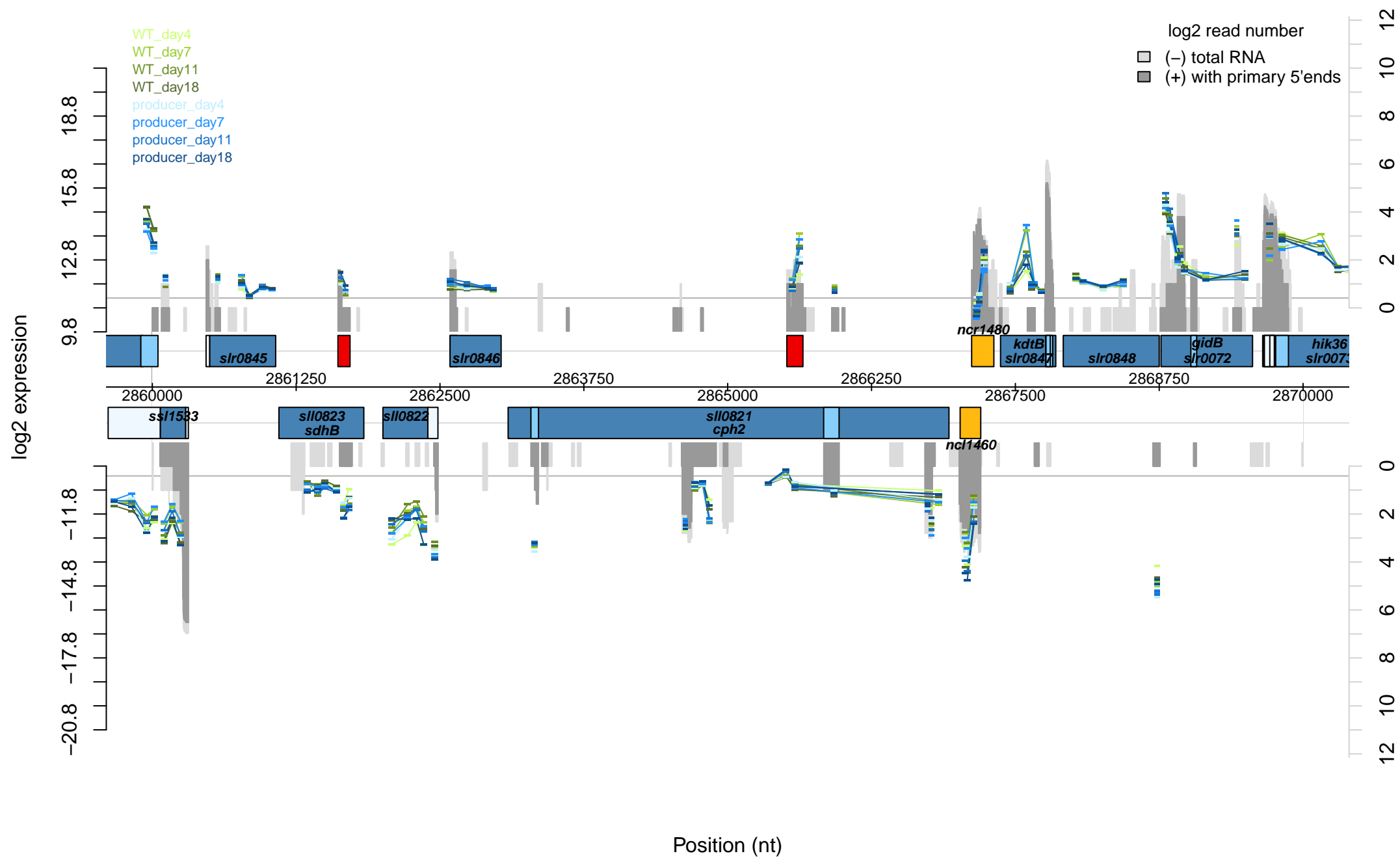

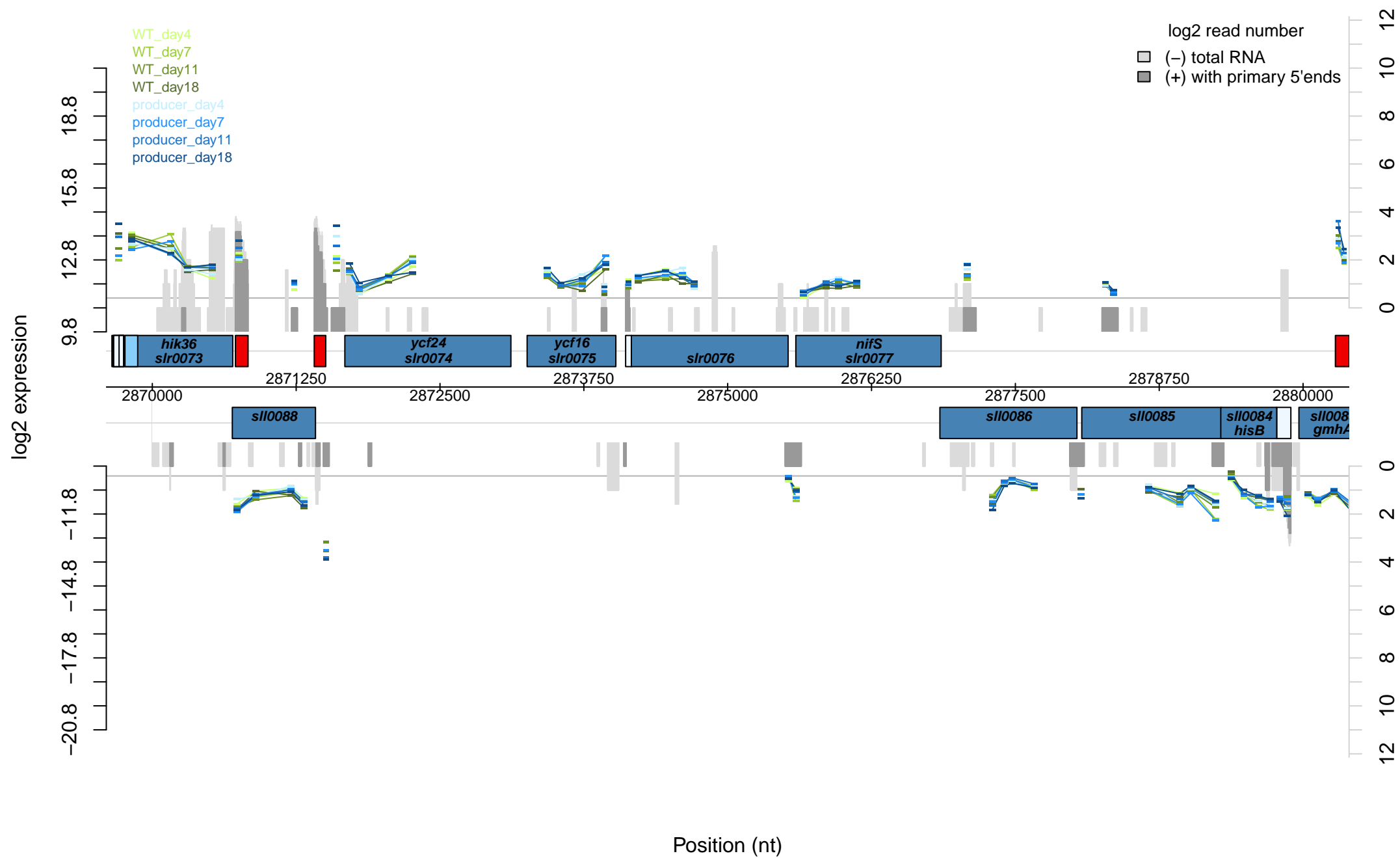

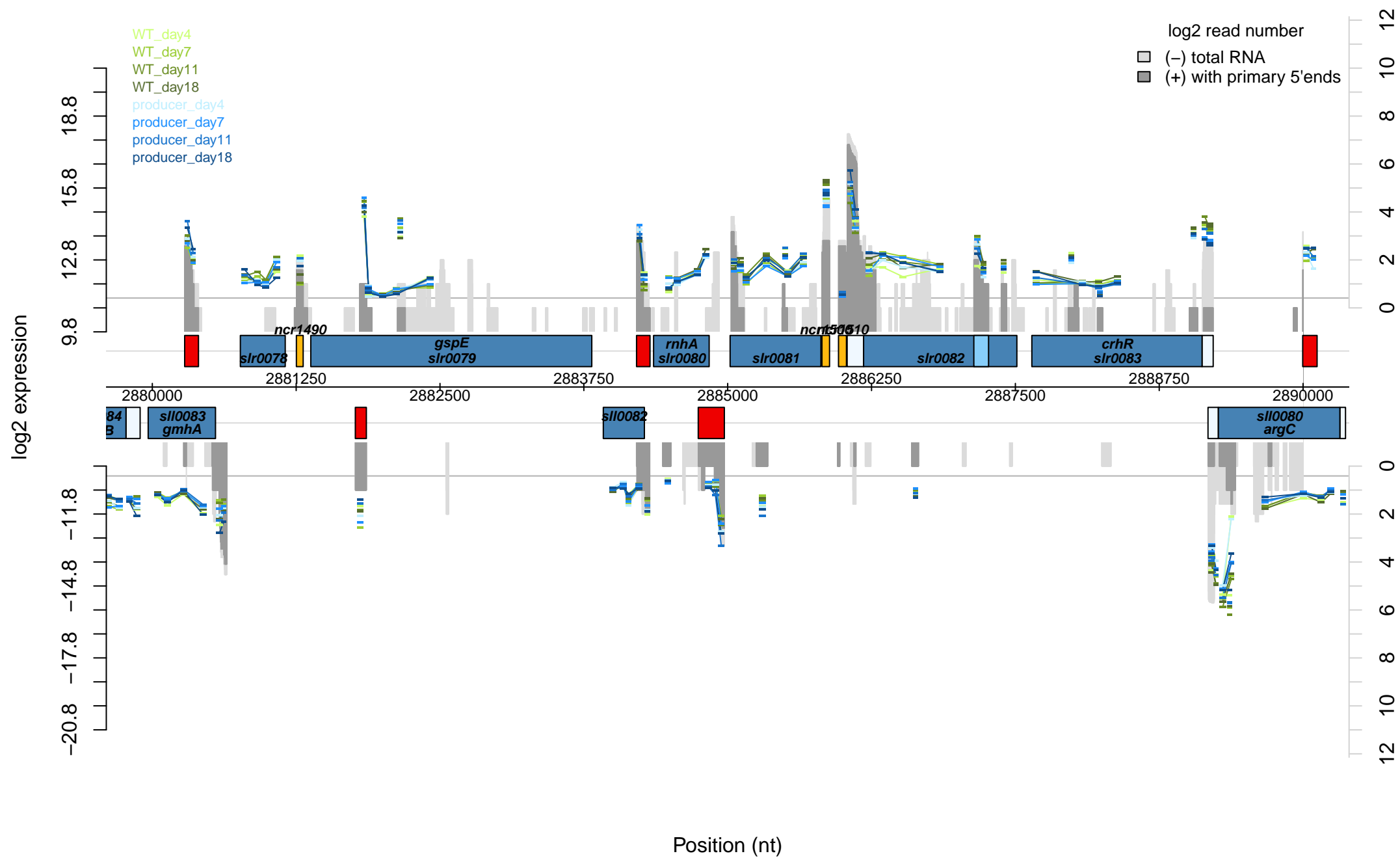

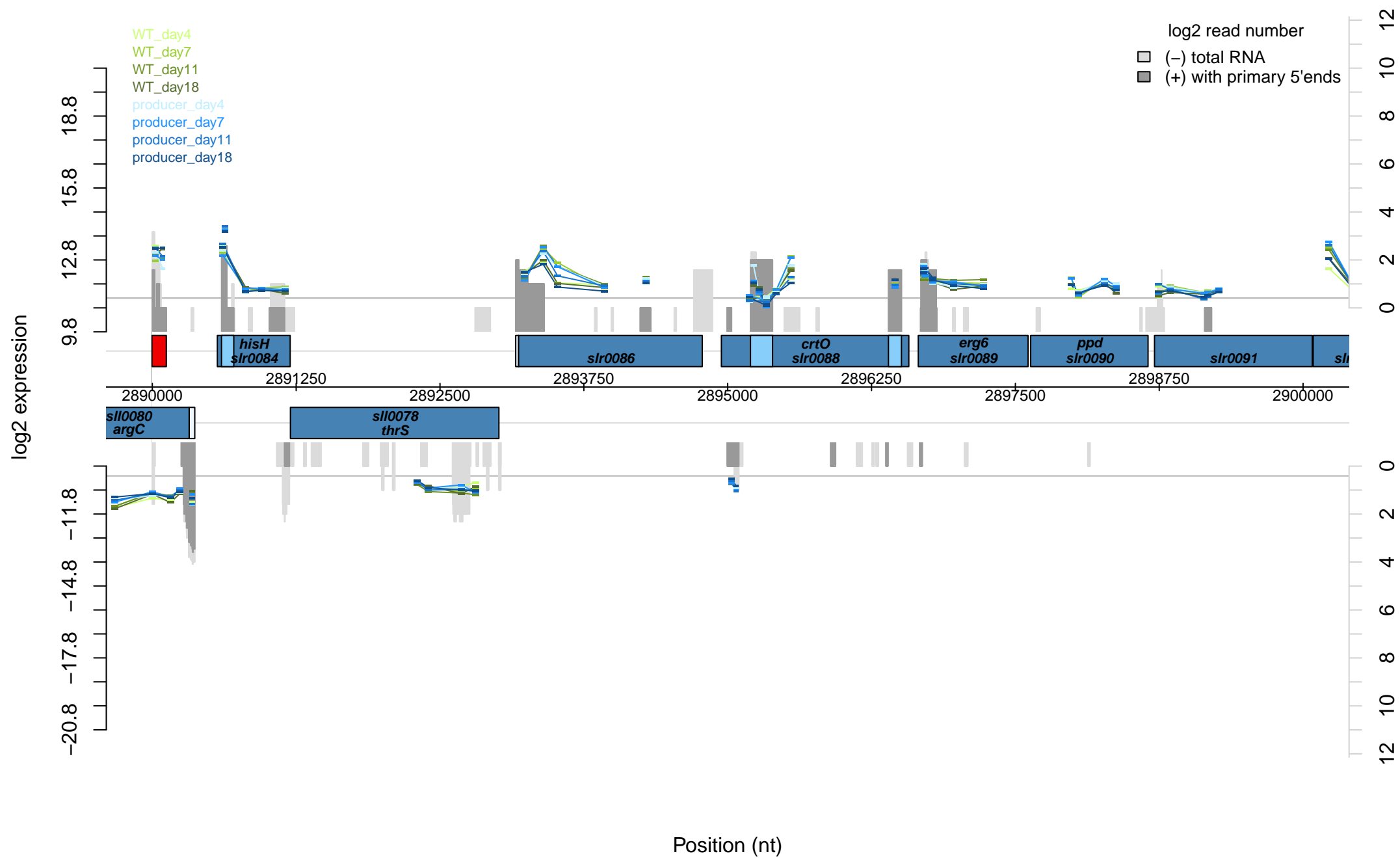

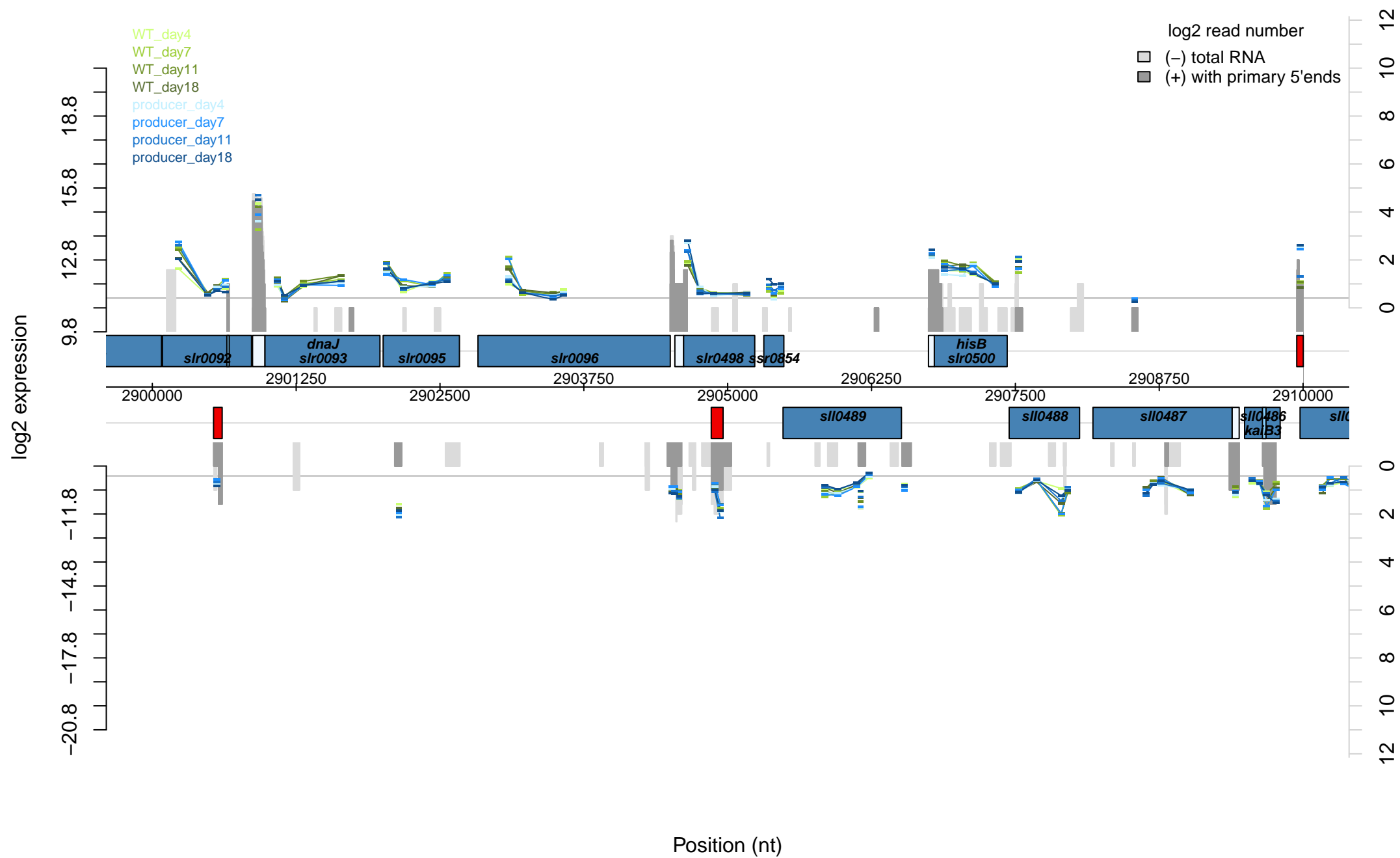

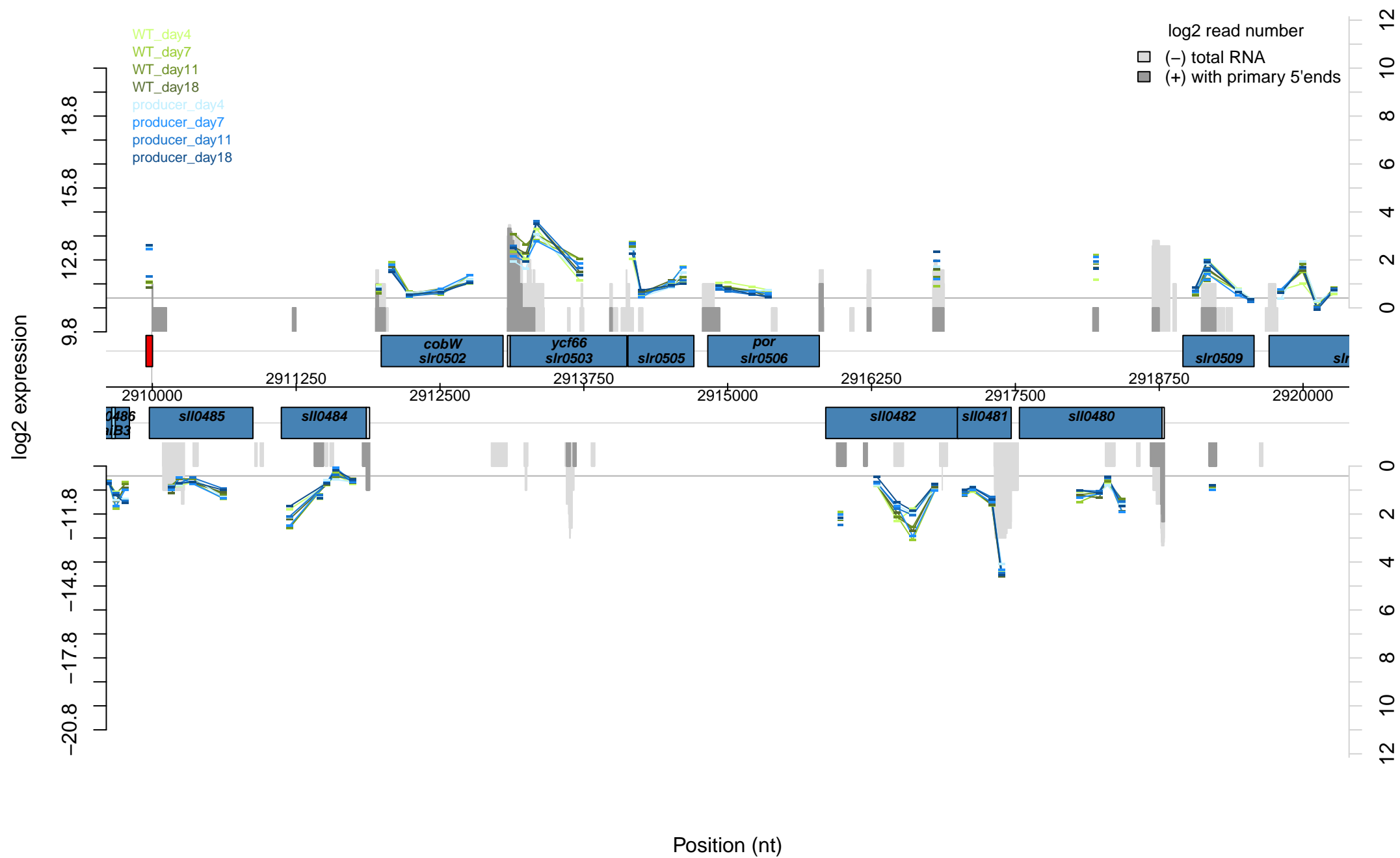

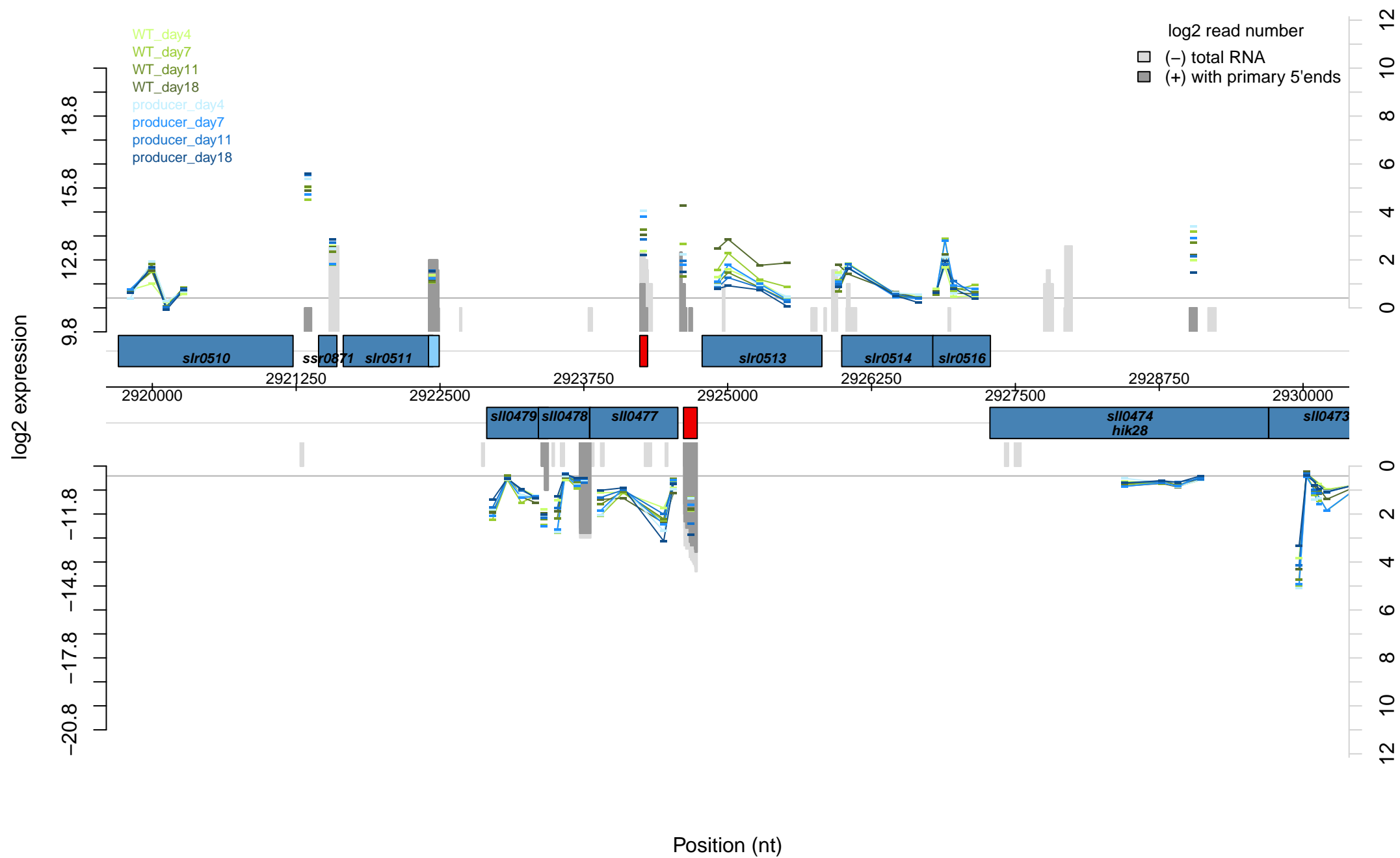

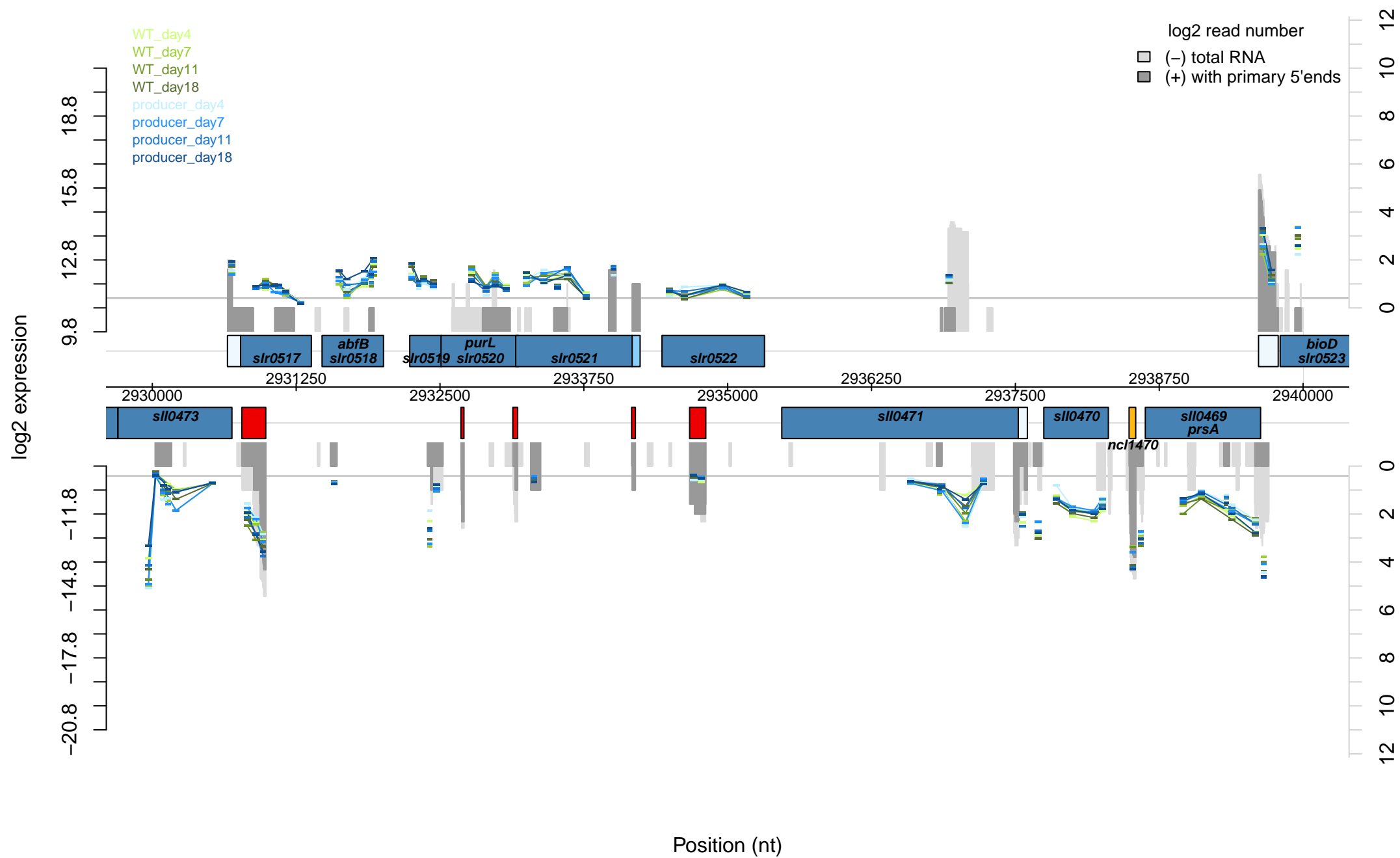

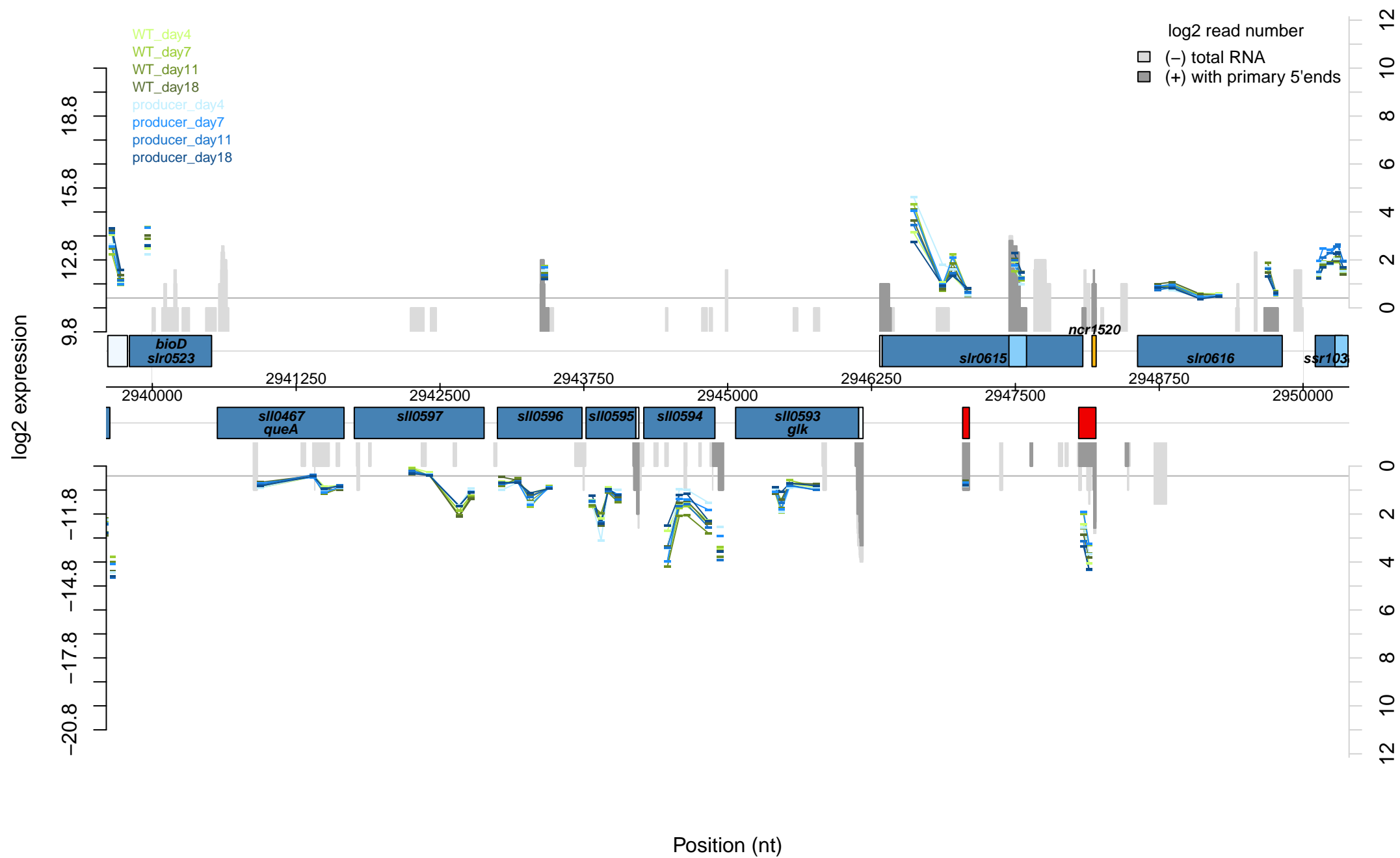

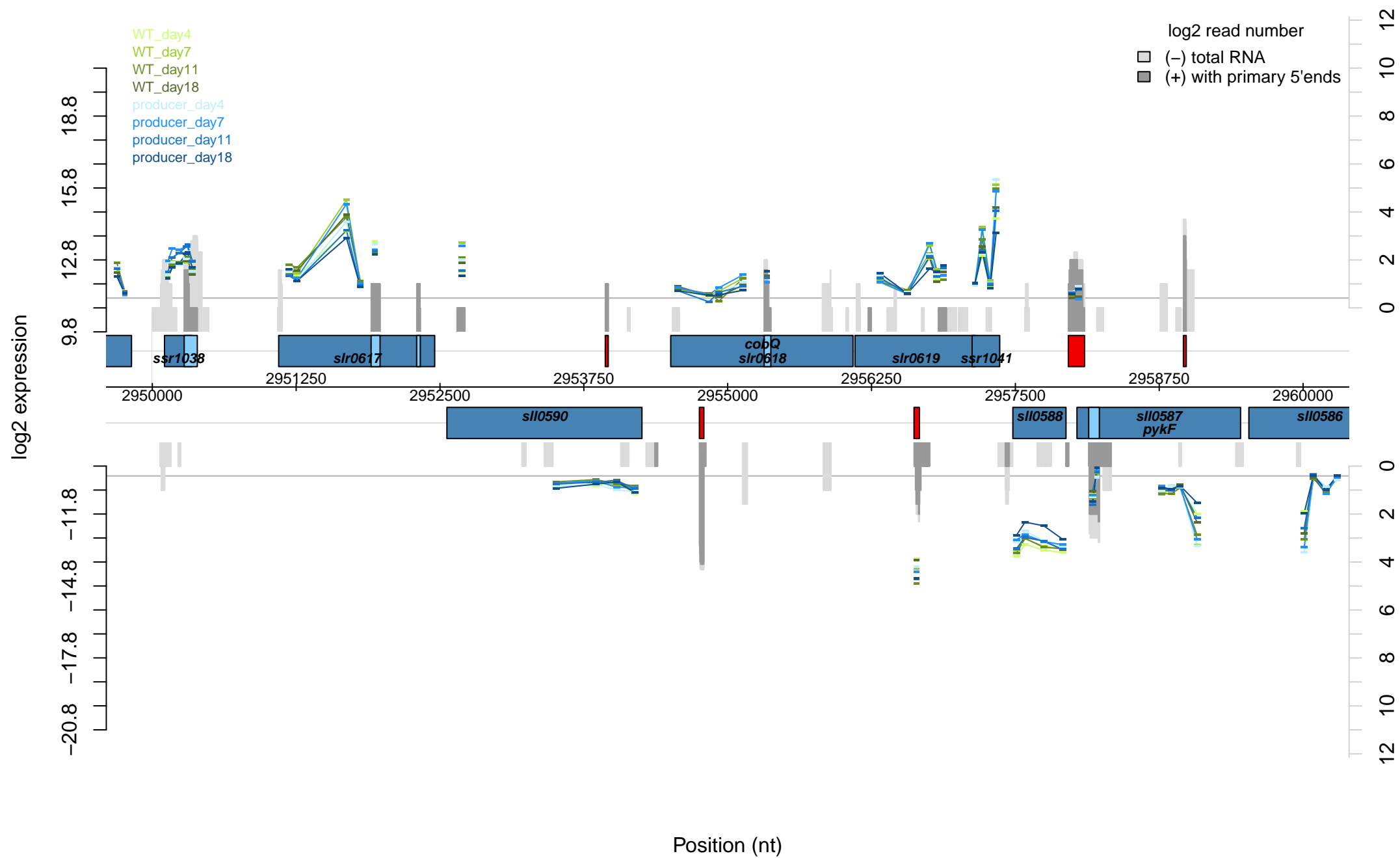

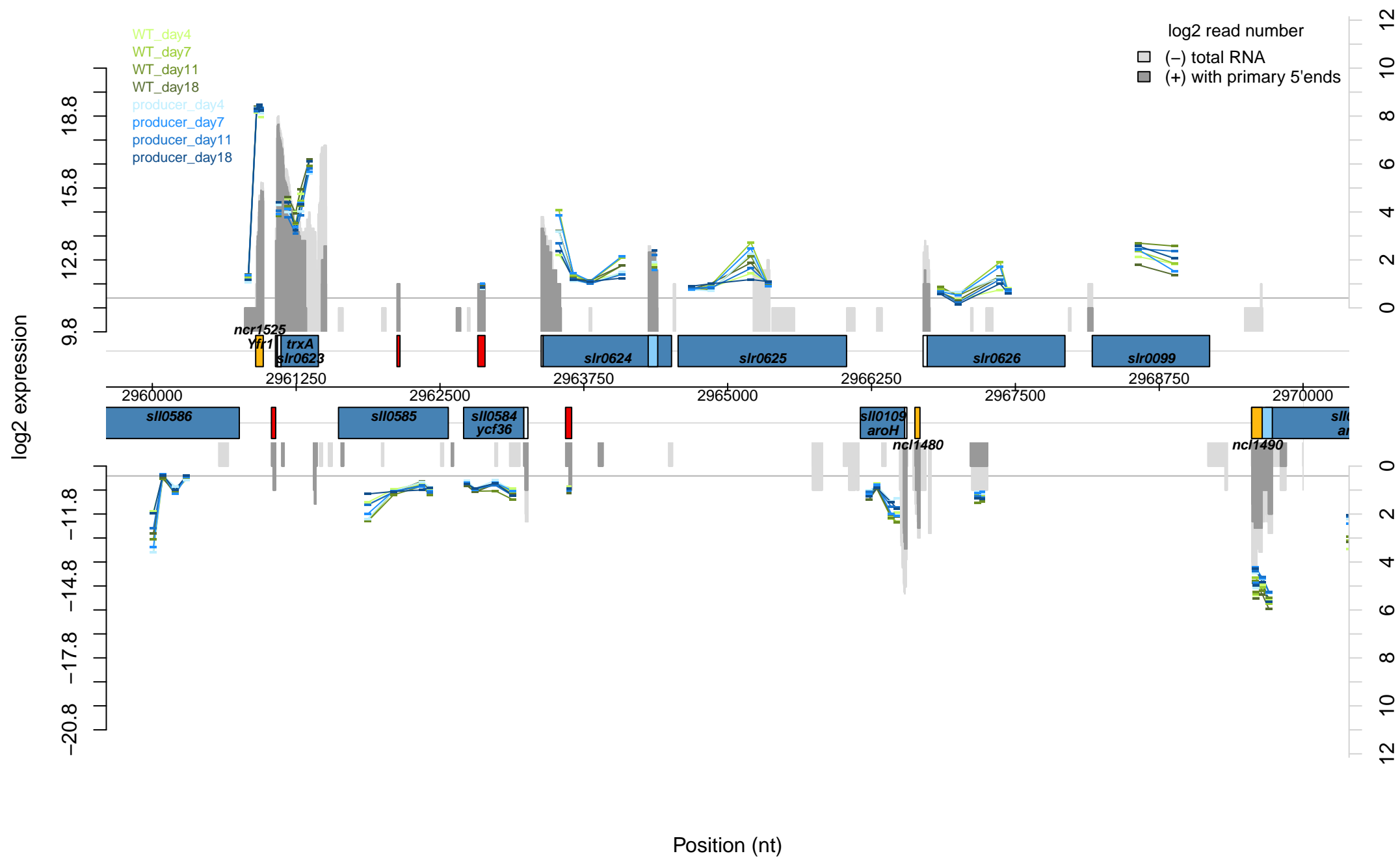

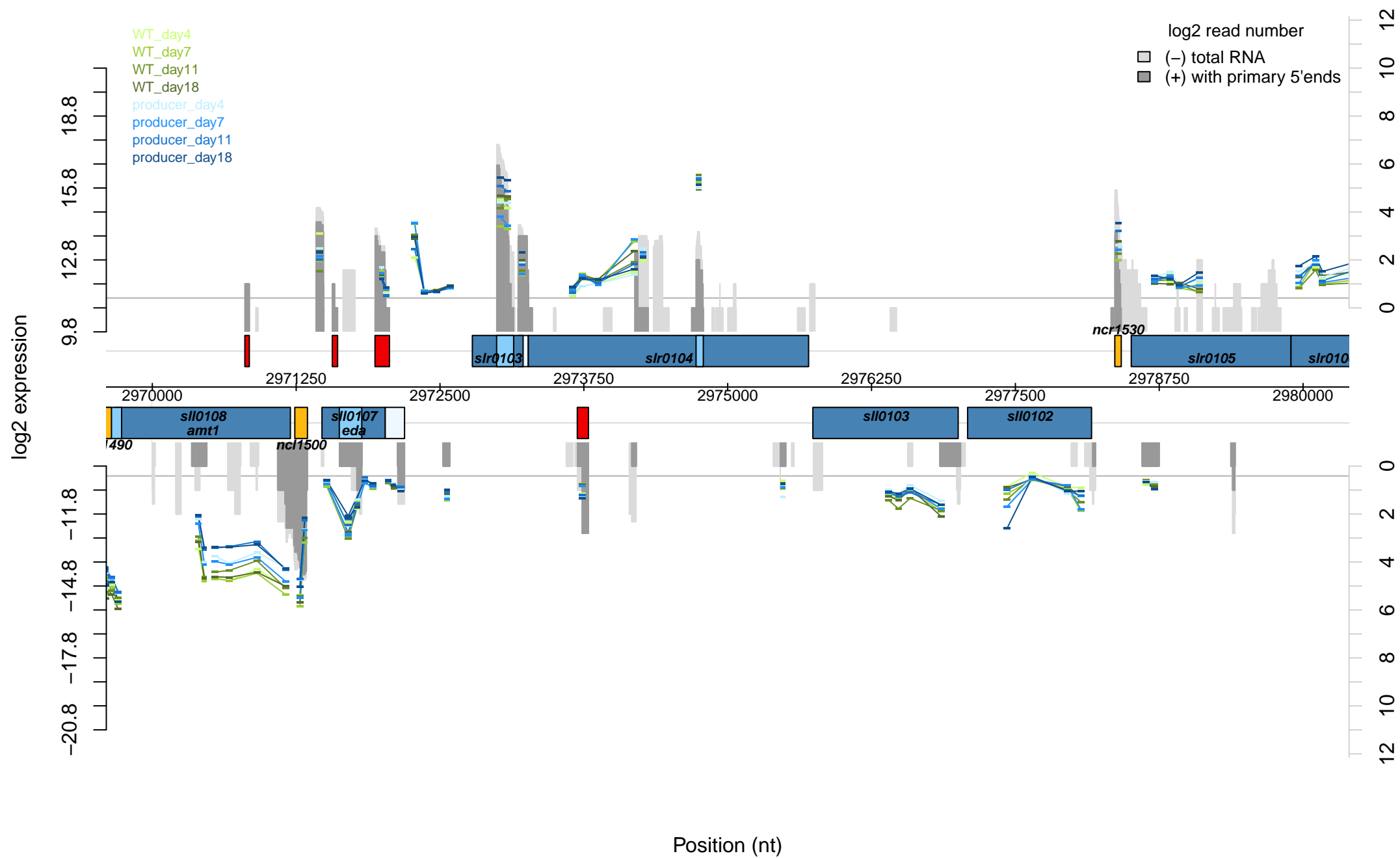

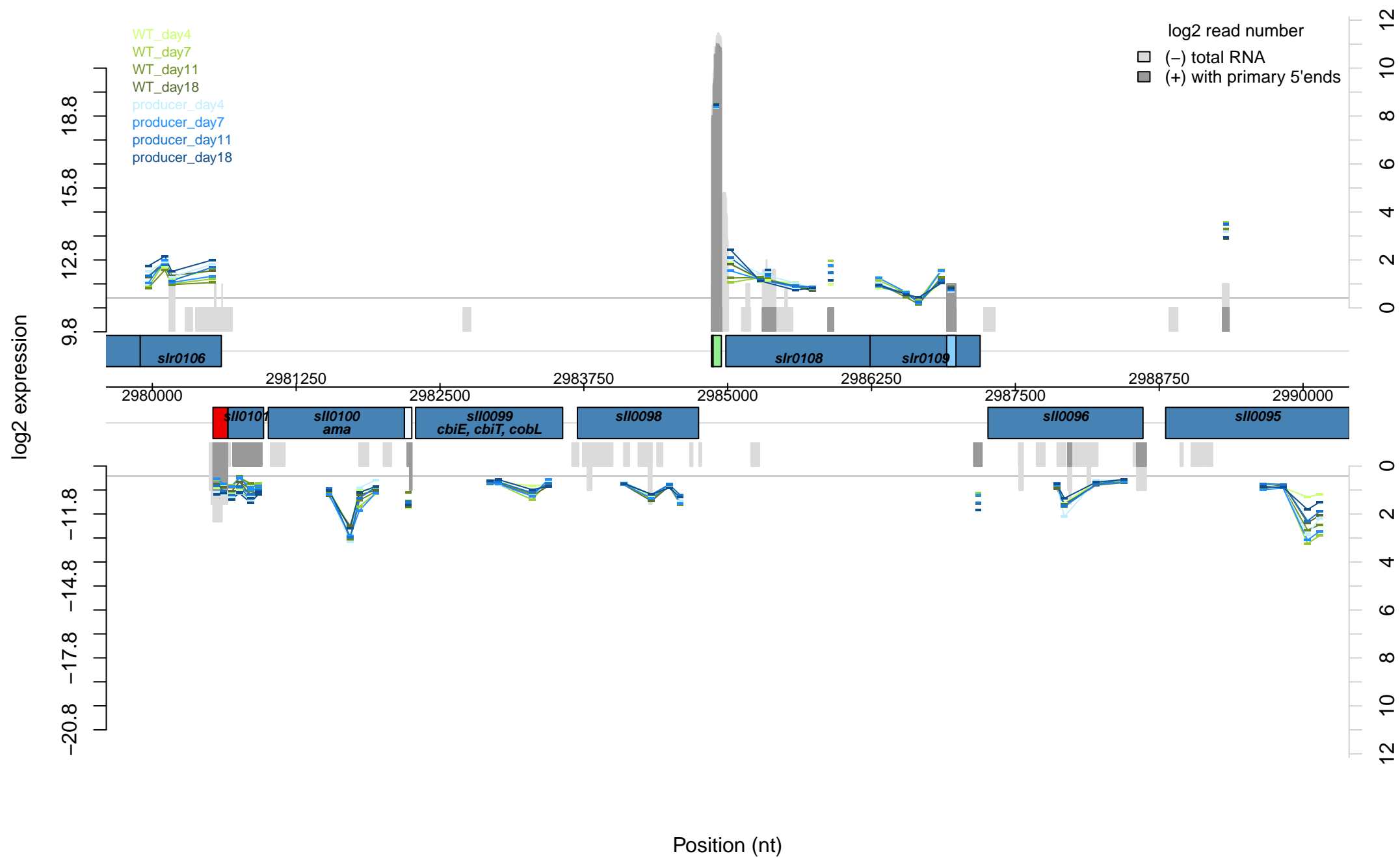

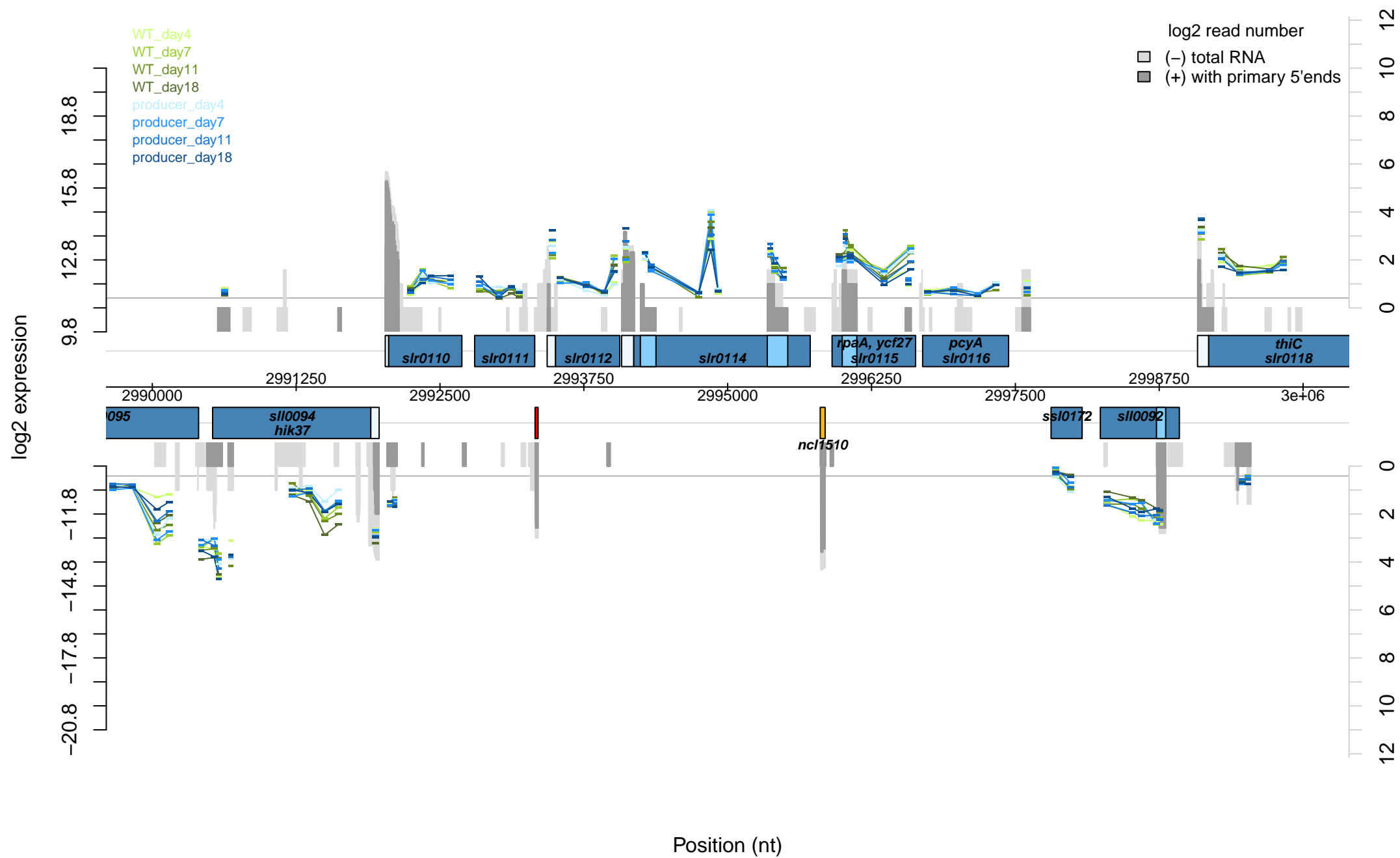

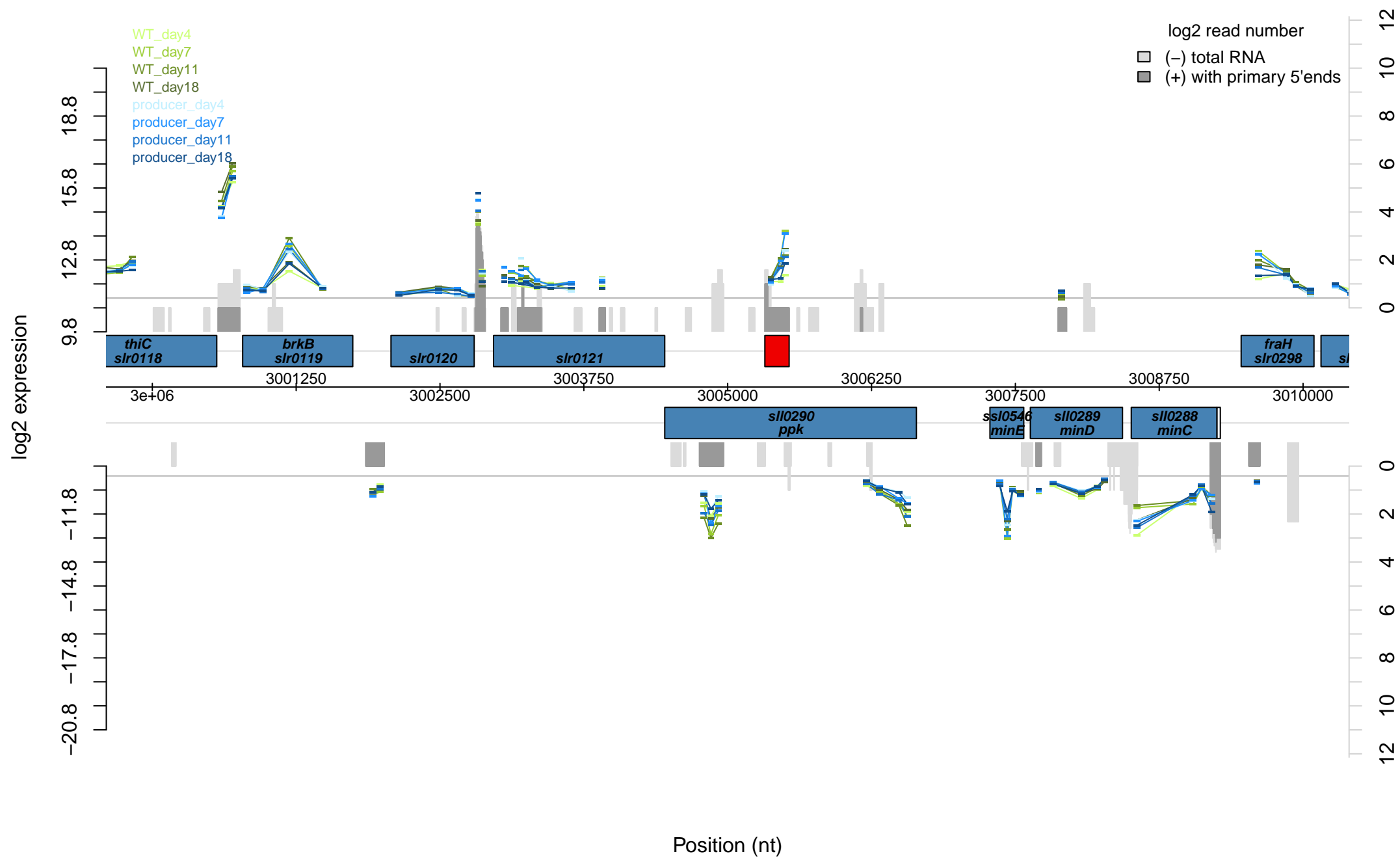

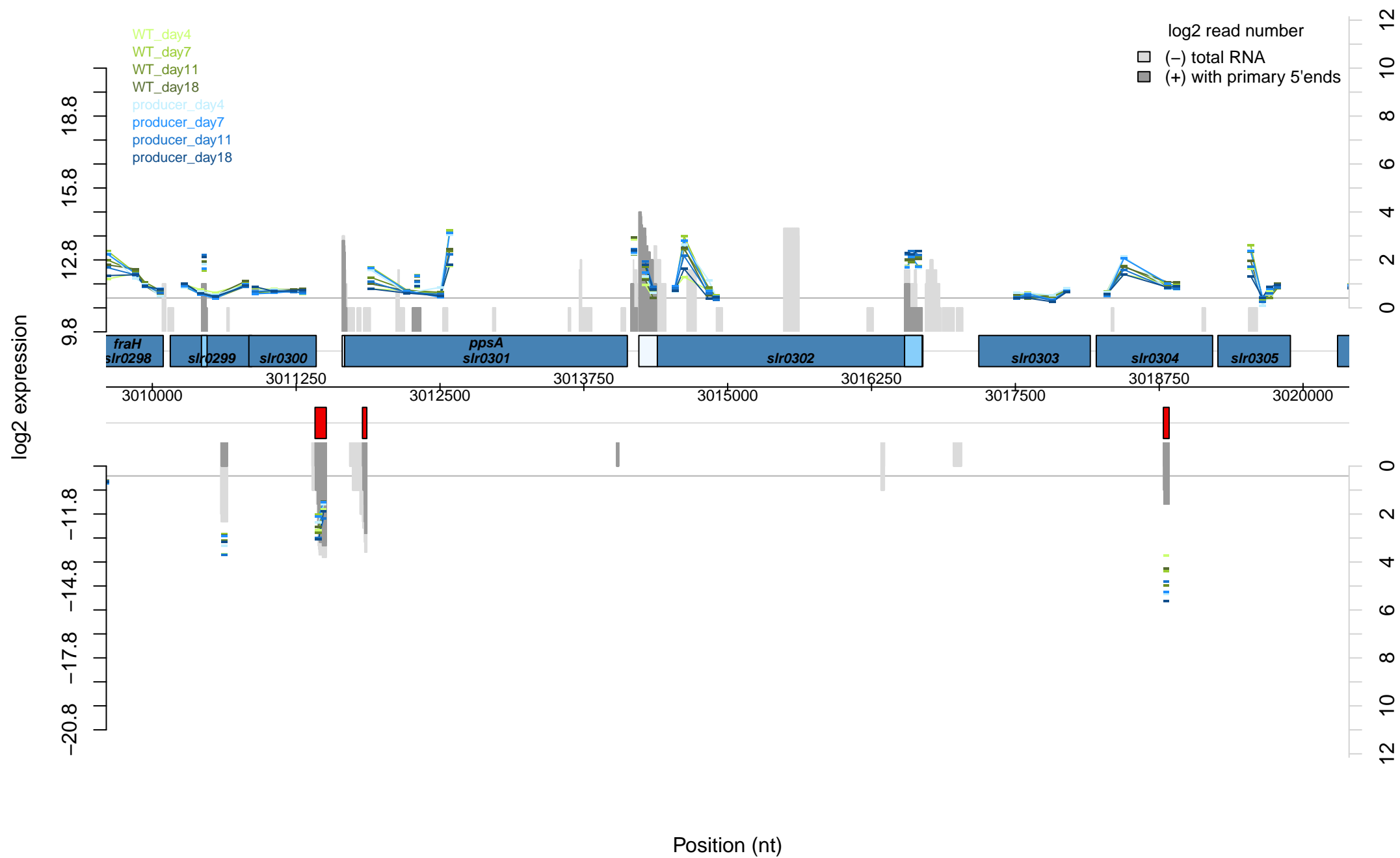

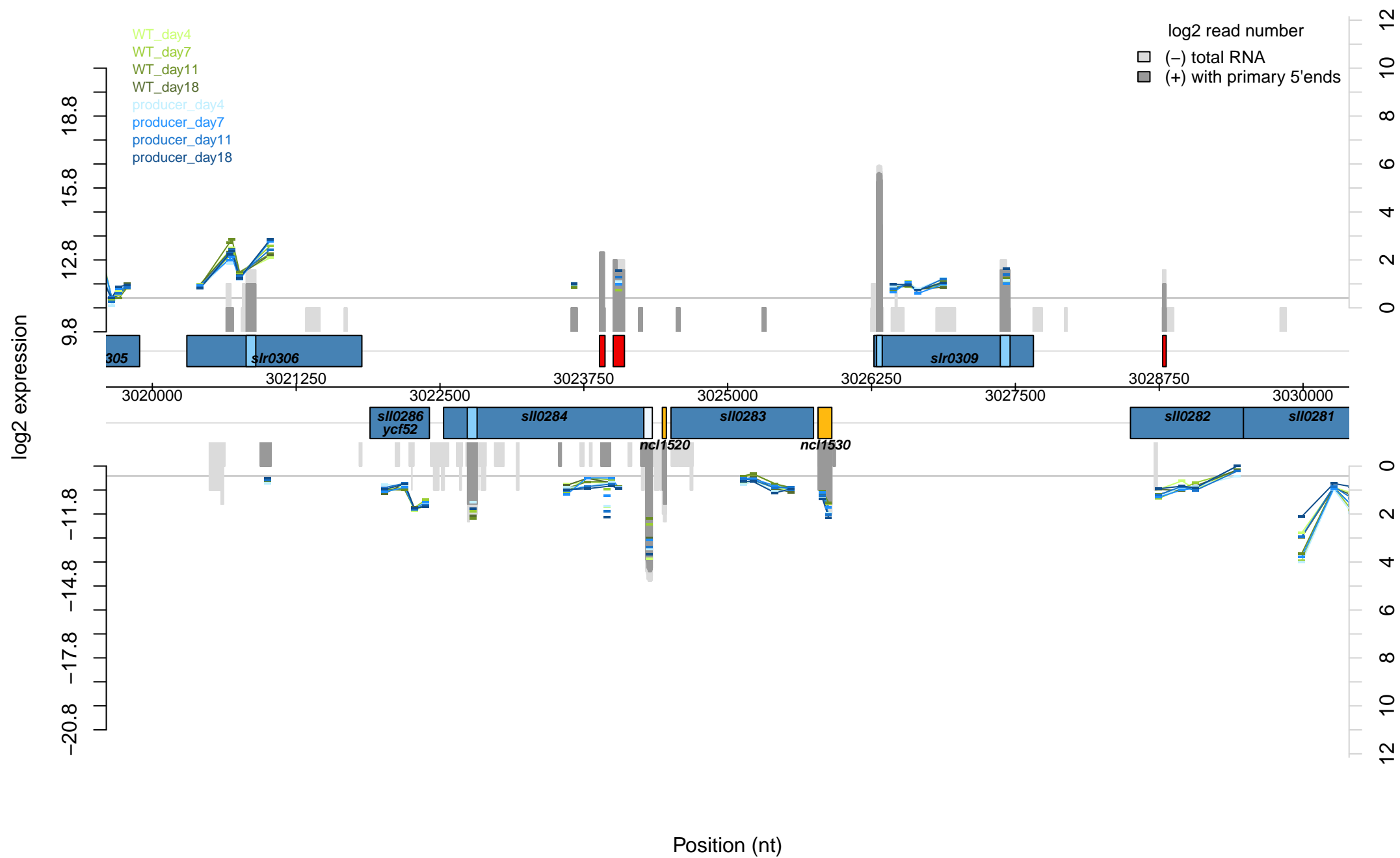

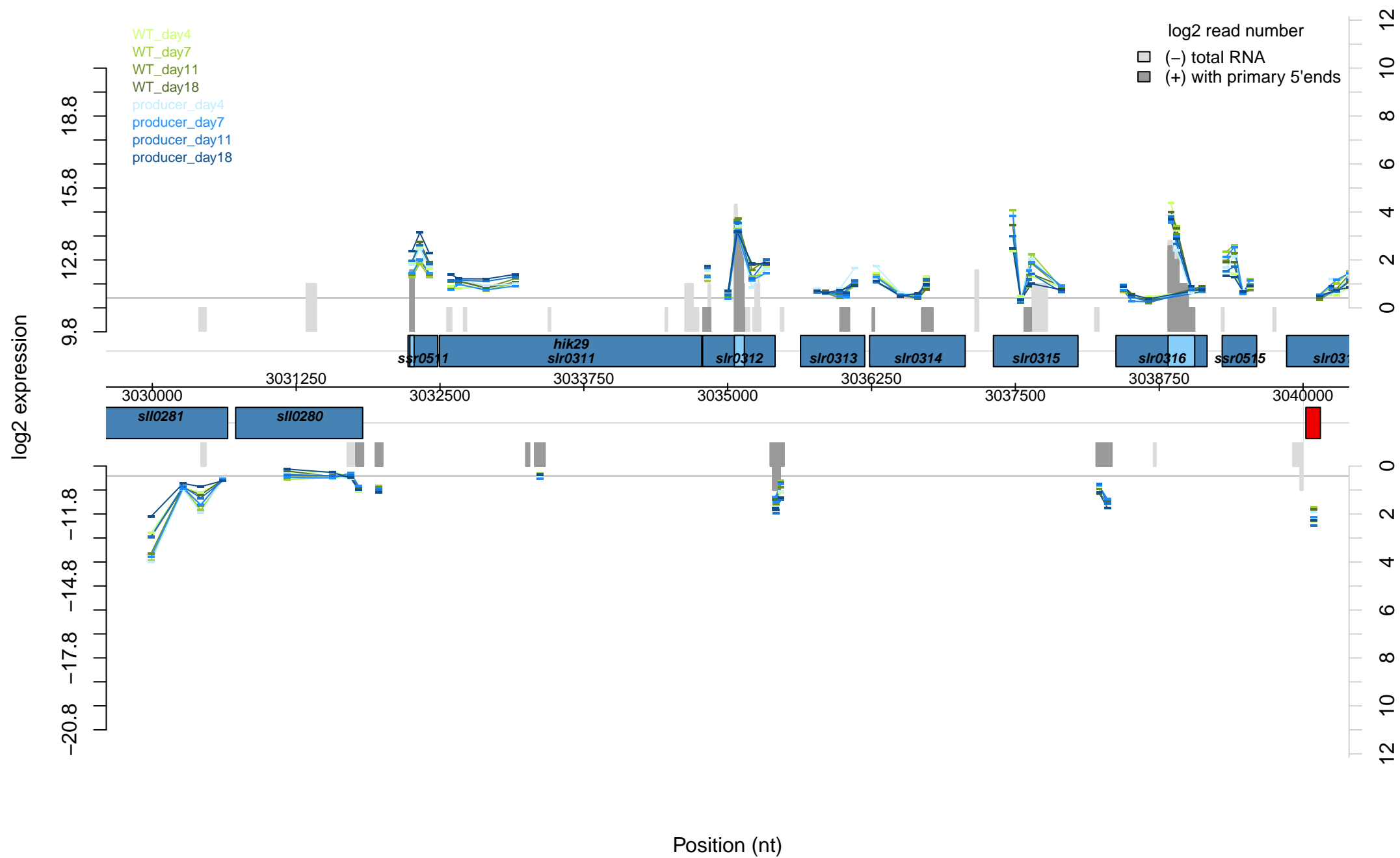

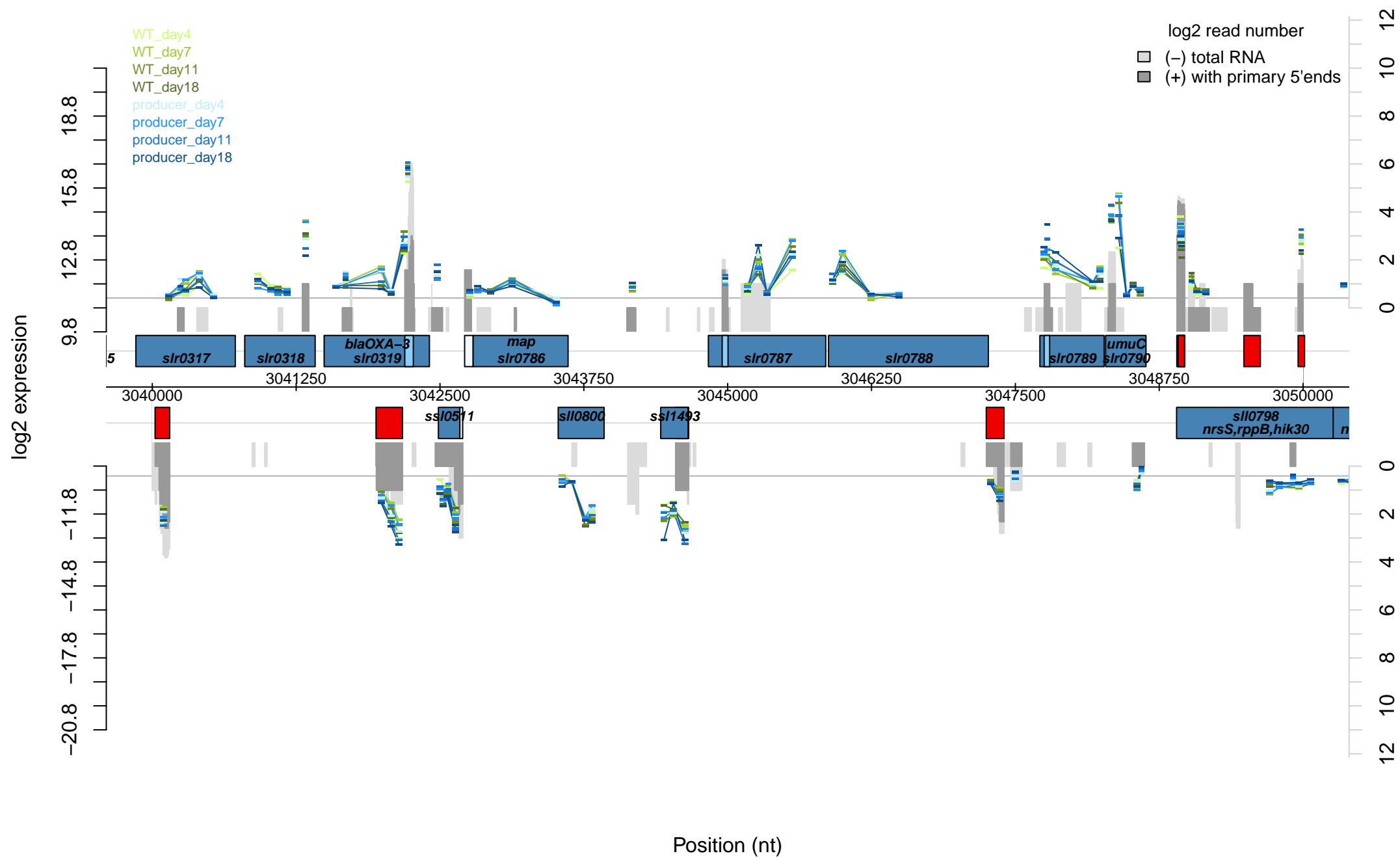

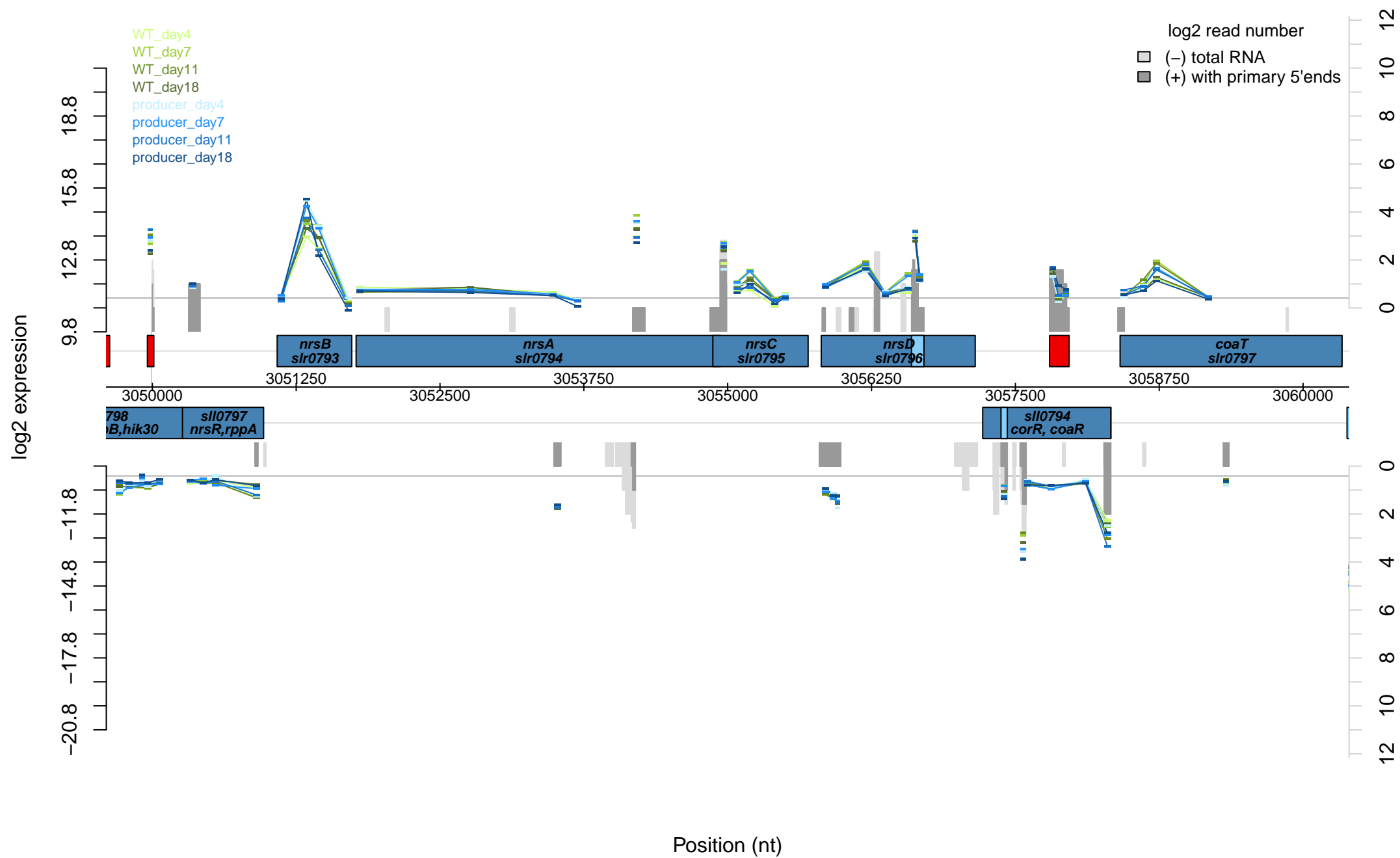

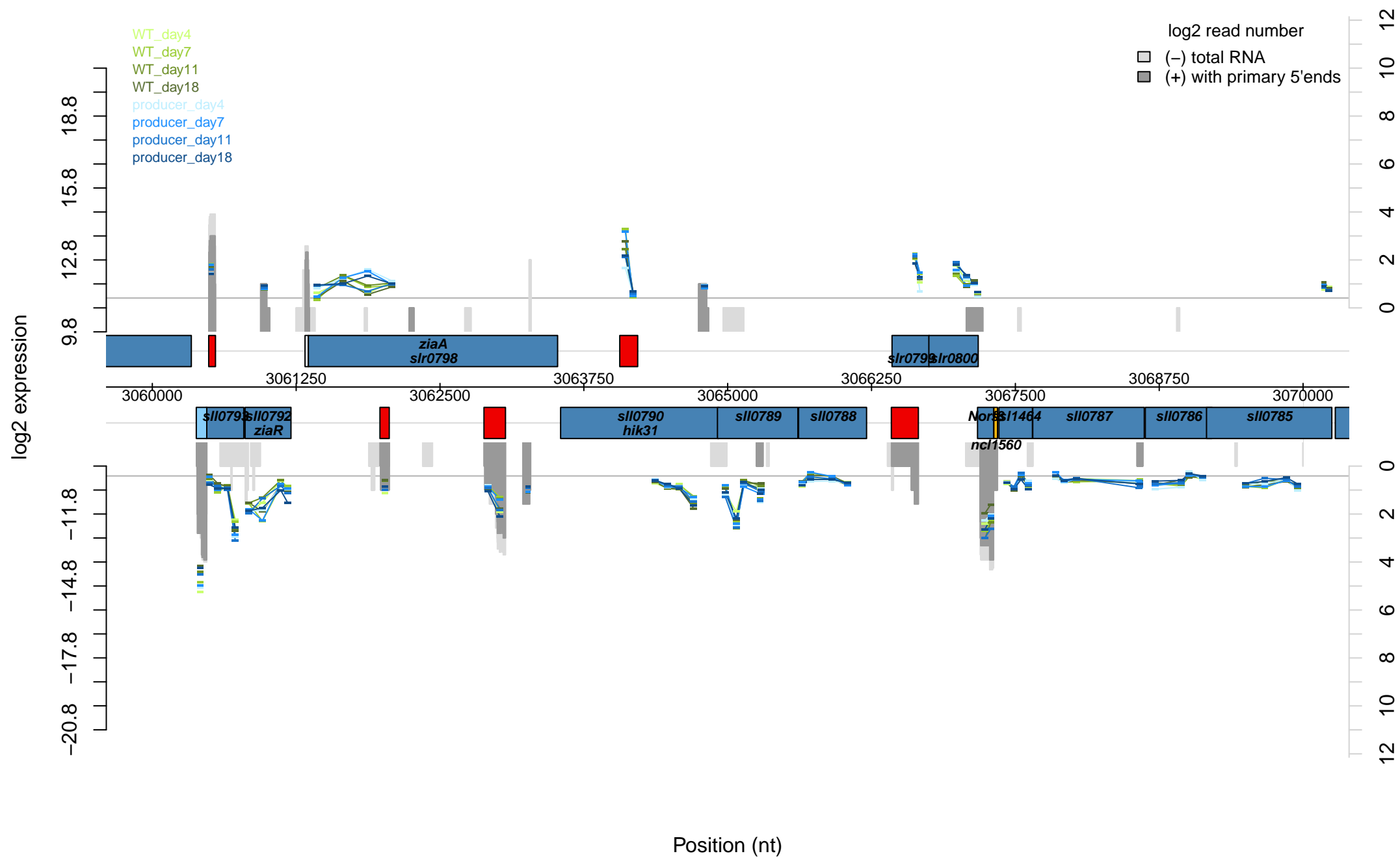

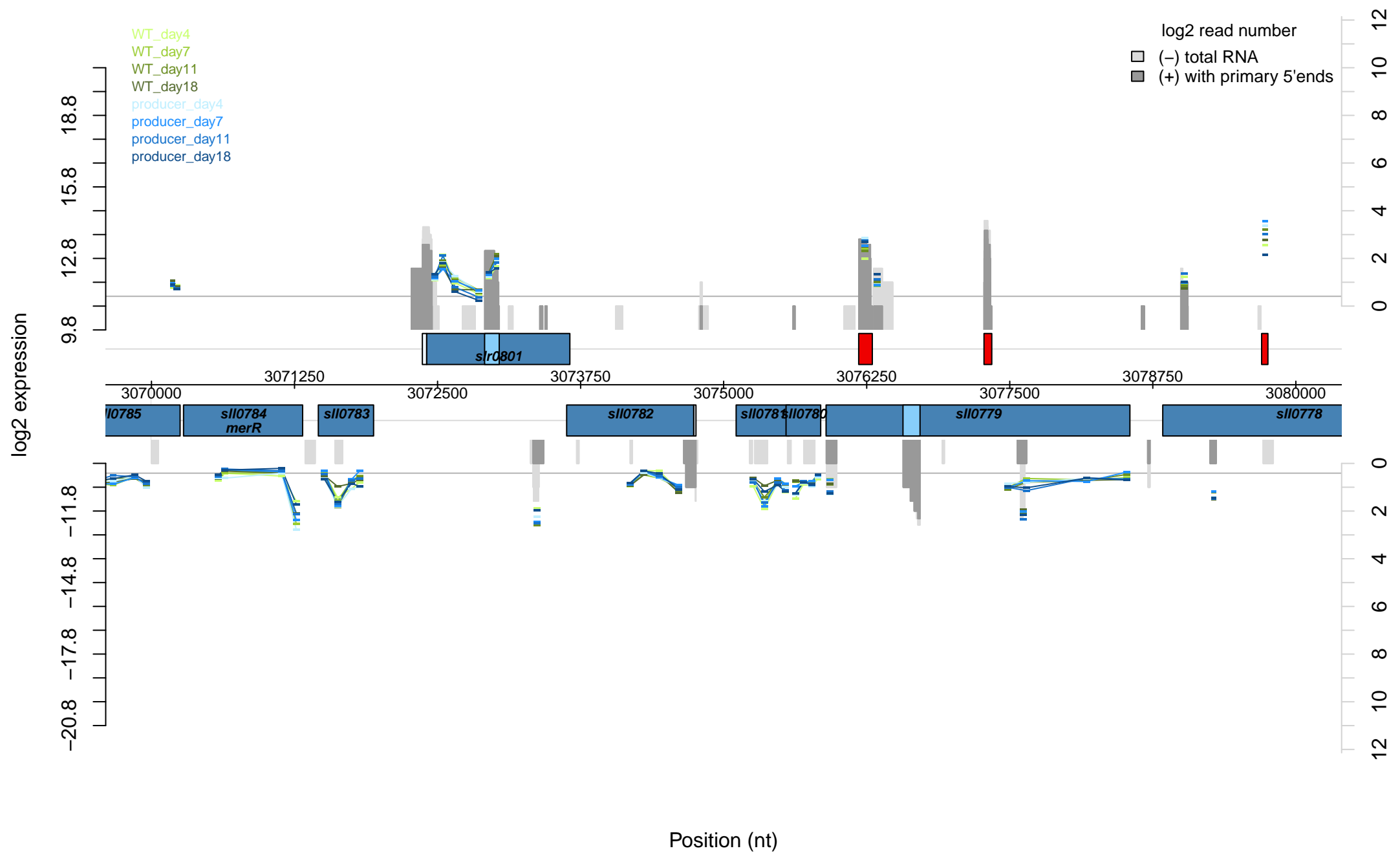

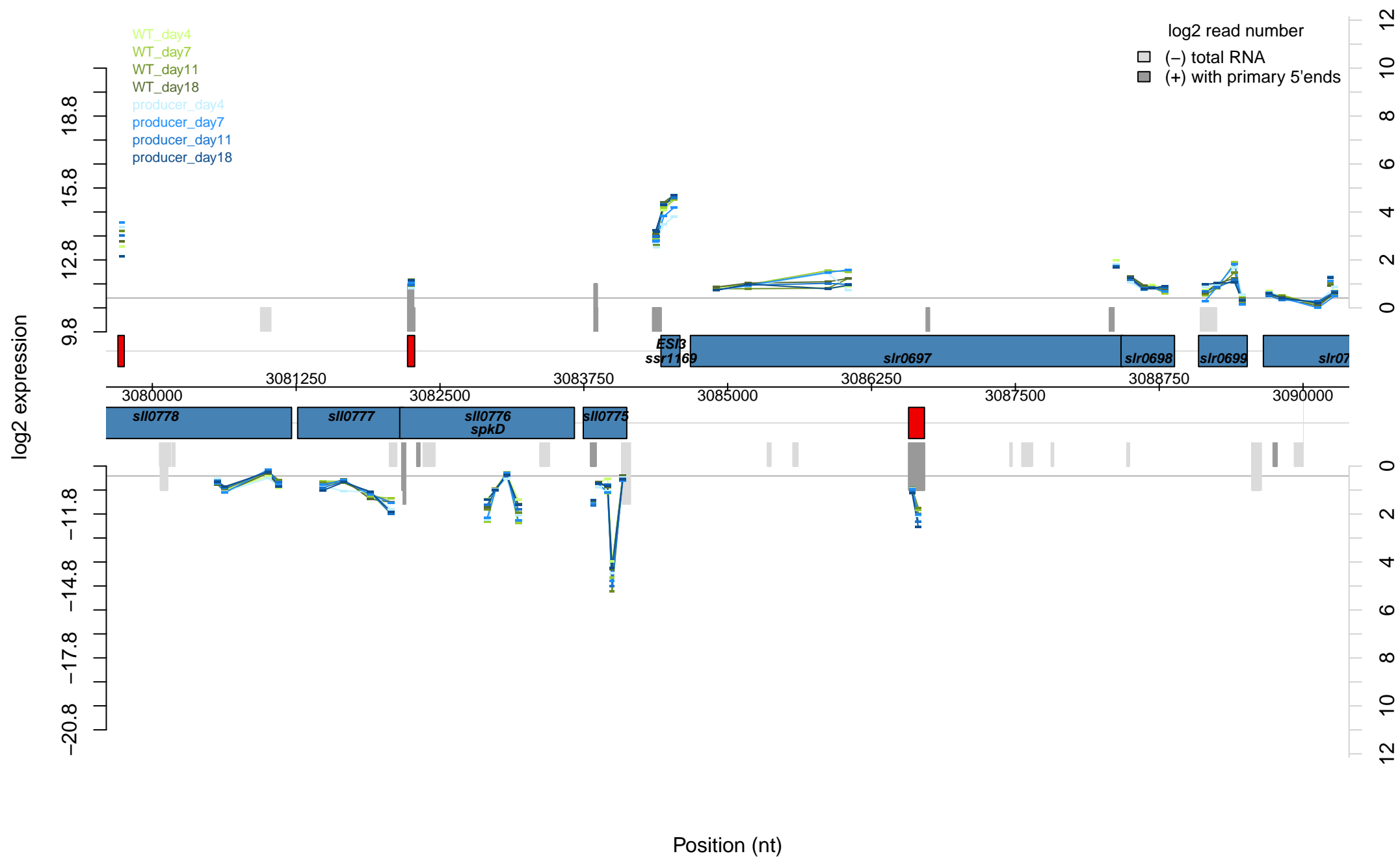

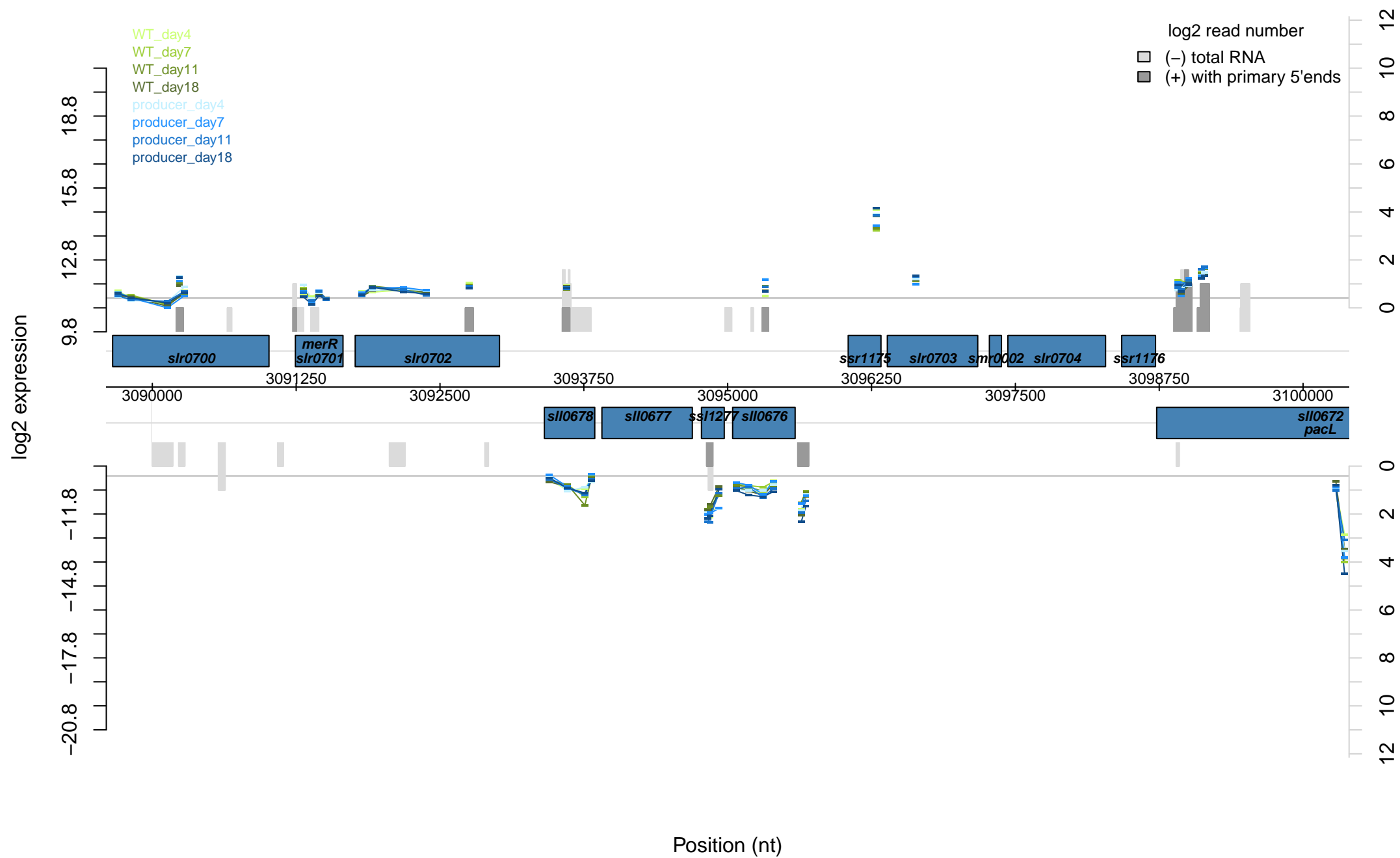

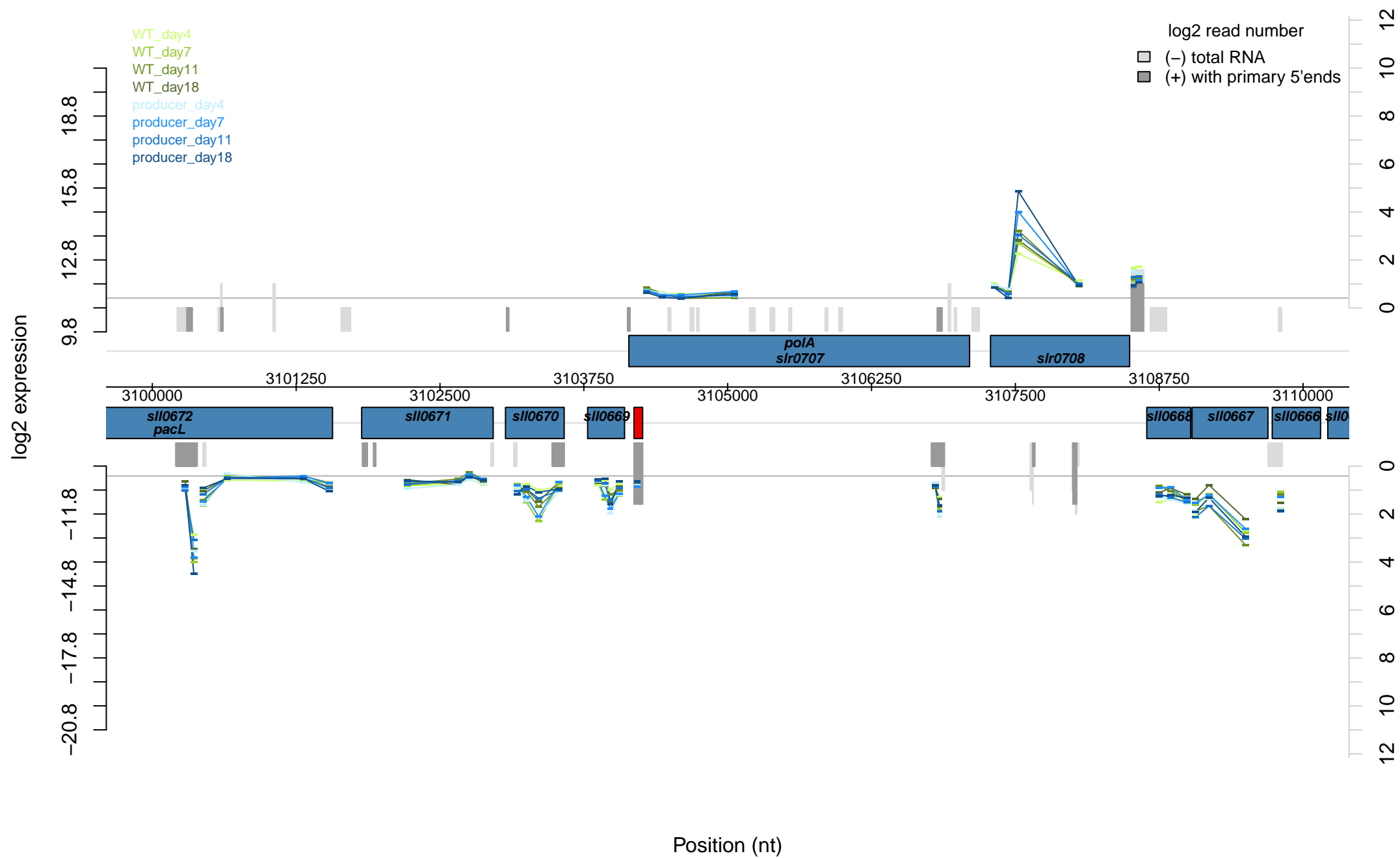



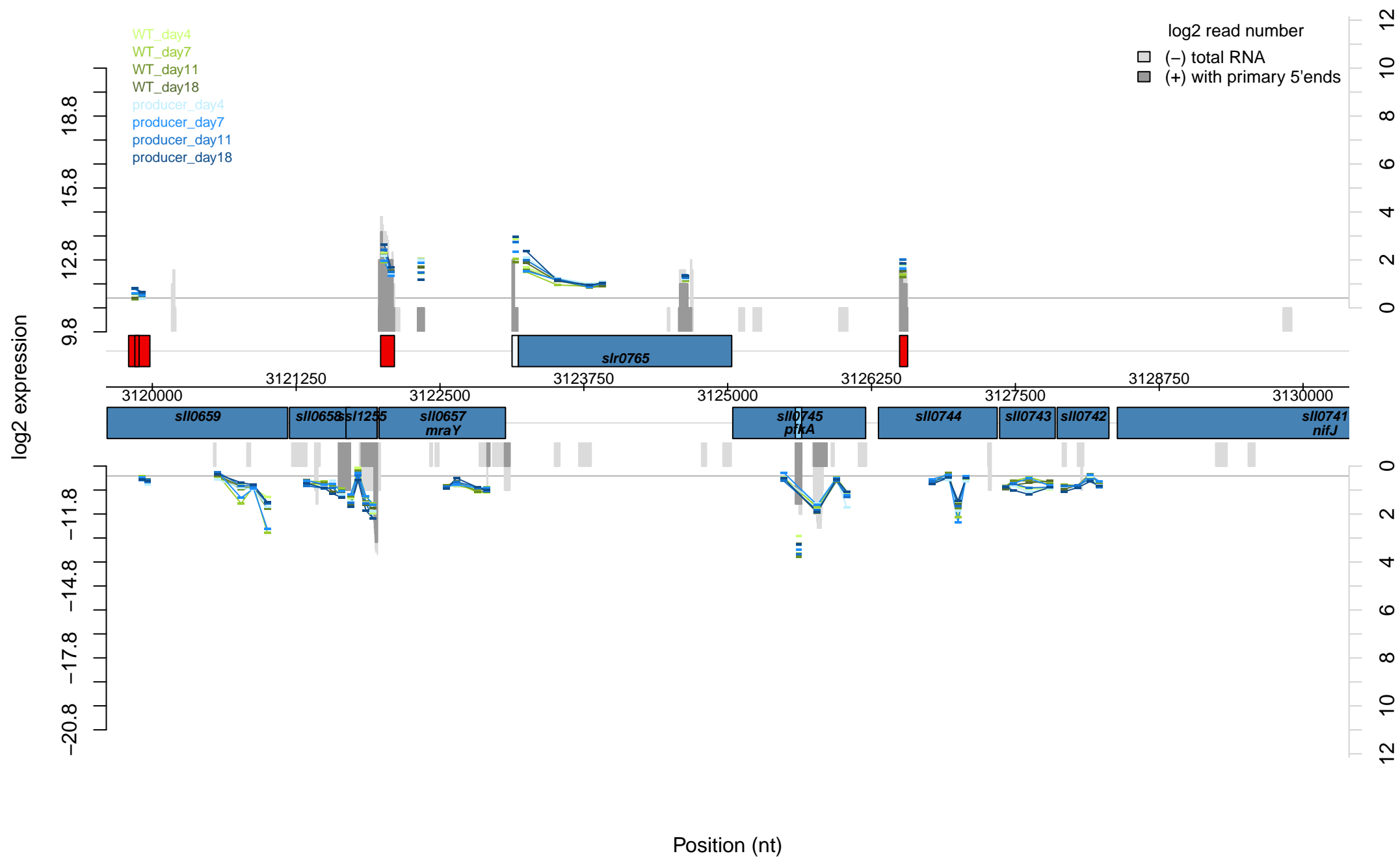

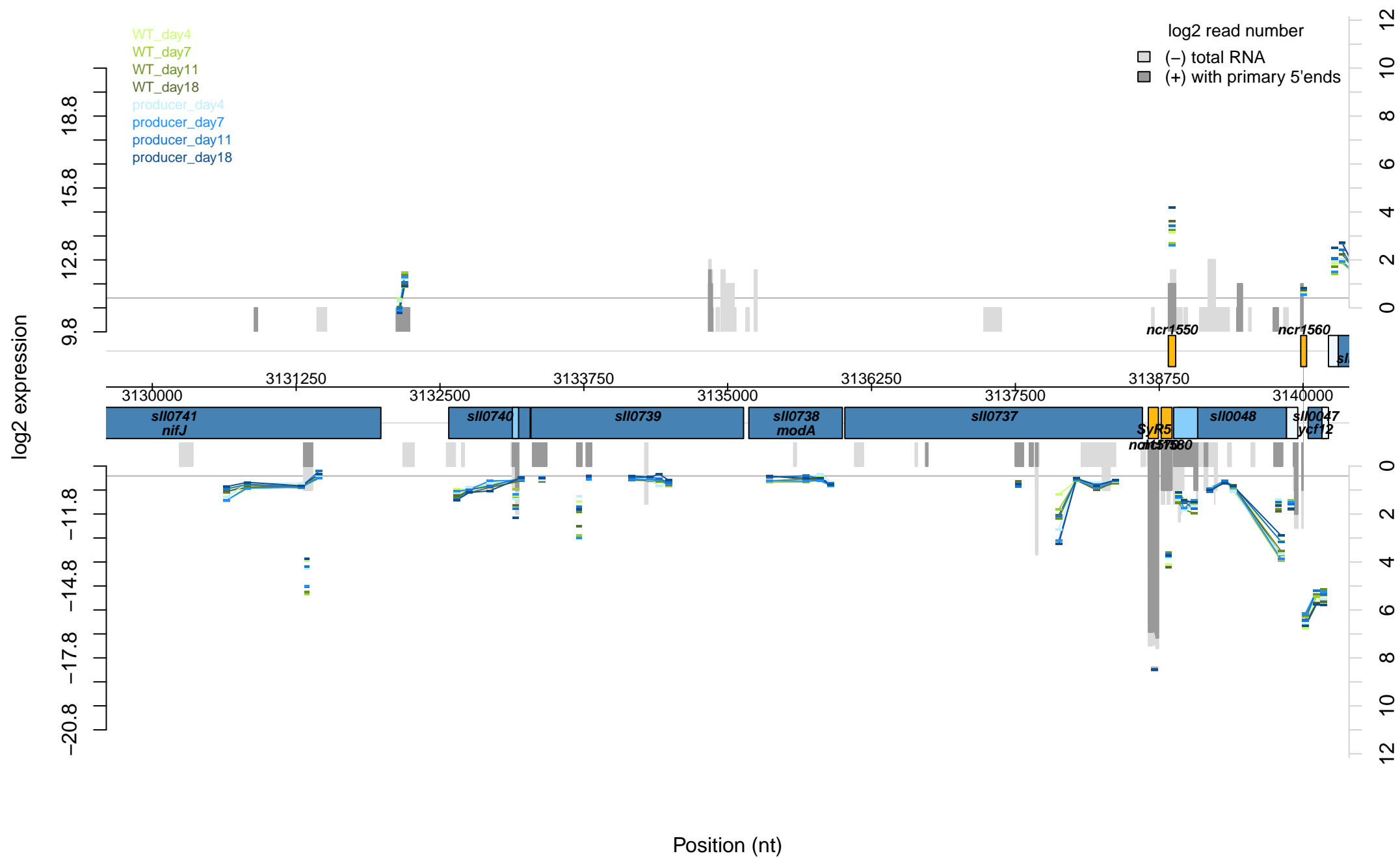

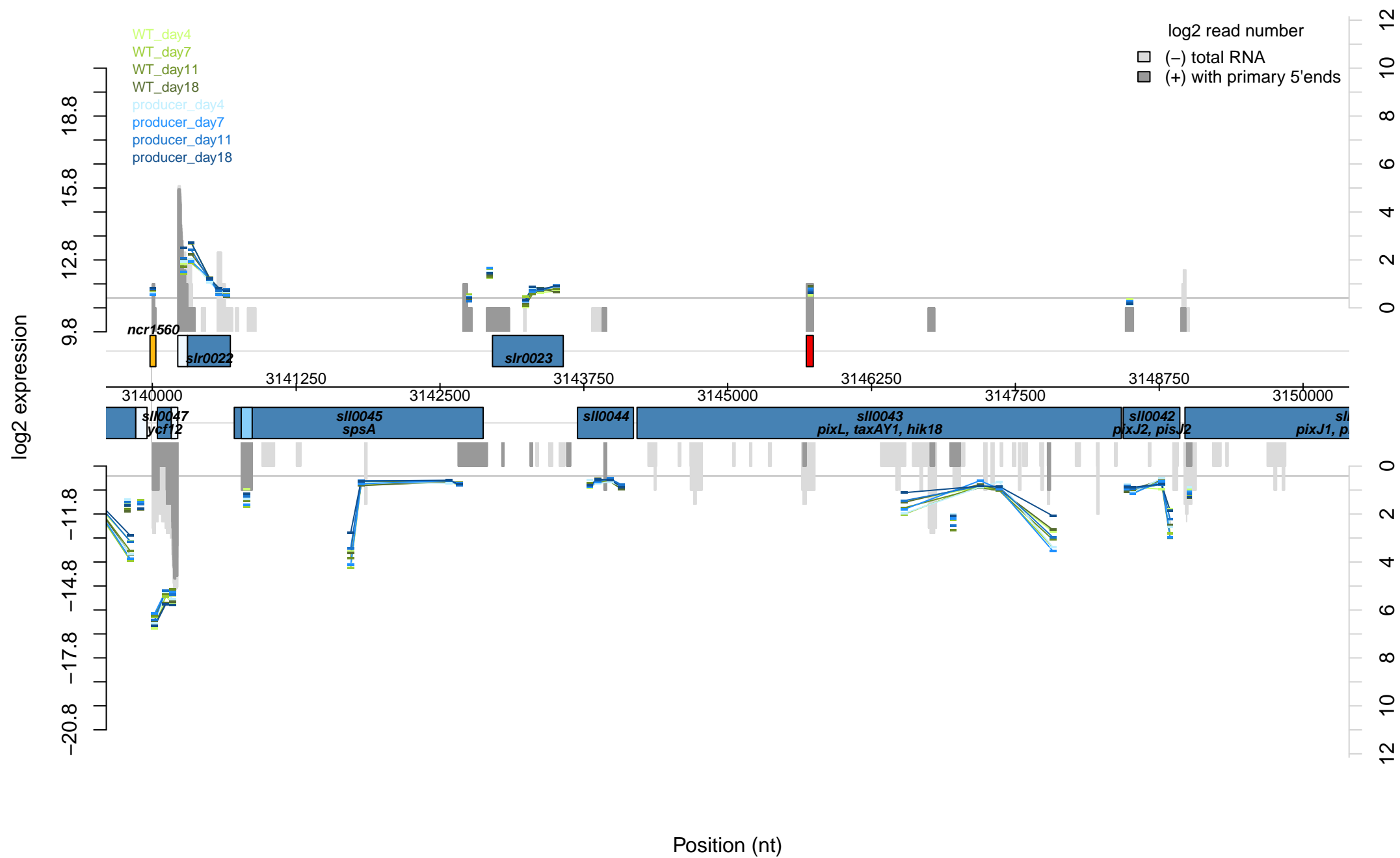

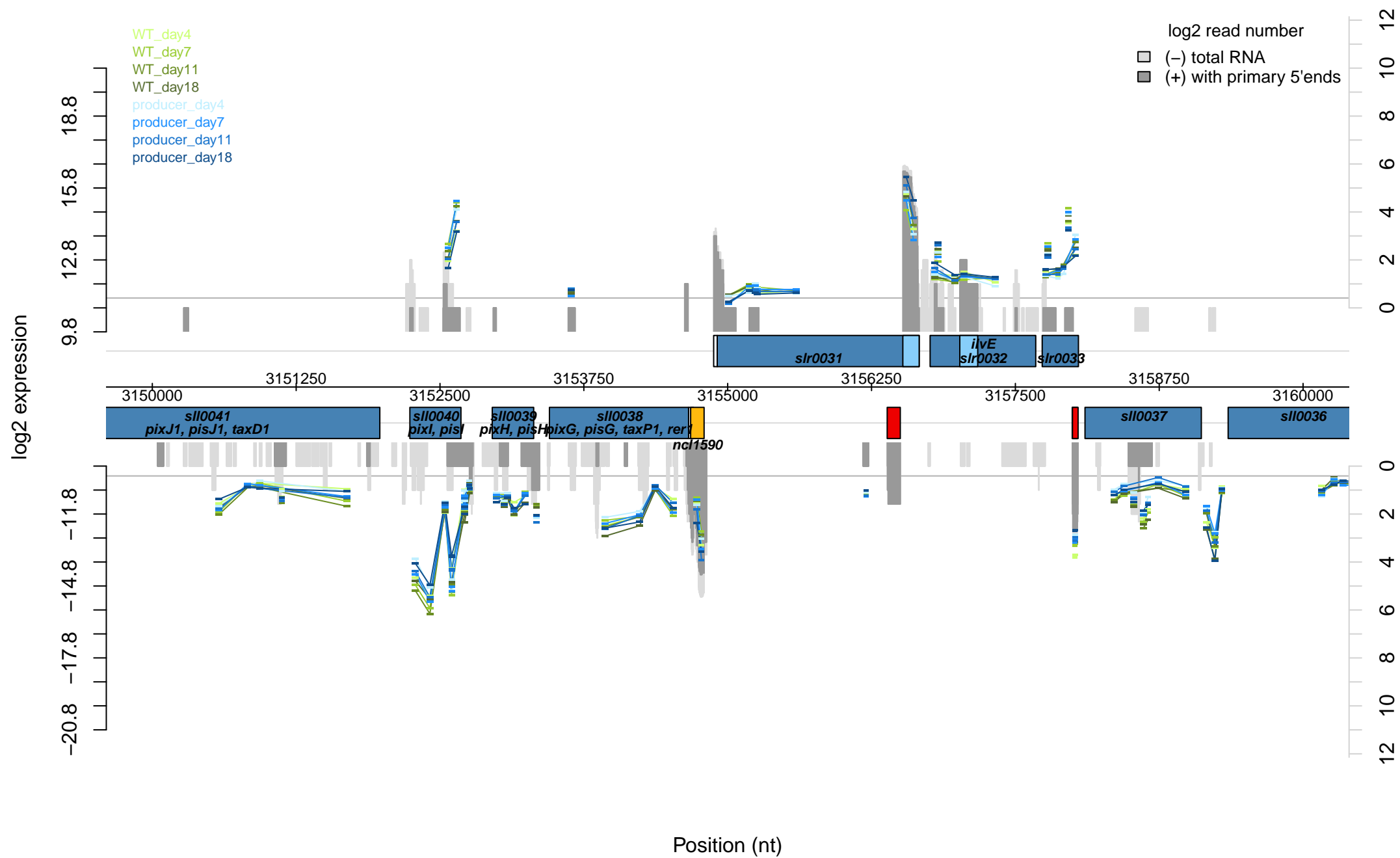

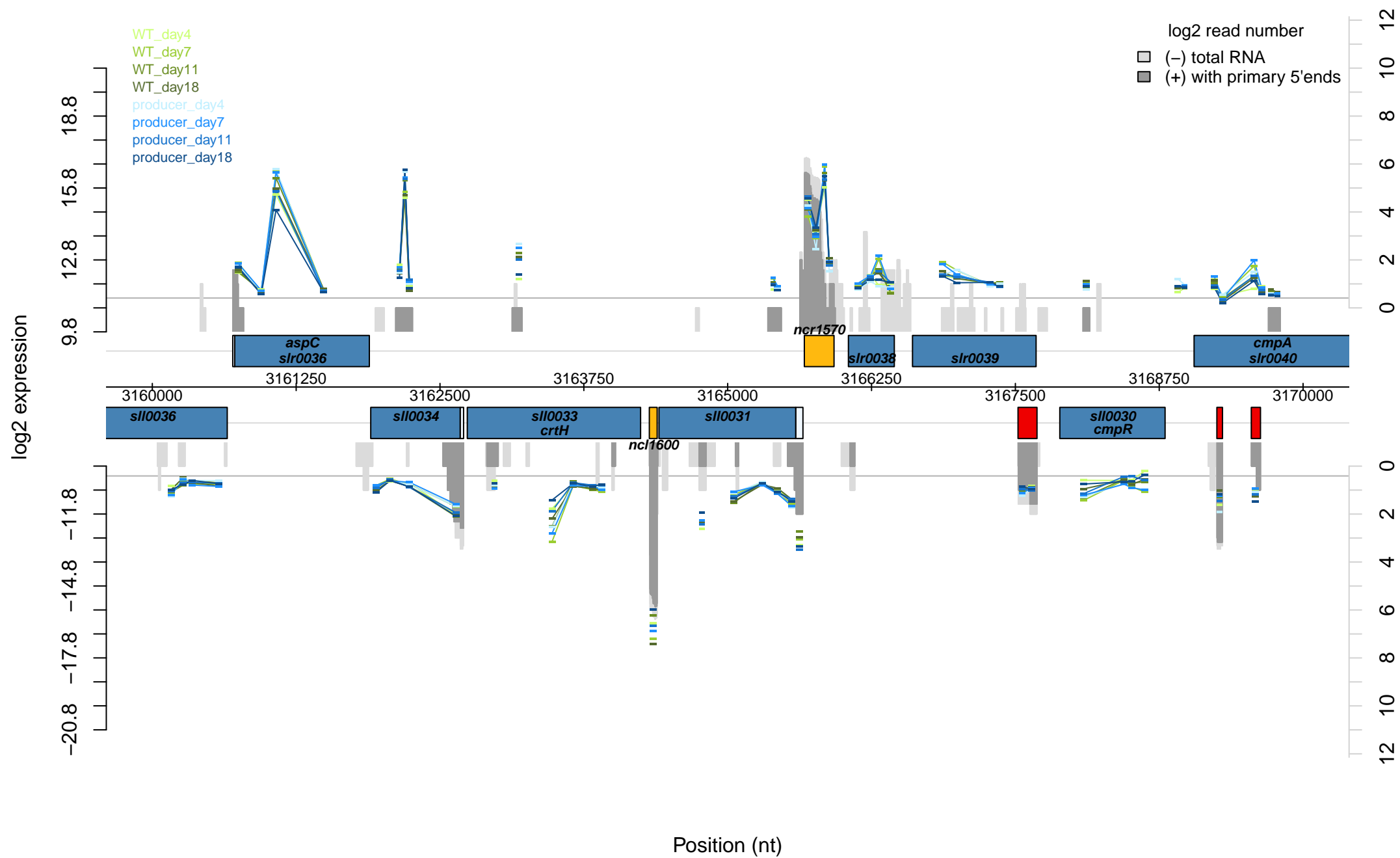

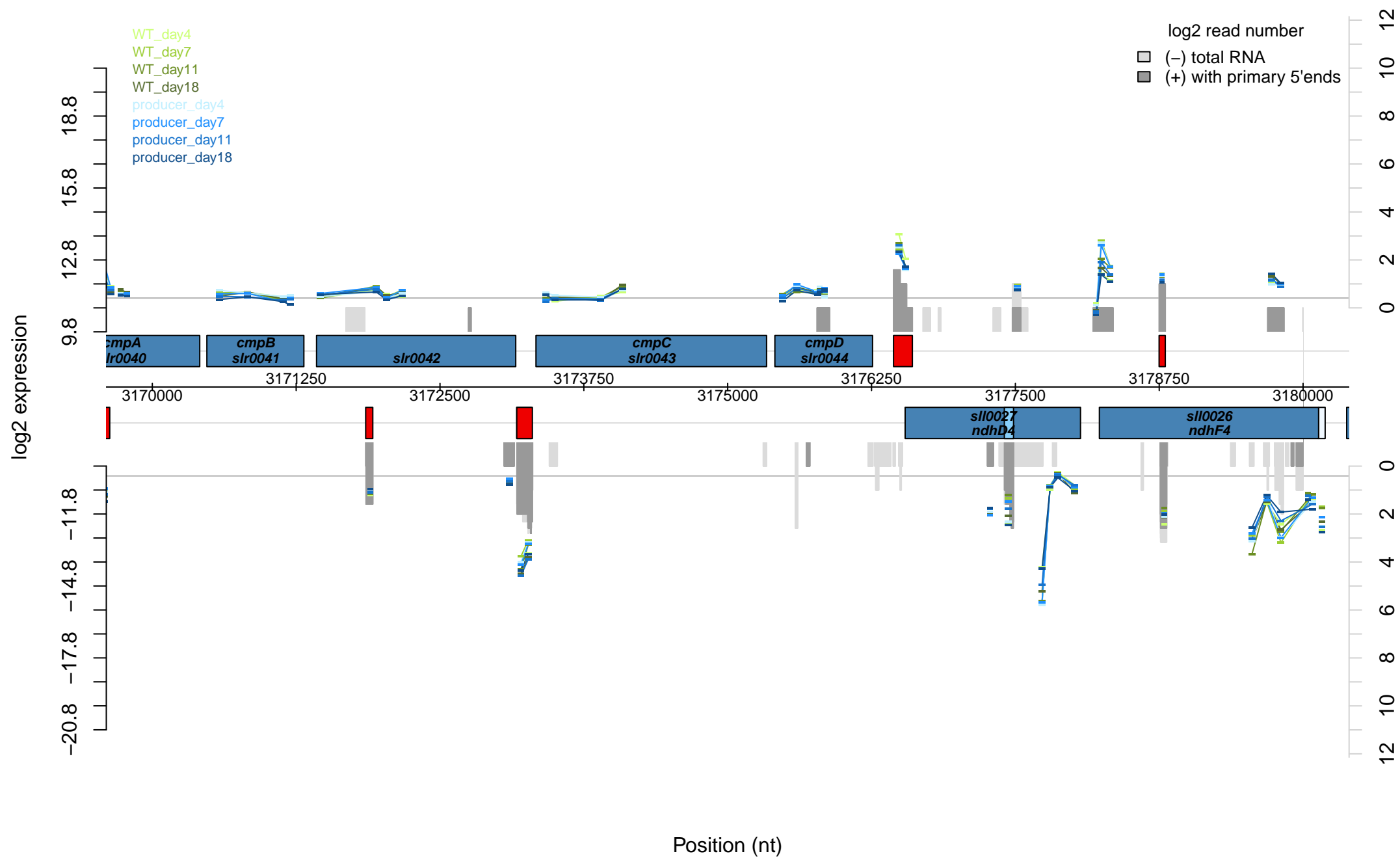

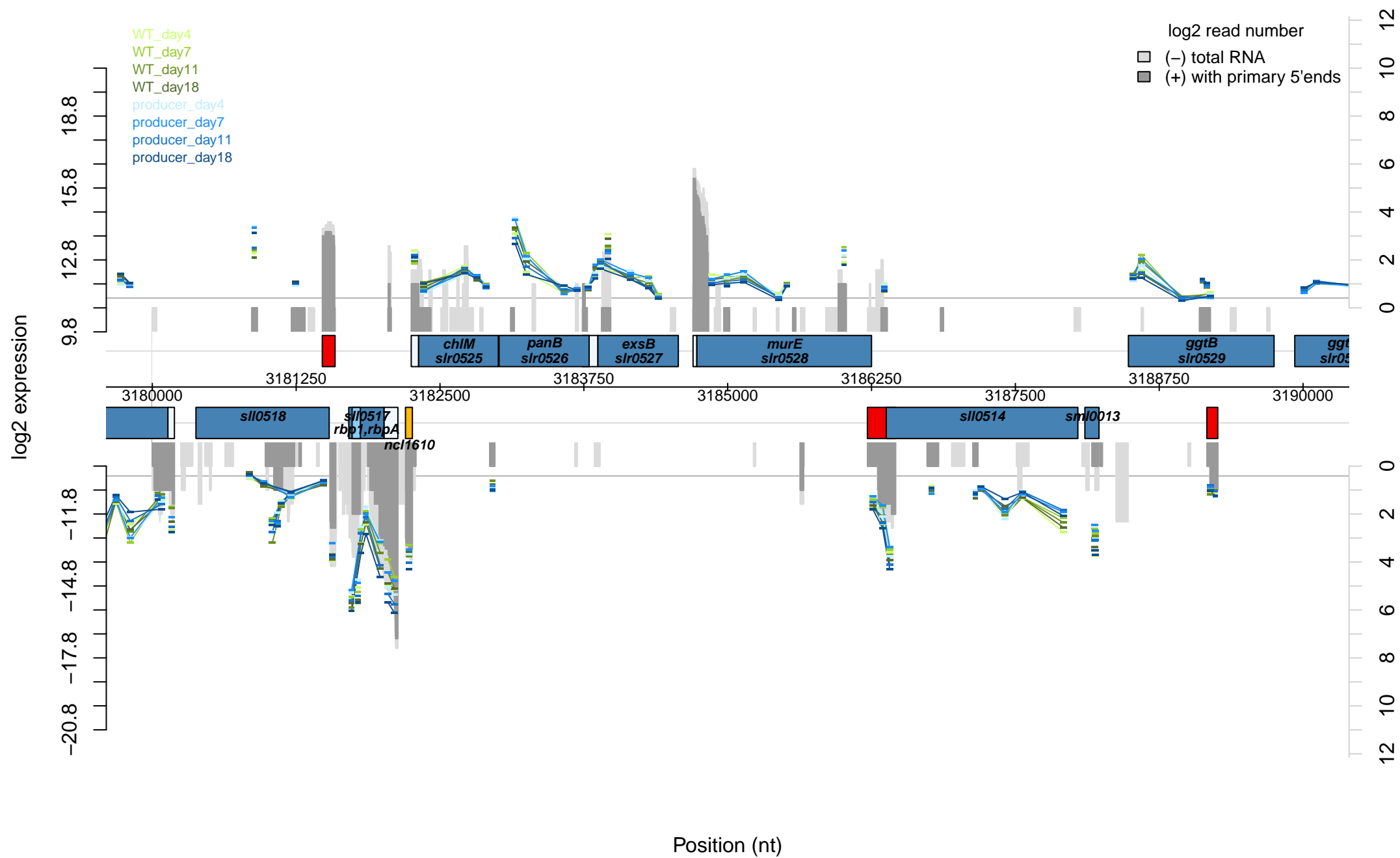

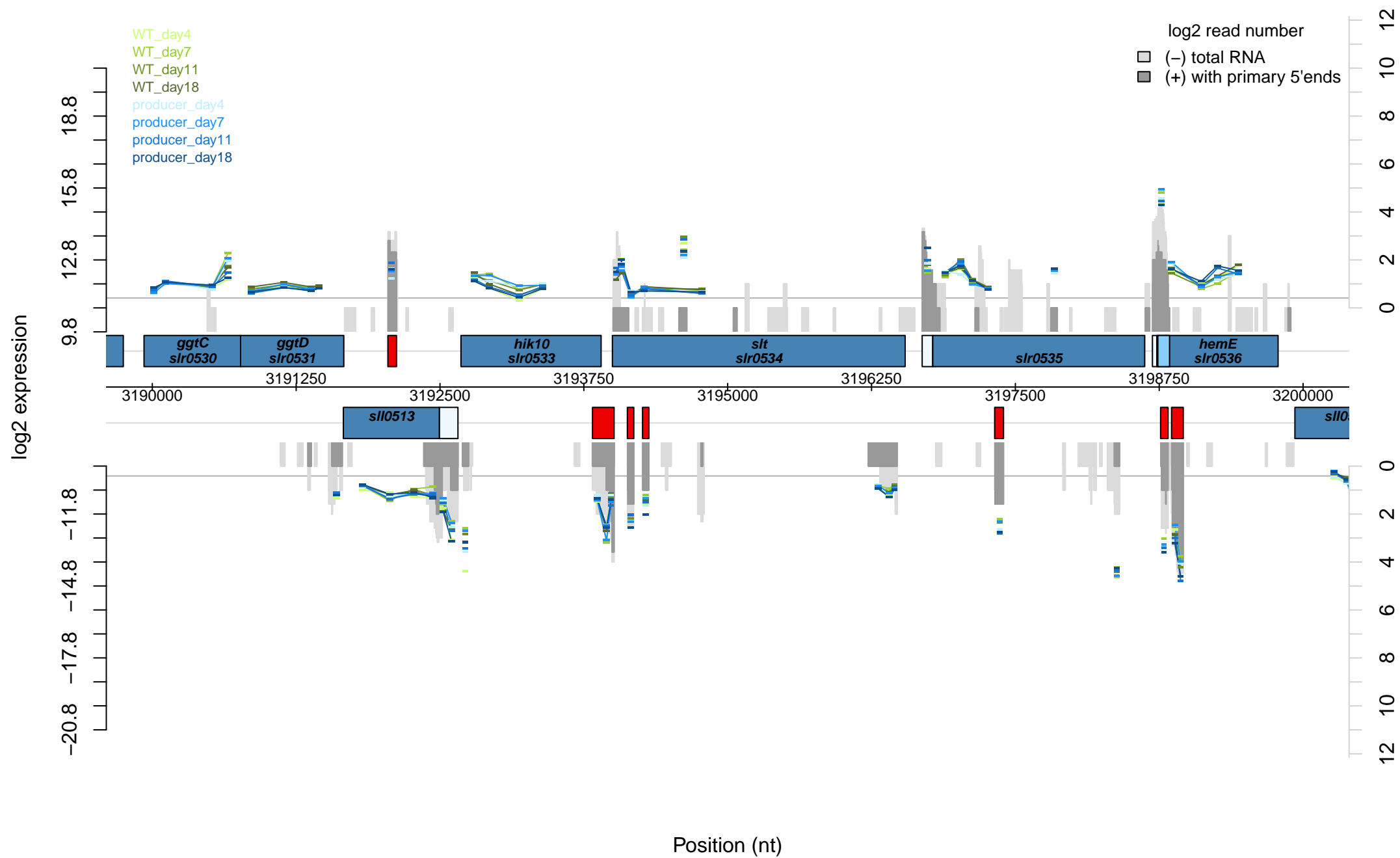

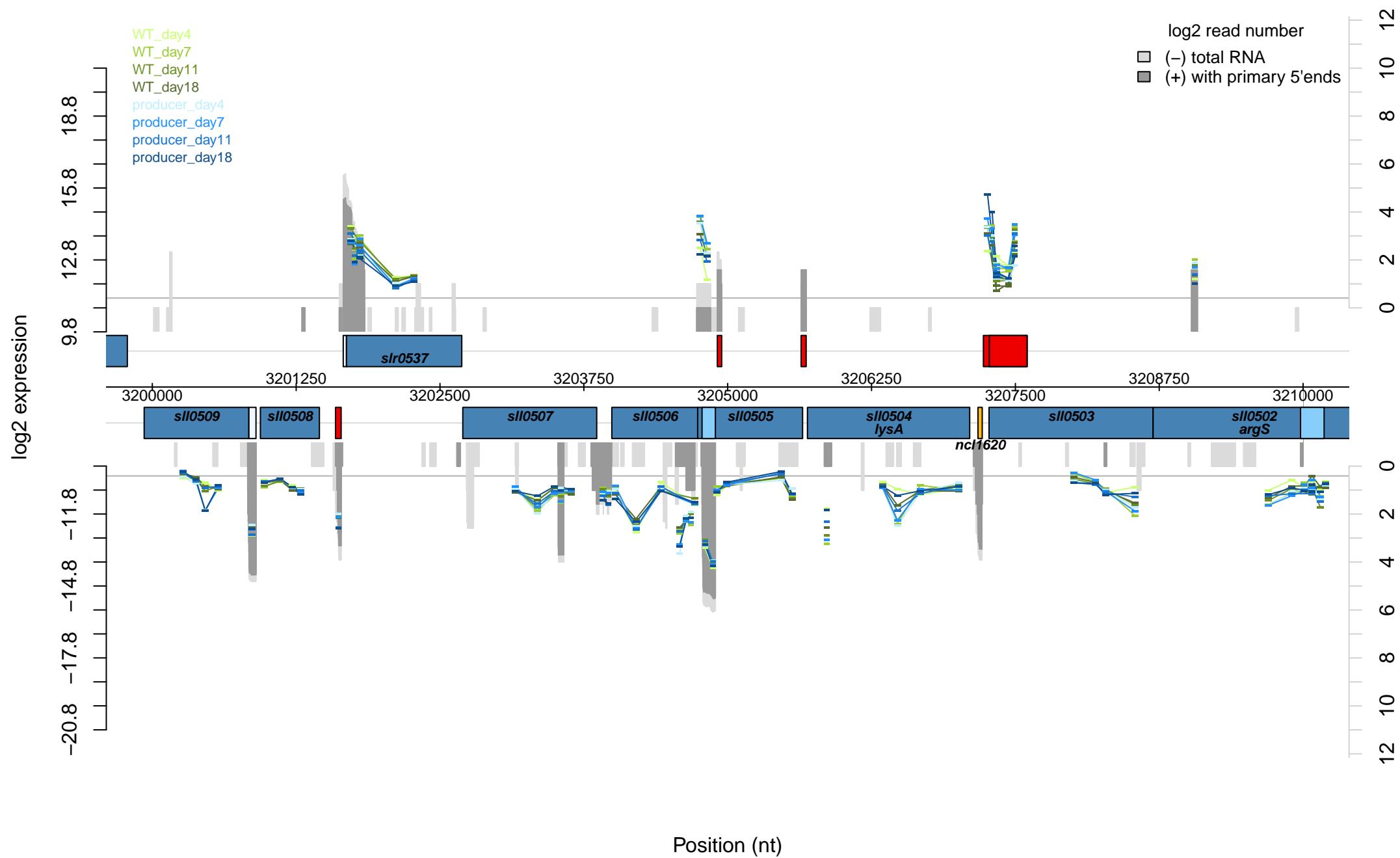

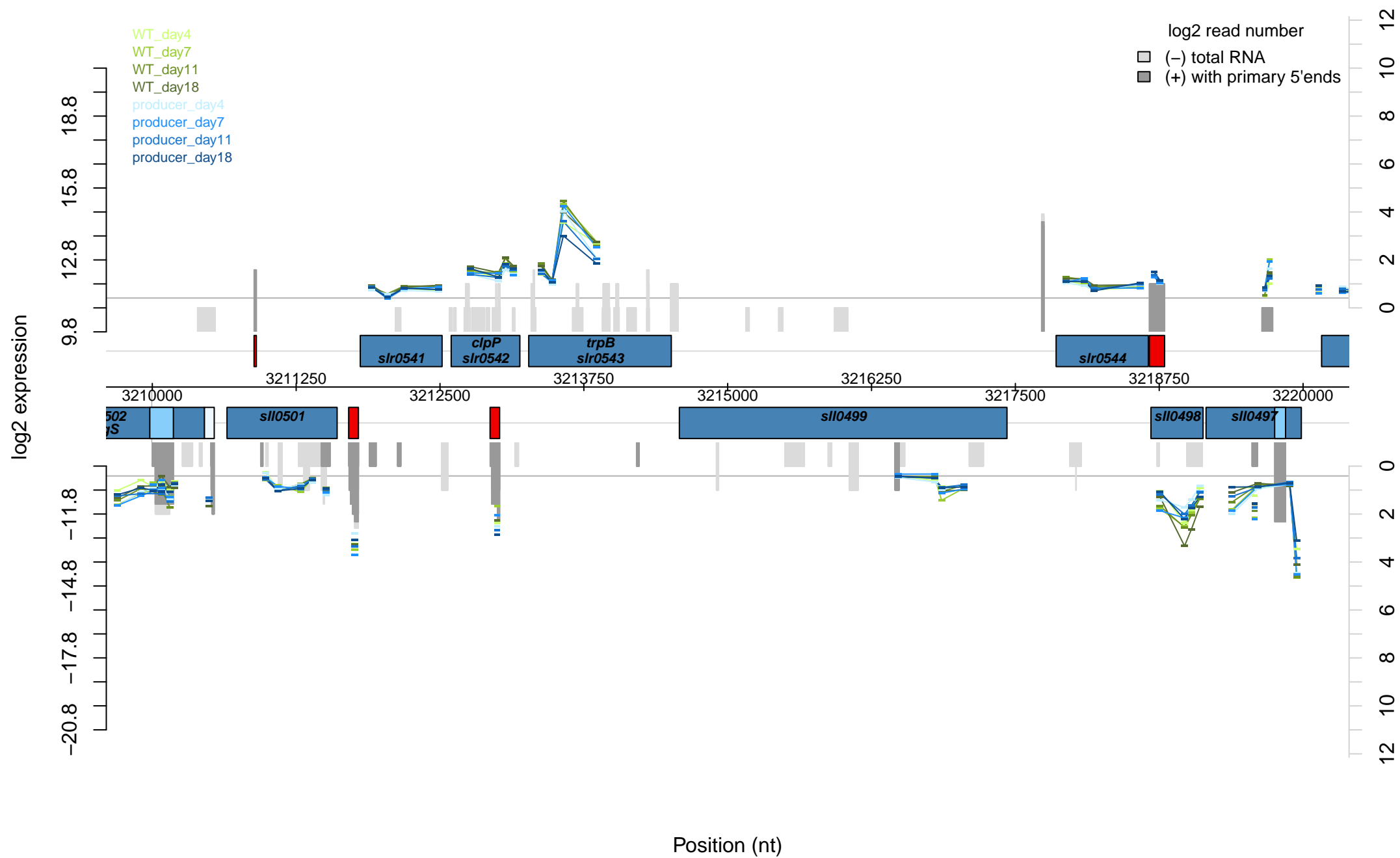

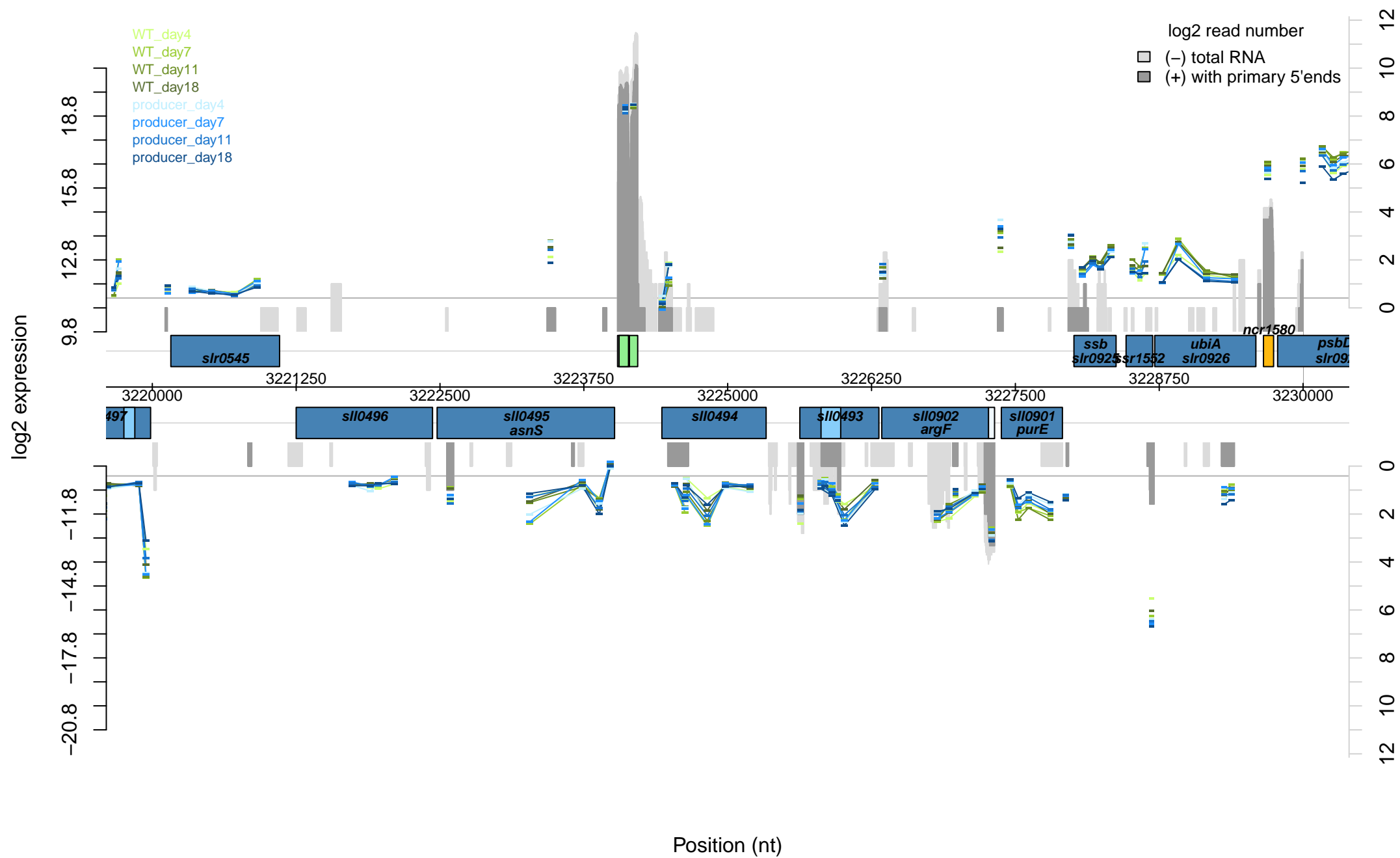



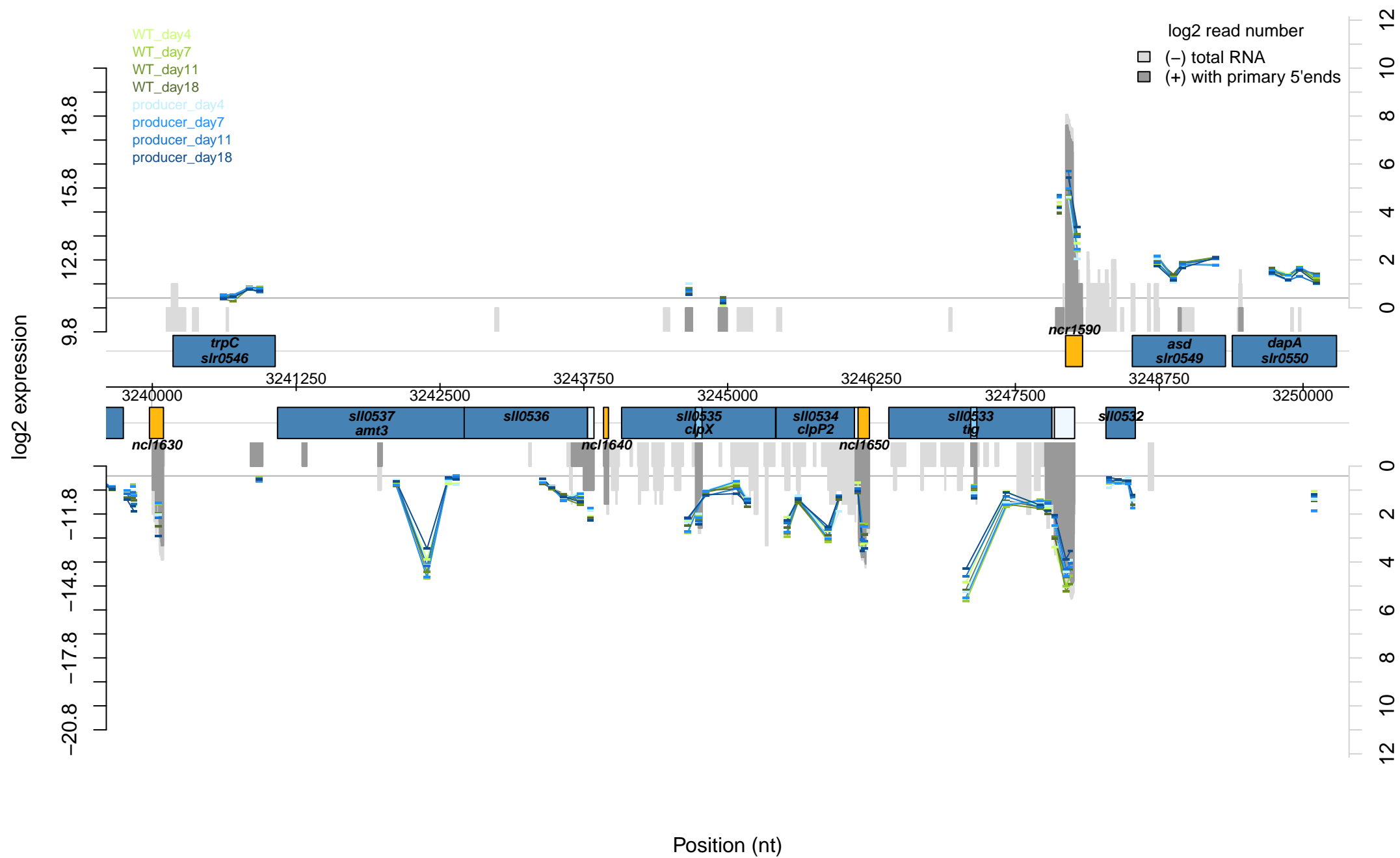

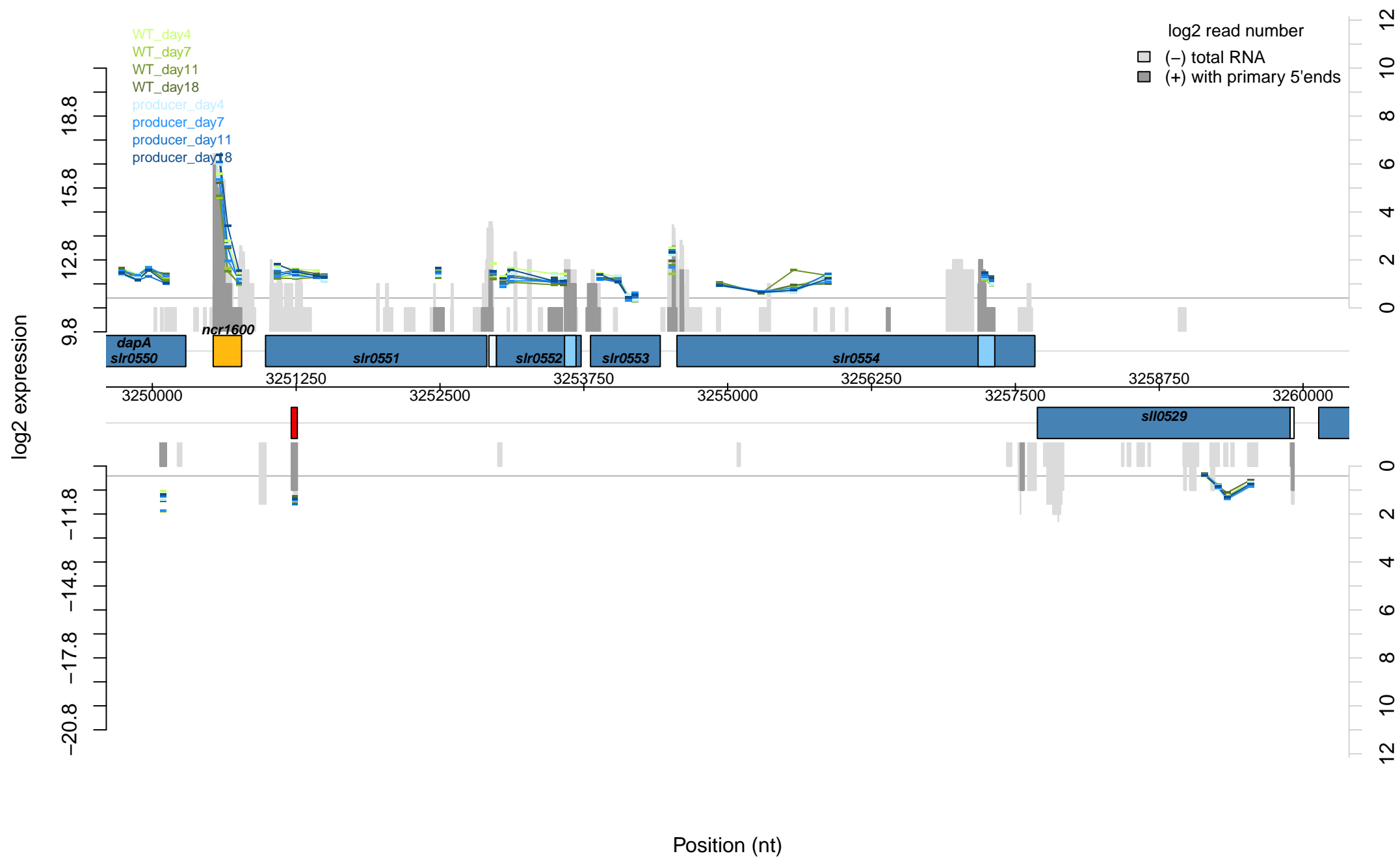

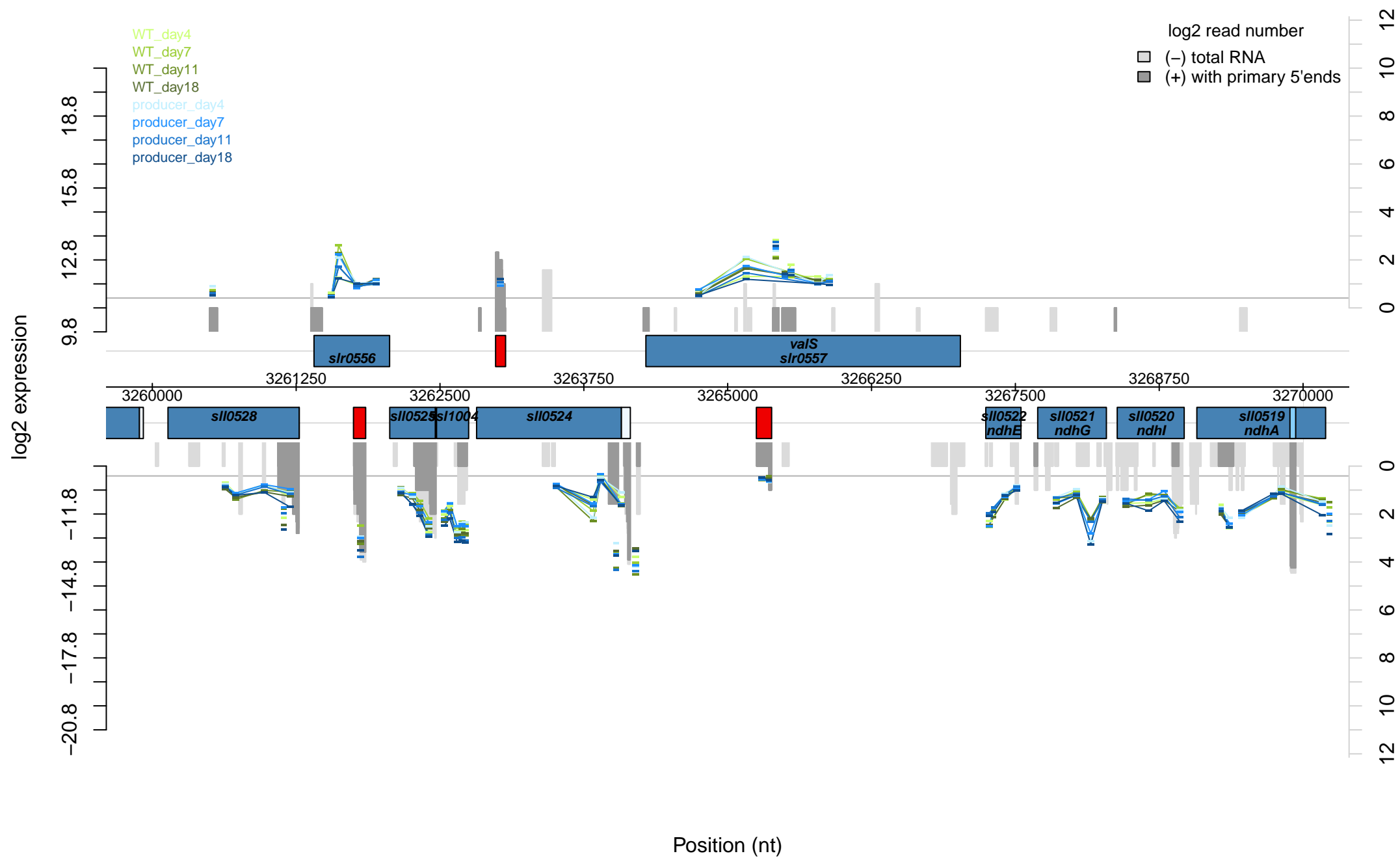

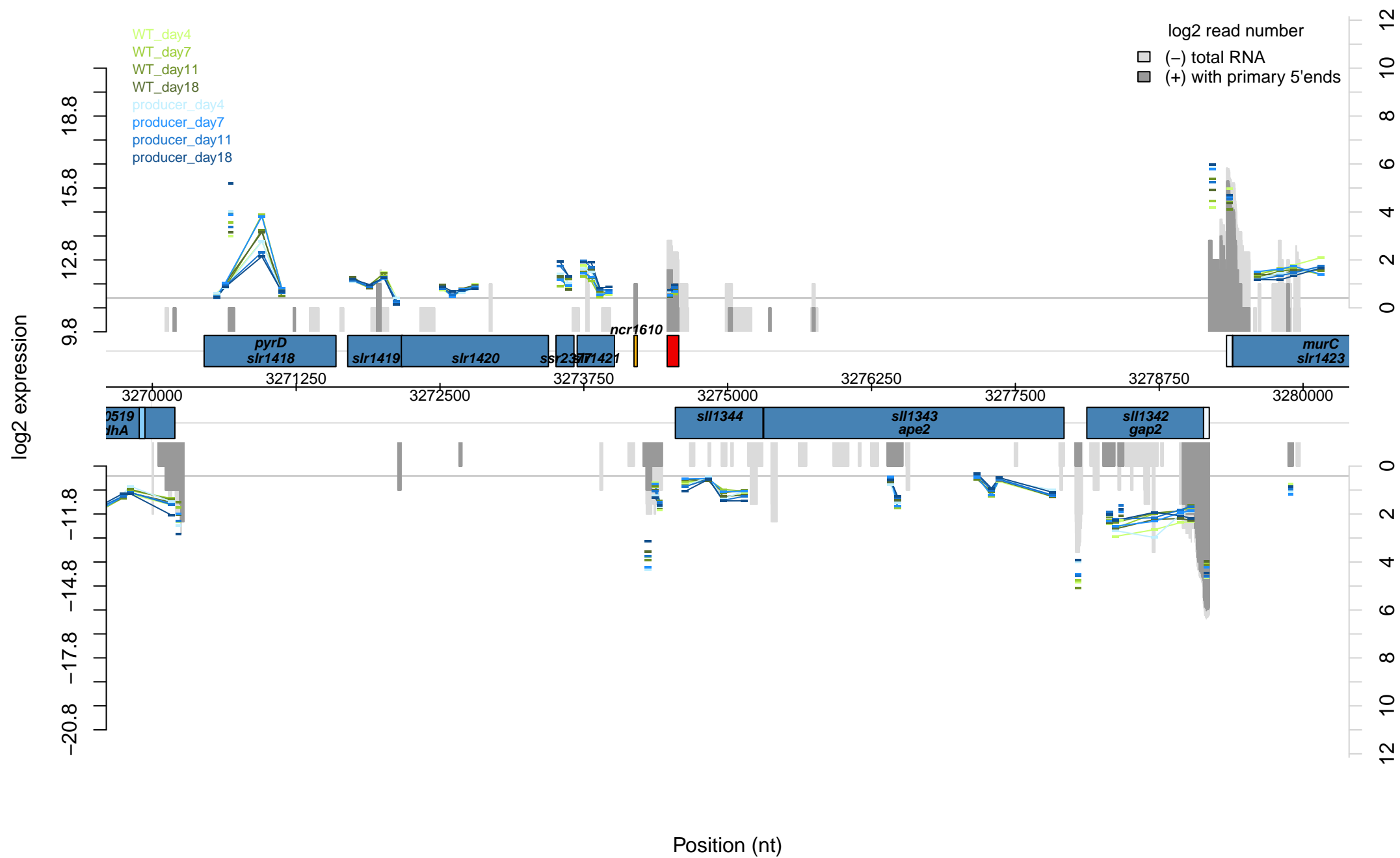

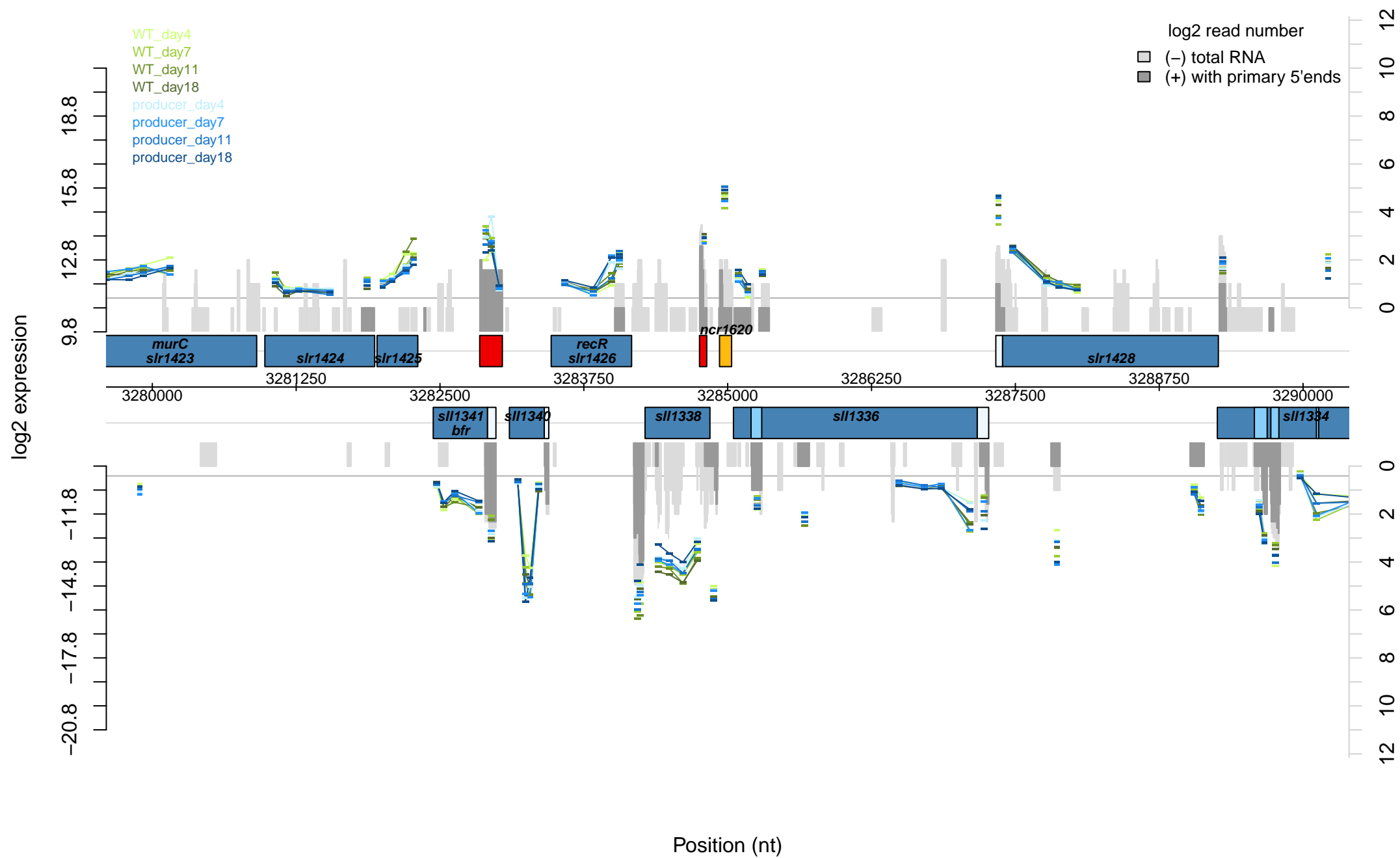

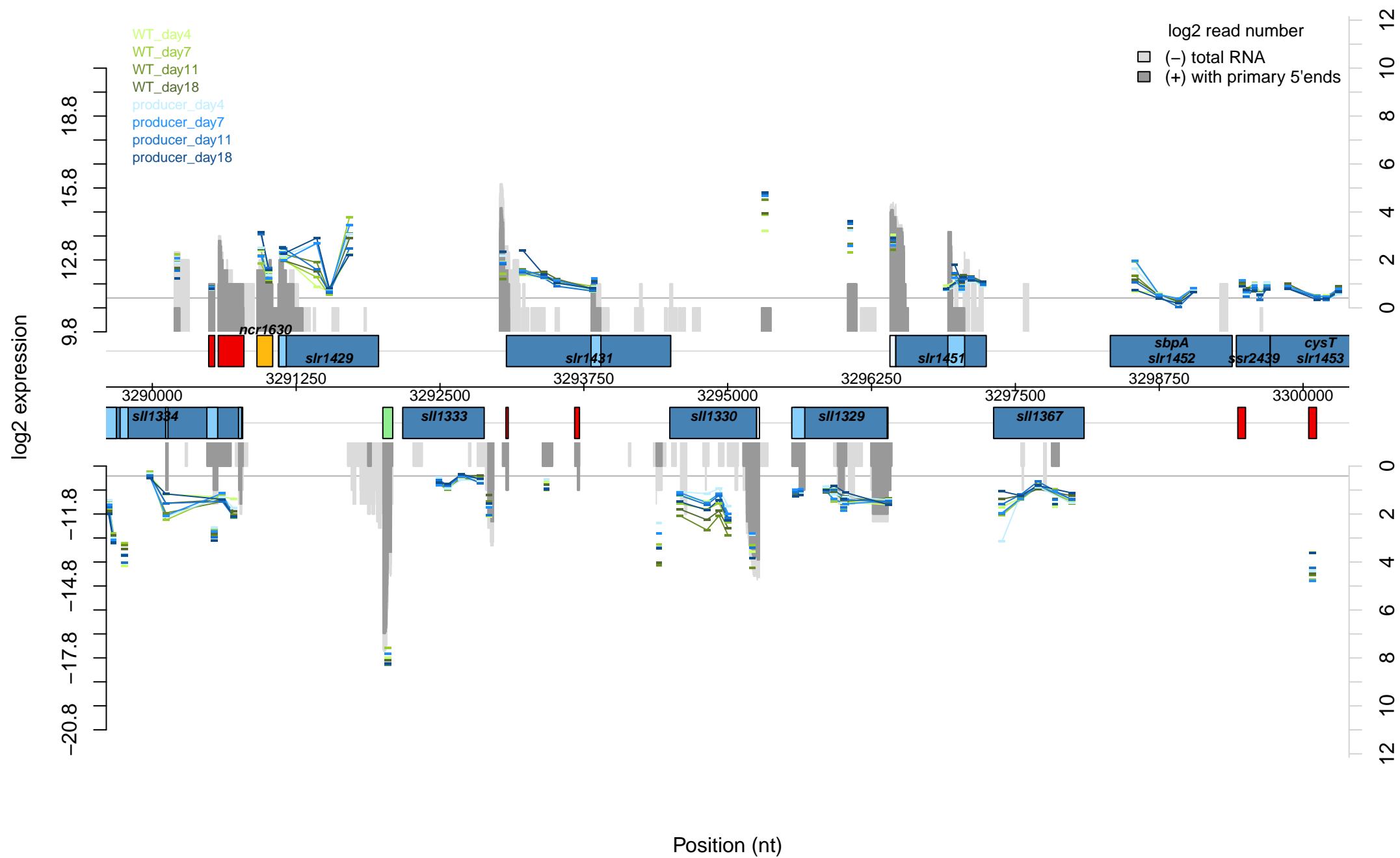

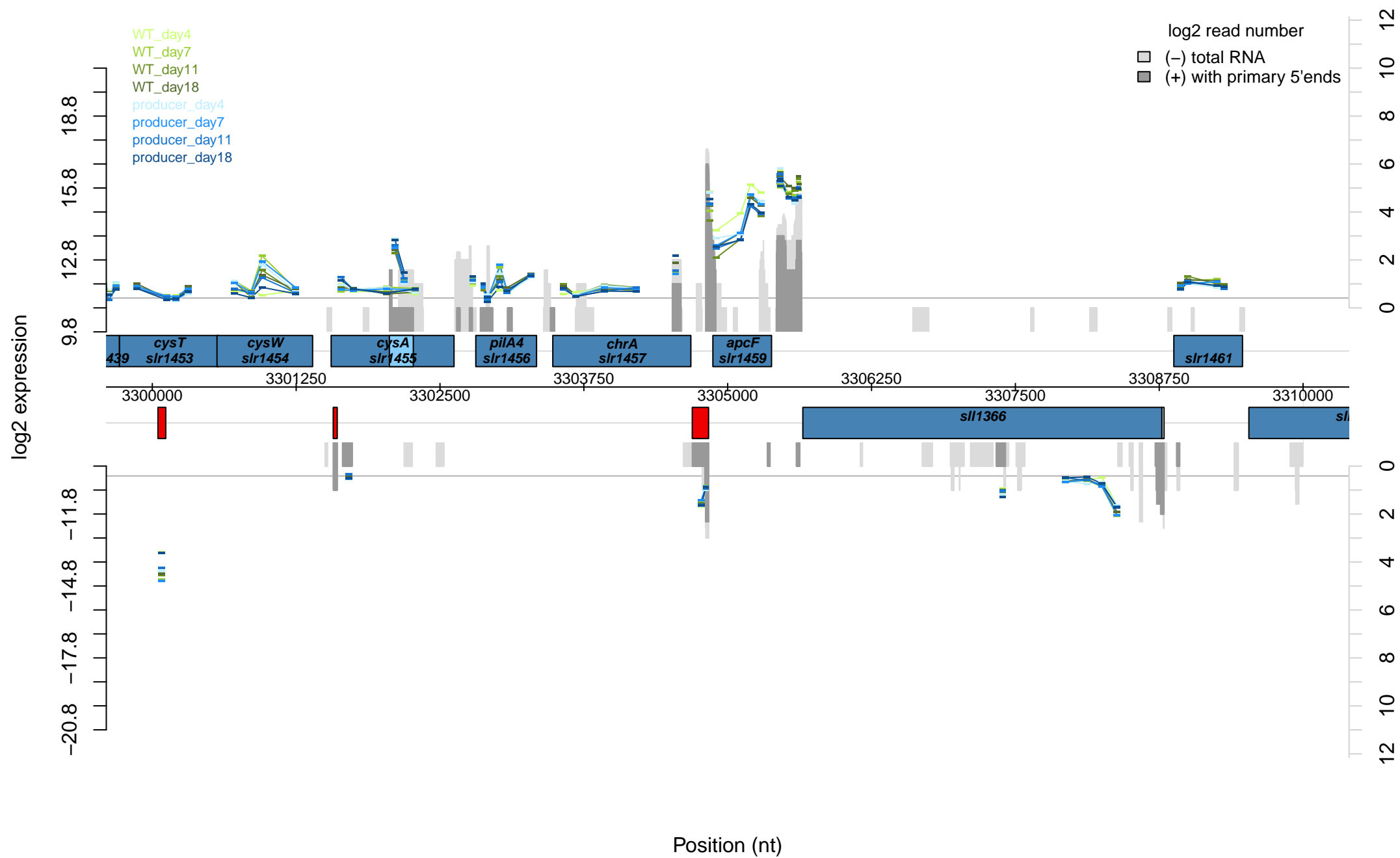

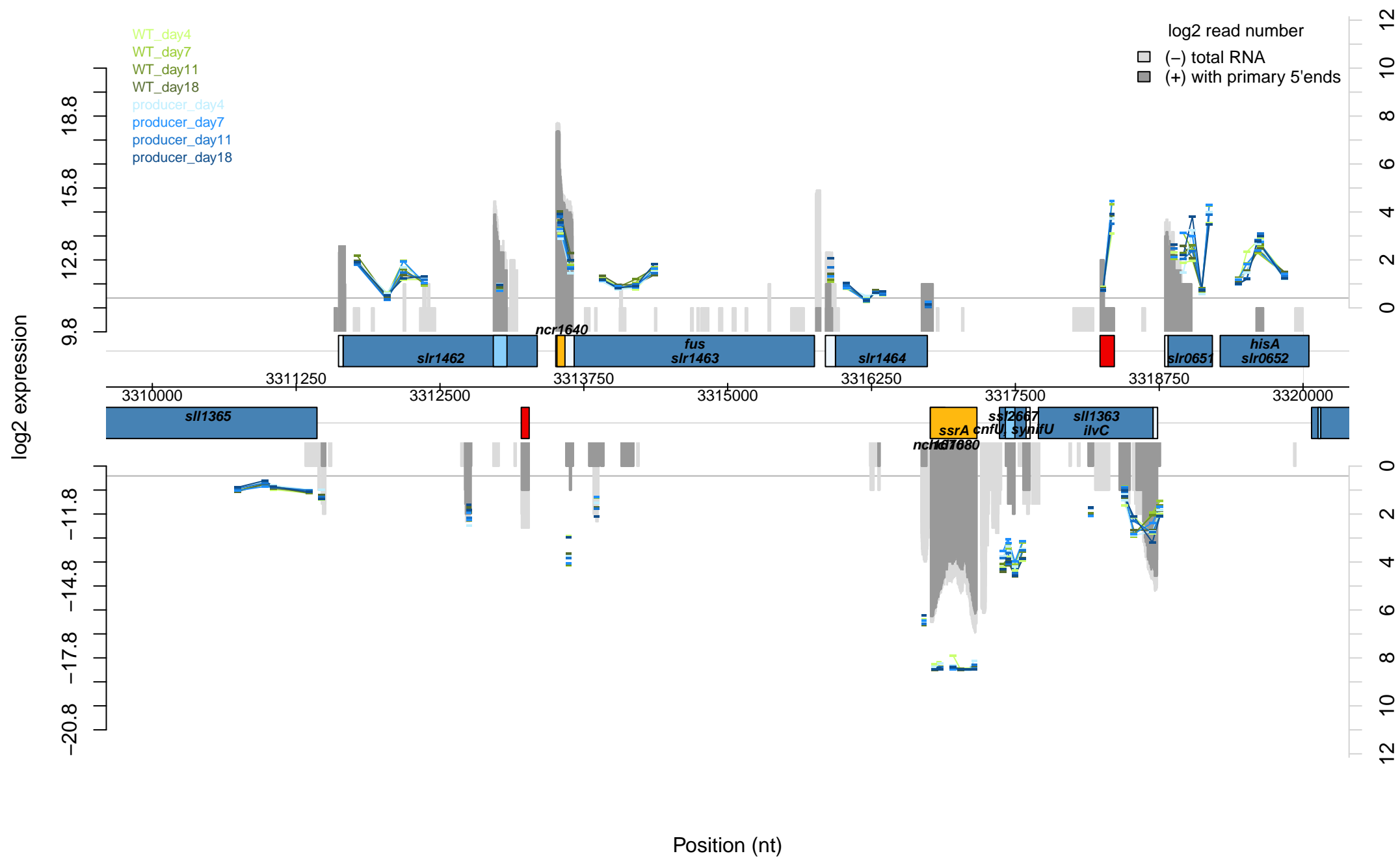

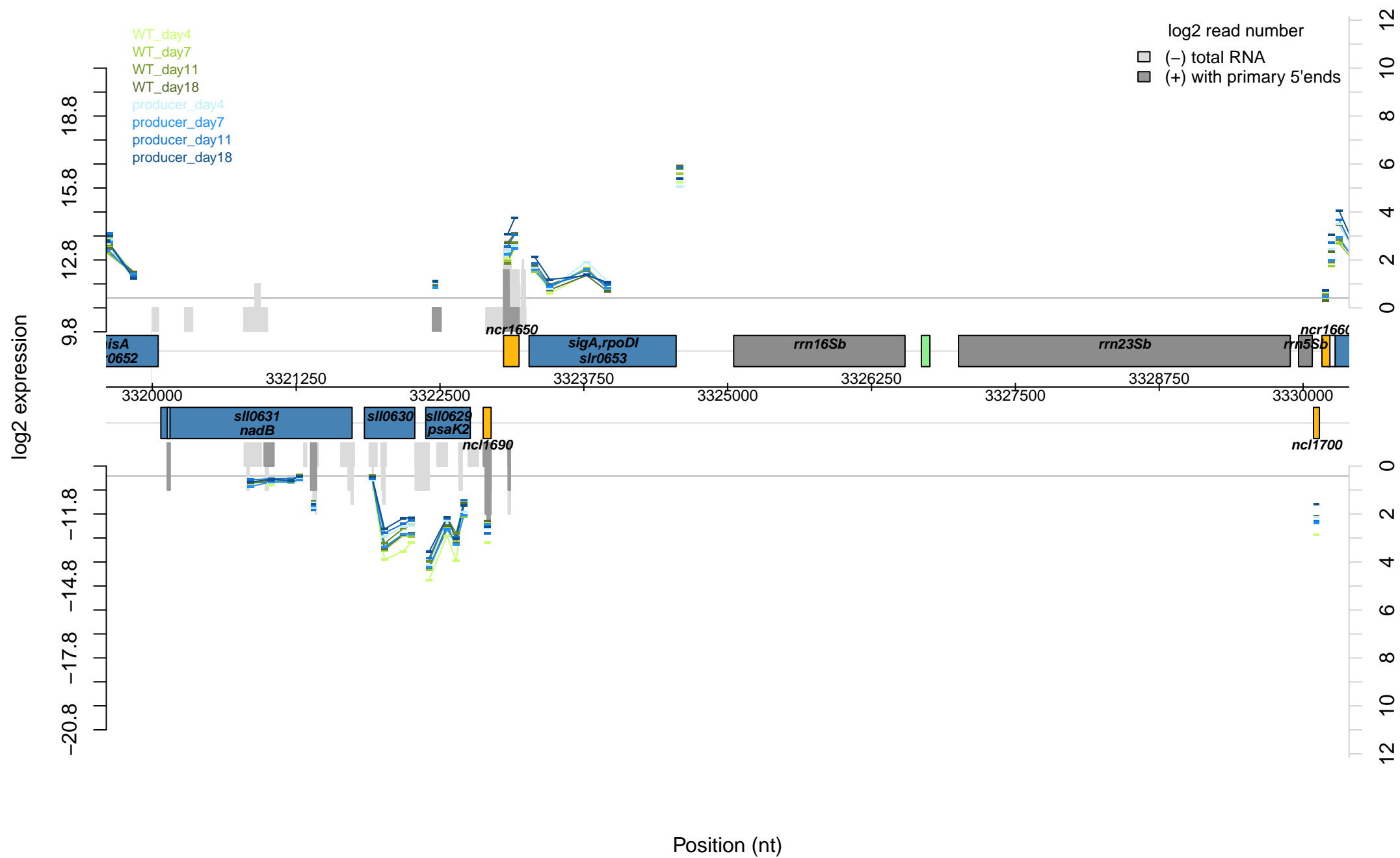

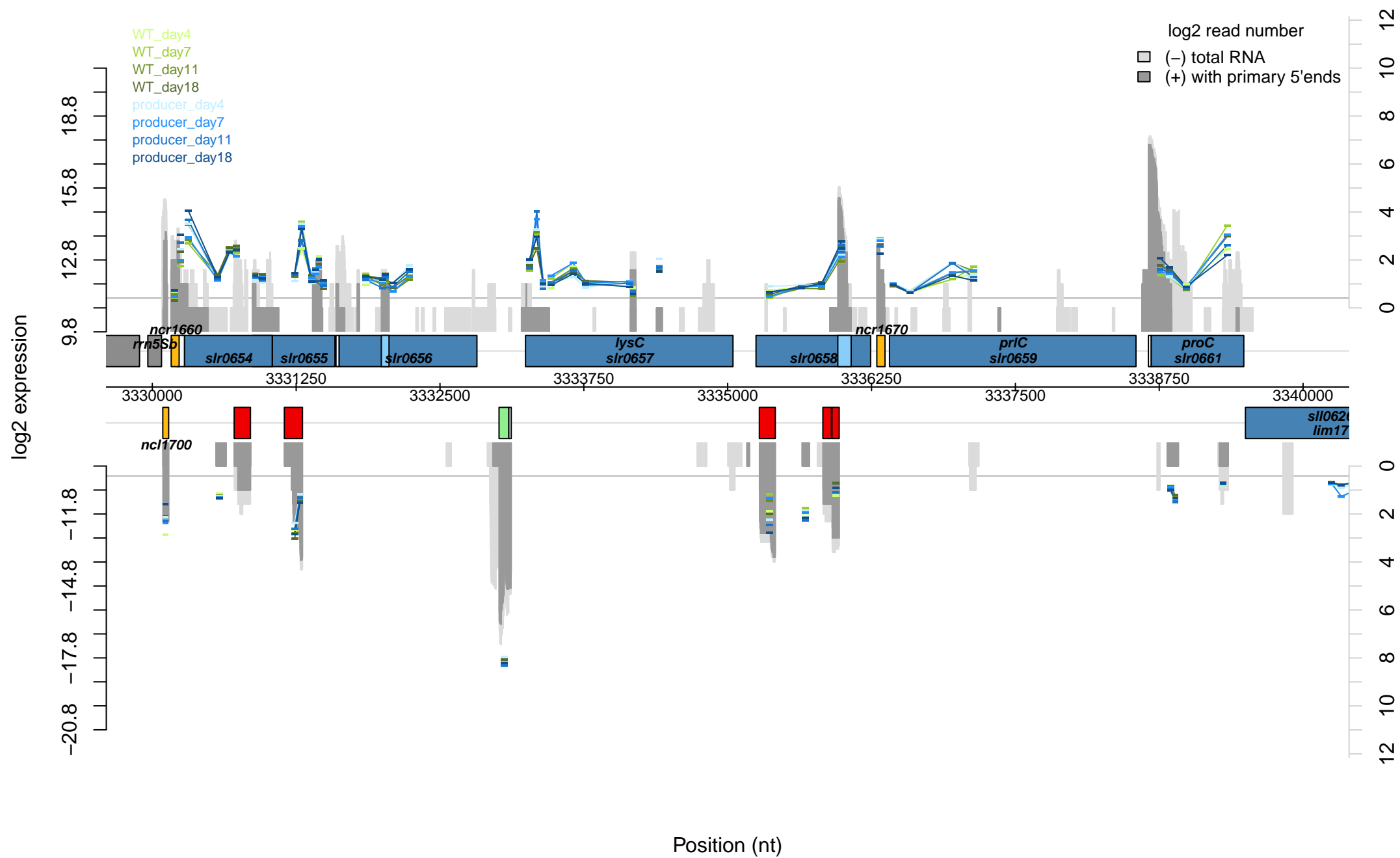

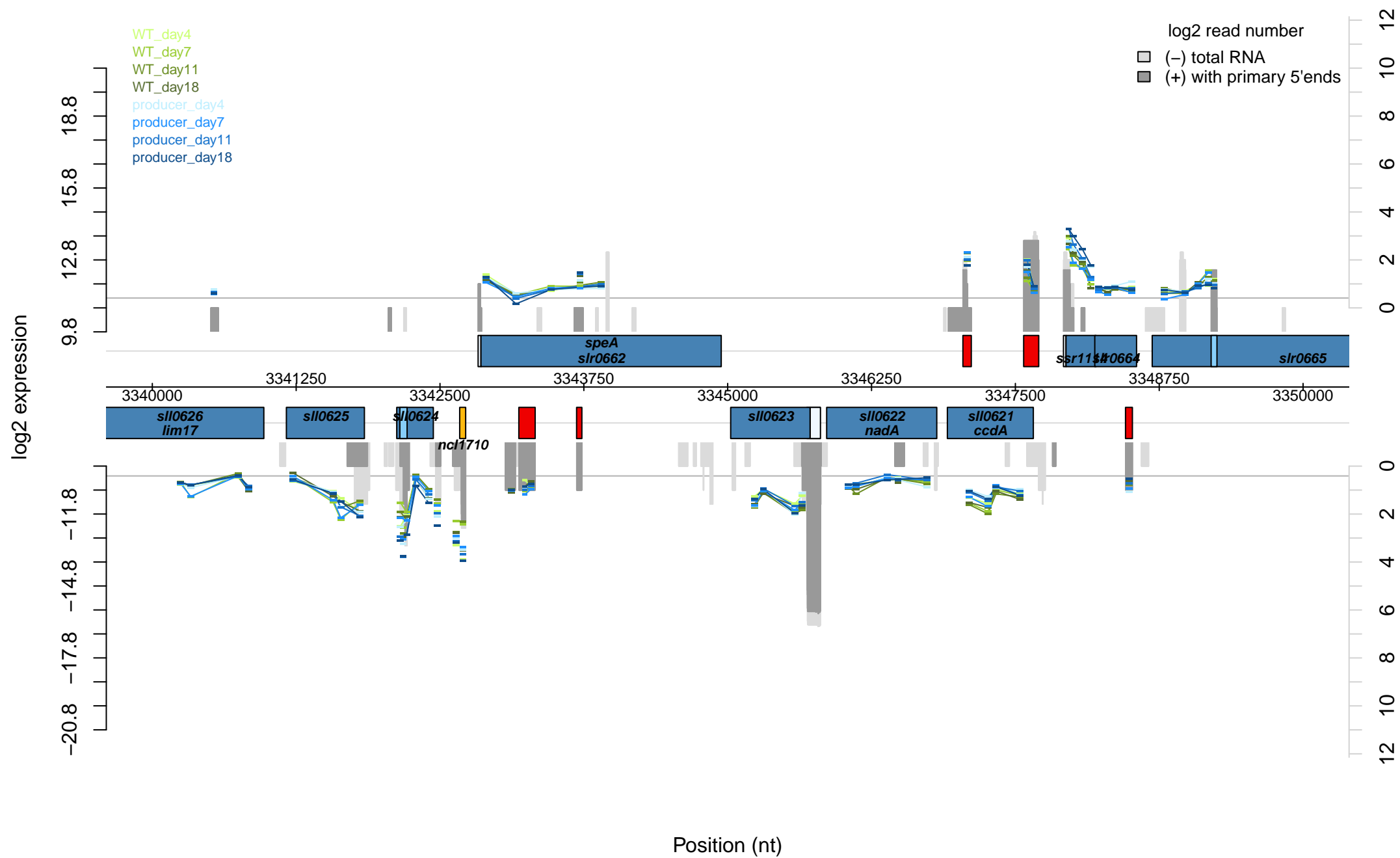

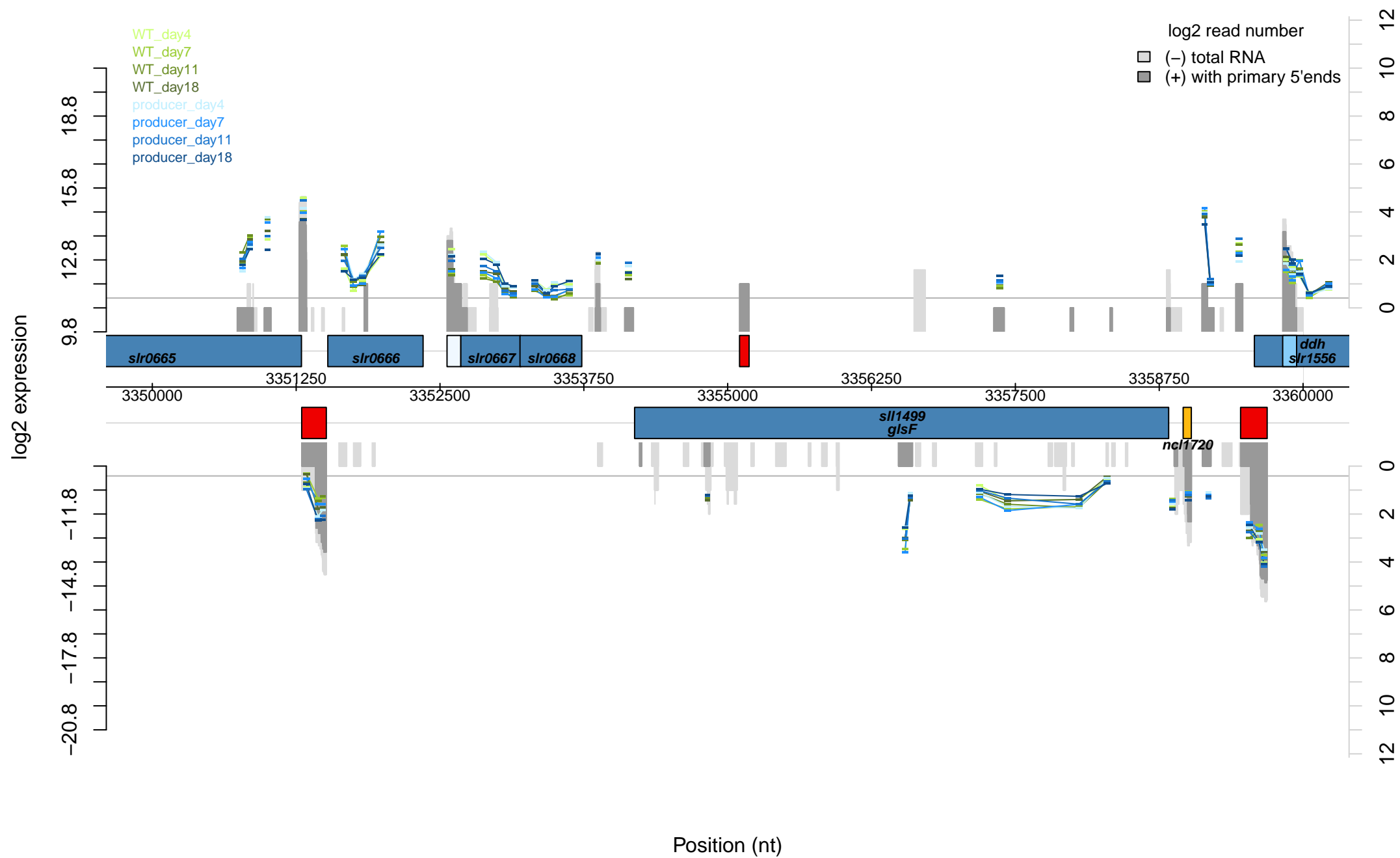

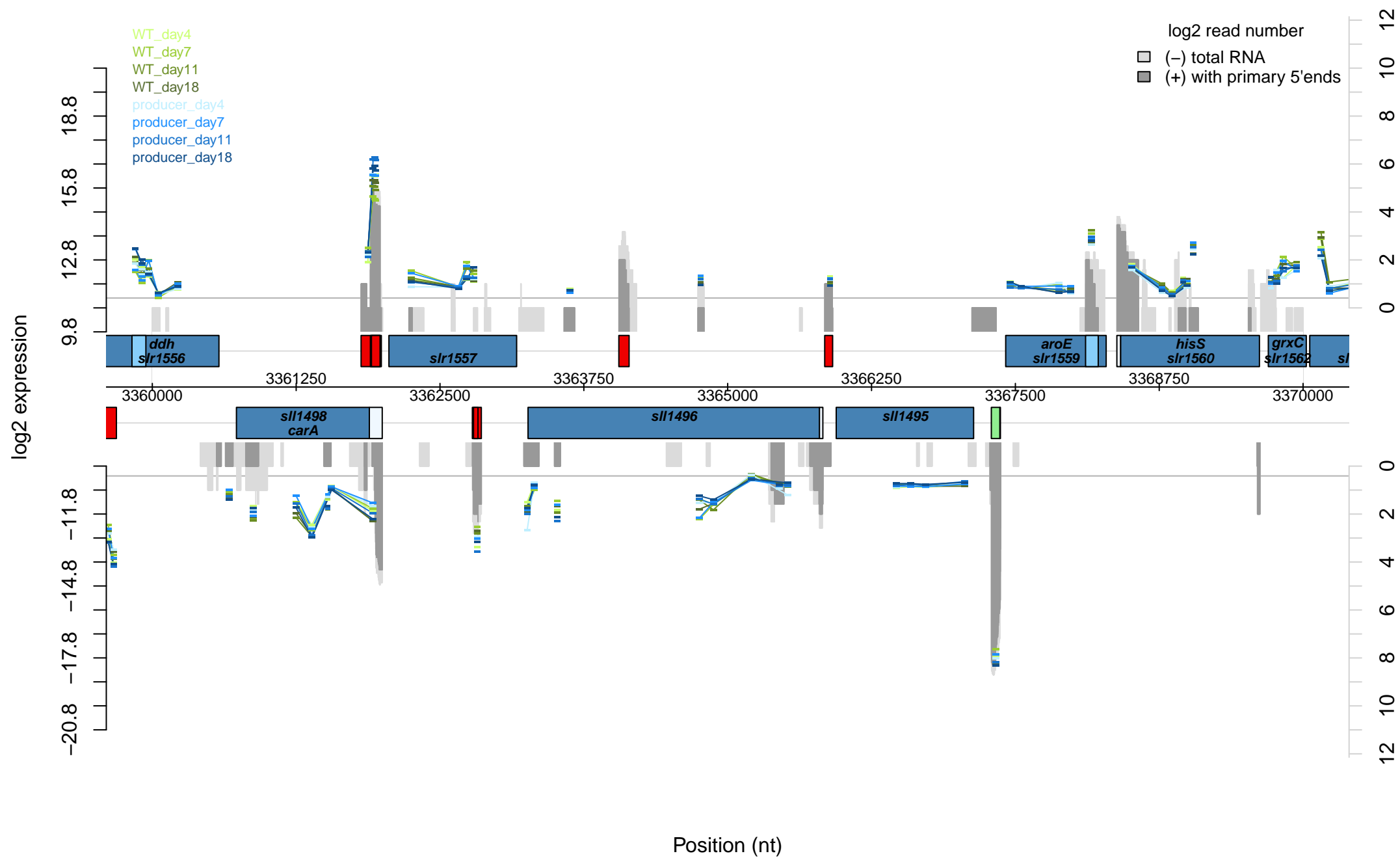

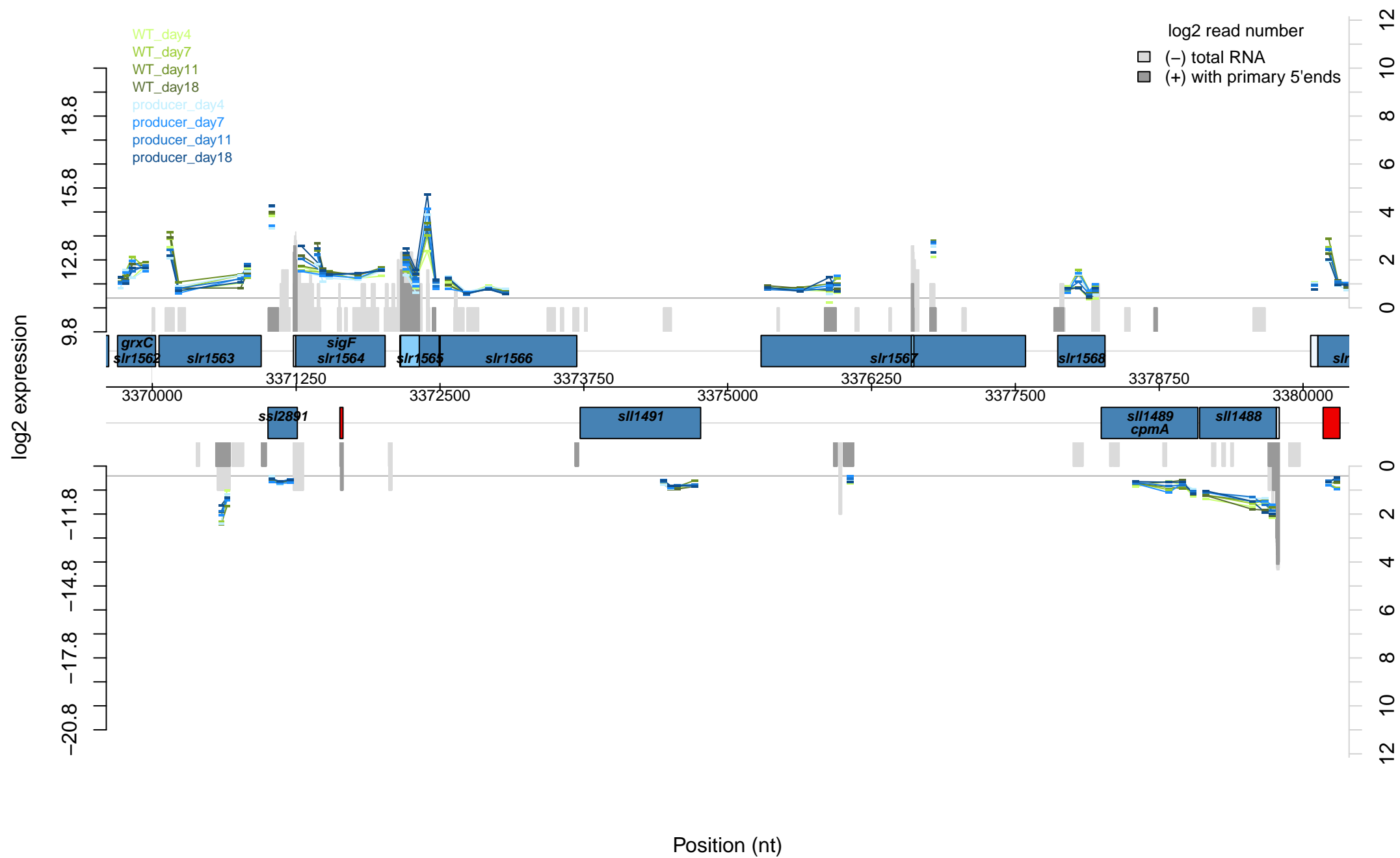

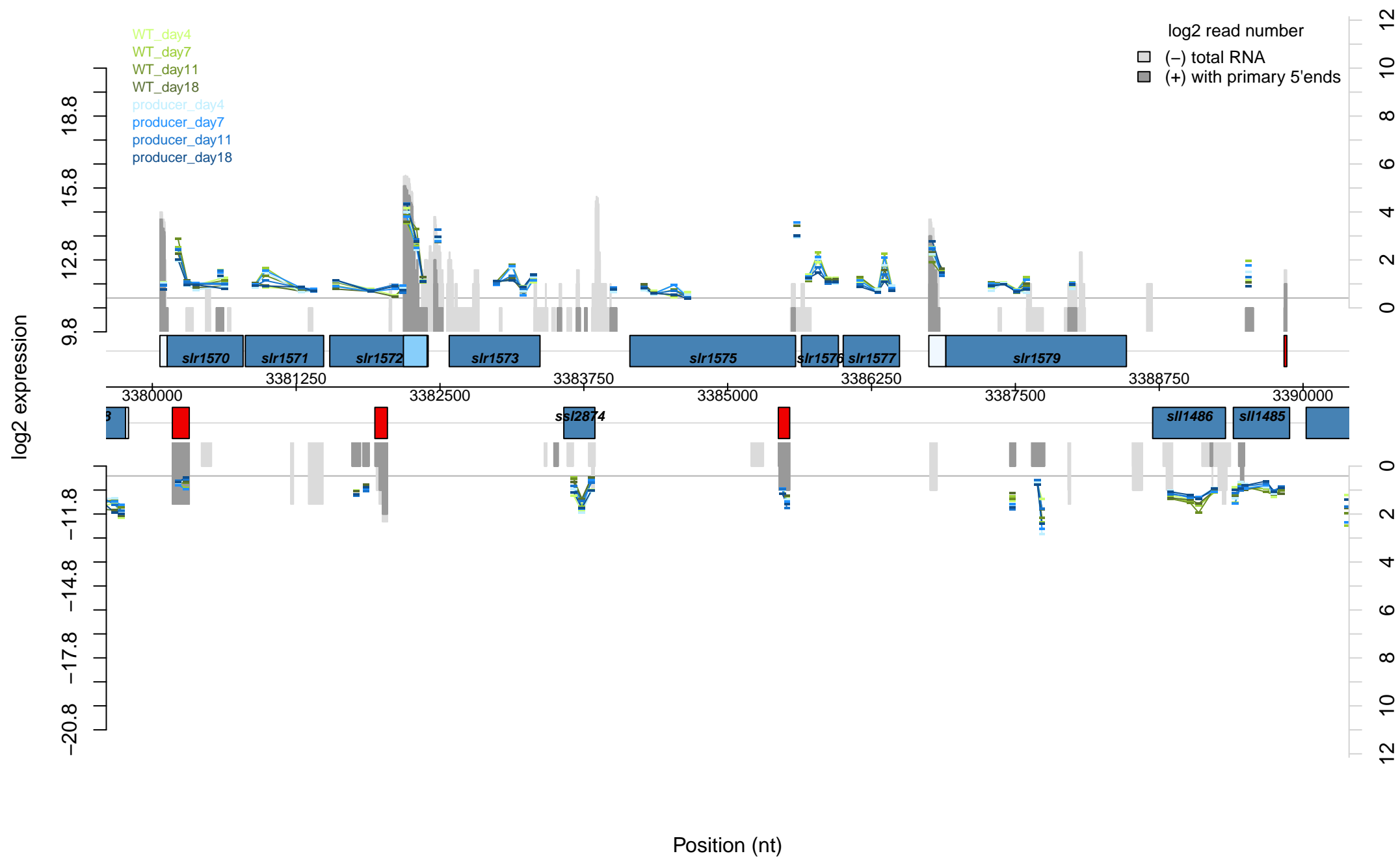

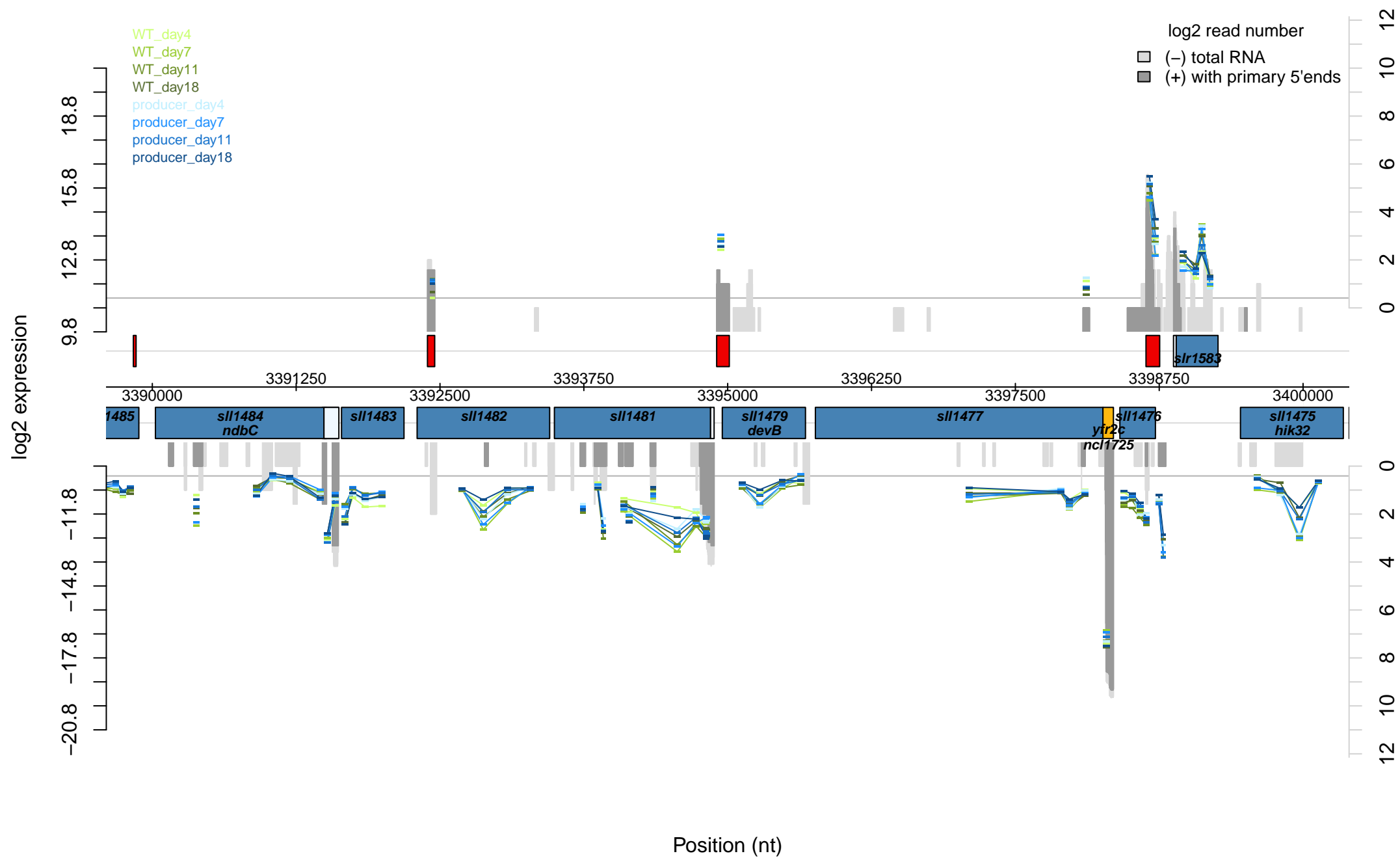

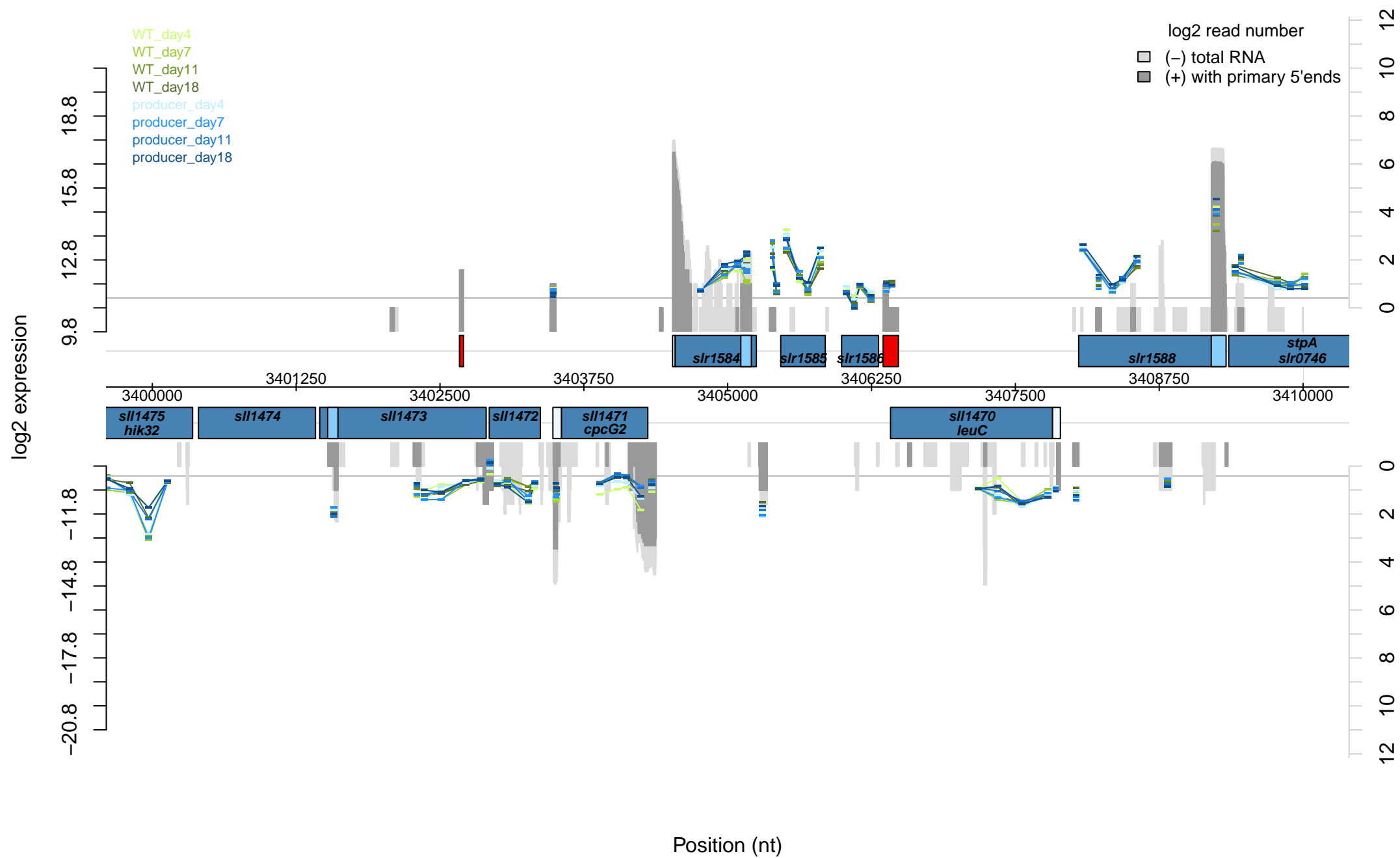

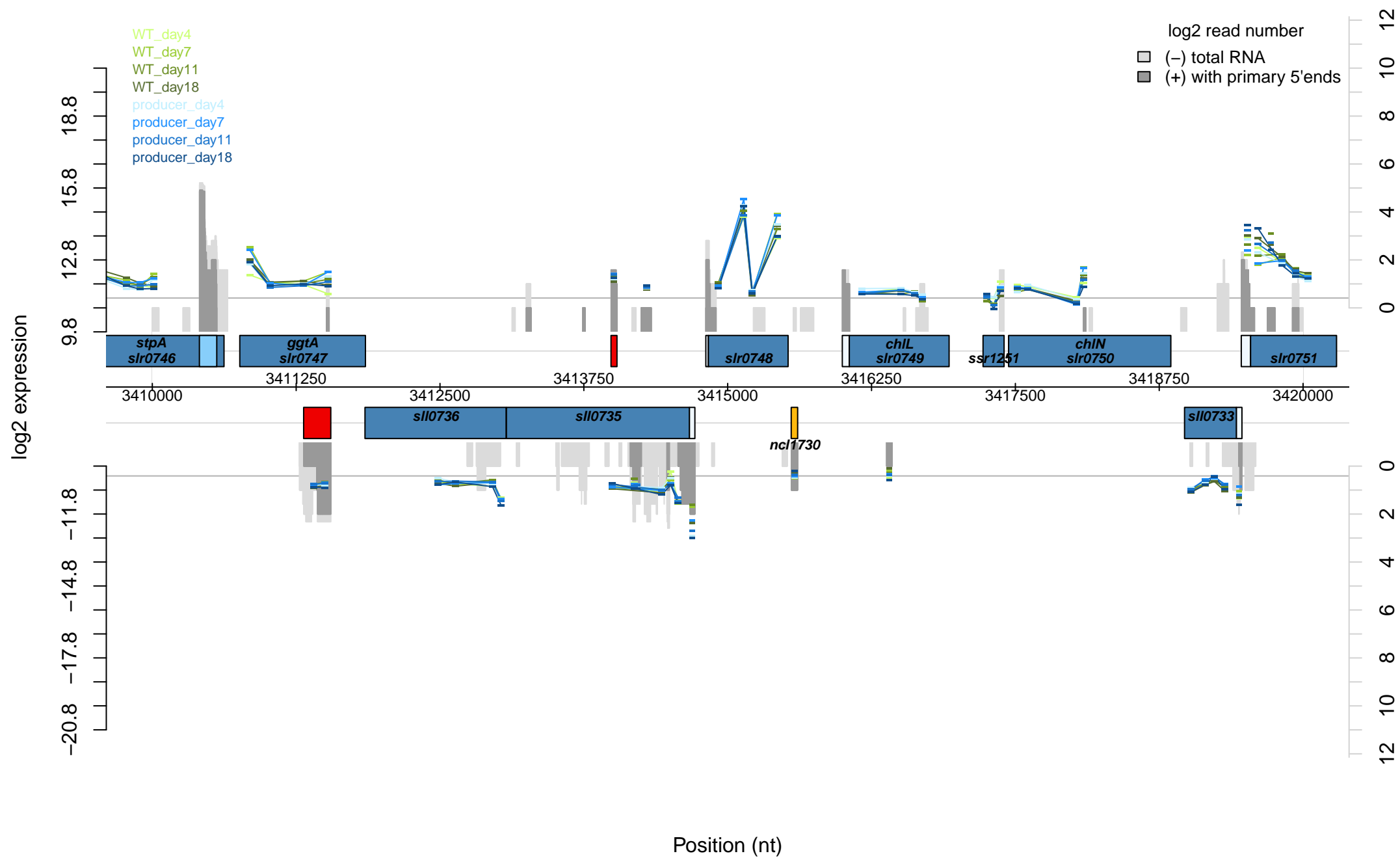



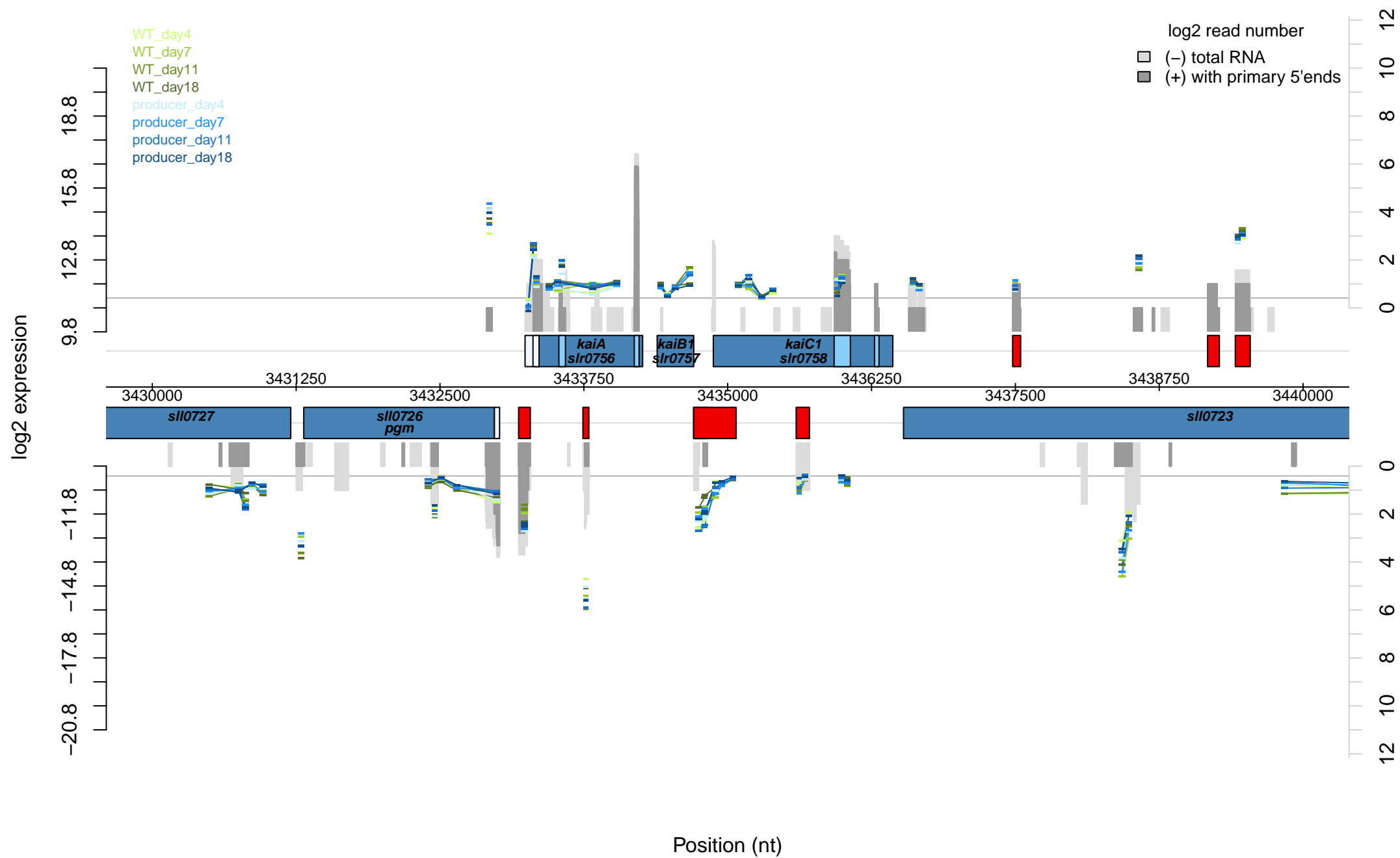

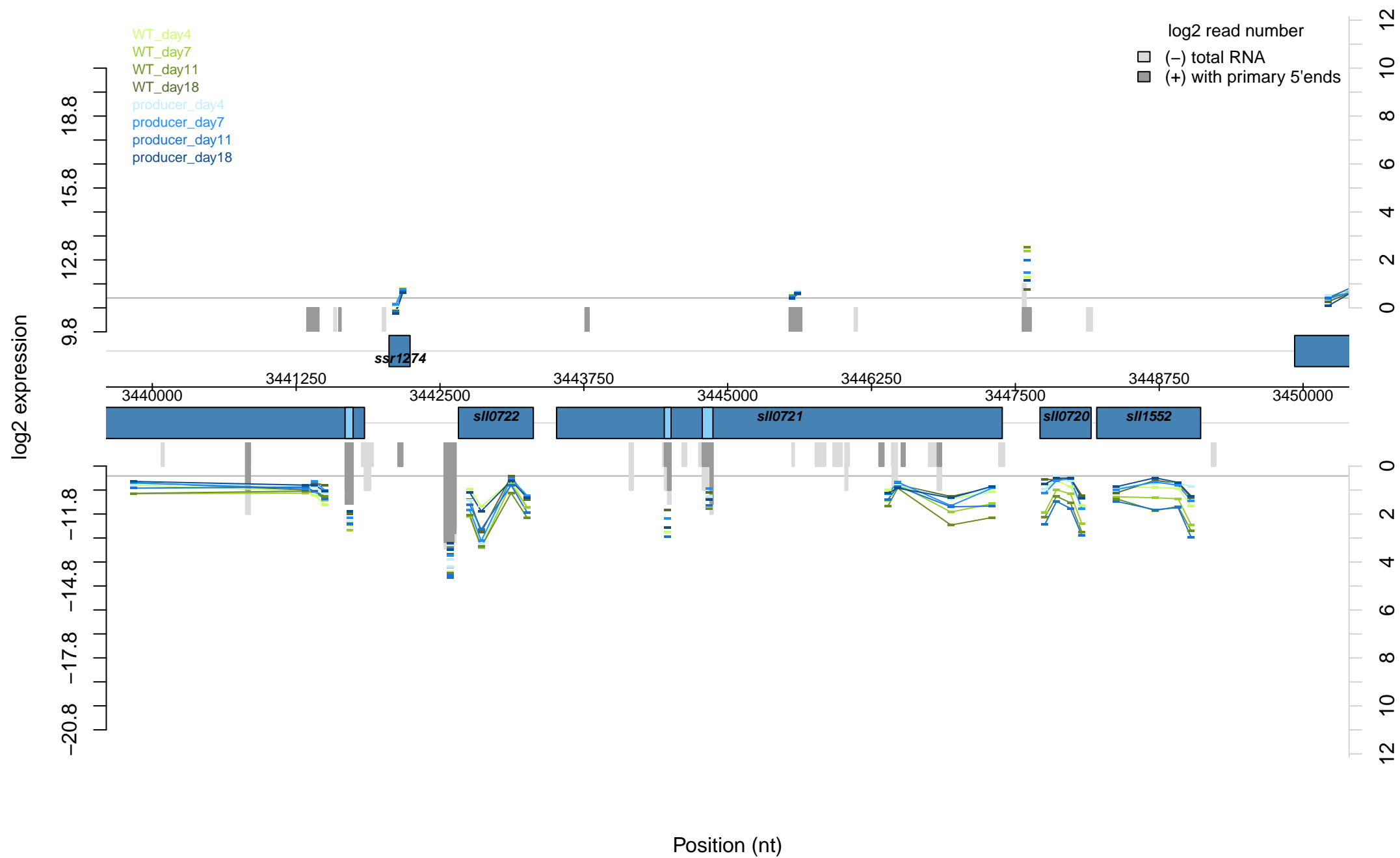

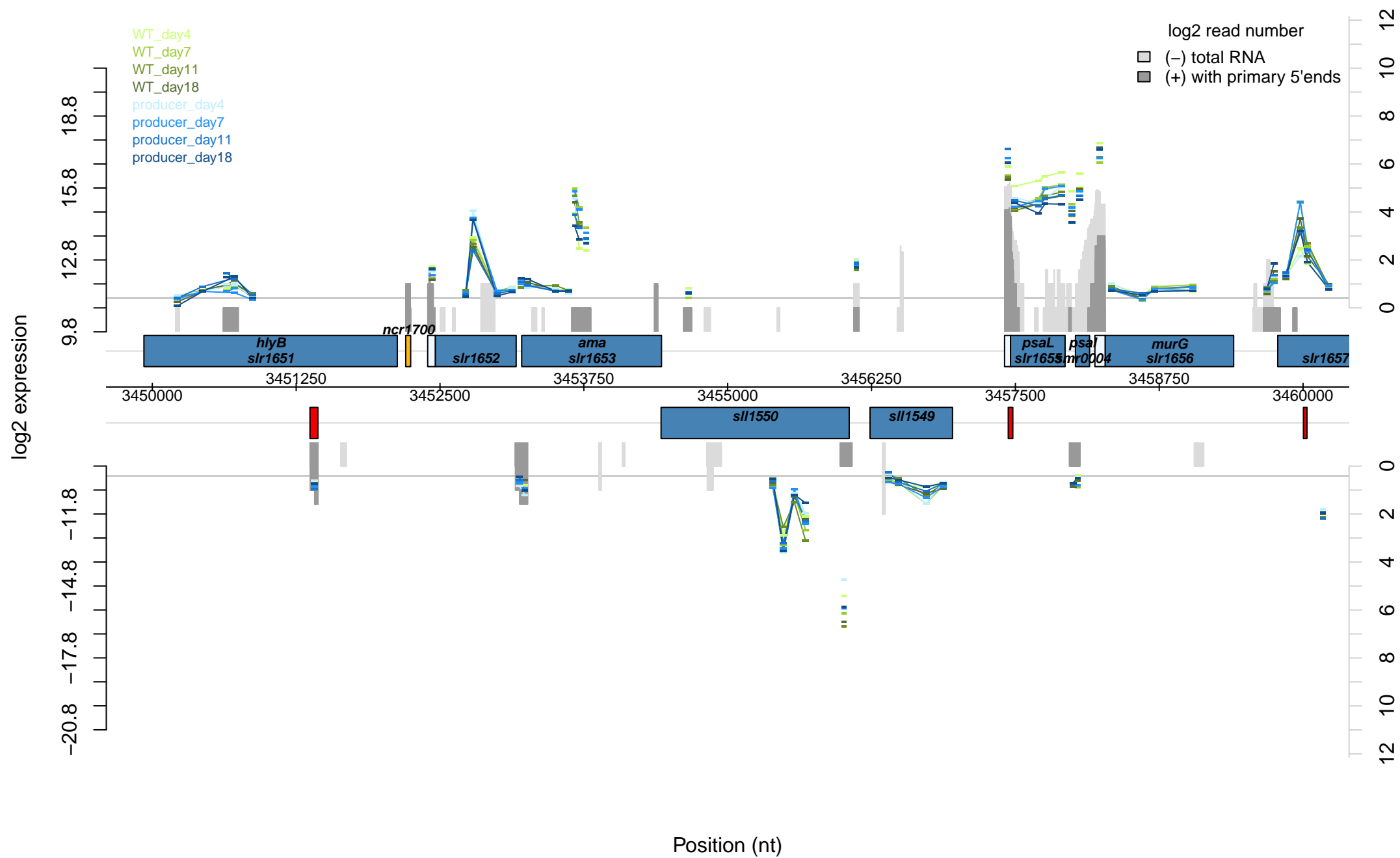

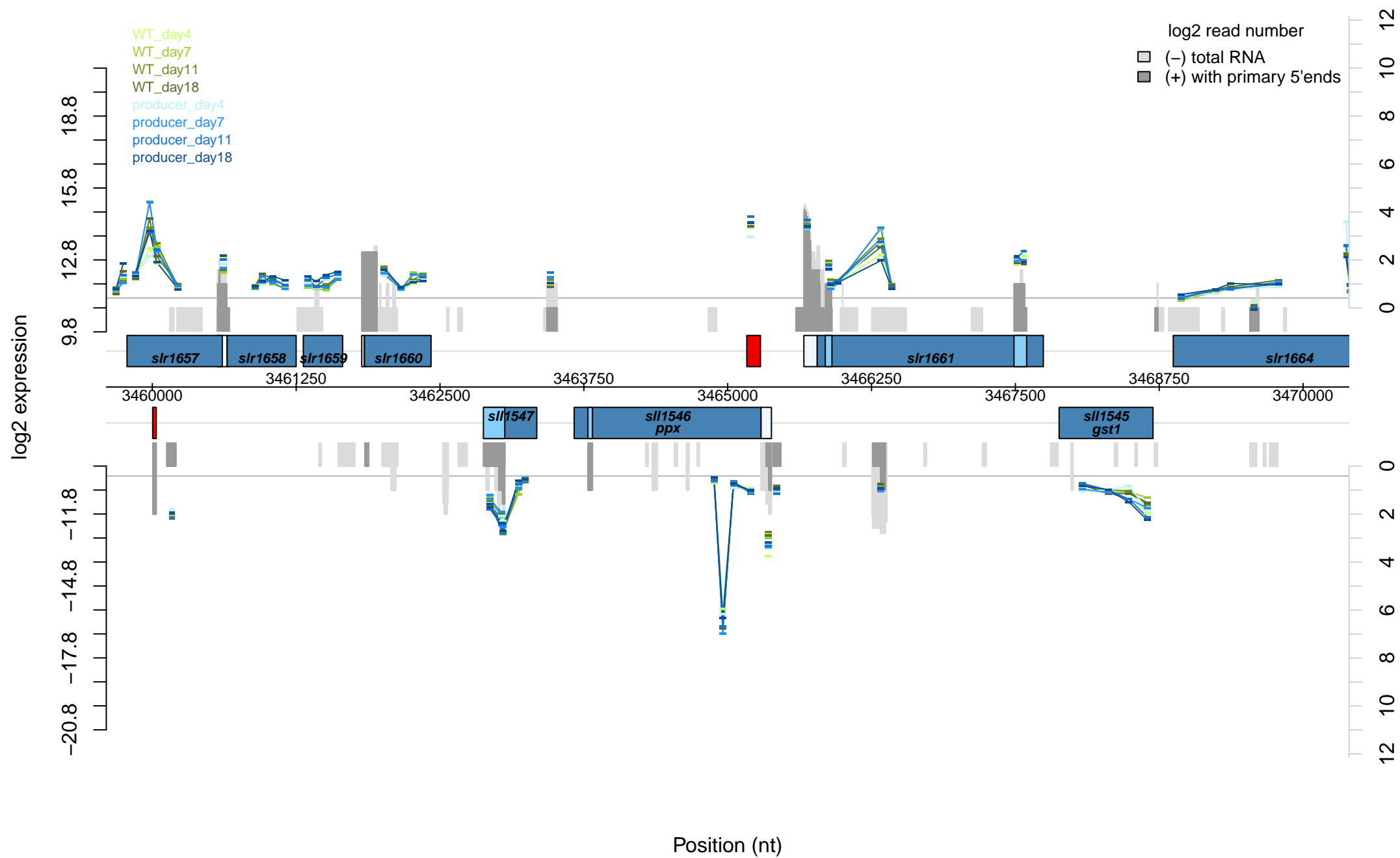

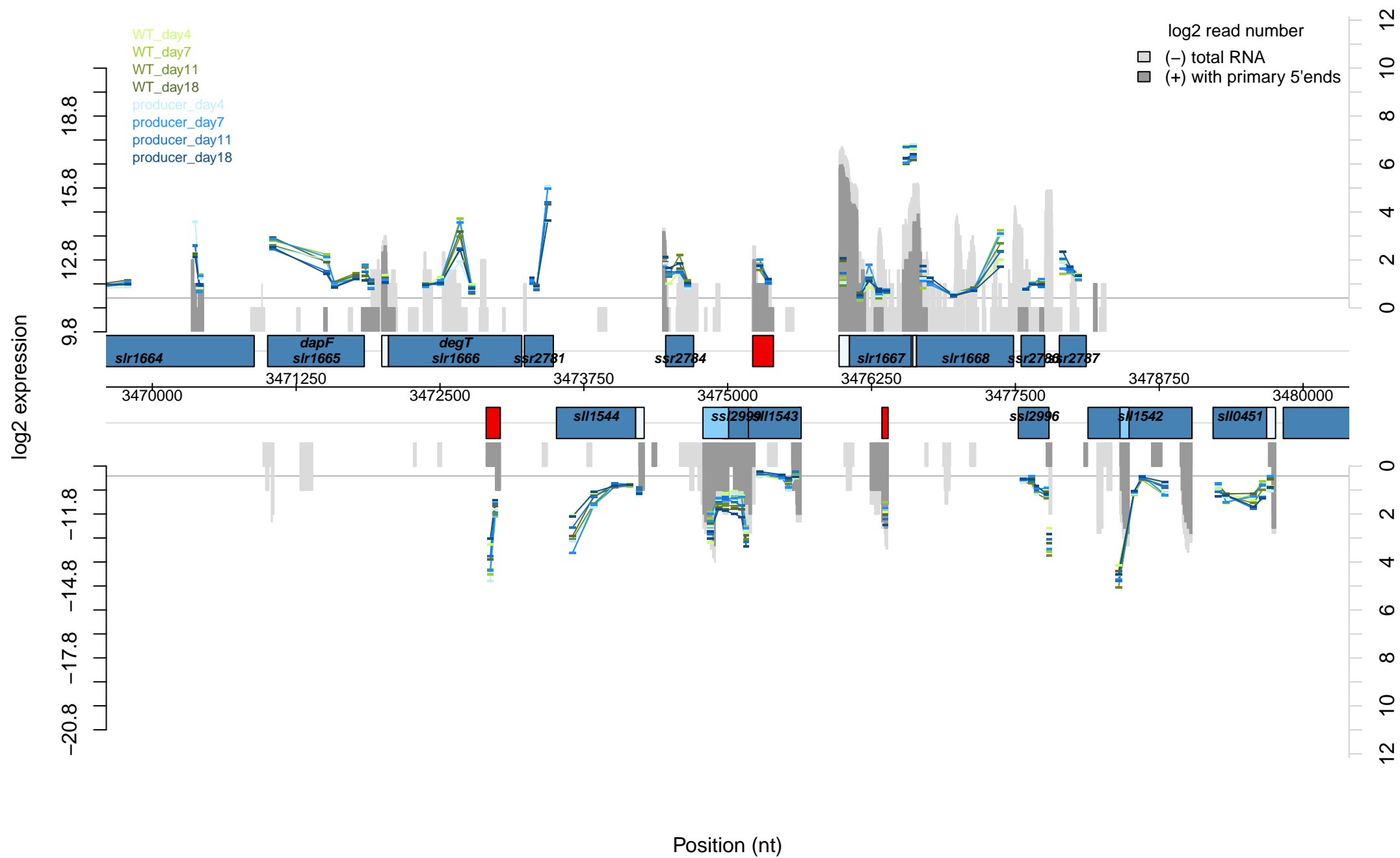



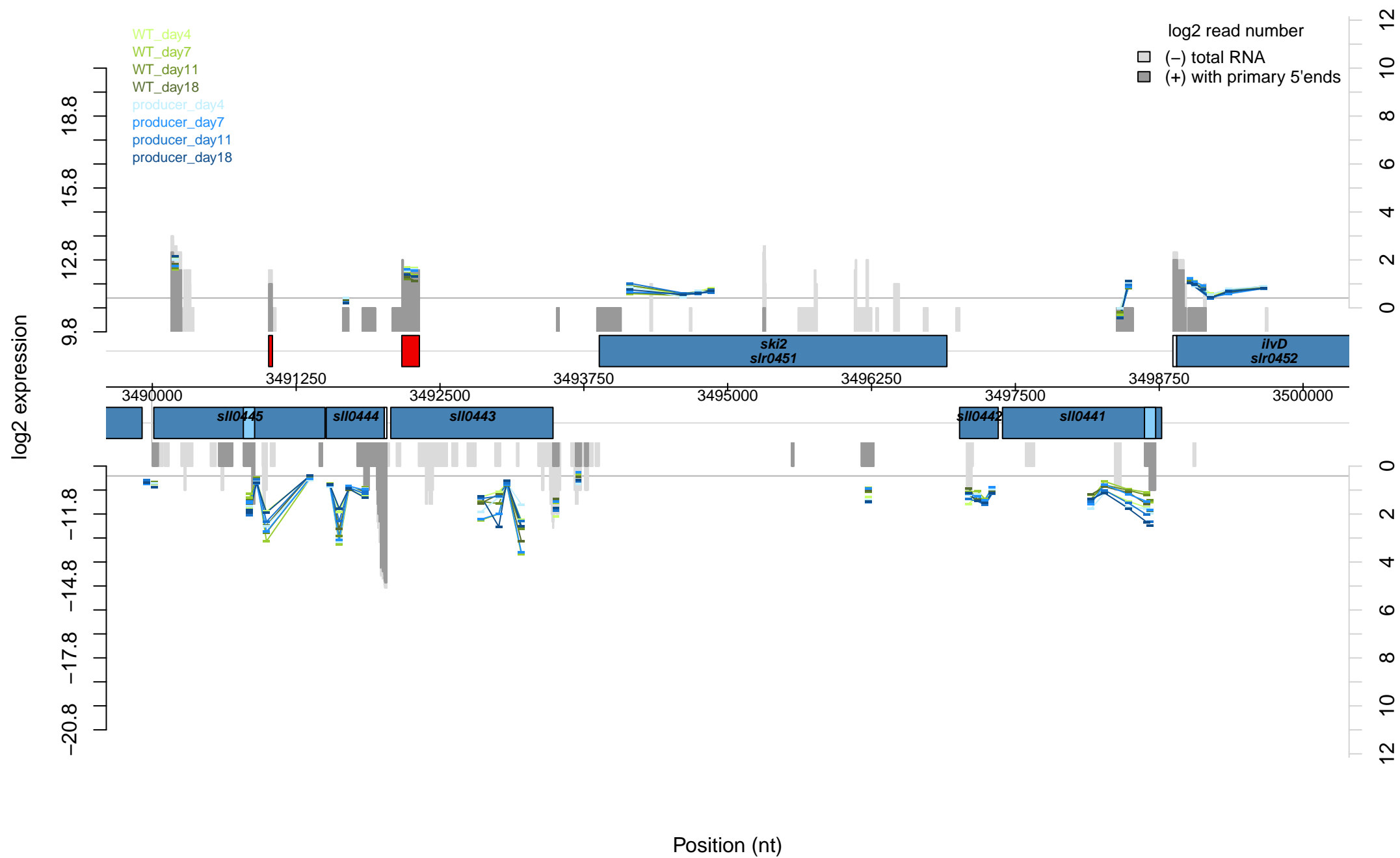

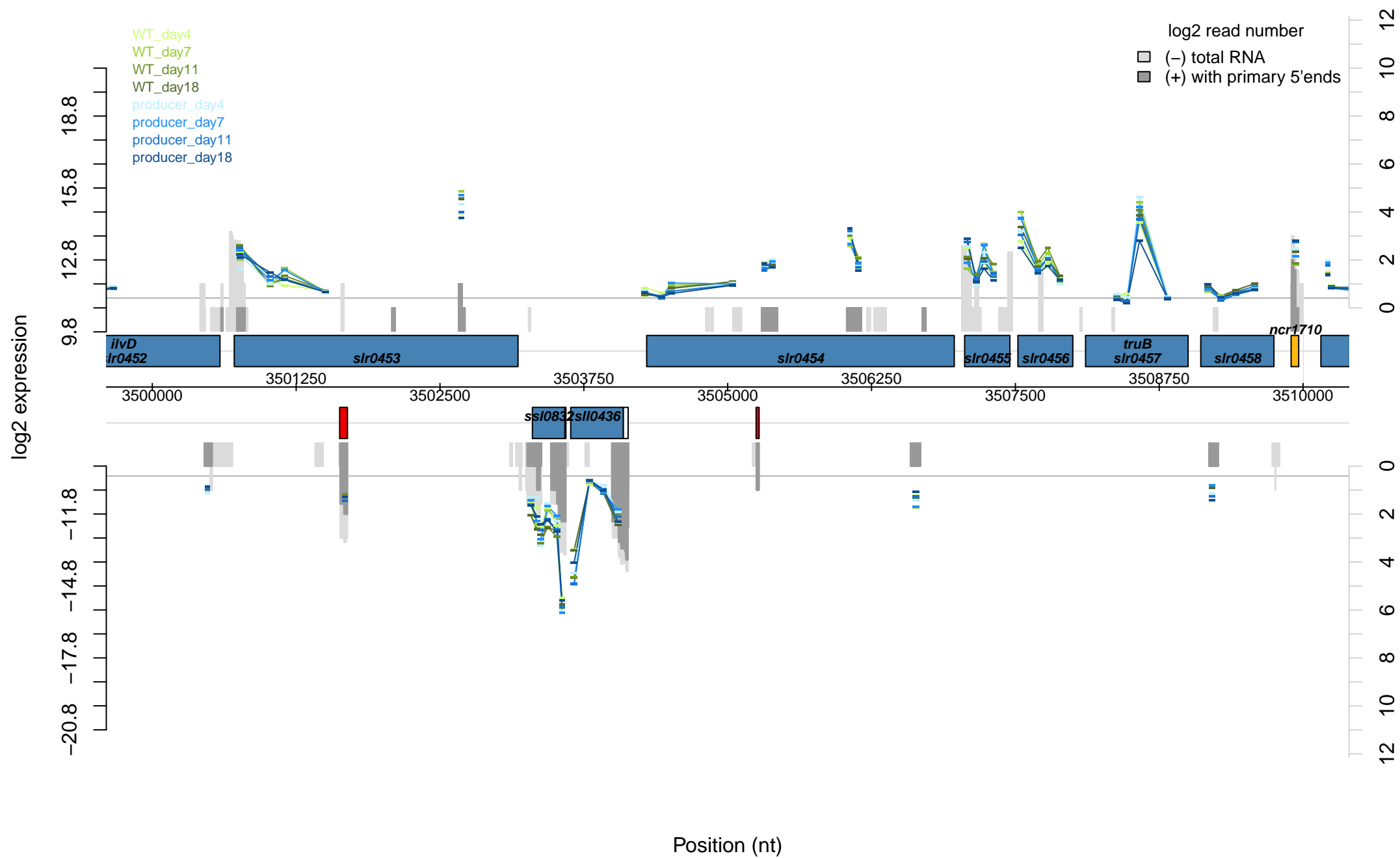

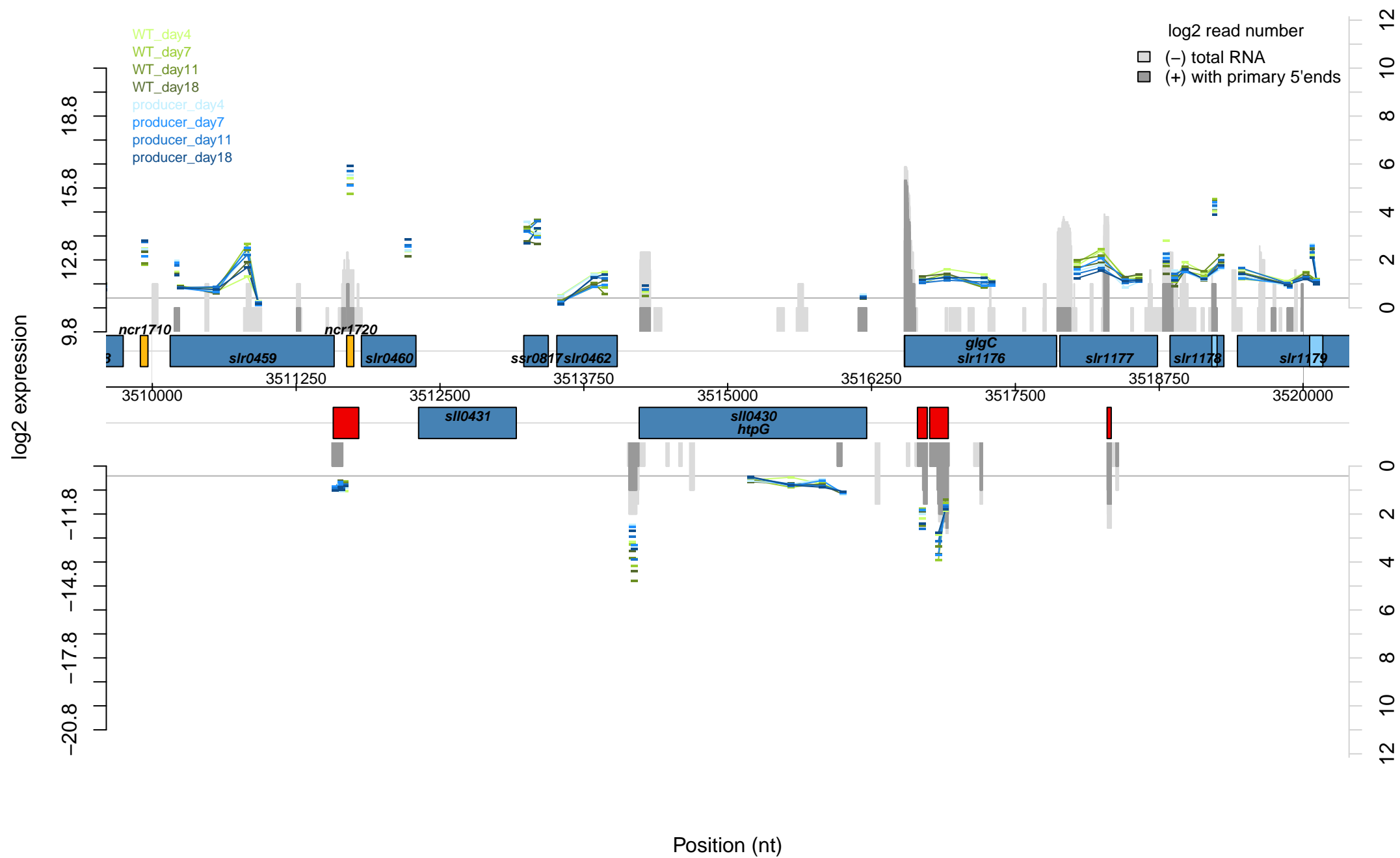

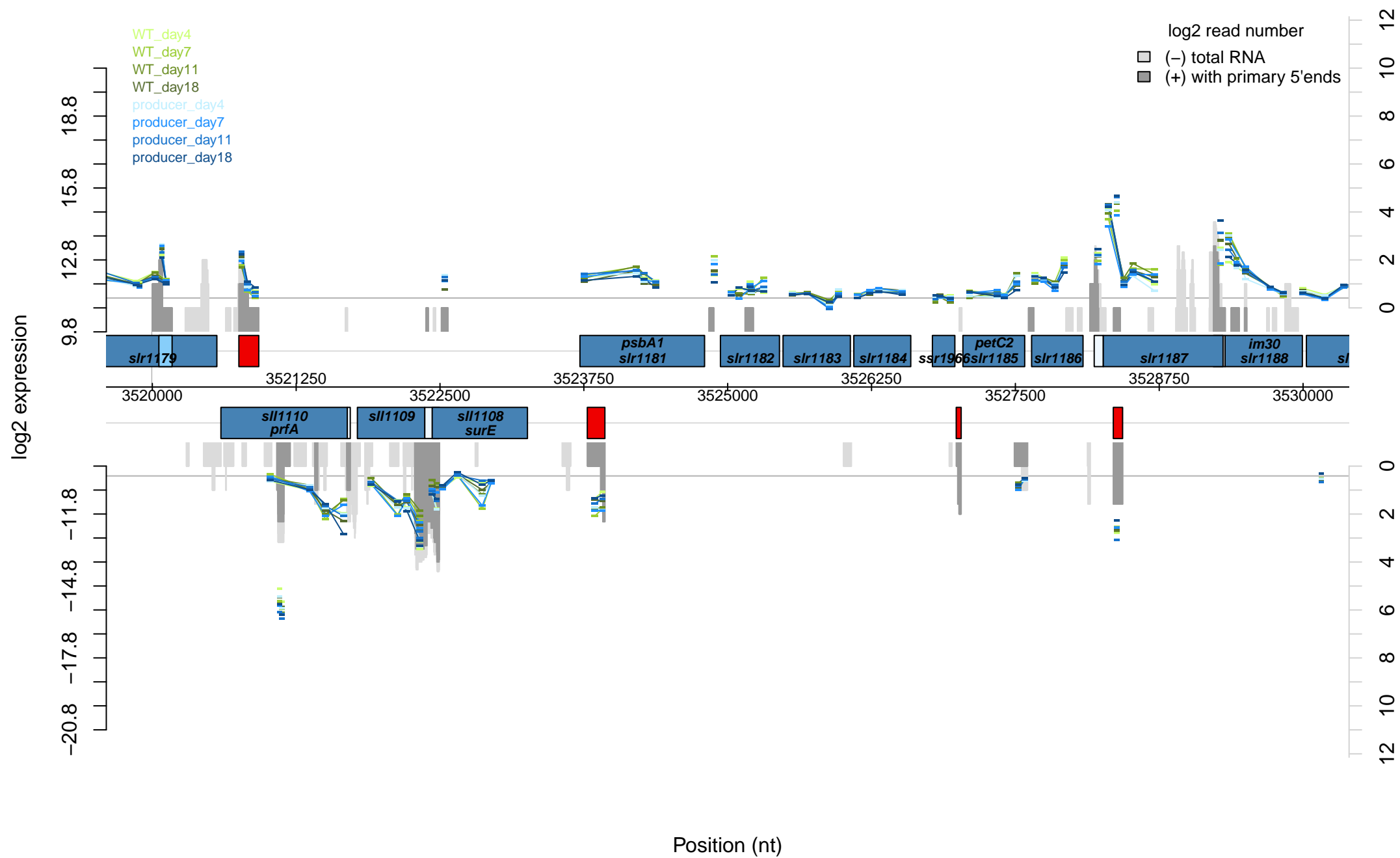

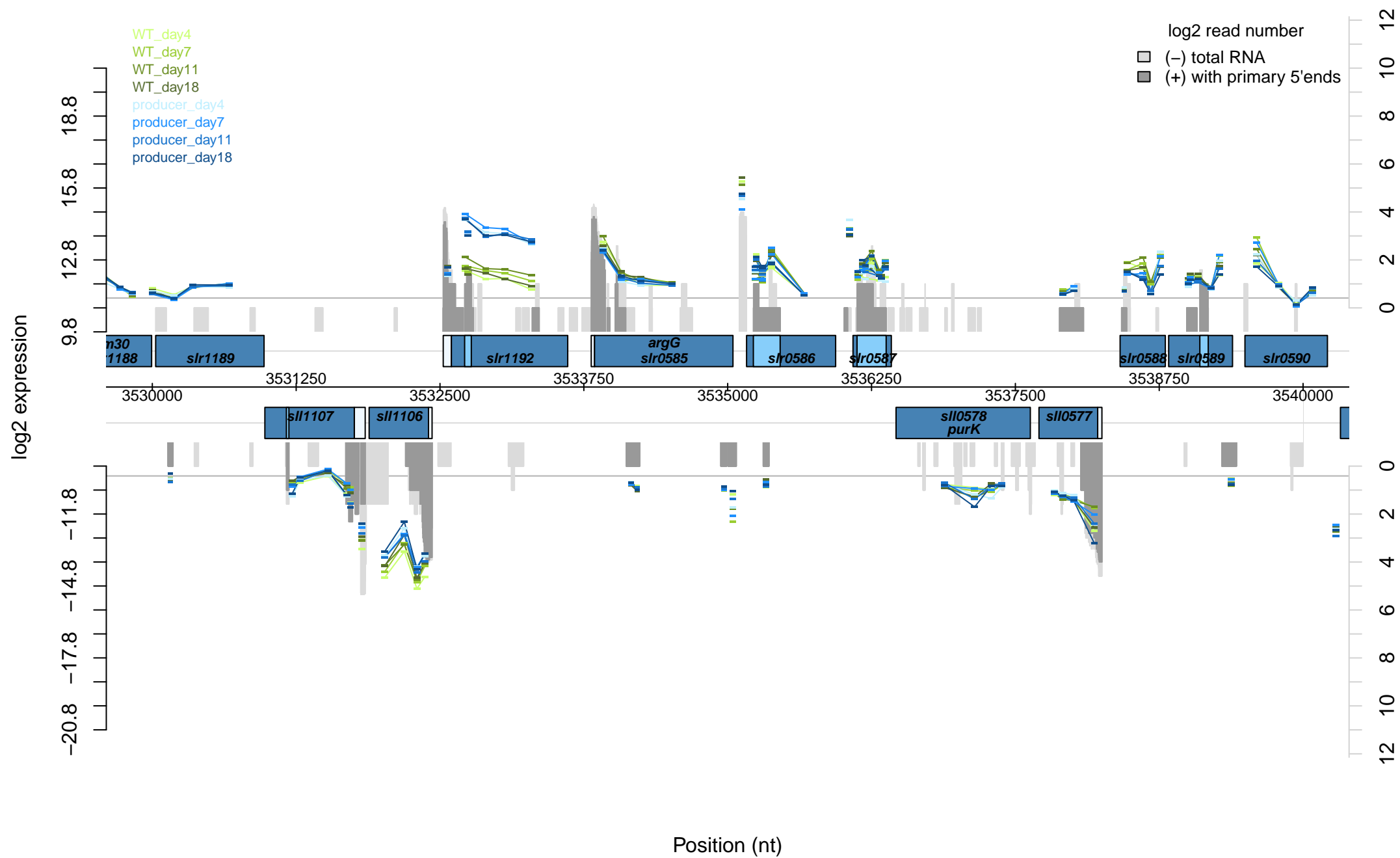

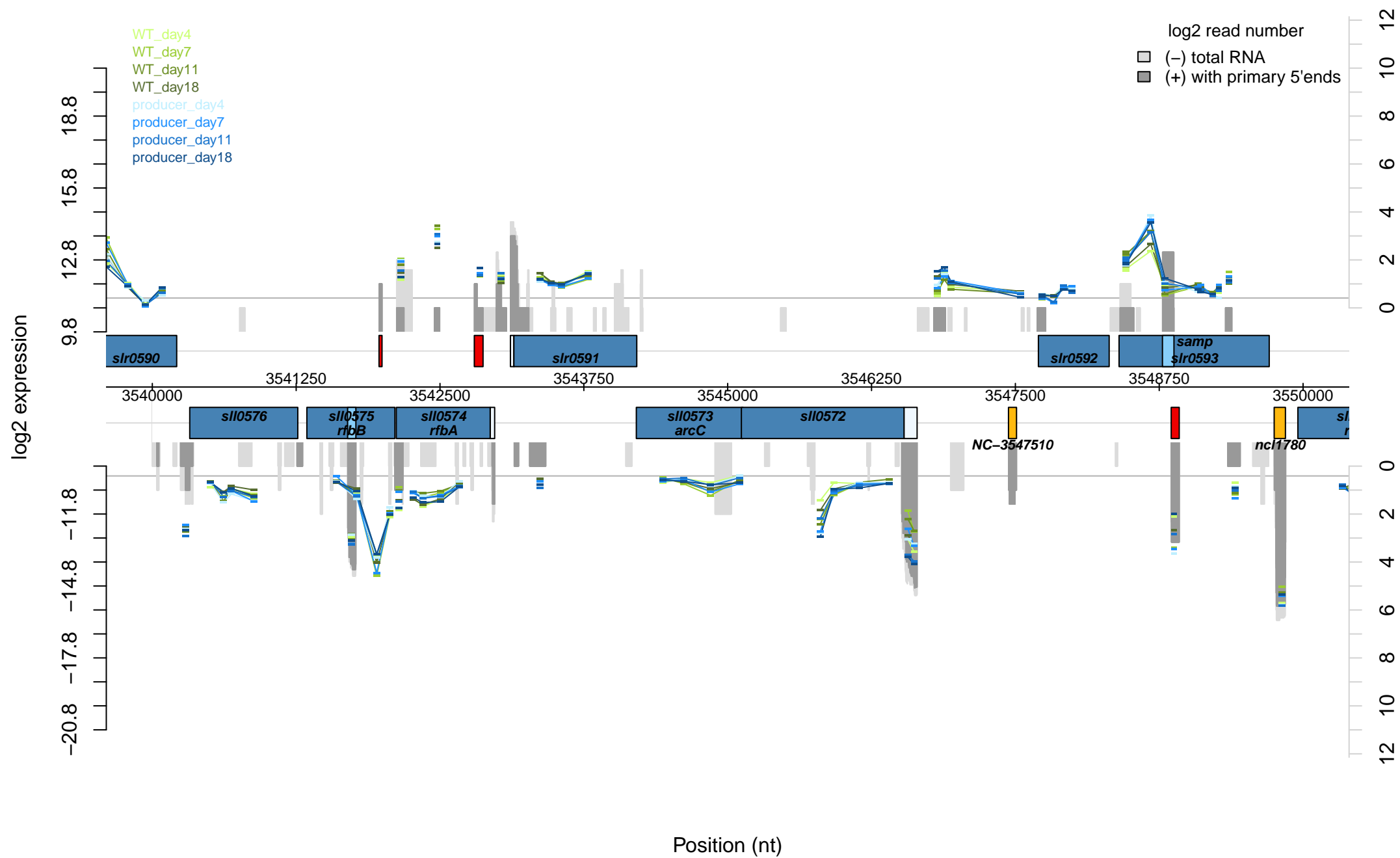

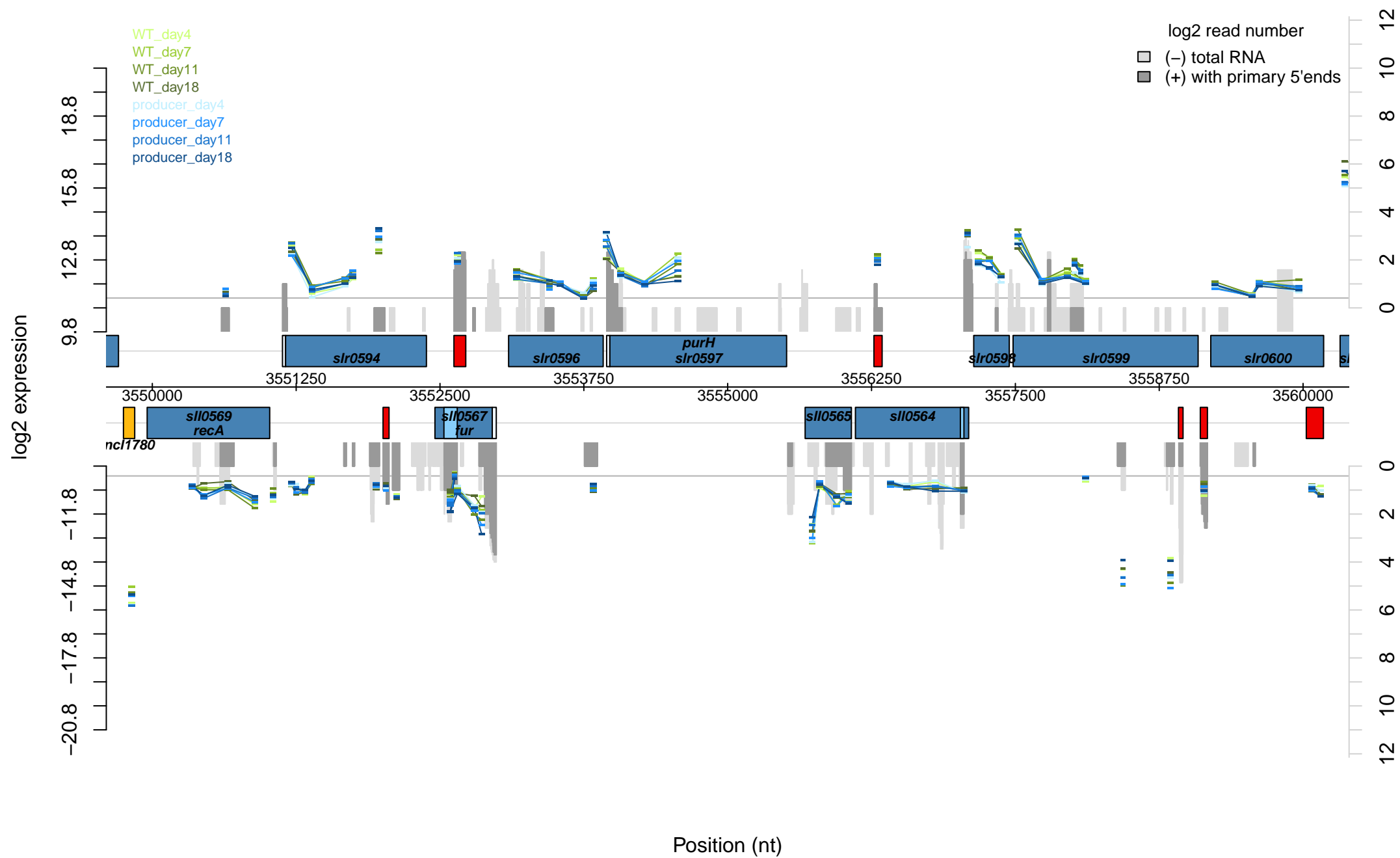

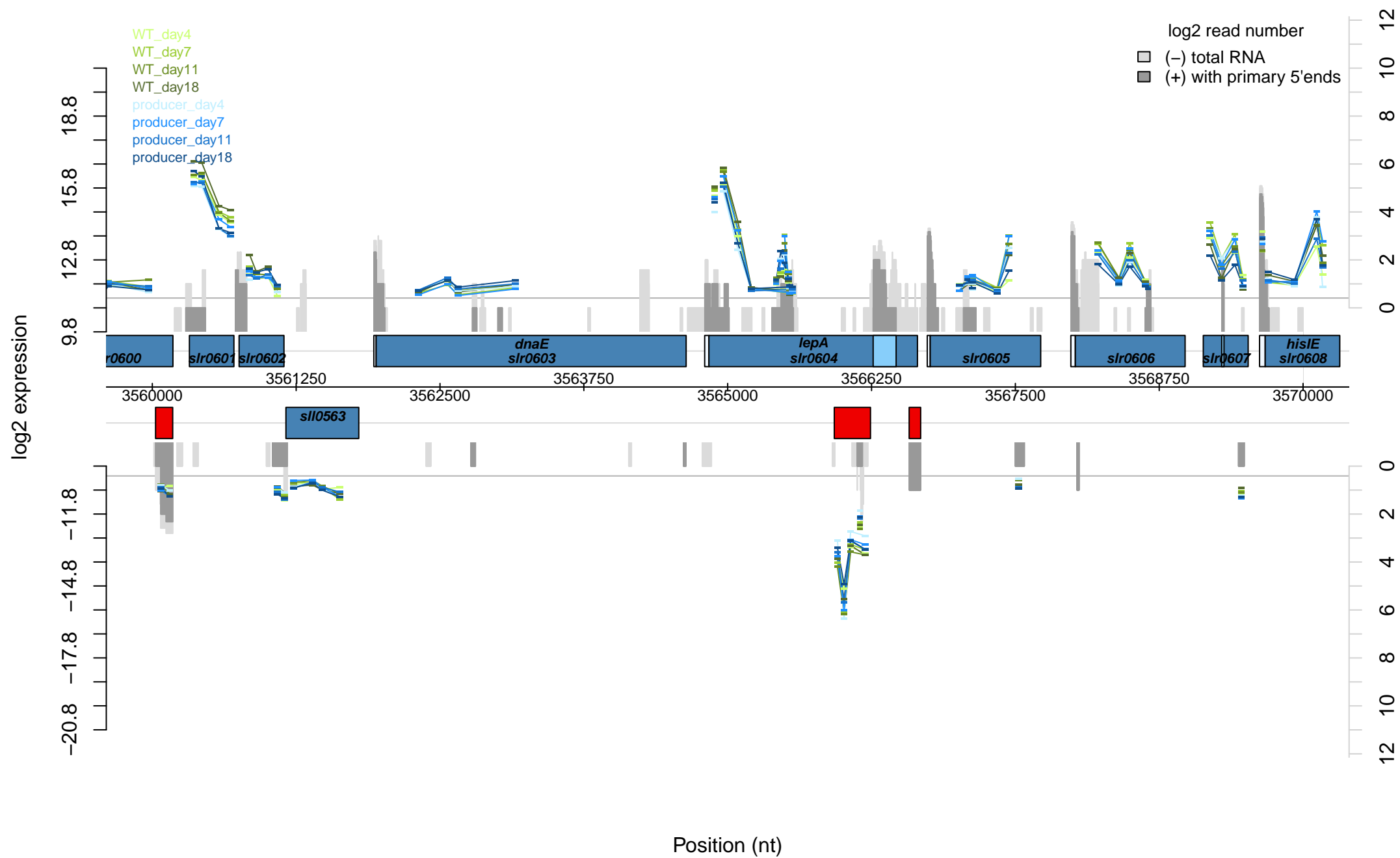

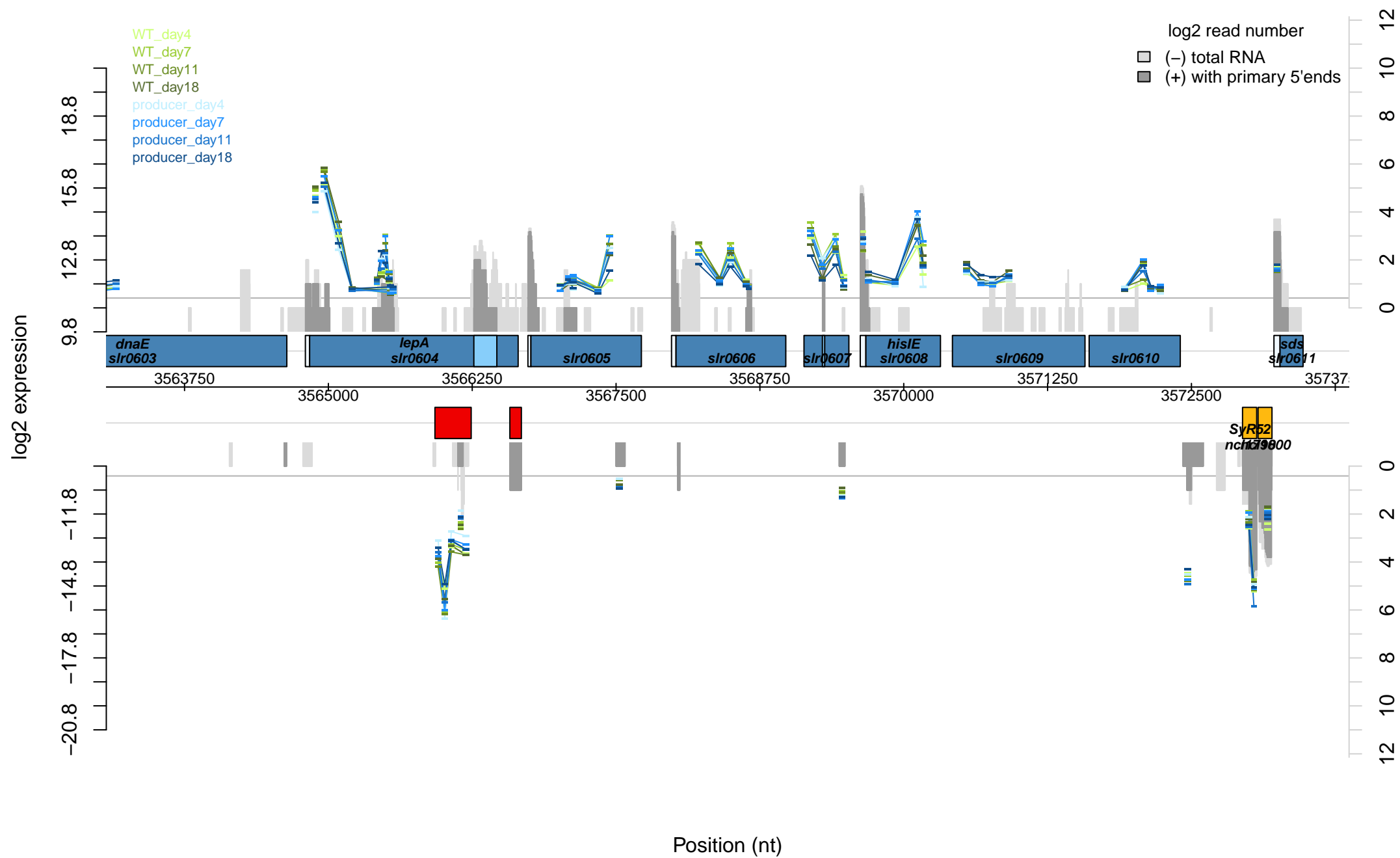

Supplement: Additional file 1 — Genome-wide overview on the log 2 -normalized expression values (left scale) from the microarray analysis of the producer strain versus control as indicated by the coloured lines. Both strands are shown with the location of annotated genes (blue boxes), 5′-UTRs (light grey), internal sense RNAs (light blue), asRNAs (red) and intergenic ncRNA genes (yellow). The normalized log2 expression values obtained by microarray analyses (normalized expression of biological duplicates A1 and A2 in two technical duplicates each) are plotted for each probe as bars in blue (producer A1 and A2, with increasing colour intensity from to to t4) or green (control incubation, with increasing colour intensity from to to t4). The scale for the microarray data is given at the left y-axis. For comparison, the numbers of RNAseq reads from previous transcriptome analyses under standard conditions [11] are plotted (dark grey, primary reads; light grey, secondary reads). [file 1754-6834-7-21-S1.pdf]
