# Supplementary material for: Transcriptomic response to prolonged ethanol production in the cyanobacterium Synechocystis sp. PCC6803
Source: Biotechnol Biofuels. 2014 Feb 6;7:21. doi: 10.1186/1754-6834-7-21 (PMC3925133; doi:10.1186/1754-6834-7-21)
Supplement: Additional file 2: Figure S1 — Ethanologenic plasmid pVZ325-PpetJ-PDC-synADH of producer strain #309. Non-ethanologenic empty-vector-control plasmid pVZ325 of control strain #621. [file 1754-6834-7-21-S2.pdf]

Additional File 2

Additional Figures

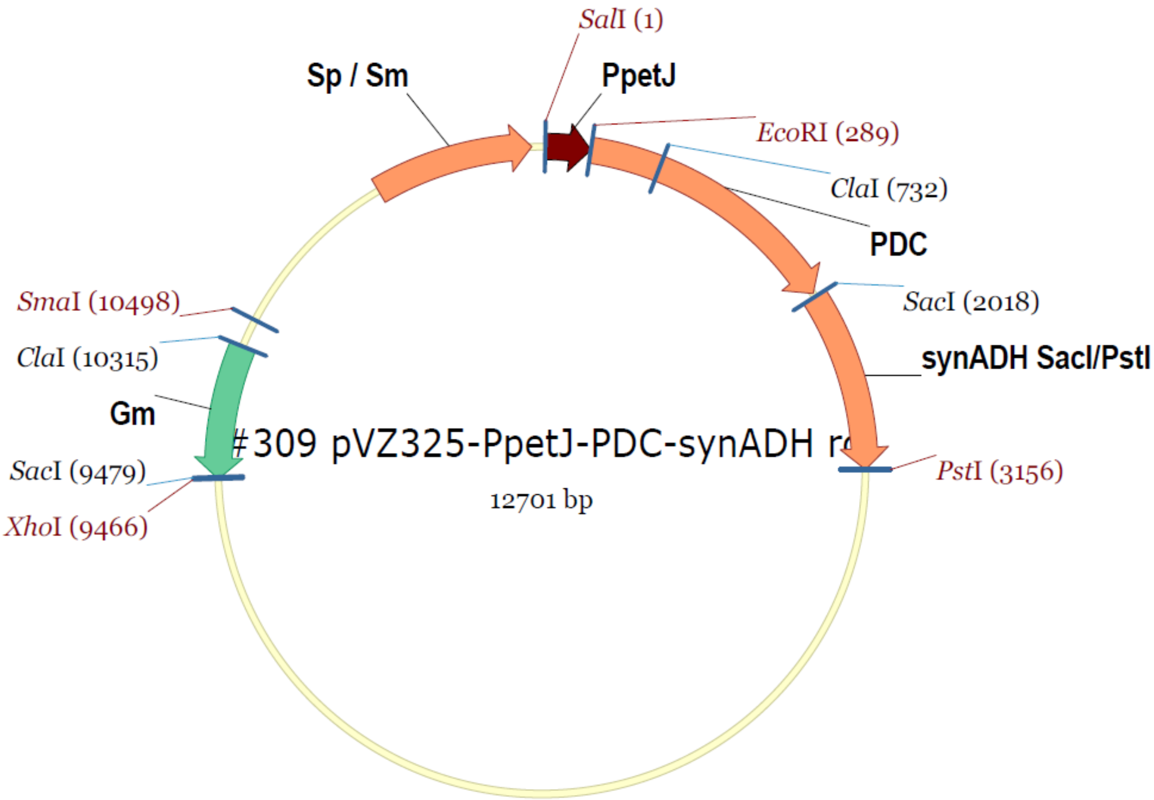

Figure 1. Ethanologenic plasmid pVZ325-PpetJ-PDC-synADH of producer strain #309.

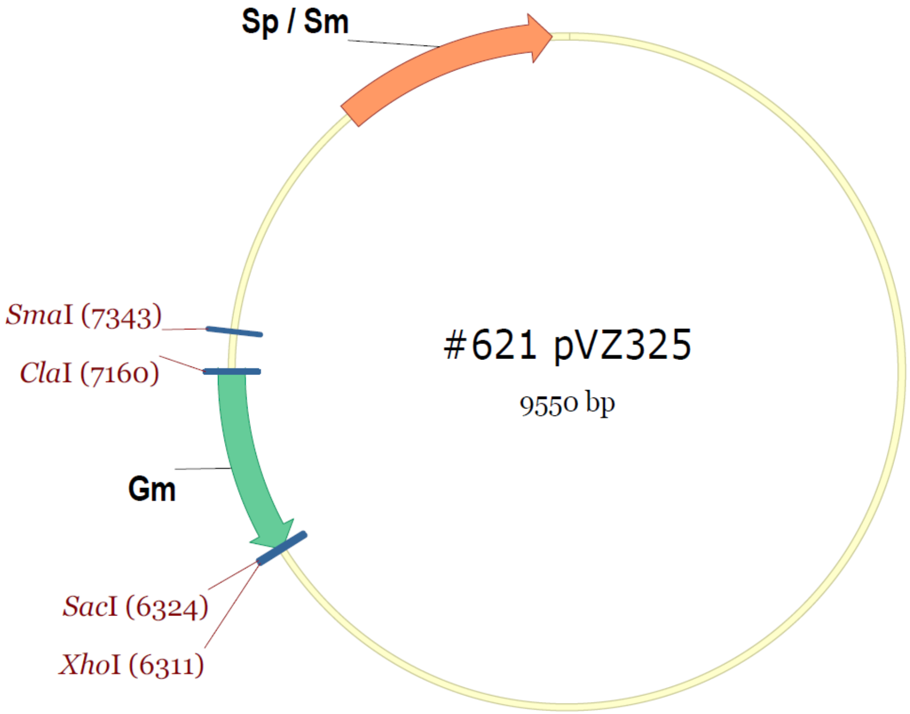

Figure 2. Non-ethanologenic empty-vector-control plasmid pVZ325 of isogenic control strain #621.
